# Supplementary material for: Empirical single-cell tracking and cell-fate simulation reveal dual roles of p53 in tumor suppression
Source: eLife. 2022 Sep 20;11:e72498. doi: 10.7554/eLife.72498 (PMC9560164; doi:10.7554/eLife.72498)
Supplement: Figure 7—source code 1. — (a) Overall scheme of single-cell tracking. (b) Processes of cell-fate simulation. [file elife-72498-fig7-code1.docx]

**(a)**

Begin:

**Create live cell imaging videos**

**Segment images**

**Perform automatic single-cell tracking and create cell-lineage database**

Verify tracking data manually

Restart automatic single-cell tracking

Record the position of each cell, an event type that occurred in a cell,

and the relation of a cell to other cells (parent and siblings)

**Display cell-lineage maps**

**Analyze tracking data**

**Create Operation data for simulation**

**Save Operation data**

End:

**(b)**

Begin:

**Upload Operation data from a saved file**

If no file was found, create Operation data from a cell-lineage database

**Set simulation parameters**

Set simulation time

Set the number of progenitors

**Create Operation arrays**

Create an array: the First event-Operation data-Time

Create an array: the First event Operation data-Event

Create arrays: the Operation data-Time for other classified event patterns

Create arrays: the Operation data-Events for other classified event patterns

**Create an array to hold simulated cell-lineage data**

**Assign the length of time and an event to progenitors**

Assign a lineage number to each progenitor

Assign cell number 0 to the progenitor

Assign the length of time to each progenitor

Generate random value

Select the length by using the First event-Operation data-Time

Assign the event type to the progenitor

Generate random value

Select the length by using the First event-Operation data-Events

**Assign the length of time and an event to progeny**

For x = simulation time

*Create a temporary array to hold cell-lineage data*

*Assign event type using relevant Operation data-Event*

Generate random value

Select event type

*Assign the length of time using relevant Operation data-Time*

Generate random value

Select the length of time

If cell fusion is assigned, check the event type of its siblings

If cell fusion is assigned to its sibling, assigning the

different length of time from one assigned to its siblings

If bipolar cell division, multipolar cell division or cell death is assigned to its sibling, assigning the

shorter length of time from one assigned to its siblings

If bipolar cell division is assigned, adjust the length of time within +- 10% of its parent cell

If bipolar or multipolar cell divisions are assigned, generate the relevant number of progeny (2 and 3 for bipolar and multipolar cell division, respectively)

Assigning new cell number

*Save assigned data into the temporary array*

*Transfer the temporarily saved data to the simulated cell-lineage data array*

If the size of simulated cell-lineage data array exceeds the allocated size, expand the array size

*Clear the temporal array*

Repeat

**Display results**

**Clear all arrays**

End:
