# Supplementary material for: Empirical single-cell tracking and cell-fate simulation reveal dual roles of p53 in tumor suppression
Source: eLife. 2022 Sep 20;11:e72498. doi: 10.7554/eLife.72498 (PMC9560164; doi:10.7554/eLife.72498)
Supplement: Figure 7—source code 2. [file elife-72498-fig7-code2.docx]

//

//  SimulationProcess.m

//  Lineage_Analysis

//

//  Created by Masahiko Sato

//

/*

 Source data setting or reading

 The following code is written in Objective-C/C++ using Xcode.

 The cell fate simulation uses up to four data arrays depending on the mode of simulation.

 getListGrowthSet, getListGrowthProgSet, getListGrowthMidSet, getListGrowthAddSet, set data to a corresponding array from cell lineage database or saved files.

 Some variables and arrays are defined as Public to use them with .xib. We mentioned the types of those variables and arrays. The following code also includimg ones that are used for a process check and monitor display.

 We wrote comments in "getListGrowthSet".

 */

-(**IBAction**)getListGrowthSet:(**id**)sender{

**if** (simulationprogress == 0){ //===(int)simulationprogress: Monitoring the progress of the simulation. If simulationprogress is 1, the process of the array set will be blocked.====

**if** (databaseLoadOptionHold == 0){ //====(int)databaseLoadOptionHold: If databaseLoadOptionHold is 0, selected cell lineage database will be used to set data to arrays. If it is 1, data will be loaded from a saved file.====

**if** (simListHoldCount != 0){ //====(int)simListHoldCount: This array holds the number of cell lineage databases loaded in the system.====

                messageStringSim = arraySimListHold [simOperationTableCurrentRow*2+1].substr(arraySimListHold [simOperationTableCurrentRow*2+1].find("-")+1); //====messageStringSim: arraySimListHold (string array) holds the names of cell lineage uploaded to the system and (int) simOperationTableCurrentRow indicates the selected row number within the arraySimListHold. messageStringSim (string) is used to display the name of the selected cell lineage database.====

                NSString *treatPickUpNameNSString = @(messageStringSim.c_str());

                [growthTreatDisplay setStringValue:treatPickUpNameNSString]; //====Display the name of the selected cell lineage database.====

                /*

                 In the system, multiple cell lineage databases are held in a 2D array ((int)**arrayLineageData). Each length of cell lineage database is held in (int)*arrayLineageDataEntryHold.

                 **********arrayLineageData (format)**********

                 1. X position

                 2. Y position

                 3. Time Point

                 4. Event Type

                 [1: First entry, 2: No event, 31: bipolar division, first entry, 32: end of cells produced by bipolar cell division, 41: tripolar division first entry, 42: end of cells produced by tripolar cell division, 51: Tetrapolar division, first entry, 52: end of cells produced by tetrapolar cell division, 6: Mitosis, 7: Cell death, 8: Out of frame, 91: Fused cell end, 92: Fused cell (be fused), 11: Impertial cell division]

                 5. Parent Cell Number/Fused cell number

                 6. Cell Number

                 7. Cell lineage Number

                 8. Fused cell lineage number

                 9. Reserve

                 */

                /*

                 In the system, names of cell lineage databases are displayed in a table, which allows to select a name by cliking. simOperationTableCurrentRow holds the row number of the selected name. Corresponding cell lineage database will be uploaded to arrayCellLineageTemp.

                 */

**int** *arrayCellLineageTemp = **new** **int** [(arrayLineageDataEntryHold [simOperationTableCurrentRow]/9)*9+100];

**int** cellLineageTempCount = 0;

**for** (**unsigned** **long** counter3 = 0; counter3 < (arrayLineageDataEntryHold [simOperationTableCurrentRow]/9)*9+100; counter3++){

                    arrayCellLineageTemp [counter3] = 0;

                }

**for** (**unsigned** **long** counter3 = 0; counter3 < arrayLineageDataEntryHold [simOperationTableCurrentRow]/9; counter3++){

                    arrayCellLineageTemp [cellLineageTempCount] = arrayLineageData [simOperationTableCurrentRow][counter3*9], cellLineageTempCount++;

                    arrayCellLineageTemp [cellLineageTempCount] = arrayLineageData [simOperationTableCurrentRow][counter3*9+1], cellLineageTempCount++;

                    arrayCellLineageTemp [cellLineageTempCount] = arrayLineageData [simOperationTableCurrentRow][counter3*9+2], cellLineageTempCount++;

                    arrayCellLineageTemp [cellLineageTempCount] = arrayLineageData [simOperationTableCurrentRow][counter3*9+3], cellLineageTempCount++;

                    arrayCellLineageTemp [cellLineageTempCount] = arrayLineageData [simOperationTableCurrentRow][counter3*9+4], cellLineageTempCount++;

                    arrayCellLineageTemp [cellLineageTempCount] = arrayLineageData [simOperationTableCurrentRow][counter3*9+5], cellLineageTempCount++;

                    arrayCellLineageTemp [cellLineageTempCount] = arrayLineageData [simOperationTableCurrentRow][counter3*9+6], cellLineageTempCount++;

                    arrayCellLineageTemp [cellLineageTempCount] = arrayLineageData [simOperationTableCurrentRow][counter3*9+7], cellLineageTempCount++;

                    arrayCellLineageTemp [cellLineageTempCount] = arrayLineageData [simOperationTableCurrentRow][counter3*9+8], cellLineageTempCount++;

                }

                /*

                 In the case that multiple cells fusion occured on a cell (Event 92), we keep only the last cell fusion point and remove others. The following code serches Event 92 and retain only the last one.

                 */

**for** (**int** counter3 = 0; counter3 < cellLineageTempCount/9; counter3++){

**if** (arrayCellLineageTemp [counter3*9+3] == 92){

**for** (**int** counter4 = counter3-1; counter4 >= 0; counter4--){

**if** (arrayCellLineageTemp [counter4*9+3] == 92) arrayCellLineageTemp [counter4*9+3] = 2;

**else** **if** (arrayCellLineageTemp [counter4*9+3] == 1 || arrayCellLineageTemp [counter4*9+3] == 31 || arrayCellLineageTemp [counter4*9+3] == 41 || arrayCellLineageTemp [counter4*9+3] == 51){

**break**;

                            }

                        }

                    }

                }

**int** timePointMax = arrayTableDetail [simOperationTableCurrentRow][3]; //====Set the end of tracking time. The system holds the information of each cell lineage database in the arrayTableDetail array (string).====

**int** twentyPercentOfMax = timePointMax-(**int**)(round(timePointMax*(**double**)(recoveryCalcPercentHold/(**double**)100))); //====Defalut of recoveryCalcPercentHold is 20 (20%).====

                //====Following arrays are used to hold the length of time to a specific events.====

**int** *dataCDOccurence = **new** **int** [timePointMax+10];

**int** *dataNonDivCDOccurence = **new** **int** [timePointMax+10];

**int** *dataBDCFOccurence = **new** **int** [timePointMax+10];

**int** *dataBDCFCDOccurence = **new** **int** [timePointMax+10];

**int** *dataTDCFOccurence = **new** **int** [timePointMax+10];

**int** *dataTDCFCDOccurence = **new** **int** [timePointMax+10];

**int** *dataTDCDOccurence = **new** **int** [timePointMax+10];

**int** *dataVariationToTheFirstDV = **new** **int** [timePointMax+10];

**for** (**int** counter3 = 0; counter3 < timePointMax+10; counter3++){

                    dataCDOccurence [counter3] = 0;

                    dataNonDivCDOccurence [counter3] = 0;

                    dataBDCFOccurence [counter3] = 0;

                    dataBDCFCDOccurence [counter3] = 0;

                    dataTDCFOccurence [counter3] = 0;

                    dataTDCFCDOccurence [counter3] = 0;

                    dataTDCDOccurence [counter3] = 0;

                    dataVariationToTheFirstDV [counter3] = 0;

                }

                //====Following variables are used to hold the number of each categorized event.====

**int** dataBD = 0;

**int** dataTD = 0;

**int** dataBDCFBD = 0;

**int** dataBDCFTD = 0;

**int** dataTDCFBD = 0;

**int** dataTDCFTD = 0;

**int** dataTDBD = 0;

**int** dataTDTD = 0;

**int** nonDivLing = 0;

**int** recoveryGR = 0;

**int** dataCD = 0;

                //====Thse variables hold average time length of BD or CD.====

**double** dataBDRand = 0;

**double** dataCDRand = 0;

                /*

                 In the paper, we defined tripolar (TD) and tetrapolar cell division as MD. As tetrapolar cell division occures less frequenty, in the simulation, only TD will be geerated.

                 */

                //====Set data to arrays or variables====

**for** (**int** counter3 = 0; counter3 < cellLineageTempCount/9; counter3++){

**if** (arrayCellLineageTemp [counter3*9+3] == 32){ //====BD====

                        dataBD++;

                        dataBDRand = dataBDRand+arrayCellLineageTemp [counter3*9+2];

                    }

**if** (arrayCellLineageTemp [counter3*9+3] == 42 || arrayCellLineageTemp [counter3*9+3] == 52) dataTD++; //====TD====

**if** (arrayCellLineageTemp [counter3*9+3] == 7){ //====BD-CD====

**for** (**int** counter4 = counter3; counter4 >= 0; counter4--){

**if** (arrayCellLineageTemp [counter4*9+3] == 31){

                                dataCDOccurence [counter3-counter4]++;

**break**;

                            }

**else** **if** (arrayCellLineageTemp [counter4*9+3] == 1 || arrayCellLineageTemp [counter4*9+3] == 41 || arrayCellLineageTemp [counter4*9+3] == 51 || arrayCellLineageTemp [counter4*9+3] == 92){

**break**;

                            }

                        }

                        dataCD++;

                        dataCDRand = dataCDRand+arrayCellLineageTemp [counter3*9+2];

                    }

**if** (arrayCellLineageTemp [counter3*9+3] == 7){ //====NonDiv-CD====

**for** (**int** counter4 = counter3; counter4 >= 0; counter4--){

**if** (arrayCellLineageTemp [counter4*9+3] == 1){

                                dataNonDivCDOccurence [counter3-counter4]++;

**break**;

                            }

**else** **if** (arrayCellLineageTemp [counter4*9+3] == 31 || arrayCellLineageTemp [counter4*9+3] == 41 || arrayCellLineageTemp [counter4*9+3] == 51 || arrayCellLineageTemp [counter4*9+3] == 92){

**break**;

                            }

                        }

                    }

**if** (arrayCellLineageTemp [counter3*9+3] == 91){ //====BD-CF====

**for** (**int** counter4 = counter3; counter4 >= 0; counter4--){

**if** (arrayCellLineageTemp [counter4*9+3] == 31){

                                dataBDCFOccurence [counter3-counter4]++;

**break**;

                            }

**else** **if** (arrayCellLineageTemp [counter4*9+3] == 1 || arrayCellLineageTemp [counter4*9+3] == 41 || arrayCellLineageTemp [counter4*9+3] == 51 || arrayCellLineageTemp [counter4*9+3] == 92){

**break**;

                            }

                        }

                    }

**if** (arrayCellLineageTemp [counter3*9+3] == 92){ //====BD-CF-BD====

**for** (**int** counter4 = counter3; counter4 < cellLineageTempCount/9; counter4++){

**if** (arrayCellLineageTemp [counter4*9+3] == 32){

**for** (**int** counter5 = counter3; counter5 >= 0; counter5--){

**if** (arrayCellLineageTemp [counter5*9+3] == 31){

                                        dataBDCFBD++;

**break**;

                                    }

**else** **if** (arrayCellLineageTemp [counter5*9+3] == 1 || arrayCellLineageTemp [counter5*9+3] == 41 || arrayCellLineageTemp [counter5*9+3] == 51){

**break**;

                                    }

                                }

**break**;

                            }

**else** **if** ((arrayCellLineageTemp [counter3*9+5] != arrayCellLineageTemp [counter4*9+5] || arrayCellLineageTemp [counter3*9+6] != arrayCellLineageTemp [counter4*9+6]) || arrayCellLineageTemp [counter4*9+3] == 8 || arrayCellLineageTemp [counter4*9+3] == 42 || arrayCellLineageTemp [counter4*9+3] == 52 || arrayCellLineageTemp [counter4*9+3] == 91 || arrayCellLineageTemp [counter4*9+2] == timePointMax){

**break**;

                            }

                        }

                    }

**if** (arrayCellLineageTemp [counter3*9+3] == 92){ //====BD-CF-TD====

**for** (**int** counter4 = counter3; counter4 < cellLineageTempCount/9; counter4++){

**if** (arrayCellLineageTemp [counter4*9+3] == 42 || arrayCellLineageTemp [counter4*9+3] == 52){

**for** (**int** counter5 = counter3; counter5 >= 0; counter5--){

**if** (arrayCellLineageTemp [counter5*9+3] == 31){

                                        dataBDCFTD++;

**break**;

                                    }

**else** **if** (arrayCellLineageTemp [counter5*9+3] == 1 || arrayCellLineageTemp [counter5*9+3] == 41 || arrayCellLineageTemp [counter5*9+3] == 51){

**break**;

                                    }

                                }

**break**;

                            }

**else** **if** ((arrayCellLineageTemp [counter3*9+5] != arrayCellLineageTemp [counter4*9+5] || arrayCellLineageTemp [counter3*9+6] != arrayCellLineageTemp [counter4*9+6]) || arrayCellLineageTemp [counter4*9+3] == 8 || arrayCellLineageTemp [counter4*9+3] == 32 || arrayCellLineageTemp [counter4*9+3] == 91 || arrayCellLineageTemp [counter4*9+2] == timePointMax){

**break**;

                            }

                        }

                    }

**if** (arrayCellLineageTemp [counter3*9+3] == 92){ //====BD-CF-CD (from CF)====

**for** (**int** counter4 = counter3; counter4 < cellLineageTempCount/9; counter4++){

**if** (arrayCellLineageTemp [counter4*9+3] == 7){

**for** (**int** counter5 = counter3; counter5 >= 0; counter5--){

**if** (arrayCellLineageTemp [counter5*9+3] == 31){

                                        dataBDCFCDOccurence [counter4-counter3]++;

**break**;

                                    }

**else** **if** (arrayCellLineageTemp [counter5*9+3] == 1 || arrayCellLineageTemp [counter5*9+3] == 41 || arrayCellLineageTemp [counter5*9+3] == 51){

**break**;

                                    }

                                }

**break**;

                            }

**else** **if** ((arrayCellLineageTemp [counter3*9+5] != arrayCellLineageTemp [counter4*9+5] || arrayCellLineageTemp [counter3*9+6] != arrayCellLineageTemp [counter4*9+6]) || arrayCellLineageTemp [counter4*9+3] == 8 || arrayCellLineageTemp [counter4*9+3] == 32 || arrayCellLineageTemp [counter4*9+3] == 42 || arrayCellLineageTemp [counter4*9+3] == 52 || arrayCellLineageTemp [counter4*9+3] == 91 || arrayCellLineageTemp [counter4*9+2] == timePointMax){

**break**;

                            }

                        }

                    }

**if** (arrayCellLineageTemp [counter3*9+3] == 91){ //====TD-CF====

**for** (**int** counter4 = counter3; counter4 >= 0; counter4--){

**if** (arrayCellLineageTemp [counter4*9+3] == 41 || arrayCellLineageTemp [counter4*9+3] == 51){

                                dataTDCFOccurence [counter3-counter4]++;

**break**;

                            }

**else** **if** (arrayCellLineageTemp [counter4*9+3] == 1 || arrayCellLineageTemp [counter4*9+3] == 31 || arrayCellLineageTemp [counter4*9+3] == 92){

**break**;

                            }

                        }

                    }

**if** (arrayCellLineageTemp [counter3*9+3] == 92){ //====TD-CF-BD====

**for** (**int** counter4 = counter3; counter4 < cellLineageTempCount/9; counter4++){

**if** (arrayCellLineageTemp [counter4*9+3] == 32){

**for** (**int** counter5 = counter3; counter5 >= 0; counter5--){

**if** (arrayCellLineageTemp [counter5*9+3] == 41 || arrayCellLineageTemp [counter5*9+3] == 51){

                                        dataTDCFBD++;

**break**;

                                    }

**else** **if** (arrayCellLineageTemp [counter5*9+3] == 1 || arrayCellLineageTemp [counter5*9+3] == 31){

**break**;

                                    }

                                }

**break**;

                            }

**else** **if** ((arrayCellLineageTemp [counter3*9+5] != arrayCellLineageTemp [counter4*9+5] || arrayCellLineageTemp [counter3*9+6] != arrayCellLineageTemp [counter4*9+6]) || arrayCellLineageTemp [counter4*9+3] == 8 || arrayCellLineageTemp [counter4*9+3] == 42 || arrayCellLineageTemp [counter4*9+3] == 52 || arrayCellLineageTemp [counter4*9+3] == 91 || arrayCellLineageTemp [counter4*9+2] == timePointMax){

**break**;

                            }

                        }

                    }

**if** (arrayCellLineageTemp [counter3*9+3] == 92){ //====TD-CF-TD====

**for** (**int** counter4 = counter3; counter4 < cellLineageTempCount/9; counter4++){

**if** (arrayCellLineageTemp [counter4*9+3] == 42 || arrayCellLineageTemp [counter4*9+3] == 52){

**for** (**int** counter5 = counter3; counter5 >= 0; counter5--){

**if** (arrayCellLineageTemp [counter5*9+3] == 41 || arrayCellLineageTemp [counter5*9+3] == 51){

                                        dataTDCFTD++;

**break**;

                                    }

**else** **if** (arrayCellLineageTemp [counter5*9+3] == 1 || arrayCellLineageTemp [counter5*9+3] == 31){

**break**;

                                    }

                                }

**break**;

                            }

**else** **if** ((arrayCellLineageTemp [counter3*9+5] != arrayCellLineageTemp [counter4*9+5] || arrayCellLineageTemp [counter3*9+6] != arrayCellLineageTemp [counter4*9+6]) || arrayCellLineageTemp [counter4*9+3] == 8 || arrayCellLineageTemp [counter4*9+3] == 32 || arrayCellLineageTemp [counter4*9+3] == 91 || arrayCellLineageTemp [counter4*9+2] == timePointMax){

**break**;

                            }

                        }

                    }

**if** (arrayCellLineageTemp [counter3*9+3] == 92){ //====TD-CF-CD====

**for** (**int** counter4 = counter3; counter4 < cellLineageTempCount/9; counter4++){

**if** (arrayCellLineageTemp [counter4*9+3] == 7){

**for** (**int** counter5 = counter3; counter5 >= 0; counter5--){

**if** (arrayCellLineageTemp [counter5*9+3] == 41 || arrayCellLineageTemp [counter5*9+3] == 51){

                                        dataTDCFCDOccurence [counter4-counter3]++;

**break**;

                                    }

**else** **if** (arrayCellLineageTemp [counter5*9+3] == 1 || arrayCellLineageTemp [counter5*9+3] == 31){

**break**;

                                    }

                                }

**break**;

                            }

**else** **if** ((arrayCellLineageTemp [counter3*9+5] != arrayCellLineageTemp [counter4*9+5] || arrayCellLineageTemp [counter3*9+6] != arrayCellLineageTemp [counter4*9+6]) || arrayCellLineageTemp [counter4*9+3] == 8 || arrayCellLineageTemp [counter4*9+3] == 32 || arrayCellLineageTemp [counter4*9+3] == 42 || arrayCellLineageTemp [counter4*9+3] == 52 || arrayCellLineageTemp [counter4*9+3] == 91 || arrayCellLineageTemp [counter4*9+2] == timePointMax){

**break**;

                            }

                        }

                    }

**if** (arrayCellLineageTemp [counter3*9+3] == 41 || arrayCellLineageTemp [counter3*9+3] == 51){ //====TD-BD====

**for** (**int** counter4 = counter3; counter4 < cellLineageTempCount/9; counter4++){

**if** (arrayCellLineageTemp [counter4*9+3] == 32){

                                dataTDBD++;

**break**;

                            }

**else** **if** ((arrayCellLineageTemp [counter3*9+5] != arrayCellLineageTemp [counter4*9+5] || arrayCellLineageTemp [counter3*9+6] != arrayCellLineageTemp [counter4*9+6]) || arrayCellLineageTemp [counter4*9+3] == 8 || arrayCellLineageTemp [counter4*9+3] == 7 || arrayCellLineageTemp [counter4*9+3] == 42 || arrayCellLineageTemp [counter4*9+3] == 52 || arrayCellLineageTemp [counter4*9+3] == 91 || arrayCellLineageTemp [counter4*9+3] == 92 || arrayCellLineageTemp [counter4*9+2] == timePointMax){

**break**;

                            }

                        }

                    }

**if** (arrayCellLineageTemp [counter3*9+3] == 41 || arrayCellLineageTemp [counter3*9+3] == 51){ //====TD-TD====

**for** (**int** counter4 = counter3; counter4 < cellLineageTempCount/9; counter4++){

**if** (arrayCellLineageTemp [counter4*9+3] == 42 || arrayCellLineageTemp [counter4*9+3] == 52){

                                dataTDTD++;

**break**;

                            }

**else** **if** ((arrayCellLineageTemp [counter3*9+5] != arrayCellLineageTemp [counter4*9+5] || arrayCellLineageTemp [counter3*9+6] != arrayCellLineageTemp [counter4*9+6]) || arrayCellLineageTemp [counter4*9+3] == 8 || arrayCellLineageTemp [counter4*9+3] == 7 || arrayCellLineageTemp [counter4*9+3] == 32 || arrayCellLineageTemp [counter4*9+3] == 91 || arrayCellLineageTemp [counter4*9+3] == 92 || arrayCellLineageTemp [counter4*9+2] == timePointMax){

**break**;

                            }

                        }

                    }

**if** (arrayCellLineageTemp [counter3*9+3] == 7){ //====TD-CD====

**for** (**int** counter4 = counter3; counter4 >= 0; counter4--){

**if** (arrayCellLineageTemp [counter4*9+3] == 41 || arrayCellLineageTemp [counter4*9+3] == 51){

                                dataTDCDOccurence [counter3-counter4]++;

**break**;

                            }

**else** **if** (arrayCellLineageTemp [counter4*9+3] == 1 || arrayCellLineageTemp [counter4*9+3] == 31 || arrayCellLineageTemp [counter4*9+3] == 92){

**break**;

                            }

                        }

                    }

**if** (arrayCellLineageTemp [counter3*9+3] == 1){ //====NonDiv====

**for** (**int** counter4 = counter3; counter4 < cellLineageTempCount/9; counter4++){

**if** (arrayCellLineageTemp [counter4*9+2] == timePointMax){

                                nonDivLing++;

**break**;

                            }

**else** **if** ((arrayCellLineageTemp [counter3*9+5] != arrayCellLineageTemp [counter4*9+5] || arrayCellLineageTemp [counter3*9+6] != arrayCellLineageTemp [counter4*9+6]) || arrayCellLineageTemp [counter4*9+3] == 8 || arrayCellLineageTemp [counter4*9+3] == 7 || arrayCellLineageTemp [counter4*9+3] == 32 || arrayCellLineageTemp [counter4*9+3] == 42 || arrayCellLineageTemp [counter4*9+3] == 52 || arrayCellLineageTemp [counter4*9+3] == 91 || arrayCellLineageTemp [counter4*9+2] == timePointMax){

**break**;

                            }

                        }

                    }

**if** (arrayCellLineageTemp [counter3*9+2] > twentyPercentOfMax && arrayCellLineageTemp [counter3*9+3] == 32){ //====Recovery====

**for** (**int** counter4 = counter3; counter4 >= 0; counter4--){

**if** (arrayCellLineageTemp [counter4*9+3] == 1){

                                recoveryGR++;

**break**;

                            }

**else** **if** (arrayCellLineageTemp [counter4*9+3] == 31 || arrayCellLineageTemp [counter4*9+3] == 41 || arrayCellLineageTemp [counter4*9+3] == 51 || arrayCellLineageTemp [counter4*9+3] == 92){

**break**;

                            }

                        }

                    }

**if** (arrayCellLineageTemp [counter3*9+3] == 1){ //====First entry to the first event====

**for** (**int** counter4 = counter3; counter4 < cellLineageTempCount/9; counter4++){

**if** (arrayCellLineageTemp [counter4*9+3] == 7 || arrayCellLineageTemp [counter4*9+3] == 32 || arrayCellLineageTemp [counter4*9+3] == 42 || arrayCellLineageTemp [counter4*9+3] == 52 || arrayCellLineageTemp [counter4*9+3] == 91 || arrayCellLineageTemp [counter4*9+2] == timePointMax){

                                dataVariationToTheFirstDV [counter4-counter3]++;

**break**;

                            }

**else** **if** ((arrayCellLineageTemp [counter3*9+5] != arrayCellLineageTemp [counter4*9+5] || arrayCellLineageTemp [counter3*9+6] != arrayCellLineageTemp [counter4*9+6]) || arrayCellLineageTemp [counter4*9+3] == 8){

**break**;

                            }

                        }

                    }

                }

                //====Caluculation of averages====

**if** (dataBD != 0) dataBDRand = dataBDRand/(**double**)dataBD;

**if** (dataCD != 0) dataCDRand = dataCDRand/(**double**)dataCD;

                //====Following arrays are used to hold the time length between BD to Any division, TD to Any division, and CF to Any division.====

**int** *dataDoublingTimeBD = **new** **int** [timePointMax+50];

**int** *dataDoublingTimeTD = **new** **int** [timePointMax+50];

**int** *dataDoublingTimeCF = **new** **int** [timePointMax+50];

**for** (**int** counter3 = 0; counter3 < timePointMax+10; counter3++){

                    dataDoublingTimeBD [counter3] = 0;

                    dataDoublingTimeTD [counter3] = 0;

                    dataDoublingTimeCF [counter3] = 0;

                }

**int** endTime = 0;

**int** startTime = 0;

**for** (**int** counter3 = 0; counter3 < cellLineageTempCount/9; counter3++){

**if** (arrayCellLineageTemp [counter3*9+3] == 31){ //====BD-Any division====

                        endTime = 0;

**for** (**int** counter4 = counter3+1; counter4 < cellLineageTempCount/9; counter4++){

**if** (arrayCellLineageTemp [counter3*9+5] == arrayCellLineageTemp [counter4*9+5] && arrayCellLineageTemp [counter3*9+6] == arrayCellLineageTemp [counter4*9+6] && (arrayCellLineageTemp [counter4*9+3] == 32 || arrayCellLineageTemp [counter4*9+3] == 42 || arrayCellLineageTemp [counter4*9+3] == 52)){

                                endTime = arrayCellLineageTemp [counter4*9+2];

                            }

**else** **if** (arrayCellLineageTemp [counter3*9+5] != arrayCellLineageTemp [counter4*9+5] || arrayCellLineageTemp [counter3*9+6] != arrayCellLineageTemp [counter4*9+6]){

**break**;

                            }

                        }

**if** (endTime != 0){

                            dataDoublingTimeBD [endTime-arrayCellLineageTemp [counter3*9+2]]++;

                        }

                    }

**if** (arrayCellLineageTemp [counter3*9+3] == 41 || arrayCellLineageTemp [counter3*9+3] == 51){ //====TD-Any division===

                        endTime = 0;

**for** (**int** counter4 = counter3+1; counter4 < cellLineageTempCount/9; counter4++){

**if** (arrayCellLineageTemp [counter3*9+5] == arrayCellLineageTemp [counter4*9+5] && arrayCellLineageTemp [counter3*9+6] == arrayCellLineageTemp [counter4*9+6] && (arrayCellLineageTemp [counter4*9+3] == 32 || arrayCellLineageTemp [counter4*9+3] == 42 || arrayCellLineageTemp [counter4*9+3] == 52)){

                                endTime = arrayCellLineageTemp [counter4*9+2];

                            }

**else** **if** (arrayCellLineageTemp [counter3*9+5] != arrayCellLineageTemp [counter4*9+5] || arrayCellLineageTemp [counter3*9+6] != arrayCellLineageTemp [counter4*9+6]){

**break**;

                            }

                        }

**if** (endTime != 0){

                            dataDoublingTimeTD [endTime-arrayCellLineageTemp [counter3*9+2]]++;

                        }

                    }

**if** (arrayCellLineageTemp [counter3*9+3] == 92){ //====CF-Any division====

                        startTime = 0;

**for** (**int** counter4 = counter3; counter4 >= 0; counter4--){

**if** (arrayCellLineageTemp [counter4*9+3] == 31 || arrayCellLineageTemp [counter4*9+3] == 41 || arrayCellLineageTemp [counter4*9+3] == 51){

                                startTime = arrayCellLineageTemp [counter4*9+2];

**break**;

                            }

**else** **if** (arrayCellLineageTemp [counter4*9+3] == 1){

**break**;

                            }

                        }

                        endTime = 0;

**for** (**int** counter4 = counter3+1; counter4 < cellLineageTempCount/9; counter4++){

**if** (arrayCellLineageTemp [counter3*9+5] == arrayCellLineageTemp [counter4*9+5] && arrayCellLineageTemp [counter3*9+6] == arrayCellLineageTemp [counter4*9+6] && (arrayCellLineageTemp [counter4*9+3] == 32 || arrayCellLineageTemp [counter4*9+3] == 42 || arrayCellLineageTemp [counter4*9+3] == 52)){

                                endTime = arrayCellLineageTemp [counter4*9+2];

                            }

**else** **if** ((arrayCellLineageTemp [counter3*9+5] != arrayCellLineageTemp [counter4*9+5] || arrayCellLineageTemp [counter3*9+6] != arrayCellLineageTemp [counter4*9+6]) || arrayCellLineageTemp [counter3*9+5] != arrayCellLineageTemp [counter4*9+5] || arrayCellLineageTemp [counter3*9+6] != arrayCellLineageTemp [counter4*9+6]){

**break**;

                            }

                        }

**if** (startTime != 0 && endTime != 0){

                            dataDoublingTimeCF [endTime-startTime]++;

                        }

                    }

                }

**int** numberOfLing = arrayTableDetail [simOperationTableCurrentRow][1]; //====Set total number of cell linease datasets in the selected database.====

**int** totalNumberOfDiv = arrayTableDetail [simOperationTableCurrentRow][13]; //====Set the total number of cell divisions.====

                //====Set data to simProcessDataBaseHold array (int, Public)====

                simProcessDataBaseHold [0] = 1;

                simProcessDataBaseHold [1] = 1;

                simProcessDataBaseHold [2] = 1;

                simProcessDataBaseHold [3] = dataBD;

                simProcessDataBaseHold [4] = dataTD;

                simProcessDataBaseHold [5] = 1;

                simProcessDataBaseHold [6] = 1;

                simProcessDataBaseHold [7] = 1;

                simProcessDataBaseHold [8] = dataBDCFBD;

                simProcessDataBaseHold [9] = dataBDCFTD;

                simProcessDataBaseHold [10] = 1;

                simProcessDataBaseHold [11] = 1;

                simProcessDataBaseHold [12] = dataTDCFBD;

                simProcessDataBaseHold [13] = dataTDCFTD;

                simProcessDataBaseHold [14] = 1;

                simProcessDataBaseHold [15] = dataTDBD;

                simProcessDataBaseHold [16] = dataTDTD;

                simProcessDataBaseHold [17] = 1;

                simProcessDataBaseHold [18] = 2;

                simProcessDataBaseHold [19] = 3;

                simProcessDataBaseHold [20] = nonDivLing;

                simProcessDataBaseHold [21] = recoveryGR;

                simProcessDataBaseHold [22] = recoveryCalcPercentHold;

                simProcessDataBaseHold [23] = totalNumberOfDiv;

                simProcessDataBaseHold [24] = numberOfLing;

                simProcessDataBaseHold [25] = timePointMax;

                simProcessDataBaseHold [26] = dataBDRand;

                simProcessDataBaseHold [27] = dataCDRand;

                //====Folowings code is used to display information to a monitor====

                [growthTimeBDDisplay setDoubleValue:simProcessDataBaseHold [0]];

                [growthTimeTDDisplay setDoubleValue:simProcessDataBaseHold [1]];

                [growthTimeCFDisplay setDoubleValue:simProcessDataBaseHold [2]];

**double** percentTemp = dataBD/(**double**)totalNumberOfDiv;

**int** percentInt = (**int**)(percentTemp*10000);

                percentTemp = percentInt/(**double**)100;

                [growthBDDisplay setDoubleValue:percentTemp];

                percentTemp = dataTD/(**double**)totalNumberOfDiv;

                percentInt = (**int**)(percentTemp*10000);

                percentTemp = percentInt/(**double**)100;

                [growthTDDisplay setDoubleValue:percentTemp];

                [growthBDCDDisplay setDoubleValue:simProcessDataBaseHold [5]];

                [growthNOCDDisplay setDoubleValue:simProcessDataBaseHold [6]];

                [growthBDCFDisplay setDoubleValue:simProcessDataBaseHold [7]];

                percentTemp = dataBDCFBD/(**double**)totalNumberOfDiv;

                percentInt = (**int**)(percentTemp*10000);

                percentTemp = percentInt/(**double**)100;

                [growthBDCFBDDisplay setDoubleValue:percentTemp];

                percentTemp = dataBDCFTD/(**double**)totalNumberOfDiv;

                percentInt = (**int**)(percentTemp*10000);

                percentTemp = percentInt/(**double**)100;

                [growthBDCFTDDisplay setDoubleValue:percentTemp];

                [growthBDCFCDDisplay setDoubleValue:simProcessDataBaseHold [10]];

                [growthTDCFDisplay setDoubleValue:simProcessDataBaseHold [11]];

                percentTemp = dataTDCFBD/(**double**)totalNumberOfDiv;

                percentInt = (**int**)(percentTemp*10000);

                percentTemp = percentInt/(**double**)100;

                [growthTDCFBDDisplay setDoubleValue:percentTemp];

                percentTemp = dataTDCFTD/(**double**)totalNumberOfDiv;

                percentInt = (**int**)(percentTemp*10000);

                percentTemp = percentInt/(**double**)100;

                [growthTDCFTDDisplay setDoubleValue:percentTemp];

                [growthTDCFCDDisplay setDoubleValue:simProcessDataBaseHold [14]];

                percentTemp = dataTDBD/(**double**)totalNumberOfDiv;

                percentInt = (**int**)(percentTemp*10000);

                percentTemp = percentInt/(**double**)100;

                [growthTDBDDisplay setDoubleValue:percentTemp];

                percentTemp = dataTDTD/(**double**)totalNumberOfDiv;

                percentInt = (**int**)(percentTemp*10000);

                percentTemp = percentInt/(**double**)100;

                [growthTDTDDisplay setDoubleValue:percentTemp];

                [growthTDCDDisplay setDoubleValue:simProcessDataBaseHold [17]];

                [growthMulTDTDDisplay setDoubleValue:simProcessDataBaseHold [18]];

                [growthSupTDBDDisplay setDoubleValue:simProcessDataBaseHold [19]];

                percentTemp = nonDivLing/(**double**)numberOfLing;

                percentInt = (**int**)(percentTemp*10000);

                percentTemp = percentInt/(**double**)100;

                [growthNoDivDisplay setDoubleValue:percentTemp];

                percentTemp = recoveryGR/(**double**)numberOfLing;

                percentInt = (**int**)(percentTemp*10000);

                percentTemp = percentInt/(**double**)100;

                [growthRecovDisplay setDoubleValue:percentTemp];

**delete** [] simulationDistributionData;

                simulationDistributionData = **new** **int** [timePointMax*11+100];

                simulationDistributionDataCount = timePointMax*11;

                //====simulationDistributionData (int) arry holds the time length of two event====

**for** (**int** counter1 = 0; counter1 < timePointMax; counter1++){

                    simulationDistributionData [counter1*11] = dataCDOccurence [counter1];

                    simulationDistributionData [counter1*11+1] = dataNonDivCDOccurence [counter1];

                    simulationDistributionData [counter1*11+2] = dataBDCFOccurence [counter1];

                    simulationDistributionData [counter1*11+3] = dataBDCFCDOccurence [counter1];

                    simulationDistributionData [counter1*11+4] = dataTDCFOccurence [counter1];

                    simulationDistributionData [counter1*11+5] = dataTDCFCDOccurence [counter1];

                    simulationDistributionData [counter1*11+6] = dataTDCDOccurence [counter1];

                    simulationDistributionData [counter1*11+7] = dataDoublingTimeBD [counter1];

                    simulationDistributionData [counter1*11+8] = dataDoublingTimeTD [counter1];

                    simulationDistributionData [counter1*11+9] = dataDoublingTimeCF [counter1];

                    simulationDistributionData [counter1*11+10] = dataVariationToTheFirstDV [counter1];

                }

**delete** [] dataCDOccurence;

**delete** [] dataNonDivCDOccurence ;

**delete** [] dataBDCFOccurence;

**delete** [] dataBDCFCDOccurence;

**delete** [] dataTDCFOccurence;

**delete** [] dataTDCFCDOccurence;

**delete** [] dataTDCDOccurence;

**delete** [] dataDoublingTimeBD;

**delete** [] dataDoublingTimeTD;

**delete** [] dataDoublingTimeCF;

**delete** [] arrayCellLineageTemp;

**delete** [] dataVariationToTheFirstDV;

                NSSound *sound = [NSSound soundNamed:@"Ping"];

                [sound play];

            }

**else**{

                NSAlert *alert = [[NSAlert alloc] init];

                [alert addButtonWithTitle:@"OK"];

                [alert setMessageText:@"List is empty"];

                [alert setAlertStyle:NSAlertStyleWarning];

                [alert runModal];

                [alert release];

                NSSound *sound = [NSSound soundNamed:@"Hero"];

                [sound play];

            }

        }

**else**{

            //====Read data from a saved file and set data to arrays====

            NSOpenPanel *openDlg = [NSOpenPanel openPanel];

            [openDlg setCanChooseFiles:**YES**];

            [openDlg setCanChooseDirectories:**YES**];

**if** ([openDlg runModal] == NSModalResponseOK){

                NSArray *files = [openDlg URLs];

                NSString *fileName = [[files objectAtIndex:0] absoluteString];

                string directryPathImport = [fileName UTF8String];

**int** findString1 = (**int**)directryPathImport.find("/Users/");

**if** (findString1 == -1) findString1 = (**int**)directryPathImport.find("/Volumes/");

**unsigned** **long** directoryLength = directryPathImport.length();

                directryPathImport = directryPathImport.substr((**unsigned** **long**)findString1, directoryLength-(**unsigned** **long**)findString1);

                string extreactedID;

                string extreactedID2;

**int** terminationFlag = 0;

**do**{

                    terminationFlag = 1;

                    findString1 = (**int**)directryPathImport.find("%20");

**if** (findString1 != -1){

                        extreactedID2 = directryPathImport.substr(0, (**unsigned** **long**)findString1);

                        directryPathImport = directryPathImport.substr((**unsigned** **long**)findString1+3);

                        directryPathImport = extreactedID2+" "+directryPathImport;

                    }

**else** terminationFlag = 0;

                } **while** (terminationFlag == 1);

                extreactedID = directryPathImport;

                string getString;

                ifstream fin;

                fin.open(extreactedID.c_str(),ios::**in**);

**if** (fin.is_open()){

                    getline(fin, getString);

**if** (getString == "Dose data array"){

                        getline(fin, getString);

                        simProcessDataBaseHold [0] = atof(getString.c_str());

                        getline(fin, getString);

                        simProcessDataBaseHold [1] = atof(getString.c_str());

                        getline(fin, getString);

                        simProcessDataBaseHold [2] = atof(getString.c_str());

                        getline(fin, getString);

                        simProcessDataBaseHold [3] = atof(getString.c_str());

                        getline(fin, getString);

                        simProcessDataBaseHold [4] = atof(getString.c_str());

                        getline(fin, getString);

                        simProcessDataBaseHold [5] = atof(getString.c_str());

                        getline(fin, getString);

                        simProcessDataBaseHold [6] = atof(getString.c_str());

                        getline(fin, getString);

                        simProcessDataBaseHold [7] = atof(getString.c_str());

                        getline(fin, getString);

                        simProcessDataBaseHold [8] = atof(getString.c_str());

                        getline(fin, getString);

                        simProcessDataBaseHold [9] = atof(getString.c_str());

                        getline(fin, getString);

                        simProcessDataBaseHold [10] = atof(getString.c_str());

                        getline(fin, getString);

                        simProcessDataBaseHold [11] = atof(getString.c_str());

                        getline(fin, getString);

                        simProcessDataBaseHold [12] = atof(getString.c_str());

                        getline(fin, getString);

                        simProcessDataBaseHold [13] = atof(getString.c_str());

                        getline(fin, getString);

                        simProcessDataBaseHold [14] = atof(getString.c_str());

                        getline(fin, getString);

                        simProcessDataBaseHold [15] = atof(getString.c_str());

                        getline(fin, getString);

                        simProcessDataBaseHold [16] = atof(getString.c_str());

                        getline(fin, getString);

                        simProcessDataBaseHold [17] = atof(getString.c_str());

                        getline(fin, getString);

                        simProcessDataBaseHold [18] = atof(getString.c_str());

                        getline(fin, getString);

                        simProcessDataBaseHold [19] = atof(getString.c_str());

                        getline(fin, getString);

                        simProcessDataBaseHold [20] = atof(getString.c_str());

                        getline(fin, getString);

                        simProcessDataBaseHold [21] = atof(getString.c_str());

                        getline(fin, getString);

                        simProcessDataBaseHold [22] = atof(getString.c_str());

                        getline(fin, getString);

                        simProcessDataBaseHold [23] = atof(getString.c_str());

                        getline(fin, getString);

                        simProcessDataBaseHold [24] = atof(getString.c_str());

                        getline(fin, getString);

                        simProcessDataBaseHold [25] = atof(getString.c_str());

                        getline(fin, getString);

                        simProcessDataBaseHold [26] = atof(getString.c_str());

                        getline(fin, getString);

                        simProcessDataBaseHold [27] = atof(getString.c_str());

                        [growthTimeBDDisplay setDoubleValue:simProcessDataBaseHold [0]];

                        [growthTimeTDDisplay setDoubleValue:simProcessDataBaseHold [1]];

                        [growthTimeCFDisplay setDoubleValue:simProcessDataBaseHold [2]];

**int** dataBD = (**int**)simProcessDataBaseHold [3];

**int** dataTD = (**int**)simProcessDataBaseHold [4];

**int** dataBDCFBD = (**int**)simProcessDataBaseHold [8];

**int** dataBDCFTD = (**int**)simProcessDataBaseHold [9];

**int** dataTDCFBD = (**int**)simProcessDataBaseHold [12];

**int** dataTDCFTD = (**int**)simProcessDataBaseHold [13];

**int** dataTDBD = (**int**)simProcessDataBaseHold [15];

**int** dataTDTD = (**int**)simProcessDataBaseHold [16];

**int** nonDivLing = (**int**)simProcessDataBaseHold [20];

**int** recoveryGR = (**int**)simProcessDataBaseHold [21];

**int** totalNumberOfDiv = (**int**)simProcessDataBaseHold [23];

**int** numberOfLing = (**int**)simProcessDataBaseHold [24];

**int** timePointMax = (**int**)simProcessDataBaseHold [25];

**double** percentTemp = dataBD/(**double**)totalNumberOfDiv;

**int** percentInt = (**int**)(percentTemp*10000);

                        percentTemp = percentInt/(**double**)100;

                        [growthBDDisplay setDoubleValue:percentTemp];

                        percentTemp = dataTD/(**double**)totalNumberOfDiv;

                        percentInt = (**int**)(percentTemp*10000);

                        percentTemp = percentInt/(**double**)100;

                        [growthTDDisplay setDoubleValue:percentTemp];

                        [growthBDCDDisplay setDoubleValue:simProcessDataBaseHold [5]];

                        [growthNOCDDisplay setDoubleValue:simProcessDataBaseHold [6]];

                        [growthBDCFDisplay setDoubleValue:simProcessDataBaseHold [7]];

                        percentTemp = dataBDCFBD/(**double**)totalNumberOfDiv;

                        percentInt = (**int**)(percentTemp*10000);

                        percentTemp = percentInt/(**double**)100;

                        [growthBDCFBDDisplay setDoubleValue:percentTemp];

                        percentTemp = dataBDCFTD/(**double**)totalNumberOfDiv;

                        percentInt = (**int**)(percentTemp*10000);

                        percentTemp = percentInt/(**double**)100;

                        [growthBDCFTDDisplay setDoubleValue:percentTemp];

                        [growthBDCFCDDisplay setDoubleValue:simProcessDataBaseHold [10]];

                        [growthTDCFDisplay setDoubleValue:simProcessDataBaseHold [11]];

                        percentTemp = dataTDCFBD/(**double**)totalNumberOfDiv;

                        percentInt = (**int**)(percentTemp*10000);

                        percentTemp = percentInt/(**double**)100;

                        [growthTDCFBDDisplay setDoubleValue:percentTemp];

                        percentTemp = dataTDCFTD/(**double**)totalNumberOfDiv;

                        percentInt = (**int**)(percentTemp*10000);

                        percentTemp = percentInt/(**double**)100;

                        [growthTDCFTDDisplay setDoubleValue:percentTemp];

                        [growthTDCFCDDisplay setDoubleValue:simProcessDataBaseHold [14]];

                        percentTemp = dataTDBD/(**double**)totalNumberOfDiv;

                        percentInt = (**int**)(percentTemp*10000);

                        percentTemp = percentInt/(**double**)100;

                        [growthTDBDDisplay setDoubleValue:percentTemp];

                        percentTemp = dataTDTD/(**double**)totalNumberOfDiv;

                        percentInt = (**int**)(percentTemp*10000);

                        percentTemp = percentInt/(**double**)100;

                        [growthTDTDDisplay setDoubleValue:percentTemp];

                        [growthTDCDDisplay setDoubleValue:simProcessDataBaseHold [17]];

                        [growthMulTDTDDisplay setDoubleValue:simProcessDataBaseHold [18]];

                        [growthSupTDBDDisplay setDoubleValue:simProcessDataBaseHold [19]];

                        percentTemp = nonDivLing/(**double**)numberOfLing;

                        percentInt = (**int**)(percentTemp*10000);

                        percentTemp = percentInt/(**double**)100;

                        [growthNoDivDisplay setDoubleValue:percentTemp];

                        percentTemp = recoveryGR/(**double**)numberOfLing;

                        percentInt = (**int**)(percentTemp*10000);

                        percentTemp = percentInt/(**double**)100;

                        [growthRecovDisplay setDoubleValue:percentTemp];

**delete** [] simulationDistributionData;

                        simulationDistributionData = **new** **int** [timePointMax*11+100];

                        simulationDistributionDataCount = timePointMax*11;

**int** entryCount = 0;

                        getline(fin, getString);

**if** (getString == "A1"){

**do**{

                                terminationFlag = 1;

                                getline(fin, getString);

**if** (getString != "End"){

                                    simulationDistributionData [entryCount*11] = atoi(getString.c_str()), entryCount++;

                                }

**else** terminationFlag = 0;

                            } **while** (terminationFlag == 1);

                        }

                        getline(fin, getString);

                        entryCount = 0;

**if** (getString == "A2"){

**do**{

                                terminationFlag = 1;

                                getline(fin, getString);

**if** (getString != "End"){

                                    simulationDistributionData [entryCount*11+1] = atoi(getString.c_str()), entryCount++;

                                }

**else** terminationFlag = 0;

                            } **while** (terminationFlag == 1);

                        }

                        getline(fin, getString);

                        entryCount = 0;

**if** (getString == "A3"){

**do**{

                                terminationFlag = 1;

                                getline(fin, getString);

**if** (getString != "End"){

                                    simulationDistributionData [entryCount*11+2] = atoi(getString.c_str()), entryCount++;

                                }

**else** terminationFlag = 0;

                            } **while** (terminationFlag == 1);

                        }

                        getline(fin, getString);

                        entryCount = 0;

**if** (getString == "A4"){

**do**{

                                terminationFlag = 1;

                                getline(fin, getString);

**if** (getString != "End"){

                                    simulationDistributionData [entryCount*11+3] = atoi(getString.c_str()), entryCount++;

                                }

**else** terminationFlag = 0;

                            } **while** (terminationFlag == 1);

                        }

                        getline(fin, getString);

                        entryCount = 0;

**if** (getString == "A5"){

**do**{

                                terminationFlag = 1;

                                getline(fin, getString);

**if** (getString != "End"){

                                    simulationDistributionData [entryCount*11+4] = atoi(getString.c_str()), entryCount++;

                                }

**else** terminationFlag = 0;

                            } **while** (terminationFlag == 1);

                        }

                        getline(fin, getString);

                        entryCount = 0;

**if** (getString == "A6"){

**do**{

                                terminationFlag = 1;

                                getline(fin, getString);

**if** (getString != "End"){

                                    simulationDistributionData [entryCount*11+5] = atoi(getString.c_str()), entryCount++;

                                }

**else** terminationFlag = 0;

                            } **while** (terminationFlag == 1);

                        }

                        getline(fin, getString);

                        entryCount = 0;

**if** (getString == "A7"){

**do**{

                                terminationFlag = 1;

                                getline(fin, getString);

**if** (getString != "End"){

                                    simulationDistributionData [entryCount*11+6] = atoi(getString.c_str()), entryCount++;

                                }

**else** terminationFlag = 0;

                            } **while** (terminationFlag == 1);

                        }

                        getline(fin, getString);

                        entryCount = 0;

**if** (getString == "A8"){

**do**{

                                terminationFlag = 1;

                                getline(fin, getString);

**if** (getString != "End"){

                                    simulationDistributionData [entryCount*11+7] = atoi(getString.c_str()), entryCount++;

                                }

**else** terminationFlag = 0;

                            } **while** (terminationFlag == 1);

                        }

                        getline(fin, getString);

                        entryCount = 0;

**if** (getString == "A9"){

**do**{

                                terminationFlag = 1;

                                getline(fin, getString);

**if** (getString != "End"){

                                    simulationDistributionData [entryCount*11+8] = atoi(getString.c_str()), entryCount++;

                                }

**else** terminationFlag = 0;

                            } **while** (terminationFlag == 1);

                        }

                        getline(fin, getString);

                        entryCount = 0;

**if** (getString == "A10"){

**do**{

                                terminationFlag = 1;

                                getline(fin, getString);

**if** (getString != "End"){

                                    simulationDistributionData [entryCount*11+9] = atoi(getString.c_str()), entryCount++;

                                }

**else** terminationFlag = 0;

                            } **while** (terminationFlag == 1);

                        }

                        getline(fin, getString);

                        entryCount = 0;

**if** (getString == "A11"){

**do**{

                                terminationFlag = 1;

                                getline(fin, getString);

**if** (getString != "End"){

                                    simulationDistributionData [entryCount*11+10] = atoi(getString.c_str()), entryCount++;

                                }

**else** terminationFlag = 0;

                            } **while** (terminationFlag == 1);

                        }

                        NSSound *sound = [NSSound soundNamed:@"Ping"];

                        [sound play];

                    }

**else**{

                        NSAlert *alert = [[NSAlert alloc] init];

                        [alert addButtonWithTitle:@"OK"];

                        [alert setMessageText:@"No Sim database file"];

                        [alert setAlertStyle:NSAlertStyleWarning];

                        [alert runModal];

                        [alert release];

                        NSSound *sound = [NSSound soundNamed:@"Hero"];

                        [sound play];

                    }

                    fin.close();

                }

            }

        }

    }

**else**{

        NSAlert *alert = [[NSAlert alloc] init];

        [alert addButtonWithTitle:@"OK"];

        [alert setMessageText:@"Sim is in progress"];

        [alert setAlertStyle:NSAlertStyleWarning];

        [alert runModal];

        [alert release];

        NSSound *sound = [NSSound soundNamed:@"Hero"];

        [sound play];

    }

}

-(**IBAction**)getListGrowthProgSet:(**id**)sender{

**if** (simulationprogress == 0){

**if** (databaseLoadOptionHold == 0){

**if** (simListHoldCount != 0){

                messageStringSim2 = arraySimListHold [simOperationTableCurrentRow*2+1].substr(arraySimListHold [simOperationTableCurrentRow*2+1].find("-")+1);

                NSString *treatPickUpNameNSString = @(messageStringSim2.c_str());

                [growthPrgTreatDisplay setStringValue: treatPickUpNameNSString];

**int** *arrayCellLineageTemp = **new** **int** [(arrayLineageDataEntryHold [simOperationTableCurrentRow]/9)*9+100];

**int** cellLineageTempCount = 0;

**for** (**unsigned** **long** counter3 = 0; counter3 < (arrayLineageDataEntryHold [simOperationTableCurrentRow]/9)*9+100; counter3++){

                    arrayCellLineageTemp [counter3] = 0;

                }

**for** (**unsigned** **long** counter3 = 0; counter3 < arrayLineageDataEntryHold [simOperationTableCurrentRow]/9; counter3++){

                    arrayCellLineageTemp [cellLineageTempCount] = arrayLineageData [simOperationTableCurrentRow][counter3*9], cellLineageTempCount++;

                    arrayCellLineageTemp [cellLineageTempCount] = arrayLineageData [simOperationTableCurrentRow][counter3*9+1], cellLineageTempCount++;

                    arrayCellLineageTemp [cellLineageTempCount] = arrayLineageData [simOperationTableCurrentRow][counter3*9+2], cellLineageTempCount++;

                    arrayCellLineageTemp [cellLineageTempCount] = arrayLineageData [simOperationTableCurrentRow][counter3*9+3], cellLineageTempCount++;

                    arrayCellLineageTemp [cellLineageTempCount] = arrayLineageData [simOperationTableCurrentRow][counter3*9+4], cellLineageTempCount++;

                    arrayCellLineageTemp [cellLineageTempCount] = arrayLineageData [simOperationTableCurrentRow][counter3*9+5], cellLineageTempCount++;

                    arrayCellLineageTemp [cellLineageTempCount] = arrayLineageData [simOperationTableCurrentRow][counter3*9+6], cellLineageTempCount++;

                    arrayCellLineageTemp [cellLineageTempCount] = arrayLineageData [simOperationTableCurrentRow][counter3*9+7], cellLineageTempCount++;

                    arrayCellLineageTemp [cellLineageTempCount] = arrayLineageData [simOperationTableCurrentRow][counter3*9+8], cellLineageTempCount++;

                }

**for** (**int** counter3 = 0; counter3 < cellLineageTempCount/9; counter3++){

**if** (arrayCellLineageTemp [counter3*9+3] == 92){

**for** (**int** counter4 = counter3-1; counter4 >= 0; counter4--){

**if** (arrayCellLineageTemp [counter4*9+3] == 92){

                                arrayCellLineageTemp [counter4*9+3] = 2;

                            }

**else** **if** (arrayCellLineageTemp [counter4*9+3] == 1 || arrayCellLineageTemp [counter4*9+3] == 31 || arrayCellLineageTemp [counter4*9+3] == 41 || arrayCellLineageTemp [counter4*9+3] == 51){

**break**;

                            }

                        }

                    }

                }

**int** timePointMax = arrayTableDetail [simOperationTableCurrentRow][3];

**int** twentyPercentOfMax = timePointMax-(**int**)(round(timePointMax*(**double**)(recoveryCalcPercentHold/(**double**)100)));

**int** *dataCDOccurence = **new** **int** [timePointMax+10];

**int** *dataNonDivCDOccurence = **new** **int** [timePointMax+10];

**int** *dataBDCFOccurence = **new** **int** [timePointMax+10];

**int** *dataBDCFCDOccurence = **new** **int** [timePointMax+10];

**int** *dataTDCFOccurence = **new** **int** [timePointMax+10];

**int** *dataTDCFCDOccurence = **new** **int** [timePointMax+10];

**int** *dataTDCDOccurence = **new** **int** [timePointMax+10];

**int** *dataVariationToTheFirstDV = **new** **int** [timePointMax+10];

**for** (**int** counter3 = 0; counter3 < timePointMax+10; counter3++){

                    dataCDOccurence [counter3] = 0;

                    dataNonDivCDOccurence [counter3] = 0;

                    dataBDCFOccurence [counter3] = 0;

                    dataBDCFCDOccurence [counter3] = 0;

                    dataTDCFOccurence [counter3] = 0;

                    dataTDCFCDOccurence [counter3] = 0;

                    dataTDCDOccurence [counter3] = 0;

                    dataVariationToTheFirstDV [counter3] = 0;

                }

**int** dataBD = 0;

**int** dataTD = 0;

**int** dataBDCFBD = 0;

**int** dataBDCFTD = 0;

**int** dataTDCFBD = 0;

**int** dataTDCFTD = 0;

**int** dataTDBD = 0;

**int** dataTDTD = 0;

**int** nonDivLing = 0;

**int** recoveryGR = 0;

**int** dataCD = 0;

**double** dataBDRand = 0;

**double** dataCDRand = 0;

**for** (**int** counter3 = 0; counter3 < cellLineageTempCount/9; counter3++){

**if** (arrayCellLineageTemp [counter3*9+3] == 32){

                        dataBD++;

                        dataBDRand = dataBDRand+arrayCellLineageTemp [counter3*9+2];

                    }

**if** (arrayCellLineageTemp [counter3*9+3] == 42 || arrayCellLineageTemp [counter3*9+3] == 52) dataTD++;

**if** (arrayCellLineageTemp [counter3*9+3] == 7){

**for** (**int** counter4 = counter3; counter4 >= 0; counter4--){

**if** (arrayCellLineageTemp [counter4*9+3] == 31){

                                dataCDOccurence [counter3-counter4]++;

**break**;

                            }

**else** **if** (arrayCellLineageTemp [counter4*9+3] == 1 || arrayCellLineageTemp [counter4*9+3] == 41 || arrayCellLineageTemp [counter4*9+3] == 51 || arrayCellLineageTemp [counter4*9+3] == 92){

**break**;

                            }

                        }

                        dataCD++;

                        dataCDRand = dataCDRand+arrayCellLineageTemp [counter3*9+2];

                    }

**if** (arrayCellLineageTemp [counter3*9+3] == 7){

**for** (**int** counter4 = counter3; counter4 >= 0; counter4--){

**if** (arrayCellLineageTemp [counter4*9+3] == 1){

                                dataNonDivCDOccurence [counter3-counter4]++;

**break**;

                            }

**else** **if** (arrayCellLineageTemp [counter4*9+3] == 31 || arrayCellLineageTemp [counter4*9+3] == 41 || arrayCellLineageTemp [counter4*9+3] == 51 || arrayCellLineageTemp [counter4*9+3] == 92){

**break**;

                            }

                        }

                    }

**if** (arrayCellLineageTemp [counter3*9+3] == 91){

**for** (**int** counter4 = counter3; counter4 >= 0; counter4--){

**if** (arrayCellLineageTemp [counter4*9+3] == 31){

                                dataBDCFOccurence [counter3-counter4]++;

**break**;

                            }

**else** **if** (arrayCellLineageTemp [counter4*9+3] == 1 || arrayCellLineageTemp [counter4*9+3] == 41 || arrayCellLineageTemp [counter4*9+3] == 51 || arrayCellLineageTemp [counter4*9+3] == 92){

**break**;

                            }

                        }

                    }

**if** (arrayCellLineageTemp [counter3*9+3] == 92){

**for** (**int** counter4 = counter3; counter4 < cellLineageTempCount/9; counter4++){

**if** (arrayCellLineageTemp [counter4*9+3] == 32){

**for** (**int** counter5 = counter3; counter5 >= 0; counter5--){

**if** (arrayCellLineageTemp [counter5*9+3] == 31){

                                        dataBDCFBD++;

**break**;

                                    }

**else** **if** (arrayCellLineageTemp [counter5*9+3] == 1 || arrayCellLineageTemp [counter5*9+3] == 41 || arrayCellLineageTemp [counter5*9+3] == 51){

**break**;

                                    }

                                }

**break**;

                            }

**else** **if** ((arrayCellLineageTemp [counter3*9+5] != arrayCellLineageTemp [counter4*9+5] || arrayCellLineageTemp [counter3*9+6] != arrayCellLineageTemp [counter4*9+6]) || arrayCellLineageTemp [counter4*9+3] == 8 || arrayCellLineageTemp [counter4*9+3] == 42 || arrayCellLineageTemp [counter4*9+3] == 52 || arrayCellLineageTemp [counter4*9+3] == 91 || arrayCellLineageTemp [counter4*9+2] == timePointMax){

**break**;

                            }

                        }

                    }

**if** (arrayCellLineageTemp [counter3*9+3] == 92){

**for** (**int** counter4 = counter3; counter4 < cellLineageTempCount/9; counter4++){

**if** (arrayCellLineageTemp [counter4*9+3] == 42 || arrayCellLineageTemp [counter4*9+3] == 52){

**for** (**int** counter5 = counter3; counter5 >= 0; counter5--){

**if** (arrayCellLineageTemp [counter5*9+3] == 31){

                                        dataBDCFTD++;

**break**;

                                    }

**else** **if** (arrayCellLineageTemp [counter5*9+3] == 1 || arrayCellLineageTemp [counter5*9+3] == 41 || arrayCellLineageTemp [counter5*9+3] == 51){

**break**;

                                    }

                                }

**break**;

                            }

**else** **if** ((arrayCellLineageTemp [counter3*9+5] != arrayCellLineageTemp [counter4*9+5] || arrayCellLineageTemp [counter3*9+6] != arrayCellLineageTemp [counter4*9+6]) || arrayCellLineageTemp [counter4*9+3] == 8 || arrayCellLineageTemp [counter4*9+3] == 32 || arrayCellLineageTemp [counter4*9+3] == 91 || arrayCellLineageTemp [counter4*9+2] == timePointMax){

**break**;

                            }

                        }

                    }

**if** (arrayCellLineageTemp [counter3*9+3] == 92){

**for** (**int** counter4 = counter3; counter4 < cellLineageTempCount/9; counter4++){

**if** (arrayCellLineageTemp [counter4*9+3] == 7){

**for** (**int** counter5 = counter3; counter5 >= 0; counter5--){

**if** (arrayCellLineageTemp [counter5*9+3] == 31){

                                        dataBDCFCDOccurence [counter4-counter3]++;

**break**;

                                    }

**else** **if** (arrayCellLineageTemp [counter5*9+3] == 1 || arrayCellLineageTemp [counter5*9+3] == 41 || arrayCellLineageTemp [counter5*9+3] == 51){

**break**;

                                    }

                                }

**break**;

                            }

**else** **if** ((arrayCellLineageTemp [counter3*9+5] != arrayCellLineageTemp [counter4*9+5] || arrayCellLineageTemp [counter3*9+6] != arrayCellLineageTemp [counter4*9+6]) || arrayCellLineageTemp [counter4*9+3] == 8 || arrayCellLineageTemp [counter4*9+3] == 32 || arrayCellLineageTemp [counter4*9+3] == 42 || arrayCellLineageTemp [counter4*9+3] == 52 || arrayCellLineageTemp [counter4*9+3] == 91 || arrayCellLineageTemp [counter4*9+2] == timePointMax){

**break**;

                            }

                        }

                    }

**if** (arrayCellLineageTemp [counter3*9+3] == 91){

**for** (**int** counter4 = counter3; counter4 >= 0; counter4--){

**if** (arrayCellLineageTemp [counter4*9+3] == 41 || arrayCellLineageTemp [counter4*9+3] == 51){

                                dataTDCFOccurence [counter3-counter4]++;

**break**;

                            }

**else** **if** (arrayCellLineageTemp [counter4*9+3] == 1 || arrayCellLineageTemp [counter4*9+3] == 31 || arrayCellLineageTemp [counter4*9+3] == 92){

**break**;

                            }

                        }

                    }

**if** (arrayCellLineageTemp [counter3*9+3] == 92){

**for** (**int** counter4 = counter3; counter4 < cellLineageTempCount/9; counter4++){

**if** (arrayCellLineageTemp [counter4*9+3] == 32){

**for** (**int** counter5 = counter3; counter5 >= 0; counter5--){

**if** (arrayCellLineageTemp [counter5*9+3] == 41 || arrayCellLineageTemp [counter5*9+3] == 51){

                                        dataTDCFBD++;

**break**;

                                    }

**else** **if** (arrayCellLineageTemp [counter5*9+3] == 1 || arrayCellLineageTemp [counter5*9+3] == 31){

**break**;

                                    }

                                }

**break**;

                            }

**else** **if** ((arrayCellLineageTemp [counter3*9+5] != arrayCellLineageTemp [counter4*9+5] || arrayCellLineageTemp [counter3*9+6] != arrayCellLineageTemp [counter4*9+6]) || arrayCellLineageTemp [counter4*9+3] == 8 || arrayCellLineageTemp [counter4*9+3] == 42 || arrayCellLineageTemp [counter4*9+3] == 52 || arrayCellLineageTemp [counter4*9+3] == 91 || arrayCellLineageTemp [counter4*9+2] == timePointMax){

**break**;

                            }

                        }

                    }

**if** (arrayCellLineageTemp [counter3*9+3] == 92){

**for** (**int** counter4 = counter3; counter4 < cellLineageTempCount/9; counter4++){

**if** (arrayCellLineageTemp [counter4*9+3] == 42 || arrayCellLineageTemp [counter4*9+3] == 52){

**for** (**int** counter5 = counter3; counter5 >= 0; counter5--){

**if** (arrayCellLineageTemp [counter5*9+3] == 41 || arrayCellLineageTemp [counter5*9+3] == 51){

                                        dataTDCFTD++;

**break**;

                                    }

**else** **if** (arrayCellLineageTemp [counter5*9+3] == 1 || arrayCellLineageTemp [counter5*9+3] == 31){

**break**;

                                    }

                                }

**break**;

                            }

**else** **if** ((arrayCellLineageTemp [counter3*9+5] != arrayCellLineageTemp [counter4*9+5] || arrayCellLineageTemp [counter3*9+6] != arrayCellLineageTemp [counter4*9+6]) || arrayCellLineageTemp [counter4*9+3] == 8 || arrayCellLineageTemp [counter4*9+3] == 32 || arrayCellLineageTemp [counter4*9+3] == 91 || arrayCellLineageTemp [counter4*9+2] == timePointMax){

**break**;

                            }

                        }

                    }

**if** (arrayCellLineageTemp [counter3*9+3] == 92){

**for** (**int** counter4 = counter3; counter4 < cellLineageTempCount/9; counter4++){

**if** (arrayCellLineageTemp [counter4*9+3] == 7){

**for** (**int** counter5 = counter3; counter5 >= 0; counter5--){

**if** (arrayCellLineageTemp [counter5*9+3] == 41 || arrayCellLineageTemp [counter5*9+3] == 51){

                                        dataTDCFCDOccurence [counter4-counter3]++;

**break**;

                                    }

**else** **if** (arrayCellLineageTemp [counter5*9+3] == 1 || arrayCellLineageTemp [counter5*9+3] == 31){

**break**;

                                    }

                                }

**break**;

                            }

**else** **if** ((arrayCellLineageTemp [counter3*9+5] != arrayCellLineageTemp [counter4*9+5] || arrayCellLineageTemp [counter3*9+6] != arrayCellLineageTemp [counter4*9+6]) || arrayCellLineageTemp [counter4*9+3] == 8 || arrayCellLineageTemp [counter4*9+3] == 32 || arrayCellLineageTemp [counter4*9+3] == 42 || arrayCellLineageTemp [counter4*9+3] == 52 || arrayCellLineageTemp [counter4*9+3] == 91 || arrayCellLineageTemp [counter4*9+2] == timePointMax){

**break**;

                            }

                        }

                    }

**if** (arrayCellLineageTemp [counter3*9+3] == 41 || arrayCellLineageTemp [counter3*9+3] == 51){

**for** (**int** counter4 = counter3; counter4 < cellLineageTempCount/9; counter4++){

**if** (arrayCellLineageTemp [counter4*9+3] == 32){

                                dataTDBD++;

**break**;

                            }

**else** **if** ((arrayCellLineageTemp [counter3*9+5] != arrayCellLineageTemp [counter4*9+5] || arrayCellLineageTemp [counter3*9+6] != arrayCellLineageTemp [counter4*9+6]) || arrayCellLineageTemp [counter4*9+3] == 8 || arrayCellLineageTemp [counter4*9+3] == 7 || arrayCellLineageTemp [counter4*9+3] == 42 || arrayCellLineageTemp [counter4*9+3] == 52 || arrayCellLineageTemp [counter4*9+3] == 91 || arrayCellLineageTemp [counter4*9+3] == 92 || arrayCellLineageTemp [counter4*9+2] == timePointMax){

**break**;

                            }

                        }

                    }

**if** (arrayCellLineageTemp [counter3*9+3] == 41 || arrayCellLineageTemp [counter3*9+3] == 51){

**for** (**int** counter4 = counter3; counter4 < cellLineageTempCount/9; counter4++){

**if** (arrayCellLineageTemp [counter4*9+3] == 42 || arrayCellLineageTemp [counter4*9+3] == 52){

                                dataTDTD++;

**break**;

                            }

**else** **if** ((arrayCellLineageTemp [counter3*9+5] != arrayCellLineageTemp [counter4*9+5] || arrayCellLineageTemp [counter3*9+6] != arrayCellLineageTemp [counter4*9+6]) || arrayCellLineageTemp [counter4*9+3] == 8 || arrayCellLineageTemp [counter4*9+3] == 7 || arrayCellLineageTemp [counter4*9+3] == 32 || arrayCellLineageTemp [counter4*9+3] == 91 || arrayCellLineageTemp [counter4*9+3] == 92 || arrayCellLineageTemp [counter4*9+2] == timePointMax){

**break**;

                            }

                        }

                    }

**if** (arrayCellLineageTemp [counter3*9+3] == 7){

**for** (**int** counter4 = counter3; counter4 >= 0; counter4--){

**if** (arrayCellLineageTemp [counter4*9+3] == 41 || arrayCellLineageTemp [counter4*9+3] == 51){

                                dataTDCDOccurence [counter3-counter4]++;

**break**;

                            }

**else** **if** (arrayCellLineageTemp [counter4*9+3] == 1 || arrayCellLineageTemp [counter4*9+3] == 31 || arrayCellLineageTemp [counter4*9+3] == 92){

**break**;

                            }

                        }

                    }

**if** (arrayCellLineageTemp [counter3*9+3] == 1){

**for** (**int** counter4 = counter3; counter4 < cellLineageTempCount/9; counter4++){

**if** (arrayCellLineageTemp [counter4*9+2] == timePointMax){

                                nonDivLing++;

**break**;

                            }

**else** **if** ((arrayCellLineageTemp [counter3*9+5] != arrayCellLineageTemp [counter4*9+5] || arrayCellLineageTemp [counter3*9+6] != arrayCellLineageTemp [counter4*9+6]) || arrayCellLineageTemp [counter4*9+3] == 8 || arrayCellLineageTemp [counter4*9+3] == 7 || arrayCellLineageTemp [counter4*9+3] == 32 || arrayCellLineageTemp [counter4*9+3] == 42 || arrayCellLineageTemp [counter4*9+3] == 52 || arrayCellLineageTemp [counter4*9+3] == 91 || arrayCellLineageTemp [counter4*9+2] == timePointMax){

**break**;

                            }

                        }

                    }

**if** (arrayCellLineageTemp [counter3*9+2] > twentyPercentOfMax && arrayCellLineageTemp [counter3*9+3] == 32){

**for** (**int** counter4 = counter3; counter4 >= 0; counter4--){

**if** (arrayCellLineageTemp [counter4*9+3] == 1){

                                recoveryGR++;

**break**;

                            }

**else** **if** (arrayCellLineageTemp [counter4*9+3] == 31 || arrayCellLineageTemp [counter4*9+3] == 41 || arrayCellLineageTemp [counter4*9+3] == 51 || arrayCellLineageTemp [counter4*9+3] == 92){

**break**;

                            }

                        }

                    }

**if** (arrayCellLineageTemp [counter3*9+3] == 1){

**for** (**int** counter4 = counter3; counter4 < cellLineageTempCount/9; counter4++){

**if** (arrayCellLineageTemp [counter4*9+3] == 7 || arrayCellLineageTemp [counter4*9+3] == 32 || arrayCellLineageTemp [counter4*9+3] == 42 || arrayCellLineageTemp [counter4*9+3] == 52 || arrayCellLineageTemp [counter4*9+3] == 91 || arrayCellLineageTemp [counter4*9+2] == timePointMax){

                                dataVariationToTheFirstDV [counter4-counter3]++;

**break**;

                            }

**else** **if** ((arrayCellLineageTemp [counter3*9+5] != arrayCellLineageTemp [counter4*9+5] || arrayCellLineageTemp [counter3*9+6] != arrayCellLineageTemp [counter4*9+6]) || arrayCellLineageTemp [counter4*9+3] == 8){

**break**;

                            }

                        }

                    }

                }

**if** (dataBD != 0) dataBDRand = dataBDRand/(**double**)dataBD;

**if** (dataCD != 0) dataCDRand = dataCDRand/(**double**)dataCD;

**int** *dataDoublingTimeBD = **new** **int** [timePointMax+50];

**int** *dataDoublingTimeTD = **new** **int** [timePointMax+50];

**int** *dataDoublingTimeCF = **new** **int** [timePointMax+50];

**for** (**int** counter3 = 0; counter3 < timePointMax+10; counter3++){

                    dataDoublingTimeBD [counter3] = 0;

                    dataDoublingTimeTD [counter3] = 0;

                    dataDoublingTimeCF [counter3] = 0;

                }

**int** endTime = 0;

**int** startTime = 0;

**for** (**int** counter3 = 0; counter3 < cellLineageTempCount/9; counter3++){

**if** (arrayCellLineageTemp [counter3*9+3] == 31){

                        endTime = 0;

**for** (**int** counter4 = counter3+1; counter4 < cellLineageTempCount/9; counter4++){

**if** (arrayCellLineageTemp [counter3*9+5] == arrayCellLineageTemp [counter4*9+5] && arrayCellLineageTemp [counter3*9+6] == arrayCellLineageTemp [counter4*9+6] && (arrayCellLineageTemp [counter4*9+3] == 32 || arrayCellLineageTemp [counter4*9+3] == 42 || arrayCellLineageTemp [counter4*9+3] == 52)){

                                endTime = arrayCellLineageTemp [counter4*9+2];

                            }

**else** **if** (arrayCellLineageTemp [counter3*9+5] != arrayCellLineageTemp [counter4*9+5] || arrayCellLineageTemp [counter3*9+6] != arrayCellLineageTemp [counter4*9+6]){

**break**;

                            }

                        }

**if** (endTime != 0){

                            dataDoublingTimeBD [endTime-arrayCellLineageTemp [counter3*9+2]]++;

                        }

                    }

**if** (arrayCellLineageTemp [counter3*9+3] == 41 || arrayCellLineageTemp [counter3*9+3] == 51){

                        endTime = 0;

**for** (**int** counter4 = counter3+1; counter4 < cellLineageTempCount/9; counter4++){

**if** (arrayCellLineageTemp [counter3*9+5] == arrayCellLineageTemp [counter4*9+5] && arrayCellLineageTemp [counter3*9+6] == arrayCellLineageTemp [counter4*9+6] && (arrayCellLineageTemp [counter4*9+3] == 32 || arrayCellLineageTemp [counter4*9+3] == 42 || arrayCellLineageTemp [counter4*9+3] == 52)){

                                endTime = arrayCellLineageTemp [counter4*9+2];

                            }

**else** **if** (arrayCellLineageTemp [counter3*9+5] != arrayCellLineageTemp [counter4*9+5] || arrayCellLineageTemp [counter3*9+6] != arrayCellLineageTemp [counter4*9+6]){

**break**;

                            }

                        }

**if** (endTime != 0){

                            dataDoublingTimeTD [endTime-arrayCellLineageTemp [counter3*9+2]]++;

                        }

                    }

**if** (arrayCellLineageTemp [counter3*9+3] == 92){

                        startTime = 0;

**for** (**int** counter4 = counter3; counter4 >= 0; counter4--){

**if** (arrayCellLineageTemp [counter4*9+3] == 31 || arrayCellLineageTemp [counter4*9+3] == 41 || arrayCellLineageTemp [counter4*9+3] == 51){

                                startTime = arrayCellLineageTemp [counter4*9+2];

**break**;

                            }

**else** **if** (arrayCellLineageTemp [counter4*9+3] == 1){

**break**;

                            }

                        }

                        endTime = 0;

**for** (**int** counter4 = counter3+1; counter4 < cellLineageTempCount/9; counter4++){

**if** (arrayCellLineageTemp [counter3*9+5] == arrayCellLineageTemp [counter4*9+5] && arrayCellLineageTemp [counter3*9+6] == arrayCellLineageTemp [counter4*9+6] && (arrayCellLineageTemp [counter4*9+3] == 32 || arrayCellLineageTemp [counter4*9+3] == 42 || arrayCellLineageTemp [counter4*9+3] == 52)){

                                endTime = arrayCellLineageTemp [counter4*9+2];

                            }

**else** **if** (arrayCellLineageTemp [counter3*9+5] != arrayCellLineageTemp [counter4*9+5] || arrayCellLineageTemp [counter3*9+6] != arrayCellLineageTemp [counter4*9+6]){

**break**;

                            }

                        }

**if** (startTime != 0 && endTime != 0){

                            dataDoublingTimeCF [endTime-startTime]++;

                        }

                    }

                }

**int** numberOfLing = arrayTableDetail [simOperationTableCurrentRow][1];

**int** totalNumberOfDiv = arrayTableDetail [simOperationTableCurrentRow][13];

                simProcessDataProgHold [0] = 1;

                simProcessDataProgHold [1] = 1;

                simProcessDataProgHold [2] = 1;

                simProcessDataProgHold [3] = dataBD;

                simProcessDataProgHold [4] = dataTD;

                simProcessDataProgHold [5] = 1;

                simProcessDataProgHold [6] = 1;

                simProcessDataProgHold [7] = 1;

                simProcessDataProgHold [8] = dataBDCFBD;

                simProcessDataProgHold [9] = dataBDCFTD;

                simProcessDataProgHold [10] = 1;

                simProcessDataProgHold [11] = 1;

                simProcessDataProgHold [12] = dataTDCFBD;

                simProcessDataProgHold [13] = dataTDCFTD;

                simProcessDataProgHold [14] = 1;

                simProcessDataProgHold [15] = dataTDBD;

                simProcessDataProgHold [16] = dataTDTD;

                simProcessDataProgHold [17] = 1;

                simProcessDataProgHold [18] = 2;

                simProcessDataProgHold [19] = 3;

                simProcessDataProgHold [20] = nonDivLing;

                simProcessDataProgHold [21] = recoveryGR;

                simProcessDataProgHold [22] = recoveryCalcPercentHold;

                simProcessDataProgHold [23] = totalNumberOfDiv;

                simProcessDataProgHold [24] = numberOfLing;

                simProcessDataProgHold [25] = timePointMax;

                simProcessDataProgHold [26] = dataBDRand;

                simProcessDataProgHold [27] = dataCDRand;

                [growthPrgTimeBDDisplay setDoubleValue:simProcessDataProgHold [0]];

                [growthPrgTimeTDDisplay setDoubleValue:simProcessDataProgHold [1]];

                [growthPrgTimeCFDisplay setDoubleValue:simProcessDataProgHold [2]];

**double** percentTemp = dataBD/(**double**)totalNumberOfDiv;

**int** percentInt = (**int**)(percentTemp*10000);

                percentTemp = percentInt/(**double**)100;

                [growthPrgBDDisplay setDoubleValue:percentTemp];

                percentTemp = dataTD/(**double**)totalNumberOfDiv;

                percentInt = (**int**)(percentTemp*10000);

                percentTemp = percentInt/(**double**)100;

                [growthPrgTDDisplay setDoubleValue:percentTemp];

                [growthPrgBDCDDisplay setDoubleValue:simProcessDataProgHold [5]];

                [growthPrgNOCDDisplay setDoubleValue:simProcessDataProgHold [6]];

                [growthPrgBDCFDisplay setDoubleValue:simProcessDataProgHold [7]];

                percentTemp = dataBDCFBD/(**double**)totalNumberOfDiv;

                percentInt = (**int**)(percentTemp*10000);

                percentTemp = percentInt/(**double**)100;

                [growthPrgBDCFBDDisplay setDoubleValue:percentTemp];

                percentTemp = dataBDCFTD/(**double**)totalNumberOfDiv;

                percentInt = (**int**)(percentTemp*10000);

                percentTemp = percentInt/(**double**)100;

                [growthPrgBDCFTDDisplay setDoubleValue:percentTemp];

                [growthPrgBDCFCDDisplay setDoubleValue:simProcessDataProgHold [10]];

                [growthPrgTDCFDisplay setDoubleValue:simProcessDataProgHold [11]];

                percentTemp = dataTDCFBD/(**double**)totalNumberOfDiv;

                percentInt = (**int**)(percentTemp*10000);

                percentTemp = percentInt/(**double**)100;

                [growthPrgTDCFBDDisplay setDoubleValue:percentTemp];

                percentTemp = dataTDCFTD/(**double**)totalNumberOfDiv;

                percentInt = (**int**)(percentTemp*10000);

                percentTemp = percentInt/(**double**)100;

                [growthPrgTDCFTDDisplay setDoubleValue:percentTemp];

                [growthPrgTDCFCDDisplay setDoubleValue:simProcessDataProgHold [14]];

                percentTemp = dataTDBD/(**double**)totalNumberOfDiv;

                percentInt = (**int**)(percentTemp*10000);

                percentTemp = percentInt/(**double**)100;

                [growthPrgTDBDDisplay setDoubleValue:percentTemp];

                percentTemp = dataTDTD/(**double**)totalNumberOfDiv;

                percentInt = (**int**)(percentTemp*10000);

                percentTemp = percentInt/(**double**)100;

                [growthPrgTDTDDisplay setDoubleValue:percentTemp];

                [growthPrgTDCDDisplay setDoubleValue:simProcessDataProgHold [17]];

                [growthPrgMulTDTDDisplay setDoubleValue:simProcessDataProgHold [18]];

                [growthPrgSupTDBDDisplay setDoubleValue:simProcessDataProgHold [19]];

                percentTemp = nonDivLing/(**double**)numberOfLing;

                percentInt = (**int**)(percentTemp*10000);

                percentTemp = percentInt/(**double**)100;

                [growthPrgNoDivDisplay setDoubleValue:percentTemp];

                percentTemp = recoveryGR/(**double**)numberOfLing;

                percentInt = (**int**)(percentTemp*10000);

                percentTemp = percentInt/(**double**)100;

                [growthPrgRecovDisplay setDoubleValue:percentTemp];

**delete** [] simulationDistributionProgData;

                simulationDistributionProgData = **new** **int** [timePointMax*11+100];

                simulationDistributionProgDataCount = timePointMax*11;

**for** (**int** counter1 = 0; counter1 < timePointMax; counter1++){

                    simulationDistributionProgData [counter1*11] = dataCDOccurence [counter1];

                    simulationDistributionProgData [counter1*11+1] = dataNonDivCDOccurence [counter1];

                    simulationDistributionProgData [counter1*11+2] = dataBDCFOccurence [counter1];

                    simulationDistributionProgData [counter1*11+3] = dataBDCFCDOccurence [counter1];

                    simulationDistributionProgData [counter1*11+4] = dataTDCFOccurence [counter1];

                    simulationDistributionProgData [counter1*11+5] = dataTDCFCDOccurence [counter1];

                    simulationDistributionProgData [counter1*11+6] = dataTDCDOccurence [counter1];

                    simulationDistributionProgData [counter1*11+7] = dataDoublingTimeBD [counter1];

                    simulationDistributionProgData [counter1*11+8] = dataDoublingTimeTD [counter1];

                    simulationDistributionProgData [counter1*11+9] = dataDoublingTimeCF [counter1];

                    simulationDistributionProgData [counter1*11+10] = dataVariationToTheFirstDV [counter1];

                }

**delete** [] dataCDOccurence;

**delete** [] dataNonDivCDOccurence ;

**delete** [] dataBDCFOccurence;

**delete** [] dataBDCFCDOccurence;

**delete** [] dataTDCFOccurence;

**delete** [] dataTDCFCDOccurence;

**delete** [] dataTDCDOccurence;

**delete** [] dataDoublingTimeBD;

**delete** [] dataDoublingTimeTD;

**delete** [] dataDoublingTimeCF;

**delete** [] arrayCellLineageTemp;

**delete** [] dataVariationToTheFirstDV;

                NSSound *sound = [NSSound soundNamed:@"Ping"];

                [sound play];

            }

**else**{

                NSAlert *alert = [[NSAlert alloc] init];

                [alert addButtonWithTitle:@"OK"];

                [alert setMessageText:@"List is empty"];

                [alert setAlertStyle:NSAlertStyleWarning];

                [alert runModal];

                [alert release];

                NSSound *sound = [NSSound soundNamed:@"Hero"];

                [sound play];

            }

        }

**else**{

            NSOpenPanel *openDlg = [NSOpenPanel openPanel];

            [openDlg setCanChooseFiles:**YES**];

            [openDlg setCanChooseDirectories:**YES**];

**if** ([openDlg runModal] == NSModalResponseOK){

                NSArray *files = [openDlg URLs];

                NSString *fileName = [[files objectAtIndex:0] absoluteString];

                string directryPathImport = [fileName UTF8String];

**int** findString1 = (**int**)directryPathImport.find("/Users/");

**if** (findString1 == -1) findString1 = (**int**)directryPathImport.find("/Volumes/");

**unsigned** **long** directoryLength = directryPathImport.length();

                directryPathImport = directryPathImport.substr((**unsigned** **long**)findString1, directoryLength-(**unsigned** **long**)findString1);

                string extreactedID;

                string extreactedID2;

**int** terminationFlag = 0;

**do**{

                    terminationFlag = 1;

                    findString1 = (**int**)directryPathImport.find("%20");

**if** (findString1 != -1){

                        extreactedID2 = directryPathImport.substr(0, (**unsigned** **long**)findString1);

                        directryPathImport = directryPathImport.substr((**unsigned** **long**)findString1+3);

                        directryPathImport = extreactedID2+" "+directryPathImport;

                    }

**else** terminationFlag = 0;

                } **while** (terminationFlag == 1);

                extreactedID = directryPathImport;

                string getString;

                ifstream fin;

                fin.open(extreactedID.c_str(),ios::**in**);

**if** (fin.is_open()){

                    getline(fin, getString);

**if** (getString == "Dose data array"){

                        getline(fin, getString);

                        simProcessDataProgHold [0] = atof(getString.c_str());

                        getline(fin, getString);

                        simProcessDataProgHold [1] = atof(getString.c_str());

                        getline(fin, getString);

                        simProcessDataProgHold [2] = atof(getString.c_str());

                        getline(fin, getString);

                        simProcessDataProgHold [3] = atof(getString.c_str());

                        getline(fin, getString);

                        simProcessDataProgHold [4] = atof(getString.c_str());

                        getline(fin, getString);

                        simProcessDataProgHold [5] = atof(getString.c_str());

                        getline(fin, getString);

                        simProcessDataProgHold [6] = atof(getString.c_str());

                        getline(fin, getString);

                        simProcessDataProgHold [7] = atof(getString.c_str());

                        getline(fin, getString);

                        simProcessDataProgHold [8] = atof(getString.c_str());

                        getline(fin, getString);

                        simProcessDataProgHold [9] = atof(getString.c_str());

                        getline(fin, getString);

                        simProcessDataProgHold [10] = atof(getString.c_str());

                        getline(fin, getString);

                        simProcessDataProgHold [11] = atof(getString.c_str());

                        getline(fin, getString);

                        simProcessDataProgHold [12] = atof(getString.c_str());

                        getline(fin, getString);

                        simProcessDataProgHold [13] = atof(getString.c_str());

                        getline(fin, getString);

                        simProcessDataProgHold [14] = atof(getString.c_str());

                        getline(fin, getString);

                        simProcessDataProgHold [15] = atof(getString.c_str());

                        getline(fin, getString);

                        simProcessDataProgHold [16] = atof(getString.c_str());

                        getline(fin, getString);

                        simProcessDataProgHold [17] = atof(getString.c_str());

                        getline(fin, getString);

                        simProcessDataProgHold [18] = atof(getString.c_str());

                        getline(fin, getString);

                        simProcessDataProgHold [19] = atof(getString.c_str());

                        getline(fin, getString);

                        simProcessDataProgHold [20] = atof(getString.c_str());

                        getline(fin, getString);

                        simProcessDataProgHold [21] = atof(getString.c_str());

                        getline(fin, getString);

                        simProcessDataProgHold [22] = atof(getString.c_str());

                        getline(fin, getString);

                        simProcessDataProgHold [23] = atof(getString.c_str());

                        getline(fin, getString);

                        simProcessDataProgHold [24] = atof(getString.c_str());

                        getline(fin, getString);

                        simProcessDataProgHold [25] = atof(getString.c_str());

                        getline(fin, getString);

                        simProcessDataProgHold [26] = atof(getString.c_str());

                        getline(fin, getString);

                        simProcessDataProgHold [27] = atof(getString.c_str());

                        [growthPrgTimeBDDisplay setDoubleValue:simProcessDataProgHold [0]];

                        [growthPrgTimeTDDisplay setDoubleValue:simProcessDataProgHold [1]];

                        [growthPrgTimeCFDisplay setDoubleValue:simProcessDataProgHold [2]];

**int** dataBD = (**int**)simProcessDataProgHold [3];

**int** dataTD = (**int**)simProcessDataProgHold [4];

**int** dataBDCFBD = (**int**)simProcessDataProgHold [8];

**int** dataBDCFTD = (**int**)simProcessDataProgHold [9];

**int** dataTDCFBD = (**int**)simProcessDataProgHold [12];

**int** dataTDCFTD = (**int**)simProcessDataProgHold [13];

**int** dataTDBD = (**int**)simProcessDataProgHold [15];

**int** dataTDTD = (**int**)simProcessDataProgHold [16];

**int** nonDivLing = (**int**)simProcessDataProgHold [20];

**int** recoveryGR = (**int**)simProcessDataProgHold [21];

**int** totalNumberOfDiv = (**int**)simProcessDataProgHold [23];

**int** numberOfLing = (**int**)simProcessDataProgHold [24];

**int** timePointMax = (**int**)simProcessDataProgHold [25];

**double** percentTemp = dataBD/(**double**)totalNumberOfDiv;

**int** percentInt = (**int**)(percentTemp*10000);

                        percentTemp = percentInt/(**double**)100;

                        [growthPrgBDDisplay setDoubleValue:percentTemp];

                        percentTemp = dataTD/(**double**)totalNumberOfDiv;

                        percentInt = (**int**)(percentTemp*10000);

                        percentTemp = percentInt/(**double**)100;

                        [growthPrgTDDisplay setDoubleValue:percentTemp];

                        [growthPrgBDCDDisplay setDoubleValue:simProcessDataProgHold [5]];

                        [growthPrgNOCDDisplay setDoubleValue:simProcessDataProgHold [6]];

                        [growthPrgBDCFDisplay setDoubleValue:simProcessDataProgHold [7]];

                        percentTemp = dataBDCFBD/(**double**)totalNumberOfDiv;

                        percentInt = (**int**)(percentTemp*10000);

                        percentTemp = percentInt/(**double**)100;

                        [growthPrgBDCFBDDisplay setDoubleValue:percentTemp];

                        percentTemp = dataBDCFTD/(**double**)totalNumberOfDiv;

                        percentInt = (**int**)(percentTemp*10000);

                        percentTemp = percentInt/(**double**)100;

                        [growthPrgBDCFTDDisplay setDoubleValue:percentTemp];

                        [growthPrgBDCFCDDisplay setDoubleValue:simProcessDataProgHold [10]];

                        [growthPrgTDCFDisplay setDoubleValue:simProcessDataProgHold [11]];

                        percentTemp = dataTDCFBD/(**double**)totalNumberOfDiv;

                        percentInt = (**int**)(percentTemp*10000);

                        percentTemp = percentInt/(**double**)100;

                        [growthPrgTDCFBDDisplay setDoubleValue:percentTemp];

                        percentTemp = dataTDCFTD/(**double**)totalNumberOfDiv;

                        percentInt = (**int**)(percentTemp*10000);

                        percentTemp = percentInt/(**double**)100;

                        [growthPrgTDCFTDDisplay setDoubleValue:percentTemp];

                        [growthPrgTDCFCDDisplay setDoubleValue:simProcessDataProgHold [14]];

                        percentTemp = dataTDBD/(**double**)totalNumberOfDiv;

                        percentInt = (**int**)(percentTemp*10000);

                        percentTemp = percentInt/(**double**)100;

                        [growthPrgTDBDDisplay setDoubleValue:percentTemp];

                        percentTemp = dataTDTD/(**double**)totalNumberOfDiv;

                        percentInt = (**int**)(percentTemp*10000);

                        percentTemp = percentInt/(**double**)100;

                        [growthPrgTDTDDisplay setDoubleValue:percentTemp];

                        [growthPrgTDCDDisplay setDoubleValue:simProcessDataProgHold [17]];

                        [growthPrgMulTDTDDisplay setDoubleValue:simProcessDataProgHold [18]];

                        [growthPrgSupTDBDDisplay setDoubleValue:simProcessDataProgHold [19]];

                        percentTemp = nonDivLing/(**double**)numberOfLing;

                        percentInt = (**int**)(percentTemp*10000);

                        percentTemp = percentInt/(**double**)100;

                        [growthPrgNoDivDisplay setDoubleValue:percentTemp];

                        percentTemp = recoveryGR/(**double**)numberOfLing;

                        percentInt = (**int**)(percentTemp*10000);

                        percentTemp = percentInt/(**double**)100;

                        [growthPrgRecovDisplay setDoubleValue:percentTemp];

**delete** [] simulationDistributionProgData;

                        simulationDistributionProgData = **new** **int** [timePointMax*11+100];

                        simulationDistributionProgDataCount = timePointMax*11;

**int** entryCount = 0;

                        getline(fin, getString);

**if** (getString == "A1"){

**do**{

                                terminationFlag = 1;

                                getline(fin, getString);

**if** (getString != "End"){

                                    simulationDistributionProgData [entryCount*11] = atoi(getString.c_str()), entryCount++;

                                }

**else** terminationFlag = 0;

                            } **while** (terminationFlag == 1);

                        }

                        getline(fin, getString);

                        entryCount = 0;

**if** (getString == "A2"){

**do**{

                                terminationFlag = 1;

                                getline(fin, getString);

**if** (getString != "End"){

                                    simulationDistributionProgData [entryCount*11+1] = atoi(getString.c_str()), entryCount++;

                                }

**else** terminationFlag = 0;

                            } **while** (terminationFlag == 1);

                        }

                        getline(fin, getString);

                        entryCount = 0;

**if** (getString == "A3"){

**do**{

                                terminationFlag = 1;

                                getline(fin, getString);

**if** (getString != "End"){

                                    simulationDistributionProgData [entryCount*11+2] = atoi(getString.c_str()), entryCount++;

                                }

**else** terminationFlag = 0;

                            } **while** (terminationFlag == 1);

                        }

                        getline(fin, getString);

                        entryCount = 0;

**if** (getString == "A4"){

**do**{

                                terminationFlag = 1;

                                getline(fin, getString);

**if** (getString != "End"){

                                    simulationDistributionProgData [entryCount*11+3] = atoi(getString.c_str()), entryCount++;

                                }

**else** terminationFlag = 0;

                            } **while** (terminationFlag == 1);

                        }

                        getline(fin, getString);

                        entryCount = 0;

**if** (getString == "A5"){

**do**{

                                terminationFlag = 1;

                                getline(fin, getString);

**if** (getString != "End"){

                                    simulationDistributionProgData [entryCount*11+4] = atoi(getString.c_str()), entryCount++;

                                }

**else** terminationFlag = 0;

                            } **while** (terminationFlag == 1);

                        }

                        getline(fin, getString);

                        entryCount = 0;

**if** (getString == "A6"){

**do**{

                                terminationFlag = 1;

                                getline(fin, getString);

**if** (getString != "End"){

                                    simulationDistributionProgData [entryCount*11+5] = atoi(getString.c_str()), entryCount++;

                                }

**else** terminationFlag = 0;

                            } **while** (terminationFlag == 1);

                        }

                        getline(fin, getString);

                        entryCount = 0;

**if** (getString == "A7"){

**do**{

                                terminationFlag = 1;

                                getline(fin, getString);

**if** (getString != "End"){

                                    simulationDistributionProgData [entryCount*11+6] = atoi(getString.c_str()), entryCount++;

                                }

**else** terminationFlag = 0;

                            } **while** (terminationFlag == 1);

                        }

                        getline(fin, getString);

                        entryCount = 0;

**if** (getString == "A8"){

**do**{

                                terminationFlag = 1;

                                getline(fin, getString);

**if** (getString != "End"){

                                    simulationDistributionProgData [entryCount*11+7] = atoi(getString.c_str()), entryCount++;

                                }

**else** terminationFlag = 0;

                            } **while** (terminationFlag == 1);

                        }

                        getline(fin, getString);

                        entryCount = 0;

**if** (getString == "A9"){

**do**{

                                terminationFlag = 1;

                                getline(fin, getString);

**if** (getString != "End"){

                                    simulationDistributionProgData [entryCount*11+8] = atoi(getString.c_str()), entryCount++;

                                }

**else** terminationFlag = 0;

                            } **while** (terminationFlag == 1);

                        }

                        getline(fin, getString);

                        entryCount = 0;

**if** (getString == "A10"){

**do**{

                                terminationFlag = 1;

                                getline(fin, getString);

**if** (getString != "End"){

                                    simulationDistributionProgData [entryCount*11+9] = atoi(getString.c_str()), entryCount++;

                                }

**else** terminationFlag = 0;

                            } **while** (terminationFlag == 1);

                        }

                        getline(fin, getString);

                        entryCount = 0;

**if** (getString == "A11"){

**do**{

                                terminationFlag = 1;

                                getline(fin, getString);

**if** (getString != "End"){

                                    simulationDistributionProgData [entryCount*11+10] = atoi(getString.c_str()), entryCount++;

                                }

**else** terminationFlag = 0;

                            } **while** (terminationFlag == 1);

                        }

                        NSSound *sound = [NSSound soundNamed:@"Ping"];

                        [sound play];

                    }

**else**{

                        NSAlert *alert = [[NSAlert alloc] init];

                        [alert addButtonWithTitle:@"OK"];

                        [alert setMessageText:@"No Sim database file"];

                        [alert setAlertStyle:NSAlertStyleWarning];

                        [alert runModal];

                        [alert release];

                        NSSound *sound = [NSSound soundNamed:@"Hero"];

                        [sound play];

                    }

                    fin.close();

                }

            }

        }

    }

**else**{

        NSAlert *alert = [[NSAlert alloc] init];

        [alert addButtonWithTitle:@"OK"];

        [alert setMessageText:@"Sim is in progress"];

        [alert setAlertStyle:NSAlertStyleWarning];

        [alert runModal];

        [alert release];

        NSSound *sound = [NSSound soundNamed:@"Hero"];

        [sound play];

    }

}

-(**IBAction**)getListGrowthMidSet:(**id**)sender{

**if** (simulationprogress == 0){

**if** (databaseLoadOptionHold == 0){

**if** (simListHoldCount != 0){

                messageStringSim3 = arraySimListHold [simOperationTableCurrentRow*2+1].substr(arraySimListHold [simOperationTableCurrentRow*2+1].find("-")+1);

                NSString *treatPickUpNameNSString = @(messageStringSim3.c_str());

                [growthMidTreatDisplay setStringValue: treatPickUpNameNSString];

**int** *arrayCellLineageTemp = **new** **int** [(arrayLineageDataEntryHold [simOperationTableCurrentRow]/9)*9+100];

**int** cellLineageTempCount = 0;

**for** (**unsigned** **long** counter3 = 0; counter3 < (arrayLineageDataEntryHold [simOperationTableCurrentRow]/9)*9+100; counter3++){

                    arrayCellLineageTemp [counter3] = 0;

                }

**for** (**unsigned** **long** counter3 = 0; counter3 < arrayLineageDataEntryHold [simOperationTableCurrentRow]/9; counter3++){

                    arrayCellLineageTemp [cellLineageTempCount] = arrayLineageData [simOperationTableCurrentRow][counter3*9], cellLineageTempCount++;

                    arrayCellLineageTemp [cellLineageTempCount] = arrayLineageData [simOperationTableCurrentRow][counter3*9+1], cellLineageTempCount++;

                    arrayCellLineageTemp [cellLineageTempCount] = arrayLineageData [simOperationTableCurrentRow][counter3*9+2], cellLineageTempCount++;

                    arrayCellLineageTemp [cellLineageTempCount] = arrayLineageData [simOperationTableCurrentRow][counter3*9+3], cellLineageTempCount++;

                    arrayCellLineageTemp [cellLineageTempCount] = arrayLineageData [simOperationTableCurrentRow][counter3*9+4], cellLineageTempCount++;

                    arrayCellLineageTemp [cellLineageTempCount] = arrayLineageData [simOperationTableCurrentRow][counter3*9+5], cellLineageTempCount++;

                    arrayCellLineageTemp [cellLineageTempCount] = arrayLineageData [simOperationTableCurrentRow][counter3*9+6], cellLineageTempCount++;

                    arrayCellLineageTemp [cellLineageTempCount] = arrayLineageData [simOperationTableCurrentRow][counter3*9+7], cellLineageTempCount++;

                    arrayCellLineageTemp [cellLineageTempCount] = arrayLineageData [simOperationTableCurrentRow][counter3*9+8], cellLineageTempCount++;

                }

**for** (**int** counter3 = 0; counter3 < cellLineageTempCount/9; counter3++){

**if** (arrayCellLineageTemp [counter3*9+3] == 92){

**for** (**int** counter4 = counter3-1; counter4 >= 0; counter4--){

**if** (arrayCellLineageTemp [counter4*9+3] == 92){

                                arrayCellLineageTemp [counter4*9+3] = 2;

                            }

**else** **if** (arrayCellLineageTemp [counter4*9+3] == 1 || arrayCellLineageTemp [counter4*9+3] == 31 || arrayCellLineageTemp [counter4*9+3] == 41 || arrayCellLineageTemp [counter4*9+3] == 51){

**break**;

                            }

                        }

                    }

                }

**int** timePointMax = arrayTableDetail [simOperationTableCurrentRow][3];

**int** twentyPercentOfMax = timePointMax-(**int**)(round(timePointMax*(**double**)(recoveryCalcPercentHold/(**double**)100)));

**int** *dataCDOccurence = **new** **int** [timePointMax+10];

**int** *dataNonDivCDOccurence = **new** **int** [timePointMax+10];

**int** *dataBDCFOccurence = **new** **int** [timePointMax+10];

**int** *dataBDCFCDOccurence = **new** **int** [timePointMax+10];

**int** *dataTDCFOccurence = **new** **int** [timePointMax+10];

**int** *dataTDCFCDOccurence = **new** **int** [timePointMax+10];

**int** *dataTDCDOccurence = **new** **int** [timePointMax+10];

**int** *dataVariationToTheFirstDV = **new** **int** [timePointMax+10];

**for** (**int** counter3 = 0; counter3 < timePointMax+10; counter3++){

                    dataCDOccurence [counter3] = 0;

                    dataNonDivCDOccurence [counter3] = 0;

                    dataBDCFOccurence [counter3] = 0;

                    dataBDCFCDOccurence [counter3] = 0;

                    dataTDCFOccurence [counter3] = 0;

                    dataTDCFCDOccurence [counter3] = 0;

                    dataTDCDOccurence [counter3] = 0;

                    dataVariationToTheFirstDV [counter3] = 0;

                }

**int** dataBD = 0;

**int** dataTD = 0;

**int** dataBDCFBD = 0;

**int** dataBDCFTD = 0;

**int** dataTDCFBD = 0;

**int** dataTDCFTD = 0;

**int** dataTDBD = 0;

**int** dataTDTD = 0;

**int** nonDivLing = 0;

**int** recoveryGR = 0;

**int** dataCD = 0;

**double** dataBDRand = 0;

**double** dataCDRand = 0;

**for** (**int** counter3 = 0; counter3 < cellLineageTempCount/9; counter3++){

**if** (arrayCellLineageTemp [counter3*9+3] == 32){

                        dataBD++;

                        dataBDRand = dataBDRand+arrayCellLineageTemp [counter3*9+2];

                    }

**if** (arrayCellLineageTemp [counter3*9+3] == 42 || arrayCellLineageTemp [counter3*9+3] == 52) dataTD++;

**if** (arrayCellLineageTemp [counter3*9+3] == 7){

**for** (**int** counter4 = counter3; counter4 >= 0; counter4--){

**if** (arrayCellLineageTemp [counter4*9+3] == 31){

                                dataCDOccurence [counter3-counter4]++;

**break**;

                            }

**else** **if** (arrayCellLineageTemp [counter4*9+3] == 1 || arrayCellLineageTemp [counter4*9+3] == 41 || arrayCellLineageTemp [counter4*9+3] == 51 || arrayCellLineageTemp [counter4*9+3] == 92){

**break**;

                            }

                        }

                        dataCD++;

                        dataCDRand = dataCDRand+arrayCellLineageTemp [counter3*9+2];

                    }

**if** (arrayCellLineageTemp [counter3*9+3] == 7){

**for** (**int** counter4 = counter3; counter4 >= 0; counter4--){

**if** (arrayCellLineageTemp [counter4*9+3] == 1){

                                dataNonDivCDOccurence [counter3-counter4]++;

**break**;

                            }

**else** **if** (arrayCellLineageTemp [counter4*9+3] == 31 || arrayCellLineageTemp [counter4*9+3] == 41 || arrayCellLineageTemp [counter4*9+3] == 51 || arrayCellLineageTemp [counter4*9+3] == 92){

**break**;

                            }

                        }

                    }

**if** (arrayCellLineageTemp [counter3*9+3] == 91){

**for** (**int** counter4 = counter3; counter4 >= 0; counter4--){

**if** (arrayCellLineageTemp [counter4*9+3] == 31){

                                dataBDCFOccurence [counter3-counter4]++;

**break**;

                            }

**else** **if** (arrayCellLineageTemp [counter4*9+3] == 1 || arrayCellLineageTemp [counter4*9+3] == 41 || arrayCellLineageTemp [counter4*9+3] == 51 || arrayCellLineageTemp [counter4*9+3] == 92){

**break**;

                            }

                        }

                    }

**if** (arrayCellLineageTemp [counter3*9+3] == 92){

**for** (**int** counter4 = counter3; counter4 < cellLineageTempCount/9; counter4++){

**if** (arrayCellLineageTemp [counter4*9+3] == 32){

**for** (**int** counter5 = counter3; counter5 >= 0; counter5--){

**if** (arrayCellLineageTemp [counter5*9+3] == 31){

                                        dataBDCFBD++;

**break**;

                                    }

**else** **if** (arrayCellLineageTemp [counter5*9+3] == 1 || arrayCellLineageTemp [counter5*9+3] == 41 || arrayCellLineageTemp [counter5*9+3] == 51){

**break**;

                                    }

                                }

**break**;

                            }

**else** **if** ((arrayCellLineageTemp [counter3*9+5] != arrayCellLineageTemp [counter4*9+5] || arrayCellLineageTemp [counter3*9+6] != arrayCellLineageTemp [counter4*9+6]) || arrayCellLineageTemp [counter4*9+3] == 8 || arrayCellLineageTemp [counter4*9+3] == 42 || arrayCellLineageTemp [counter4*9+3] == 52 || arrayCellLineageTemp [counter4*9+3] == 91 || arrayCellLineageTemp [counter4*9+2] == timePointMax){

**break**;

                            }

                        }

                    }

**if** (arrayCellLineageTemp [counter3*9+3] == 92){

**for** (**int** counter4 = counter3; counter4 < cellLineageTempCount/9; counter4++){

**if** (arrayCellLineageTemp [counter4*9+3] == 42 || arrayCellLineageTemp [counter4*9+3] == 52){

**for** (**int** counter5 = counter3; counter5 >= 0; counter5--){

**if** (arrayCellLineageTemp [counter5*9+3] == 31){

                                        dataBDCFTD++;

**break**;

                                    }

**else** **if** (arrayCellLineageTemp [counter5*9+3] == 1 || arrayCellLineageTemp [counter5*9+3] == 41 || arrayCellLineageTemp [counter5*9+3] == 51){

**break**;

                                    }

                                }

**break**;

                            }

**else** **if** ((arrayCellLineageTemp [counter3*9+5] != arrayCellLineageTemp [counter4*9+5] || arrayCellLineageTemp [counter3*9+6] != arrayCellLineageTemp [counter4*9+6]) || arrayCellLineageTemp [counter4*9+3] == 8 || arrayCellLineageTemp [counter4*9+3] == 32 || arrayCellLineageTemp [counter4*9+3] == 91 || arrayCellLineageTemp [counter4*9+2] == timePointMax){

**break**;

                            }

                        }

                    }

**if** (arrayCellLineageTemp [counter3*9+3] == 92){

**for** (**int** counter4 = counter3; counter4 < cellLineageTempCount/9; counter4++){

**if** (arrayCellLineageTemp [counter4*9+3] == 7){

**for** (**int** counter5 = counter3; counter5 >= 0; counter5--){

**if** (arrayCellLineageTemp [counter5*9+3] == 31){

                                        dataBDCFCDOccurence [counter4-counter3]++;

**break**;

                                    }

**else** **if** (arrayCellLineageTemp [counter5*9+3] == 1 || arrayCellLineageTemp [counter5*9+3] == 41 || arrayCellLineageTemp [counter5*9+3] == 51){

**break**;

                                    }

                                }

**break**;

                            }

**else** **if** ((arrayCellLineageTemp [counter3*9+5] != arrayCellLineageTemp [counter4*9+5] || arrayCellLineageTemp [counter3*9+6] != arrayCellLineageTemp [counter4*9+6]) || arrayCellLineageTemp [counter4*9+3] == 8 || arrayCellLineageTemp [counter4*9+3] == 32 || arrayCellLineageTemp [counter4*9+3] == 42 || arrayCellLineageTemp [counter4*9+3] == 52 || arrayCellLineageTemp [counter4*9+3] == 91 || arrayCellLineageTemp [counter4*9+2] == timePointMax){

**break**;

                            }

                        }

                    }

**if** (arrayCellLineageTemp [counter3*9+3] == 91){

**for** (**int** counter4 = counter3; counter4 >= 0; counter4--){

**if** (arrayCellLineageTemp [counter4*9+3] == 41 || arrayCellLineageTemp [counter4*9+3] == 51){

                                dataTDCFOccurence [counter3-counter4]++;

**break**;

                            }

**else** **if** (arrayCellLineageTemp [counter4*9+3] == 1 || arrayCellLineageTemp [counter4*9+3] == 31 || arrayCellLineageTemp [counter4*9+3] == 92){

**break**;

                            }

                        }

                    }

**if** (arrayCellLineageTemp [counter3*9+3] == 92){

**for** (**int** counter4 = counter3; counter4 < cellLineageTempCount/9; counter4++){

**if** (arrayCellLineageTemp [counter4*9+3] == 32){

**for** (**int** counter5 = counter3; counter5 >= 0; counter5--){

**if** (arrayCellLineageTemp [counter5*9+3] == 41 || arrayCellLineageTemp [counter5*9+3] == 51){

                                        dataTDCFBD++;

**break**;

                                    }

**else** **if** (arrayCellLineageTemp [counter5*9+3] == 1 || arrayCellLineageTemp [counter5*9+3] == 31){

**break**;

                                    }

                                }

**break**;

                            }

**else** **if** ((arrayCellLineageTemp [counter3*9+5] != arrayCellLineageTemp [counter4*9+5] || arrayCellLineageTemp [counter3*9+6] != arrayCellLineageTemp [counter4*9+6]) || arrayCellLineageTemp [counter4*9+3] == 8 || arrayCellLineageTemp [counter4*9+3] == 42 || arrayCellLineageTemp [counter4*9+3] == 52 || arrayCellLineageTemp [counter4*9+3] == 91 || arrayCellLineageTemp [counter4*9+2] == timePointMax){

**break**;

                            }

                        }

                    }

**if** (arrayCellLineageTemp [counter3*9+3] == 92){

**for** (**int** counter4 = counter3; counter4 < cellLineageTempCount/9; counter4++){

**if** (arrayCellLineageTemp [counter4*9+3] == 42 || arrayCellLineageTemp [counter4*9+3] == 52){

**for** (**int** counter5 = counter3; counter5 >= 0; counter5--){

**if** (arrayCellLineageTemp [counter5*9+3] == 41 || arrayCellLineageTemp [counter5*9+3] == 51){

                                        dataTDCFTD++;

**break**;

                                    }

**else** **if** (arrayCellLineageTemp [counter5*9+3] == 1 || arrayCellLineageTemp [counter5*9+3] == 31){

**break**;

                                    }

                                }

**break**;

                            }

**else** **if** ((arrayCellLineageTemp [counter3*9+5] != arrayCellLineageTemp [counter4*9+5] || arrayCellLineageTemp [counter3*9+6] != arrayCellLineageTemp [counter4*9+6]) || arrayCellLineageTemp [counter4*9+3] == 8 || arrayCellLineageTemp [counter4*9+3] == 32 || arrayCellLineageTemp [counter4*9+3] == 91 || arrayCellLineageTemp [counter4*9+2] == timePointMax){

**break**;

                            }

                        }

                    }

**if** (arrayCellLineageTemp [counter3*9+3] == 92){

**for** (**int** counter4 = counter3; counter4 < cellLineageTempCount/9; counter4++){

**if** (arrayCellLineageTemp [counter4*9+3] == 7){

**for** (**int** counter5 = counter3; counter5 >= 0; counter5--){

**if** (arrayCellLineageTemp [counter5*9+3] == 41 || arrayCellLineageTemp [counter5*9+3] == 51){

                                        dataTDCFCDOccurence [counter4-counter3]++;

**break**;

                                    }

**else** **if** (arrayCellLineageTemp [counter5*9+3] == 1 || arrayCellLineageTemp [counter5*9+3] == 31){

**break**;

                                    }

                                }

**break**;

                            }

**else** **if** ((arrayCellLineageTemp [counter3*9+5] != arrayCellLineageTemp [counter4*9+5] || arrayCellLineageTemp [counter3*9+6] != arrayCellLineageTemp [counter4*9+6]) || arrayCellLineageTemp [counter4*9+3] == 8 || arrayCellLineageTemp [counter4*9+3] == 32 || arrayCellLineageTemp [counter4*9+3] == 42 || arrayCellLineageTemp [counter4*9+3] == 52 || arrayCellLineageTemp [counter4*9+3] == 91 || arrayCellLineageTemp [counter4*9+2] == timePointMax){

**break**;

                            }

                        }

                    }

**if** (arrayCellLineageTemp [counter3*9+3] == 41 || arrayCellLineageTemp [counter3*9+3] == 51){

**for** (**int** counter4 = counter3; counter4 < cellLineageTempCount/9; counter4++){

**if** (arrayCellLineageTemp [counter4*9+3] == 32){

                                dataTDBD++;

**break**;

                            }

**else** **if** ((arrayCellLineageTemp [counter3*9+5] != arrayCellLineageTemp [counter4*9+5] || arrayCellLineageTemp [counter3*9+6] != arrayCellLineageTemp [counter4*9+6]) || arrayCellLineageTemp [counter4*9+3] == 8 || arrayCellLineageTemp [counter4*9+3] == 7 || arrayCellLineageTemp [counter4*9+3] == 42 || arrayCellLineageTemp [counter4*9+3] == 52 || arrayCellLineageTemp [counter4*9+3] == 91 || arrayCellLineageTemp [counter4*9+3] == 92 || arrayCellLineageTemp [counter4*9+2] == timePointMax){

**break**;

                            }

                        }

                    }

**if** (arrayCellLineageTemp [counter3*9+3] == 41 || arrayCellLineageTemp [counter3*9+3] == 51){

**for** (**int** counter4 = counter3; counter4 < cellLineageTempCount/9; counter4++){

**if** (arrayCellLineageTemp [counter4*9+3] == 42 || arrayCellLineageTemp [counter4*9+3] == 52){

                                dataTDTD++;

**break**;

                            }

**else** **if** ((arrayCellLineageTemp [counter3*9+5] != arrayCellLineageTemp [counter4*9+5] || arrayCellLineageTemp [counter3*9+6] != arrayCellLineageTemp [counter4*9+6]) || arrayCellLineageTemp [counter4*9+3] == 8 || arrayCellLineageTemp [counter4*9+3] == 7 || arrayCellLineageTemp [counter4*9+3] == 32 || arrayCellLineageTemp [counter4*9+3] == 91 || arrayCellLineageTemp [counter4*9+3] == 92 || arrayCellLineageTemp [counter4*9+2] == timePointMax){

**break**;

                            }

                        }

                    }

**if** (arrayCellLineageTemp [counter3*9+3] == 7){

**for** (**int** counter4 = counter3; counter4 >= 0; counter4--){

**if** (arrayCellLineageTemp [counter4*9+3] == 41 || arrayCellLineageTemp [counter4*9+3] == 51){

                                dataTDCDOccurence [counter3-counter4]++;

**break**;

                            }

**else** **if** (arrayCellLineageTemp [counter4*9+3] == 1 || arrayCellLineageTemp [counter4*9+3] == 31 || arrayCellLineageTemp [counter4*9+3] == 92){

**break**;

                            }

                        }

                    }

**if** (arrayCellLineageTemp [counter3*9+3] == 1){

**for** (**int** counter4 = counter3; counter4 < cellLineageTempCount/9; counter4++){

**if** (arrayCellLineageTemp [counter4*9+2] == timePointMax){

                                nonDivLing++;

**break**;

                            }

**else** **if** ((arrayCellLineageTemp [counter3*9+5] != arrayCellLineageTemp [counter4*9+5] || arrayCellLineageTemp [counter3*9+6] != arrayCellLineageTemp [counter4*9+6]) || arrayCellLineageTemp [counter4*9+3] == 8 || arrayCellLineageTemp [counter4*9+3] == 7 || arrayCellLineageTemp [counter4*9+3] == 32 || arrayCellLineageTemp [counter4*9+3] == 42 || arrayCellLineageTemp [counter4*9+3] == 52 || arrayCellLineageTemp [counter4*9+3] == 91 || arrayCellLineageTemp [counter4*9+2] == timePointMax){

**break**;

                            }

                        }

                    }

**if** (arrayCellLineageTemp [counter3*9+2] > twentyPercentOfMax && arrayCellLineageTemp [counter3*9+3] == 32){

**for** (**int** counter4 = counter3; counter4 >= 0; counter4--){

**if** (arrayCellLineageTemp [counter4*9+3] == 1){

                                recoveryGR++;

**break**;

                            }

**else** **if** (arrayCellLineageTemp [counter4*9+3] == 31 || arrayCellLineageTemp [counter4*9+3] == 41 || arrayCellLineageTemp [counter4*9+3] == 51 || arrayCellLineageTemp [counter4*9+3] == 92){

**break**;

                            }

                        }

                    }

**if** (arrayCellLineageTemp [counter3*9+3] == 1){

**for** (**int** counter4 = counter3; counter4 < cellLineageTempCount/9; counter4++){

**if** (arrayCellLineageTemp [counter4*9+3] == 7 || arrayCellLineageTemp [counter4*9+3] == 32 || arrayCellLineageTemp [counter4*9+3] == 42 || arrayCellLineageTemp [counter4*9+3] == 52 || arrayCellLineageTemp [counter4*9+3] == 91 || arrayCellLineageTemp [counter4*9+2] == timePointMax){

                                dataVariationToTheFirstDV [counter4-counter3]++;

**break**;

                            }

**else** **if** ((arrayCellLineageTemp [counter3*9+5] != arrayCellLineageTemp [counter4*9+5] || arrayCellLineageTemp [counter3*9+6] != arrayCellLineageTemp [counter4*9+6]) || arrayCellLineageTemp [counter4*9+3] == 8){

**break**;

                            }

                        }

                    }

                }

**if** (dataBD != 0) dataBDRand = dataBDRand/(**double**)dataBD;

**if** (dataCD != 0) dataCDRand = dataCDRand/(**double**)dataCD;

**int** *dataDoublingTimeBD = **new** **int** [timePointMax+50];

**int** *dataDoublingTimeTD = **new** **int** [timePointMax+50];

**int** *dataDoublingTimeCF = **new** **int** [timePointMax+50];

**for** (**int** counter3 = 0; counter3 < timePointMax+10; counter3++){

                    dataDoublingTimeBD [counter3] = 0;

                    dataDoublingTimeTD [counter3] = 0;

                    dataDoublingTimeCF [counter3] = 0;

                }

**int** endTime = 0;

**int** startTime = 0;

**for** (**int** counter3 = 0; counter3 < cellLineageTempCount/9; counter3++){

**if** (arrayCellLineageTemp [counter3*9+3] == 31){

                        endTime = 0;

**for** (**int** counter4 = counter3+1; counter4 < cellLineageTempCount/9; counter4++){

**if** (arrayCellLineageTemp [counter3*9+5] == arrayCellLineageTemp [counter4*9+5] && arrayCellLineageTemp [counter3*9+6] == arrayCellLineageTemp [counter4*9+6] && (arrayCellLineageTemp [counter4*9+3] == 32 || arrayCellLineageTemp [counter4*9+3] == 42 || arrayCellLineageTemp [counter4*9+3] == 52)){

                                endTime = arrayCellLineageTemp [counter4*9+2];

                            }

**else** **if** (arrayCellLineageTemp [counter3*9+5] != arrayCellLineageTemp [counter4*9+5] || arrayCellLineageTemp [counter3*9+6] != arrayCellLineageTemp [counter4*9+6]){

**break**;

                            }

                        }

**if** (endTime != 0){

                            dataDoublingTimeBD [endTime-arrayCellLineageTemp [counter3*9+2]]++;

                        }

                    }

**if** (arrayCellLineageTemp [counter3*9+3] == 41 || arrayCellLineageTemp [counter3*9+3] == 51){

                        endTime = 0;

**for** (**int** counter4 = counter3+1; counter4 < cellLineageTempCount/9; counter4++){

**if** (arrayCellLineageTemp [counter3*9+5] == arrayCellLineageTemp [counter4*9+5] && arrayCellLineageTemp [counter3*9+6] == arrayCellLineageTemp [counter4*9+6] && (arrayCellLineageTemp [counter4*9+3] == 32 || arrayCellLineageTemp [counter4*9+3] == 42 || arrayCellLineageTemp [counter4*9+3] == 52)){

                                endTime = arrayCellLineageTemp [counter4*9+2];

                            }

**else** **if** (arrayCellLineageTemp [counter3*9+5] != arrayCellLineageTemp [counter4*9+5] || arrayCellLineageTemp [counter3*9+6] != arrayCellLineageTemp [counter4*9+6]){

**break**;

                            }

                        }

**if** (endTime != 0){

                            dataDoublingTimeTD [endTime-arrayCellLineageTemp [counter3*9+2]]++;

                        }

                    }

**if** (arrayCellLineageTemp [counter3*9+3] == 92){

                        startTime = 0;

**for** (**int** counter4 = counter3; counter4 >= 0; counter4--){

**if** (arrayCellLineageTemp [counter4*9+3] == 31 || arrayCellLineageTemp [counter4*9+3] == 41 || arrayCellLineageTemp [counter4*9+3] == 51){

                                startTime = arrayCellLineageTemp [counter4*9+2];

**break**;

                            }

**else** **if** ((arrayCellLineageTemp [counter3*9+5] != arrayCellLineageTemp [counter4*9+5] || arrayCellLineageTemp [counter3*9+6] != arrayCellLineageTemp [counter4*9+6]) || arrayCellLineageTemp [counter4*9+3] == 1){

**break**;

                            }

                        }

                        endTime = 0;

**for** (**int** counter4 = counter3+1; counter4 < cellLineageTempCount/9; counter4++){

**if** (arrayCellLineageTemp [counter3*9+5] == arrayCellLineageTemp [counter4*9+5] && arrayCellLineageTemp [counter3*9+6] == arrayCellLineageTemp [counter4*9+6] && (arrayCellLineageTemp [counter4*9+3] == 32 || arrayCellLineageTemp [counter4*9+3] == 42 || arrayCellLineageTemp [counter4*9+3] == 52)){

                                endTime = arrayCellLineageTemp [counter4*9+2];

                            }

**else** **if** (arrayCellLineageTemp [counter3*9+5] != arrayCellLineageTemp [counter4*9+5] || arrayCellLineageTemp [counter3*9+6] != arrayCellLineageTemp [counter4*9+6]){

**break**;

                            }

                        }

**if** (startTime != 0 && endTime != 0){

                            dataDoublingTimeCF [endTime-startTime]++;

                        }

                    }

                }

**int** numberOfLing = arrayTableDetail [simOperationTableCurrentRow][1];

**int** totalNumberOfDiv = arrayTableDetail [simOperationTableCurrentRow][13];

                simProcessDataMiddleHold [0] = 1;

                simProcessDataMiddleHold [1] = 1;

                simProcessDataMiddleHold [2] = 1;

                simProcessDataMiddleHold [3] = dataBD;

                simProcessDataMiddleHold [4] = dataTD;

                simProcessDataMiddleHold [5] = 1;

                simProcessDataMiddleHold [6] = 1;

                simProcessDataMiddleHold [7] = 1;

                simProcessDataMiddleHold [8] = dataBDCFBD;

                simProcessDataMiddleHold [9] = dataBDCFTD;

                simProcessDataMiddleHold [10] = 1;

                simProcessDataMiddleHold [11] = 1;

                simProcessDataMiddleHold [12] = dataTDCFBD;

                simProcessDataMiddleHold [13] = dataTDCFTD;

                simProcessDataMiddleHold [14] = 1;

                simProcessDataMiddleHold [15] = dataTDBD;

                simProcessDataMiddleHold [16] = dataTDTD;

                simProcessDataMiddleHold [17] = 1;

                simProcessDataMiddleHold [18] = 2;

                simProcessDataMiddleHold [19] = 3;

                simProcessDataMiddleHold [20] = nonDivLing;

                simProcessDataMiddleHold [21] = recoveryGR;

                simProcessDataMiddleHold [22] = recoveryCalcPercentHold;

                simProcessDataMiddleHold [23] = totalNumberOfDiv;

                simProcessDataMiddleHold [24] = numberOfLing;

                simProcessDataMiddleHold [25] = timePointMax;

                simProcessDataMiddleHold [26] = dataBDRand;

                simProcessDataMiddleHold [27] = dataCDRand;

                [growthMidTimeBDDisplay setDoubleValue:simProcessDataMiddleHold [0]];

                [growthMidTimeTDDisplay setDoubleValue:simProcessDataMiddleHold [1]];

                [growthMidTimeCFDisplay setDoubleValue:simProcessDataMiddleHold [2]];

**double** percentTemp = dataBD/(**double**)totalNumberOfDiv;

**int** percentInt = (**int**)(percentTemp*10000);

                percentTemp = percentInt/(**double**)100;

                [growthMidBDDisplay setDoubleValue:percentTemp];

                percentTemp = dataTD/(**double**)totalNumberOfDiv;

                percentInt = (**int**)(percentTemp*10000);

                percentTemp = percentInt/(**double**)100;

                [growthMidTDDisplay setDoubleValue:percentTemp];

                [growthMidBDCDDisplay setDoubleValue:simProcessDataMiddleHold [5]];

                [growthMidNOCDDisplay setDoubleValue:simProcessDataMiddleHold [6]];

                [growthMidBDCFDisplay setDoubleValue:simProcessDataMiddleHold [7]];

                percentTemp = dataBDCFBD/(**double**)totalNumberOfDiv;

                percentInt = (**int**)(percentTemp*10000);

                percentTemp = percentInt/(**double**)100;

                [growthMidBDCFBDDisplay setDoubleValue:percentTemp];

                percentTemp = dataBDCFTD/(**double**)totalNumberOfDiv;

                percentInt = (**int**)(percentTemp*10000);

                percentTemp = percentInt/(**double**)100;

                [growthMidBDCFTDDisplay setDoubleValue:percentTemp];

                [growthMidBDCFCDDisplay setDoubleValue:simProcessDataMiddleHold [10]];

                [growthMidTDCFDisplay setDoubleValue:simProcessDataMiddleHold [11]];

                percentTemp = dataTDCFBD/(**double**)totalNumberOfDiv;

                percentInt = (**int**)(percentTemp*10000);

                percentTemp = percentInt/(**double**)100;

                [growthMidTDCFBDDisplay setDoubleValue:percentTemp];

                percentTemp = dataTDCFTD/(**double**)totalNumberOfDiv;

                percentInt = (**int**)(percentTemp*10000);

                percentTemp = percentInt/(**double**)100;

                [growthMidTDCFTDDisplay setDoubleValue:percentTemp];

                [growthMidTDCFCDDisplay setDoubleValue:simProcessDataMiddleHold [14]];

                percentTemp = dataTDBD/(**double**)totalNumberOfDiv;

                percentInt = (**int**)(percentTemp*10000);

                percentTemp = percentInt/(**double**)100;

                [growthMidTDBDDisplay setDoubleValue:percentTemp];

                percentTemp = dataTDTD/(**double**)totalNumberOfDiv;

                percentInt = (**int**)(percentTemp*10000);

                percentTemp = percentInt/(**double**)100;

                [growthMidTDTDDisplay setDoubleValue:percentTemp];

                [growthMidTDCDDisplay setDoubleValue:simProcessDataMiddleHold [17]];

                [growthMidMulTDTDDisplay setDoubleValue:simProcessDataMiddleHold [18]];

                [growthMidSupTDBDDisplay setDoubleValue:simProcessDataMiddleHold [19]];

                percentTemp = nonDivLing/(**double**)numberOfLing;

                percentInt = (**int**)(percentTemp*10000);

                percentTemp = percentInt/(**double**)100;

                [growthMidNoDivDisplay setDoubleValue:percentTemp];

                percentTemp = recoveryGR/(**double**)numberOfLing;

                percentInt = (**int**)(percentTemp*10000);

                percentTemp = percentInt/(**double**)100;

                [growthMidRecovDisplay setDoubleValue:percentTemp];

**delete** [] simulationDistributionMidData;

                simulationDistributionMidData = **new** **int** [timePointMax*11+100];

                simulationDistributionMidDataCount = timePointMax*11;

**for** (**int** counter1 = 0; counter1 < timePointMax; counter1++){

                    simulationDistributionMidData [counter1*11] = dataCDOccurence [counter1];

                    simulationDistributionMidData [counter1*11+1] = dataNonDivCDOccurence [counter1];

                    simulationDistributionMidData [counter1*11+2] = dataBDCFOccurence [counter1];

                    simulationDistributionMidData [counter1*11+3] = dataBDCFCDOccurence [counter1];

                    simulationDistributionMidData [counter1*11+4] = dataTDCFOccurence [counter1];

                    simulationDistributionMidData [counter1*11+5] = dataTDCFCDOccurence [counter1];

                    simulationDistributionMidData [counter1*11+6] = dataTDCDOccurence [counter1];

                    simulationDistributionMidData [counter1*11+7] = dataDoublingTimeBD [counter1];

                    simulationDistributionMidData [counter1*11+8] = dataDoublingTimeTD [counter1];

                    simulationDistributionMidData [counter1*11+9] = dataDoublingTimeCF [counter1];

                    simulationDistributionMidData [counter1*11+10] = dataVariationToTheFirstDV [counter1];

                }

**delete** [] dataCDOccurence;

**delete** [] dataNonDivCDOccurence ;

**delete** [] dataBDCFOccurence;

**delete** [] dataBDCFCDOccurence;

**delete** [] dataTDCFOccurence;

**delete** [] dataTDCFCDOccurence;

**delete** [] dataTDCDOccurence;

**delete** [] dataDoublingTimeBD;

**delete** [] dataDoublingTimeTD;

**delete** [] dataDoublingTimeCF;

**delete** [] arrayCellLineageTemp;

**delete** [] dataVariationToTheFirstDV;

                NSSound *sound = [NSSound soundNamed:@"Ping"];

                [sound play];

            }

**else**{

                NSAlert *alert = [[NSAlert alloc] init];

                [alert addButtonWithTitle:@"OK"];

                [alert setMessageText:@"List is empty"];

                [alert setAlertStyle:NSAlertStyleWarning];

                [alert runModal];

                [alert release];

                NSSound *sound = [NSSound soundNamed:@"Hero"];

                [sound play];

            }

        }

**else**{

            NSOpenPanel *openDlg = [NSOpenPanel openPanel];

            [openDlg setCanChooseFiles:**YES**];

            [openDlg setCanChooseDirectories:**YES**];

**if** ([openDlg runModal] == NSModalResponseOK){

                NSArray *files = [openDlg URLs];

                NSString *fileName = [[files objectAtIndex:0] absoluteString];

                string directryPathImport = [fileName UTF8String];

**int** findString1 = (**int**)directryPathImport.find("/Users/");

**if** (findString1 == -1) findString1 = (**int**)directryPathImport.find("/Volumes/");

**unsigned** **long** directoryLength = directryPathImport.length();

                directryPathImport = directryPathImport.substr((**unsigned** **long**)findString1, directoryLength-(**unsigned** **long**)findString1);

                string extreactedID;

                string extreactedID2;

**int** terminationFlag = 0;

**do**{

                    terminationFlag = 1;

                    findString1 = (**int**)directryPathImport.find("%20");

**if** (findString1 != -1){

                        extreactedID2 = directryPathImport.substr(0, (**unsigned** **long**)findString1);

                        directryPathImport = directryPathImport.substr((**unsigned** **long**)findString1+3);

                        directryPathImport = extreactedID2+" "+directryPathImport;

                    }

**else** terminationFlag = 0;

                } **while** (terminationFlag == 1);

                extreactedID = directryPathImport;

                string getString;

                ifstream fin;

                fin.open(extreactedID.c_str(),ios::**in**);

**if** (fin.is_open()){

                    getline(fin, getString);

**if** (getString == "Dose data array"){

                        getline(fin, getString);

                        simProcessDataMiddleHold [0] = atof(getString.c_str());

                        getline(fin, getString);

                        simProcessDataMiddleHold [1] = atof(getString.c_str());

                        getline(fin, getString);

                        simProcessDataMiddleHold [2] = atof(getString.c_str());

                        getline(fin, getString);

                        simProcessDataMiddleHold [3] = atof(getString.c_str());

                        getline(fin, getString);

                        simProcessDataMiddleHold [4] = atof(getString.c_str());

                        getline(fin, getString);

                        simProcessDataMiddleHold [5] = atof(getString.c_str());

                        getline(fin, getString);

                        simProcessDataMiddleHold [6] = atof(getString.c_str());

                        getline(fin, getString);

                        simProcessDataMiddleHold [7] = atof(getString.c_str());

                        getline(fin, getString);

                        simProcessDataMiddleHold [8] = atof(getString.c_str());

                        getline(fin, getString);

                        simProcessDataMiddleHold [9] = atof(getString.c_str());

                        getline(fin, getString);

                        simProcessDataMiddleHold [10] = atof(getString.c_str());

                        getline(fin, getString);

                        simProcessDataMiddleHold [11] = atof(getString.c_str());

                        getline(fin, getString);

                        simProcessDataMiddleHold [12] = atof(getString.c_str());

                        getline(fin, getString);

                        simProcessDataMiddleHold [13] = atof(getString.c_str());

                        getline(fin, getString);

                        simProcessDataMiddleHold [14] = atof(getString.c_str());

                        getline(fin, getString);

                        simProcessDataMiddleHold [15] = atof(getString.c_str());

                        getline(fin, getString);

                        simProcessDataMiddleHold [16] = atof(getString.c_str());

                        getline(fin, getString);

                        simProcessDataMiddleHold [17] = atof(getString.c_str());

                        getline(fin, getString);

                        simProcessDataMiddleHold [18] = atof(getString.c_str());

                        getline(fin, getString);

                        simProcessDataMiddleHold [19] = atof(getString.c_str());

                        getline(fin, getString);

                        simProcessDataMiddleHold [20] = atof(getString.c_str());

                        getline(fin, getString);

                        simProcessDataMiddleHold [21] = atof(getString.c_str());

                        getline(fin, getString);

                        simProcessDataMiddleHold [22] = atof(getString.c_str());

                        getline(fin, getString);

                        simProcessDataMiddleHold [23] = atof(getString.c_str());

                        getline(fin, getString);

                        simProcessDataMiddleHold [24] = atof(getString.c_str());

                        getline(fin, getString);

                        simProcessDataMiddleHold [25] = atof(getString.c_str());

                        getline(fin, getString);

                        simProcessDataMiddleHold [26] = atof(getString.c_str());

                        getline(fin, getString);

                        simProcessDataMiddleHold [27] = atof(getString.c_str());

                        [growthMidTimeBDDisplay setDoubleValue:simProcessDataMiddleHold [0]];

                        [growthMidTimeTDDisplay setDoubleValue:simProcessDataMiddleHold [1]];

                        [growthMidTimeCFDisplay setDoubleValue:simProcessDataMiddleHold [2]];

**int** dataBD = (**int**)simProcessDataMiddleHold [3];

**int** dataTD = (**int**)simProcessDataMiddleHold [4];

**int** dataBDCFBD = (**int**)simProcessDataMiddleHold [8];

**int** dataBDCFTD = (**int**)simProcessDataMiddleHold [9];

**int** dataTDCFBD = (**int**)simProcessDataMiddleHold [12];

**int** dataTDCFTD = (**int**)simProcessDataMiddleHold [13];

**int** dataTDBD = (**int**)simProcessDataMiddleHold [15];

**int** dataTDTD = (**int**)simProcessDataMiddleHold [16];

**int** nonDivLing = (**int**)simProcessDataMiddleHold [20];

**int** recoveryGR = (**int**)simProcessDataMiddleHold [21];

**int** totalNumberOfDiv = (**int**)simProcessDataMiddleHold [23];

**int** numberOfLing = (**int**)simProcessDataMiddleHold [24];

**int** timePointMax = (**int**)simProcessDataMiddleHold [25];

**double** percentTemp = dataBD/(**double**)totalNumberOfDiv;

**int** percentInt = (**int**)(percentTemp*10000);

                        percentTemp = percentInt/(**double**)100;

                        [growthMidBDDisplay setDoubleValue:percentTemp];

                        percentTemp = dataTD/(**double**)totalNumberOfDiv;

                        percentInt = (**int**)(percentTemp*10000);

                        percentTemp = percentInt/(**double**)100;

                        [growthMidTDDisplay setDoubleValue:percentTemp];

                        [growthMidBDCDDisplay setDoubleValue:simProcessDataMiddleHold [5]];

                        [growthMidNOCDDisplay setDoubleValue:simProcessDataMiddleHold [6]];

                        [growthMidBDCFDisplay setDoubleValue:simProcessDataMiddleHold [7]];

                        percentTemp = dataBDCFBD/(**double**)totalNumberOfDiv;

                        percentInt = (**int**)(percentTemp*10000);

                        percentTemp = percentInt/(**double**)100;

                        [growthMidBDCFBDDisplay setDoubleValue:percentTemp];

                        percentTemp = dataBDCFTD/(**double**)totalNumberOfDiv;

                        percentInt = (**int**)(percentTemp*10000);

                        percentTemp = percentInt/(**double**)100;

                        [growthMidBDCFTDDisplay setDoubleValue:percentTemp];

                        [growthMidBDCFCDDisplay setDoubleValue:simProcessDataMiddleHold [10]];

                        [growthMidTDCFDisplay setDoubleValue:simProcessDataMiddleHold [11]];

                        percentTemp = dataTDCFBD/(**double**)totalNumberOfDiv;

                        percentInt = (**int**)(percentTemp*10000);

                        percentTemp = percentInt/(**double**)100;

                        [growthMidTDCFBDDisplay setDoubleValue:percentTemp];

                        percentTemp = dataTDCFTD/(**double**)totalNumberOfDiv;

                        percentInt = (**int**)(percentTemp*10000);

                        percentTemp = percentInt/(**double**)100;

                        [growthMidTDCFTDDisplay setDoubleValue:percentTemp];

                        [growthMidTDCFCDDisplay setDoubleValue:simProcessDataMiddleHold [14]];

                        percentTemp = dataTDBD/(**double**)totalNumberOfDiv;

                        percentInt = (**int**)(percentTemp*10000);

                        percentTemp = percentInt/(**double**)100;

                        [growthMidTDBDDisplay setDoubleValue:percentTemp];

                        percentTemp = dataTDTD/(**double**)totalNumberOfDiv;

                        percentInt = (**int**)(percentTemp*10000);

                        percentTemp = percentInt/(**double**)100;

                        [growthMidTDTDDisplay setDoubleValue:percentTemp];

                        [growthMidTDCDDisplay setDoubleValue:simProcessDataMiddleHold [17]];

                        [growthMidMulTDTDDisplay setDoubleValue:simProcessDataMiddleHold [18]];

                        [growthMidSupTDBDDisplay setDoubleValue:simProcessDataMiddleHold [19]];

                        percentTemp = nonDivLing/(**double**)numberOfLing;

                        percentInt = (**int**)(percentTemp*10000);

                        percentTemp = percentInt/(**double**)100;

                        [growthMidNoDivDisplay setDoubleValue:percentTemp];

                        percentTemp = recoveryGR/(**double**)numberOfLing;

                        percentInt = (**int**)(percentTemp*10000);

                        percentTemp = percentInt/(**double**)100;

                        [growthMidRecovDisplay setDoubleValue:percentTemp];

**delete** [] simulationDistributionMidData;

                        simulationDistributionMidData = **new** **int** [timePointMax*11+100];

                        simulationDistributionMidDataCount = timePointMax*11;

**int** entryCount = 0;

                        getline(fin, getString);

**if** (getString == "A1"){

**do**{

                                terminationFlag = 1;

                                getline(fin, getString);

**if** (getString != "End"){

                                    simulationDistributionMidData [entryCount*11] = atoi(getString.c_str()), entryCount++;

                                }

**else** terminationFlag = 0;

                            } **while** (terminationFlag == 1);

                        }

                        getline(fin, getString);

                        entryCount = 0;

**if** (getString == "A2"){

**do**{

                                terminationFlag = 1;

                                getline(fin, getString);

**if** (getString != "End"){

                                    simulationDistributionMidData [entryCount*11+1] = atoi(getString.c_str()), entryCount++;

                                }

**else** terminationFlag = 0;

                            } **while** (terminationFlag == 1);

                        }

                        getline(fin, getString);

                        entryCount = 0;

**if** (getString == "A3"){

**do**{

                                terminationFlag = 1;

                                getline(fin, getString);

**if** (getString != "End"){

                                    simulationDistributionMidData [entryCount*11+2] = atoi(getString.c_str()), entryCount++;

                                }

**else** terminationFlag = 0;

                            } **while** (terminationFlag == 1);

                        }

                        getline(fin, getString);

                        entryCount = 0;

**if** (getString == "A4"){

**do**{

                                terminationFlag = 1;

                                getline(fin, getString);

**if** (getString != "End"){

                                    simulationDistributionMidData [entryCount*11+3] = atoi(getString.c_str()), entryCount++;

                                }

**else** terminationFlag = 0;

                            } **while** (terminationFlag == 1);

                        }

                        getline(fin, getString);

                        entryCount = 0;

**if** (getString == "A5"){

**do**{

                                terminationFlag = 1;

                                getline(fin, getString);

**if** (getString != "End"){

                                    simulationDistributionMidData [entryCount*11+4] = atoi(getString.c_str()), entryCount++;

                                }

**else** terminationFlag = 0;

                            } **while** (terminationFlag == 1);

                        }

                        getline(fin, getString);

                        entryCount = 0;

**if** (getString == "A6"){

**do**{

                                terminationFlag = 1;

                                getline(fin, getString);

**if** (getString != "End"){

                                    simulationDistributionMidData [entryCount*11+5] = atoi(getString.c_str()), entryCount++;

                                }

**else** terminationFlag = 0;

                            } **while** (terminationFlag == 1);

                        }

                        getline(fin, getString);

                        entryCount = 0;

**if** (getString == "A7"){

**do**{

                                terminationFlag = 1;

                                getline(fin, getString);

**if** (getString != "End"){

                                    simulationDistributionMidData [entryCount*11+6] = atoi(getString.c_str()), entryCount++;

                                }

**else** terminationFlag = 0;

                            } **while** (terminationFlag == 1);

                        }

                        getline(fin, getString);

                        entryCount = 0;

**if** (getString == "A8"){

**do**{

                                terminationFlag = 1;

                                getline(fin, getString);

**if** (getString != "End"){

                                    simulationDistributionMidData [entryCount*11+7] = atoi(getString.c_str()), entryCount++;

                                }

**else** terminationFlag = 0;

                            } **while** (terminationFlag == 1);

                        }

                        getline(fin, getString);

                        entryCount = 0;

**if** (getString == "A9"){

**do**{

                                terminationFlag = 1;

                                getline(fin, getString);

**if** (getString != "End"){

                                    simulationDistributionMidData [entryCount*11+8] = atoi(getString.c_str()), entryCount++;

                                }

**else** terminationFlag = 0;

                            } **while** (terminationFlag == 1);

                        }

                        getline(fin, getString);

                        entryCount = 0;

**if** (getString == "A10"){

**do**{

                                terminationFlag = 1;

                                getline(fin, getString);

**if** (getString != "End"){

                                    simulationDistributionMidData [entryCount*11+9] = atoi(getString.c_str()), entryCount++;

                                }

**else** terminationFlag = 0;

                            } **while** (terminationFlag == 1);

                        }

                        getline(fin, getString);

                        entryCount = 0;

**if** (getString == "A11"){

**do**{

                                terminationFlag = 1;

                                getline(fin, getString);

**if** (getString != "End"){

                                    simulationDistributionMidData [entryCount*11+10] = atoi(getString.c_str()), entryCount++;

                                }

**else** terminationFlag = 0;

                            } **while** (terminationFlag == 1);

                        }

                        NSSound *sound = [NSSound soundNamed:@"Ping"];

                        [sound play];

                    }

**else**{

                        NSAlert *alert = [[NSAlert alloc] init];

                        [alert addButtonWithTitle:@"OK"];

                        [alert setMessageText:@"No Sim database file"];

                        [alert setAlertStyle:NSAlertStyleWarning];

                        [alert runModal];

                        [alert release];

                        NSSound *sound = [NSSound soundNamed:@"Hero"];

                        [sound play];

                    }

                    fin.close();

                }

            }

        }

    }

**else**{

        NSAlert *alert = [[NSAlert alloc] init];

        [alert addButtonWithTitle:@"OK"];

        [alert setMessageText:@"Sim is in progress"];

        [alert setAlertStyle:NSAlertStyleWarning];

        [alert runModal];

        [alert release];

        NSSound *sound = [NSSound soundNamed:@"Hero"];

        [sound play];

    }

}

-(**IBAction**)getListGrowthAddSet:(**id**)sender{

**if** (simulationprogress == 0){

**if** (databaseLoadOptionHold == 0){

**if** (simListHoldCount != 0){

                messageStringSim4 = arraySimListHold [simOperationTableCurrentRow*2+1].substr(arraySimListHold [simOperationTableCurrentRow*2+1].find("-")+1);

                NSString *treatPickUpNameNSString = @(messageStringSim4.c_str());

                [growthAddTreatDisplay setStringValue: treatPickUpNameNSString];

**int** *arrayCellLineageTemp = **new** **int** [(arrayLineageDataEntryHold [simOperationTableCurrentRow]/9)*9+100];

**int** cellLineageTempCount = 0;

**for** (**unsigned** **long** counter3 = 0; counter3 < (arrayLineageDataEntryHold [simOperationTableCurrentRow]/9)*9+100; counter3++){

                    arrayCellLineageTemp [counter3] = 0;

                }

**for** (**unsigned** **long** counter3 = 0; counter3 < arrayLineageDataEntryHold [simOperationTableCurrentRow]/9; counter3++){

                    arrayCellLineageTemp [cellLineageTempCount] = arrayLineageData [simOperationTableCurrentRow][counter3*9], cellLineageTempCount++;

                    arrayCellLineageTemp [cellLineageTempCount] = arrayLineageData [simOperationTableCurrentRow][counter3*9+1], cellLineageTempCount++;

                    arrayCellLineageTemp [cellLineageTempCount] = arrayLineageData [simOperationTableCurrentRow][counter3*9+2], cellLineageTempCount++;

                    arrayCellLineageTemp [cellLineageTempCount] = arrayLineageData [simOperationTableCurrentRow][counter3*9+3], cellLineageTempCount++;

                    arrayCellLineageTemp [cellLineageTempCount] = arrayLineageData [simOperationTableCurrentRow][counter3*9+4], cellLineageTempCount++;

                    arrayCellLineageTemp [cellLineageTempCount] = arrayLineageData [simOperationTableCurrentRow][counter3*9+5], cellLineageTempCount++;

                    arrayCellLineageTemp [cellLineageTempCount] = arrayLineageData [simOperationTableCurrentRow][counter3*9+6], cellLineageTempCount++;

                    arrayCellLineageTemp [cellLineageTempCount] = arrayLineageData [simOperationTableCurrentRow][counter3*9+7], cellLineageTempCount++;

                    arrayCellLineageTemp [cellLineageTempCount] = arrayLineageData [simOperationTableCurrentRow][counter3*9+8], cellLineageTempCount++;

                }

**for** (**int** counter3 = 0; counter3 < cellLineageTempCount/9; counter3++){

**if** (arrayCellLineageTemp [counter3*9+3] == 92){

**for** (**int** counter4 = counter3-1; counter4 >= 0; counter4--){

**if** (arrayCellLineageTemp [counter4*9+3] == 92){

                                arrayCellLineageTemp [counter4*9+3] = 2;

                            }

**else** **if** (arrayCellLineageTemp [counter4*9+3] == 1 || arrayCellLineageTemp [counter4*9+3] == 31 || arrayCellLineageTemp [counter4*9+3] == 41 || arrayCellLineageTemp [counter4*9+3] == 51){

**break**;

                            }

                        }

                    }

                }

**int** timePointMax = arrayTableDetail [simOperationTableCurrentRow][3];

**int** twentyPercentOfMax = timePointMax-(**int**)(round(timePointMax*(**double**)(recoveryCalcPercentHold/(**double**)100)));

**int** *dataCDOccurence = **new** **int** [timePointMax+10];

**int** *dataNonDivCDOccurence = **new** **int** [timePointMax+10];

**int** *dataBDCFOccurence = **new** **int** [timePointMax+10];

**int** *dataBDCFCDOccurence = **new** **int** [timePointMax+10];

**int** *dataTDCFOccurence = **new** **int** [timePointMax+10];

**int** *dataTDCFCDOccurence = **new** **int** [timePointMax+10];

**int** *dataTDCDOccurence = **new** **int** [timePointMax+10];

**int** *dataVariationToTheFirstDV = **new** **int** [timePointMax+10];

**for** (**int** counter3 = 0; counter3 < timePointMax+10; counter3++){

                    dataCDOccurence [counter3] = 0;

                    dataNonDivCDOccurence [counter3] = 0;

                    dataBDCFOccurence [counter3] = 0;

                    dataBDCFCDOccurence [counter3] = 0;

                    dataTDCFOccurence [counter3] = 0;

                    dataTDCFCDOccurence [counter3] = 0;

                    dataTDCDOccurence [counter3] = 0;

                    dataVariationToTheFirstDV [counter3] = 0;

                }

**int** dataBD = 0;

**int** dataTD = 0;

**int** dataBDCFBD = 0;

**int** dataBDCFTD = 0;

**int** dataTDCFBD = 0;

**int** dataTDCFTD = 0;

**int** dataTDBD = 0;

**int** dataTDTD = 0;

**int** nonDivLing = 0;

**int** recoveryGR = 0;

**int** dataCD = 0;

**double** dataBDRand = 0;

**double** dataCDRand = 0;

**for** (**int** counter3 = 0; counter3 < cellLineageTempCount/9; counter3++){

**if** (arrayCellLineageTemp [counter3*9+3] == 32){

                        dataBD++;

                        dataBDRand = dataBDRand+arrayCellLineageTemp [counter3*9+2];

                    }

**if** (arrayCellLineageTemp [counter3*9+3] == 42 || arrayCellLineageTemp [counter3*9+3] == 52) dataTD++;

**if** (arrayCellLineageTemp [counter3*9+3] == 7){

**for** (**int** counter4 = counter3; counter4 >= 0; counter4--){

**if** (arrayCellLineageTemp [counter4*9+3] == 31){

                                dataCDOccurence [counter3-counter4]++;

**break**;

                            }

**else** **if** (arrayCellLineageTemp [counter4*9+3] == 1 || arrayCellLineageTemp [counter4*9+3] == 41 || arrayCellLineageTemp [counter4*9+3] == 51 || arrayCellLineageTemp [counter4*9+3] == 92){

**break**;

                            }

                        }

                        dataCD++;

                        dataCDRand = dataCDRand+arrayCellLineageTemp [counter3*9+2];

                    }

**if** (arrayCellLineageTemp [counter3*9+3] == 7){

**for** (**int** counter4 = counter3; counter4 >= 0; counter4--){

**if** (arrayCellLineageTemp [counter4*9+3] == 1){

                                dataNonDivCDOccurence [counter3-counter4]++;

**break**;

                            }

**else** **if** (arrayCellLineageTemp [counter4*9+3] == 31 || arrayCellLineageTemp [counter4*9+3] == 41 || arrayCellLineageTemp [counter4*9+3] == 51 || arrayCellLineageTemp [counter4*9+3] == 92){

**break**;

                            }

                        }

                    }

**if** (arrayCellLineageTemp [counter3*9+3] == 91){

**for** (**int** counter4 = counter3; counter4 >= 0; counter4--){

**if** (arrayCellLineageTemp [counter4*9+3] == 31){

                                dataBDCFOccurence [counter3-counter4]++;

**break**;

                            }

**else** **if** (arrayCellLineageTemp [counter4*9+3] == 1 || arrayCellLineageTemp [counter4*9+3] == 41 || arrayCellLineageTemp [counter4*9+3] == 51 || arrayCellLineageTemp [counter4*9+3] == 92){

**break**;

                            }

                        }

                    }

**if** (arrayCellLineageTemp [counter3*9+3] == 92){

**for** (**int** counter4 = counter3; counter4 < cellLineageTempCount/9; counter4++){

**if** (arrayCellLineageTemp [counter4*9+3] == 32){

**for** (**int** counter5 = counter3; counter5 >= 0; counter5--){

**if** (arrayCellLineageTemp [counter5*9+3] == 31){

                                        dataBDCFBD++;

**break**;

                                    }

**else** **if** (arrayCellLineageTemp [counter5*9+3] == 1 || arrayCellLineageTemp [counter5*9+3] == 41 || arrayCellLineageTemp [counter5*9+3] == 51){

**break**;

                                    }

                                }

**break**;

                            }

**else** **if** ((arrayCellLineageTemp [counter3*9+5] != arrayCellLineageTemp [counter4*9+5] || arrayCellLineageTemp [counter3*9+6] != arrayCellLineageTemp [counter4*9+6]) || arrayCellLineageTemp [counter4*9+3] == 8 || arrayCellLineageTemp [counter4*9+3] == 42 || arrayCellLineageTemp [counter4*9+3] == 52 || arrayCellLineageTemp [counter4*9+3] == 91 || arrayCellLineageTemp [counter4*9+2] == timePointMax){

**break**;

                            }

                        }

                    }

**if** (arrayCellLineageTemp [counter3*9+3] == 92){

**for** (**int** counter4 = counter3; counter4 < cellLineageTempCount/9; counter4++){

**if** (arrayCellLineageTemp [counter4*9+3] == 42 || arrayCellLineageTemp [counter4*9+3] == 52){

**for** (**int** counter5 = counter3; counter5 >= 0; counter5--){

**if** (arrayCellLineageTemp [counter5*9+3] == 31){

                                        dataBDCFTD++;

**break**;

                                    }

**else** **if** (arrayCellLineageTemp [counter5*9+3] == 1 || arrayCellLineageTemp [counter5*9+3] == 41 || arrayCellLineageTemp [counter5*9+3] == 51){

**break**;

                                    }

                                }

**break**;

                            }

**else** **if** ((arrayCellLineageTemp [counter3*9+5] != arrayCellLineageTemp [counter4*9+5] || arrayCellLineageTemp [counter3*9+6] != arrayCellLineageTemp [counter4*9+6]) || arrayCellLineageTemp [counter4*9+3] == 8 || arrayCellLineageTemp [counter4*9+3] == 32 || arrayCellLineageTemp [counter4*9+3] == 91 || arrayCellLineageTemp [counter4*9+2] == timePointMax){

**break**;

                            }

                        }

                    }

**if** (arrayCellLineageTemp [counter3*9+3] == 92){

**for** (**int** counter4 = counter3; counter4 < cellLineageTempCount/9; counter4++){

**if** (arrayCellLineageTemp [counter4*9+3] == 7){

**for** (**int** counter5 = counter3; counter5 >= 0; counter5--){

**if** (arrayCellLineageTemp [counter5*9+3] == 31){

                                        dataBDCFCDOccurence [counter4-counter3]++;

**break**;

                                    }

**else** **if** (arrayCellLineageTemp [counter5*9+3] == 1 || arrayCellLineageTemp [counter5*9+3] == 41 || arrayCellLineageTemp [counter5*9+3] == 51){

**break**;

                                    }

                                }

**break**;

                            }

**else** **if** ((arrayCellLineageTemp [counter3*9+5] != arrayCellLineageTemp [counter4*9+5] || arrayCellLineageTemp [counter3*9+6] != arrayCellLineageTemp [counter4*9+6]) || arrayCellLineageTemp [counter4*9+3] == 8 || arrayCellLineageTemp [counter4*9+3] == 32 || arrayCellLineageTemp [counter4*9+3] == 42 || arrayCellLineageTemp [counter4*9+3] == 52 || arrayCellLineageTemp [counter4*9+3] == 91 || arrayCellLineageTemp [counter4*9+2] == timePointMax){

**break**;

                            }

                        }

                    }

**if** (arrayCellLineageTemp [counter3*9+3] == 91){

**for** (**int** counter4 = counter3; counter4 >= 0; counter4--){

**if** (arrayCellLineageTemp [counter4*9+3] == 41 || arrayCellLineageTemp [counter4*9+3] == 51){

                                dataTDCFOccurence [counter3-counter4]++;

**break**;

                            }

**else** **if** (arrayCellLineageTemp [counter4*9+3] == 1 || arrayCellLineageTemp [counter4*9+3] == 31 || arrayCellLineageTemp [counter4*9+3] == 92){

**break**;

                            }

                        }

                    }

**if** (arrayCellLineageTemp [counter3*9+3] == 92){

**for** (**int** counter4 = counter3; counter4 < cellLineageTempCount/9; counter4++){

**if** (arrayCellLineageTemp [counter4*9+3] == 32){

**for** (**int** counter5 = counter3; counter5 >= 0; counter5--){

**if** (arrayCellLineageTemp [counter5*9+3] == 41 || arrayCellLineageTemp [counter5*9+3] == 51){

                                        dataTDCFBD++;

**break**;

                                    }

**else** **if** (arrayCellLineageTemp [counter5*9+3] == 1 || arrayCellLineageTemp [counter5*9+3] == 31){

**break**;

                                    }

                                }

**break**;

                            }

**else** **if** ((arrayCellLineageTemp [counter3*9+5] != arrayCellLineageTemp [counter4*9+5] || arrayCellLineageTemp [counter3*9+6] != arrayCellLineageTemp [counter4*9+6]) || arrayCellLineageTemp [counter4*9+3] == 8 || arrayCellLineageTemp [counter4*9+3] == 42 || arrayCellLineageTemp [counter4*9+3] == 52 || arrayCellLineageTemp [counter4*9+3] == 91 || arrayCellLineageTemp [counter4*9+2] == timePointMax){

**break**;

                            }

                        }

                    }

**if** (arrayCellLineageTemp [counter3*9+3] == 92){

**for** (**int** counter4 = counter3; counter4 < cellLineageTempCount/9; counter4++){

**if** (arrayCellLineageTemp [counter4*9+3] == 42 || arrayCellLineageTemp [counter4*9+3] == 52){

**for** (**int** counter5 = counter3; counter5 >= 0; counter5--){

**if** (arrayCellLineageTemp [counter5*9+3] == 41 || arrayCellLineageTemp [counter5*9+3] == 51){

                                        dataTDCFTD++;

**break**;

                                    }

**else** **if** (arrayCellLineageTemp [counter5*9+3] == 1 || arrayCellLineageTemp [counter5*9+3] == 31){

**break**;

                                    }

                                }

**break**;

                            }

**else** **if** ((arrayCellLineageTemp [counter3*9+5] != arrayCellLineageTemp [counter4*9+5] || arrayCellLineageTemp [counter3*9+6] != arrayCellLineageTemp [counter4*9+6]) || arrayCellLineageTemp [counter4*9+3] == 8 || arrayCellLineageTemp [counter4*9+3] == 32 || arrayCellLineageTemp [counter4*9+3] == 91 || arrayCellLineageTemp [counter4*9+2] == timePointMax){

**break**;

                            }

                        }

                    }

**if** (arrayCellLineageTemp [counter3*9+3] == 92){

**for** (**int** counter4 = counter3; counter4 < cellLineageTempCount/9; counter4++){

**if** (arrayCellLineageTemp [counter4*9+3] == 7){

**for** (**int** counter5 = counter3; counter5 >= 0; counter5--){

**if** (arrayCellLineageTemp [counter5*9+3] == 41 || arrayCellLineageTemp [counter5*9+3] == 51){

                                        dataTDCFCDOccurence [counter4-counter3]++;

**break**;

                                    }

**else** **if** (arrayCellLineageTemp [counter5*9+3] == 1 || arrayCellLineageTemp [counter5*9+3] == 31){

**break**;

                                    }

                                }

**break**;

                            }

**else** **if** ((arrayCellLineageTemp [counter3*9+5] != arrayCellLineageTemp [counter4*9+5] || arrayCellLineageTemp [counter3*9+6] != arrayCellLineageTemp [counter4*9+6]) || arrayCellLineageTemp [counter4*9+3] == 8 || arrayCellLineageTemp [counter4*9+3] == 32 || arrayCellLineageTemp [counter4*9+3] == 42 || arrayCellLineageTemp [counter4*9+3] == 52 || arrayCellLineageTemp [counter4*9+3] == 91 || arrayCellLineageTemp [counter4*9+2] == timePointMax){

**break**;

                            }

                        }

                    }

**if** (arrayCellLineageTemp [counter3*9+3] == 41 || arrayCellLineageTemp [counter3*9+3] == 51){

**for** (**int** counter4 = counter3; counter4 < cellLineageTempCount/9; counter4++){

**if** (arrayCellLineageTemp [counter4*9+3] == 32){

                                dataTDBD++;

**break**;

                            }

**else** **if** ((arrayCellLineageTemp [counter3*9+5] != arrayCellLineageTemp [counter4*9+5] || arrayCellLineageTemp [counter3*9+6] != arrayCellLineageTemp [counter4*9+6]) || arrayCellLineageTemp [counter4*9+3] == 8 || arrayCellLineageTemp [counter4*9+3] == 7 || arrayCellLineageTemp [counter4*9+3] == 42 || arrayCellLineageTemp [counter4*9+3] == 52 || arrayCellLineageTemp [counter4*9+3] == 91 || arrayCellLineageTemp [counter4*9+3] == 92 || arrayCellLineageTemp [counter4*9+2] == timePointMax){

**break**;

                            }

                        }

                    }

**if** (arrayCellLineageTemp [counter3*9+3] == 41 || arrayCellLineageTemp [counter3*9+3] == 51){

**for** (**int** counter4 = counter3; counter4 < cellLineageTempCount/9; counter4++){

**if** (arrayCellLineageTemp [counter4*9+3] == 42 || arrayCellLineageTemp [counter4*9+3] == 52){

                                dataTDTD++;

**break**;

                            }

**else** **if** ((arrayCellLineageTemp [counter3*9+5] != arrayCellLineageTemp [counter4*9+5] || arrayCellLineageTemp [counter3*9+6] != arrayCellLineageTemp [counter4*9+6]) || arrayCellLineageTemp [counter4*9+3] == 8 || arrayCellLineageTemp [counter4*9+3] == 7 || arrayCellLineageTemp [counter4*9+3] == 32 || arrayCellLineageTemp [counter4*9+3] == 91 || arrayCellLineageTemp [counter4*9+3] == 92 || arrayCellLineageTemp [counter4*9+2] == timePointMax){

**break**;

                            }

                        }

                    }

**if** (arrayCellLineageTemp [counter3*9+3] == 7){

**for** (**int** counter4 = counter3; counter4 >= 0; counter4--){

**if** (arrayCellLineageTemp [counter4*9+3] == 41 || arrayCellLineageTemp [counter4*9+3] == 51){

                                dataTDCDOccurence [counter3-counter4]++;

**break**;

                            }

**else** **if** (arrayCellLineageTemp [counter4*9+3] == 1 || arrayCellLineageTemp [counter4*9+3] == 31 || arrayCellLineageTemp [counter4*9+3] == 92){

**break**;

                            }

                        }

                    }

**if** (arrayCellLineageTemp [counter3*9+3] == 1){

**for** (**int** counter4 = counter3; counter4 < cellLineageTempCount/9; counter4++){

**if** (arrayCellLineageTemp [counter4*9+2] == timePointMax){

                                nonDivLing++;

**break**;

                            }

**else** **if** ((arrayCellLineageTemp [counter3*9+5] != arrayCellLineageTemp [counter4*9+5] || arrayCellLineageTemp [counter3*9+6] != arrayCellLineageTemp [counter4*9+6]) || arrayCellLineageTemp [counter4*9+3] == 8 || arrayCellLineageTemp [counter4*9+3] == 7 || arrayCellLineageTemp [counter4*9+3] == 32 || arrayCellLineageTemp [counter4*9+3] == 42 || arrayCellLineageTemp [counter4*9+3] == 52 || arrayCellLineageTemp [counter4*9+3] == 91 || arrayCellLineageTemp [counter4*9+2] == timePointMax){

**break**;

                            }

                        }

                    }

**if** (arrayCellLineageTemp [counter3*9+2] > twentyPercentOfMax && arrayCellLineageTemp [counter3*9+3] == 32){

**for** (**int** counter4 = counter3; counter4 >= 0; counter4--){

**if** (arrayCellLineageTemp [counter4*9+3] == 1){

                                recoveryGR++;

**break**;

                            }

**else** **if** (arrayCellLineageTemp [counter4*9+3] == 31 || arrayCellLineageTemp [counter4*9+3] == 41 || arrayCellLineageTemp [counter4*9+3] == 51 || arrayCellLineageTemp [counter4*9+3] == 92){

**break**;

                            }

                        }

                    }

**if** (arrayCellLineageTemp [counter3*9+3] == 1){

**for** (**int** counter4 = counter3; counter4 < cellLineageTempCount/9; counter4++){

**if** (arrayCellLineageTemp [counter4*9+3] == 7 || arrayCellLineageTemp [counter4*9+3] == 32 || arrayCellLineageTemp [counter4*9+3] == 42 || arrayCellLineageTemp [counter4*9+3] == 52 || arrayCellLineageTemp [counter4*9+3] == 91 || arrayCellLineageTemp [counter4*9+2] == timePointMax){

                                dataVariationToTheFirstDV [counter4-counter3]++;

**break**;

                            }

**else** **if** ((arrayCellLineageTemp [counter3*9+5] != arrayCellLineageTemp [counter4*9+5] || arrayCellLineageTemp [counter3*9+6] != arrayCellLineageTemp [counter4*9+6]) || arrayCellLineageTemp [counter4*9+3] == 8){

**break**;

                            }

                        }

                    }

                }

**if** (dataBD != 0) dataBDRand = dataBDRand/(**double**)dataBD;

**if** (dataCD != 0) dataCDRand = dataCDRand/(**double**)dataCD;

**int** *dataDoublingTimeBD = **new** **int** [timePointMax+50];

**int** *dataDoublingTimeTD = **new** **int** [timePointMax+50];

**int** *dataDoublingTimeCF = **new** **int** [timePointMax+50];

**for** (**int** counter3 = 0; counter3 < timePointMax+10; counter3++){

                    dataDoublingTimeBD [counter3] = 0;

                    dataDoublingTimeTD [counter3] = 0;

                    dataDoublingTimeCF [counter3] = 0;

                }

**int** endTime = 0;

**int** startTime = 0;

**for** (**int** counter3 = 0; counter3 < cellLineageTempCount/9; counter3++){

**if** (arrayCellLineageTemp [counter3*9+3] == 31){

                        endTime = 0;

**for** (**int** counter4 = counter3+1; counter4 < cellLineageTempCount/9; counter4++){

**if** (arrayCellLineageTemp [counter3*9+5] == arrayCellLineageTemp [counter4*9+5] && arrayCellLineageTemp [counter3*9+6] == arrayCellLineageTemp [counter4*9+6] && (arrayCellLineageTemp [counter4*9+3] == 32 || arrayCellLineageTemp [counter4*9+3] == 42 || arrayCellLineageTemp [counter4*9+3] == 52)){

                                endTime = arrayCellLineageTemp [counter4*9+2];

                            }

**else** **if** (arrayCellLineageTemp [counter3*9+5] != arrayCellLineageTemp [counter4*9+5] || arrayCellLineageTemp [counter3*9+6] != arrayCellLineageTemp [counter4*9+6]){

**break**;

                            }

                        }

**if** (endTime != 0){

                            dataDoublingTimeBD [endTime-arrayCellLineageTemp [counter3*9+2]]++;

                        }

                    }

**if** (arrayCellLineageTemp [counter3*9+3] == 41 || arrayCellLineageTemp [counter3*9+3] == 51){

                        endTime = 0;

**for** (**int** counter4 = counter3+1; counter4 < cellLineageTempCount/9; counter4++){

**if** (arrayCellLineageTemp [counter3*9+5] == arrayCellLineageTemp [counter4*9+5] && arrayCellLineageTemp [counter3*9+6] == arrayCellLineageTemp [counter4*9+6] && (arrayCellLineageTemp [counter4*9+3] == 32 || arrayCellLineageTemp [counter4*9+3] == 42 || arrayCellLineageTemp [counter4*9+3] == 52)){

                                endTime = arrayCellLineageTemp [counter4*9+2];

                            }

**else** **if** (arrayCellLineageTemp [counter3*9+5] != arrayCellLineageTemp [counter4*9+5] || arrayCellLineageTemp [counter3*9+6] != arrayCellLineageTemp [counter4*9+6]){

**break**;

                            }

                        }

**if** (endTime != 0){

                            dataDoublingTimeTD [endTime-arrayCellLineageTemp [counter3*9+2]]++;

                        }

                    }

**if** (arrayCellLineageTemp [counter3*9+3] == 92){

                        startTime = 0;

**for** (**int** counter4 = counter3; counter4 >= 0; counter4--){

**if** (arrayCellLineageTemp [counter4*9+3] == 31 || arrayCellLineageTemp [counter4*9+3] == 41 || arrayCellLineageTemp [counter4*9+3] == 51){

                                startTime = arrayCellLineageTemp [counter4*9+2];

**break**;

                            }

**else** **if** ((arrayCellLineageTemp [counter3*9+5] != arrayCellLineageTemp [counter4*9+5] || arrayCellLineageTemp [counter3*9+6] != arrayCellLineageTemp [counter4*9+6]) || arrayCellLineageTemp [counter4*9+3] == 1){

**break**;

                            }

                        }

                        endTime = 0;

**for** (**int** counter4 = counter3+1; counter4 < cellLineageTempCount/9; counter4++){

**if** (arrayCellLineageTemp [counter3*9+5] == arrayCellLineageTemp [counter4*9+5] && arrayCellLineageTemp [counter3*9+6] == arrayCellLineageTemp [counter4*9+6] && (arrayCellLineageTemp [counter4*9+3] == 32 || arrayCellLineageTemp [counter4*9+3] == 42 || arrayCellLineageTemp [counter4*9+3] == 52)){

                                endTime = arrayCellLineageTemp [counter4*9+2];

                            }

**else** **if** (arrayCellLineageTemp [counter3*9+5] != arrayCellLineageTemp [counter4*9+5] || arrayCellLineageTemp [counter3*9+6] != arrayCellLineageTemp [counter4*9+6]){

**break**;

                            }

                        }

**if** (startTime != 0 && endTime != 0){

                            dataDoublingTimeCF [endTime-startTime]++;

                        }

                    }

                }

**int** numberOfLing = arrayTableDetail [simOperationTableCurrentRow][1];

**int** totalNumberOfDiv = arrayTableDetail [simOperationTableCurrentRow][13];

                simProcessDataAddHold [0] = 1;

                simProcessDataAddHold [1] = 1;

                simProcessDataAddHold [2] = 1;

                simProcessDataAddHold [3] = dataBD;

                simProcessDataAddHold [4] = dataTD;

                simProcessDataAddHold [5] = 1;

                simProcessDataAddHold [6] = 1;

                simProcessDataAddHold [7] = 1;

                simProcessDataAddHold [8] = dataBDCFBD;

                simProcessDataAddHold [9] = dataBDCFTD;

                simProcessDataAddHold [10] = 1;

                simProcessDataAddHold [11] = 1;

                simProcessDataAddHold [12] = dataTDCFBD;

                simProcessDataAddHold [13] = dataTDCFTD;

                simProcessDataAddHold [14] = 1;

                simProcessDataAddHold [15] = dataTDBD;

                simProcessDataAddHold [16] = dataTDTD;

                simProcessDataAddHold [17] = 1;

                simProcessDataAddHold [18] = 2;

                simProcessDataAddHold [19] = 3;

                simProcessDataAddHold [20] = nonDivLing;

                simProcessDataAddHold [21] = recoveryGR;

                simProcessDataAddHold [22] = recoveryCalcPercentHold;

                simProcessDataAddHold [23] = totalNumberOfDiv;

                simProcessDataAddHold [24] = numberOfLing;

                simProcessDataAddHold [25] = timePointMax;

                simProcessDataAddHold [26] = dataBDRand;

                simProcessDataAddHold [27] = dataCDRand;

                [growthAddTimeBDDisplay setDoubleValue:simProcessDataAddHold [0]];

                [growthAddTimeTDDisplay setDoubleValue:simProcessDataAddHold [1]];

                [growthAddTimeCFDisplay setDoubleValue:simProcessDataAddHold [2]];

**double** percentTemp = dataBD/(**double**)totalNumberOfDiv;

**int** percentInt = (**int**)(percentTemp*10000);

                percentTemp = percentInt/(**double**)100;

                [growthAddBDDisplay setDoubleValue:percentTemp];

                percentTemp = dataTD/(**double**)totalNumberOfDiv;

                percentInt = (**int**)(percentTemp*10000);

                percentTemp = percentInt/(**double**)100;

                [growthAddTDDisplay setDoubleValue:percentTemp];

                [growthAddBDCDDisplay setDoubleValue:simProcessDataAddHold [5]];

                [growthAddNOCDDisplay setDoubleValue:simProcessDataAddHold [6]];

                [growthAddBDCFDisplay setDoubleValue:simProcessDataAddHold [7]];

                percentTemp = dataBDCFBD/(**double**)totalNumberOfDiv;

                percentInt = (**int**)(percentTemp*10000);

                percentTemp = percentInt/(**double**)100;

                [growthAddBDCFBDDisplay setDoubleValue:percentTemp];

                percentTemp = dataBDCFTD/(**double**)totalNumberOfDiv;

                percentInt = (**int**)(percentTemp*10000);

                percentTemp = percentInt/(**double**)100;

                [growthAddBDCFTDDisplay setDoubleValue:percentTemp];

                [growthAddBDCFCDDisplay setDoubleValue:simProcessDataAddHold [10]];

                [growthAddTDCFDisplay setDoubleValue:simProcessDataAddHold [11]];

                percentTemp = dataTDCFBD/(**double**)totalNumberOfDiv;

                percentInt = (**int**)(percentTemp*10000);

                percentTemp = percentInt/(**double**)100;

                [growthAddTDCFBDDisplay setDoubleValue:percentTemp];

                percentTemp = dataTDCFTD/(**double**)totalNumberOfDiv;

                percentInt = (**int**)(percentTemp*10000);

                percentTemp = percentInt/(**double**)100;

                [growthAddTDCFTDDisplay setDoubleValue:percentTemp];

                [growthAddTDCFCDDisplay setDoubleValue:simProcessDataAddHold [14]];

                percentTemp = dataTDBD/(**double**)totalNumberOfDiv;

                percentInt = (**int**)(percentTemp*10000);

                percentTemp = percentInt/(**double**)100;

                [growthAddTDBDDisplay setDoubleValue:percentTemp];

                percentTemp = dataTDTD/(**double**)totalNumberOfDiv;

                percentInt = (**int**)(percentTemp*10000);

                percentTemp = percentInt/(**double**)100;

                [growthAddTDTDDisplay setDoubleValue:percentTemp];

                [growthAddTDCDDisplay setDoubleValue:simProcessDataAddHold [17]];

                [growthAddMulTDTDDisplay setDoubleValue:simProcessDataAddHold [18]];

                [growthAddSupTDBDDisplay setDoubleValue:simProcessDataAddHold [19]];

                percentTemp = nonDivLing/(**double**)numberOfLing;

                percentInt = (**int**)(percentTemp*10000);

                percentTemp = percentInt/(**double**)100;

                [growthAddNoDivDisplay setDoubleValue:percentTemp];

                percentTemp = recoveryGR/(**double**)numberOfLing;

                percentInt = (**int**)(percentTemp*10000);

                percentTemp = percentInt/(**double**)100;

                [growthAddRecovDisplay setDoubleValue:percentTemp];

**delete** [] simulationDistributionAddData;

                simulationDistributionAddData = **new** **int** [timePointMax*11+100];

                simulationDistributionAddDataCount = timePointMax*11;

**for** (**int** counter1 = 0; counter1 < timePointMax; counter1++){

                    simulationDistributionAddData [counter1*11] = dataCDOccurence [counter1];

                    simulationDistributionAddData [counter1*11+1] = dataNonDivCDOccurence [counter1];

                    simulationDistributionAddData [counter1*11+2] = dataBDCFOccurence [counter1];

                    simulationDistributionAddData [counter1*11+3] = dataBDCFCDOccurence [counter1];

                    simulationDistributionAddData [counter1*11+4] = dataTDCFOccurence [counter1];

                    simulationDistributionAddData [counter1*11+5] = dataTDCFCDOccurence [counter1];

                    simulationDistributionAddData [counter1*11+6] = dataTDCDOccurence [counter1];

                    simulationDistributionAddData [counter1*11+7] = dataDoublingTimeBD [counter1];

                    simulationDistributionAddData [counter1*11+8] = dataDoublingTimeTD [counter1];

                    simulationDistributionAddData [counter1*11+9] = dataDoublingTimeCF [counter1];

                    simulationDistributionAddData [counter1*11+10] = dataVariationToTheFirstDV [counter1];

                }

**delete** [] dataCDOccurence;

**delete** [] dataNonDivCDOccurence ;

**delete** [] dataBDCFOccurence;

**delete** [] dataBDCFCDOccurence;

**delete** [] dataTDCFOccurence;

**delete** [] dataTDCFCDOccurence;

**delete** [] dataTDCDOccurence;

**delete** [] dataDoublingTimeBD;

**delete** [] dataDoublingTimeTD;

**delete** [] dataDoublingTimeCF;

**delete** [] arrayCellLineageTemp;

**delete** [] dataVariationToTheFirstDV;

                NSSound *sound = [NSSound soundNamed:@"Ping"];

                [sound play];

            }

**else**{

                NSAlert *alert = [[NSAlert alloc] init];

                [alert addButtonWithTitle:@"OK"];

                [alert setMessageText:@"List is empty"];

                [alert setAlertStyle:NSAlertStyleWarning];

                [alert runModal];

                [alert release];

                NSSound *sound = [NSSound soundNamed:@"Hero"];

                [sound play];

            }

        }

**else**{

            NSOpenPanel *openDlg = [NSOpenPanel openPanel];

            [openDlg setCanChooseFiles:**YES**];

            [openDlg setCanChooseDirectories:**YES**];

**if** ([openDlg runModal] == NSModalResponseOK){

                NSArray *files = [openDlg URLs];

                NSString *fileName = [[files objectAtIndex:0] absoluteString];

                string directryPathImport = [fileName UTF8String];

**int** findString1 = (**int**)directryPathImport.find("/Users/");

**if** (findString1 == -1) findString1 = (**int**)directryPathImport.find("/Volumes/");

**unsigned** **long** directoryLength = directryPathImport.length();

                directryPathImport = directryPathImport.substr((**unsigned** **long**)findString1, directoryLength-(**unsigned** **long**)findString1);

                string extreactedID;

                string extreactedID2;

**int** terminationFlag = 0;

**do**{

                    terminationFlag = 1;

                    findString1 = (**int**)directryPathImport.find("%20");

**if** (findString1 != -1){

                        extreactedID2 = directryPathImport.substr(0, (**unsigned** **long**)findString1);

                        directryPathImport = directryPathImport.substr((**unsigned** **long**)findString1+3);

                        directryPathImport = extreactedID2+" "+directryPathImport;

                    }

**else** terminationFlag = 0;

                } **while** (terminationFlag == 1);

                extreactedID = directryPathImport;

                string getString;

                ifstream fin;

                fin.open(extreactedID.c_str(),ios::**in**);

**if** (fin.is_open()){

                    getline(fin, getString);

**if** (getString == "Dose data array"){

                        getline(fin, getString);

                        simProcessDataAddHold [0] = atof(getString.c_str());

                        getline(fin, getString);

                        simProcessDataAddHold [1] = atof(getString.c_str());

                        getline(fin, getString);

                        simProcessDataAddHold [2] = atof(getString.c_str());

                        getline(fin, getString);

                        simProcessDataAddHold [3] = atof(getString.c_str());

                        getline(fin, getString);

                        simProcessDataAddHold [4] = atof(getString.c_str());

                        getline(fin, getString);

                        simProcessDataAddHold [5] = atof(getString.c_str());

                        getline(fin, getString);

                        simProcessDataAddHold [6] = atof(getString.c_str());

                        getline(fin, getString);

                        simProcessDataAddHold [7] = atof(getString.c_str());

                        getline(fin, getString);

                        simProcessDataAddHold [8] = atof(getString.c_str());

                        getline(fin, getString);

                        simProcessDataAddHold [9] = atof(getString.c_str());

                        getline(fin, getString);

                        simProcessDataAddHold [10] = atof(getString.c_str());

                        getline(fin, getString);

                        simProcessDataAddHold [11] = atof(getString.c_str());

                        getline(fin, getString);

                        simProcessDataAddHold [12] = atof(getString.c_str());

                        getline(fin, getString);

                        simProcessDataAddHold [13] = atof(getString.c_str());

                        getline(fin, getString);

                        simProcessDataAddHold [14] = atof(getString.c_str());

                        getline(fin, getString);

                        simProcessDataAddHold [15] = atof(getString.c_str());

                        getline(fin, getString);

                        simProcessDataAddHold [16] = atof(getString.c_str());

                        getline(fin, getString);

                        simProcessDataAddHold [17] = atof(getString.c_str());

                        getline(fin, getString);

                        simProcessDataAddHold [18] = atof(getString.c_str());

                        getline(fin, getString);

                        simProcessDataAddHold [19] = atof(getString.c_str());

                        getline(fin, getString);

                        simProcessDataAddHold [20] = atof(getString.c_str());

                        getline(fin, getString);

                        simProcessDataAddHold [21] = atof(getString.c_str());

                        getline(fin, getString);

                        simProcessDataAddHold [22] = atof(getString.c_str());

                        getline(fin, getString);

                        simProcessDataAddHold [23] = atof(getString.c_str());

                        getline(fin, getString);

                        simProcessDataAddHold [24] = atof(getString.c_str());

                        getline(fin, getString);

                        simProcessDataAddHold [25] = atof(getString.c_str());

                        getline(fin, getString);

                        simProcessDataAddHold [26] = atof(getString.c_str());

                        getline(fin, getString);

                        simProcessDataAddHold [27] = atof(getString.c_str());

                        [growthAddTimeBDDisplay setDoubleValue:simProcessDataAddHold [0]];

                        [growthAddTimeTDDisplay setDoubleValue:simProcessDataAddHold [1]];

                        [growthAddTimeCFDisplay setDoubleValue:simProcessDataAddHold [2]];

**int** dataBD = (**int**)simProcessDataAddHold [3];

**int** dataTD = (**int**)simProcessDataAddHold [4];

**int** dataBDCFBD = (**int**)simProcessDataAddHold [8];

**int** dataBDCFTD = (**int**)simProcessDataAddHold [9];

**int** dataTDCFBD = (**int**)simProcessDataAddHold [12];

**int** dataTDCFTD = (**int**)simProcessDataAddHold [13];

**int** dataTDBD = (**int**)simProcessDataAddHold [15];

**int** dataTDTD = (**int**)simProcessDataAddHold [16];

**int** nonDivLing = (**int**)simProcessDataAddHold [20];

**int** recoveryGR = (**int**)simProcessDataAddHold [21];

**int** totalNumberOfDiv = (**int**)simProcessDataAddHold [23];

**int** numberOfLing = (**int**)simProcessDataAddHold [24];

**int** timePointMax = (**int**)simProcessDataAddHold [25];

**double** percentTemp = dataBD/(**double**)totalNumberOfDiv;

**int** percentInt = (**int**)(percentTemp*10000);

                        percentTemp = percentInt/(**double**)100;

                        [growthAddBDDisplay setDoubleValue:percentTemp];

                        percentTemp = dataTD/(**double**)totalNumberOfDiv;

                        percentInt = (**int**)(percentTemp*10000);

                        percentTemp = percentInt/(**double**)100;

                        [growthAddTDDisplay setDoubleValue:percentTemp];

                        [growthAddBDCDDisplay setDoubleValue:simProcessDataAddHold [5]];

                        [growthAddNOCDDisplay setDoubleValue:simProcessDataAddHold [6]];

                        [growthAddBDCFDisplay setDoubleValue:simProcessDataAddHold [7]];

                        percentTemp = dataBDCFBD/(**double**)totalNumberOfDiv;

                        percentInt = (**int**)(percentTemp*10000);

                        percentTemp = percentInt/(**double**)100;

                        [growthAddBDCFBDDisplay setDoubleValue:percentTemp];

                        percentTemp = dataBDCFTD/(**double**)totalNumberOfDiv;

                        percentInt = (**int**)(percentTemp*10000);

                        percentTemp = percentInt/(**double**)100;

                        [growthAddBDCFTDDisplay setDoubleValue:percentTemp];

                        [growthAddBDCFCDDisplay setDoubleValue:simProcessDataAddHold [10]];

                        [growthAddTDCFDisplay setDoubleValue:simProcessDataAddHold [11]];

                        percentTemp = dataTDCFBD/(**double**)totalNumberOfDiv;

                        percentInt = (**int**)(percentTemp*10000);

                        percentTemp = percentInt/(**double**)100;

                        [growthAddTDCFBDDisplay setDoubleValue:percentTemp];

                        percentTemp = dataTDCFTD/(**double**)totalNumberOfDiv;

                        percentInt = (**int**)(percentTemp*10000);

                        percentTemp = percentInt/(**double**)100;

                        [growthAddTDCFTDDisplay setDoubleValue:percentTemp];

                        [growthAddTDCFCDDisplay setDoubleValue:simProcessDataAddHold [14]];

                        percentTemp = dataTDBD/(**double**)totalNumberOfDiv;

                        percentInt = (**int**)(percentTemp*10000);

                        percentTemp = percentInt/(**double**)100;

                        [growthAddTDBDDisplay setDoubleValue:percentTemp];

                        percentTemp = dataTDTD/(**double**)totalNumberOfDiv;

                        percentInt = (**int**)(percentTemp*10000);

                        percentTemp = percentInt/(**double**)100;

                        [growthAddTDTDDisplay setDoubleValue:percentTemp];

                        [growthAddTDCDDisplay setDoubleValue:simProcessDataAddHold [17]];

                        [growthAddMulTDTDDisplay setDoubleValue:simProcessDataAddHold [18]];

                        [growthAddSupTDBDDisplay setDoubleValue:simProcessDataAddHold [19]];

                        percentTemp = nonDivLing/(**double**)numberOfLing;

                        percentInt = (**int**)(percentTemp*10000);

                        percentTemp = percentInt/(**double**)100;

                        [growthAddNoDivDisplay setDoubleValue:percentTemp];

                        percentTemp = recoveryGR/(**double**)numberOfLing;

                        percentInt = (**int**)(percentTemp*10000);

                        percentTemp = percentInt/(**double**)100;

                        [growthAddRecovDisplay setDoubleValue:percentTemp];

**delete** [] simulationDistributionAddData;

                        simulationDistributionAddData = **new** **int** [timePointMax*11+100];

                        simulationDistributionAddDataCount = timePointMax*11;

**int** entryCount = 0;

                        getline(fin, getString);

**if** (getString == "A1"){

**do**{

                                terminationFlag = 1;

                                getline(fin, getString);

**if** (getString != "End"){

                                    simulationDistributionAddData [entryCount*11] = atoi(getString.c_str()), entryCount++;

                                }

**else** terminationFlag = 0;

                            } **while** (terminationFlag == 1);

                        }

                        getline(fin, getString);

                        entryCount = 0;

**if** (getString == "A2"){

**do**{

                                terminationFlag = 1;

                                getline(fin, getString);

**if** (getString != "End"){

                                    simulationDistributionAddData [entryCount*11+1] = atoi(getString.c_str()), entryCount++;

                                }

**else** terminationFlag = 0;

                            } **while** (terminationFlag == 1);

                        }

                        getline(fin, getString);

                        entryCount = 0;

**if** (getString == "A3"){

**do**{

                                terminationFlag = 1;

                                getline(fin, getString);

**if** (getString != "End"){

                                    simulationDistributionAddData [entryCount*11+2] = atoi(getString.c_str()), entryCount++;

                                }

**else** terminationFlag = 0;

                            } **while** (terminationFlag == 1);

                        }

                        getline(fin, getString);

                        entryCount = 0;

**if** (getString == "A4"){

**do**{

                                terminationFlag = 1;

                                getline(fin, getString);

**if** (getString != "End"){

                                    simulationDistributionAddData [entryCount*11+3] = atoi(getString.c_str()), entryCount++;

                                }

**else** terminationFlag = 0;

                            } **while** (terminationFlag == 1);

                        }

                        getline(fin, getString);

                        entryCount = 0;

**if** (getString == "A5"){

**do**{

                                terminationFlag = 1;

                                getline(fin, getString);

**if** (getString != "End"){

                                    simulationDistributionAddData [entryCount*11+4] = atoi(getString.c_str()), entryCount++;

                                }

**else** terminationFlag = 0;

                            } **while** (terminationFlag == 1);

                        }

                        getline(fin, getString);

                        entryCount = 0;

**if** (getString == "A6"){

**do**{

                                terminationFlag = 1;

                                getline(fin, getString);

**if** (getString != "End"){

                                    simulationDistributionAddData [entryCount*11+5] = atoi(getString.c_str()), entryCount++;

                                }

**else** terminationFlag = 0;

                            } **while** (terminationFlag == 1);

                        }

                        getline(fin, getString);

                        entryCount = 0;

**if** (getString == "A7"){

**do**{

                                terminationFlag = 1;

                                getline(fin, getString);

**if** (getString != "End"){

                                    simulationDistributionAddData [entryCount*11+6] = atoi(getString.c_str()), entryCount++;

                                }

**else** terminationFlag = 0;

                            } **while** (terminationFlag == 1);

                        }

                        getline(fin, getString);

                        entryCount = 0;

**if** (getString == "A8"){

**do**{

                                terminationFlag = 1;

                                getline(fin, getString);

**if** (getString != "End"){

                                    simulationDistributionAddData [entryCount*11+7] = atoi(getString.c_str()), entryCount++;

                                }

**else** terminationFlag = 0;

                            } **while** (terminationFlag == 1);

                        }

                        getline(fin, getString);

                        entryCount = 0;

**if** (getString == "A9"){

**do**{

                                terminationFlag = 1;

                                getline(fin, getString);

**if** (getString != "End"){

                                    simulationDistributionAddData [entryCount*11+8] = atoi(getString.c_str()), entryCount++;

                                }

**else** terminationFlag = 0;

                            } **while** (terminationFlag == 1);

                        }

                        getline(fin, getString);

                        entryCount = 0;

**if** (getString == "A10"){

**do**{

                                terminationFlag = 1;

                                getline(fin, getString);

**if** (getString != "End"){

                                    simulationDistributionAddData [entryCount*11+9] = atoi(getString.c_str()), entryCount++;

                                }

**else** terminationFlag = 0;

                            } **while** (terminationFlag == 1);

                        }

                        getline(fin, getString);

                        entryCount = 0;

**if** (getString == "A11"){

**do**{

                                terminationFlag = 1;

                                getline(fin, getString);

**if** (getString != "End"){

                                    simulationDistributionAddData [entryCount*11+10] = atoi(getString.c_str()), entryCount++;

                                }

**else** terminationFlag = 0;

                            } **while** (terminationFlag == 1);

                        }

                        NSSound *sound = [NSSound soundNamed:@"Ping"];

                        [sound play];

                    }

**else**{

                        NSAlert *alert = [[NSAlert alloc] init];

                        [alert addButtonWithTitle:@"OK"];

                        [alert setMessageText:@"No Sim database file"];

                        [alert setAlertStyle:NSAlertStyleWarning];

                        [alert runModal];

                        [alert release];

                        NSSound *sound = [NSSound soundNamed:@"Hero"];

                        [sound play];

                    }

                    fin.close();

                }

            }

        }

    }

**else**{

        NSAlert *alert = [[NSAlert alloc] init];

        [alert addButtonWithTitle:@"OK"];

        [alert setMessageText:@"Sim is in progress"];

        [alert setAlertStyle:NSAlertStyleWarning];

        [alert runModal];

        [alert release];

        NSSound *sound = [NSSound soundNamed:@"Hero"];

        [sound play];

    }

}
