# Supplementary material for: Empirical single-cell tracking and cell-fate simulation reveal dual roles of p53 in tumor suppression
Source: eLife. 2022 Sep 20;11:e72498. doi: 10.7554/eLife.72498 (PMC9560164; doi:10.7554/eLife.72498)
Supplement: Figure 7—source code 3. [file elife-72498-fig7-code3.docx]

//

//  SimulationPerform.m

//  Lineage_Analysis

//

//  Created by Masahiko Sato

//

/*

 Simulation: the first condition

 The following code is written in Objective-C/C++ using Xcode.

 This is the code for the main simulation process. growthPerformSet is used for all modes. midPerform is used for SS and MCS modes.

 Some variables and arrays are defined as Public. We mentioned the types of those variables and arrays. The following code also including ones that are used for a process check and monitor display.

 */

-(**IBAction**)growthPerformSet:(**id**)sender{

**if** (simulationprogress == 0){ //====(int)simulationprogress: Monitoring the progress of the simulation. If simulationprogress is 1, the process of the array set will be blocked.====

**if** (simulationDistributionDataCount != 0){ //====(int)simulationDistributionDataCount: The number of data points that are entered into the simulationDistributionData array.====

**if** (growthInitHold >= 1 && growthCycleHold > 10){

                dispatch_async(dispatch_get_global_queue(DISPATCH_QUEUE_PRIORITY_DEFAULT, 0), ^(**void**){ //====Bring the process to the background=======

                    simulationprogress = 1;

                    selArraySet = 0; //=====(int) selArraySet: if the second set of the array for simulation is allocated, selArraySet will be 1..=====

                    //======Following code checks whether the Time end of the first (Std, Ind, and MX mode), the second (SS and MCS), and the third (progressive recovery) is set in the right order (related code for the progressive recovery is not included as it has not been used in the current paper). If the order is incorrect, the system will ask to correct it.====

**int** growthCycleBase = 0; //====End Time of the first cycle====

**int** growthCycleMid = 0; //====End time of the second cycle====

**int** growthCycleProgEnd = 0; //====End time of the progressive recoverry====

**int** growthCycleProgEnd2 = 0; //====End of progressive recovery phase: growthCycleProgEnd and growthCycleProgEnd2 can be the same, but if growthCycleProgEnd is < growthCycleProgEnd2, progressive recovery will be applied from the end of the first/second cycle to the end of recovery time.====

**int** growthCycleProgStartTemp = 0;

**int** growthCycleProgEndTemp = 0;

**int** growthCycleProgEndTemp2 = 0;

                    //====(int) growthMidTimeStartHold, growthProgTimeStartHold, growthProgTimeEndHold, growthCycleHold: Those can be set by reading input data.=====

**if** (growthProgTimeEndHold <= growthCycleHold && growthProgTimeStartHold <= growthCycleHold && growthProgTimeEndHold > growthProgTimeStartHold+100){

                        growthCycleProgStartTemp = growthProgTimeStartHold;

**if** (growthCycleHold > growthCycleProgEndTemp+100){

                            growthCycleProgEndTemp = growthProgTimeEndHold;

                            growthCycleProgEndTemp2 = growthCycleHold;

                        }

**else** {

                            growthCycleProgEndTemp = growthCycleHold;

                            growthCycleProgEndTemp2 = growthCycleHold;

                        }

                    }

**if** (growthMidTimeStartHold != 0 && growthCycleHold >= growthMidTimeStartHold+100 && (simProcessDataMiddleHold [3] != 0 || simProcessDataMiddleHold [4] != 0 || simProcessDataMiddleHold [5] != 0 || simProcessDataMiddleHold [6] != 0)){

                        growthCycleBase = growthMidTimeStartHold;

**if** (growthCycleProgStartTemp != 0 && growthCycleProgStartTemp > growthCycleBase+100 && (simProcessDataProgHold [3] != 0 || simProcessDataProgHold [4] != 0 || simProcessDataProgHold [5] != 0 || simProcessDataProgHold [6] != 0)){

                            growthCycleMid = growthCycleProgStartTemp;

                            growthCycleProgEnd = growthCycleProgEndTemp;

                            growthCycleProgEnd2 = growthCycleProgEndTemp2;

                        }

**else** growthCycleMid = growthCycleHold;

                    }

**else** **if** (growthCycleProgStartTemp != 0 && growthCycleProgEndTemp > 100 && (simProcessDataProgHold [3] != 0 || simProcessDataProgHold [4] != 0 || simProcessDataProgHold [5] != 0 || simProcessDataProgHold [6] != 0)){

                        growthCycleBase = growthCycleProgStartTemp;

                        growthCycleProgEnd = growthCycleProgEndTemp;

                        growthCycleProgEnd2 = growthCycleProgEndTemp2;

                    }

**else** growthCycleBase = growthCycleHold;

                    //====Following code is used to display lineage information, (int) selectLingNoHold defines (int)*lingNoAssigineSim array size. lingNoAssigineSim hold lineage information to display or export.====

**int** doseSimStatusCheck = 0;

**if** (selectLingNoHold > growthInitHold*(**double**)0.1) selectLingNoHold = (**int**)(round(growthInitHold*(**double**)0.1));

**if** (lingNoAssigineSimStatus == 1) **delete** [] lingNoAssigineSim;

                    lingNoAssigineSim = **new** **int** [selectLingNoHold+10];

                    lingNoAssigineSimCount = 0;

                    lingNoAssigineSimStatus = 1;

                    //=====(int)doseSimStatusHold: If doseSimStatusHold is 0, the mode will be Std or MX. If doseSimStatusHold is 1, the mode will be Ind. If doseSimStatusHold is 2, the mode will be MX or MCS. messageStringSim, messageStringSim2, messageStringSim3, and messageStringSim4 will be used to display messeages.====

**if** (doseSimStatusHold == 1){

**if** (doseBaseHold >= 0 && doseMiddleHold >= 0 && doseTargetHold > doseBaseHold && doseBaseHold < doseMiddleHold && doseTargetHold < doseMiddleHold && (simProcessDataMiddleHold [3] != 0 || simProcessDataMiddleHold [4] != 0 || simProcessDataMiddleHold [5] != 0 || simProcessDataMiddleHold [6] != 0)){

                            growthCycleBase = growthCycleHold;

                            growthCycleMid = 0;

                            growthCycleProgEnd = 0;

                            growthCycleProgEnd2 = 0;

                            messageStringSim = to_string(doseTargetHold);

                            messageStringSim2 = "nil";

                            messageStringSim3 = "nil";

                            messageStringSim4 = "nil";

                            doseSimStatusCheck = 1;

                        }

                    }

**else** **if** (doseSimStatusHold == 2){

**if** (selectLingNoHold != 0 && selectLingNoHold < growthInitHold){

**if** (growthCycleMid != 0){

**if** ((simProcessDataProgHold [3] != 0 || simProcessDataProgHold [4] != 0 || simProcessDataProgHold [5] != 0 || simProcessDataProgHold [6] != 0) && (simProcessDataAddHold [3] != 0 || simProcessDataAddHold [4] != 0 || simProcessDataAddHold [5] != 0 || simProcessDataAddHold [6] != 0)){

                                    growthCycleProgEnd = 0;

                                    growthCycleProgEnd2 = 0;

                                    messageStringSim3 = "nil";

                                    messageStringSim4 = "nil";

                                    doseSimStatusCheck = 1;

                                }

**else**{

**if** (simProcessDataProgHold [3] != 0 || simProcessDataProgHold [4] != 0 || simProcessDataProgHold [5] != 0 || simProcessDataProgHold [6] != 0){

                                        growthCycleBase = growthCycleHold;

                                        growthCycleMid = 0;

                                        growthCycleProgEnd = 0;

                                        growthCycleProgEnd2 = 0;

                                        messageStringSim2 = "nil";

                                        messageStringSim3 = "nil";

                                        messageStringSim4 = "nil";

                                        doseSimStatusCheck = 1;

                                    }

                                }

                            }

**else**{

**if** (simProcessDataProgHold [3] != 0 || simProcessDataProgHold [4] != 0 || simProcessDataProgHold [5] != 0 || simProcessDataProgHold [6] != 0){

                                    growthCycleBase = growthCycleHold;

                                    growthCycleMid = 0;

                                    growthCycleProgEnd = 0;

                                    growthCycleProgEnd2 = 0;

                                    messageStringSim2 = "nil";

                                    messageStringSim3 = "nil";

                                    messageStringSim4 = "nil";

                                    doseSimStatusCheck = 1;

                                }

                            }

**if** (doseSimStatusCheck == 1){

**int** randLing = 0;

**int** termibationFlag = 0;

**int** breakSim = 0;

**int** missCount = 0;

**do**{

                                    termibationFlag = 1;

                                    randLing = rand() % growthInitHold + 1;

                                    breakSim = 0;

**for** (**int** counter1 = 0; counter1 < lingNoAssigineSimCount; counter1++){

**if** (lingNoAssigineSim [counter1] == randLing){

                                            breakSim = 1;

                                            missCount++;

                                        }

                                    }

**if** (breakSim == 0) lingNoAssigineSim [lingNoAssigineSimCount] = randLing, lingNoAssigineSimCount++;

**if** (lingNoAssigineSimCount == selectLingNoHold || missCount > 200) termibationFlag = 0;

                                } **while** (termibationFlag == 1);

                                messageLingSim = "1: nil";

                                messageLingSim2 = "2: nil";

                                messageLingSim3 = "3: nil";

                                messageLingSim4 = "4: nil";

                                messageLingSim5 = "5: nil";

                                messageLingSim6 = "6: nil";

                                messageLingSim7 = "7: nil";

                                messageLingSim8 = "8: nil";

**if** (selectLingNoHold >= 1) messageLingSim = "1: L"+to_string(lingNoAssigineSim [0]);

**if** (selectLingNoHold >= 2) messageLingSim2 = "2: L"+to_string(lingNoAssigineSim [1]);

**if** (selectLingNoHold >= 3) messageLingSim3 = "3: L"+to_string(lingNoAssigineSim [2]);

**if** (selectLingNoHold >= 4) messageLingSim4 = "4: L"+to_string(lingNoAssigineSim [3]);

**if** (selectLingNoHold >= 5) messageLingSim5 = "5: L"+to_string(lingNoAssigineSim [4]);

**if** (selectLingNoHold >= 6) messageLingSim6 = "6: L"+to_string(lingNoAssigineSim [5]);

**if** (selectLingNoHold >= 7) messageLingSim7 = "7: L"+to_string(lingNoAssigineSim [6]);

**if** (selectLingNoHold >= 8) messageLingSim8 = "8: L"+to_string(lingNoAssigineSim [7]);

                                lingSetDisplayCall = 1;

                            }

                        }

                    }

**if** (endVariationtHold > 90) endVariationtHold = 90; //====AendVariationtHold holds a value to add variation at the end of empirical data to make the growth curve smooth. The offset value is 30, and the max value is 90.====

                    //********If all variables are set correctly, the simulation will start.=====

**if** (doseSimStatusHold == 0 || (doseSimStatusHold == 1 && doseSimStatusCheck == 1) || (doseSimStatusHold == 2 && doseSimStatusCheck == 1)){

                        /*

                         All arrays used in the simulation are allocated statically. Speed of simulation is significantly reduced when memory is allocated dynamically. We reserve memory for those arrays (Public) when the simulation window is open. When the data size reaches the limit of those arrays, the size of those arrays will be expanded.

                         */

                        activeCellStatusList = **new** **int** [10000]; //====activeCellStatusList holds the status of cells that are under the simulation. This list will be updated following the creation of new cells. Cells, of which simulations have been completed, will be removed from the list.======

                        activeCellStatusListCount = 0;

                        activeCellStatusListLimitHold = 10000;

                        /*

                         ******"activeCellStatusList, activeCellStatusListHold, activeCellStatusListKeep"******

                         1. Cell no

                         2. Sibling cell no 1 //------Set -1 when info is entered into the array list--

                         3. Sibling cell no 2

                         4. Lineage no.

                         5. Doubling time:

                         First-round: Start 1 and assigning Doubling time, or use initial length to an Event

                         The first round: use BD time. The following cycle: If the TD flag is ON, use TD time.

                         The doubling time limit applies (add bais based on the doubling time of the previous one) to determine the variation based on the lineage data.

                         6. End event set based on the frequency of BD, TD, CD, CF, and nonDiv.

                         First-round: apply nonDiv (no growth)--apply recovery % (recovery %, use once, remaining ones are non-growing till the end or undergo CD, use nonDiv CD parameter)

                         First-round (remaining): Determine based on BD, TD, and nonDivCD frequency.

                         Following round: Determine based on BD, TD, and CF frequency.

                         If the TD-BD count is ON, and the TD-BD limit is set, the BD chance is reduced by 1/3.

                         End Event Type mark: BD:1 or 7 (BD for recovery), TD:2, CD:3, NonDiv: 4 (if recovery does not apply, set 5), CF: 6, Reaches Max division: 9

                         End Event set based on the frequency of TD-CF, TD-BD, TD-TD, and TD-CD: IF the TD is the second TD (the TD-TD limit on), exclude TD (in this case, BD is also excluded).

                         End event set based on TD-CF-BD, TD-CF-TD, and TD-CF-CD frequency: If the TD is the second-TD, TD, and BD will be excluded.

                         7. Process flag: For event type determination, second for fusion, hold fusion partner cell no

                         8. TD count

                         9. If TD-TD occurred, a flag will be set.

                         10. If TD-BD occurred, a count will be set. If a BD occurs in the following event, the count will be -1. If the count reaches 0, the BD suppression will be removed.

                         11. Parent cell no.

                         12. Previous double time.

                         */

**int** *activeCellStatusListHold = **new** **int** [10000]; //=====This local array holds the initial status of activeCellStatusList. This array will not be updated during one cycle of simulation.====

**int** activeCellStatusListHoldCount = 0;

                        activeCellStatusListKeep = **new** **int** [600000]; //====activeCellStatusListKeep holds the status of cells, of which simulation has been completed. Corresponding data will be copied from activeCellStatusListHold.=====

                        activeCellStatusListKeepCount = 0;

                        activeCellStatusListKeepLimit = 600000;

                        cellLineageTempArray = **new** **int** [100000000000]; //====This array temporarily holds cell lineage data.====

                        cellLineageTempArrayCount = 0;

                        cellLineageTempArrayLimit = 100000000000;

                        cellLineageSummaryArray = **new** **long** [100000000]; //====This array holds a summary of each cell.====

                        cellLineageSummaryArrayCount = 0;

                        cellLineageSummaryArrayLimit = 100000000;

                        /*

                         ******cellLineageTempArray******

                         1.Start time

                         2.X position

                         3.Y position

                         4.Event type

                         5.Partner cell No.

                         6.Cell No.

                         7.Lineage No.

                         8.Pertner Lineage No.

                         9.Color paint no (use when cell lineage data is displayed)

                         ******"cellLineageSummaryArray"******

                         1.Start time

                         2.End time

                         3.Lineage no.

                         4.Cell no.

                         5.Parent cell no.

                         6.Partner lineage no.

                         7.Start event

                         8.End Event

                         9.Fusion time by event 92

                         */

                        //====Creation of simulation arrays=====

                        //Following code create simulation arrays from source data arrays.

**int** totalNoOfEntryFirstDV = 0;

**int** totalNoOfEntryDoubBD = 0;

**int** totalNoOfEntryDoubTD = 0;

**int** totalNoOfEntryDoubCF = 0;

                        totalNoOfNonDivCD = 0;

**int** totalNoOfBDCD = 0;

**int** totalNoOfBDCF = 0;

**int** totalNoOfBDCFCD = 0;

**int** totalNoOfTDCF = 0;

**int** totalNoOfTDCFCD = 0;

**int** totalNoOfTDCD = 0;

                        //=====Count the number of events======

**for** (**int** counter1 = 0; counter1 < simulationDistributionDataCount/11; counter1++){

**if** (simulationDistributionData [counter1*11] != 0) totalNoOfBDCD = totalNoOfBDCD+simulationDistributionData [counter1*11];

**if** (simulationDistributionData [counter1*11+1] != 0) totalNoOfNonDivCD = totalNoOfNonDivCD+simulationDistributionData [counter1*11+1];

**if** (simulationDistributionData [counter1*11+2] != 0) totalNoOfBDCF = totalNoOfBDCF+simulationDistributionData [counter1*11+2];

**if** (simulationDistributionData [counter1*11+3] != 0) totalNoOfBDCFCD = totalNoOfBDCFCD+simulationDistributionData [counter1*11+3];

**if** (simulationDistributionData [counter1*11+4] != 0) totalNoOfTDCF = totalNoOfTDCF+simulationDistributionData [counter1*11+4];

**if** (simulationDistributionData [counter1*11+5] != 0) totalNoOfTDCFCD = totalNoOfTDCFCD+simulationDistributionData [counter1*11+5];

**if** (simulationDistributionData [counter1*11+6] != 0) totalNoOfTDCD = totalNoOfTDCD+simulationDistributionData [counter1*11+6];

**if** (simulationDistributionData [counter1*11+7] != 0) totalNoOfEntryDoubBD = totalNoOfEntryDoubBD+simulationDistributionData [counter1*11+7];

**if** (simulationDistributionData [counter1*11+8] != 0) totalNoOfEntryDoubTD = totalNoOfEntryDoubTD+simulationDistributionData [counter1*11+8];

**if** (simulationDistributionData [counter1*11+9] != 0) totalNoOfEntryDoubCF = totalNoOfEntryDoubCF+simulationDistributionData [counter1*11+9];

**if** (simulationDistributionData [counter1*11+10] != 0) totalNoOfEntryFirstDV = totalNoOfEntryFirstDV+simulationDistributionData [counter1*11+10];

                        }

                        //=======Apply a bias=======

                        /*

                         When Source data is created, information related to each event is displayed. The values of each event frequency can be changed manually and changed values will be taken into account when a simulation is performed. Those are not used in the current paper.

                         */

                        totalNoOfEntryDoubBD = (**int**)(round(totalNoOfEntryDoubBD*(**double**)simProcessDataBaseHold [0]));

                        totalNoOfEntryDoubTD = (**int**)(round(totalNoOfEntryDoubTD*(**double**)simProcessDataBaseHold [1]));

                        totalNoOfEntryDoubCF = (**int**)(round(totalNoOfEntryDoubCF*(**double**)simProcessDataBaseHold [2]));

                        totalNoOfBDCD = (**int**)(round(totalNoOfBDCD*(**double**)simProcessDataBaseHold [5]));

                        totalNoOfNonDivCD = (**int**)(round(totalNoOfNonDivCD*(**double**)simProcessDataBaseHold [6]));

                        totalNoOfBDCF = (**int**)(round(totalNoOfBDCF*(**double**)simProcessDataBaseHold [7]));

                        totalNoOfBDCFCD = (**int**)(round(totalNoOfBDCFCD*(**double**)simProcessDataBaseHold [10]));

                        totalNoOfTDCF = (**int**)(round(totalNoOfTDCF*(**double**)simProcessDataBaseHold [11]));

                        totalNoOfTDCFCD = (**int**)(round(totalNoOfTDCFCD*(**double**)simProcessDataBaseHold [14]));

                        totalNoOfTDCD = (**int**)(round(totalNoOfTDCD*(**double**)simProcessDataBaseHold [17]));

                        /*

                         Those arrays can be defined as Local and be passed to a function, although we defined those as Publick or Private.

                         */

                        expandFirsDVList = **new** **int** [totalNoOfEntryFirstDV*2+1];

                        expandFirsDVListCount = 0;

                        expandDoublingDoubBD = **new** **int** [totalNoOfEntryDoubBD*2+1];

                        expandDoublingDoubBDCount = 0;

                        expandDoublingDoubTD = **new** **int** [totalNoOfEntryDoubTD*2+1];

                        expandDoublingDoubTDCount = 0;

                        expandDoublingDoubCF = **new** **int** [totalNoOfEntryDoubCF*2+1];

                        expandDoublingDoubCFCount = 0;

                        expandBDCD = **new** **int** [totalNoOfBDCD*2+1];

                        expandBDCDCount = 0;

                        expandBDCF = **new** **int** [totalNoOfBDCF*2+1];

                        expandBDCFCount = 0;

                        expandNonCD = **new** **int** [totalNoOfNonDivCD*2+1];

                        expandNonCDCount = 0;

                        expandBDCFCD = **new** **int** [totalNoOfBDCFCD*2+1];

                        expandBDCFCDCount = 0;

                        expandTDCF = **new** **int** [totalNoOfTDCF*2+1];

                        expandTDCFCount = 0;

                        expandTDCFCD = **new** **int** [totalNoOfTDCFCD*2+1];

                        expandTDCFCDCount = 0;

                        expandTDCD = **new** **int** [totalNoOfTDCD*2+1];

                        expandTDCDCount = 0;

**int** countTemp = 0;

**for** (**int** counter1 = 0; counter1 < simulationDistributionDataCount/11; counter1++){

**if** (simulationDistributionData [counter1*11] != 0){ //====Bias: if Bais > 1, no change for Entry 1, Entry > 1, Entry+(Entry-1)*bias====

**if** (simProcessDataBaseHold [5] > 1){

**if** (simulationDistributionData [counter1*11] > 1) countTemp = (**int**)(round(simulationDistributionData [counter1*11]+(simulationDistributionData [counter1*11]-1)*(**double**)simProcessDataBaseHold [5]));

**else** countTemp = simulationDistributionData [counter1*11];

                                }

**else** countTemp = (**int**)(round(simulationDistributionData [counter1*11]*(**double**)simProcessDataBaseHold [5]));

**for** (**int** counter2 = 0; counter2 < countTemp; counter2++){

                                    expandBDCD [expandBDCDCount] = counter1+1, expandBDCDCount++;

                                    expandBDCD [expandBDCDCount] = 0, expandBDCDCount++;

                                }

                            }

**if** (simulationDistributionData [counter1*11+1] != 0){

**if** (simProcessDataBaseHold [6] > 1){

**if** (simulationDistributionData [counter1*11+1] > 1) countTemp = (**int**)(round(simulationDistributionData [counter1*11+1]+(simulationDistributionData [counter1*11+1]-1)*(**double**)simProcessDataBaseHold [6]));

**else** countTemp = simulationDistributionData [counter1*11+1];

                                }

**else** countTemp = (**int**)(round(simulationDistributionData [counter1*11+1]*(**double**)simProcessDataBaseHold [6]));

**for** (**int** counter2 = 0; counter2 < countTemp; counter2++){

                                    expandNonCD [expandNonCDCount] = counter1+1, expandNonCDCount++;

                                    expandNonCD [expandNonCDCount] = 0, expandNonCDCount++;

                                }

                            }

**if** (simulationDistributionData [counter1*11+2] != 0){

**if** (simProcessDataBaseHold [7] > 1){

**if** (simulationDistributionData [counter1*11+2] > 1) countTemp = (**int**)(round(simulationDistributionData [counter1*11+2]+(simulationDistributionData [counter1*11+2]-1)*(**double**)simProcessDataBaseHold [7]));

**else** countTemp = simulationDistributionData [counter1*11+2];

                                }

**else** countTemp = (**int**)(round(simulationDistributionData [counter1*11+2]*(**double**)simProcessDataBaseHold [7]));

**for** (**int** counter2 = 0; counter2 < countTemp; counter2++){

                                    expandBDCF [expandBDCFCount] = counter1+1, expandBDCFCount++;

                                    expandBDCF [expandBDCFCount] = 0, expandBDCFCount++;

                                }

                            }

**if** (simulationDistributionData [counter1*11+3] != 0){

**if** (simProcessDataBaseHold [10] > 1){

**if** (simulationDistributionData [counter1*11+3] > 1) countTemp = (**int**)(round(simulationDistributionData [counter1*11+3]+(simulationDistributionData [counter1*11+3]-1)*(**double**)simProcessDataBaseHold [10]));

**else** countTemp = simulationDistributionData [counter1*11+3];

                                }

**else** countTemp = (**int**)(round(simulationDistributionData [counter1*11+3]*(**double**)simProcessDataBaseHold [10]));

**for** (**int** counter2 = 0; counter2 < countTemp; counter2++){

                                    expandBDCFCD [expandBDCFCDCount] = counter1+1, expandBDCFCDCount++;

                                    expandBDCFCD [expandBDCFCDCount] = 0, expandBDCFCDCount++;

                                }

                            }

**if** (simulationDistributionData [counter1*11+4] != 0){

**if** (simProcessDataBaseHold [11] > 1){

**if** (simulationDistributionData [counter1*11+4] > 1) countTemp = (**int**)(round(simulationDistributionData [counter1*11+4]+(simulationDistributionData [counter1*11+4]-1)*(**double**)simProcessDataBaseHold [11]));

**else** countTemp = simulationDistributionData [counter1*11+4];

                                }

**else** countTemp = (**int**)(round(simulationDistributionData [counter1*11+4]*(**double**)simProcessDataBaseHold [11]));

**for** (**int** counter2 = 0; counter2 < countTemp; counter2++){

                                    expandTDCF [expandTDCFCount] = counter1+1, expandTDCFCount++;

                                    expandTDCF [expandTDCFCount] = 0, expandTDCFCount++;

                                }

                            }

**if** (simulationDistributionData [counter1*11+5] != 0){

**if** (simProcessDataBaseHold [14] > 1){

**if** (simulationDistributionData [counter1*11+5] > 1) countTemp = (**int**)(round(simulationDistributionData [counter1*11+5]+(simulationDistributionData [counter1*11+5]-1)*(**double**)simProcessDataBaseHold [14]));

**else** countTemp = simulationDistributionData [counter1*11+5];

                                }

**else** countTemp = (**int**)(round(simulationDistributionData [counter1*11+5]*(**double**)simProcessDataBaseHold [14]));

**for** (**int** counter2 = 0; counter2 < countTemp; counter2++){

                                    expandTDCFCD [expandTDCFCDCount] = counter1+1, expandTDCFCDCount++;

                                    expandTDCFCD [expandTDCFCDCount] = 0, expandTDCFCDCount++;

                                }

                            }

**if** (simulationDistributionData [counter1*11+6] != 0){

**if** (simProcessDataBaseHold [17] > 1){

**if** (simulationDistributionData [counter1*11+6] > 1) countTemp = (**int**)(round(simulationDistributionData [counter1*11+6]+(simulationDistributionData [counter1*11+6]-1)*(**double**)simProcessDataBaseHold [17]));

**else** countTemp = simulationDistributionData [counter1*11+6];

                                }

**else** countTemp = (**int**)(round(simulationDistributionData [counter1*11+6]*(**double**)simProcessDataBaseHold [17]));

**for** (**int** counter2 = 0; counter2 < countTemp; counter2++){

                                    expandTDCD [expandTDCDCount] = counter1+1, expandTDCDCount++;

                                    expandTDCD [expandTDCDCount] = 0, expandTDCDCount++;

                                }

                            }

**if** (simulationDistributionData [counter1*11+7] != 0){

**if** (simProcessDataBaseHold [0] > 1){

**if** (simulationDistributionData [counter1*11+7] > 1) countTemp = (**int**)(round(simulationDistributionData [counter1*11+7]+(simulationDistributionData [counter1*11+7]-1)*(**double**)simProcessDataBaseHold [0]));

**else** countTemp = simulationDistributionData [counter1*11+7];

                                }

**else** countTemp = (**int**)(round(simulationDistributionData [counter1*11+7]*(**double**)simProcessDataBaseHold [0]));

**for** (**int** counter2 = 0; counter2 < countTemp; counter2++){

                                    expandDoublingDoubBD [expandDoublingDoubBDCount] = counter1+1, expandDoublingDoubBDCount++;

                                    expandDoublingDoubBD [expandDoublingDoubBDCount] = 0, expandDoublingDoubBDCount++;

                                }

                            }

**if** (simulationDistributionData [counter1*11+8] != 0){

**if** (simProcessDataBaseHold [1] > 1){

**if** (simulationDistributionData [counter1*11+8] > 1) countTemp = (**int**)(round(simulationDistributionData [counter1*11+8]+(simulationDistributionData [counter1*11+8]-1)*(**double**)simProcessDataBaseHold [1]));

**else** countTemp = simulationDistributionData [counter1*11+8];

                                }

**else** countTemp = (**int**)(round(simulationDistributionData [counter1*11+8]*(**double**)simProcessDataBaseHold [1]));

**for** (**int** counter2 = 0; counter2 < countTemp; counter2++){

                                    expandDoublingDoubTD [expandDoublingDoubTDCount] = counter1+1, expandDoublingDoubTDCount++;

                                    expandDoublingDoubTD [expandDoublingDoubTDCount] = 0, expandDoublingDoubTDCount++;

                                }

                            }

**if** (simulationDistributionData [counter1*11+9] != 0){

**if** (simProcessDataBaseHold [2] > 1){

**if** (simulationDistributionData [counter1*11+9] > 1) countTemp = (**int**)(round(simulationDistributionData [counter1*11+9]+(simulationDistributionData [counter1*11+9]-1)*(**double**)simProcessDataBaseHold [2]));

**else** countTemp = simulationDistributionData [counter1*11+9];

                                }

**else** countTemp = (**int**)(round(simulationDistributionData [counter1*11+9]*(**double**)simProcessDataBaseHold [2]));

**for** (**int** counter2 = 0; counter2 < countTemp; counter2++){

                                    expandDoublingDoubCF [expandDoublingDoubCFCount] = counter1+1, expandDoublingDoubCFCount++;

                                    expandDoublingDoubCF [expandDoublingDoubCFCount] = 0, expandDoublingDoubCFCount++;

                                }

                            }

**if** (simulationDistributionData [counter1*11+10] != 0){

**for** (**int** counter2 = 0; counter2 < simulationDistributionData [counter1*11+10]; counter2++){

                                    expandFirsDVList [expandFirsDVListCount] = counter1+1, expandFirsDVListCount++;

                                    expandFirsDVList [expandFirsDVListCount] = 0, expandFirsDVListCount++;

                                }

                            }

                        }

                        //====Percentage data expand to 100====

                        firstEventList = **new** **int** [150];

                        secondEventBDList = **new** **int** [150];

                        secondEventBDCFList = **new** **int** [150];

                        secondEventTDList = **new** **int** [150];

                        secondEventTDCFList = **new** **int** [150];

**for** (**int** counter1 = 0; counter1 < 150; counter1++){

                            firstEventList [counter1] = 0;

                            secondEventBDList [counter1] = 0;

                            secondEventBDCFList [counter1] = 0;

                            secondEventTDList [counter1] = 0;

                            secondEventTDCFList [counter1] = 0;

                        }

**int** totalNoOfNonDivCDWithBias = (**int**)(round(totalNoOfNonDivCD*(**double**)simProcessDataBaseHold [6])); //=====Apply baisa value to the total of nonDivCD====

**int** totalNumberOfnonDivLingCD = (**int**)simProcessDataBaseHold [20]+totalNoOfNonDivCDWithBias; //====Sum of nonDiv cell ling. + CD that undergos without cell division (nonDivCD)====

**int** remainingLingNo = (**int**)simProcessDataBaseHold [24]-totalNumberOfnonDivLingCD; //====Remaining no. of cell ling.====

**if** (remainingLingNo < 0) remainingLingNo = 0;

**int** totalNoOfBDTD = (**int**)(simProcessDataBaseHold [3]+simProcessDataBaseHold [4]); //====Sum of BD and TD====

**double** nonDivIn100 = totalNumberOfnonDivLingCD/(**double**)(remainingLingNo+totalNumberOfnonDivLingCD); //====Non div portion of % (non Div Ling + CD without Div)====

**double** divIn100 = remainingLingNo/(**double**)(remainingLingNo+totalNumberOfnonDivLingCD); //====Div portion of % (BD and TD)====

**int** percentBD = 0; //1

**int** percentTD = 0; //2

**int** percentCD = 0; //3

**int** nonDivPercent = 0; //4

**double** percentTemp = 0;

                        //====Determien % of each event A====

**if** (totalNoOfBDTD != 0){

                            percentTemp = (simProcessDataBaseHold [3]/(**double**)totalNoOfBDTD)*100;

                            percentBD = (**int**)(round((percentTemp*divIn100)));

                            percentTemp = (simProcessDataBaseHold [4]/(**double**)totalNoOfBDTD)*100;

                            percentTD = (**int**)(round((percentTemp*divIn100)));

                        }

**if** (totalNumberOfnonDivLingCD != 0){

                            percentTemp = (totalNoOfNonDivCDWithBias/(**double**)totalNumberOfnonDivLingCD)*100;

                            percentCD = (**int**)(round(percentTemp*nonDivIn100));

                            percentTemp = (simProcessDataBaseHold [20]/(**double**)totalNumberOfnonDivLingCD)*100;

                            nonDivPercent = (**int**)(round(percentTemp*nonDivIn100));

                        }

**if** (totalNoOfNonDivCD > simProcessDataBaseHold [4] && percentCD < percentTD){

                            percentBD = (percentBD+percentTD)-percentCD;

                            percentTD = percentCD;

                        }

**else** **if** (totalNoOfNonDivCD < simProcessDataBaseHold [4] && percentCD > percentTD){

                            nonDivPercent = (percentCD+nonDivPercent)-percentTD;

                            percentCD = percentTD;

                        }

                        //====Total of percentBD, percentTD, percentCD, nonDivPercent has to be 100%====

**int** entryCount = 0; //====Enter event type following %=====

**for** (**int** counter1 = 0; counter1 < nonDivPercent; counter1++){

**if** (entryCount < 100) firstEventList [entryCount] = 4, entryCount++;

                        }

**for** (**int** counter1 = 0; counter1 < percentCD; counter1++){

**if** (entryCount < 100) firstEventList [entryCount] = 3, entryCount++;

                        }

**for** (**int** counter1 = 0; counter1 < percentTD; counter1++){

**if** (entryCount < 100) firstEventList [entryCount] = 2, entryCount++;

                        }

**for** (**int** counter1 = 0; counter1 < percentBD; counter1++){

**if** (entryCount < 100) firstEventList [entryCount] = 1, entryCount++;

                        }

**if** (entryCount != 100){ //====If the total % is less than 100 (due to the rounding) fill with 1 (percentBD)====

**for** (**int** counter1 = 0; counter1 < 100; counter1++){

**if** (entryCount < 100) firstEventList [entryCount] = 1, entryCount++;

                            }

                        }

                        //====Determine % of each event B====

**int** totalNoOfAfterBD = (**int**)(simProcessDataBaseHold [3]+simProcessDataBaseHold [4])+totalNoOfBDCD+totalNoOfBDCF;

                        percentBD = 0; //1

                        percentTD = 0; //2

**int** percentBDCD = 0; //5

**int** percentBDCF = 0; //6

**if** (totalNoOfAfterBD != 0) percentBD = (**int**)(round((simProcessDataBaseHold [3]/(**double**)totalNoOfAfterBD)*100));

**if** (totalNoOfAfterBD != 0) percentTD = (**int**)(round((simProcessDataBaseHold [4]/(**double**)totalNoOfAfterBD)*100));

**if** (totalNoOfAfterBD != 0) percentBDCD = (**int**)(round((totalNoOfBDCD/(**double**)totalNoOfAfterBD)*100));

**if** (totalNoOfAfterBD != 0) percentBDCF = (**int**)(round((totalNoOfBDCF/(**double**)totalNoOfAfterBD)*100));

**if** (totalNoOfAfterBD != 0){ //====Low frequency events adjustment: In the case that the % is < 0.5 (due to the rounding, % is 0), % will be 1%. The 1% will be subtracted from others.====

**if** ((simProcessDataBaseHold [3]/(**double**)totalNoOfAfterBD)*100 != 0 && percentBD == 0){

                                percentBD = 1;

**if** (percentTD > 2) percentTD--;

**else** **if** (percentBDCD > 2) percentBDCD--;

**else** **if** (percentBDCF > 2) percentBDCF--;

                            }

**if** ((simProcessDataBaseHold [4]/(**double**)totalNoOfAfterBD)*100 != 0 && percentTD == 0){

                                percentTD = 1;

**if** (percentBD > 2) percentBD--;

**else** **if** (percentBDCD > 2) percentBDCD--;

**else** **if** (percentBDCF > 2) percentBDCF--;

                            }

**if** ((totalNoOfBDCD/(**double**)totalNoOfAfterBD)*100 != 0 && percentBDCD == 0){

                                percentBDCD = 1;

**if** (percentBD > 2) percentBD--;

**else** **if** (percentTD > 2) percentTD--;

**else** **if** (percentBDCF > 2) percentBDCF--;

                            }

**if** ((totalNoOfBDCF/(**double**)totalNoOfAfterBD)*100 != 0 && percentBDCF == 0){

                                percentBDCF = 1;

**if** (percentBD > 2) percentBD--;

**else** **if** (percentTD > 2) percentTD--;

**else** **if** (percentBDCD > 2) percentBDCD--;

                            }

                        }

                        entryCount = 0;

**for** (**int** counter1 = 0; counter1 < percentBDCF; counter1++){

**if** (entryCount < 100) secondEventBDList [entryCount] = 6, entryCount++;

                        }

**for** (**int** counter1 = 0; counter1 < percentBDCD; counter1++){

**if** (entryCount < 100) secondEventBDList [entryCount] = 5, entryCount++;

                        }

**for** (**int** counter1 = 0; counter1 < percentTD; counter1++){

**if** (entryCount < 100) secondEventBDList [entryCount] = 2, entryCount++;

                        }

**for** (**int** counter1 = 0; counter1 < percentBD; counter1++){

**if** (entryCount < 100) secondEventBDList [entryCount] = 1, entryCount++;

                        }

**if** (entryCount != 100){

**for** (**int** counter1 = 0; counter1 < 100; counter1++){

**if** (entryCount < 100) secondEventBDList [entryCount] = 1, entryCount++;

                            }

                        }

                        //====Determine % of each event C====

**int** totalNoOfAfterBDCF = (**int**)(simProcessDataBaseHold [8]+simProcessDataBaseHold [9])+totalNoOfBDCFCD;

**int** percentBDCFBD = 0; //7

**int** percentBDCFTD = 0; //8

**int** percentBDCFCD = 0; //9

**if** (totalNoOfAfterBDCF != 0) percentBDCFBD = (**int**)(round((simProcessDataBaseHold [8]/(**double**)totalNoOfAfterBDCF)*100));

**if** (totalNoOfAfterBDCF != 0) percentBDCFTD = (**int**)(round((simProcessDataBaseHold [9]/(**double**)totalNoOfAfterBDCF)*100));

**if** (totalNoOfAfterBDCF != 0) percentBDCFCD = (**int**)(round((totalNoOfBDCFCD/(**double**)totalNoOfAfterBDCF)*100));

**if** (totalNoOfAfterBDCF != 0){

**if** ((simProcessDataBaseHold [8]/(**double**)totalNoOfAfterBDCF)*100 != 0 && percentBDCFBD == 0){

                                percentBDCFBD = 1;

**if** (percentBDCFTD > 2) percentBDCFTD--;

**else** **if** (percentBDCFCD > 2) percentBDCFCD--;

                            }

**if** ((simProcessDataBaseHold [9]/(**double**)totalNoOfAfterBDCF)*100 != 0 && percentBDCFTD == 0){

                                percentBDCFTD = 1;

**if** (percentBDCFBD > 2) percentBDCFBD--;

**else** **if** (percentBDCFCD > 2) percentBDCFCD--;

                            }

**if** ((totalNoOfBDCFCD/(**double**)totalNoOfAfterBDCF)*100 != 0 && percentBDCFCD == 0){

                                percentBDCFCD = 1;

**if** (percentBDCFBD > 2) percentBDCFBD--;

**else** **if** (percentBDCFTD > 2) percentBDCFTD--;

                            }

                        }

                        entryCount = 0;

**int** lastEntry = 0;

**for** (**int** counter1 = 0; counter1 < percentBDCFBD; counter1++){

**if** (entryCount < 100){

                                secondEventBDCFList [entryCount] = 7, entryCount++;

                                lastEntry = 7;

                            }

                        }

**for** (**int** counter1 = 0; counter1 < percentBDCFTD; counter1++){

**if** (entryCount < 100){

                                secondEventBDCFList [entryCount] = 8, entryCount++;

                                lastEntry = 8;

                            }

                        }

**for** (**int** counter1 = 0; counter1 < percentBDCFCD; counter1++){

**if** (entryCount < 100){

                                secondEventBDCFList [entryCount] = 9, entryCount++;

                                lastEntry = 9;

                            }

                        }

**if** (entryCount != 0 && entryCount != 100){

**for** (**int** counter1 = 0; counter1 < 100; counter1++){

**if** (entryCount < 100) secondEventBDCFList [entryCount] = lastEntry, entryCount++;

                            }

                        }

                        //====Determine % of each event D====

**int** totalNoOfAfterTD = (**int**)(simProcessDataBaseHold [15]+simProcessDataBaseHold [16])+totalNoOfTDCD+totalNoOfTDCF;

**int** percentTDCF = 0; //10

**int** percentTDBD = 0; //11

**int** percentTDTD = 0; //12

**int** percentTDCD = 0; //13

**if** (totalNoOfAfterTD != 0) percentTDBD = (**int**)(round((simProcessDataBaseHold [15]/(**double**)totalNoOfAfterTD)*100));

**if** (totalNoOfAfterTD != 0) percentTDTD = (**int**)(round((simProcessDataBaseHold [16]/(**double**)totalNoOfAfterTD)*100));

**if** (totalNoOfAfterTD != 0) percentTDCD = (**int**)(round((totalNoOfTDCD/(**double**)totalNoOfAfterTD)*100));

**if** (totalNoOfAfterTD != 0) percentTDCF = (**int**)(round((totalNoOfTDCF/(**double**)totalNoOfAfterTD)*100));

**if** (totalNoOfAfterTD != 0){

**if** ((simProcessDataBaseHold [15]/(**double**)totalNoOfAfterTD)*100 != 0 && percentTDBD == 0){

                                percentTDBD = 1;

**if** (percentTDTD > 2) percentTDTD--;

**else** **if** (percentTDCD > 2) percentTDCD--;

**else** **if** (percentTDCF > 2) percentTDCF--;

                            }

**if** ((simProcessDataBaseHold [16]/(**double**)totalNoOfAfterTD)*100 != 0 && percentTDTD == 0){

                                percentTDTD = 1;

**if** (percentTDBD > 2) percentTDBD--;

**else** **if** (percentTDCD > 2) percentTDCD--;

**else** **if** (percentTDCF > 2) percentTDCF--;

                            }

**if** ((totalNoOfTDCD/(**double**)totalNoOfAfterTD)*100 != 0 && percentTDCD == 0){

                                percentTDCD = 1;

**if** (percentTDBD > 2) percentTDBD--;

**else** **if** (percentTDTD > 2) percentTDTD--;

**else** **if** (percentTDCF > 2) percentTDCF--;

                            }

**if** ((totalNoOfTDCF/(**double**)totalNoOfAfterTD)*100 != 0 && percentTDCF == 0){

                                percentTDCF = 1;

**if** (percentTDBD > 2) percentTDBD--;

**else** **if** (percentTDTD > 2) percentTDTD--;

**else** **if** (percentTDCD > 2) percentTDCD--;

                            }

                        }

                        entryCount = 0;

                        lastEntry = 0;

**for** (**int** counter1 = 0; counter1 < percentTDCF; counter1++){

**if** (entryCount < 100){

                                secondEventTDList [entryCount] = 10, entryCount++;

                                lastEntry = 10;

                            }

                        }

**for** (**int** counter1 = 0; counter1 < percentTDBD; counter1++){

**if** (entryCount < 100){

                                secondEventTDList [entryCount] = 11, entryCount++;

                                lastEntry = 11;

                            }

                        }

**for** (**int** counter1 = 0; counter1 < percentTDTD; counter1++){

**if** (entryCount < 100){

                                secondEventTDList [entryCount] = 12, entryCount++;

                                lastEntry = 12;

                            }

                        }

**for** (**int** counter1 = 0; counter1 < percentTDCD; counter1++){

**if** (entryCount < 100){

                                secondEventTDList [entryCount] = 13, entryCount++;

                                lastEntry = 13;

                            }

                        }

**if** (entryCount != 0 && entryCount != 100){

**for** (**int** counter1 = 0; counter1 < 100; counter1++){

**if** (entryCount < 100) secondEventTDList [entryCount] = lastEntry, entryCount++;

                            }

                        }

**int** totalNoOfAfterTDCF = (**int**)(simProcessDataBaseHold [12]+simProcessDataBaseHold [13])+totalNoOfTDCFCD;

**int** percentTDCFBD = 0; //14

**int** percentTDCFTD = 0; //15

**int** percentTDCFCD = 0; //16

**if** (totalNoOfAfterTDCF != 0) percentTDCFBD = (**int**)(round((simProcessDataBaseHold [12]/(**double**)totalNoOfAfterTDCF)*100));

**if** (totalNoOfAfterTDCF != 0) percentTDCFTD = (**int**)(round((simProcessDataBaseHold [13]/(**double**)totalNoOfAfterTDCF)*100));

**if** (totalNoOfAfterTDCF != 0) percentTDCFCD = (**int**)(round((totalNoOfTDCFCD/(**double**)totalNoOfAfterTDCF)*100));

**if** (totalNoOfAfterTDCF != 0){

**if** ((simProcessDataBaseHold [12]/(**double**)totalNoOfAfterTDCF)*100 != 0 && percentTDCFBD == 0){

                                percentTDCFBD = 1;

**if** (percentTDCFTD > 2) percentTDCFTD--;

**else** **if** (percentTDCFCD > 2) percentTDCFCD--;

                            }

**if** ((simProcessDataBaseHold [13]/(**double**)totalNoOfAfterTDCF)*100 != 0&& percentTDCFTD == 0){

                                percentTDCFTD = 1;

**if** (percentTDCFBD > 2) percentTDCFBD--;

**else** **if** (percentTDCFCD > 2) percentTDCFCD--;

                            }

**if** ((totalNoOfTDCFCD/(**double**)totalNoOfAfterTDCF)*100 != 0 && percentTDCFCD == 0){

                                percentTDCFCD = 1;

**if** (percentTDCFBD > 2) percentTDCFBD--;

**else** **if** (percentTDCFTD > 2) percentTDCFTD--;

                            }

                        }

                        entryCount = 0;

                        lastEntry = 0;

**for** (**int** counter1 = 0; counter1 < percentTDCFBD; counter1++){

**if** (entryCount < 100){

                                secondEventTDCFList [entryCount] = 14, entryCount++;

                                lastEntry = 14;

                            }

                        }

**for** (**int** counter1 = 0; counter1 < percentTDCFTD; counter1++){

**if** (entryCount < 100){

                                secondEventTDCFList [entryCount] = 15, entryCount++;

                                lastEntry = 15;

                            }

                        }

**for** (**int** counter1 = 0; counter1 < percentTDCFCD; counter1++){

**if** (entryCount < 100){

                                secondEventTDCFList [entryCount] = 16, entryCount++;

                                lastEntry = 16;

                            }

                        }

**if** (entryCount != 0 && entryCount != 100){

**for** (**int** counter1 = 0; counter1 < 100; counter1++){

**if** (entryCount < 100) secondEventTDCFList [entryCount] = lastEntry, entryCount++;

                            }

                        }

                        //======Calculate the +- 25% of the average time between BD, or BD to CD====

                        randBDRangeA = (**int**)(round(simProcessDataBaseHold [26]-simProcessDataBaseHold [26]*0.25));

                        randBDRangeB = (**int**)(round(simProcessDataBaseHold [26]+simProcessDataBaseHold [26]*0.25));

                        randCDRangeA = (**int**)(round(simProcessDataBaseHold [27]-simProcessDataBaseHold [27]*0.25));

                        randCDRangeB = (**int**)(round(simProcessDataBaseHold [27]+simProcessDataBaseHold [27]*0.25));

**if** (randBDRangeA == 0 && randBDRangeB == 0){

                            randBDRangeA = (**int**)(round(simProcessDataBaseHold [25]*0.8-simProcessDataBaseHold [25]*0.8*0.25));

                            randBDRangeB = (**int**)(round(simProcessDataBaseHold [25]*0.8));

                        }

**if** (randCDRangeA == 0 && randCDRangeB == 0){

                            randCDRangeA = (**int**)(round(simProcessDataBaseHold [25]*0.5-simProcessDataBaseHold [25]*0.5*0.25));

                            randCDRangeB = (**int**)(round(simProcessDataBaseHold [25]*0.5));

                        }

**if** (doseSimStatusHold == 1){

                            /*

                             doseSimStatusHold == 1 is Ind mode, which requires to re-define simulation arrays by calculating them from Source data, e.g. Source data 1 and 2.

                             In this case, the above-defined simulation arrays will be replaced by ones determined by the following function.

                             */

                            [**self** arraysRedetermine];

                        }

**else** **if** (doseSimStatusHold == 2){

                            /*

                             doseSimStatusHold == 2 is MX or MSC mode, which requires performing simulation using two sets of simulation assays. The following function allows creating the second set of simulation arrays.

                             */

                            [**self** secondArraySet];

                        }

**int** randInit = 0;

**int** randInit2 = 0;

**int** selectCheck = 0;

                        //**********First data set******

                        /*

                         The system has two modes to start with the simulation, i.e. synchronize or random. In synchronized mode, the code determines the first cell doubling time (first cell division to the next) and uses the data array that holds the assigned doubling time of the first cells. In the random mode, the time length to the first events (BD, TD, CD, or CF) will be determined and arrays containing the time length will be used to assigning the time to an event of the first cells. In the current paper, we used the random mode. The assigned length of time to the first event is stored in the activeCellStatusList.

                         */

**if** (simStartModeHold == 0){

**for** (**int** counter1 = 0; counter1 < growthInitHold; counter1++){

**if** (activeCellStatusListCount+50 > activeCellStatusListLimitHold){ //====Expand array size if nesseary====

**int** *arrayUpDate = **new** **int** [activeCellStatusListCount+10];

**for** (**int** counter2 = 0; counter2 < activeCellStatusListCount; counter2++) arrayUpDate [counter2] = activeCellStatusList [counter2];

**delete** [] activeCellStatusList;

                                    activeCellStatusList = **new** **int** [activeCellStatusListLimitHold+10000];

                                    activeCellStatusListLimitHold = activeCellStatusListLimitHold+10000;

**for** (**int** counter2 = 0; counter2 < activeCellStatusListCount; counter2++) activeCellStatusList [counter2] = arrayUpDate [counter2];

**delete** [] arrayUpDate;

                                }

                                activeCellStatusList [activeCellStatusListCount] = 0, activeCellStatusListCount++;

                                activeCellStatusList [activeCellStatusListCount] = 0, activeCellStatusListCount++;

                                activeCellStatusList [activeCellStatusListCount] = 0, activeCellStatusListCount++;

                                activeCellStatusList [activeCellStatusListCount] = counter1+1, activeCellStatusListCount++;

                                selectCheck = 0;

**if** (doseSimStatusHold == 2){

**for** (**int** counter2 = 0; counter2 < lingNoAssigineSimCount; counter2++){

**if** (lingNoAssigineSim [counter2] == counter1+1){

                                            selectCheck = 1;

**break**;

                                        }

                                    }

                                }

**if** (selectCheck == 0){

**if** (expandFirsDVListCount == 0){

                                        randInit = rand() % randBDRangeB + randBDRangeA;

                                        activeCellStatusList [activeCellStatusListCount] = randInit, activeCellStatusListCount++;

                                        activeCellStatusList [activeCellStatusListCount] = 7, activeCellStatusListCount++;

                                        activeCellStatusList [activeCellStatusListCount] = 1, activeCellStatusListCount++;

                                        activeCellStatusList [activeCellStatusListCount] = 0, activeCellStatusListCount++;

                                        activeCellStatusList [activeCellStatusListCount] = 0, activeCellStatusListCount++;

                                        activeCellStatusList [activeCellStatusListCount] = 0, activeCellStatusListCount++;

                                        activeCellStatusList [activeCellStatusListCount] = 0, activeCellStatusListCount++;

                                        activeCellStatusList [activeCellStatusListCount] = 0, activeCellStatusListCount++;

                                    }

**else**{

                                        randInit = rand() % expandFirsDVListCount/2 + 0; //====Obtain a random number to assign the length of time to the next event====

**if** (expandFirsDVList [randInit*2] == simProcessDataBaseHold [25]){

                                            activeCellStatusList [activeCellStatusListCount] = expandFirsDVList [randInit*2], activeCellStatusListCount++;

                                            randInit2 = rand() % (**int**)(round(simProcessDataBaseHold [25]*(endVariationtHold/(**double**)100))) + 0;

**if** (randInit%2 == 0) activeCellStatusList [activeCellStatusListCount-1] = activeCellStatusList [activeCellStatusListCount-1]+randInit2;

**else** activeCellStatusList [activeCellStatusListCount-1] = activeCellStatusList [activeCellStatusListCount-1]-randInit2;

                                        }

**else** activeCellStatusList [activeCellStatusListCount] = expandFirsDVList [randInit*2], activeCellStatusListCount++;

                                        randInit = rand() % 100 + 0;

**if** (firstEventList [randInit] == 0) activeCellStatusList [activeCellStatusListCount] = 4, activeCellStatusListCount++;

**else** activeCellStatusList [activeCellStatusListCount] = firstEventList [randInit], activeCellStatusListCount++;

**if** (firstEventList [randInit] == 4){

                                            randInit2 = rand() % (**int**)(round(simProcessDataBaseHold [25]*(endVariationtHold/(**double**)100))) + 0;

**if** (randInit2%2 == 0) activeCellStatusList [activeCellStatusListCount-2] = (**int**)simProcessDataBaseHold [25]+randInit2;

**else** activeCellStatusList [activeCellStatusListCount-2] = (**int**)simProcessDataBaseHold [25]-randInit2;

                                        }

**if** (firstEventList [randInit] == 3 && totalNoOfNonDivCD != 0){

                                            randInit2 = rand() % totalNoOfNonDivCD/2 + 0;

                                            activeCellStatusList [activeCellStatusListCount-2] = expandNonCD [randInit2*2];

                                        }

                                        activeCellStatusList [activeCellStatusListCount] = 0, activeCellStatusListCount++;

**if** (firstEventList [randInit] == 2){

                                            activeCellStatusList [activeCellStatusListCount] = 1, activeCellStatusListCount++;

                                            activeCellStatusList [activeCellStatusListCount] = (**int**)simProcessDataBaseHold [18], activeCellStatusListCount++;

                                        }

**else**{

                                            activeCellStatusList [activeCellStatusListCount] = 0, activeCellStatusListCount++;

                                            activeCellStatusList [activeCellStatusListCount] = 0, activeCellStatusListCount++;

                                        }

                                        activeCellStatusList [activeCellStatusListCount] = 0, activeCellStatusListCount++;

                                        activeCellStatusList [activeCellStatusListCount] = 0, activeCellStatusListCount++;

                                        activeCellStatusList [activeCellStatusListCount] = 0, activeCellStatusListCount++;

                                    }

                                }

**else**{ //====The following process is the same as above, but the arrays used here are for mixed cells=====

**if** (expandFirsDVListSelCount == 0){

                                        randInit = rand() % randBDRangeSelB + randBDRangeSelA;

                                        activeCellStatusList [activeCellStatusListCount] = randInit, activeCellStatusListCount++;

                                        activeCellStatusList [activeCellStatusListCount] = 7, activeCellStatusListCount++;

                                        activeCellStatusList [activeCellStatusListCount] = 1, activeCellStatusListCount++;

                                        activeCellStatusList [activeCellStatusListCount] = 0, activeCellStatusListCount++;

                                        activeCellStatusList [activeCellStatusListCount] = 0, activeCellStatusListCount++;

                                        activeCellStatusList [activeCellStatusListCount] = 0, activeCellStatusListCount++;

                                        activeCellStatusList [activeCellStatusListCount] = 0, activeCellStatusListCount++;

                                        activeCellStatusList [activeCellStatusListCount] = 0, activeCellStatusListCount++;

                                    }

**else**{

                                        randInit = rand() % expandFirsDVListSelCount/2 + 0;

**if** (expandFirsDVListSel [randInit*2] == simProcessDataProgHold [25]){

                                            activeCellStatusList [activeCellStatusListCount] = expandFirsDVListSel [randInit*2], activeCellStatusListCount++;

                                            randInit2 = rand() % (**int**)(round(simProcessDataProgHold [25]*(endVariationtHold/(**double**)100))) + 0;

**if** (randInit2%2 == 0) activeCellStatusList [activeCellStatusListCount-1] = activeCellStatusList [activeCellStatusListCount-1]+randInit2;

**else** activeCellStatusList [activeCellStatusListCount-1] = activeCellStatusList [activeCellStatusListCount-1]-randInit2;

                                        }

**else** activeCellStatusList [activeCellStatusListCount] = expandFirsDVListSel [randInit*2], activeCellStatusListCount++;

                                        randInit = rand() % 100 + 0;

**if** (firstEventListSel [randInit] == 0) activeCellStatusList [activeCellStatusListCount] = 4, activeCellStatusListCount++;

**else** activeCellStatusList [activeCellStatusListCount] = firstEventListSel [randInit], activeCellStatusListCount++;

**if** (firstEventListSel [randInit] == 4){

                                            randInit2 = rand() % (**int**)(round(simProcessDataProgHold [25]*(endVariationtHold/(**double**)100))) + 0;

**if** (randInit2%2 == 0) activeCellStatusList [activeCellStatusListCount-2] = (**int**)simProcessDataProgHold [25]+randInit2;

**else** activeCellStatusList [activeCellStatusListCount-2] = (**int**)simProcessDataProgHold [25]-randInit2;

                                        }

**if** (firstEventListSel [randInit] == 3 && totalNoOfNonDivCDSel != 0){

                                            randInit2 = rand() % totalNoOfNonDivCDSel/2 + 0;

                                            activeCellStatusList [activeCellStatusListCount-2] = expandNonCDSel [randInit2*2];

                                        }

                                        activeCellStatusList [activeCellStatusListCount] = 0, activeCellStatusListCount++;

**if** (firstEventListSel [randInit] == 2){

                                            activeCellStatusList [activeCellStatusListCount] = 1, activeCellStatusListCount++;

                                            activeCellStatusList [activeCellStatusListCount] = (**int**)simProcessDataProgHold [18], activeCellStatusListCount++;

                                        }

**else**{

                                            activeCellStatusList [activeCellStatusListCount] = 0, activeCellStatusListCount++;

                                            activeCellStatusList [activeCellStatusListCount] = 0, activeCellStatusListCount++;

                                        }

                                        activeCellStatusList [activeCellStatusListCount] = 0, activeCellStatusListCount++;

                                        activeCellStatusList [activeCellStatusListCount] = 0, activeCellStatusListCount++;

                                        activeCellStatusList [activeCellStatusListCount] = 0, activeCellStatusListCount++;

                                    }

                                }

                            }

                        }

**else**{

**for** (**int** counter1 = 0; counter1 < growthInitHold; counter1++){

**if** (activeCellStatusListCount+50 > activeCellStatusListLimitHold){

**int** *arrayUpDate = **new** **int** [activeCellStatusListCount+10];

**for** (**int** counter2 = 0; counter2 < activeCellStatusListCount; counter2++) arrayUpDate [counter2] = activeCellStatusList [counter2];

**delete** [] activeCellStatusList;

                                    activeCellStatusList = **new** **int** [activeCellStatusListLimitHold+10000];

                                    activeCellStatusListLimitHold = activeCellStatusListLimitHold+10000;

**for** (**int** counter2 = 0; counter2 < activeCellStatusListCount; counter2++) activeCellStatusList [counter2] = arrayUpDate [counter2];

**delete** [] arrayUpDate;

                                }

                                activeCellStatusList [activeCellStatusListCount] = 0, activeCellStatusListCount++;

                                activeCellStatusList [activeCellStatusListCount] = 0, activeCellStatusListCount++;

                                activeCellStatusList [activeCellStatusListCount] = 0, activeCellStatusListCount++;

                                activeCellStatusList [activeCellStatusListCount] = counter1+1, activeCellStatusListCount++;

                                selectCheck = 0;

**if** (doseSimStatusHold == 2){

**for** (**int** counter2 = 0; counter2 < lingNoAssigineSimCount; counter2++){

**if** (lingNoAssigineSim [counter2] == counter1+1){

                                            selectCheck = 1;

**break**;

                                        }

                                    }

                                }

**if** (selectCheck == 0){

**if** (expandDoublingDoubBDCount == 0){

                                        randInit = rand() % randBDRangeB + randBDRangeA;

                                        activeCellStatusList [activeCellStatusListCount] = randInit, activeCellStatusListCount++;

                                        activeCellStatusList [activeCellStatusListCount] = 7, activeCellStatusListCount++;

                                        activeCellStatusList [activeCellStatusListCount] = 1, activeCellStatusListCount++;

                                        activeCellStatusList [activeCellStatusListCount] = 0, activeCellStatusListCount++;

                                        activeCellStatusList [activeCellStatusListCount] = 0, activeCellStatusListCount++;

                                        activeCellStatusList [activeCellStatusListCount] = 0, activeCellStatusListCount++;

                                        activeCellStatusList [activeCellStatusListCount] = 0, activeCellStatusListCount++;

                                        activeCellStatusList [activeCellStatusListCount] = 0, activeCellStatusListCount++;

                                    }

**else**{

                                        randInit = rand() % expandDoublingDoubBDCount/2 + 0;

**if** (expandDoublingDoubBD [randInit*2] == simProcessDataBaseHold [25]){

                                            activeCellStatusList [activeCellStatusListCount] = expandDoublingDoubBD [randInit*2], activeCellStatusListCount++;

                                            randInit2 = rand() % (**int**)(round(simProcessDataBaseHold [25]*0.30)) + 0;

**if** (randInit2%2 == 0) activeCellStatusList [activeCellStatusListCount-1] = activeCellStatusList [activeCellStatusListCount-1]+randInit2;

**else** activeCellStatusList [activeCellStatusListCount-1] = activeCellStatusList [activeCellStatusListCount-1]-randInit2;

                                        }

**else** activeCellStatusList [activeCellStatusListCount] = expandFirsDVList [randInit*2], activeCellStatusListCount++;

                                        randInit = rand() % 100 + 0;

**if** (firstEventList [randInit] == 0) activeCellStatusList [activeCellStatusListCount] = 4, activeCellStatusListCount++;

**else** activeCellStatusList [activeCellStatusListCount] = firstEventList [randInit], activeCellStatusListCount++;

**if** (firstEventList [randInit] == 4){

                                            randInit2 = rand() % (**int**)(round(simProcessDataBaseHold [25]*(endVariationtHold/(**double**)100))) + 0;

**if** (randInit2%2 == 0) activeCellStatusList [activeCellStatusListCount-2] = (**int**)simProcessDataBaseHold [25]+randInit2;

**else** activeCellStatusList [activeCellStatusListCount-2] = (**int**)simProcessDataBaseHold [25]-randInit2;

                                        }

**if** (firstEventList [randInit] == 3 && totalNoOfNonDivCD != 0){

                                            randInit2 = rand() % totalNoOfNonDivCD/2 + 0;

                                            activeCellStatusList [activeCellStatusListCount-2] = expandNonCD [randInit2*2];

                                        }

                                        activeCellStatusList [activeCellStatusListCount] = 0, activeCellStatusListCount++;

**if** (firstEventList [randInit] == 2){

                                            activeCellStatusList [activeCellStatusListCount] = 1, activeCellStatusListCount++;

                                            activeCellStatusList [activeCellStatusListCount] = (**int**)simProcessDataBaseHold [18], activeCellStatusListCount++;

                                        }

**else**{

                                            activeCellStatusList [activeCellStatusListCount] = 0, activeCellStatusListCount++;

                                            activeCellStatusList [activeCellStatusListCount] = 0, activeCellStatusListCount++;

                                        }

                                        activeCellStatusList [activeCellStatusListCount] = 0, activeCellStatusListCount++;

                                        activeCellStatusList [activeCellStatusListCount] = 0, activeCellStatusListCount++;

                                        activeCellStatusList [activeCellStatusListCount] = 0, activeCellStatusListCount++;

                                    }

                                }

**else**{ //====The following process is the same as above, but the arrays used here are for mixed cells=====

**if** (expandDoublingDoubBDSelCount == 0){

                                        randInit = rand() % randBDRangeSelB + randBDRangeSelA;

                                        activeCellStatusList [activeCellStatusListCount] = randInit, activeCellStatusListCount++;

                                        activeCellStatusList [activeCellStatusListCount] = 7, activeCellStatusListCount++;

                                        activeCellStatusList [activeCellStatusListCount] = 1, activeCellStatusListCount++;

                                        activeCellStatusList [activeCellStatusListCount] = 0, activeCellStatusListCount++;

                                        activeCellStatusList [activeCellStatusListCount] = 0, activeCellStatusListCount++;

                                        activeCellStatusList [activeCellStatusListCount] = 0, activeCellStatusListCount++;

                                        activeCellStatusList [activeCellStatusListCount] = 0, activeCellStatusListCount++;

                                        activeCellStatusList [activeCellStatusListCount] = 0, activeCellStatusListCount++;

                                    }

**else**{

                                        randInit = rand() % expandDoublingDoubBDSelCount/2 + 0;

**if** (expandDoublingDoubBDSel [randInit*2] == simProcessDataProgHold [25]){

                                            activeCellStatusList [activeCellStatusListCount] = expandDoublingDoubBDSel [randInit*2], activeCellStatusListCount++;

                                            randInit2 = rand() % (**int**)(round(simProcessDataProgHold [25]*0.30)) + 0;

**if** (randInit2%2 == 0) activeCellStatusList [activeCellStatusListCount-1] = activeCellStatusList [activeCellStatusListCount-1]+randInit2;

**else** activeCellStatusList [activeCellStatusListCount-1] = activeCellStatusList [activeCellStatusListCount-1]-randInit2;

                                        }

**else** activeCellStatusList [activeCellStatusListCount] = expandFirsDVListSel [randInit*2], activeCellStatusListCount++;

                                        randInit = rand() % 100 + 0;

**if** (firstEventListSel [randInit] == 0) activeCellStatusList [activeCellStatusListCount] = 4, activeCellStatusListCount++;

**else** activeCellStatusList [activeCellStatusListCount] = firstEventListSel [randInit], activeCellStatusListCount++;

**if** (firstEventListSel [randInit] == 4){

                                            randInit2 = rand() % (**int**)(round(simProcessDataProgHold [25]*(endVariationtHold/(**double**)100))) + 0;

**if** (randInit2%2 == 0) activeCellStatusList [activeCellStatusListCount-2] = (**int**)simProcessDataProgHold [25]+randInit2;

**else** activeCellStatusList [activeCellStatusListCount-2] = (**int**)simProcessDataProgHold [25]-randInit2;

                                        }

**if** (firstEventListSel [randInit] == 3 && totalNoOfNonDivCDSel != 0){

                                            randInit2 = rand() % totalNoOfNonDivCDSel/2 + 0;

                                            activeCellStatusList [activeCellStatusListCount-2] = expandNonCDSel [randInit2*2];

                                        }

                                        activeCellStatusList [activeCellStatusListCount] = 0, activeCellStatusListCount++;

**if** (firstEventListSel [randInit] == 2){

                                            activeCellStatusList [activeCellStatusListCount] = 1, activeCellStatusListCount++;

                                            activeCellStatusList [activeCellStatusListCount] = (**int**)simProcessDataProgHold [18], activeCellStatusListCount++;

                                        }

**else**{

                                            activeCellStatusList [activeCellStatusListCount] = 0, activeCellStatusListCount++;

                                            activeCellStatusList [activeCellStatusListCount] = 0, activeCellStatusListCount++;

                                        }

                                        activeCellStatusList [activeCellStatusListCount] = 0, activeCellStatusListCount++;

                                        activeCellStatusList [activeCellStatusListCount] = 0, activeCellStatusListCount++;

                                        activeCellStatusList [activeCellStatusListCount] = 0, activeCellStatusListCount++;

                                    }

                                }

                            }

                        }

**delete** [] activeCellStatusListHold;

                        activeCellStatusListHold = **new** **int** [activeCellStatusListCount+10];

                        activeCellStatusListHoldCount = 0;

                        //====Set activeCellStatusListHold array====

**for** (**int** counter1 = 0; counter1 < activeCellStatusListCount; counter1++) activeCellStatusListHold [activeCellStatusListHoldCount] = activeCellStatusList [counter1], activeCellStatusListHoldCount++;

**int** nonDivAssign = 0;

**int** otherDivAssign = 0;

**for** (**int** counterA = 0; counterA < activeCellStatusListCount/12; counterA++){

**if** (activeCellStatusList [counterA*12+5] == 5 || activeCellStatusList [counterA*12+5] == 4){

                                nonDivAssign++;

                            }

**else** otherDivAssign++;

                        }

                        //====For display information====

                        processingStatusCall3 = 1;

                        processingStatus3 = to_string (nonDivAssign)+"/"+to_string (otherDivAssign);

**if** (growthCycleMid == 0){

                            processingStatusCall4 = 1;

                            processingStatus4 = "nil";

                        }

**int** extendEnd = 0;

**int** newCellNumber1 = 0;

**int** newCellNumber2 = 0;

**int** newCellNumber3 = 0;

**int** *tempListOfCells = **new** **int** [cellLineageTempArrayCount+10];

**unsigned** **long** tempListOfCellsCount = 0;

**unsigned** **long** tempListOfCellsLimit = cellLineageTempArrayCount+10;

                        cellNoLingNoList = **new** **int** [600000];

                        cellNoLingNoListCount = 0;

                        cellNoLingNoListLimit = 600000;

**for** (**int** counter1 = 0; counter1 < cellLineageTempArrayCount+10; counter1++) tempListOfCells [counter1] = 0;

                        /*

                         ******tempListOfCells******

                         1. Lineage no

                         2. Parent cell No

                         3. Cell No 1

                         4. Cell No 2

                         5. Cell No 3

                         6. Event type

                         7. Dubling time

                         ******cellNoLingNoList******

                         1. Lineage no

                         2. Cell no

                         */

                        //**********First Cell lineage data creation******

                        /*

                         Cell lineage data will be created based on data stored in the "activeCellStatusList". Lineage data will be stored in the "cellLineageTempArray".

                         */

**unsigned** **long** entryStart = 0;

                        string cellNumberExtract;

                        string lineageNumberExtract;

                        string newExtensionEntryNo;

                        string degitExtract;

**for** (**int** counter1 = 0; counter1 < activeCellStatusListCount/12; counter1++){

**if** (activeCellStatusList [counter1*12+4] > growthCycleBase) extendEnd = growthCycleBase;

**else** extendEnd = (**int**)activeCellStatusList [counter1*12+4];

**if** (cellNoLingNoListCount+10 > cellNoLingNoListLimit){ //====Array size management====

**int** *arrayUpDate = **new** **int** [cellNoLingNoListCount+10];

**for** (**int** counter3 = 0; counter3 < cellNoLingNoListCount; counter3++) arrayUpDate [counter3] = cellNoLingNoList [counter3];

**delete** [] cellNoLingNoList;

                                cellNoLingNoList = **new** **int** [cellNoLingNoListLimit+10000];

                                cellNoLingNoListLimit = cellNoLingNoListLimit+10000;

**for** (**int** counter3 = 0; counter3 < cellNoLingNoListCount; counter3++) cellNoLingNoList [counter3] = arrayUpDate [counter3];

**delete** [] arrayUpDate;

                            }

**if** (cellLineageSummaryArrayCount+50 > cellLineageSummaryArrayLimit){ //====Array size management====

**long** *arrayUpDate = **new** **long** [cellLineageSummaryArrayCount+10];

**for** (**unsigned** **long** counter3 = 0; counter3 < cellLineageSummaryArrayCount; counter3++) arrayUpDate [counter3] = cellLineageSummaryArray [counter3];

**delete** [] cellLineageSummaryArray;

                                cellLineageSummaryArray = **new** **long** [cellLineageSummaryArrayLimit+100000000];

                                cellLineageSummaryArrayLimit = cellLineageSummaryArrayLimit+100000000;

**for** (**unsigned** **long** counter3 = 0; counter3 < cellLineageSummaryArrayCount; counter3++) cellLineageSummaryArray [counter3] = arrayUpDate [counter3];

**delete** [] arrayUpDate;

                            }

                            //====Enter cell lineage no. and cell no. to the "cellNoLingNoList"====

                            cellNoLingNoList [cellNoLingNoListCount] = activeCellStatusList [counter1*12+3], cellNoLingNoListCount++;

                            cellNoLingNoList [cellNoLingNoListCount] = activeCellStatusList [counter1*12], cellNoLingNoListCount++;

                            //====Write cell lineage data into the "cellLineageTempArray" till the time point where an event occurs. This time point is the last data entry of a cell that undergoes a simulation.====

                            //====Then, the "activeCellStatusListKeep" is updated and relevant data is entered into the "cellLineageSummaryArray".====

                            entryStart = cellLineageTempArrayCount;

**for** (**int** counter2 = 0; counter2 < extendEnd; counter2++){

**if** (cellLineageTempArrayCount+9 > cellLineageTempArrayLimit){ //====Array size management====

**int** *arrayUpDate = **new** **int** [cellLineageTempArrayCount+10];

**for** (**int** counter3 = 0; counter3 < cellLineageTempArrayCount; counter3++) arrayUpDate [counter3] = cellLineageTempArray [counter3];

**delete** [] cellLineageTempArray;

                                    cellLineageTempArray = **new** **int** [cellLineageTempArrayLimit+100000000000];

                                    cellLineageTempArrayLimit = cellLineageTempArrayLimit+100000000000;

**for** (**int** counter3 = 0; counter3 < cellLineageTempArrayCount; counter3++) cellLineageTempArray [counter3] = arrayUpDate [counter3];

**delete** [] arrayUpDate;

                                }

                                selectCheck = 0;

**if** (doseSimStatusHold == 2){

**for** (**int** counter3 = 0; counter3 < lingNoAssigineSimCount; counter3++){

**if** (lingNoAssigineSim [counter3] == counter1+1){

                                            selectCheck = 1;

**break**;

                                        }

                                    }

                                }

**if** (doseSimStatusHold == 2 && selectCheck == 1){

                                    cellLineageTempArray [cellLineageTempArrayCount] = 20, cellLineageTempArrayCount++;

                                    cellLineageTempArray [cellLineageTempArrayCount] = 20, cellLineageTempArrayCount++;

                                }

**else**{

                                    cellLineageTempArray [cellLineageTempArrayCount] = 1, cellLineageTempArrayCount++;

                                    cellLineageTempArray [cellLineageTempArrayCount] = 1, cellLineageTempArrayCount++;

                                }

                                cellLineageTempArray [cellLineageTempArrayCount] = counter2+1, cellLineageTempArrayCount++;

**if** (counter2 == 0) cellLineageTempArray [cellLineageTempArrayCount] = 1, cellLineageTempArrayCount++;

**else** **if** (counter2 != 0) cellLineageTempArray [cellLineageTempArrayCount] = 2, cellLineageTempArrayCount++;

                                cellLineageTempArray [cellLineageTempArrayCount] = 0, cellLineageTempArrayCount++;

                                cellLineageTempArray [cellLineageTempArrayCount] = activeCellStatusList [counter1*12], cellLineageTempArrayCount++;

                                cellLineageTempArray [cellLineageTempArrayCount] = activeCellStatusList [counter1*12+3], cellLineageTempArrayCount++;

                                cellLineageTempArray [cellLineageTempArrayCount] = 0, cellLineageTempArrayCount++;

                                cellLineageTempArray [cellLineageTempArrayCount] = 0, cellLineageTempArrayCount++;

**if** (counter2+1 == growthCycleBase){

                                    activeCellStatusListKeep [activeCellStatusListKeepCount] = activeCellStatusListHold [counter1*12], activeCellStatusListKeepCount++;

                                    activeCellStatusListKeep [activeCellStatusListKeepCount] = activeCellStatusListHold [counter1*12+1], activeCellStatusListKeepCount++;

                                    activeCellStatusListKeep [activeCellStatusListKeepCount] = activeCellStatusListHold [counter1*12+2], activeCellStatusListKeepCount++;

                                    activeCellStatusListKeep [activeCellStatusListKeepCount] = activeCellStatusListHold [counter1*12+3], activeCellStatusListKeepCount++;

                                    activeCellStatusListKeep [activeCellStatusListKeepCount] = activeCellStatusListHold [counter1*12+4], activeCellStatusListKeepCount++;

                                    activeCellStatusListKeep [activeCellStatusListKeepCount] = activeCellStatusListHold [counter1*12+5], activeCellStatusListKeepCount++;

                                    activeCellStatusListKeep [activeCellStatusListKeepCount] = activeCellStatusListHold [counter1*12+6], activeCellStatusListKeepCount++;

                                    activeCellStatusListKeep [activeCellStatusListKeepCount] = activeCellStatusListHold [counter1*12+7], activeCellStatusListKeepCount++;

                                    activeCellStatusListKeep [activeCellStatusListKeepCount] = activeCellStatusListHold [counter1*12+8], activeCellStatusListKeepCount++;

                                    activeCellStatusListKeep [activeCellStatusListKeepCount] = activeCellStatusListHold [counter1*12+9], activeCellStatusListKeepCount++;

                                    activeCellStatusListKeep [activeCellStatusListKeepCount] = activeCellStatusListHold [counter1*12+10], activeCellStatusListKeepCount++;

                                    activeCellStatusListKeep [activeCellStatusListKeepCount] = activeCellStatusListHold [counter1*12+11], activeCellStatusListKeepCount++;

                                    activeCellStatusList [counter1*12+1] = -1;

**break**;

                                }

                            }

                            cellLineageSummaryArray [cellLineageSummaryArrayCount] = (**long**)entryStart, cellLineageSummaryArrayCount++;

                            cellLineageSummaryArray [cellLineageSummaryArrayCount] = (**long**)cellLineageTempArrayCount-9, cellLineageSummaryArrayCount++;

                            cellLineageSummaryArray [cellLineageSummaryArrayCount] = (**long**)activeCellStatusList [counter1*12+3], cellLineageSummaryArrayCount++;

                            cellLineageSummaryArray [cellLineageSummaryArrayCount] = (**long**)activeCellStatusList [counter1*12], cellLineageSummaryArrayCount++;

                            cellLineageSummaryArray [cellLineageSummaryArrayCount] = 0, cellLineageSummaryArrayCount++;

                            cellLineageSummaryArray [cellLineageSummaryArrayCount] = 0, cellLineageSummaryArrayCount++;

                            cellLineageSummaryArray [cellLineageSummaryArrayCount] = 1, cellLineageSummaryArrayCount++;

                            cellLineageSummaryArray [cellLineageSummaryArrayCount] = 2, cellLineageSummaryArrayCount++;

                            cellLineageSummaryArray [cellLineageSummaryArrayCount] = 0, cellLineageSummaryArrayCount++;

**if** (cellLineageTempArrayCount+50 > cellLineageTempArrayLimit){ //====Array size management====

**int** *arrayUpDate = **new** **int** [cellLineageTempArrayCount+10];

**for** (**int** counter3 = 0; counter3 < cellLineageTempArrayCount; counter3++) arrayUpDate [counter3] = cellLineageTempArray [counter3];

**delete** [] cellLineageTempArray;

                                cellLineageTempArray = **new** **int** [cellLineageTempArrayLimit+100000000000];

                                cellLineageTempArrayLimit = cellLineageTempArrayLimit+100000000000;

**for** (**int** counter3 = 0; counter3 < cellLineageTempArrayCount; counter3++) cellLineageTempArray [counter3] = arrayUpDate [counter3];

**delete** [] arrayUpDate;

                            }

**if** (tempListOfCellsCount+6 > tempListOfCellsLimit){ //====Array size management====

**int** *arrayUpDate = **new** **int** [tempListOfCellsCount+10];

**for** (**int** counter2 = 0; counter2 < tempListOfCellsCount; counter2++) arrayUpDate [counter2] = tempListOfCells [counter2];

**delete** [] tempListOfCells;

                                tempListOfCells = **new** **int** [tempListOfCellsLimit+10000];

                                tempListOfCellsLimit = tempListOfCellsLimit+10000;

**for** (**int** counter2 = 0; counter2 < tempListOfCellsCount; counter2++) tempListOfCells [counter2] = arrayUpDate [counter2];

**delete** [] arrayUpDate;

                            }

**if** (activeCellStatusListKeepCount+100 > activeCellStatusListKeepLimit){ //====Array size management====

**int** *arrayUpDate = **new** **int** [activeCellStatusListKeepCount+50];

**for** (**int** counter3 = 0; counter3 < activeCellStatusListKeepCount; counter3++) arrayUpDate [counter3] = activeCellStatusListKeep [counter3];

**delete** [] activeCellStatusListKeep;

                                activeCellStatusListKeep = **new** **int** [activeCellStatusListKeepLimit+10000];

                                activeCellStatusListKeepLimit = activeCellStatusListKeepLimit+10000;

**for** (**int** counter3 = 0; counter3 < activeCellStatusListKeepCount; counter3++) activeCellStatusListKeep [counter3] = arrayUpDate [counter3];

**delete** [] arrayUpDate;

                            }

**if** (cellNoLingNoListCount+50 > cellNoLingNoListLimit){ //====Array size management====

**int** *arrayUpDate = **new** **int** [cellNoLingNoListCount+50];

**for** (**int** counter3 = 0; counter3 < cellNoLingNoListCount; counter3++) arrayUpDate [counter3] = cellNoLingNoList [counter3];

**delete** [] cellNoLingNoList;

                                cellNoLingNoList = **new** **int** [cellNoLingNoListLimit+10000];

                                cellNoLingNoListLimit = cellNoLingNoListLimit+10000;

**for** (**int** counter3 = 0; counter3 < cellNoLingNoListCount; counter3++) cellNoLingNoList [counter3] = arrayUpDate [counter3];

**delete** [] arrayUpDate;

                            }

                            //====If the next event is BD or TD, generate new cells (progenies). If the next event is CD, it will be marked as an end of cell status.======

**if** (activeCellStatusList [counter1*12+5] == 1){

**if** (extendEnd+3 <= growthCycleBase){

                                    cellLineageTempArray [cellLineageTempArrayCount] = 1, cellLineageTempArrayCount++;

                                    cellLineageTempArray [cellLineageTempArrayCount] = 1, cellLineageTempArrayCount++;

                                    cellLineageTempArray [cellLineageTempArrayCount] = extendEnd+1, cellLineageTempArrayCount++;

                                    cellLineageTempArray [cellLineageTempArrayCount] = 6, cellLineageTempArrayCount++;

                                    cellLineageTempArray [cellLineageTempArrayCount] = 0, cellLineageTempArrayCount++;

                                    cellLineageTempArray [cellLineageTempArrayCount] = activeCellStatusList [counter1*12], cellLineageTempArrayCount++;

                                    cellLineageTempArray [cellLineageTempArrayCount] = activeCellStatusList [counter1*12+3], cellLineageTempArrayCount++;

                                    cellLineageTempArray [cellLineageTempArrayCount] = 0, cellLineageTempArrayCount++;

                                    cellLineageTempArray [cellLineageTempArrayCount] = 0, cellLineageTempArrayCount++;

                                    cellLineageTempArray [cellLineageTempArrayCount] = 1, cellLineageTempArrayCount++;

                                    cellLineageTempArray [cellLineageTempArrayCount] = 1, cellLineageTempArrayCount++;

                                    cellLineageTempArray [cellLineageTempArrayCount] = extendEnd+2, cellLineageTempArrayCount++;

                                    cellLineageTempArray [cellLineageTempArrayCount] = 32, cellLineageTempArrayCount++;

                                    cellLineageTempArray [cellLineageTempArrayCount] = 0, cellLineageTempArrayCount++;

                                    cellLineageTempArray [cellLineageTempArrayCount] = activeCellStatusList [counter1*12], cellLineageTempArrayCount++;

                                    cellLineageTempArray [cellLineageTempArrayCount] = activeCellStatusList [counter1*12+3], cellLineageTempArrayCount++;

                                    cellLineageTempArray [cellLineageTempArrayCount] = 0, cellLineageTempArrayCount++;

                                    cellLineageTempArray [cellLineageTempArrayCount] = 0, cellLineageTempArrayCount++;

**for** (**unsigned** **long** counter2 = 0; counter2 < cellLineageSummaryArrayCount/9; counter2++){

**if** (cellLineageSummaryArray [counter2*9+2] == activeCellStatusList [counter1*12+3] && cellLineageSummaryArray [counter2*9+3] == activeCellStatusList [counter1*12]){

                                            cellLineageSummaryArray [counter2*9+1] = (**long**)(cellLineageTempArrayCount-9);

                                            cellLineageSummaryArray [counter2*9+7] = 32;

**break**;

                                        }

                                    }

                                    //========Assign cell no to generated progeny========

                                    createNewCellNo = [[CreateNewCellNo alloc] init];

                                    newCellNumber1 = [createNewCellNo cellNumberAddition:counter1];

                                    [CreateNewCellNo release];

                                    cellNoLingNoList [cellNoLingNoListCount] = activeCellStatusList [counter1*12+3], cellNoLingNoListCount++;

                                    cellNoLingNoList [cellNoLingNoListCount] = newCellNumber1, cellNoLingNoListCount++;

                                    createNewCellNo = [[CreateNewCellNo alloc] init];

                                    newCellNumber2 = [createNewCellNo cellNumberSubtraction:counter1];

                                    [CreateNewCellNo release];

                                    cellNoLingNoList [cellNoLingNoListCount] = activeCellStatusList [counter1*12+3], cellNoLingNoListCount++;

                                    cellNoLingNoList [cellNoLingNoListCount] = newCellNumber2, cellNoLingNoListCount++;

                                    cellLineageTempArray [cellLineageTempArrayCount] = 1, cellLineageTempArrayCount++;

                                    cellLineageTempArray [cellLineageTempArrayCount] = 1, cellLineageTempArrayCount++;

                                    cellLineageTempArray [cellLineageTempArrayCount] = extendEnd+3, cellLineageTempArrayCount++;

                                    cellLineageTempArray [cellLineageTempArrayCount] = 31, cellLineageTempArrayCount++;

                                    cellLineageTempArray [cellLineageTempArrayCount] = activeCellStatusList [counter1*12], cellLineageTempArrayCount++;

                                    cellLineageTempArray [cellLineageTempArrayCount] = newCellNumber1, cellLineageTempArrayCount++;

                                    cellLineageTempArray [cellLineageTempArrayCount] = activeCellStatusList [counter1*12+3], cellLineageTempArrayCount++;

                                    cellLineageTempArray [cellLineageTempArrayCount] = 0, cellLineageTempArrayCount++;

                                    cellLineageTempArray [cellLineageTempArrayCount] = 0, cellLineageTempArrayCount++;

                                    cellLineageTempArray [cellLineageTempArrayCount] = 1, cellLineageTempArrayCount++;

                                    cellLineageTempArray [cellLineageTempArrayCount] = 1, cellLineageTempArrayCount++;

                                    cellLineageTempArray [cellLineageTempArrayCount] = extendEnd+3, cellLineageTempArrayCount++;

                                    cellLineageTempArray [cellLineageTempArrayCount] = 31, cellLineageTempArrayCount++;

                                    cellLineageTempArray [cellLineageTempArrayCount] = activeCellStatusList [counter1*12], cellLineageTempArrayCount++;

                                    cellLineageTempArray [cellLineageTempArrayCount] = newCellNumber2, cellLineageTempArrayCount++;

                                    cellLineageTempArray [cellLineageTempArrayCount] = activeCellStatusList [counter1*12+3], cellLineageTempArrayCount++;

                                    cellLineageTempArray [cellLineageTempArrayCount] = 0, cellLineageTempArrayCount++;

                                    cellLineageTempArray [cellLineageTempArrayCount] = 0, cellLineageTempArrayCount++;

                                    cellLineageSummaryArray [cellLineageSummaryArrayCount] = (**long**)cellLineageTempArrayCount-18, cellLineageSummaryArrayCount++;

                                    cellLineageSummaryArray [cellLineageSummaryArrayCount] = (**long**)cellLineageTempArrayCount-18, cellLineageSummaryArrayCount++;

                                    cellLineageSummaryArray [cellLineageSummaryArrayCount] = (**long**)activeCellStatusList [counter1*12+3], cellLineageSummaryArrayCount++;

                                    cellLineageSummaryArray [cellLineageSummaryArrayCount] = newCellNumber1, cellLineageSummaryArrayCount++;

                                    cellLineageSummaryArray [cellLineageSummaryArrayCount] = (**long**)activeCellStatusList [counter1*12], cellLineageSummaryArrayCount++;

                                    cellLineageSummaryArray [cellLineageSummaryArrayCount] = (**long**)activeCellStatusList [counter1*12+3], cellLineageSummaryArrayCount++;

                                    cellLineageSummaryArray [cellLineageSummaryArrayCount] = 31, cellLineageSummaryArrayCount++;

                                    cellLineageSummaryArray [cellLineageSummaryArrayCount] = 2, cellLineageSummaryArrayCount++;

                                    cellLineageSummaryArray [cellLineageSummaryArrayCount] = 0, cellLineageSummaryArrayCount++;

                                    cellLineageSummaryArray [cellLineageSummaryArrayCount] = (**long**)cellLineageTempArrayCount-9, cellLineageSummaryArrayCount++;

                                    cellLineageSummaryArray [cellLineageSummaryArrayCount] = (**long**)cellLineageTempArrayCount-9, cellLineageSummaryArrayCount++;

                                    cellLineageSummaryArray [cellLineageSummaryArrayCount] = (**long**)activeCellStatusList [counter1*12+3], cellLineageSummaryArrayCount++;

                                    cellLineageSummaryArray [cellLineageSummaryArrayCount] = newCellNumber2, cellLineageSummaryArrayCount++;

                                    cellLineageSummaryArray [cellLineageSummaryArrayCount] = (**long**)activeCellStatusList [counter1*12], cellLineageSummaryArrayCount++;

                                    cellLineageSummaryArray [cellLineageSummaryArrayCount] = (**long**)activeCellStatusList [counter1*12+3], cellLineageSummaryArrayCount++;

                                    cellLineageSummaryArray [cellLineageSummaryArrayCount] = 31, cellLineageSummaryArrayCount++;

                                    cellLineageSummaryArray [cellLineageSummaryArrayCount] = 2, cellLineageSummaryArrayCount++;

                                    cellLineageSummaryArray [cellLineageSummaryArrayCount] = 0, cellLineageSummaryArrayCount++;

**if** (extendEnd+3 < growthCycleBase){

                                        tempListOfCells [tempListOfCellsCount] = activeCellStatusList [counter1*12+3], tempListOfCellsCount++;

                                        tempListOfCells [tempListOfCellsCount] = activeCellStatusList [counter1*12], tempListOfCellsCount++;

                                        tempListOfCells [tempListOfCellsCount] = newCellNumber1, tempListOfCellsCount++;

                                        tempListOfCells [tempListOfCellsCount] = newCellNumber2, tempListOfCellsCount++;

                                        tempListOfCells [tempListOfCellsCount] = 0, tempListOfCellsCount++;

                                        tempListOfCells [tempListOfCellsCount] = 31, tempListOfCellsCount++;

                                        tempListOfCells [tempListOfCellsCount] = activeCellStatusList [counter1*12+4], tempListOfCellsCount++;

                                    }

**else** **if** (extendEnd+3 == growthCycleBase){

                                        activeCellStatusListKeep [activeCellStatusListKeepCount] = newCellNumber1, activeCellStatusListKeepCount++;

                                        activeCellStatusListKeep [activeCellStatusListKeepCount] = newCellNumber2, activeCellStatusListKeepCount++;

                                        activeCellStatusListKeep [activeCellStatusListKeepCount] = 0, activeCellStatusListKeepCount++;

                                        activeCellStatusListKeep [activeCellStatusListKeepCount] = activeCellStatusList [counter1*12+3], activeCellStatusListKeepCount++;

                                        activeCellStatusListKeep [activeCellStatusListKeepCount] = 0, activeCellStatusListKeepCount++;

                                        activeCellStatusListKeep [activeCellStatusListKeepCount] = 1, activeCellStatusListKeepCount++;

                                        activeCellStatusListKeep [activeCellStatusListKeepCount] = 0, activeCellStatusListKeepCount++;

                                        activeCellStatusListKeep [activeCellStatusListKeepCount] = activeCellStatusList [counter1*12+7], activeCellStatusListKeepCount++;

                                        activeCellStatusListKeep [activeCellStatusListKeepCount] = activeCellStatusList [counter1*12+8], activeCellStatusListKeepCount++;

                                        activeCellStatusListKeep [activeCellStatusListKeepCount] = activeCellStatusList [counter1*12+9], activeCellStatusListKeepCount++;

                                        activeCellStatusListKeep [activeCellStatusListKeepCount] = activeCellStatusList [counter1*12], activeCellStatusListKeepCount++;

                                        activeCellStatusListKeep [activeCellStatusListKeepCount] = activeCellStatusList [counter1*12+4], activeCellStatusListKeepCount++;

                                        activeCellStatusListKeep [activeCellStatusListKeepCount] = newCellNumber2, activeCellStatusListKeepCount++;

                                        activeCellStatusListKeep [activeCellStatusListKeepCount] = newCellNumber1, activeCellStatusListKeepCount++;

                                        activeCellStatusListKeep [activeCellStatusListKeepCount] = 0, activeCellStatusListKeepCount++;

                                        activeCellStatusListKeep [activeCellStatusListKeepCount] = activeCellStatusList [counter1*12+3], activeCellStatusListKeepCount++;

                                        activeCellStatusListKeep [activeCellStatusListKeepCount] = 0, activeCellStatusListKeepCount++;

                                        activeCellStatusListKeep [activeCellStatusListKeepCount] = 1, activeCellStatusListKeepCount++;

                                        activeCellStatusListKeep [activeCellStatusListKeepCount] = 0, activeCellStatusListKeepCount++;

                                        activeCellStatusListKeep [activeCellStatusListKeepCount] = activeCellStatusList [counter1*12+7], activeCellStatusListKeepCount++;

                                        activeCellStatusListKeep [activeCellStatusListKeepCount] = activeCellStatusList [counter1*12+8], activeCellStatusListKeepCount++;

                                        activeCellStatusListKeep [activeCellStatusListKeepCount] = activeCellStatusList [counter1*12+9], activeCellStatusListKeepCount++;

                                        activeCellStatusListKeep [activeCellStatusListKeepCount] = activeCellStatusList [counter1*12], activeCellStatusListKeepCount++;

                                        activeCellStatusListKeep [activeCellStatusListKeepCount] = activeCellStatusList [counter1*12+4], activeCellStatusListKeepCount++;

                                    }

                                    activeCellStatusList [counter1*12+1] = -1;

                                }

**else** **if** (extendEnd+2 == growthCycleBase){

                                    cellLineageTempArray [cellLineageTempArrayCount] = 1, cellLineageTempArrayCount++;

                                    cellLineageTempArray [cellLineageTempArrayCount] = 1, cellLineageTempArrayCount++;

                                    cellLineageTempArray [cellLineageTempArrayCount] = extendEnd+1, cellLineageTempArrayCount++;

                                    cellLineageTempArray [cellLineageTempArrayCount] = 2, cellLineageTempArrayCount++;

                                    cellLineageTempArray [cellLineageTempArrayCount] = 0, cellLineageTempArrayCount++;

                                    cellLineageTempArray [cellLineageTempArrayCount] = activeCellStatusList [counter1*12], cellLineageTempArrayCount++;

                                    cellLineageTempArray [cellLineageTempArrayCount] = activeCellStatusList [counter1*12+3], cellLineageTempArrayCount++;

                                    cellLineageTempArray [cellLineageTempArrayCount] = 0, cellLineageTempArrayCount++;

                                    cellLineageTempArray [cellLineageTempArrayCount] = 0, cellLineageTempArrayCount++;

                                    cellLineageTempArray [cellLineageTempArrayCount] = 1, cellLineageTempArrayCount++;

                                    cellLineageTempArray [cellLineageTempArrayCount] = 1, cellLineageTempArrayCount++;

                                    cellLineageTempArray [cellLineageTempArrayCount] = extendEnd+2, cellLineageTempArrayCount++;

                                    cellLineageTempArray [cellLineageTempArrayCount] = 2, cellLineageTempArrayCount++;

                                    cellLineageTempArray [cellLineageTempArrayCount] = 0, cellLineageTempArrayCount++;

                                    cellLineageTempArray [cellLineageTempArrayCount] = activeCellStatusList [counter1*12], cellLineageTempArrayCount++;

                                    cellLineageTempArray [cellLineageTempArrayCount] = activeCellStatusList [counter1*12+3], cellLineageTempArrayCount++;

                                    cellLineageTempArray [cellLineageTempArrayCount] = 0, cellLineageTempArrayCount++;

                                    cellLineageTempArray [cellLineageTempArrayCount] = 0, cellLineageTempArrayCount++;

**for** (**unsigned** **long** counter2 = 0; counter2 < cellLineageSummaryArrayCount/9; counter2++){

**if** (cellLineageSummaryArray [counter2*9+2] == activeCellStatusList [counter1*12+3] && cellLineageSummaryArray [counter2*9+3] == activeCellStatusList [counter1*12]){

                                            cellLineageSummaryArray [counter2*9+1] = (**long**)cellLineageTempArrayCount-9;

                                            cellLineageSummaryArray [counter2*9+7] = 2;

**break**;

                                        }

                                    }

                                    activeCellStatusListKeep [activeCellStatusListKeepCount] = activeCellStatusListHold [counter1*12], activeCellStatusListKeepCount++;

                                    activeCellStatusListKeep [activeCellStatusListKeepCount] = activeCellStatusListHold [counter1*12+1], activeCellStatusListKeepCount++;

                                    activeCellStatusListKeep [activeCellStatusListKeepCount] = activeCellStatusListHold [counter1*12+2], activeCellStatusListKeepCount++;

                                    activeCellStatusListKeep [activeCellStatusListKeepCount] = activeCellStatusListHold [counter1*12+3], activeCellStatusListKeepCount++;

                                    activeCellStatusListKeep [activeCellStatusListKeepCount] = activeCellStatusListHold [counter1*12+4], activeCellStatusListKeepCount++;

                                    activeCellStatusListKeep [activeCellStatusListKeepCount] = activeCellStatusListHold [counter1*12+5], activeCellStatusListKeepCount++;

                                    activeCellStatusListKeep [activeCellStatusListKeepCount] = activeCellStatusListHold [counter1*12+6], activeCellStatusListKeepCount++;

                                    activeCellStatusListKeep [activeCellStatusListKeepCount] = activeCellStatusListHold [counter1*12+7], activeCellStatusListKeepCount++;

                                    activeCellStatusListKeep [activeCellStatusListKeepCount] = activeCellStatusListHold [counter1*12+8], activeCellStatusListKeepCount++;

                                    activeCellStatusListKeep [activeCellStatusListKeepCount] = activeCellStatusListHold [counter1*12+9], activeCellStatusListKeepCount++;

                                    activeCellStatusListKeep [activeCellStatusListKeepCount] = activeCellStatusListHold [counter1*12+10], activeCellStatusListKeepCount++;

                                    activeCellStatusListKeep [activeCellStatusListKeepCount] = activeCellStatusListHold [counter1*12+11], activeCellStatusListKeepCount++;

                                    activeCellStatusList [counter1*12+1] = -1;

                                }

**else** **if** (extendEnd+1 == growthCycleBase){

                                    cellLineageTempArray [cellLineageTempArrayCount] = 1, cellLineageTempArrayCount++;

                                    cellLineageTempArray [cellLineageTempArrayCount] = 1, cellLineageTempArrayCount++;

                                    cellLineageTempArray [cellLineageTempArrayCount] = extendEnd+1, cellLineageTempArrayCount++;

                                    cellLineageTempArray [cellLineageTempArrayCount] = 2, cellLineageTempArrayCount++;

                                    cellLineageTempArray [cellLineageTempArrayCount] = 0, cellLineageTempArrayCount++;

                                    cellLineageTempArray [cellLineageTempArrayCount] = activeCellStatusList [counter1*12], cellLineageTempArrayCount++;

                                    cellLineageTempArray [cellLineageTempArrayCount] = activeCellStatusList [counter1*12+3], cellLineageTempArrayCount++;

                                    cellLineageTempArray [cellLineageTempArrayCount] = 0, cellLineageTempArrayCount++;

                                    cellLineageTempArray [cellLineageTempArrayCount] = 0, cellLineageTempArrayCount++;

**for** (**unsigned** **long** counter2 = 0; counter2 < cellLineageSummaryArrayCount/9; counter2++){

**if** (cellLineageSummaryArray [counter2*9+2] == activeCellStatusList [counter1*12+3] && cellLineageSummaryArray [counter2*9+3] == activeCellStatusList [counter1*12]){

                                            cellLineageSummaryArray [counter2*9+1] = (**long**)cellLineageTempArrayCount-9;

                                            cellLineageSummaryArray [counter2*9+7] = 2;

**break**;

                                        }

                                    }

                                    activeCellStatusListKeep [activeCellStatusListKeepCount] = activeCellStatusListHold [counter1*12], activeCellStatusListKeepCount++;

                                    activeCellStatusListKeep [activeCellStatusListKeepCount] = activeCellStatusListHold [counter1*12+1], activeCellStatusListKeepCount++;

                                    activeCellStatusListKeep [activeCellStatusListKeepCount] = activeCellStatusListHold [counter1*12+2], activeCellStatusListKeepCount++;

                                    activeCellStatusListKeep [activeCellStatusListKeepCount] = activeCellStatusListHold [counter1*12+3], activeCellStatusListKeepCount++;

                                    activeCellStatusListKeep [activeCellStatusListKeepCount] = activeCellStatusListHold [counter1*12+4], activeCellStatusListKeepCount++;

                                    activeCellStatusListKeep [activeCellStatusListKeepCount] = activeCellStatusListHold [counter1*12+5], activeCellStatusListKeepCount++;

                                    activeCellStatusListKeep [activeCellStatusListKeepCount] = activeCellStatusListHold [counter1*12+6], activeCellStatusListKeepCount++;

                                    activeCellStatusListKeep [activeCellStatusListKeepCount] = activeCellStatusListHold [counter1*12+7], activeCellStatusListKeepCount++;

                                    activeCellStatusListKeep [activeCellStatusListKeepCount] = activeCellStatusListHold [counter1*12+8], activeCellStatusListKeepCount++;

                                    activeCellStatusListKeep [activeCellStatusListKeepCount] = activeCellStatusListHold [counter1*12+9], activeCellStatusListKeepCount++;

                                    activeCellStatusListKeep [activeCellStatusListKeepCount] = activeCellStatusListHold [counter1*12+10], activeCellStatusListKeepCount++;

                                    activeCellStatusListKeep [activeCellStatusListKeepCount] = activeCellStatusListHold [counter1*12+11], activeCellStatusListKeepCount++;

                                    activeCellStatusList [counter1*12+1] = -1;

                                }

                            }

**if** (activeCellStatusList [counter1*12+5] == 2){

**if** (extendEnd+3 <= growthCycleBase){

                                    cellLineageTempArray [cellLineageTempArrayCount] = 1, cellLineageTempArrayCount++;

                                    cellLineageTempArray [cellLineageTempArrayCount] = 1, cellLineageTempArrayCount++;

                                    cellLineageTempArray [cellLineageTempArrayCount] = extendEnd+1, cellLineageTempArrayCount++;

                                    cellLineageTempArray [cellLineageTempArrayCount] = 6, cellLineageTempArrayCount++;

                                    cellLineageTempArray [cellLineageTempArrayCount] = 0, cellLineageTempArrayCount++;

                                    cellLineageTempArray [cellLineageTempArrayCount] = activeCellStatusList [counter1*12], cellLineageTempArrayCount++;

                                    cellLineageTempArray [cellLineageTempArrayCount] = activeCellStatusList [counter1*12+3], cellLineageTempArrayCount++;

                                    cellLineageTempArray [cellLineageTempArrayCount] = 0, cellLineageTempArrayCount++;

                                    cellLineageTempArray [cellLineageTempArrayCount] = 0, cellLineageTempArrayCount++;

                                    cellLineageTempArray [cellLineageTempArrayCount] = 1, cellLineageTempArrayCount++;

                                    cellLineageTempArray [cellLineageTempArrayCount] = 1, cellLineageTempArrayCount++;

                                    cellLineageTempArray [cellLineageTempArrayCount] = extendEnd+2, cellLineageTempArrayCount++;

                                    cellLineageTempArray [cellLineageTempArrayCount] = 42, cellLineageTempArrayCount++;

                                    cellLineageTempArray [cellLineageTempArrayCount] = 0, cellLineageTempArrayCount++;

                                    cellLineageTempArray [cellLineageTempArrayCount] = activeCellStatusList [counter1*12], cellLineageTempArrayCount++;

                                    cellLineageTempArray [cellLineageTempArrayCount] = activeCellStatusList [counter1*12+3], cellLineageTempArrayCount++;

                                    cellLineageTempArray [cellLineageTempArrayCount] = 0, cellLineageTempArrayCount++;

                                    cellLineageTempArray [cellLineageTempArrayCount] = 0, cellLineageTempArrayCount++;

**for** (**unsigned** **long** counter2 = 0; counter2 < cellLineageSummaryArrayCount/9; counter2++){

**if** (cellLineageSummaryArray [counter2*9+2] == activeCellStatusList [counter1*12+3] && cellLineageSummaryArray [counter2*9+3] == activeCellStatusList [counter1*12]){

                                            cellLineageSummaryArray [counter2*9+1] = (**long**)cellLineageTempArrayCount-9;

                                            cellLineageSummaryArray [counter2*9+7] = 42;

**break**;

                                        }

                                    }

                                    //========Assign cell no to generated progeny========

                                    createNewCellNo = [[CreateNewCellNo alloc] init];

                                    newCellNumber1 = [createNewCellNo cellNumberAddition:counter1];

                                    [CreateNewCellNo release];

                                    cellNoLingNoList [cellNoLingNoListCount] = activeCellStatusList [counter1*12+3], cellNoLingNoListCount++;

                                    cellNoLingNoList [cellNoLingNoListCount] = newCellNumber1, cellNoLingNoListCount++;

                                    createNewCellNo = [[CreateNewCellNo alloc] init];

                                    newCellNumber2 = [createNewCellNo cellNumberSubtraction:counter1];

                                    [CreateNewCellNo release];

                                    cellNoLingNoList [cellNoLingNoListCount] = activeCellStatusList [counter1*12+3], cellNoLingNoListCount++;

                                    cellNoLingNoList [cellNoLingNoListCount] = newCellNumber2, cellNoLingNoListCount++;

                                    createNewCellNo = [[CreateNewCellNo alloc] init];

                                    newCellNumber3 = [createNewCellNo cellNumberAdditionSecond:counter1];

                                    [CreateNewCellNo release];

                                    cellNoLingNoList [cellNoLingNoListCount] = activeCellStatusList [counter1*12+3], cellNoLingNoListCount++;

                                    cellNoLingNoList [cellNoLingNoListCount] = newCellNumber3, cellNoLingNoListCount++;

                                    cellLineageTempArray [cellLineageTempArrayCount] = 1, cellLineageTempArrayCount++;

                                    cellLineageTempArray [cellLineageTempArrayCount] = 1, cellLineageTempArrayCount++;

                                    cellLineageTempArray [cellLineageTempArrayCount] = extendEnd+3, cellLineageTempArrayCount++;

                                    cellLineageTempArray [cellLineageTempArrayCount] = 41, cellLineageTempArrayCount++;

                                    cellLineageTempArray [cellLineageTempArrayCount] = activeCellStatusList [counter1*12], cellLineageTempArrayCount++;

                                    cellLineageTempArray [cellLineageTempArrayCount] = newCellNumber1, cellLineageTempArrayCount++;

                                    cellLineageTempArray [cellLineageTempArrayCount] = activeCellStatusList [counter1*12+3], cellLineageTempArrayCount++;

                                    cellLineageTempArray [cellLineageTempArrayCount] = 0, cellLineageTempArrayCount++;

                                    cellLineageTempArray [cellLineageTempArrayCount] = 0, cellLineageTempArrayCount++;

                                    cellLineageTempArray [cellLineageTempArrayCount] = 1, cellLineageTempArrayCount++;

                                    cellLineageTempArray [cellLineageTempArrayCount] = 1, cellLineageTempArrayCount++;

                                    cellLineageTempArray [cellLineageTempArrayCount] = extendEnd+3, cellLineageTempArrayCount++;

                                    cellLineageTempArray [cellLineageTempArrayCount] = 41, cellLineageTempArrayCount++;

                                    cellLineageTempArray [cellLineageTempArrayCount] = activeCellStatusList [counter1*12], cellLineageTempArrayCount++;

                                    cellLineageTempArray [cellLineageTempArrayCount] = newCellNumber2, cellLineageTempArrayCount++;

                                    cellLineageTempArray [cellLineageTempArrayCount] = activeCellStatusList [counter1*12+3], cellLineageTempArrayCount++;

                                    cellLineageTempArray [cellLineageTempArrayCount] = 0, cellLineageTempArrayCount++;

                                    cellLineageTempArray [cellLineageTempArrayCount] = 0, cellLineageTempArrayCount++;

                                    cellLineageTempArray [cellLineageTempArrayCount] = 1, cellLineageTempArrayCount++;

                                    cellLineageTempArray [cellLineageTempArrayCount] = 1, cellLineageTempArrayCount++;

                                    cellLineageTempArray [cellLineageTempArrayCount] = extendEnd+3, cellLineageTempArrayCount++;

                                    cellLineageTempArray [cellLineageTempArrayCount] = 41, cellLineageTempArrayCount++;

                                    cellLineageTempArray [cellLineageTempArrayCount] = activeCellStatusList [counter1*12], cellLineageTempArrayCount++;

                                    cellLineageTempArray [cellLineageTempArrayCount] = newCellNumber3, cellLineageTempArrayCount++;

                                    cellLineageTempArray [cellLineageTempArrayCount] = activeCellStatusList [counter1*12+3], cellLineageTempArrayCount++;

                                    cellLineageTempArray [cellLineageTempArrayCount] = 0, cellLineageTempArrayCount++;

                                    cellLineageTempArray [cellLineageTempArrayCount] = 0, cellLineageTempArrayCount++;

                                    cellLineageSummaryArray [cellLineageSummaryArrayCount] = (**long**)cellLineageTempArrayCount-27, cellLineageSummaryArrayCount++;

                                    cellLineageSummaryArray [cellLineageSummaryArrayCount] = (**long**)cellLineageTempArrayCount-27, cellLineageSummaryArrayCount++;

                                    cellLineageSummaryArray [cellLineageSummaryArrayCount] = (**long**)activeCellStatusList [counter1*12+3], cellLineageSummaryArrayCount++;

                                    cellLineageSummaryArray [cellLineageSummaryArrayCount] = (**long**)newCellNumber1, cellLineageSummaryArrayCount++;

                                    cellLineageSummaryArray [cellLineageSummaryArrayCount] = (**long**)activeCellStatusList [counter1*12], cellLineageSummaryArrayCount++;

                                    cellLineageSummaryArray [cellLineageSummaryArrayCount] = (**long**)activeCellStatusList [counter1*12+3], cellLineageSummaryArrayCount++;

                                    cellLineageSummaryArray [cellLineageSummaryArrayCount] = 41, cellLineageSummaryArrayCount++;

                                    cellLineageSummaryArray [cellLineageSummaryArrayCount] = 2, cellLineageSummaryArrayCount++;

                                    cellLineageSummaryArray [cellLineageSummaryArrayCount] = 0, cellLineageSummaryArrayCount++;

                                    cellLineageSummaryArray [cellLineageSummaryArrayCount] = (**long**)cellLineageTempArrayCount-18, cellLineageSummaryArrayCount++;

                                    cellLineageSummaryArray [cellLineageSummaryArrayCount] = (**long**)cellLineageTempArrayCount-18, cellLineageSummaryArrayCount++;

                                    cellLineageSummaryArray [cellLineageSummaryArrayCount] = (**long**)activeCellStatusList [counter1*12+3], cellLineageSummaryArrayCount++;

                                    cellLineageSummaryArray [cellLineageSummaryArrayCount] = (**long**)newCellNumber2, cellLineageSummaryArrayCount++;

                                    cellLineageSummaryArray [cellLineageSummaryArrayCount] = (**long**)activeCellStatusList [counter1*12], cellLineageSummaryArrayCount++;

                                    cellLineageSummaryArray [cellLineageSummaryArrayCount] = (**long**)activeCellStatusList [counter1*12+3], cellLineageSummaryArrayCount++;

                                    cellLineageSummaryArray [cellLineageSummaryArrayCount] = 41, cellLineageSummaryArrayCount++;

                                    cellLineageSummaryArray [cellLineageSummaryArrayCount] = 2, cellLineageSummaryArrayCount++;

                                    cellLineageSummaryArray [cellLineageSummaryArrayCount] = 0, cellLineageSummaryArrayCount++;

                                    cellLineageSummaryArray [cellLineageSummaryArrayCount] = (**long**)cellLineageTempArrayCount-9, cellLineageSummaryArrayCount++;

                                    cellLineageSummaryArray [cellLineageSummaryArrayCount] = (**long**)cellLineageTempArrayCount-9, cellLineageSummaryArrayCount++;

                                    cellLineageSummaryArray [cellLineageSummaryArrayCount] = (**long**)activeCellStatusList [counter1*12+3], cellLineageSummaryArrayCount++;

                                    cellLineageSummaryArray [cellLineageSummaryArrayCount] = (**long**)newCellNumber3, cellLineageSummaryArrayCount++;

                                    cellLineageSummaryArray [cellLineageSummaryArrayCount] = (**long**)activeCellStatusList [counter1*12], cellLineageSummaryArrayCount++;

                                    cellLineageSummaryArray [cellLineageSummaryArrayCount] = (**long**)activeCellStatusList [counter1*12+3], cellLineageSummaryArrayCount++;

                                    cellLineageSummaryArray [cellLineageSummaryArrayCount] = 41, cellLineageSummaryArrayCount++;

                                    cellLineageSummaryArray [cellLineageSummaryArrayCount] = 2, cellLineageSummaryArrayCount++;

                                    cellLineageSummaryArray [cellLineageSummaryArrayCount] = 0, cellLineageSummaryArrayCount++;

**if** (extendEnd+3 < growthCycleBase){

                                        tempListOfCells [tempListOfCellsCount] = activeCellStatusList [counter1*12+3], tempListOfCellsCount++;

                                        tempListOfCells [tempListOfCellsCount] = activeCellStatusList [counter1*12], tempListOfCellsCount++;

                                        tempListOfCells [tempListOfCellsCount] = newCellNumber1, tempListOfCellsCount++;

                                        tempListOfCells [tempListOfCellsCount] = newCellNumber2, tempListOfCellsCount++;

                                        tempListOfCells [tempListOfCellsCount] = newCellNumber3, tempListOfCellsCount++;

                                        tempListOfCells [tempListOfCellsCount] = 41, tempListOfCellsCount++;

                                        tempListOfCells [tempListOfCellsCount] = activeCellStatusList [counter1*12+4], tempListOfCellsCount++;

                                    }

**else** **if** (extendEnd+3 == growthCycleBase){

                                        activeCellStatusListKeep [activeCellStatusListKeepCount] = newCellNumber1, activeCellStatusListKeepCount++;

                                        activeCellStatusListKeep [activeCellStatusListKeepCount] = newCellNumber2, activeCellStatusListKeepCount++;

                                        activeCellStatusListKeep [activeCellStatusListKeepCount] = newCellNumber3, activeCellStatusListKeepCount++;

                                        activeCellStatusListKeep [activeCellStatusListKeepCount] = activeCellStatusList [counter1*12+3], activeCellStatusListKeepCount++;

                                        activeCellStatusListKeep [activeCellStatusListKeepCount] = 0, activeCellStatusListKeepCount++;

                                        activeCellStatusListKeep [activeCellStatusListKeepCount] = 1, activeCellStatusListKeepCount++;

                                        activeCellStatusListKeep [activeCellStatusListKeepCount] = 0, activeCellStatusListKeepCount++;

                                        activeCellStatusListKeep [activeCellStatusListKeepCount] = activeCellStatusList [counter1*12+7], activeCellStatusListKeepCount++;

                                        activeCellStatusListKeep [activeCellStatusListKeepCount] = activeCellStatusList [counter1*12+8], activeCellStatusListKeepCount++;

                                        activeCellStatusListKeep [activeCellStatusListKeepCount] = activeCellStatusList [counter1*12+9], activeCellStatusListKeepCount++;

                                        activeCellStatusListKeep [activeCellStatusListKeepCount] = activeCellStatusList [counter1*12], activeCellStatusListKeepCount++;

                                        activeCellStatusListKeep [activeCellStatusListKeepCount] = activeCellStatusList [counter1*12+4], activeCellStatusListKeepCount++;

                                        activeCellStatusListKeep [activeCellStatusListKeepCount] = newCellNumber2, activeCellStatusListKeepCount++;

                                        activeCellStatusListKeep [activeCellStatusListKeepCount] = newCellNumber3, activeCellStatusListKeepCount++;

                                        activeCellStatusListKeep [activeCellStatusListKeepCount] = newCellNumber1, activeCellStatusListKeepCount++;

                                        activeCellStatusListKeep [activeCellStatusListKeepCount] = activeCellStatusList [counter1*12+3], activeCellStatusListKeepCount++;

                                        activeCellStatusListKeep [activeCellStatusListKeepCount] = 0, activeCellStatusListKeepCount++;

                                        activeCellStatusListKeep [activeCellStatusListKeepCount] = 1, activeCellStatusListKeepCount++;

                                        activeCellStatusListKeep [activeCellStatusListKeepCount] = 0, activeCellStatusListKeepCount++;

                                        activeCellStatusListKeep [activeCellStatusListKeepCount] = activeCellStatusList [counter1*12+7], activeCellStatusListKeepCount++;

                                        activeCellStatusListKeep [activeCellStatusListKeepCount] = activeCellStatusList [counter1*12+8], activeCellStatusListKeepCount++;

                                        activeCellStatusListKeep [activeCellStatusListKeepCount] = activeCellStatusList [counter1*12+9], activeCellStatusListKeepCount++;

                                        activeCellStatusListKeep [activeCellStatusListKeepCount] = activeCellStatusList [counter1*12], activeCellStatusListKeepCount++;

                                        activeCellStatusListKeep [activeCellStatusListKeepCount] = activeCellStatusList [counter1*12+4], activeCellStatusListKeepCount++;

                                        activeCellStatusListKeep [activeCellStatusListKeepCount] = newCellNumber3, activeCellStatusListKeepCount++;

                                        activeCellStatusListKeep [activeCellStatusListKeepCount] = newCellNumber1, activeCellStatusListKeepCount++;

                                        activeCellStatusListKeep [activeCellStatusListKeepCount] = newCellNumber2, activeCellStatusListKeepCount++;

                                        activeCellStatusListKeep [activeCellStatusListKeepCount] = activeCellStatusList [counter1*12+3], activeCellStatusListKeepCount++;

                                        activeCellStatusListKeep [activeCellStatusListKeepCount] = 0, activeCellStatusListKeepCount++;

                                        activeCellStatusListKeep [activeCellStatusListKeepCount] = 1, activeCellStatusListKeepCount++;

                                        activeCellStatusListKeep [activeCellStatusListKeepCount] = 0, activeCellStatusListKeepCount++;

                                        activeCellStatusListKeep [activeCellStatusListKeepCount] = activeCellStatusList [counter1*12+7], activeCellStatusListKeepCount++;

                                        activeCellStatusListKeep [activeCellStatusListKeepCount] = activeCellStatusList [counter1*12+8], activeCellStatusListKeepCount++;

                                        activeCellStatusListKeep [activeCellStatusListKeepCount] = activeCellStatusList [counter1*12+9], activeCellStatusListKeepCount++;

                                        activeCellStatusListKeep [activeCellStatusListKeepCount] = activeCellStatusList [counter1*12], activeCellStatusListKeepCount++;

                                        activeCellStatusListKeep [activeCellStatusListKeepCount] = activeCellStatusList [counter1*12+4], activeCellStatusListKeepCount++;

                                    }

                                    activeCellStatusList [counter1*12+1] = -1;

                                }

**else** **if** (extendEnd+2 == growthCycleBase){

                                    cellLineageTempArray [cellLineageTempArrayCount] = 1, cellLineageTempArrayCount++;

                                    cellLineageTempArray [cellLineageTempArrayCount] = 1, cellLineageTempArrayCount++;

                                    cellLineageTempArray [cellLineageTempArrayCount] = extendEnd+1, cellLineageTempArrayCount++;

                                    cellLineageTempArray [cellLineageTempArrayCount] = 2, cellLineageTempArrayCount++;

                                    cellLineageTempArray [cellLineageTempArrayCount] = 0, cellLineageTempArrayCount++;

                                    cellLineageTempArray [cellLineageTempArrayCount] = activeCellStatusList [counter1*12], cellLineageTempArrayCount++;

                                    cellLineageTempArray [cellLineageTempArrayCount] = activeCellStatusList [counter1*12+3], cellLineageTempArrayCount++;

                                    cellLineageTempArray [cellLineageTempArrayCount] = 0, cellLineageTempArrayCount++;

                                    cellLineageTempArray [cellLineageTempArrayCount] = 0, cellLineageTempArrayCount++;

                                    cellLineageTempArray [cellLineageTempArrayCount] = 1, cellLineageTempArrayCount++;

                                    cellLineageTempArray [cellLineageTempArrayCount] = 1, cellLineageTempArrayCount++;

                                    cellLineageTempArray [cellLineageTempArrayCount] = extendEnd+2, cellLineageTempArrayCount++;

                                    cellLineageTempArray [cellLineageTempArrayCount] = 2, cellLineageTempArrayCount++;

                                    cellLineageTempArray [cellLineageTempArrayCount] = 0, cellLineageTempArrayCount++;

                                    cellLineageTempArray [cellLineageTempArrayCount] = activeCellStatusList [counter1*12], cellLineageTempArrayCount++;

                                    cellLineageTempArray [cellLineageTempArrayCount] = activeCellStatusList [counter1*12+3], cellLineageTempArrayCount++;

                                    cellLineageTempArray [cellLineageTempArrayCount] = 0, cellLineageTempArrayCount++;

                                    cellLineageTempArray [cellLineageTempArrayCount] = 0, cellLineageTempArrayCount++;

**for** (**unsigned** **long** counter2 = 0; counter2 < cellLineageSummaryArrayCount/9; counter2++){

**if** (cellLineageSummaryArray [counter2*9+2] == activeCellStatusList [counter1*12+3] && cellLineageSummaryArray [counter2*9+3] == activeCellStatusList [counter1*12]){

                                            cellLineageSummaryArray [counter2*9+1] = (**long**)cellLineageTempArrayCount-9;

                                            cellLineageSummaryArray [counter2*9+7] = 2;

**break**;

                                        }

                                    }

                                    activeCellStatusListKeep [activeCellStatusListKeepCount] = activeCellStatusListHold [counter1*12], activeCellStatusListKeepCount++;

                                    activeCellStatusListKeep [activeCellStatusListKeepCount] = activeCellStatusListHold [counter1*12+1], activeCellStatusListKeepCount++;

                                    activeCellStatusListKeep [activeCellStatusListKeepCount] = activeCellStatusListHold [counter1*12+2], activeCellStatusListKeepCount++;

                                    activeCellStatusListKeep [activeCellStatusListKeepCount] = activeCellStatusListHold [counter1*12+3], activeCellStatusListKeepCount++;

                                    activeCellStatusListKeep [activeCellStatusListKeepCount] = activeCellStatusListHold [counter1*12+4], activeCellStatusListKeepCount++;

                                    activeCellStatusListKeep [activeCellStatusListKeepCount] = activeCellStatusListHold [counter1*12+5], activeCellStatusListKeepCount++;

                                    activeCellStatusListKeep [activeCellStatusListKeepCount] = activeCellStatusListHold [counter1*12+6], activeCellStatusListKeepCount++;

                                    activeCellStatusListKeep [activeCellStatusListKeepCount] = activeCellStatusListHold [counter1*12+7], activeCellStatusListKeepCount++;

                                    activeCellStatusListKeep [activeCellStatusListKeepCount] = activeCellStatusListHold [counter1*12+8], activeCellStatusListKeepCount++;

                                    activeCellStatusListKeep [activeCellStatusListKeepCount] = activeCellStatusListHold [counter1*12+9], activeCellStatusListKeepCount++;

                                    activeCellStatusListKeep [activeCellStatusListKeepCount] = activeCellStatusListHold [counter1*12+10], activeCellStatusListKeepCount++;

                                    activeCellStatusListKeep [activeCellStatusListKeepCount] = activeCellStatusListHold [counter1*12+11], activeCellStatusListKeepCount++;

                                    activeCellStatusList [counter1*12+1] = -1;

                                }

**else** **if** (extendEnd+1 == growthCycleBase){

                                    cellLineageTempArray [cellLineageTempArrayCount] = 1, cellLineageTempArrayCount++;

                                    cellLineageTempArray [cellLineageTempArrayCount] = 1, cellLineageTempArrayCount++;

                                    cellLineageTempArray [cellLineageTempArrayCount] = extendEnd+1, cellLineageTempArrayCount++;

                                    cellLineageTempArray [cellLineageTempArrayCount] = 2, cellLineageTempArrayCount++;

                                    cellLineageTempArray [cellLineageTempArrayCount] = 0, cellLineageTempArrayCount++;

                                    cellLineageTempArray [cellLineageTempArrayCount] = activeCellStatusList [counter1*12], cellLineageTempArrayCount++;

                                    cellLineageTempArray [cellLineageTempArrayCount] = activeCellStatusList [counter1*12+3], cellLineageTempArrayCount++;

                                    cellLineageTempArray [cellLineageTempArrayCount] = 0, cellLineageTempArrayCount++;

                                    cellLineageTempArray [cellLineageTempArrayCount] = 0, cellLineageTempArrayCount++;

**for** (**unsigned** **long** counter2 = 0; counter2 < cellLineageSummaryArrayCount/9; counter2++){

**if** (cellLineageSummaryArray [counter2*9+2] == activeCellStatusList [counter1*12+3] && cellLineageSummaryArray [counter2*9+3] == activeCellStatusList [counter1*12]){

                                            cellLineageSummaryArray [counter2*9+1] = (**long**)cellLineageTempArrayCount-9;

                                            cellLineageSummaryArray [counter2*9+7] = 2;

**break**;

                                        }

                                    }

                                    activeCellStatusListKeep [activeCellStatusListKeepCount] = activeCellStatusListHold [counter1*12], activeCellStatusListKeepCount++;

                                    activeCellStatusListKeep [activeCellStatusListKeepCount] = activeCellStatusListHold [counter1*12+1], activeCellStatusListKeepCount++;

                                    activeCellStatusListKeep [activeCellStatusListKeepCount] = activeCellStatusListHold [counter1*12+2], activeCellStatusListKeepCount++;

                                    activeCellStatusListKeep [activeCellStatusListKeepCount] = activeCellStatusListHold [counter1*12+3], activeCellStatusListKeepCount++;

                                    activeCellStatusListKeep [activeCellStatusListKeepCount] = activeCellStatusListHold [counter1*12+4], activeCellStatusListKeepCount++;

                                    activeCellStatusListKeep [activeCellStatusListKeepCount] = activeCellStatusListHold [counter1*12+5], activeCellStatusListKeepCount++;

                                    activeCellStatusListKeep [activeCellStatusListKeepCount] = activeCellStatusListHold [counter1*12+6], activeCellStatusListKeepCount++;

                                    activeCellStatusListKeep [activeCellStatusListKeepCount] = activeCellStatusListHold [counter1*12+7], activeCellStatusListKeepCount++;

                                    activeCellStatusListKeep [activeCellStatusListKeepCount] = activeCellStatusListHold [counter1*12+8], activeCellStatusListKeepCount++;

                                    activeCellStatusListKeep [activeCellStatusListKeepCount] = activeCellStatusListHold [counter1*12+9], activeCellStatusListKeepCount++;

                                    activeCellStatusListKeep [activeCellStatusListKeepCount] = activeCellStatusListHold [counter1*12+10], activeCellStatusListKeepCount++;

                                    activeCellStatusListKeep [activeCellStatusListKeepCount] = activeCellStatusListHold [counter1*12+11], activeCellStatusListKeepCount++;

                                    activeCellStatusList [counter1*12+1] = -1;

                                }

                            }

**if** (activeCellStatusList [counter1*12+5] == 3){

**if** (extendEnd+1 <= growthCycleBase){

                                    cellLineageTempArray [cellLineageTempArrayCount] = 1, cellLineageTempArrayCount++;

                                    cellLineageTempArray [cellLineageTempArrayCount] = 1, cellLineageTempArrayCount++;

                                    cellLineageTempArray [cellLineageTempArrayCount] = extendEnd+1, cellLineageTempArrayCount++;

                                    cellLineageTempArray [cellLineageTempArrayCount] = 7, cellLineageTempArrayCount++;

                                    cellLineageTempArray [cellLineageTempArrayCount] = 0, cellLineageTempArrayCount++;

                                    cellLineageTempArray [cellLineageTempArrayCount] = activeCellStatusList [counter1*12], cellLineageTempArrayCount++;

                                    cellLineageTempArray [cellLineageTempArrayCount] = activeCellStatusList [counter1*12+3], cellLineageTempArrayCount++;

                                    cellLineageTempArray [cellLineageTempArrayCount] = 0, cellLineageTempArrayCount++;

                                    cellLineageTempArray [cellLineageTempArrayCount] = 0, cellLineageTempArrayCount++;

**for** (**unsigned** **long** counter2 = 0; counter2 < cellLineageSummaryArrayCount/9; counter2++){

**if** (cellLineageSummaryArray [counter2*9+2] == activeCellStatusList [counter1*12+3] && cellLineageSummaryArray [counter2*9+3] == activeCellStatusList [counter1*12]){

                                            cellLineageSummaryArray [counter2*9+1] = (**long**)cellLineageTempArrayCount-9;

                                            cellLineageSummaryArray [counter2*9+7] = 7;

**break**;

                                        }

                                    }

                                    activeCellStatusList [counter1*12+1] = -1;

                                }

                            }

                        }

**int** prevTDCount = 0;

**int** prevTDTD = 0;

                        //====Enter information of cells to the "activeCellStatusList" from the "tempListOfCells".====

**for** (**int** counter1 = 0; counter1 < tempListOfCellsCount/7; counter1++){

**if** (activeCellStatusListCount+50 > activeCellStatusListLimitHold){ //====Array size management====

**int** *arrayUpDate = **new** **int** [activeCellStatusListCount+10];

**for** (**int** counter2 = 0; counter2 < activeCellStatusListCount; counter2++) arrayUpDate [counter2] = activeCellStatusList [counter2];

**delete** [] activeCellStatusList;

                                activeCellStatusList = **new** **int** [activeCellStatusListLimitHold+10000];

                                activeCellStatusListLimitHold = activeCellStatusListLimitHold+10000;

**for** (**int** counter2 = 0; counter2 < activeCellStatusListCount; counter2++) activeCellStatusList [counter2] = arrayUpDate [counter2];

**delete** [] arrayUpDate;

                            }

                            prevTDCount = 0;

                            prevTDTD = 0;

**for** (**int** counter2 = 0; counter2 < activeCellStatusListCount/12; counter2++){

**if** (activeCellStatusList [counter2*12] == tempListOfCells [counter1*7+1] && activeCellStatusList [counter2*12+3] == tempListOfCells [counter1*7]){

                                    prevTDCount = activeCellStatusList [counter2*12+7];

                                    prevTDTD = activeCellStatusList [counter2*12+8];

**break**;

                                }

                            }

**if** (tempListOfCells [counter1*7+5]== 31){

                                activeCellStatusList [activeCellStatusListCount] = tempListOfCells [counter1*7+2], activeCellStatusListCount++;

                                activeCellStatusList [activeCellStatusListCount] = tempListOfCells [counter1*7+3], activeCellStatusListCount++;

                                activeCellStatusList [activeCellStatusListCount] = 0, activeCellStatusListCount++;

                                activeCellStatusList [activeCellStatusListCount] = tempListOfCells [counter1*7], activeCellStatusListCount++;

                                activeCellStatusList [activeCellStatusListCount] = 0, activeCellStatusListCount++;

                                activeCellStatusList [activeCellStatusListCount] = 1, activeCellStatusListCount++;

                                activeCellStatusList [activeCellStatusListCount] = 0, activeCellStatusListCount++;

                                activeCellStatusList [activeCellStatusListCount] = prevTDCount, activeCellStatusListCount++;

                                activeCellStatusList [activeCellStatusListCount] = prevTDTD, activeCellStatusListCount++;

                                activeCellStatusList [activeCellStatusListCount] = 0, activeCellStatusListCount++;

                                activeCellStatusList [activeCellStatusListCount] = tempListOfCells [counter1*7+1], activeCellStatusListCount++;

**if** (simStartModeHold == 0) activeCellStatusList [activeCellStatusListCount] = 0, activeCellStatusListCount++;

**else** activeCellStatusList [activeCellStatusListCount] = tempListOfCells [counter1*7+6], activeCellStatusListCount++;

                                activeCellStatusList [activeCellStatusListCount] = tempListOfCells [counter1*7+3], activeCellStatusListCount++;

                                activeCellStatusList [activeCellStatusListCount] = tempListOfCells [counter1*7+2], activeCellStatusListCount++;

                                activeCellStatusList [activeCellStatusListCount] = 0, activeCellStatusListCount++;

                                activeCellStatusList [activeCellStatusListCount] = tempListOfCells [counter1*7], activeCellStatusListCount++;

                                activeCellStatusList [activeCellStatusListCount] = 0, activeCellStatusListCount++;

                                activeCellStatusList [activeCellStatusListCount] = 1, activeCellStatusListCount++;

                                activeCellStatusList [activeCellStatusListCount] = 0, activeCellStatusListCount++;

                                activeCellStatusList [activeCellStatusListCount] = prevTDCount, activeCellStatusListCount++;

                                activeCellStatusList [activeCellStatusListCount] = prevTDTD, activeCellStatusListCount++;

                                activeCellStatusList [activeCellStatusListCount] = 0, activeCellStatusListCount++;

                                activeCellStatusList [activeCellStatusListCount] = tempListOfCells [counter1*7+1], activeCellStatusListCount++;

**if** (simStartModeHold == 0) activeCellStatusList [activeCellStatusListCount] = 0, activeCellStatusListCount++;

**else** activeCellStatusList [activeCellStatusListCount] = tempListOfCells [counter1*7+6], activeCellStatusListCount++;

                            }

**if** (tempListOfCells [counter1*7+5]== 41){

                                activeCellStatusList [activeCellStatusListCount] = tempListOfCells [counter1*7+2], activeCellStatusListCount++;

                                activeCellStatusList [activeCellStatusListCount] = tempListOfCells [counter1*7+3], activeCellStatusListCount++;

                                activeCellStatusList [activeCellStatusListCount] = tempListOfCells [counter1*7+4], activeCellStatusListCount++;

                                activeCellStatusList [activeCellStatusListCount] = tempListOfCells [counter1*7], activeCellStatusListCount++;

                                activeCellStatusList [activeCellStatusListCount] = 0, activeCellStatusListCount++;

                                activeCellStatusList [activeCellStatusListCount] = 2, activeCellStatusListCount++;

                                activeCellStatusList [activeCellStatusListCount] = 0, activeCellStatusListCount++;

                                activeCellStatusList [activeCellStatusListCount] = prevTDCount, activeCellStatusListCount++;

                                activeCellStatusList [activeCellStatusListCount] = prevTDTD, activeCellStatusListCount++;

                                activeCellStatusList [activeCellStatusListCount] = 0, activeCellStatusListCount++;

                                activeCellStatusList [activeCellStatusListCount] = tempListOfCells [counter1*7+1], activeCellStatusListCount++;

**if** (simStartModeHold == 0) activeCellStatusList [activeCellStatusListCount] = 0, activeCellStatusListCount++;

**else** activeCellStatusList [activeCellStatusListCount] = tempListOfCells [counter1*7+6], activeCellStatusListCount++;

                                activeCellStatusList [activeCellStatusListCount] = tempListOfCells [counter1*7+3], activeCellStatusListCount++;

                                activeCellStatusList [activeCellStatusListCount] = tempListOfCells [counter1*7+2], activeCellStatusListCount++;

                                activeCellStatusList [activeCellStatusListCount] = tempListOfCells [counter1*7+4], activeCellStatusListCount++;

                                activeCellStatusList [activeCellStatusListCount] = tempListOfCells [counter1*7], activeCellStatusListCount++;

                                activeCellStatusList [activeCellStatusListCount] = 0, activeCellStatusListCount++;

                                activeCellStatusList [activeCellStatusListCount] = 2, activeCellStatusListCount++;

                                activeCellStatusList [activeCellStatusListCount] = 0, activeCellStatusListCount++;

                                activeCellStatusList [activeCellStatusListCount] = prevTDCount, activeCellStatusListCount++;

                                activeCellStatusList [activeCellStatusListCount] = prevTDTD, activeCellStatusListCount++;

                                activeCellStatusList [activeCellStatusListCount] = 0, activeCellStatusListCount++;

                                activeCellStatusList [activeCellStatusListCount] = tempListOfCells [counter1*7+1], activeCellStatusListCount++;

**if** (simStartModeHold == 0) activeCellStatusList [activeCellStatusListCount] = 0, activeCellStatusListCount++;

**else** activeCellStatusList [activeCellStatusListCount] = tempListOfCells [counter1*7+6], activeCellStatusListCount++;

                                activeCellStatusList [activeCellStatusListCount] = tempListOfCells [counter1*7+4], activeCellStatusListCount++;

                                activeCellStatusList [activeCellStatusListCount] = tempListOfCells [counter1*7+2], activeCellStatusListCount++;

                                activeCellStatusList [activeCellStatusListCount] = tempListOfCells [counter1*7+3], activeCellStatusListCount++;

                                activeCellStatusList [activeCellStatusListCount] = tempListOfCells [counter1*7], activeCellStatusListCount++;

                                activeCellStatusList [activeCellStatusListCount] = 0, activeCellStatusListCount++;

                                activeCellStatusList [activeCellStatusListCount] = 2, activeCellStatusListCount++;

                                activeCellStatusList [activeCellStatusListCount] = 0, activeCellStatusListCount++;

                                activeCellStatusList [activeCellStatusListCount] = prevTDCount, activeCellStatusListCount++;

                                activeCellStatusList [activeCellStatusListCount] = prevTDTD, activeCellStatusListCount++;

                                activeCellStatusList [activeCellStatusListCount] = 0, activeCellStatusListCount++;

                                activeCellStatusList [activeCellStatusListCount] = tempListOfCells [counter1*7+1], activeCellStatusListCount++;

**if** (simStartModeHold == 0) activeCellStatusList [activeCellStatusListCount] = 0, activeCellStatusListCount++;

**else** activeCellStatusList [activeCellStatusListCount] = tempListOfCells [counter1*7+6], activeCellStatusListCount++;

                            }

                        }

                        //=====Remove info of cells, of which simulation has been completed, from the "activeCellStatusList"=====

**int** *activeCellStatusListTemp = **new** **int** [activeCellStatusListCount+100];

**int** activeCellStatusListTempCount = 0;

**for** (**int** counter1 = 0; counter1 < activeCellStatusListCount/12; counter1++){

**if** (activeCellStatusList [counter1*12+1] != -1){

                                activeCellStatusListTemp [activeCellStatusListTempCount] = activeCellStatusList [counter1*12], activeCellStatusListTempCount++;

                                activeCellStatusListTemp [activeCellStatusListTempCount] = activeCellStatusList [counter1*12+1], activeCellStatusListTempCount++;

                                activeCellStatusListTemp [activeCellStatusListTempCount] = activeCellStatusList [counter1*12+2], activeCellStatusListTempCount++;

                                activeCellStatusListTemp [activeCellStatusListTempCount] = activeCellStatusList [counter1*12+3], activeCellStatusListTempCount++;

                                activeCellStatusListTemp [activeCellStatusListTempCount] = activeCellStatusList [counter1*12+4], activeCellStatusListTempCount++;

                                activeCellStatusListTemp [activeCellStatusListTempCount] = activeCellStatusList [counter1*12+5], activeCellStatusListTempCount++;

                                activeCellStatusListTemp [activeCellStatusListTempCount] = activeCellStatusList [counter1*12+6], activeCellStatusListTempCount++;

                                activeCellStatusListTemp [activeCellStatusListTempCount] = activeCellStatusList [counter1*12+7], activeCellStatusListTempCount++;

                                activeCellStatusListTemp [activeCellStatusListTempCount] = activeCellStatusList [counter1*12+8], activeCellStatusListTempCount++;

                                activeCellStatusListTemp [activeCellStatusListTempCount] = activeCellStatusList [counter1*12+9], activeCellStatusListTempCount++;

                                activeCellStatusListTemp [activeCellStatusListTempCount] = activeCellStatusList [counter1*12+10], activeCellStatusListTempCount++;

                                activeCellStatusListTemp [activeCellStatusListTempCount] = activeCellStatusList [counter1*12+11], activeCellStatusListTempCount++;

                            }

                        }

                        activeCellStatusListCount = 0;

**for** (**int** counter1 = 0; counter1 < activeCellStatusListTempCount; counter1++) activeCellStatusList [activeCellStatusListCount] = activeCellStatusListTemp [counter1], activeCellStatusListCount++;

**delete** [] tempListOfCells;

**delete** [] activeCellStatusListTemp;

**delete** [] activeCellStatusListHold;

                        activeCellStatusListHold = **new** **int** [activeCellStatusListCount+10];

                        activeCellStatusListHoldCount = 0;

**for** (**int** counter1 = 0; counter1 < activeCellStatusListCount; counter1++) activeCellStatusListHold [activeCellStatusListHoldCount] = activeCellStatusList [counter1], activeCellStatusListHoldCount++;

                        //====Summary of Expand and % arrays====

                        //expandFirsDVList

                        //expandDoublingDoubBD

                        //expandDoublingDoubTD

                        //expandDoublingDoubCD

                        //expandBDCD

                        //expandBDCF

                        //expandNonCD

                        //expandBDCFCD

                        //expandTDCF

                        //expandTDCFCD

                        //expandTDCD

                        //firstEventList; percentBD //1, percentTD //2, percentCD //3, nonDivPercent //4

                        //secondEventBDList; percentBD //1, percentTD //2, percentBDCD //5, percentBDCF //6

                        //secondEventBDCFList; percentBDCFBD //7, percentBDCFTD //8, percentBDCFCD //9

                        //secondEventTDList; percentTDCF //10, percentTDBD //11, percentTDTD //12, percentTDCD //13

                        //secondEventTDCFList; percentTDCFBD //14, percentTDCFTD //15, percentTDCFCD //16

                        //=======The "SecondBD List"; After entry of initial simulation data, the frequencies of BD, TD, CD, and CF are readjusted.======

**int** totalEvent = (**int**)(round(simProcessDataBaseHold [3]+simProcessDataBaseHold [4]))+totalNoOfBDCD+totalNoOfBDCF;

**int** percentTotalBD = (**int**)(round(simProcessDataBaseHold [3]/(**double**)totalEvent));

**int** percentTotalTD = (**int**)(round(simProcessDataBaseHold [4]/(**double**)totalEvent));

**int** percentTotalCD = (**int**)(round(totalNoOfBDCD/(**double**)totalEvent));

**int** percentTotalCF = (**int**)(round(totalNoOfBDCF/(**double**)totalEvent));

**int** countBD = 0;

**int** countTD = 0;

**int** countCD = 0;

**int** countCF = 0;

**for** (**int** counter2 = 0; counter2 < 1000; counter2++){ //====Try max 100 times, and if not event is found, set BD====

                            randInit = rand() % 100 + 0;

**if** (secondEventBDList [randInit] == 1) countBD++;

**else** **if** (secondEventBDList [randInit] == 2) countTD++;

**else** **if** (secondEventBDList [randInit] == 5) countCD++;

**else** **if** (secondEventBDList [randInit] == 6) countCF++;

                        }

**int** totalCheck = countBD+countTD+countCD+countCF;

**int** percentCheckTD = 0;

**int** percentCheckCD = 0;

**int** percentCheckCF = 0;

**if** (totalCheck != 0){

                            percentCheckTD = (**int**)(round(countTD/(**double**)totalCheck));

                            percentCheckCD = (**int**)(round(countCD/(**double**)totalCheck));

                            percentCheckCF = (**int**)(round(countCF/(**double**)totalCheck));

**int** checkCount = 0;

**if** (percentCheckTD-percentTotalTD < 0 && percentTotalTD-percentCheckTD < percentTotalBD){

                                checkCount = percentTotalTD-percentCheckTD;

**for** (**int** counter2 = 0; counter2 < 100; counter2++){

**if** (secondEventBDList [counter2] == 1){

                                        secondEventBDList [counter2] = 2;

                                        checkCount--;

**if** (checkCount == 0){

**break**;

                                        }

                                    }

                                }

                            }

**if** (percentCheckCD-percentTotalCD < 0 && percentTotalCD-percentCheckCD < percentTotalBD-(percentTotalTD-percentCheckTD)){

                                checkCount = percentTotalCD-percentCheckCD;

**for** (**int** counter2 = 0; counter2 < 100; counter2++){

**if** (secondEventBDList [counter2] == 1){

                                        secondEventBDList [counter2] = 5;

                                        checkCount--;

**if** (checkCount == 0){

**break**;

                                        }

                                    }

                                }

                            }

**if** (percentCheckCF-percentTotalCF < 0 && percentTotalCF-percentCheckCF < percentTotalBD-(percentTotalTD-percentCheckTD)-(percentTotalCD-percentCheckCD)){

                                checkCount = percentTotalCF-percentCheckCF;

**for** (**int** counter2 = 0; counter2 < 100; counter2++){

**if** (secondEventBDList [counter2] == 1){

                                        secondEventBDList [counter2] = 6;

                                        checkCount--;

**if** (checkCount == 0){

**break**;

                                        }

                                    }

                                }

                            }

                        }

**if** (doseSimStatusHold == 2){ //====For MX or MSC mode====

                            totalEvent = (**int**)(round(simProcessDataProgHold [3]+simProcessDataProgHold [4]))+totalNoOfBDCD+totalNoOfBDCF;

                            percentTotalBD = (**int**)(round(simProcessDataProgHold [3]/(**double**)totalEvent));

                            percentTotalTD = (**int**)(round(simProcessDataProgHold [4]/(**double**)totalEvent));

                            percentTotalCD = (**int**)(round(totalNoOfBDCD/(**double**)totalEvent));

                            percentTotalCF = (**int**)(round(totalNoOfBDCF/(**double**)totalEvent));

                            countBD = 0;

                            countTD = 0;

                            countCD = 0;

                            countCF = 0;

**for** (**int** counter2 = 0; counter2 < 1000; counter2++){ //====Try max 100 times, and if not event is found, set BD====

                                randInit = rand() % 100 + 0;

**if** (secondEventBDListSel [randInit] == 1) countBD++;

**else** **if** (secondEventBDListSel [randInit] == 2) countTD++;

**else** **if** (secondEventBDListSel [randInit] == 5) countCD++;

**else** **if** (secondEventBDListSel [randInit] == 6) countCF++;

                            }

                            totalCheck = countBD+countTD+countCD+countCF;

                            percentCheckTD = 0;

                            percentCheckCD = 0;

                            percentCheckCF = 0;

**if** (totalCheck != 0){

                                percentCheckTD = (**int**)(round(countTD/(**double**)totalCheck));

                                percentCheckCD = (**int**)(round(countCD/(**double**)totalCheck));

                                percentCheckCF = (**int**)(round(countCF/(**double**)totalCheck));

**int** checkCount = 0;

**if** (percentCheckTD-percentTotalTD < 0 && percentTotalTD-percentCheckTD < percentTotalBD){

                                    checkCount = percentTotalTD-percentCheckTD;

**for** (**int** counter2 = 0; counter2 < 100; counter2++){

**if** (secondEventBDListSel [counter2] == 1){

                                            secondEventBDListSel [counter2] = 2;

                                            checkCount--;

**if** (checkCount == 0){

**break**;

                                            }

                                        }

                                    }

                                }

**if** (percentCheckCD-percentTotalCD < 0 && percentTotalCD-percentCheckCD < percentTotalBD-(percentTotalTD-percentCheckTD)){

                                    checkCount = percentTotalCD-percentCheckCD;

**for** (**int** counter2 = 0; counter2 < 100; counter2++){

**if** (secondEventBDListSel [counter2] == 1){

                                            secondEventBDListSel [counter2] = 5;

                                            checkCount--;

**if** (checkCount == 0){

**break**;

                                            }

                                        }

                                    }

                                }

**if** (percentCheckCF-percentTotalCF < 0 && percentTotalCF-percentCheckCF < percentTotalBD-(percentTotalTD-percentCheckTD)-(percentTotalCD-percentCheckCD)){

                                    checkCount = percentTotalCF-percentCheckCF;

**for** (**int** counter2 = 0; counter2 < 100; counter2++){

**if** (secondEventBDListSel [counter2] == 1){

                                            secondEventBDListSel [counter2] = 6;

                                            checkCount--;

**if** (checkCount == 0){

**break**;

                                            }

                                        }

                                    }

                                }

                            }

                        }

                        //******Continue the creation of cell lineage data till the "activeCellStatusList" become 0.*******

**int** terminationFlag = 0;

**int** lowextValue = 0;

**int** highestValue = 0;

**int** eventCount = 0;

**int** eventType = 0;

**int** loopCheck = 0;

**int** siblinFusionCheck1 = 0;

**int** siblinFusionCheck2 = 0;

**int** siblinFusionDoub1 = 0;

**int** siblinFusionDoub2 = 0;

**int** siblinFusionDoubLarge = 0;

**int** siblingCellNo1 = 0;

**int** siblingCellNo2 = 0;

**int** dataTempInt = 0;

**int** cellDoubLimitCheck = 0;

**int** siblingCellPosition1 = 0;

**int** siblingCellPosition2 = 0;

**int** siblingCellPositionSelect = 0;

**int** siblingCellNoSelect = 0;

**int** siblingCellDoubSelect = 0;

**int** lineageAddTempCount = 0;

**int** lineageAddTempLimit = 0;

**int** parentTDCount = 0;

**int** parentTDTD = 0;

**int** parentTDBD = 0;

**int** fusionCount = 0;

**int** reachMaxDivision = 1000000000;

**int** timeKeep = 0;

**int** arrayOverflow = 0;

**int** newLimit = 0;

**int** startPositionListCount = 0;

**int** cellNoFusionCheckHold = 0;

**int** missingPartnerCheck = 0;

**int** selectChange = 0;

**int** loopCount = 0;

**int** terminate2 = 0;

**int** cellNoforSummary = 0;

**int** clingNoforSummary = 0;

**int** fusionForSummary = 0;

**int** cycleMaxReachFlag = 0;

**unsigned** **long** setTime = 0;

**double** dataTempDouble = 0;

**double** dataTempDouble2 = 0;

**double** dataTempDouble3 = 0;

**double** duration = 0;

**double** targetAdjust = 0;

**unsigned** **long** fusionPartnerListCount = 0;

**unsigned** **long** entryCount2 = 0;

                        string cellNumberString;

                        //****The main loop for the simulation******

**do**{

                            //====For display current processing status====

                            processingStatusCall = 1;

                            loopCount++;

                            processingStatus = "Base:C"+to_string (loopCount);

                            terminationFlag = 1;

                            fusionCount = 0;

**for** (**int** counter1 = 0; counter1 < activeCellStatusListCount/12; counter1++){

**if** (terminateSimFlag == 1){

                                    terminate2 = 1;

**break**;

                                }

                                selectCheck = 0;

**if** (doseSimStatusHold == 2){

**for** (**int** counter3 = 0; counter3 < lingNoAssigineSimCount; counter3++){

**if** (lingNoAssigineSim [counter3] == activeCellStatusList [counter1*12+3]){

                                            selectCheck = 1;

**break**;

                                        }

                                    }

                                }

                                cellDoubLimitCheck = activeCellStatusList [counter1*12];

**if** (cellDoubLimitCheck < 0) cellDoubLimitCheck = cellDoubLimitCheck*-1;

                                cellNumberString = to_string(cellDoubLimitCheck);

                                //********Assign the length of time to an event and event type to each cell.******

                                //=====If cell division reaches 15, the "growthCycleBase" will be changed to the shortest time that reaches 15 division+max time point.====

**if** (((**int**)cellNumberString.length() == 9 && (cellNumberString.substr(0, 1) == "5" || cellNumberString.substr(0, 1) == "6") && cellNumberString.substr(cellNumberString.length()-1) != "0") || cycleMaxReachFlag == 1){

                                    activeCellStatusList [counter1*12+4] = 50; //====When cell division reaches 15, a 100-time point will be added, and then terminate the simulation of the cell.====

                                    activeCellStatusList [counter1*12+5] = 9;

                                    activeCellStatusList [counter1*12+6] = 1;

                                    cycleMaxReachFlag = 1;

                                }

**else**{

**if** (selectCheck == 0){

**if** (activeCellStatusList [counter1*12+5] == 4){ //=====NonDiv=====

**if** (doseSimStatusHold == 0){

**if** (simProcessDataBaseHold [21]/(**double**)simProcessDataBaseHold [24] > 1.0) dataTempDouble = 1;

**else** dataTempDouble = simProcessDataBaseHold [21]/(**double**)simProcessDataBaseHold [24];

                                            }

**else**{

**if** (doseMiddleHold-doseBaseHold > 0){

                                                    duration = doseMiddleHold-doseBaseHold;

                                                    targetAdjust = doseTargetHold-doseBaseHold;

                                                }

**else**{

                                                    duration = doseBaseHold-doseMiddleHold;

                                                    targetAdjust = (doseTargetHold-doseBaseHold)*-1;

                                                }

                                                dataTempDouble2 = simProcessDataBaseHold [21]+((simProcessDataMiddleHold [21]-simProcessDataBaseHold [21])/(**double**)duration)*targetAdjust;

                                                dataTempDouble3 = simProcessDataBaseHold [24]+((simProcessDataMiddleHold [24]-simProcessDataBaseHold [24])/(**double**)duration)*targetAdjust;

**if** (dataTempDouble2/(**double**)dataTempDouble3 > 1.0) dataTempDouble = 1;

**else** dataTempDouble = dataTempDouble2/(**double**)dataTempDouble3;

                                            }

                                            randInit = rand() % 100 + 0;

                                            //======Determine whether Recovery should apply; if the result of the "rand" is less than Recovery%, "recovery" will be applied. If not, extend the time point or assign CD; Recovery applies only once. after applying recovery, set 7 (BD after recovery to adjust the length of cell doubling time======

**if** (randInit < dataTempDouble*100){

                                                lowextValue = 100000;

                                                highestValue = 0;

                                                //======Use the "expandDoublingDoubBD" to determine max and min doubling time=====

**for** (**int** counter2 = 0; counter2 < expandDoublingDoubBDCount/2; counter2++){

**if** (lowextValue > expandDoublingDoubBD [counter2*2]) lowextValue = expandDoublingDoubBD [counter2*2];

**if** (highestValue < expandDoublingDoubBD [counter2*2]) highestValue = expandDoublingDoubBD [counter2*2];

                                                }

                                                //=====The first doubling time is 80% of the "timePointMax"=====

**if** (simProcessDataBaseHold [25] < lowextValue){

                                                    activeCellStatusList [counter1*12+4] = (**int**)(round((highestValue+lowextValue)/(**double**)2));

                                                    activeCellStatusList [counter1*12+5] = 70; //=====BD flag for recovery; assign the lowest value of BD if the value is below the lowest doubling time=====

                                                    activeCellStatusList [counter1*12+6] = 1;

                                                }

**else**{

**if** (highestValue != 0){

**if** (highestValue-10 > lowextValue) dataTempInt = highestValue-10;

**else** **if** (highestValue-10 <= lowextValue) dataTempInt = highestValue;

                                                    }

**else**{

**if** (simProcessDataBaseHold [25]-10 > (**int**)(round(simProcessDataBaseHold [25]*(**double**)0.8))) dataTempInt = (**int**)simProcessDataBaseHold [25]-10;

**else** dataTempInt = (**int**)(round(simProcessDataBaseHold [25]*(**double**)0.8));

                                                    }

                                                    activeCellStatusList [counter1*12+4] = dataTempInt; //=====BD flag for recovery; set 80% of end-time or highest=====

                                                    activeCellStatusList [counter1*12+5] = 70;

                                                    activeCellStatusList [counter1*12+6] = 1;

                                                }

                                            }

**else**{

                                                dataTempDouble = ((expandNonCDCount/(**double**)2)/(**double**)simProcessDataBaseHold [24])*100; //=====Calculate % of CD occurrence=====

                                                randInit = rand() % 100 + 0;

**if** (randInit < dataTempDouble && expandNonCDCount != 0){ //=====CD occurence %=====

                                                    randInit = rand() % expandNonCDCount/2 + 0;

                                                    activeCellStatusList [counter1*12+4] = expandNonCD [randInit*2]; //=====After set CD, determine the time that CD occurs======

                                                    activeCellStatusList [counter1*12+5] = 30;

                                                    activeCellStatusList [counter1*12+6] = 1;

                                                }

**else**{

                                                    randInit2 = rand() % (**int**)(round(simProcessDataBaseHold [25]*(endVariationtHold/(**double**)100))) + 0;

**if** (randInit2%2 == 0) activeCellStatusList [counter1*12+4] = (**int**)simProcessDataBaseHold [25]+randInit2; //=====Set 5: no recovery will be applied.=====

**else** activeCellStatusList [counter1*12+4] = (**int**)simProcessDataBaseHold [25]-randInit2;

                                                    activeCellStatusList [counter1*12+5] = 5;

                                                    activeCellStatusList [counter1*12+6] = 1;

                                                }

                                            }

                                        }

**else** **if** (activeCellStatusList [counter1*12+5] == 5){ //=====Either set CD or extend new "End time"=====

                                            dataTempDouble = ((expandNonCDCount/(**double**)2)/(**double**)simProcessDataBaseHold [24])*100; //=====Calculate % of CD occurrence=====

                                            randInit = rand() % 100 + 0;

**if** (randInit < dataTempDouble && expandNonCDCount != 0){ //=====Set 10% limit=====

                                                randInit = rand() % expandNonCDCount/2 + 0;

                                                activeCellStatusList [counter1*12+4] = expandNonCD [randInit*2];

                                                activeCellStatusList [counter1*12+5] = 30;

                                                activeCellStatusList [counter1*12+6] = 1;

                                            }

**else**{

                                                randInit2 = rand() % (**int**)(round(simProcessDataBaseHold [25]*(endVariationtHold/(**double**)100))) + 0;

**if** (randInit2%2 == 0) activeCellStatusList [counter1*12+4] = (**int**)simProcessDataBaseHold [25]+randInit2; //=====Set 5: no recovery will be applied=====

**else** activeCellStatusList [counter1*12+4] = (**int**)simProcessDataBaseHold [25]-randInit2;

                                                activeCellStatusList [counter1*12+5] = 5;

                                                activeCellStatusList [counter1*12+6] = 1;

                                            }

                                        }

**else** **if** (activeCellStatusList [counter1*12+5] == 7){ //=====BD for recovery=====

                                            eventType = 1; //=====BD recovery: set BD for longer doubling time. The next round will be the normal BD or 7=====

**if** (eventType == 1){

                                                //=====Use the "expandDoublingDoubBD", Set BD=====

**if** (activeCellStatusList [counter1*12+11] == 0){

**if** (expandDoublingDoubBDCount != 0){

                                                        randInit = rand() % expandDoublingDoubBDCount/2 + 0;

                                                        activeCellStatusList [counter1*12+4] = expandDoublingDoubBD [randInit*2];

                                                        activeCellStatusList [counter1*12+5] = 1;

                                                        activeCellStatusList [counter1*12+6] = 1;

                                                    }

**else**{

                                                        randInit = rand() % randBDRangeB + randBDRangeA;

                                                        activeCellStatusList [counter1*12+4] = randInit;

                                                        activeCellStatusList [counter1*12+5] = 7;

                                                        activeCellStatusList [counter1*12+6] = 1;

                                                    }

                                                }

**else**{

                                                    loopCheck = 0;

**for** (**int** counter2 = 0; counter2 < 100; counter2++){

**if** (expandDoublingDoubBDCount != 0){

                                                            randInit = rand() % expandDoublingDoubBDCount/2 + 0;

**if** (expandDoublingDoubBD [randInit*2] > activeCellStatusList [counter1*12+11]-50 && expandDoublingDoubBD [randInit*2] < activeCellStatusList [counter1*12+11]+50){

                                                                activeCellStatusList [counter1*12+4] = expandDoublingDoubBD [randInit*2];

                                                                activeCellStatusList [counter1*12+5] = 1;

                                                                activeCellStatusList [counter1*12+6] = 1;

                                                                loopCheck = 1;

**break**;

                                                            }

                                                        }

                                                    }

**if** (loopCheck == 0){

                                                        activeCellStatusList [counter1*12+4] = activeCellStatusList [counter1*12+11];

                                                        activeCellStatusList [counter1*12+5] = 7;

                                                        activeCellStatusList [counter1*12+6] = 1;

                                                    }

                                                }

                                            }

                                        }

**else** **if** (activeCellStatusList [counter1*12+5] == 1){ //=====BD=====

                                            siblinFusionCheck1 = 0;

                                            siblinFusionDoub1 = 0;

                                            siblingCellNo1 = 0;

                                            //====Check the status of sibling=====

**for** (**int** counter2 = 0; counter2 < activeCellStatusListCount/12; counter2++){

**if** (activeCellStatusList [counter2*12+6] == 1 && activeCellStatusList [counter2*12+1] == activeCellStatusList [counter1*12] && activeCellStatusList [counter2*12+3] == activeCellStatusList [counter1*12+3]){

                                                    siblingCellNo1 = counter2;

**if** (activeCellStatusList [counter2*12+5] != 0){

                                                        siblinFusionCheck1 = activeCellStatusList [counter2*12+5];

                                                        siblinFusionDoub1 = activeCellStatusList [counter2*12+4];

                                                    }

**break**;

                                                }

                                            }

                                            //====Event type assignment====

                                            eventType = 0;

**if** (activeCellStatusList [counter1*12+9] == 0){ //=====Limit off======

**if** (siblinFusionCheck1 == 6){ //=====If CF is set to another sibling, assign an event other than CF=====

                                                    loopCheck = 0;

**for** (**int** counter2 = 0; counter2 < 100; counter2++){ //=====Search max 100 times. If no CF is found, set BD=====

                                                        randInit = rand() % 100 + 0;

**if** (secondEventBDList [randInit] != 6){

                                                            eventType = secondEventBDList [randInit];

                                                            loopCheck = 1;

**break**;

                                                        }

                                                    }

**if** (loopCheck == 0) eventType = 1;

                                                }

**else**{

                                                    randInit = rand() % 100 + 0;

**if** (secondEventBDList [randInit] != 0) eventType = secondEventBDList [randInit];

**else** eventType = 1;

                                                }

                                            }

**else** **if** (activeCellStatusList [counter1*12+9] != 0){ //=====Limit on=====

                                                eventCount = 0;

                                                eventType = 0;

**if** (siblinFusionCheck1 == 6) eventType = 5; //=====If CF is set to another sibling, set CD=====

**else**{

**for** (**int** counter2 = 0; counter2 < 5; counter2++){ //=====If BD is selected five times, set BD=====

                                                        randInit = rand() % 100 + 0;

**if** (secondEventBDList [randInit] == 1) eventCount++;

**else**{

                                                            eventType = secondEventBDList [randInit];

**break**;

                                                        }

                                                    }

**if** (eventCount == 5 || eventType == 0) eventType = 1;

                                                }

                                            }

**if** (eventType == 1){

                                                //=====Use the "expandDoublingDoubBD", Set BD=====

**if** (activeCellStatusList [counter1*12+11] == 0){

**if** (siblinFusionCheck1 != 6){

**if** (expandDoublingDoubBDCount != 0){

                                                            randInit = rand() % expandDoublingDoubBDCount/2 + 0;

                                                            activeCellStatusList [counter1*12+4] = expandDoublingDoubBD [randInit*2];

                                                            activeCellStatusList [counter1*12+5] = 1;

                                                            activeCellStatusList [counter1*12+6] = 1;

**if** (activeCellStatusList [counter1*12+9] != 0) activeCellStatusList [counter1*12+9]--;

                                                        }

**else**{

                                                            randInit = rand() % randBDRangeB + randBDRangeA;

                                                            activeCellStatusList [counter1*12+4] = randInit;

                                                            activeCellStatusList [counter1*12+5] = 1;

                                                            activeCellStatusList [counter1*12+6] = 1;

**if** (activeCellStatusList [counter1*12+9] != 0) activeCellStatusList [counter1*12+9]--;

                                                        }

                                                    }

**else**{

**if** (siblinFusionDoub1 < randBDRangeA) siblinFusionDoub1 = randBDRangeA;

                                                        activeCellStatusList [counter1*12+4] = siblinFusionDoub1+10;

                                                        activeCellStatusList [counter1*12+5] = 1;

                                                        activeCellStatusList [counter1*12+6] = 1;

**if** (activeCellStatusList [counter1*12+9] != 0) activeCellStatusList [counter1*12+9]--;

                                                    }

                                                }

**else**{

                                                    loopCheck = 0;

**for** (**int** counter2 = 0; counter2 < 100; counter2++){ //=====Doubling time bias: +- 50-time point of the previous doubling time=====

**if** (siblinFusionCheck1 != 6){ //=====Set BD except for one, of which sibling undergoes CF=====

**if** (expandDoublingDoubBDCount != 0){

                                                                randInit = rand() % expandDoublingDoubBDCount/2 + 0;

**if** (expandDoublingDoubBD [randInit*2] > activeCellStatusList [counter1*12+11]-50 && expandDoublingDoubBD [randInit*2] < activeCellStatusList [counter1*12+11]+50){

                                                                    activeCellStatusList [counter1*12+4] = expandDoublingDoubBD [randInit*2];

                                                                    activeCellStatusList [counter1*12+5] = 1;

                                                                    activeCellStatusList [counter1*12+6] = 1;

**if** (activeCellStatusList [counter1*12+9] != 0) activeCellStatusList [counter1*12+9]--;

                                                                    loopCheck = 1;

**break**;

                                                                }

                                                            }

                                                        }

**else** **if** (siblinFusionCheck1 == 6){

**if** (expandDoublingDoubBDCount != 0){

                                                                randInit = rand() % expandDoublingDoubBDCount/2 + 0;

**if** (expandDoublingDoubBD [randInit*2] > activeCellStatusList [counter1*12+11]-50 && expandDoublingDoubBD [randInit*2] < activeCellStatusList [counter1*12+11]+50 && siblinFusionDoub1+10 < expandDoublingDoubBD [randInit*2]){

                                                                    activeCellStatusList [counter1*12+4] = expandDoublingDoubBD [randInit*2];

                                                                    activeCellStatusList [counter1*12+5] = 1;

                                                                    activeCellStatusList [counter1*12+6] = 1;

**if** (activeCellStatusList [counter1*12+9] != 0) activeCellStatusList [counter1*12+9]--;

                                                                    loopCheck = 1;

**break**;

                                                                }

                                                            }

                                                        }

                                                    }

**if** (loopCheck == 0){ //=====If no match is found, set the previous doubling time=====

                                                        lowextValue = 100000;

                                                        highestValue = 0;

**for** (**int** counter2 = 0; counter2 < expandDoublingDoubBDCount/2; counter2++){

**if** (lowextValue > expandDoublingDoubBD [counter2*2]) lowextValue = expandDoublingDoubBD [counter2*2];

**if** (highestValue < expandDoublingDoubBD [counter2*2]) highestValue = expandDoublingDoubBD [counter2*2];

                                                        }

**if** (siblinFusionDoub1 < randBDRangeA) siblinFusionDoub1 = randBDRangeA;

**if** (lowextValue != 100000 && highestValue != 0){

**if** ((highestValue+lowextValue)/(**double**)2 > siblinFusionDoub1+10){

                                                                activeCellStatusList [counter1*12+4] = (**int**)(round((highestValue+lowextValue)/(**double**)2));

                                                                activeCellStatusList [counter1*12+5] = 1;

                                                                activeCellStatusList [counter1*12+6] = 1;

**if** (activeCellStatusList [counter1*12+9] != 0) activeCellStatusList [counter1*12+9]--;

                                                            }

**else**{

                                                                activeCellStatusList [counter1*12+4] = siblinFusionDoub1+10;

                                                                activeCellStatusList [counter1*12+5] = 1;

                                                                activeCellStatusList [counter1*12+6] = 1;

**if** (activeCellStatusList [counter1*12+9] != 0) activeCellStatusList [counter1*12+9]--;

                                                            }

                                                        }

**else**{

                                                            activeCellStatusList [counter1*12+4] = siblinFusionDoub1+10;

                                                            activeCellStatusList [counter1*12+5] = 1;

                                                            activeCellStatusList [counter1*12+6] = 1;

**if** (activeCellStatusList [counter1*12+9] != 0) activeCellStatusList [counter1*12+9]--;

                                                        }

                                                    }

                                                }

                                            }

**else** **if** (eventType == 2){

                                                //=====Use the "expandDoublingDoubBD", Set TD=====

**if** (activeCellStatusList [counter1*12+11] == 0){

**if** (siblinFusionCheck1 != 6){

**if** (expandDoublingDoubBDCount != 0){

                                                            randInit = rand() % expandDoublingDoubBDCount/2 + 0;

                                                            activeCellStatusList [counter1*12+4] = expandDoublingDoubBD [randInit*2];

                                                            activeCellStatusList [counter1*12+5] = 2;

                                                            activeCellStatusList [counter1*12+6] = 1;

                                                            activeCellStatusList [counter1*12+7] = 1;

                                                            activeCellStatusList [counter1*12+9] = 0;

                                                            activeCellStatusList [counter1*12+8] = (**int**)simProcessDataBaseHold [18];

                                                        }

**else**{

                                                            randInit = rand() % randBDRangeB + randBDRangeA;

                                                            activeCellStatusList [counter1*12+4] = randInit;

                                                            activeCellStatusList [counter1*12+5] = 2;

                                                            activeCellStatusList [counter1*12+6] = 1;

                                                            activeCellStatusList [counter1*12+7] = 1;

                                                            activeCellStatusList [counter1*12+9] = 0;

                                                            activeCellStatusList [counter1*12+8] = (**int**)simProcessDataBaseHold [18];

                                                        }

                                                    }

**else**{

**if** (siblinFusionDoub1 < randBDRangeA) siblinFusionDoub1 = randBDRangeA;

                                                        activeCellStatusList [counter1*12+4] = siblinFusionDoub1+10;

                                                        activeCellStatusList [counter1*12+5] = 2;

                                                        activeCellStatusList [counter1*12+6] = 1;

                                                        activeCellStatusList [counter1*12+7] = 1;

                                                        activeCellStatusList [counter1*12+9] = 0;

                                                        activeCellStatusList [counter1*12+8] = (**int**)simProcessDataBaseHold [18];

                                                    }

                                                }

**else**{

                                                    loopCheck = 0;

**for** (**int** counter2 = 0; counter2 < 100; counter2++){

**if** (siblinFusionCheck1 != 6){

**if** (expandDoublingDoubBDCount != 0){

                                                                randInit = rand() % expandDoublingDoubBDCount/2 + 0;

**if** (expandDoublingDoubBD [randInit*2] > activeCellStatusList [counter1*12+11]-50 && expandDoublingDoubBD [randInit*2] < activeCellStatusList [counter1*12+11]+50){

                                                                    activeCellStatusList [counter1*12+4] = expandDoublingDoubBD [randInit*2];

                                                                    activeCellStatusList [counter1*12+5] = 2;

                                                                    activeCellStatusList [counter1*12+6] = 1;

                                                                    activeCellStatusList [counter1*12+7] = 1;

                                                                    activeCellStatusList [counter1*12+9] = 0;

                                                                    activeCellStatusList [counter1*12+8] = (**int**)simProcessDataBaseHold [18];

                                                                    loopCheck = 1;

**break**;

                                                                }

                                                            }

                                                        }

**else** **if** (siblinFusionCheck1 == 6){

**if** (expandDoublingDoubBDCount != 0){

                                                                randInit = rand() % expandDoublingDoubBDCount/2 + 0;

**if** (expandDoublingDoubBD [randInit*2] > activeCellStatusList [counter1*12+11]-50 && expandDoublingDoubBD [randInit*2] < activeCellStatusList [counter1*12+11]+50 && siblinFusionDoub1+10 < expandDoublingDoubBD [randInit*2]){

                                                                    activeCellStatusList [counter1*12+4] = expandDoublingDoubBD [randInit*2];

                                                                    activeCellStatusList [counter1*12+5] = 2;

                                                                    activeCellStatusList [counter1*12+6] = 1;

                                                                    activeCellStatusList [counter1*12+7] = 1;

                                                                    activeCellStatusList [counter1*12+9] = 0;

                                                                    activeCellStatusList [counter1*12+8] = (**int**)simProcessDataBaseHold [18];

                                                                    loopCheck = 1;

**break**;

                                                                }

                                                            }

                                                        }

                                                    }

**if** (loopCheck == 0){

                                                        lowextValue = 100000;

                                                        highestValue = 0;

**for** (**int** counter2 = 0; counter2 < expandDoublingDoubBDCount/2; counter2++){

**if** (lowextValue > expandDoublingDoubBD [counter2*2]) lowextValue = expandDoublingDoubBD [counter2*2];

**if** (highestValue < expandDoublingDoubBD [counter2*2]) highestValue = expandDoublingDoubBD [counter2*2];

                                                        }

**if** (siblinFusionDoub1 < randBDRangeA) siblinFusionDoub1 = randBDRangeA;

**if** (lowextValue != 100000 && highestValue != 0){

**if** ((highestValue+lowextValue)/(**double**)2 > siblinFusionDoub1+10){

                                                                activeCellStatusList [counter1*12+4] = (**int**)(round((highestValue+lowextValue)/(**double**)2));

                                                                activeCellStatusList [counter1*12+5] = 2;

                                                                activeCellStatusList [counter1*12+6] = 1;

                                                                activeCellStatusList [counter1*12+7] = 1;

                                                                activeCellStatusList [counter1*12+9] = 0;

                                                                activeCellStatusList [counter1*12+8] = (**int**)simProcessDataBaseHold [18];

                                                            }

**else**{

                                                                activeCellStatusList [counter1*12+4] = siblinFusionDoub1+10;

                                                                activeCellStatusList [counter1*12+5] = 2;

                                                                activeCellStatusList [counter1*12+6] = 1;

                                                                activeCellStatusList [counter1*12+7] = 1;

                                                                activeCellStatusList [counter1*12+9] = 0;

                                                                activeCellStatusList [counter1*12+8] = (**int**)simProcessDataBaseHold [18];

                                                            }

                                                        }

**else**{

                                                            activeCellStatusList [counter1*12+4] = siblinFusionDoub1+10;

                                                            activeCellStatusList [counter1*12+5] = 2;

                                                            activeCellStatusList [counter1*12+6] = 1;

                                                            activeCellStatusList [counter1*12+7] = 1;

                                                            activeCellStatusList [counter1*12+9] = 0;

                                                            activeCellStatusList [counter1*12+8] = (**int**)simProcessDataBaseHold [18];

                                                        }

                                                    }

                                                }

                                            }

**else** **if** (eventType == 5){

                                                //=====Use the "expandBDCD", Set CD=====

**if** (expandBDCDCount == 0){

                                                    randInit = rand() % randCDRangeB + randCDRangeA;

**if** (siblinFusionCheck1 != 6){

                                                        activeCellStatusList [counter1*12+4] = randInit;

                                                        activeCellStatusList [counter1*12+5] = 3;

                                                        activeCellStatusList [counter1*12+6] = 1;

                                                        activeCellStatusList [counter1*12+9] = 0;

                                                    }

**else**{

                                                        activeCellStatusList [counter1*12+4] = siblinFusionDoub1+10;

                                                        activeCellStatusList [counter1*12+5] = 3;

                                                        activeCellStatusList [counter1*12+6] = 1;

                                                        activeCellStatusList [counter1*12+9] = 0;

                                                    }

                                                }

**else**{

**if** (siblinFusionCheck1 != 6){

**if** (expandBDCDCount != 0){

                                                            randInit = rand() % expandBDCDCount/2 + 0;

                                                            activeCellStatusList [counter1*12+4] = expandBDCD [randInit*2];

                                                            activeCellStatusList [counter1*12+5] = 3;

                                                            activeCellStatusList [counter1*12+6] = 1;

                                                            activeCellStatusList [counter1*12+9] = 0;

                                                        }

**else**{

                                                            activeCellStatusList [counter1*12+4] = siblinFusionDoub1+10;

                                                            activeCellStatusList [counter1*12+5] = 3;

                                                            activeCellStatusList [counter1*12+6] = 1;

                                                            activeCellStatusList [counter1*12+9] = 0;

                                                        }

                                                    }

**else**{

**if** (expandBDCDCount != 0){

                                                            randInit = rand() % expandBDCDCount/2 + 0;

**if**(expandBDCD [randInit*2] > siblinFusionDoub1){

                                                                activeCellStatusList [counter1*12+4] = expandBDCD [randInit*2]+10;

                                                                activeCellStatusList [counter1*12+5] = 3;

                                                                activeCellStatusList [counter1*12+6] = 1;

                                                                activeCellStatusList [counter1*12+9] = 0;

                                                            }

**else**{

                                                                activeCellStatusList [counter1*12+4] = siblinFusionDoub1+10;

                                                                activeCellStatusList [counter1*12+5] = 3;

                                                                activeCellStatusList [counter1*12+6] = 1;

                                                                activeCellStatusList [counter1*12+9] = 0;

                                                            }

                                                        }

**else**{

                                                            activeCellStatusList [counter1*12+4] = siblinFusionDoub1+10;

                                                            activeCellStatusList [counter1*12+5] = 3;

                                                            activeCellStatusList [counter1*12+6] = 1;

                                                            activeCellStatusList [counter1*12+9] = 0;

                                                        }

                                                    }

                                                }

                                            }

**else** **if** (eventType == 6){

                                                //=====Use the "expandBDCF", Set CF=====

**if** (expandBDCFCount == 0){

                                                    randInit = rand() % randCDRangeB + randCDRangeA; //=====Use CD data=======

**if** (siblinFusionCheck1 != 6){

                                                        activeCellStatusList [counter1*12+4] = randInit;

                                                        activeCellStatusList [counter1*12+5] = 6;

                                                        activeCellStatusList [counter1*12+6] = 1;

                                                        activeCellStatusList [counter1*12+9] = 0;

                                                    }

**else**{

**if** (siblinFusionDoub1 < randBDRangeA) siblinFusionDoub1 = randBDRangeA;

                                                        activeCellStatusList [siblingCellNo1*12+4] = siblinFusionDoub1+10;

                                                        activeCellStatusList [counter1*12+4] = siblinFusionDoub1; //=====Switch assigned time length=====

                                                        activeCellStatusList [counter1*12+5] = 6;

                                                        activeCellStatusList [counter1*12+6] = 1;

                                                        activeCellStatusList [counter1*12+9] = 0;

                                                    }

                                                }

**else**{

**if** (siblinFusionCheck1 == 0){ //=====Set CF=====

**if** (expandBDCFCount != 0){

                                                            randInit = rand() % expandBDCFCount/2 + 0;

                                                            activeCellStatusList [counter1*12+4] = expandBDCF [randInit*2];

                                                            activeCellStatusList [counter1*12+5] = 6;

                                                            activeCellStatusList [counter1*12+6] = 1;

                                                            activeCellStatusList [counter1*12+9] = 0;

                                                        }

**else** {

                                                            activeCellStatusList [counter1*12+4] = siblinFusionDoub1+10;

                                                            activeCellStatusList [counter1*12+5] = 6;

                                                            activeCellStatusList [counter1*12+6] = 1;

                                                            activeCellStatusList [counter1*12+9] = 0;

                                                        }

                                                    }

**else**{ //=====In the case that an event is set to another sibling (BD, TD, or CD), recheck doubling time=====

**if** (expandBDCDCount != 0){

                                                            randInit = rand() % expandBDCDCount/2 + 0;

**if**(expandBDCD [randInit*2] > siblinFusionDoub1){

                                                                activeCellStatusList [counter1*12+4] = expandBDCD [randInit*2]+10;

                                                                activeCellStatusList [counter1*12+5] = 3;

                                                                activeCellStatusList [counter1*12+6] = 1;

                                                                activeCellStatusList [counter1*12+9] = 0;

                                                            }

**else**{

                                                                activeCellStatusList [counter1*12+4] = siblinFusionDoub1+10;

                                                                activeCellStatusList [counter1*12+5] = 3;

                                                                activeCellStatusList [counter1*12+6] = 1;

                                                                activeCellStatusList [counter1*12+9] = 0;

                                                            }

                                                        }

**else**{

                                                            activeCellStatusList [siblingCellNo1*12+4] = siblinFusionDoub1+10;

                                                            activeCellStatusList [counter1*12+4] = siblinFusionDoub1; //=====Switch assigned time length=====

                                                            activeCellStatusList [counter1*12+5] = 6;

                                                            activeCellStatusList [counter1*12+6] = 1;

                                                            activeCellStatusList [counter1*12+9] = 0;

                                                        }

                                                    }

                                                }

                                            }

                                        }

**else** **if** (activeCellStatusList [counter1*12+5] == 2){ //=====TD=====

                                            siblinFusionCheck1 = 0;

                                            siblinFusionCheck2 = 0;

                                            siblinFusionDoub1 = 0;

                                            siblinFusionDoub2 = 0;

                                            siblingCellNo1 = 0;

                                            siblingCellNo2 = 0;

                                            //====Check the status of siblings=====

**for** (**int** counter2 = 0; counter2 < activeCellStatusListCount/12; counter2++){

**if** (activeCellStatusList [counter2*12+6] == 1 && (activeCellStatusList [counter2*12+1] == activeCellStatusList [counter1*12] || activeCellStatusList [counter2*12+2] == activeCellStatusList [counter1*12]) && activeCellStatusList [counter2*12+3] == activeCellStatusList [counter1*12+3]){

**if** (siblinFusionCheck1 == 0 && activeCellStatusList [counter2*12+5] != 0){

                                                        siblinFusionCheck1 = activeCellStatusList [counter2*12+5];

                                                        siblinFusionDoub1 = activeCellStatusList [counter2*12+4];

                                                        siblingCellNo1 = counter2;

                                                    }

**else** **if** (siblinFusionCheck1 != 0 && siblinFusionCheck2 == 0 && activeCellStatusList [counter2*12+5] != 0){

                                                        siblinFusionCheck2 = activeCellStatusList [counter2*12+5];

                                                        siblinFusionDoub2 = activeCellStatusList [counter2*12+4];

                                                        siblingCellNo2 = counter2;

**break**;

                                                    }

                                                }

                                            }

                                            //======Event type set======

                                            eventType = 0;

**if** (siblinFusionCheck1 == 6 && siblinFusionCheck2 == 6){

**for** (**int** counter2 = 0; counter2 < 100; counter2++){

                                                    randInit = rand() % 100 + 0;

**if** (secondEventTDList [randInit] != 10){

                                                        eventType = secondEventTDList [randInit];

                                                        loopCheck = 1;

**break**;

                                                    }

                                                }

**if** (loopCheck == 0) eventType = 13;

                                            }

**else**{

                                                randInit = rand() % 100 + 0;

                                                eventType = secondEventTDList [randInit];

**if** (eventType == 0) eventType = 13;

                                            }

**if** ((activeCellStatusList [counter1*12+8] == 2 || activeCellStatusList [counter1*12+8] == 3) && activeCellStatusList [counter1*12+8] == activeCellStatusList [counter1*12+7] && eventType == 12){

                                                eventType = 13;

                                            }

**if** (siblinFusionDoub1 < siblinFusionDoub2) siblinFusionDoubLarge = siblinFusionDoub2;

**else** siblinFusionDoubLarge = siblinFusionDoub1;

**if** (siblinFusionCheck1 == 6 && siblinFusionCheck2 == 6 && eventType == 10){

                                                eventType = 13;

                                            }

**if** (eventType == 10){

                                                //====Use the "expandTDCF", set CF====

                                                loopCheck = 0;

**if** (siblinFusionCheck1 != 0 && siblinFusionCheck2 != 0){

**if** (siblinFusionCheck1 == 6 && siblinFusionCheck2 != 6){

**if** (expandTDCFCount == 0){

                                                            randInit = rand() % randCDRangeB + randCDRangeA; //=====Use CD data=====

**if** (siblinFusionDoub2 < randBDRangeA) siblinFusionDoub2 = randBDRangeA;

**if** (siblinFusionDoub1 > siblinFusionDoub2){

**if** (randInit > siblinFusionDoub1){

                                                                    activeCellStatusList [siblingCellNo2*12+4] = randInit+10;

                                                                    activeCellStatusList [counter1*12+4] = siblinFusionDoub2; //=====Switch assigned time length=====

                                                                    activeCellStatusList [counter1*12+5] = 6;

                                                                    activeCellStatusList [counter1*12+6] = 1;

                                                                }

**else**{

                                                                    activeCellStatusList [siblingCellNo2*12+4] = siblinFusionDoub1+10;

                                                                    activeCellStatusList [counter1*12+4] = siblinFusionDoub2; //=====Switch assigned time length=====

                                                                    activeCellStatusList [counter1*12+5] = 6;

                                                                    activeCellStatusList [counter1*12+6] = 1;

                                                                }

                                                            }

**else** **if** (siblinFusionDoub1 < siblinFusionDoub2){

**if** (randInit > siblinFusionDoub2){

                                                                    activeCellStatusList [siblingCellNo2*12+4] = randInit+10;

                                                                    activeCellStatusList [counter1*12+4] = siblinFusionDoub2; //=====Switch assigned time length=====

                                                                    activeCellStatusList [counter1*12+5] = 6;

                                                                    activeCellStatusList [counter1*12+6] = 1;

                                                                }

**else**{

                                                                    activeCellStatusList [siblingCellNo2*12+4] = siblinFusionDoub2+10;

                                                                    activeCellStatusList [counter1*12+4] = siblinFusionDoub2; //=====Switch assigned time length=====

                                                                    activeCellStatusList [counter1*12+5] = 6;

                                                                    activeCellStatusList [counter1*12+6] = 1;

                                                                }

                                                            }

                                                        }

**else**{

**if** (siblinFusionDoub1 > siblinFusionDoub2){

**if** (expandTDCFCount != 0){

                                                                    randInit = rand() % expandTDCFCount/2 + 0;

**if** (expandTDCF [randInit*2] > siblinFusionDoub1){

                                                                        activeCellStatusList [siblingCellNo2*12+4] = expandTDCF [randInit*2]+10;

                                                                        activeCellStatusList [counter1*12+4] = siblinFusionDoub2; //=====Switch assigned time length=====

                                                                        activeCellStatusList [counter1*12+5] = 6;

                                                                        activeCellStatusList [counter1*12+6] = 1;

                                                                    }

**else**{

                                                                        activeCellStatusList [siblingCellNo2*12+4] = siblinFusionDoub1+10;

                                                                        activeCellStatusList [counter1*12+4] = siblinFusionDoub2; //=====Switch assigned time length=====

                                                                        activeCellStatusList [counter1*12+5] = 6;

                                                                        activeCellStatusList [counter1*12+6] = 1;

                                                                    }

                                                                }

**else**{

                                                                    activeCellStatusList [siblingCellNo2*12+4] = siblinFusionDoub1+10;

                                                                    activeCellStatusList [counter1*12+4] = siblinFusionDoub2; //=====Switch assigned time length=====

                                                                    activeCellStatusList [counter1*12+5] = 6;

                                                                    activeCellStatusList [counter1*12+6] = 1;

                                                                }

                                                            }

**else** **if** (siblinFusionDoub1 < siblinFusionDoub2){

**if** (expandTDCFCount != 0){

                                                                    randInit = rand() % expandTDCFCount/2 + 0;

**if** (expandTDCF [randInit*2] > siblinFusionDoub2){

                                                                        activeCellStatusList [siblingCellNo2*12+4] = expandTDCF [randInit*2]+10;

                                                                        activeCellStatusList [counter1*12+4] = siblinFusionDoub2; //=====Switch assigned time length=====

                                                                        activeCellStatusList [counter1*12+5] = 6;

                                                                        activeCellStatusList [counter1*12+6] = 1;

                                                                    }

**else**{

                                                                        activeCellStatusList [siblingCellNo2*12+4] = siblinFusionDoub2+10;

                                                                        activeCellStatusList [counter1*12+4] = siblinFusionDoub2; //=====Switch assigned time length=====

                                                                        activeCellStatusList [counter1*12+5] = 6;

                                                                        activeCellStatusList [counter1*12+6] = 1;

                                                                    }

                                                                }

**else**{

                                                                    activeCellStatusList [siblingCellNo2*12+4] = siblinFusionDoub2+10;

                                                                    activeCellStatusList [counter1*12+4] = siblinFusionDoub2; //=====Switch assigned time length=====

                                                                    activeCellStatusList [counter1*12+5] = 6;

                                                                    activeCellStatusList [counter1*12+6] = 1;

                                                                }

                                                            }

                                                        }

                                                    }

**else** **if** (siblinFusionCheck1 != 6 && siblinFusionCheck2 == 6){

**if** (expandTDCFCount == 0){

                                                            randInit = rand() % randCDRangeB + randCDRangeA; //=====Use CD data======

**if** (siblinFusionDoub1 > siblinFusionDoub2){

**if** (randInit > siblinFusionDoub1){

                                                                    activeCellStatusList [siblingCellNo1*12+4] = randInit+10;

                                                                    activeCellStatusList [counter1*12+4] = siblinFusionDoub1; //=====Switch assigned time length=====

                                                                    activeCellStatusList [counter1*12+5] = 6;

                                                                    activeCellStatusList [counter1*12+6] = 1;

                                                                }

**else**{

                                                                    activeCellStatusList [siblingCellNo1*12+4] = siblinFusionDoub1+10;

                                                                    activeCellStatusList [counter1*12+4] = siblinFusionDoub1; //=====Switch assigned time length=====

                                                                    activeCellStatusList [counter1*12+5] = 6;

                                                                    activeCellStatusList [counter1*12+6] = 1;

                                                                }

                                                            }

**else** **if** (siblinFusionDoub1 < siblinFusionDoub2){

**if** (randInit > siblinFusionDoub2){

                                                                    activeCellStatusList [siblingCellNo1*12+4] = randInit+10;

                                                                    activeCellStatusList [counter1*12+4] = siblinFusionDoub1; //=====Switch assigned time length=====

                                                                    activeCellStatusList [counter1*12+5] = 6;

                                                                    activeCellStatusList [counter1*12+6] = 1;

                                                                }

**else**{

                                                                    activeCellStatusList [siblingCellNo1*12+4] = siblinFusionDoub2+10;

                                                                    activeCellStatusList [counter1*12+4] = siblinFusionDoub1; //=====Switch assigned time length=====

                                                                    activeCellStatusList [counter1*12+5] = 6;

                                                                    activeCellStatusList [counter1*12+6] = 1;

                                                                }

                                                            }

                                                        }

**else**{

**if** (siblinFusionDoub1 > siblinFusionDoub2){

**if** (expandTDCFCount != 0){

                                                                    randInit = rand() % expandTDCFCount/2 + 0;

**if** (expandTDCF [randInit*2] > siblinFusionDoub1){

                                                                        activeCellStatusList [siblingCellNo1*12+4] = expandTDCF [randInit*2]+10;

                                                                        activeCellStatusList [counter1*12+4] = siblinFusionDoub1; //=====Switch assigned time length=====

                                                                        activeCellStatusList [counter1*12+5] = 6;

                                                                        activeCellStatusList [counter1*12+6] = 1;

                                                                    }

**else**{

                                                                        activeCellStatusList [siblingCellNo1*12+4] = siblinFusionDoub1+10;

                                                                        activeCellStatusList [counter1*12+4] = siblinFusionDoub1; //=====Switch assigned time length=====

                                                                        activeCellStatusList [counter1*12+5] = 6;

                                                                        activeCellStatusList [counter1*12+6] = 1;

                                                                    }

                                                                }

**else**{

                                                                    activeCellStatusList [siblingCellNo1*12+4] = siblinFusionDoub1+10;

                                                                    activeCellStatusList [counter1*12+4] = siblinFusionDoub1; //=====Switch assigned time length=====

                                                                    activeCellStatusList [counter1*12+5] = 6;

                                                                    activeCellStatusList [counter1*12+6] = 1;

                                                                }

                                                            }

**else** **if** (siblinFusionDoub1 < siblinFusionDoub2){

**if** (expandTDCFCount != 0){

                                                                    randInit = rand() % expandTDCFCount/2 + 0;

**if** (expandTDCF [randInit*2] >= siblinFusionDoub2){

                                                                        activeCellStatusList [siblingCellNo1*12+4] = expandTDCF [randInit*2]+10;

                                                                        activeCellStatusList [counter1*12+4] = siblinFusionDoub1; //=====Switch assigned time length=====

                                                                        activeCellStatusList [counter1*12+5] = 6;

                                                                        activeCellStatusList [counter1*12+6] = 1;

                                                                    }

**else**{

                                                                        activeCellStatusList [siblingCellNo1*12+4] = siblinFusionDoub2+10;

                                                                        activeCellStatusList [counter1*12+4] = siblinFusionDoub1; //=====Switch assigned time length=====

                                                                        activeCellStatusList [counter1*12+5] = 6;

                                                                        activeCellStatusList [counter1*12+6] = 1;

                                                                    }

                                                                }

**else**{

                                                                    activeCellStatusList [siblingCellNo1*12+4] = siblinFusionDoub2+10;

                                                                    activeCellStatusList [counter1*12+4] = siblinFusionDoub1; //=====Switch assigned time length=====

                                                                    activeCellStatusList [counter1*12+5] = 6;

                                                                    activeCellStatusList [counter1*12+6] = 1;

                                                                }

                                                            }

                                                        }

                                                    }

**else** **if** (siblinFusionCheck1 != 6 && siblinFusionCheck2 != 6){

                                                        activeCellStatusList [siblingCellNo1*12+4] = siblinFusionDoub1+10;

                                                        activeCellStatusList [siblingCellNo2*12+4] = siblinFusionDoub2+10;

                                                        activeCellStatusList [counter1*12+4] = siblinFusionDoub2; //=====Switch assigned time length=====

                                                        activeCellStatusList [counter1*12+5] = 6;

                                                        activeCellStatusList [counter1*12+6] = 1;

                                                    }

                                                }

**else** **if** (siblinFusionCheck1 != 0 && siblinFusionCheck2 == 0){

**if** (expandTDCFCount == 0){

                                                        randInit = rand() % randCDRangeB + randCDRangeA; //=====Use CD data=====

**if** (randInit > siblinFusionDoub1+10){

                                                            activeCellStatusList [counter1*12+4] = randInit;

                                                            activeCellStatusList [counter1*12+5] = 6;

                                                            activeCellStatusList [counter1*12+6] = 1;

                                                        }

**else**{

                                                            activeCellStatusList [counter1*12+4] = siblinFusionDoub1+5;

                                                            activeCellStatusList [counter1*12+5] = 6;

                                                            activeCellStatusList [counter1*12+6] = 1;

                                                        }

                                                    }

**else**{

**if** (expandTDCFCount != 0){

                                                            randInit = rand() % expandTDCFCount/2 + 0;

**if** (expandTDCF [randInit*2] > siblinFusionDoub1+10){

                                                                activeCellStatusList [counter1*12+4] = expandTDCF [randInit*2];

                                                                activeCellStatusList [counter1*12+5] = 6;

                                                                activeCellStatusList [counter1*12+6] = 1;

                                                            }

**else**{

                                                                activeCellStatusList [counter1*12+4] = siblinFusionDoub1+5;

                                                                activeCellStatusList [counter1*12+5] = 6;

                                                                activeCellStatusList [counter1*12+6] = 1;

                                                            }

                                                        }

**else**{

                                                            activeCellStatusList [counter1*12+4] = siblinFusionDoub1+5;

                                                            activeCellStatusList [counter1*12+5] = 6;

                                                            activeCellStatusList [counter1*12+6] = 1;

                                                        }

                                                    }

                                                }

**else** **if** (siblinFusionCheck1 == 0 && siblinFusionCheck2 == 0){

**if** (expandTDCFCount == 0){

                                                        randInit = rand() % randCDRangeB + randCDRangeA; //=====Use CD data=====

                                                        activeCellStatusList [counter1*12+4] = randInit;

                                                        activeCellStatusList [counter1*12+5] = 6;

                                                        activeCellStatusList [counter1*12+6] = 1;

                                                    }

**else**{

**if** (activeCellStatusList [counter1*12+11] == 0){

                                                            randInit = rand() % expandTDCFCount/2 + 0;

                                                            activeCellStatusList [counter1*12+4] = expandTDCF [randInit*2];

                                                            activeCellStatusList [counter1*12+5] = 6;

                                                            activeCellStatusList [counter1*12+6] = 1;

                                                        }

**else**{

                                                            randInit = rand() % expandTDCFCount/2 + 0;

                                                            activeCellStatusList [counter1*12+4] = expandTDCF [randInit*2];

                                                            activeCellStatusList [counter1*12+5] = 6;

                                                            activeCellStatusList [counter1*12+6] = 1;

                                                        }

                                                    }

                                                }

                                            }

**else** **if** (eventType == 11){

                                                //====Use the "expandDoublingDoubTD", set BD====

**if** (expandDoublingDoubTDCount == 0){

                                                    randInit = rand() % randBDRangeB + randBDRangeA; //=====Use CD data=====

**if** (randInit > siblinFusionDoubLarge+10){

                                                        activeCellStatusList [counter1*12+4] = randInit;

                                                        activeCellStatusList [counter1*12+5] = 1;

                                                        activeCellStatusList [counter1*12+6] = 1;

                                                        activeCellStatusList [counter1*12+9] = (**int**)simProcessDataBaseHold [19];

                                                        activeCellStatusList [counter1*12+7] = 0;

                                                    }

**else**{

**if** (siblinFusionDoubLarge < randBDRangeA) siblinFusionDoubLarge = randBDRangeA;

                                                        activeCellStatusList [counter1*12+4] = siblinFusionDoubLarge+10;

                                                        activeCellStatusList [counter1*12+5] = 1;

                                                        activeCellStatusList [counter1*12+6] = 1;

                                                        activeCellStatusList [counter1*12+9] = (**int**)simProcessDataBaseHold [19];

                                                        activeCellStatusList [counter1*12+7] = 0;

                                                    }

                                                }

**else**{

**if** (activeCellStatusList [counter1*12+11] == 0){

**if** (expandDoublingDoubTDCount != 0){

                                                            randInit = rand() % expandDoublingDoubTDCount/2 + 0;

**if** (randInit > siblinFusionDoubLarge+10){

                                                                activeCellStatusList [counter1*12+4] = expandDoublingDoubTD [randInit*2];

                                                                activeCellStatusList [counter1*12+5] = 1;

                                                                activeCellStatusList [counter1*12+6] = 1;

                                                                activeCellStatusList [counter1*12+9] = (**int**)simProcessDataBaseHold [19];

                                                                activeCellStatusList [counter1*12+7] = 0;

                                                            }

**else**{

**if** (siblinFusionDoubLarge < randBDRangeA) siblinFusionDoubLarge = randBDRangeA;

                                                                activeCellStatusList [counter1*12+4] = siblinFusionDoubLarge+10;

                                                                activeCellStatusList [counter1*12+5] = 1;

                                                                activeCellStatusList [counter1*12+6] = 1;

                                                                activeCellStatusList [counter1*12+9] = (**int**)simProcessDataBaseHold [19];

                                                                activeCellStatusList [counter1*12+7] = 0;

                                                            }

                                                        }

**else**{

**if** (siblinFusionDoubLarge < randBDRangeA) siblinFusionDoubLarge = randBDRangeA;

                                                            activeCellStatusList [counter1*12+4] = siblinFusionDoubLarge+10;

                                                            activeCellStatusList [counter1*12+5] = 1;

                                                            activeCellStatusList [counter1*12+6] = 1;

                                                            activeCellStatusList [counter1*12+9] = (**int**)simProcessDataBaseHold [19];

                                                            activeCellStatusList [counter1*12+7] = 0;

                                                        }

                                                    }

**else**{

**if** (expandDoublingDoubTDCount != 0){

                                                            randInit = rand() % expandDoublingDoubTDCount/2 + 0;

**if** (expandDoublingDoubTD [randInit*2] > siblinFusionDoubLarge+10){

                                                                activeCellStatusList [counter1*12+4] = expandDoublingDoubTD [randInit*2];

                                                                activeCellStatusList [counter1*12+5] = 1;

                                                                activeCellStatusList [counter1*12+6] = 1;

                                                                activeCellStatusList [counter1*12+9] = (**int**)simProcessDataBaseHold [19];

                                                                activeCellStatusList [counter1*12+7] = 0;

                                                            }

**else**{

**if** (siblinFusionDoubLarge < randBDRangeA) siblinFusionDoubLarge = randBDRangeA;

                                                                activeCellStatusList [counter1*12+4] = siblinFusionDoubLarge+10;

                                                                activeCellStatusList [counter1*12+5] = 1;

                                                                activeCellStatusList [counter1*12+6] = 1;

                                                                activeCellStatusList [counter1*12+9] = (**int**)simProcessDataBaseHold [19];

                                                                activeCellStatusList [counter1*12+7] = 0;

                                                            }

                                                        }

**else**{

**if** (siblinFusionDoubLarge < randBDRangeA) siblinFusionDoubLarge = randBDRangeA;

                                                            activeCellStatusList [counter1*12+4] = siblinFusionDoubLarge+10;

                                                            activeCellStatusList [counter1*12+5] = 1;

                                                            activeCellStatusList [counter1*12+6] = 1;

                                                            activeCellStatusList [counter1*12+9] = (**int**)simProcessDataBaseHold [19];

                                                            activeCellStatusList [counter1*12+7] = 0;

                                                        }

                                                    }

                                                }

                                            }

**else** **if** (eventType == 12){

                                                //====Use the "expandDoublingDoubTD", set TD====

**if** (expandDoublingDoubTDCount == 0){

                                                    randInit = rand() % randBDRangeB + randBDRangeA; //=====Use CD data=====

**if** (randInit > siblinFusionDoubLarge+10){

                                                        activeCellStatusList [counter1*12+4] = randInit;

                                                        activeCellStatusList [counter1*12+5] = 2;

                                                        activeCellStatusList [counter1*12+6] = 1;

                                                        activeCellStatusList [counter1*12+7]++;

                                                    }

**else**{

**if** (siblinFusionDoubLarge < randBDRangeA) siblinFusionDoubLarge = randBDRangeA;

                                                        activeCellStatusList [counter1*12+4] = siblinFusionDoubLarge+10;

                                                        activeCellStatusList [counter1*12+5] = 2;

                                                        activeCellStatusList [counter1*12+6] = 1;

                                                        activeCellStatusList [counter1*12+7]++;

                                                    }

                                                }

**else**{

**if** (activeCellStatusList [counter1*12+11] == 0){

**if** (expandDoublingDoubTDCount != 0){

                                                            randInit = rand() % expandDoublingDoubTDCount/2 + 0;

**if** (randInit >= siblinFusionDoubLarge+10){

                                                                activeCellStatusList [counter1*12+4] = expandDoublingDoubTD [randInit*2];

                                                                activeCellStatusList [counter1*12+5] = 2;

                                                                activeCellStatusList [counter1*12+6] = 1;

                                                                activeCellStatusList [counter1*12+7]++;

                                                            }

**else**{

**if** (siblinFusionDoubLarge < randBDRangeA) siblinFusionDoubLarge = randBDRangeA;

                                                                activeCellStatusList [counter1*12+4] = siblinFusionDoubLarge+10;

                                                                activeCellStatusList [counter1*12+5] = 2;

                                                                activeCellStatusList [counter1*12+6] = 1;

                                                                activeCellStatusList [counter1*12+7]++;

                                                            }

                                                        }

**else**{

**if** (siblinFusionDoubLarge < randBDRangeA) siblinFusionDoubLarge = randBDRangeA;

                                                            activeCellStatusList [counter1*12+4] = siblinFusionDoubLarge+10;

                                                            activeCellStatusList [counter1*12+5] = 2;

                                                            activeCellStatusList [counter1*12+6] = 1;

                                                            activeCellStatusList [counter1*12+7]++;

                                                        }

                                                    }

**else**{

**if** (expandDoublingDoubTDCount != 0){

                                                            randInit = rand() % expandDoublingDoubTDCount/2 + 0;

**if** (expandDoublingDoubTD [randInit*2] > siblinFusionDoubLarge+10){

                                                                activeCellStatusList [counter1*12+4] = expandDoublingDoubTD [randInit*2];

                                                                activeCellStatusList [counter1*12+5] = 2;

                                                                activeCellStatusList [counter1*12+7]++;

                                                            }

**else**{

**if** (siblinFusionDoubLarge < randBDRangeA) siblinFusionDoubLarge = randBDRangeA;

                                                                activeCellStatusList [counter1*12+4] = siblinFusionDoubLarge+10;

                                                                activeCellStatusList [counter1*12+5] = 2;

                                                                activeCellStatusList [counter1*12+7]++;

                                                            }

                                                        }

**else**{

**if** (siblinFusionDoubLarge < randBDRangeA) siblinFusionDoubLarge = randBDRangeA;

                                                            activeCellStatusList [counter1*12+4] = siblinFusionDoubLarge+10;

                                                            activeCellStatusList [counter1*12+5] = 2;

                                                            activeCellStatusList [counter1*12+7]++;

                                                        }

                                                    }

                                                }

                                            }

**else** **if** (eventType == 13){

                                                //====Use the "expandTDCD", set CD====

**if** (expandTDCDCount == 0){

                                                    randInit = rand() % randCDRangeB + randCDRangeA; //=====Use CD data=====

**if** (randInit > siblinFusionDoubLarge+10){

                                                        activeCellStatusList [counter1*12+4] = randInit;

                                                        activeCellStatusList [counter1*12+5] = 3;

                                                        activeCellStatusList [counter1*12+6] = 1;

                                                    }

**else**{

                                                        activeCellStatusList [counter1*12+4] = siblinFusionDoubLarge+10;

                                                        activeCellStatusList [counter1*12+5] = 3;

                                                        activeCellStatusList [counter1*12+6] = 1;

                                                    }

                                                }

**else**{

**if** (expandTDCDCount != 0){

                                                        randInit = rand() % expandTDCDCount/2 + 0;

**if** (expandTDCD [randInit*2] > siblinFusionDoubLarge+10){

                                                            activeCellStatusList [counter1*12+4] = expandTDCD [randInit*2];

                                                            activeCellStatusList [counter1*12+5] = 3;

                                                            activeCellStatusList [counter1*12+6] = 1;

                                                        }

**else**{

                                                            activeCellStatusList [counter1*12+4] = siblinFusionDoubLarge+10;

                                                            activeCellStatusList [counter1*12+5] = 3;

                                                            activeCellStatusList [counter1*12+6] = 1;

                                                        }

                                                    }

**else**{

                                                        activeCellStatusList [counter1*12+4] = siblinFusionDoubLarge+10;

                                                        activeCellStatusList [counter1*12+5] = 3;

                                                        activeCellStatusList [counter1*12+6] = 1;

                                                    }

                                                }

                                            }

                                        }

                                    }

**else**{

                                        //====The following process is the same as above, but the arrays used here are for mixed cells=====

**if** (activeCellStatusList [counter1*12+5] == 4){

**if** (simProcessDataProgHold [21]/(**double**)simProcessDataProgHold [24] > 1.0) dataTempDouble = 1;

**else** dataTempDouble = simProcessDataProgHold [21]/(**double**)simProcessDataProgHold [24];

                                            randInit = rand() % 100 + 0;

**if** (randInit < dataTempDouble*100){

                                                lowextValue = 100000;

                                                highestValue = 0;

**for** (**int** counter2 = 0; counter2 < expandDoublingDoubBDSelCount/2; counter2++){

**if** (lowextValue > expandDoublingDoubBDSel [counter2*2]) lowextValue = expandDoublingDoubBDSel [counter2*2];

**if** (highestValue < expandDoublingDoubBDSel [counter2*2]) highestValue = expandDoublingDoubBDSel [counter2*2];

                                                }

**if** (simProcessDataProgHold [25] < lowextValue){

                                                    activeCellStatusList [counter1*12+4] = (**int**)(round((highestValue+lowextValue)/(**double**)2));

                                                    activeCellStatusList [counter1*12+5] = 70;

                                                    activeCellStatusList [counter1*12+6] = 1;

                                                }

**else**{

**if** (highestValue != 0){

**if** (highestValue-10 > lowextValue) dataTempInt = highestValue-10;

**else** **if** (highestValue-10 <= lowextValue) dataTempInt = highestValue;

                                                    }

**else**{

**if** (simProcessDataProgHold [25]-10 > (**int**)(round(simProcessDataProgHold [25]*(**double**)0.8))) dataTempInt = (**int**)simProcessDataProgHold [25]-10;

**else** dataTempInt = (**int**)(round(simProcessDataProgHold [25]*(**double**)0.8));

                                                    }

                                                    activeCellStatusList [counter1*12+4] = dataTempInt;

                                                    activeCellStatusList [counter1*12+5] = 70;

                                                    activeCellStatusList [counter1*12+6] = 1;

                                                }

                                            }

**else**{

                                                dataTempDouble = ((expandNonCDSelCount/(**double**)2)/(**double**)simProcessDataProgHold [24])*100;

                                                randInit = rand() % 100 + 0;

**if** (randInit < dataTempDouble && expandNonCDSelCount != 0){

                                                    randInit = rand() % expandNonCDSelCount/2 + 0;

                                                    activeCellStatusList [counter1*12+4] = expandNonCDSel [randInit*2];

                                                    activeCellStatusList [counter1*12+5] = 30;

                                                    activeCellStatusList [counter1*12+6] = 1;

                                                }

**else**{

                                                    randInit2 = rand() % (**int**)(round(simProcessDataProgHold [25]*(endVariationtHold/(**double**)100))) + 0;

**if** (randInit2%2 == 0) activeCellStatusList [counter1*12+4] = (**int**)simProcessDataProgHold [25]+randInit2;

**else** activeCellStatusList [counter1*12+4] = (**int**)simProcessDataProgHold [25]-randInit2;

                                                    activeCellStatusList [counter1*12+5] = 5;

                                                    activeCellStatusList [counter1*12+6] = 1;

                                                }

                                            }

                                        }

**else** **if** (activeCellStatusList [counter1*12+5] == 5){

                                            dataTempDouble = ((expandNonCDSelCount/(**double**)2)/(**double**)simProcessDataProgHold [24])*100;

                                            randInit = rand() % 100 + 0;

**if** (randInit < dataTempDouble && expandNonCDSelCount != 0){

                                                randInit = rand() % expandNonCDSelCount/2 + 0;

                                                activeCellStatusList [counter1*12+4] = expandNonCDSel [randInit*2];

                                                activeCellStatusList [counter1*12+5] = 30;

                                                activeCellStatusList [counter1*12+6] = 1;

                                            }

**else**{

                                                randInit2 = rand() % (**int**)(round(simProcessDataProgHold [25]*(endVariationtHold/(**double**)100))) + 0;

**if** (randInit2%2 == 0) activeCellStatusList [counter1*12+4] = (**int**)simProcessDataProgHold [25]+randInit2;

**else** activeCellStatusList [counter1*12+4] = (**int**)simProcessDataProgHold [25]-randInit2;

                                                activeCellStatusList [counter1*12+5] = 5;

                                                activeCellStatusList [counter1*12+6] = 1;

                                            }

                                        }

**else** **if** (activeCellStatusList [counter1*12+5] == 7){

                                            eventType = 1;

**if** (eventType == 1){

**if** (activeCellStatusList [counter1*12+11] == 0){

**if** (expandDoublingDoubBDSelCount != 0){

                                                        randInit = rand() % expandDoublingDoubBDSelCount/2 + 0;

                                                        activeCellStatusList [counter1*12+4] = expandDoublingDoubBDSel [randInit*2];

                                                        activeCellStatusList [counter1*12+5] = 1;

                                                        activeCellStatusList [counter1*12+6] = 1;

                                                    }

**else**{

                                                        randInit = rand() % randBDRangeSelB + randBDRangeSelA;

                                                        activeCellStatusList [counter1*12+4] = randInit;

                                                        activeCellStatusList [counter1*12+5] = 7;

                                                        activeCellStatusList [counter1*12+6] = 1;

                                                    }

                                                }

**else**{

                                                    loopCheck = 0;

**for** (**int** counter2 = 0; counter2 < 100; counter2++){

**if** (expandDoublingDoubBDSelCount != 0){

                                                            randInit = rand() % expandDoublingDoubBDSelCount/2 + 0;

**if** (expandDoublingDoubBDSel [randInit*2] > activeCellStatusList [counter1*12+11]-50 && expandDoublingDoubBDSel [randInit*2] < activeCellStatusList [counter1*12+11]+50){

                                                                activeCellStatusList [counter1*12+4] = expandDoublingDoubBDSel [randInit*2];

                                                                activeCellStatusList [counter1*12+5] = 1;

                                                                activeCellStatusList [counter1*12+6] = 1;

                                                                loopCheck = 1;

**break**;

                                                            }

                                                        }

                                                    }

**if** (loopCheck == 0){

                                                        activeCellStatusList [counter1*12+4] = activeCellStatusList [counter1*12+11];

                                                        activeCellStatusList [counter1*12+5] = 7;

                                                        activeCellStatusList [counter1*12+6] = 1;

                                                    }

                                                }

                                            }

                                        }

**else** **if** (activeCellStatusList [counter1*12+5] == 1){

                                            siblinFusionCheck1 = 0;

                                            siblinFusionDoub1 = 0;

                                            siblingCellNo1 = 0;

**for** (**int** counter2 = 0; counter2 < activeCellStatusListCount/12; counter2++){

**if** (activeCellStatusList [counter2*12+6] == 1 && activeCellStatusList [counter2*12+1] == activeCellStatusList [counter1*12] && activeCellStatusList [counter2*12+3] == activeCellStatusList [counter1*12+3]){

                                                    siblingCellNo1 = counter2;

**if** (activeCellStatusList [counter2*12+5] != 0){

                                                        siblinFusionCheck1 = activeCellStatusList [counter2*12+5];

                                                        siblinFusionDoub1 = activeCellStatusList [counter2*12+4];

                                                    }

**break**;

                                                }

                                            }

                                            eventType = 0;

**if** (activeCellStatusList [counter1*12+9] == 0){

**if** (siblinFusionCheck1 == 6){

                                                    loopCheck = 0;

**for** (**int** counter2 = 0; counter2 < 100; counter2++){

                                                        randInit = rand() % 100 + 0;

**if** (secondEventBDListSel [randInit] != 6){

                                                            eventType = secondEventBDListSel [randInit];

                                                            loopCheck = 1;

**break**;

                                                        }

                                                    }

**if** (loopCheck == 0) eventType = 1;

                                                }

**else**{

                                                    randInit = rand() % 100 + 0;

**if** (secondEventBDListSel [randInit] != 0) eventType = secondEventBDListSel [randInit];

**else** eventType = 1;

                                                }

                                            }

**else** **if** (activeCellStatusList [counter1*12+9] != 0){

                                                eventCount = 0;

                                                eventType = 0;

**if** (siblinFusionCheck1 == 6) eventType = 5;

**else**{

**for** (**int** counter2 = 0; counter2 < 5; counter2++){

                                                        randInit = rand() % 100 + 0;

**if** (secondEventBDListSel [randInit] == 1) eventCount++;

**else**{

                                                            eventType = secondEventBDListSel [randInit];

**break**;

                                                        }

                                                    }

**if** (eventCount == 5 || eventType == 0) eventType = 1;

                                                }

                                            }

**if** (eventType == 1){

**if** (activeCellStatusList [counter1*12+11] == 0){

**if** (siblinFusionCheck1 != 6){

**if** (expandDoublingDoubBDSelCount != 0){

                                                            randInit = rand() % expandDoublingDoubBDSelCount/2 + 0;

                                                            activeCellStatusList [counter1*12+4] = expandDoublingDoubBDSel [randInit*2];

                                                            activeCellStatusList [counter1*12+5] = 1;

                                                            activeCellStatusList [counter1*12+6] = 1;

**if** (activeCellStatusList [counter1*12+9] != 0) activeCellStatusList [counter1*12+9]--;

                                                        }

**else**{

                                                            randInit = rand() % randBDRangeSelB + randBDRangeSelA;

                                                            activeCellStatusList [counter1*12+4] = randInit;

                                                            activeCellStatusList [counter1*12+5] = 1;

                                                            activeCellStatusList [counter1*12+6] = 1;

**if** (activeCellStatusList [counter1*12+9] != 0) activeCellStatusList [counter1*12+9]--;

                                                        }

                                                    }

**else**{

**if** (siblinFusionDoub1 < randBDRangeSelA) siblinFusionDoub1 = randBDRangeSelA;

                                                        activeCellStatusList [counter1*12+4] = siblinFusionDoub1+10;

                                                        activeCellStatusList [counter1*12+5] = 1;

                                                        activeCellStatusList [counter1*12+6] = 1;

**if** (activeCellStatusList [counter1*12+9] != 0) activeCellStatusList [counter1*12+9]--;

                                                    }

                                                }

**else**{

                                                    loopCheck = 0;

**for** (**int** counter2 = 0; counter2 < 100; counter2++){

**if** (siblinFusionCheck1 != 6){

**if** (expandDoublingDoubBDSelCount != 0){

                                                                randInit = rand() % expandDoublingDoubBDSelCount/2 + 0;

**if** (expandDoublingDoubBDSel [randInit*2] > activeCellStatusList [counter1*12+11]-50 && expandDoublingDoubBDSel [randInit*2] < activeCellStatusList [counter1*12+11]+50){

                                                                    activeCellStatusList [counter1*12+4] = expandDoublingDoubBDSel [randInit*2];

                                                                    activeCellStatusList [counter1*12+5] = 1;

                                                                    activeCellStatusList [counter1*12+6] = 1;

**if** (activeCellStatusList [counter1*12+9] != 0) activeCellStatusList [counter1*12+9]--;

                                                                    loopCheck = 1;

**break**;

                                                                }

                                                            }

                                                        }

**else** **if** (siblinFusionCheck1 == 6){

**if** (expandDoublingDoubBDSelCount != 0){

                                                                randInit = rand() % expandDoublingDoubBDSelCount/2 + 0;

**if** (expandDoublingDoubBDSel [randInit*2] > activeCellStatusList [counter1*12+11]-50 && expandDoublingDoubBDSel [randInit*2] < activeCellStatusList [counter1*12+11]+50 && siblinFusionDoub1+10 < expandDoublingDoubBDSel [randInit*2]){

                                                                    activeCellStatusList [counter1*12+4] = expandDoublingDoubBDSel [randInit*2];

                                                                    activeCellStatusList [counter1*12+5] = 1;

                                                                    activeCellStatusList [counter1*12+6] = 1;

**if** (activeCellStatusList [counter1*12+9] != 0) activeCellStatusList [counter1*12+9]--;

                                                                    loopCheck = 1;

**break**;

                                                                }

                                                            }

                                                        }

                                                    }

**if** (loopCheck == 0){

                                                        lowextValue = 100000;

                                                        highestValue = 0;

**for** (**int** counter2 = 0; counter2 < expandDoublingDoubBDSelCount/2; counter2++){

**if** (lowextValue > expandDoublingDoubBDSel [counter2*2]) lowextValue = expandDoublingDoubBDSel [counter2*2];

**if** (highestValue < expandDoublingDoubBDSel [counter2*2]) highestValue = expandDoublingDoubBDSel [counter2*2];

                                                        }

**if** (siblinFusionDoub1 < randBDRangeSelA) siblinFusionDoub1 = randBDRangeSelA;

**if** (lowextValue != 100000 && highestValue != 0){

**if** ((highestValue+lowextValue)/(**double**)2 > siblinFusionDoub1+10){

                                                                activeCellStatusList [counter1*12+4] = (**int**)(round((highestValue+lowextValue)/(**double**)2));

                                                                activeCellStatusList [counter1*12+5] = 1;

                                                                activeCellStatusList [counter1*12+6] = 1;

**if** (activeCellStatusList [counter1*12+9] != 0) activeCellStatusList [counter1*12+9]--;

                                                            }

**else**{

                                                                activeCellStatusList [counter1*12+4] = siblinFusionDoub1+10;

                                                                activeCellStatusList [counter1*12+5] = 1;

                                                                activeCellStatusList [counter1*12+6] = 1;

**if** (activeCellStatusList [counter1*12+9] != 0) activeCellStatusList [counter1*12+9]--;

                                                            }

                                                        }

**else**{

                                                            activeCellStatusList [counter1*12+4] = siblinFusionDoub1+10;

                                                            activeCellStatusList [counter1*12+5] = 1;

                                                            activeCellStatusList [counter1*12+6] = 1;

**if** (activeCellStatusList [counter1*12+9] != 0) activeCellStatusList [counter1*12+9]--;

                                                        }

                                                    }

                                                }

                                            }

**else** **if** (eventType == 2){

**if** (activeCellStatusList [counter1*12+11] == 0){

**if** (siblinFusionCheck1 != 6){

**if** (expandDoublingDoubBDSelCount != 0){

                                                            randInit = rand() % expandDoublingDoubBDSelCount/2 + 0;

                                                            activeCellStatusList [counter1*12+4] = expandDoublingDoubBDSel [randInit*2];

                                                            activeCellStatusList [counter1*12+5] = 2;

                                                            activeCellStatusList [counter1*12+6] = 1;

                                                            activeCellStatusList [counter1*12+7] = 1;

                                                            activeCellStatusList [counter1*12+9] = 0;

                                                            activeCellStatusList [counter1*12+8] = (**int**)simProcessDataProgHold [18];

                                                        }

**else**{

                                                            randInit = rand() % randBDRangeSelB + randBDRangeSelA;

                                                            activeCellStatusList [counter1*12+4] = randInit;

                                                            activeCellStatusList [counter1*12+5] = 2;

                                                            activeCellStatusList [counter1*12+6] = 1;

                                                            activeCellStatusList [counter1*12+7] = 1;

                                                            activeCellStatusList [counter1*12+9] = 0;

                                                            activeCellStatusList [counter1*12+8] = (**int**)simProcessDataProgHold [18];

                                                        }

                                                    }

**else**{

**if** (siblinFusionDoub1 < randBDRangeSelA) siblinFusionDoub1 = randBDRangeSelA;

                                                        activeCellStatusList [counter1*12+4] = siblinFusionDoub1+10;

                                                        activeCellStatusList [counter1*12+5] = 2;

                                                        activeCellStatusList [counter1*12+6] = 1;

                                                        activeCellStatusList [counter1*12+7] = 1;

                                                        activeCellStatusList [counter1*12+9] = 0;

                                                        activeCellStatusList [counter1*12+8] = (**int**)simProcessDataProgHold [18];

                                                    }

                                                }

**else**{

                                                    loopCheck = 0;

**for** (**int** counter2 = 0; counter2 < 100; counter2++){

**if** (siblinFusionCheck1 != 6){

**if** (expandDoublingDoubBDSelCount != 0){

                                                                randInit = rand() % expandDoublingDoubBDSelCount/2 + 0;

**if** (expandDoublingDoubBDSel [randInit*2] > activeCellStatusList [counter1*12+11]-50 && expandDoublingDoubBDSel [randInit*2] < activeCellStatusList [counter1*12+11]+50){

                                                                    activeCellStatusList [counter1*12+4] = expandDoublingDoubBDSel [randInit*2];

                                                                    activeCellStatusList [counter1*12+5] = 2;

                                                                    activeCellStatusList [counter1*12+6] = 1;

                                                                    activeCellStatusList [counter1*12+7] = 1;

                                                                    activeCellStatusList [counter1*12+9] = 0;

                                                                    activeCellStatusList [counter1*12+8] = (**int**)simProcessDataProgHold [18];

                                                                    loopCheck = 1;

**break**;

                                                                }

                                                            }

                                                        }

**else** **if** (siblinFusionCheck1 == 6){

**if** (expandDoublingDoubBDSelCount != 0){

                                                                randInit = rand() % expandDoublingDoubBDSelCount/2 + 0;

**if** (expandDoublingDoubBDSel [randInit*2] > activeCellStatusList [counter1*12+11]-50 && expandDoublingDoubBDSel [randInit*2] < activeCellStatusList [counter1*12+11]+50 && siblinFusionDoub1+10 < expandDoublingDoubBDSel [randInit*2]){

                                                                    activeCellStatusList [counter1*12+4] = expandDoublingDoubBDSel [randInit*2];

                                                                    activeCellStatusList [counter1*12+5] = 2;

                                                                    activeCellStatusList [counter1*12+6] = 1;

                                                                    activeCellStatusList [counter1*12+7] = 1;

                                                                    activeCellStatusList [counter1*12+9] = 0;

                                                                    activeCellStatusList [counter1*12+8] = (**int**)simProcessDataProgHold [18];

                                                                    loopCheck = 1;

**break**;

                                                                }

                                                            }

                                                        }

                                                    }

**if** (loopCheck == 0){

                                                        lowextValue = 100000;

                                                        highestValue = 0;

**for** (**int** counter2 = 0; counter2 < expandDoublingDoubBDSelCount/2; counter2++){

**if** (lowextValue > expandDoublingDoubBDSel [counter2*2]) lowextValue = expandDoublingDoubBDSel [counter2*2];

**if** (highestValue < expandDoublingDoubBDSel [counter2*2]) highestValue = expandDoublingDoubBDSel [counter2*2];

                                                        }

**if** (siblinFusionDoub1 < randBDRangeSelA) siblinFusionDoub1 = randBDRangeSelA;

**if** (lowextValue != 100000 && highestValue != 0){

**if** ((highestValue+lowextValue)/(**double**)2 > siblinFusionDoub1+10){

                                                                activeCellStatusList [counter1*12+4] = (**int**)(round((highestValue+lowextValue)/(**double**)2));

                                                                activeCellStatusList [counter1*12+5] = 2;

                                                                activeCellStatusList [counter1*12+6] = 1;

                                                                activeCellStatusList [counter1*12+7] = 1;

                                                                activeCellStatusList [counter1*12+9] = 0;

                                                                activeCellStatusList [counter1*12+8] = (**int**)simProcessDataProgHold [18];

                                                            }

**else**{

                                                                activeCellStatusList [counter1*12+4] = siblinFusionDoub1+10;

                                                                activeCellStatusList [counter1*12+5] = 2;

                                                                activeCellStatusList [counter1*12+6] = 1;

                                                                activeCellStatusList [counter1*12+7] = 1;

                                                                activeCellStatusList [counter1*12+9] = 0;

                                                                activeCellStatusList [counter1*12+8] = (**int**)simProcessDataProgHold [18];

                                                            }

                                                        }

**else**{

                                                            activeCellStatusList [counter1*12+4] = siblinFusionDoub1+10;

                                                            activeCellStatusList [counter1*12+5] = 2;

                                                            activeCellStatusList [counter1*12+6] = 1;

                                                            activeCellStatusList [counter1*12+7] = 1;

                                                            activeCellStatusList [counter1*12+9] = 0;

                                                            activeCellStatusList [counter1*12+8] = (**int**)simProcessDataProgHold [18];

                                                        }

                                                    }

                                                }

                                            }

**else** **if** (eventType == 5){

**if** (expandBDCDSelCount == 0){

                                                    randInit = rand() % randCDRangeSelB + randCDRangeSelA;

**if** (siblinFusionCheck1 != 6){

                                                        activeCellStatusList [counter1*12+4] = randInit;

                                                        activeCellStatusList [counter1*12+5] = 3;

                                                        activeCellStatusList [counter1*12+6] = 1;

                                                        activeCellStatusList [counter1*12+9] = 0;

                                                    }

**else**{

                                                        activeCellStatusList [counter1*12+4] = siblinFusionDoub1+10;

                                                        activeCellStatusList [counter1*12+5] = 3;

                                                        activeCellStatusList [counter1*12+6] = 1;

                                                        activeCellStatusList [counter1*12+9] = 0;

                                                    }

                                                }

**else**{

**if** (siblinFusionCheck1 != 6){

**if** (expandBDCDSelCount != 0){

                                                            randInit = rand() % expandBDCDSelCount/2 + 0;

                                                            activeCellStatusList [counter1*12+4] = expandBDCDSel [randInit*2];

                                                            activeCellStatusList [counter1*12+5] = 3;

                                                            activeCellStatusList [counter1*12+6] = 1;

                                                            activeCellStatusList [counter1*12+9] = 0;

                                                        }

**else**{

                                                            activeCellStatusList [counter1*12+4] = siblinFusionDoub1+10;

                                                            activeCellStatusList [counter1*12+5] = 3;

                                                            activeCellStatusList [counter1*12+6] = 1;

                                                            activeCellStatusList [counter1*12+9] = 0;

                                                        }

                                                    }

**else**{

**if** (expandBDCDSelCount != 0){

                                                            randInit = rand() % expandBDCDSelCount/2 + 0;

**if**(expandBDCDSel [randInit*2] > siblinFusionDoub1){

                                                                activeCellStatusList [counter1*12+4] = expandBDCDSel [randInit*2]+10;

                                                                activeCellStatusList [counter1*12+5] = 3;

                                                                activeCellStatusList [counter1*12+6] = 1;

                                                                activeCellStatusList [counter1*12+9] = 0;

                                                            }

**else**{

                                                                activeCellStatusList [counter1*12+4] = siblinFusionDoub1+10;

                                                                activeCellStatusList [counter1*12+5] = 3;

                                                                activeCellStatusList [counter1*12+6] = 1;

                                                                activeCellStatusList [counter1*12+9] = 0;

                                                            }

                                                        }

**else**{

                                                            activeCellStatusList [counter1*12+4] = siblinFusionDoub1+10;

                                                            activeCellStatusList [counter1*12+5] = 3;

                                                            activeCellStatusList [counter1*12+6] = 1;

                                                            activeCellStatusList [counter1*12+9] = 0;

                                                        }

                                                    }

                                                }

                                            }

**else** **if** (eventType == 6){

**if** (expandBDCFSelCount == 0){

                                                    randInit = rand() % randCDRangeSelB + randCDRangeSelA; //------Use CD data

**if** (siblinFusionCheck1 != 6){

                                                        activeCellStatusList [counter1*12+4] = randInit;

                                                        activeCellStatusList [counter1*12+5] = 6;

                                                        activeCellStatusList [counter1*12+6] = 1;

                                                        activeCellStatusList [counter1*12+9] = 0;

                                                    }

**else**{

**if** (siblinFusionDoub1 < randBDRangeSelA) siblinFusionDoub1 = randBDRangeSelA;

                                                        activeCellStatusList [siblingCellNo1*12+4] = siblinFusionDoub1+10;

                                                        activeCellStatusList [counter1*12+4] = siblinFusionDoub1; //-----switch--

                                                        activeCellStatusList [counter1*12+5] = 6;

                                                        activeCellStatusList [counter1*12+6] = 1;

                                                        activeCellStatusList [counter1*12+9] = 0;

                                                    }

                                                }

**else**{

**if** (siblinFusionCheck1 == 0){

**if** (expandBDCFSelCount != 0){

                                                            randInit = rand() % expandBDCFSelCount/2 + 0;

                                                            activeCellStatusList [counter1*12+4] = expandBDCFSel [randInit*2];

                                                            activeCellStatusList [counter1*12+5] = 6;

                                                            activeCellStatusList [counter1*12+6] = 1;

                                                            activeCellStatusList [counter1*12+9] = 0;

                                                        }

**else** {

                                                            activeCellStatusList [counter1*12+4] = siblinFusionDoub1+10;

                                                            activeCellStatusList [counter1*12+5] = 6;

                                                            activeCellStatusList [counter1*12+6] = 1;

                                                            activeCellStatusList [counter1*12+9] = 0;

                                                        }

                                                    }

**else**{

**if** (expandBDCDSelCount != 0){

                                                            randInit = rand() % expandBDCDSelCount/2 + 0;

**if**(expandBDCDSel [randInit*2] > siblinFusionDoub1){

                                                                activeCellStatusList [counter1*12+4] = expandBDCDSel [randInit*2]+10;

                                                                activeCellStatusList [counter1*12+5] = 3;

                                                                activeCellStatusList [counter1*12+6] = 1;

                                                                activeCellStatusList [counter1*12+9] = 0;

                                                            }

**else**{

                                                                activeCellStatusList [counter1*12+4] = siblinFusionDoub1+10;

                                                                activeCellStatusList [counter1*12+5] = 3;

                                                                activeCellStatusList [counter1*12+6] = 1;

                                                                activeCellStatusList [counter1*12+9] = 0;

                                                            }

                                                        }

**else**{

                                                            activeCellStatusList [siblingCellNo1*12+4] = siblinFusionDoub1+10;

                                                            activeCellStatusList [counter1*12+4] = siblinFusionDoub1;

                                                            activeCellStatusList [counter1*12+5] = 6;

                                                            activeCellStatusList [counter1*12+6] = 1;

                                                            activeCellStatusList [counter1*12+9] = 0;

                                                        }

                                                    }

                                                }

                                            }

                                        }

**else** **if** (activeCellStatusList [counter1*12+5] == 2){

                                            siblinFusionCheck1 = 0;

                                            siblinFusionCheck2 = 0;

                                            siblinFusionDoub1 = 0;

                                            siblinFusionDoub2 = 0;

                                            siblingCellNo1 = 0;

                                            siblingCellNo2 = 0;

**for** (**int** counter2 = 0; counter2 < activeCellStatusListCount/12; counter2++){

**if** (activeCellStatusList [counter2*12+6] == 1 && (activeCellStatusList [counter2*12+1] == activeCellStatusList [counter1*12] || activeCellStatusList [counter2*12+2] == activeCellStatusList [counter1*12]) && activeCellStatusList [counter2*12+3] == activeCellStatusList [counter1*12+3]){

**if** (siblinFusionCheck1 == 0 && activeCellStatusList [counter2*12+5] != 0){

                                                        siblinFusionCheck1 = activeCellStatusList [counter2*12+5];

                                                        siblinFusionDoub1 = activeCellStatusList [counter2*12+4];

                                                        siblingCellNo1 = counter2;

                                                    }

**else** **if** (siblinFusionCheck1 != 0 && siblinFusionCheck2 == 0 && activeCellStatusList [counter2*12+5] != 0){

                                                        siblinFusionCheck2 = activeCellStatusList [counter2*12+5];

                                                        siblinFusionDoub2 = activeCellStatusList [counter2*12+4];

                                                        siblingCellNo2 = counter2;

**break**;

                                                    }

                                                }

                                            }

                                            eventType = 0;

**if** (siblinFusionCheck1 == 6 && siblinFusionCheck2 == 6){

**for** (**int** counter2 = 0; counter2 < 100; counter2++){

                                                    randInit = rand() % 100 + 0;

**if** (secondEventTDListSel [randInit] != 10){

                                                        eventType = secondEventTDListSel [randInit];

                                                        loopCheck = 1;

**break**;

                                                    }

                                                }

**if** (loopCheck == 0) eventType = 13;

                                            }

**else**{

                                                randInit = rand() % 100 + 0;

                                                eventType = secondEventTDListSel [randInit];

**if** (eventType == 0) eventType = 13;

                                            }

**if** ((activeCellStatusList [counter1*12+8] == 2 || activeCellStatusList [counter1*12+8] == 3) && activeCellStatusList [counter1*12+8] == activeCellStatusList [counter1*12+7] && eventType == 12){

                                                eventType = 13;

                                            }

**if** (siblinFusionDoub1 < siblinFusionDoub2) siblinFusionDoubLarge = siblinFusionDoub2;

**else** siblinFusionDoubLarge = siblinFusionDoub1;

**if** (siblinFusionCheck1 == 6 && siblinFusionCheck2 == 6 && eventType == 10){

                                                eventType = 13;

                                            }

**if** (eventType == 10){

                                                loopCheck = 0;

**if** (siblinFusionCheck1 != 0 && siblinFusionCheck2 != 0){

**if** (siblinFusionCheck1 == 6 && siblinFusionCheck2 != 6){

**if** (expandTDCFSelCount == 0){

                                                            randInit = rand() % randCDRangeSelB + randCDRangeSelA;

**if** (siblinFusionDoub2 < randBDRangeSelA) siblinFusionDoub2 = randBDRangeSelA;

**if** (siblinFusionDoub1 > siblinFusionDoub2){

**if** (randInit > siblinFusionDoub1){

                                                                    activeCellStatusList [siblingCellNo2*12+4] = randInit+10;

                                                                    activeCellStatusList [counter1*12+4] = siblinFusionDoub2;

                                                                    activeCellStatusList [counter1*12+5] = 6;

                                                                    activeCellStatusList [counter1*12+6] = 1;

                                                                }

**else**{

                                                                    activeCellStatusList [siblingCellNo2*12+4] = siblinFusionDoub1+10;

                                                                    activeCellStatusList [counter1*12+4] = siblinFusionDoub2;

                                                                    activeCellStatusList [counter1*12+5] = 6;

                                                                    activeCellStatusList [counter1*12+6] = 1;

                                                                }

                                                            }

**else** **if** (siblinFusionDoub1 < siblinFusionDoub2){

**if** (randInit > siblinFusionDoub2){

                                                                    activeCellStatusList [siblingCellNo2*12+4] = randInit+10;

                                                                    activeCellStatusList [counter1*12+4] = siblinFusionDoub2;

                                                                    activeCellStatusList [counter1*12+5] = 6;

                                                                    activeCellStatusList [counter1*12+6] = 1;

                                                                }

**else**{

                                                                    activeCellStatusList [siblingCellNo2*12+4] = siblinFusionDoub2+10;

                                                                    activeCellStatusList [counter1*12+4] = siblinFusionDoub2;

                                                                    activeCellStatusList [counter1*12+5] = 6;

                                                                    activeCellStatusList [counter1*12+6] = 1;

                                                                }

                                                            }

                                                        }

**else**{

**if** (siblinFusionDoub1 > siblinFusionDoub2){

**if** (expandTDCFSelCount != 0){

                                                                    randInit = rand() % expandTDCFSelCount/2 + 0;

**if** (expandTDCFSel [randInit*2] > siblinFusionDoub1){

                                                                        activeCellStatusList [siblingCellNo2*12+4] = expandTDCFSel [randInit*2]+10;

                                                                        activeCellStatusList [counter1*12+4] = siblinFusionDoub2;

                                                                        activeCellStatusList [counter1*12+5] = 6;

                                                                        activeCellStatusList [counter1*12+6] = 1;

                                                                    }

**else**{

                                                                        activeCellStatusList [siblingCellNo2*12+4] = siblinFusionDoub1+10;

                                                                        activeCellStatusList [counter1*12+4] = siblinFusionDoub2;

                                                                        activeCellStatusList [counter1*12+5] = 6;

                                                                        activeCellStatusList [counter1*12+6] = 1;

                                                                    }

                                                                }

**else**{

                                                                    activeCellStatusList [siblingCellNo2*12+4] = siblinFusionDoub1+10;

                                                                    activeCellStatusList [counter1*12+4] = siblinFusionDoub2;

                                                                    activeCellStatusList [counter1*12+5] = 6;

                                                                    activeCellStatusList [counter1*12+6] = 1;

                                                                }

                                                            }

**else** **if** (siblinFusionDoub1 < siblinFusionDoub2){

**if** (expandTDCFSelCount != 0){

                                                                    randInit = rand() % expandTDCFSelCount/2 + 0;

**if** (expandTDCFSel [randInit*2] > siblinFusionDoub2){

                                                                        activeCellStatusList [siblingCellNo2*12+4] = expandTDCFSel [randInit*2]+10;

                                                                        activeCellStatusList [counter1*12+4] = siblinFusionDoub2;

                                                                        activeCellStatusList [counter1*12+5] = 6;

                                                                        activeCellStatusList [counter1*12+6] = 1;

                                                                    }

**else**{

                                                                        activeCellStatusList [siblingCellNo2*12+4] = siblinFusionDoub2+10;

                                                                        activeCellStatusList [counter1*12+4] = siblinFusionDoub2;

                                                                        activeCellStatusList [counter1*12+5] = 6;

                                                                        activeCellStatusList [counter1*12+6] = 1;

                                                                    }

                                                                }

**else**{

                                                                    activeCellStatusList [siblingCellNo2*12+4] = siblinFusionDoub2+10;

                                                                    activeCellStatusList [counter1*12+4] = siblinFusionDoub2;

                                                                    activeCellStatusList [counter1*12+5] = 6;

                                                                    activeCellStatusList [counter1*12+6] = 1;

                                                                }

                                                            }

                                                        }

                                                    }

**else** **if** (siblinFusionCheck1 != 6 && siblinFusionCheck2 == 6){

**if** (expandTDCFSelCount == 0){

                                                            randInit = rand() % randCDRangeSelB + randCDRangeSelA;

**if** (siblinFusionDoub1 > siblinFusionDoub2){

**if** (randInit > siblinFusionDoub1){

                                                                    activeCellStatusList [siblingCellNo1*12+4] = randInit+10;

                                                                    activeCellStatusList [counter1*12+4] = siblinFusionDoub1;

                                                                    activeCellStatusList [counter1*12+5] = 6;

                                                                    activeCellStatusList [counter1*12+6] = 1;

                                                                }

**else**{

                                                                    activeCellStatusList [siblingCellNo1*12+4] = siblinFusionDoub1+10;

                                                                    activeCellStatusList [counter1*12+4] = siblinFusionDoub1;

                                                                    activeCellStatusList [counter1*12+5] = 6;

                                                                    activeCellStatusList [counter1*12+6] = 1;

                                                                }

                                                            }

**else** **if** (siblinFusionDoub1 < siblinFusionDoub2){

**if** (randInit > siblinFusionDoub2){

                                                                    activeCellStatusList [siblingCellNo1*12+4] = randInit+10;

                                                                    activeCellStatusList [counter1*12+4] = siblinFusionDoub1;

                                                                    activeCellStatusList [counter1*12+5] = 6;

                                                                    activeCellStatusList [counter1*12+6] = 1;

                                                                }

**else**{

                                                                    activeCellStatusList [siblingCellNo1*12+4] = siblinFusionDoub2+10;

                                                                    activeCellStatusList [counter1*12+4] = siblinFusionDoub1;

                                                                    activeCellStatusList [counter1*12+5] = 6;

                                                                    activeCellStatusList [counter1*12+6] = 1;

                                                                }

                                                            }

                                                        }

**else**{

**if** (siblinFusionDoub1 > siblinFusionDoub2){

**if** (expandTDCFSelCount != 0){

                                                                    randInit = rand() % expandTDCFSelCount/2 + 0;

**if** (expandTDCFSel [randInit*2] > siblinFusionDoub1){

                                                                        activeCellStatusList [siblingCellNo1*12+4] = expandTDCFSel [randInit*2]+10;

                                                                        activeCellStatusList [counter1*12+4] = siblinFusionDoub1;

                                                                        activeCellStatusList [counter1*12+5] = 6;

                                                                        activeCellStatusList [counter1*12+6] = 1;

                                                                    }

**else**{

                                                                        activeCellStatusList [siblingCellNo1*12+4] = siblinFusionDoub1+10;

                                                                        activeCellStatusList [counter1*12+4] = siblinFusionDoub1;

                                                                        activeCellStatusList [counter1*12+5] = 6;

                                                                        activeCellStatusList [counter1*12+6] = 1;

                                                                    }

                                                                }

**else**{

                                                                    activeCellStatusList [siblingCellNo1*12+4] = siblinFusionDoub1+10;

                                                                    activeCellStatusList [counter1*12+4] = siblinFusionDoub1;

                                                                    activeCellStatusList [counter1*12+5] = 6;

                                                                    activeCellStatusList [counter1*12+6] = 1;

                                                                }

                                                            }

**else** **if** (siblinFusionDoub1 < siblinFusionDoub2){

**if** (expandTDCFSelCount != 0){

                                                                    randInit = rand() % expandTDCFSelCount/2 + 0;

**if** (expandTDCFSel [randInit*2] >= siblinFusionDoub2){

                                                                        activeCellStatusList [siblingCellNo1*12+4] = expandTDCFSel [randInit*2]+10;

                                                                        activeCellStatusList [counter1*12+4] = siblinFusionDoub1;

                                                                        activeCellStatusList [counter1*12+5] = 6;

                                                                        activeCellStatusList [counter1*12+6] = 1;

                                                                    }

**else**{

                                                                        activeCellStatusList [siblingCellNo1*12+4] = siblinFusionDoub2+10;

                                                                        activeCellStatusList [counter1*12+4] = siblinFusionDoub1;

                                                                        activeCellStatusList [counter1*12+5] = 6;

                                                                        activeCellStatusList [counter1*12+6] = 1;

                                                                    }

                                                                }

**else**{

                                                                    activeCellStatusList [siblingCellNo1*12+4] = siblinFusionDoub2+10;

                                                                    activeCellStatusList [counter1*12+4] = siblinFusionDoub1;

                                                                    activeCellStatusList [counter1*12+5] = 6;

                                                                    activeCellStatusList [counter1*12+6] = 1;

                                                                }

                                                            }

                                                        }

                                                    }

**else** **if** (siblinFusionCheck1 != 6 && siblinFusionCheck2 != 6){

                                                        activeCellStatusList [siblingCellNo1*12+4] = siblinFusionDoub1+10;

                                                        activeCellStatusList [siblingCellNo2*12+4] = siblinFusionDoub2+10;

                                                        activeCellStatusList [counter1*12+4] = siblinFusionDoub2;

                                                        activeCellStatusList [counter1*12+5] = 6;

                                                        activeCellStatusList [counter1*12+6] = 1;

                                                    }

                                                }

**else** **if** (siblinFusionCheck1 != 0 && siblinFusionCheck2 == 0){

**if** (expandTDCFSelCount == 0){

                                                        randInit = rand() % randCDRangeSelB + randCDRangeSelA;

**if** (randInit > siblinFusionDoub1+10){

                                                            activeCellStatusList [counter1*12+4] = randInit;

                                                            activeCellStatusList [counter1*12+5] = 6;

                                                            activeCellStatusList [counter1*12+6] = 1;

                                                        }

**else**{

                                                            activeCellStatusList [counter1*12+4] = siblinFusionDoub1+5;

                                                            activeCellStatusList [counter1*12+5] = 6;

                                                            activeCellStatusList [counter1*12+6] = 1;

                                                        }

                                                    }

**else**{

**if** (expandTDCFSelCount != 0){

                                                            randInit = rand() % expandTDCFSelCount/2 + 0;

**if** (expandTDCFSel [randInit*2] > siblinFusionDoub1+10){

                                                                activeCellStatusList [counter1*12+4] = expandTDCFSel [randInit*2];

                                                                activeCellStatusList [counter1*12+5] = 6;

                                                                activeCellStatusList [counter1*12+6] = 1;

                                                            }

**else**{

                                                                activeCellStatusList [counter1*12+4] = siblinFusionDoub1+5;

                                                                activeCellStatusList [counter1*12+5] = 6;

                                                                activeCellStatusList [counter1*12+6] = 1;

                                                            }

                                                        }

**else**{

                                                            activeCellStatusList [counter1*12+4] = siblinFusionDoub1+5;

                                                            activeCellStatusList [counter1*12+5] = 6;

                                                            activeCellStatusList [counter1*12+6] = 1;

                                                        }

                                                    }

                                                }

**else** **if** (siblinFusionCheck1 == 0 && siblinFusionCheck2 == 0){

**if** (expandTDCFSelCount == 0){

                                                        randInit = rand() % randCDRangeSelB + randCDRangeSelA;

                                                        activeCellStatusList [counter1*12+4] = randInit;

                                                        activeCellStatusList [counter1*12+5] = 6;

                                                        activeCellStatusList [counter1*12+6] = 1;

                                                    }

**else**{

**if** (activeCellStatusList [counter1*12+11] == 0){

                                                            randInit = rand() % expandTDCFSelCount/2 + 0;

                                                            activeCellStatusList [counter1*12+4] = expandTDCFSel [randInit*2];

                                                            activeCellStatusList [counter1*12+5] = 6;

                                                            activeCellStatusList [counter1*12+6] = 1;

                                                        }

**else**{

                                                            randInit = rand() % expandTDCFSelCount/2 + 0;

                                                            activeCellStatusList [counter1*12+4] = expandTDCFSel [randInit*2];

                                                            activeCellStatusList [counter1*12+5] = 6;

                                                            activeCellStatusList [counter1*12+6] = 1;

                                                        }

                                                    }

                                                }

                                            }

**else** **if** (eventType == 11){

**if** (expandDoublingDoubTDSelCount == 0){

                                                    randInit = rand() % randBDRangeSelB + randBDRangeSelA;

**if** (randInit > siblinFusionDoubLarge+10){

                                                        activeCellStatusList [counter1*12+4] = randInit;

                                                        activeCellStatusList [counter1*12+5] = 1;

                                                        activeCellStatusList [counter1*12+6] = 1;

                                                        activeCellStatusList [counter1*12+9] = (**int**)simProcessDataProgHold [19];

                                                        activeCellStatusList [counter1*12+7] = 0;

                                                    }

**else**{

**if** (siblinFusionDoubLarge < randBDRangeSelA) siblinFusionDoubLarge = randBDRangeSelA;

                                                        activeCellStatusList [counter1*12+4] = siblinFusionDoubLarge+10;

                                                        activeCellStatusList [counter1*12+5] = 1;

                                                        activeCellStatusList [counter1*12+6] = 1;

                                                        activeCellStatusList [counter1*12+9] = (**int**)simProcessDataProgHold [19];

                                                        activeCellStatusList [counter1*12+7] = 0;

                                                    }

                                                }

**else**{

**if** (activeCellStatusList [counter1*12+11] == 0){

**if** (expandDoublingDoubTDSelCount != 0){

                                                            randInit = rand() % expandDoublingDoubTDSelCount/2 + 0;

**if** (randInit > siblinFusionDoubLarge+10){

                                                                activeCellStatusList [counter1*12+4] = expandDoublingDoubTDSel [randInit*2];

                                                                activeCellStatusList [counter1*12+5] = 1;

                                                                activeCellStatusList [counter1*12+6] = 1;

                                                                activeCellStatusList [counter1*12+9] = (**int**)simProcessDataProgHold [19];

                                                                activeCellStatusList [counter1*12+7] = 0;

                                                            }

**else**{

**if** (siblinFusionDoubLarge < randBDRangeSelA) siblinFusionDoubLarge = randBDRangeSelA;

                                                                activeCellStatusList [counter1*12+4] = siblinFusionDoubLarge+10;

                                                                activeCellStatusList [counter1*12+5] = 1;

                                                                activeCellStatusList [counter1*12+6] = 1;

                                                                activeCellStatusList [counter1*12+9] = (**int**)simProcessDataProgHold [19];

                                                                activeCellStatusList [counter1*12+7] = 0;

                                                            }

                                                        }

**else**{

**if** (siblinFusionDoubLarge < randBDRangeSelA) siblinFusionDoubLarge = randBDRangeSelA;

                                                            activeCellStatusList [counter1*12+4] = siblinFusionDoubLarge+10;

                                                            activeCellStatusList [counter1*12+5] = 1;

                                                            activeCellStatusList [counter1*12+6] = 1;

                                                            activeCellStatusList [counter1*12+9] = (**int**)simProcessDataProgHold [19];

                                                            activeCellStatusList [counter1*12+7] = 0;

                                                        }

                                                    }

**else**{

**if** (expandDoublingDoubTDSelCount != 0){

                                                            randInit = rand() % expandDoublingDoubTDSelCount/2 + 0;

**if** (expandDoublingDoubTDSel [randInit*2] > siblinFusionDoubLarge+10){

                                                                activeCellStatusList [counter1*12+4] = expandDoublingDoubTDSel [randInit*2];

                                                                activeCellStatusList [counter1*12+5] = 1;

                                                                activeCellStatusList [counter1*12+6] = 1;

                                                                activeCellStatusList [counter1*12+9] = (**int**)simProcessDataProgHold [19];

                                                                activeCellStatusList [counter1*12+7] = 0;

                                                            }

**else**{

**if** (siblinFusionDoubLarge < randBDRangeSelA) siblinFusionDoubLarge = randBDRangeSelA;

                                                                activeCellStatusList [counter1*12+4] = siblinFusionDoubLarge+10;

                                                                activeCellStatusList [counter1*12+5] = 1;

                                                                activeCellStatusList [counter1*12+6] = 1;

                                                                activeCellStatusList [counter1*12+9] = (**int**)simProcessDataProgHold [19];

                                                                activeCellStatusList [counter1*12+7] = 0;

                                                            }

                                                        }

**else**{

**if** (siblinFusionDoubLarge < randBDRangeSelA) siblinFusionDoubLarge = randBDRangeSelA;

                                                            activeCellStatusList [counter1*12+4] = siblinFusionDoubLarge+10;

                                                            activeCellStatusList [counter1*12+5] = 1;

                                                            activeCellStatusList [counter1*12+6] = 1;

                                                            activeCellStatusList [counter1*12+9] = (**int**)simProcessDataProgHold [19];

                                                            activeCellStatusList [counter1*12+7] = 0;

                                                        }

                                                    }

                                                }

                                            }

**else** **if** (eventType == 12){

**if** (expandDoublingDoubTDSelCount == 0){

                                                    randInit = rand() % randBDRangeSelB + randBDRangeSelA;

**if** (randInit > siblinFusionDoubLarge+10){

                                                        activeCellStatusList [counter1*12+4] = randInit;

                                                        activeCellStatusList [counter1*12+5] = 2;

                                                        activeCellStatusList [counter1*12+6] = 1;

                                                        activeCellStatusList [counter1*12+7]++;

                                                    }

**else**{

**if** (siblinFusionDoubLarge < randBDRangeSelA) siblinFusionDoubLarge = randBDRangeSelA;

                                                        activeCellStatusList [counter1*12+4] = siblinFusionDoubLarge+10;

                                                        activeCellStatusList [counter1*12+5] = 2;

                                                        activeCellStatusList [counter1*12+6] = 1;

                                                        activeCellStatusList [counter1*12+7]++;

                                                    }

                                                }

**else**{

**if** (activeCellStatusList [counter1*12+11] == 0){

**if** (expandDoublingDoubTDSelCount != 0){

                                                            randInit = rand() % expandDoublingDoubTDSelCount/2 + 0;

**if** (randInit >= siblinFusionDoubLarge+10){

                                                                activeCellStatusList [counter1*12+4] = expandDoublingDoubTDSel [randInit*2];

                                                                activeCellStatusList [counter1*12+5] = 2;

                                                                activeCellStatusList [counter1*12+6] = 1;

                                                                activeCellStatusList [counter1*12+7]++;

                                                            }

**else**{

**if** (siblinFusionDoubLarge < randBDRangeSelA) siblinFusionDoubLarge = randBDRangeSelA;

                                                                activeCellStatusList [counter1*12+4] = siblinFusionDoubLarge+10;

                                                                activeCellStatusList [counter1*12+5] = 2;

                                                                activeCellStatusList [counter1*12+6] = 1;

                                                                activeCellStatusList [counter1*12+7]++;

                                                            }

                                                        }

**else**{

**if** (siblinFusionDoubLarge < randBDRangeSelA) siblinFusionDoubLarge = randBDRangeSelA;

                                                            activeCellStatusList [counter1*12+4] = siblinFusionDoubLarge+10;

                                                            activeCellStatusList [counter1*12+5] = 2;

                                                            activeCellStatusList [counter1*12+6] = 1;

                                                            activeCellStatusList [counter1*12+7]++;

                                                        }

                                                    }

**else**{

**if** (expandDoublingDoubTDSelCount != 0){

                                                            randInit = rand() % expandDoublingDoubTDSelCount/2 + 0;

**if** (expandDoublingDoubTDSel [randInit*2] > siblinFusionDoubLarge+10){

                                                                activeCellStatusList [counter1*12+4] = expandDoublingDoubTDSel [randInit*2];

                                                                activeCellStatusList [counter1*12+5] = 2;

                                                                activeCellStatusList [counter1*12+7]++;

                                                            }

**else**{

**if** (siblinFusionDoubLarge < randBDRangeSelA) siblinFusionDoubLarge = randBDRangeSelA;

                                                                activeCellStatusList [counter1*12+4] = siblinFusionDoubLarge+10;

                                                                activeCellStatusList [counter1*12+5] = 2;

                                                                activeCellStatusList [counter1*12+7]++;

                                                            }

                                                        }

**else**{

**if** (siblinFusionDoubLarge < randBDRangeSelA) siblinFusionDoubLarge = randBDRangeSelA;

                                                            activeCellStatusList [counter1*12+4] = siblinFusionDoubLarge+10;

                                                            activeCellStatusList [counter1*12+5] = 2;

                                                            activeCellStatusList [counter1*12+7]++;

                                                        }

                                                    }

                                                }

                                            }

**else** **if** (eventType == 13){

**if** (expandTDCDSelCount == 0){

                                                    randInit = rand() % randCDRangeSelB + randCDRangeSelA;

**if** (randInit > siblinFusionDoubLarge+10){

                                                        activeCellStatusList [counter1*12+4] = randInit;

                                                        activeCellStatusList [counter1*12+5] = 3;

                                                        activeCellStatusList [counter1*12+6] = 1;

                                                    }

**else**{

                                                        activeCellStatusList [counter1*12+4] = siblinFusionDoubLarge+10;

                                                        activeCellStatusList [counter1*12+5] = 3;

                                                        activeCellStatusList [counter1*12+6] = 1;

                                                    }

                                                }

**else**{

**if** (expandTDCDSelCount != 0){

                                                        randInit = rand() % expandTDCDSelCount/2 + 0;

**if** (expandTDCDSel [randInit*2] > siblinFusionDoubLarge+10){

                                                            activeCellStatusList [counter1*12+4] = expandTDCDSel [randInit*2];

                                                            activeCellStatusList [counter1*12+5] = 3;

                                                            activeCellStatusList [counter1*12+6] = 1;

                                                        }

**else**{

                                                            activeCellStatusList [counter1*12+4] = siblinFusionDoubLarge+10;

                                                            activeCellStatusList [counter1*12+5] = 3;

                                                            activeCellStatusList [counter1*12+6] = 1;

                                                        }

                                                    }

**else**{

                                                        activeCellStatusList [counter1*12+4] = siblinFusionDoubLarge+10;

                                                        activeCellStatusList [counter1*12+5] = 3;

                                                        activeCellStatusList [counter1*12+6] = 1;

                                                    }

                                                }

                                            }

                                        }

                                    }

                                }

                            }

**if** (terminate2 == 0){ //====terminate2 == 1: for forced exit====

**for** (**int** counter1 = 0; counter1 < activeCellStatusListCount/12; counter1++){

                                    activeCellStatusList [counter1*12+6] = 0;

                                }

                                siblingCellPosition1 = 0;

                                siblingCellPosition2 = 0;

                                //====Fusion position adjustment. The above process assigned the fusion-end of a cell (event type 91). This end is fused to another cell. Following the process find such cell and enter Event type 92. This process also checks the order of cell fusion.====

**for** (**int** counter1 = 0; counter1 < activeCellStatusListCount/12; counter1++){

**if** (terminateSimFlag == 1){

                                        terminate2 = 1;

**break**;

                                    }

                                    selectCheck = 0;

**if** (doseSimStatusHold == 2){

**for** (**int** counter3 = 0; counter3 < lingNoAssigineSimCount; counter3++){

**if** (lingNoAssigineSim [counter3] == activeCellStatusList [counter1*12+3]){

                                                selectCheck = 1;

**break**;

                                            }

                                        }

                                    }

**if** (selectCheck == 0){

**if** (activeCellStatusList [counter1*12+5] == 6){

**if** (activeCellStatusList [counter1*12+2] == 0){

                                                siblinFusionDoub1 = 0;

                                                siblingCellNo1 = 0;

**for** (**int** counter2 = 0; counter2 < activeCellStatusListCount/12; counter2++){

**if** (activeCellStatusList [counter2*12+1] == activeCellStatusList [counter1*12] && activeCellStatusList [counter2*12+3] == activeCellStatusList [counter1*12+3]){

                                                        siblingCellNo1 = activeCellStatusList [counter2*12];

                                                        siblinFusionDoub1 = activeCellStatusList [counter2*12+4];

                                                        siblingCellPosition1 = counter2;

                                                        fusionCount++;

**break**;

                                                    }

                                                }

                                                randInit = rand() % 100 + 0;

**if** (secondEventBDCFList [randInit] == 0){

                                                    activeCellStatusList [counter1*12+6] = siblingCellNo1;

                                                    fusionCount++;

                                                }

**else** **if** (secondEventBDCFList [randInit] == 7){

                                                    loopCheck = 0;

**if** (expandDoublingDoubCFCount != 0){

                                                        randInit = rand() % expandDoublingDoubCFCount/2 + 0;

**if** (expandDoublingDoubCF [randInit*2] > siblinFusionDoub1){

                                                            activeCellStatusList [siblingCellPosition1*12+4] = expandDoublingDoubCF [randInit*2];

                                                            activeCellStatusList [siblingCellPosition1*12+5] = 1;

                                                            activeCellStatusList [counter1*12+6] = siblingCellNo1;

                                                            activeCellStatusList [siblingCellPosition1*12+7] = 0;

                                                            activeCellStatusList [siblingCellPosition1*12+8] = 0;

                                                            fusionCount++;

                                                            loopCheck = 1;

                                                        }

**else**{

                                                            activeCellStatusList [siblingCellPosition1*12+5] = 1;

                                                            activeCellStatusList [counter1*12+6] = siblingCellNo1;

                                                            activeCellStatusList [siblingCellPosition1*12+7] = 0;

                                                            activeCellStatusList [siblingCellPosition1*12+8] = 0;

                                                            fusionCount++;

                                                            loopCheck = 1;

                                                        }

                                                    }

**if** (loopCheck == 0){

                                                        activeCellStatusList [siblingCellPosition1*12+5] = 1;

                                                        activeCellStatusList [counter1*12+6] = siblingCellNo1;

                                                        activeCellStatusList [siblingCellPosition1*12+7] = 0;

                                                        activeCellStatusList [siblingCellPosition1*12+8] = 0;

                                                        fusionCount++;

                                                    }

                                                }

**else** **if** (secondEventBDCFList [randInit] == 8){

                                                    loopCheck = 0;

**if** (expandDoublingDoubCFCount != 0){

                                                        randInit = rand() % expandDoublingDoubCFCount/2 + 0;

**if** (expandDoublingDoubCF [randInit*2] > siblinFusionDoub1){

**if** (activeCellStatusList [siblingCellPosition1*12+5] != 2) activeCellStatusList [siblingCellPosition1*12+7]++;

                                                            activeCellStatusList [siblingCellPosition1*12+4] = expandDoublingDoubCF [randInit*2];

                                                            activeCellStatusList [siblingCellPosition1*12+5] = 2;

                                                            activeCellStatusList [counter1*12+6] = siblingCellNo1;

                                                            activeCellStatusList [siblingCellPosition1*12+8] = (**int**)simProcessDataBaseHold [18];

                                                            fusionCount++;

                                                            loopCheck = 1;

                                                        }

**else**{

**if** (activeCellStatusList [siblingCellPosition1*12+5] != 2) activeCellStatusList [siblingCellPosition1*12+7]++;

                                                            activeCellStatusList [siblingCellPosition1*12+5] = 2;

                                                            activeCellStatusList [counter1*12+6] = siblingCellNo1;

                                                            activeCellStatusList [siblingCellPosition1*12+8] = (**int**)simProcessDataBaseHold [18];

                                                            fusionCount++;

                                                            loopCheck = 1;

                                                        }

                                                    }

**if** (loopCheck == 0){

**if** (activeCellStatusList [siblingCellPosition1*12+5] != 2) activeCellStatusList [siblingCellPosition1*12+7]++;

                                                        activeCellStatusList [siblingCellPosition1*12+5] = 2;

                                                        activeCellStatusList [counter1*12+6] = siblingCellNo1;

                                                        activeCellStatusList [siblingCellPosition1*12+8] = (**int**)simProcessDataBaseHold [18];

                                                        fusionCount++;

                                                    }

                                                }

**else** **if** (secondEventBDCFList [randInit] == 9){

**if** (expandBDCFCDCount != 0){

                                                        randInit = rand() % expandBDCFCDCount/2 + 0;

**if** (expandBDCFCD [randInit*2] > siblinFusionDoub1){

                                                            activeCellStatusList [siblingCellPosition1*12+4] = expandBDCFCD [randInit*2];

                                                            activeCellStatusList [siblingCellPosition1*12+5] = 3;

                                                            activeCellStatusList [counter1*12+6] = siblingCellNo1;

                                                            fusionCount++;

                                                        }

**else**{

                                                            activeCellStatusList [siblingCellPosition1*12+5] = 3;

                                                            activeCellStatusList [counter1*12+6] = siblingCellNo1;

                                                            fusionCount++;

                                                        }

                                                    }

**else**{

                                                        activeCellStatusList [siblingCellPosition1*12+5] = 3;

                                                        activeCellStatusList [counter1*12+6] = siblingCellNo1;

                                                        fusionCount++;

                                                    }

                                                }

                                            }

**else**{

                                                siblinFusionCheck1 = 0;

                                                siblinFusionCheck2 = 0;

                                                siblinFusionDoub1 = 0;

                                                siblinFusionDoub2 = 0;

                                                siblingCellNo1 = 0;

                                                siblingCellNo2 = 0;

**for** (**int** counter2 = 0; counter2 < activeCellStatusListCount/12; counter2++){

**if** ((activeCellStatusList [counter2*12+1] == activeCellStatusList [counter1*12] || activeCellStatusList [counter2*12+2] == activeCellStatusList [counter1*12]) && activeCellStatusList [counter2*12+3] == activeCellStatusList [counter1*12+3]){

**if** (siblinFusionCheck1 == 0 && activeCellStatusList [counter2*12+5] != 0){

                                                            siblinFusionCheck1 = activeCellStatusList [counter2*12+5];

                                                            siblinFusionDoub1 = activeCellStatusList [counter2*12+4];

                                                            siblingCellNo1 = activeCellStatusList [counter2*12];

                                                            siblingCellPosition1 = counter2;

                                                        }

**else** **if** (siblinFusionCheck1 != 0 && siblinFusionCheck2 == 0 && activeCellStatusList [counter2*12+5] != 0){

                                                            siblinFusionCheck2 = activeCellStatusList [counter2*12+5];

                                                            siblinFusionDoub2 = activeCellStatusList [counter2*12+4];

                                                            siblingCellNo2 = activeCellStatusList [counter2*12];

                                                            siblingCellPosition2 = counter2;

**break**;

                                                        }

                                                    }

                                                }

**if** (siblinFusionCheck1 == 6 && siblinFusionCheck2 != 6){

                                                    randInit = rand() % 100 + 0;

**if** (secondEventTDCFList [randInit] == 0){

                                                        activeCellStatusList [siblingCellPosition1*12+6] = siblingCellNo2;

                                                        activeCellStatusList [counter1*12+6] = siblingCellNo2;

                                                        fusionCount++;

                                                    }

**else**{

**if** ((activeCellStatusList [counter1*12+8] == 2 || activeCellStatusList [counter1*12+8] == 3) && activeCellStatusList [counter1*12+8] == activeCellStatusList [counter1*12+7] && secondEventTDCFList [randInit] == 15){

                                                            selectChange = 16;

                                                        }

**else** selectChange = secondEventTDCFList [randInit];

**if** (selectChange == 14){

                                                            loopCheck = 0;

**if** (expandDoublingDoubCFCount != 0){

                                                                randInit = rand() % expandDoublingDoubCFCount/2 + 0;

**if** (expandDoublingDoubCF [randInit*2] > siblinFusionDoub2){

                                                                    activeCellStatusList [siblingCellPosition2*12+4] = expandDoublingDoubCF [randInit*2];

                                                                    activeCellStatusList [siblingCellPosition2*12+5] = 1;

                                                                    activeCellStatusList [siblingCellPosition1*12+6] = siblingCellNo2;

                                                                    activeCellStatusList [counter1*12+6] = siblingCellNo2;

                                                                    activeCellStatusList [siblingCellPosition2*12+7] = 0;

                                                                    activeCellStatusList [siblingCellPosition2*12+8] = 0;

                                                                    fusionCount++;

                                                                    loopCheck = 1;

                                                                }

**else**{

                                                                    activeCellStatusList [siblingCellPosition2*12+5] = 1;

                                                                    activeCellStatusList [siblingCellPosition1*12+6] = siblingCellNo2;

                                                                    activeCellStatusList [counter1*12+6] = siblingCellNo2;

                                                                    activeCellStatusList [siblingCellPosition2*12+7] = 0;

                                                                    activeCellStatusList [siblingCellPosition2*12+8] = 0;

                                                                    fusionCount++;

                                                                    loopCheck = 1;

                                                                }

                                                            }

**if** (loopCheck == 0){

                                                                activeCellStatusList [siblingCellPosition2*12+5] = 1;

                                                                activeCellStatusList [siblingCellPosition1*12+6] = siblingCellNo2;

                                                                activeCellStatusList [counter1*12+6] = siblingCellNo2;

                                                                activeCellStatusList [siblingCellPosition2*12+7] = 0;

                                                                activeCellStatusList [siblingCellPosition2*12+8] = 0;

                                                                fusionCount++;

                                                            }

                                                        }

**else** **if** (selectChange == 15){

                                                            loopCheck = 0;

**if** (expandDoublingDoubCFCount != 0){

                                                                randInit = rand() % expandDoublingDoubCFCount/2 + 0;

**if** (expandDoublingDoubCF [randInit*2] > siblinFusionDoub2){

**if** (activeCellStatusList [siblingCellPosition2*12+5] != 2) activeCellStatusList [siblingCellPosition2*12+7]++;

                                                                    activeCellStatusList [siblingCellPosition2*12+4] = expandDoublingDoubCF [randInit*2];

                                                                    activeCellStatusList [siblingCellPosition2*12+5] = 2;

                                                                    activeCellStatusList [siblingCellPosition1*12+6] = siblingCellNo2;

                                                                    activeCellStatusList [counter1*12+6] = siblingCellNo2;

                                                                    activeCellStatusList [siblingCellPosition2*12+8] = (**int**)simProcessDataBaseHold [18];

                                                                    fusionCount++;

                                                                    loopCheck = 1;

                                                                }

**else**{

**if** (activeCellStatusList [siblingCellPosition2*12+5] != 2) activeCellStatusList [siblingCellPosition2*12+7]++;

                                                                    activeCellStatusList [siblingCellPosition2*12+5] = 2;

                                                                    activeCellStatusList [siblingCellPosition1*12+6] = siblingCellNo2;

                                                                    activeCellStatusList [counter1*12+6] = siblingCellNo2;

                                                                    activeCellStatusList [siblingCellPosition2*12+8] = (**int**)simProcessDataBaseHold [18];

                                                                    fusionCount++;

                                                                    loopCheck = 1;

                                                                }

                                                            }

**if** (loopCheck == 0){

**if** (activeCellStatusList [siblingCellPosition2*12+5] != 2) activeCellStatusList [siblingCellPosition2*12+7]++;

                                                                activeCellStatusList [siblingCellPosition2*12+5] = 2;

                                                                activeCellStatusList [siblingCellPosition1*12+6] = siblingCellNo2;

                                                                activeCellStatusList [counter1*12+6] = siblingCellNo2;

                                                                activeCellStatusList [siblingCellPosition2*12+8] = (**int**)simProcessDataBaseHold [18];

                                                                fusionCount++;

                                                            }

                                                        }

**else** **if** (selectChange == 16){

**if** (expandTDCFCDCount != 0){

                                                                randInit = rand() % expandTDCFCDCount/2 + 0;

**if** (expandTDCFCD [randInit*2] > siblinFusionDoub2){

                                                                    activeCellStatusList [siblingCellPosition2*12+4] = expandTDCFCD [randInit*2];

                                                                    activeCellStatusList [siblingCellPosition2*12+5] = 3;

                                                                    activeCellStatusList [siblingCellPosition1*12+6] = siblingCellNo2;

                                                                    activeCellStatusList [counter1*12+6] = siblingCellNo2;

                                                                    fusionCount++;

                                                                }

**else**{

                                                                    activeCellStatusList [siblingCellPosition2*12+5] = 3;

                                                                    activeCellStatusList [siblingCellPosition1*12+6] = siblingCellNo2;

                                                                    activeCellStatusList [counter1*12+6] = siblingCellNo2;

                                                                    fusionCount++;

                                                                }

                                                            }

**else**{

                                                                activeCellStatusList [siblingCellPosition2*12+5] = 3;

                                                                activeCellStatusList [siblingCellPosition1*12+6] = siblingCellNo2;

                                                                activeCellStatusList [counter1*12+6] = siblingCellNo2;

                                                                fusionCount++;

                                                            }

                                                        }

                                                    }

                                                }

**else** **if** (siblinFusionCheck1 != 6 && siblinFusionCheck2 == 6){ //--------sib1 is one to be changed--

                                                    randInit = rand() % 100 + 0;

**if** (secondEventTDCFList [randInit] == 0){

                                                        activeCellStatusList [siblingCellPosition2*12+6] = siblingCellNo1;

                                                        activeCellStatusList [counter1*12+6] = siblingCellNo1;

                                                        fusionCount++;

                                                    }

**else**{

**if** ((activeCellStatusList [counter1*12+8] == 2 || activeCellStatusList [counter1*12+8] == 3) && activeCellStatusList [counter1*12+8] == activeCellStatusList [counter1*12+7] && secondEventTDCFList [randInit] == 15){

                                                            selectChange = 16;

                                                        }

**else** selectChange = secondEventTDCFList [randInit];

**if** (selectChange == 14){

                                                            loopCheck = 0;

**if** (expandDoublingDoubCFCount != 0){

                                                                randInit = rand() % expandDoublingDoubCFCount/2 + 0;

**if** (expandDoublingDoubCF [randInit*2] > siblinFusionDoub1){

                                                                    activeCellStatusList [siblingCellPosition1*12+4] = expandDoublingDoubCF [randInit*2];

                                                                    activeCellStatusList [siblingCellPosition1*12+5] = 1;

                                                                    activeCellStatusList [siblingCellPosition2*12+6] = siblingCellNo1;

                                                                    activeCellStatusList [counter1*12+6] = siblingCellNo1;

                                                                    activeCellStatusList [siblingCellPosition1*12+7] = 0;

                                                                    activeCellStatusList [siblingCellPosition1*12+8] = 0;

                                                                    fusionCount++;

                                                                    loopCheck = 1;

                                                                }

**else**{

                                                                    activeCellStatusList [siblingCellPosition1*12+5] = 1;

                                                                    activeCellStatusList [siblingCellPosition2*12+6] = siblingCellNo1;

                                                                    activeCellStatusList [counter1*12+6] = siblingCellNo1;

                                                                    activeCellStatusList [siblingCellPosition1*12+7] = 0;

                                                                    activeCellStatusList [siblingCellPosition1*12+8] = 0;

                                                                    fusionCount++;

                                                                    loopCheck = 1;

                                                                }

                                                            }

**if** (loopCheck == 0){

                                                                activeCellStatusList [siblingCellPosition1*12+5] = 1;

                                                                activeCellStatusList [siblingCellPosition2*12+6] = siblingCellNo1;

                                                                activeCellStatusList [counter1*12+6] = siblingCellNo1;

                                                                activeCellStatusList [siblingCellPosition1*12+7] = 0;

                                                                activeCellStatusList [siblingCellPosition1*12+8] = 0;

                                                                fusionCount++;

                                                            }

                                                        }

**else** **if** (selectChange == 15){

                                                            loopCheck = 0;

**if** (expandDoublingDoubCFCount != 0){

                                                                randInit = rand() % expandDoublingDoubCFCount/2 + 0;

**if** (expandDoublingDoubCF [randInit*2] > siblinFusionDoub1){

**if** (activeCellStatusList [siblingCellPosition1*12+5] != 2) activeCellStatusList [siblingCellPosition1*12+7]++;

                                                                    activeCellStatusList [siblingCellPosition1*12+4] = expandDoublingDoubCF [randInit*2];

                                                                    activeCellStatusList [siblingCellPosition1*12+5] = 2;

                                                                    activeCellStatusList [siblingCellPosition2*12+6] = siblingCellNo1;

                                                                    activeCellStatusList [counter1*12+6] = siblingCellNo1;

                                                                    activeCellStatusList [siblingCellPosition1*12+8] = (**int**)simProcessDataBaseHold [18];

                                                                    fusionCount++;

                                                                    loopCheck = 1;

                                                                }

**else**{

**if** (activeCellStatusList [siblingCellPosition1*12+5] != 2) activeCellStatusList [siblingCellPosition1*12+7]++;

                                                                    activeCellStatusList [siblingCellPosition1*12+5] = 2;

                                                                    activeCellStatusList [siblingCellPosition2*12+6] = siblingCellNo1;

                                                                    activeCellStatusList [counter1*12+6] = siblingCellNo1;

                                                                    activeCellStatusList [siblingCellPosition1*12+8] = (**int**)simProcessDataBaseHold [18];

                                                                    fusionCount++;

                                                                    loopCheck = 1;

                                                                }

                                                            }

**if** (loopCheck == 0){

**if** (activeCellStatusList [siblingCellPosition1*12+5] != 2) activeCellStatusList [siblingCellPosition1*12+7]++;

                                                                activeCellStatusList [siblingCellPosition1*12+5] = 2;

                                                                activeCellStatusList [siblingCellPosition2*12+6] = siblingCellNo1;

                                                                activeCellStatusList [counter1*12+6] = siblingCellNo1;

                                                                activeCellStatusList [siblingCellPosition1*12+8] = (**int**)simProcessDataBaseHold [18];

                                                                fusionCount++;

                                                            }

                                                        }

**else** **if** (selectChange == 16){

**if** (expandTDCFCDCount != 0){

                                                                randInit = rand() % expandTDCFCDCount/2 + 0;

**if** (expandTDCFCD [randInit*2] > siblinFusionDoub1){

                                                                    activeCellStatusList [siblingCellPosition1*12+4] = expandTDCFCD [randInit*2];

                                                                    activeCellStatusList [siblingCellPosition1*12+5] = 3;

                                                                    activeCellStatusList [siblingCellPosition2*12+6] = siblingCellNo1;

                                                                    activeCellStatusList [counter1*12+6] = siblingCellNo1;

                                                                    fusionCount++;

                                                                }

**else**{

                                                                    activeCellStatusList [siblingCellPosition1*12+5] = 3;

                                                                    activeCellStatusList [siblingCellPosition2*12+6] = siblingCellNo1;

                                                                    activeCellStatusList [counter1*12+6] = siblingCellNo1;

                                                                    fusionCount++;

                                                                }

                                                            }

**else**{

                                                                activeCellStatusList [siblingCellPosition1*12+5] = 3;

                                                                activeCellStatusList [siblingCellPosition2*12+6] = siblingCellNo1;

                                                                activeCellStatusList [counter1*12+6] = siblingCellNo1;

                                                                fusionCount++;

                                                            }

                                                        }

                                                    }

                                                }

**else** **if** (siblinFusionCheck1 != 6 && siblinFusionCheck2 != 6){

                                                    randInit = rand() % 1 + 0;

**if** (randInit == 1){

                                                        siblingCellPositionSelect = siblingCellPosition1;

                                                        siblingCellNoSelect = siblingCellNo1;

                                                        siblingCellDoubSelect = siblinFusionDoub1;

                                                        fusionCount++;

                                                    }

**else**{

                                                        siblingCellPositionSelect = siblingCellPosition2;

                                                        siblingCellNoSelect = siblingCellNo2;

                                                        siblingCellDoubSelect = siblinFusionDoub2;

                                                        fusionCount++;

                                                    }

                                                    randInit = rand() % 100 + 0;

**if** (secondEventTDCFList [randInit] == 0){

                                                        activeCellStatusList [counter1*12+6] = siblingCellNoSelect;

                                                        fusionCount++;

                                                    }

**else**{

**if** ((activeCellStatusList [counter1*12+8] == 2 || activeCellStatusList [counter1*12+8] == 3) && activeCellStatusList [counter1*12+8] == activeCellStatusList [counter1*12+7] && secondEventTDCFList [randInit] == 15){

                                                            selectChange = 16;

                                                        }

**else** selectChange = secondEventTDCFList [randInit];

**if** (selectChange == 14){

                                                            loopCheck = 0;

**if** (expandDoublingDoubCFCount != 0){

                                                                randInit = rand() % expandDoublingDoubCFCount/2 + 0;

**if** (expandDoublingDoubCF [randInit*2] > siblingCellDoubSelect){

                                                                    activeCellStatusList [siblingCellPositionSelect*12+4] = expandDoublingDoubCF [randInit*2];

                                                                    activeCellStatusList [siblingCellPositionSelect*12+5] = 1;

                                                                    activeCellStatusList [counter1*12+6] = siblingCellNoSelect;

                                                                    activeCellStatusList [siblingCellPositionSelect*12+7] = 0;

                                                                    activeCellStatusList [siblingCellPositionSelect*12+8] = 0;

                                                                    fusionCount++;

                                                                    loopCheck = 1;

                                                                }

**else**{

                                                                    activeCellStatusList [siblingCellPositionSelect*12+5] = 1;

                                                                    activeCellStatusList [counter1*12+6] = siblingCellNoSelect;

                                                                    activeCellStatusList [siblingCellPositionSelect*12+7] = 0;

                                                                    activeCellStatusList [siblingCellPositionSelect*12+8] = 0;

                                                                    fusionCount++;

                                                                    loopCheck = 1;

                                                                }

                                                            }

**if** (loopCheck == 0){

                                                                activeCellStatusList [siblingCellPositionSelect*12+5] = 1;

                                                                activeCellStatusList [counter1*12+6] = siblingCellNoSelect;

                                                                activeCellStatusList [siblingCellPositionSelect*12+7] = 0;

                                                                activeCellStatusList [siblingCellPositionSelect*12+8] = 0;

                                                                fusionCount++;

                                                            }

                                                        }

**else** **if** (selectChange == 15){

                                                            loopCheck = 0;

**if** (expandDoublingDoubCFCount != 0){

                                                                randInit = rand() % expandDoublingDoubCFCount/2 + 0;

**if** (expandDoublingDoubCF [randInit*2] > siblingCellDoubSelect){

**if** (activeCellStatusList [siblingCellPositionSelect*12+5] != 2) activeCellStatusList [siblingCellPositionSelect*12+7]++;

                                                                    activeCellStatusList [siblingCellPositionSelect*12+4] = expandDoublingDoubCF [randInit*2];

                                                                    activeCellStatusList [siblingCellPositionSelect*12+5] = 2;

                                                                    activeCellStatusList [counter1*12+6] = siblingCellNoSelect;

                                                                    activeCellStatusList [siblingCellPositionSelect*12+8] = (**int**)simProcessDataBaseHold [18];

                                                                    fusionCount++;

                                                                    loopCheck = 1;

                                                                }

**else**{

**if** (activeCellStatusList [siblingCellPositionSelect*12+5] != 2) activeCellStatusList [siblingCellPositionSelect*12+7]++;

                                                                    activeCellStatusList [siblingCellPosition1*12+5] = 2;

                                                                    activeCellStatusList [counter1*12+6] = siblingCellNoSelect;

                                                                    activeCellStatusList [siblingCellPositionSelect*12+8] = (**int**)simProcessDataBaseHold [18];

                                                                    fusionCount++;

                                                                    loopCheck = 1;

                                                                }

                                                            }

**if** (loopCheck == 0){

**if** (activeCellStatusList [siblingCellPositionSelect*12+5] != 2) activeCellStatusList [siblingCellPositionSelect*12+7]++;

                                                                activeCellStatusList [siblingCellPositionSelect*12+5] = 2;

                                                                activeCellStatusList [counter1*12+6] = siblingCellNoSelect;

                                                                activeCellStatusList [siblingCellPositionSelect*12+8] = (**int**)simProcessDataBaseHold [18];

                                                                fusionCount++;

                                                            }

                                                        }

**else** **if** (selectChange == 16){

**if** (expandTDCFCDCount != 0){

                                                                randInit = rand() % expandTDCFCDCount/2 + 0;

**if** (expandTDCFCD [randInit*2] > siblingCellDoubSelect){

                                                                    activeCellStatusList [siblingCellPositionSelect*12+4] = expandTDCFCD [randInit*2];

                                                                    activeCellStatusList [siblingCellPositionSelect*12+5] = 3;

                                                                    activeCellStatusList [counter1*12+6] = siblingCellNoSelect;

                                                                    fusionCount++;

                                                                }

**else**{

                                                                    activeCellStatusList [siblingCellPositionSelect*12+5] = 3;

                                                                    activeCellStatusList [counter1*12+6] = siblingCellNoSelect;

                                                                    fusionCount++;

                                                                }

                                                            }

**else**{

                                                                activeCellStatusList [siblingCellPositionSelect*12+5] = 3;

                                                                activeCellStatusList [counter1*12+6] = siblingCellNoSelect;

                                                                fusionCount++;

                                                            }

                                                        }

                                                    }

                                                }

                                            }

                                        }

                                    }

**else** **if** (activeCellStatusList [counter1*12+5] == 6){ //====The following process is the same as above, but the arrays used here are for mixed cells=====

**if** (activeCellStatusList [counter1*12+2] == 0){

                                            siblinFusionDoub1 = 0;

                                            siblingCellNo1 = 0;

**for** (**int** counter2 = 0; counter2 < activeCellStatusListCount/12; counter2++){

**if** (activeCellStatusList [counter2*12+1] == activeCellStatusList [counter1*12] && activeCellStatusList [counter2*12+3] == activeCellStatusList [counter1*12+3]){

                                                    siblingCellNo1 = activeCellStatusList [counter2*12];

                                                    siblinFusionDoub1 = activeCellStatusList [counter2*12+4];

                                                    siblingCellPosition1 = counter2;

                                                    fusionCount++;

**break**;

                                                }

                                            }

                                            randInit = rand() % 100 + 0;

**if** (secondEventBDCFListSel [randInit] == 0){

                                                activeCellStatusList [counter1*12+6] = siblingCellNo1;

                                                fusionCount++;

                                            }

**else** **if** (secondEventBDCFListSel [randInit] == 7){

                                                loopCheck = 0;

**if** (expandDoublingDoubCFSelCount != 0){

                                                    randInit = rand() % expandDoublingDoubCFSelCount/2 + 0;

**if** (expandDoublingDoubCFSel [randInit*2] > siblinFusionDoub1){

                                                        activeCellStatusList [siblingCellPosition1*12+4] = expandDoublingDoubCFSel [randInit*2];

                                                        activeCellStatusList [siblingCellPosition1*12+5] = 1;

                                                        activeCellStatusList [counter1*12+6] = siblingCellNo1;

                                                        activeCellStatusList [siblingCellPosition1*12+7] = 0;

                                                        activeCellStatusList [siblingCellPosition1*12+8] = 0;

                                                        fusionCount++;

                                                        loopCheck = 1;

                                                    }

**else**{

                                                        activeCellStatusList [siblingCellPosition1*12+5] = 1;

                                                        activeCellStatusList [counter1*12+6] = siblingCellNo1;

                                                        activeCellStatusList [siblingCellPosition1*12+7] = 0;

                                                        activeCellStatusList [siblingCellPosition1*12+8] = 0;

                                                        fusionCount++;

                                                        loopCheck = 1;

                                                    }

                                                }

**if** (loopCheck == 0){

                                                    activeCellStatusList [siblingCellPosition1*12+5] = 1;

                                                    activeCellStatusList [counter1*12+6] = siblingCellNo1;

                                                    activeCellStatusList [siblingCellPosition1*12+7] = 0;

                                                    activeCellStatusList [siblingCellPosition1*12+8] = 0;

                                                    fusionCount++;

                                                }

                                            }

**else** **if** (secondEventBDCFListSel [randInit] == 8){

                                                loopCheck = 0;

**if** (expandDoublingDoubCFSelCount != 0){

                                                    randInit = rand() % expandDoublingDoubCFSelCount/2 + 0;

**if** (expandDoublingDoubCFSel [randInit*2] > siblinFusionDoub1){

**if** (activeCellStatusList [siblingCellPosition1*12+5] != 2) activeCellStatusList [siblingCellPosition1*12+7]++;

                                                        activeCellStatusList [siblingCellPosition1*12+4] = expandDoublingDoubCFSel [randInit*2];

                                                        activeCellStatusList [siblingCellPosition1*12+5] = 2;

                                                        activeCellStatusList [counter1*12+6] = siblingCellNo1;

                                                        activeCellStatusList [siblingCellPosition1*12+8] = (**int**)simProcessDataProgHold [18];

                                                        fusionCount++;

                                                        loopCheck = 1;

                                                    }

**else**{

**if** (activeCellStatusList [siblingCellPosition1*12+5] != 2) activeCellStatusList [siblingCellPosition1*12+7]++;

                                                        activeCellStatusList [siblingCellPosition1*12+5] = 2;

                                                        activeCellStatusList [counter1*12+6] = siblingCellNo1;

                                                        activeCellStatusList [siblingCellPosition1*12+8] = (**int**)simProcessDataProgHold [18];

                                                        fusionCount++;

                                                        loopCheck = 1;

                                                    }

                                                }

**if** (loopCheck == 0){

**if** (activeCellStatusList [siblingCellPosition1*12+5] != 2) activeCellStatusList [siblingCellPosition1*12+7]++;

                                                    activeCellStatusList [siblingCellPosition1*12+5] = 2;

                                                    activeCellStatusList [counter1*12+6] = siblingCellNo1;

                                                    activeCellStatusList [siblingCellPosition1*12+8] = (**int**)simProcessDataProgHold [18];

                                                    fusionCount++;

                                                }

                                            }

**else** **if** (secondEventBDCFListSel [randInit] == 9){

**if** (expandBDCFCDSelCount != 0){

                                                    randInit = rand() % expandBDCFCDSelCount/2 + 0;

**if** (expandBDCFCDSel [randInit*2] > siblinFusionDoub1){

                                                        activeCellStatusList [siblingCellPosition1*12+4] = expandBDCFCDSel [randInit*2];

                                                        activeCellStatusList [siblingCellPosition1*12+5] = 3;

                                                        activeCellStatusList [counter1*12+6] = siblingCellNo1;

                                                        fusionCount++;

                                                    }

**else**{

                                                        activeCellStatusList [siblingCellPosition1*12+5] = 3;

                                                        activeCellStatusList [counter1*12+6] = siblingCellNo1;

                                                        fusionCount++;

                                                    }

                                                }

**else**{

                                                    activeCellStatusList [siblingCellPosition1*12+5] = 3;

                                                    activeCellStatusList [counter1*12+6] = siblingCellNo1;

                                                    fusionCount++;

                                                }

                                            }

                                        }

**else**{

                                            siblinFusionCheck1 = 0;

                                            siblinFusionCheck2 = 0;

                                            siblinFusionDoub1 = 0;

                                            siblinFusionDoub2 = 0;

                                            siblingCellNo1 = 0;

                                            siblingCellNo2 = 0;

**for** (**int** counter2 = 0; counter2 < activeCellStatusListCount/12; counter2++){

**if** ((activeCellStatusList [counter2*12+1] == activeCellStatusList [counter1*12] || activeCellStatusList [counter2*12+2] == activeCellStatusList [counter1*12]) && activeCellStatusList [counter2*12+3] == activeCellStatusList [counter1*12+3]){

**if** (siblinFusionCheck1 == 0 && activeCellStatusList [counter2*12+5] != 0){

                                                        siblinFusionCheck1 = activeCellStatusList [counter2*12+5];

                                                        siblinFusionDoub1 = activeCellStatusList [counter2*12+4];

                                                        siblingCellNo1 = activeCellStatusList [counter2*12];

                                                        siblingCellPosition1 = counter2;

                                                    }

**else** **if** (siblinFusionCheck1 != 0 && siblinFusionCheck2 == 0 && activeCellStatusList [counter2*12+5] != 0){

                                                        siblinFusionCheck2 = activeCellStatusList [counter2*12+5];

                                                        siblinFusionDoub2 = activeCellStatusList [counter2*12+4];

                                                        siblingCellNo2 = activeCellStatusList [counter2*12];

                                                        siblingCellPosition2 = counter2;

**break**;

                                                    }

                                                }

                                            }

**if** (siblinFusionCheck1 == 6 && siblinFusionCheck2 != 6){

                                                randInit = rand() % 100 + 0;

**if** (secondEventTDCFListSel [randInit] == 0){

                                                    activeCellStatusList [siblingCellPosition1*12+6] = siblingCellNo2;

                                                    activeCellStatusList [counter1*12+6] = siblingCellNo2;

                                                    fusionCount++;

                                                }

**else**{

**if** ((activeCellStatusList [counter1*12+8] == 2 || activeCellStatusList [counter1*12+8] == 3) && activeCellStatusList [counter1*12+8] == activeCellStatusList [counter1*12+7] && secondEventTDCFListSel [randInit] == 15){

                                                        selectChange = 16;

                                                    }

**else** selectChange = secondEventTDCFListSel [randInit];

**if** (selectChange == 14){

                                                        loopCheck = 0;

**if** (expandDoublingDoubCFSelCount != 0){

                                                            randInit = rand() % expandDoublingDoubCFSelCount/2 + 0;

**if** (expandDoublingDoubCFSel [randInit*2] > siblinFusionDoub2){

                                                                activeCellStatusList [siblingCellPosition2*12+4] = expandDoublingDoubCFSel [randInit*2];

                                                                activeCellStatusList [siblingCellPosition2*12+5] = 1;

                                                                activeCellStatusList [siblingCellPosition1*12+6] = siblingCellNo2;

                                                                activeCellStatusList [counter1*12+6] = siblingCellNo2;

                                                                activeCellStatusList [siblingCellPosition2*12+7] = 0;

                                                                activeCellStatusList [siblingCellPosition2*12+8] = 0;

                                                                fusionCount++;

                                                                loopCheck = 1;

                                                            }

**else**{

                                                                activeCellStatusList [siblingCellPosition2*12+5] = 1;

                                                                activeCellStatusList [siblingCellPosition1*12+6] = siblingCellNo2;

                                                                activeCellStatusList [counter1*12+6] = siblingCellNo2;

                                                                activeCellStatusList [siblingCellPosition2*12+7] = 0;

                                                                activeCellStatusList [siblingCellPosition2*12+8] = 0;

                                                                fusionCount++;

                                                                loopCheck = 1;

                                                            }

                                                        }

**if** (loopCheck == 0){

                                                            activeCellStatusList [siblingCellPosition2*12+5] = 1;

                                                            activeCellStatusList [siblingCellPosition1*12+6] = siblingCellNo2;

                                                            activeCellStatusList [counter1*12+6] = siblingCellNo2;

                                                            activeCellStatusList [siblingCellPosition2*12+7] = 0;

                                                            activeCellStatusList [siblingCellPosition2*12+8] = 0;

                                                            fusionCount++;

                                                        }

                                                    }

**else** **if** (selectChange == 15){

                                                        loopCheck = 0;

**if** (expandDoublingDoubCFSelCount != 0){

                                                            randInit = rand() % expandDoublingDoubCFSelCount/2 + 0;

**if** (expandDoublingDoubCFSel [randInit*2] > siblinFusionDoub2){

**if** (activeCellStatusList [siblingCellPosition2*12+5] != 2) activeCellStatusList [siblingCellPosition2*12+7]++;

                                                                activeCellStatusList [siblingCellPosition2*12+4] = expandDoublingDoubCFSel [randInit*2];

                                                                activeCellStatusList [siblingCellPosition2*12+5] = 2;

                                                                activeCellStatusList [siblingCellPosition1*12+6] = siblingCellNo2;

                                                                activeCellStatusList [counter1*12+6] = siblingCellNo2;

                                                                activeCellStatusList [siblingCellPosition2*12+8] = (**int**)simProcessDataProgHold [18];

                                                                fusionCount++;

                                                                loopCheck = 1;

                                                            }

**else**{

**if** (activeCellStatusList [siblingCellPosition2*12+5] != 2) activeCellStatusList [siblingCellPosition2*12+7]++;

                                                                activeCellStatusList [siblingCellPosition2*12+5] = 2;

                                                                activeCellStatusList [siblingCellPosition1*12+6] = siblingCellNo2;

                                                                activeCellStatusList [counter1*12+6] = siblingCellNo2;

                                                                activeCellStatusList [siblingCellPosition2*12+8] = (**int**)simProcessDataProgHold [18];

                                                                fusionCount++;

                                                                loopCheck = 1;

                                                            }

                                                        }

**if** (loopCheck == 0){

**if** (activeCellStatusList [siblingCellPosition2*12+5] != 2) activeCellStatusList [siblingCellPosition2*12+7]++;

                                                            activeCellStatusList [siblingCellPosition2*12+5] = 2;

                                                            activeCellStatusList [siblingCellPosition1*12+6] = siblingCellNo2;

                                                            activeCellStatusList [counter1*12+6] = siblingCellNo2;

                                                            activeCellStatusList [siblingCellPosition2*12+8] = (**int**)simProcessDataProgHold [18];

                                                            fusionCount++;

                                                        }

                                                    }

**else** **if** (selectChange == 16){

**if** (expandTDCFCDSelCount != 0){

                                                            randInit = rand() % expandTDCFCDSelCount/2 + 0;

**if** (expandTDCFCDSel [randInit*2] > siblinFusionDoub2){

                                                                activeCellStatusList [siblingCellPosition2*12+4] = expandTDCFCDSel [randInit*2];

                                                                activeCellStatusList [siblingCellPosition2*12+5] = 3;

                                                                activeCellStatusList [siblingCellPosition1*12+6] = siblingCellNo2;

                                                                activeCellStatusList [counter1*12+6] = siblingCellNo2;

                                                                fusionCount++;

                                                            }

**else**{

                                                                activeCellStatusList [siblingCellPosition2*12+5] = 3;

                                                                activeCellStatusList [siblingCellPosition1*12+6] = siblingCellNo2;

                                                                activeCellStatusList [counter1*12+6] = siblingCellNo2;

                                                                fusionCount++;

                                                            }

                                                        }

**else**{

                                                            activeCellStatusList [siblingCellPosition2*12+5] = 3;

                                                            activeCellStatusList [siblingCellPosition1*12+6] = siblingCellNo2;

                                                            activeCellStatusList [counter1*12+6] = siblingCellNo2;

                                                            fusionCount++;

                                                        }

                                                    }

                                                }

                                            }

**else** **if** (siblinFusionCheck1 != 6 && siblinFusionCheck2 == 6){

                                                randInit = rand() % 100 + 0;

**if** (secondEventTDCFListSel [randInit] == 0){

                                                    activeCellStatusList [siblingCellPosition2*12+6] = siblingCellNo1;

                                                    activeCellStatusList [counter1*12+6] = siblingCellNo1;

                                                    fusionCount++;

                                                }

**else**{

**if** ((activeCellStatusList [counter1*12+8] == 2 || activeCellStatusList [counter1*12+8] == 3) && activeCellStatusList [counter1*12+8] == activeCellStatusList [counter1*12+7] && secondEventTDCFListSel [randInit] == 15){

                                                        selectChange = 16;

                                                    }

**else** selectChange = secondEventTDCFListSel [randInit];

**if** (selectChange == 14){

                                                        loopCheck = 0;

**if** (expandDoublingDoubCFSelCount != 0){

                                                            randInit = rand() % expandDoublingDoubCFSelCount/2 + 0;

**if** (expandDoublingDoubCFSel [randInit*2] > siblinFusionDoub1){

                                                                activeCellStatusList [siblingCellPosition1*12+4] = expandDoublingDoubCFSel [randInit*2];

                                                                activeCellStatusList [siblingCellPosition1*12+5] = 1;

                                                                activeCellStatusList [siblingCellPosition2*12+6] = siblingCellNo1;

                                                                activeCellStatusList [counter1*12+6] = siblingCellNo1;

                                                                activeCellStatusList [siblingCellPosition1*12+7] = 0;

                                                                activeCellStatusList [siblingCellPosition1*12+8] = 0;

                                                                fusionCount++;

                                                                loopCheck = 1;

                                                            }

**else**{

                                                                activeCellStatusList [siblingCellPosition1*12+5] = 1;

                                                                activeCellStatusList [siblingCellPosition2*12+6] = siblingCellNo1;

                                                                activeCellStatusList [counter1*12+6] = siblingCellNo1;

                                                                activeCellStatusList [siblingCellPosition1*12+7] = 0;

                                                                activeCellStatusList [siblingCellPosition1*12+8] = 0;

                                                                fusionCount++;

                                                                loopCheck = 1;

                                                            }

                                                        }

**if** (loopCheck == 0){

                                                            activeCellStatusList [siblingCellPosition1*12+5] = 1;

                                                            activeCellStatusList [siblingCellPosition2*12+6] = siblingCellNo1;

                                                            activeCellStatusList [counter1*12+6] = siblingCellNo1;

                                                            activeCellStatusList [siblingCellPosition1*12+7] = 0;

                                                            activeCellStatusList [siblingCellPosition1*12+8] = 0;

                                                            fusionCount++;

                                                        }

                                                    }

**else** **if** (selectChange == 15){

                                                        loopCheck = 0;

**if** (expandDoublingDoubCFSelCount != 0){

                                                            randInit = rand() % expandDoublingDoubCFSelCount/2 + 0;

**if** (expandDoublingDoubCFSel [randInit*2] > siblinFusionDoub1){

**if** (activeCellStatusList [siblingCellPosition1*12+5] != 2) activeCellStatusList [siblingCellPosition1*12+7]++;

                                                                activeCellStatusList [siblingCellPosition1*12+4] = expandDoublingDoubCFSel [randInit*2];

                                                                activeCellStatusList [siblingCellPosition1*12+5] = 2;

                                                                activeCellStatusList [siblingCellPosition2*12+6] = siblingCellNo1;

                                                                activeCellStatusList [counter1*12+6] = siblingCellNo1;

                                                                activeCellStatusList [siblingCellPosition1*12+8] = (**int**)simProcessDataProgHold [18];

                                                                fusionCount++;

                                                                loopCheck = 1;

                                                            }

**else**{

**if** (activeCellStatusList [siblingCellPosition1*12+5] != 2) activeCellStatusList [siblingCellPosition1*12+7]++;

                                                                activeCellStatusList [siblingCellPosition1*12+5] = 2;

                                                                activeCellStatusList [siblingCellPosition2*12+6] = siblingCellNo1;

                                                                activeCellStatusList [counter1*12+6] = siblingCellNo1;

                                                                activeCellStatusList [siblingCellPosition1*12+8] = (**int**)simProcessDataProgHold [18];

                                                                fusionCount++;

                                                                loopCheck = 1;

                                                            }

                                                        }

**if** (loopCheck == 0){

**if** (activeCellStatusList [siblingCellPosition1*12+5] != 2) activeCellStatusList [siblingCellPosition1*12+7]++;

                                                            activeCellStatusList [siblingCellPosition1*12+5] = 2;

                                                            activeCellStatusList [siblingCellPosition2*12+6] = siblingCellNo1;

                                                            activeCellStatusList [counter1*12+6] = siblingCellNo1;

                                                            activeCellStatusList [siblingCellPosition1*12+8] = (**int**)simProcessDataProgHold [18];

                                                            fusionCount++;

                                                        }

                                                    }

**else** **if** (selectChange == 16){

**if** (expandTDCFCDSelCount != 0){

                                                            randInit = rand() % expandTDCFCDSelCount/2 + 0;

**if** (expandTDCFCD [randInit*2] > siblinFusionDoub1){

                                                                activeCellStatusList [siblingCellPosition1*12+4] = expandTDCFCDSel [randInit*2];

                                                                activeCellStatusList [siblingCellPosition1*12+5] = 3;

                                                                activeCellStatusList [siblingCellPosition2*12+6] = siblingCellNo1;

                                                                activeCellStatusList [counter1*12+6] = siblingCellNo1;

                                                                fusionCount++;

                                                            }

**else**{

                                                                activeCellStatusList [siblingCellPosition1*12+5] = 3;

                                                                activeCellStatusList [siblingCellPosition2*12+6] = siblingCellNo1;

                                                                activeCellStatusList [counter1*12+6] = siblingCellNo1;

                                                                fusionCount++;

                                                            }

                                                        }

**else**{

                                                            activeCellStatusList [siblingCellPosition1*12+5] = 3;

                                                            activeCellStatusList [siblingCellPosition2*12+6] = siblingCellNo1;

                                                            activeCellStatusList [counter1*12+6] = siblingCellNo1;

                                                            fusionCount++;

                                                        }

                                                    }

                                                }

                                            }

**else** **if** (siblinFusionCheck1 != 6 && siblinFusionCheck2 != 6){

                                                randInit = rand() % 1 + 0;

**if** (randInit == 1){

                                                    siblingCellPositionSelect = siblingCellPosition1;

                                                    siblingCellNoSelect = siblingCellNo1;

                                                    siblingCellDoubSelect = siblinFusionDoub1;

                                                    fusionCount++;

                                                }

**else**{

                                                    siblingCellPositionSelect = siblingCellPosition2;

                                                    siblingCellNoSelect = siblingCellNo2;

                                                    siblingCellDoubSelect = siblinFusionDoub2;

                                                    fusionCount++;

                                                }

                                                randInit = rand() % 100 + 0;

**if** (secondEventTDCFListSel [randInit] == 0){

                                                    activeCellStatusList [counter1*12+6] = siblingCellNoSelect;

                                                    fusionCount++;

                                                }

**else**{

**if** ((activeCellStatusList [counter1*12+8] == 2 || activeCellStatusList [counter1*12+8] == 3) && activeCellStatusList [counter1*12+8] == activeCellStatusList [counter1*12+7] && secondEventTDCFListSel [randInit] == 15){

                                                        selectChange = 16;

                                                    }

**else** selectChange = secondEventTDCFListSel [randInit];

**if** (selectChange == 14){

                                                        loopCheck = 0;

**if** (expandDoublingDoubCFSelCount != 0){

                                                            randInit = rand() % expandDoublingDoubCFSelCount/2 + 0;

**if** (expandDoublingDoubCFSel [randInit*2] > siblingCellDoubSelect){

                                                                activeCellStatusList [siblingCellPositionSelect*12+4] = expandDoublingDoubCFSel [randInit*2];

                                                                activeCellStatusList [siblingCellPositionSelect*12+5] = 1;

                                                                activeCellStatusList [counter1*12+6] = siblingCellNoSelect;

                                                                activeCellStatusList [siblingCellPositionSelect*12+7] = 0;

                                                                activeCellStatusList [siblingCellPositionSelect*12+8] = 0;

                                                                fusionCount++;

                                                                loopCheck = 1;

                                                            }

**else**{

                                                                activeCellStatusList [siblingCellPositionSelect*12+5] = 1;

                                                                activeCellStatusList [counter1*12+6] = siblingCellNoSelect;

                                                                activeCellStatusList [siblingCellPositionSelect*12+7] = 0;

                                                                activeCellStatusList [siblingCellPositionSelect*12+8] = 0;

                                                                fusionCount++;

                                                                loopCheck = 1;

                                                            }

                                                        }

**if** (loopCheck == 0){

                                                            activeCellStatusList [siblingCellPositionSelect*12+5] = 1;

                                                            activeCellStatusList [counter1*12+6] = siblingCellNoSelect;

                                                            activeCellStatusList [siblingCellPositionSelect*12+7] = 0;

                                                            activeCellStatusList [siblingCellPositionSelect*12+8] = 0;

                                                            fusionCount++;

                                                        }

                                                    }

**else** **if** (selectChange == 15){

                                                        loopCheck = 0;

**if** (expandDoublingDoubCFSelCount != 0){

                                                            randInit = rand() % expandDoublingDoubCFSelCount/2 + 0;

**if** (expandDoublingDoubCFSel [randInit*2] > siblingCellDoubSelect){

**if** (activeCellStatusList [siblingCellPositionSelect*12+5] != 2) activeCellStatusList [siblingCellPositionSelect*12+7]++;

                                                                activeCellStatusList [siblingCellPositionSelect*12+4] = expandDoublingDoubCFSel [randInit*2];

                                                                activeCellStatusList [siblingCellPositionSelect*12+5] = 2;

                                                                activeCellStatusList [counter1*12+6] = siblingCellNoSelect;

                                                                activeCellStatusList [siblingCellPositionSelect*12+8] = (**int**)simProcessDataProgHold [18];

                                                                fusionCount++;

                                                                loopCheck = 1;

                                                            }

**else**{

**if** (activeCellStatusList [siblingCellPositionSelect*12+5] != 2) activeCellStatusList [siblingCellPositionSelect*12+7]++;

                                                                activeCellStatusList [siblingCellPosition1*12+5] = 2;

                                                                activeCellStatusList [counter1*12+6] = siblingCellNoSelect;

                                                                activeCellStatusList [siblingCellPositionSelect*12+8] = (**int**)simProcessDataProgHold [18];

                                                                fusionCount++;

                                                                loopCheck = 1;

                                                            }

                                                        }

**if** (loopCheck == 0){

**if** (activeCellStatusList [siblingCellPositionSelect*12+5] != 2) activeCellStatusList [siblingCellPositionSelect*12+7]++;

                                                            activeCellStatusList [siblingCellPositionSelect*12+5] = 2;

                                                            activeCellStatusList [counter1*12+6] = siblingCellNoSelect;

                                                            activeCellStatusList [siblingCellPositionSelect*12+8] = (**int**)simProcessDataProgHold [18];

                                                            fusionCount++;

                                                        }

                                                    }

**else** **if** (selectChange == 16){

**if** (expandTDCFCDSelCount != 0){

                                                            randInit = rand() % expandTDCFCDSelCount/2 + 0;

**if** (expandTDCFCDSel [randInit*2] > siblingCellDoubSelect){

                                                                activeCellStatusList [siblingCellPositionSelect*12+4] = expandTDCFCDSel [randInit*2];

                                                                activeCellStatusList [siblingCellPositionSelect*12+5] = 3;

                                                                activeCellStatusList [counter1*12+6] = siblingCellNoSelect;

                                                                fusionCount++;

                                                            }

**else**{

                                                                activeCellStatusList [siblingCellPositionSelect*12+5] = 3;

                                                                activeCellStatusList [counter1*12+6] = siblingCellNoSelect;

                                                                fusionCount++;

                                                            }

                                                        }

**else**{

                                                            activeCellStatusList [siblingCellPositionSelect*12+5] = 3;

                                                            activeCellStatusList [counter1*12+6] = siblingCellNoSelect;

                                                            fusionCount++;

                                                        }

                                                    }

                                                }

                                            }

                                        }
[truncated: 205,013 more chars]
