# Supplementary material for: Empirical single-cell tracking and cell-fate simulation reveal dual roles of p53 in tumor suppression
Source: eLife. 2022 Sep 20;11:e72498. doi: 10.7554/eLife.72498 (PMC9560164; doi:10.7554/eLife.72498)
Supplement: Figure 7—source code 4. [file elife-72498-fig7-code4.docx]

//

//  SimulationPerformMid.m

//  Lineage_Analysis

//

//  Created by Masahiko Sato

//

/*

 Simulation: the second condition

 The following code is written in Objective-C/C++ using Xcode.

 Comments: see SimulationPerform

 */

-(**void**)midPerform:(**int**)growthCycleMid :(**int**)growthCycleProgEnd :(**int**)growthCycleProgEnd2{

**int** growthCycleBase = growthCycleMid;

    selArraySet = 0;

**int** totalNoOfEntryFirstDV = 0;

**int** totalNoOfEntryDoubBD = 0;

**int** totalNoOfEntryDoubTD = 0;

**int** totalNoOfEntryDoubCF = 0;

**int** totalNoOfNonDivCD = 0;

**int** totalNoOfBDCD = 0;

**int** totalNoOfBDCF = 0;

**int** totalNoOfBDCFCD = 0;

**int** totalNoOfTDCF = 0;

**int** totalNoOfTDCFCD = 0;

**int** totalNoOfTDCD = 0;

**for** (**int** counter1 = 0; counter1 < simulationDistributionMidDataCount/11; counter1++){

**if** (simulationDistributionMidData [counter1*11] != 0) totalNoOfBDCD = totalNoOfBDCD+simulationDistributionMidData [counter1*11];

**if** (simulationDistributionMidData [counter1*11+1] != 0) totalNoOfNonDivCD = totalNoOfNonDivCD+simulationDistributionMidData [counter1*11+1];

**if** (simulationDistributionMidData [counter1*11+2] != 0) totalNoOfBDCF = totalNoOfBDCF+simulationDistributionMidData [counter1*11+2];

**if** (simulationDistributionMidData [counter1*11+3] != 0) totalNoOfBDCFCD = totalNoOfBDCFCD+simulationDistributionMidData [counter1*11+3];

**if** (simulationDistributionMidData [counter1*11+4] != 0) totalNoOfTDCF = totalNoOfTDCF+simulationDistributionMidData [counter1*11+4];

**if** (simulationDistributionMidData [counter1*11+5] != 0) totalNoOfTDCFCD = totalNoOfTDCFCD+simulationDistributionMidData [counter1*11+5];

**if** (simulationDistributionMidData [counter1*11+6] != 0) totalNoOfTDCD = totalNoOfTDCD+simulationDistributionMidData [counter1*11+6];

**if** (simulationDistributionMidData [counter1*11+7] != 0) totalNoOfEntryDoubBD = totalNoOfEntryDoubBD+simulationDistributionMidData [counter1*11+7];

**if** (simulationDistributionMidData [counter1*11+8] != 0) totalNoOfEntryDoubTD = totalNoOfEntryDoubTD+simulationDistributionMidData [counter1*11+8];

**if** (simulationDistributionMidData [counter1*11+9] != 0) totalNoOfEntryDoubCF = totalNoOfEntryDoubCF+simulationDistributionMidData [counter1*11+9];

**if** (simulationDistributionMidData [counter1*11+10] != 0) totalNoOfEntryFirstDV = totalNoOfEntryFirstDV+simulationDistributionMidData [counter1*11+10];

    }

    totalNoOfEntryDoubBD = (**int**)(round(totalNoOfEntryDoubBD*(**double**)simProcessDataMiddleHold [0]));

    totalNoOfEntryDoubTD = (**int**)(round(totalNoOfEntryDoubTD*(**double**)simProcessDataMiddleHold [1]));

    totalNoOfEntryDoubCF = (**int**)(round(totalNoOfEntryDoubCF*(**double**)simProcessDataMiddleHold [2]));

    totalNoOfBDCD = (**int**)(round(totalNoOfBDCD*(**double**)simProcessDataMiddleHold [5]));

    totalNoOfNonDivCD = (**int**)(round(totalNoOfNonDivCD*(**double**)simProcessDataMiddleHold [6]));

    totalNoOfBDCF = (**int**)(round(totalNoOfBDCF*(**double**)simProcessDataMiddleHold [7]));

    totalNoOfBDCFCD = (**int**)(round(totalNoOfBDCFCD*(**double**)simProcessDataMiddleHold [10]));

    totalNoOfTDCF = (**int**)(round(totalNoOfTDCF*(**double**)simProcessDataMiddleHold [11]));

    totalNoOfTDCFCD = (**int**)(round(totalNoOfTDCFCD*(**double**)simProcessDataMiddleHold [14]));

    totalNoOfTDCD = (**int**)(round(totalNoOfTDCD*(**double**)simProcessDataMiddleHold [17]));

**int** *expandFirsDVList = **new** **int** [totalNoOfEntryFirstDV*2+1];

**int** expandFirsDVListCount = 0;

**int** *expandDoublingDoubBD = **new** **int** [totalNoOfEntryDoubBD*2+1];

**int** expandDoublingDoubBDCount = 0;

**int** *expandDoublingDoubTD = **new** **int** [totalNoOfEntryDoubTD*2+1];

**int** expandDoublingDoubTDCount = 0;

**int** *expandDoublingDoubCF = **new** **int** [totalNoOfEntryDoubCF*2+1];

**int** expandDoublingDoubCFCount = 0;

**int** *expandBDCD = **new** **int** [totalNoOfBDCD*2+1];

**int** expandBDCDCount = 0;

**int** *expandBDCF = **new** **int** [totalNoOfBDCF*2+1];

**int** expandBDCFCount = 0;

**int** *expandNonCD = **new** **int** [totalNoOfNonDivCD*2+1];

**int** expandNonCDCount = 0;

**int** *expandBDCFCD = **new** **int** [totalNoOfBDCFCD*2+1];

**int** expandBDCFCDCount = 0;

**int** *expandTDCF = **new** **int** [totalNoOfTDCF*2+1];

**int** expandTDCFCount = 0;

**int** *expandTDCFCD = **new** **int** [totalNoOfTDCFCD*2+1];

**int** expandTDCFCDCount = 0;

**int** *expandTDCD = **new** **int** [totalNoOfTDCD*2+1];

**int** expandTDCDCount = 0;

**int** countTemp = 0;

**for** (**int** counter1 = 0; counter1 < simulationDistributionMidDataCount/11; counter1++){

**if** (simulationDistributionMidData [counter1*11] != 0){

**if** (simProcessDataMiddleHold [5] > 1){

**if** (simulationDistributionMidData [counter1*11] > 1) countTemp = (**int**)(round(simulationDistributionMidData [counter1*11]+(simulationDistributionMidData [counter1*11]-1)*(**double**)simProcessDataMiddleHold [5]));

**else** countTemp = simulationDistributionMidData [counter1*11];

            }

**else** countTemp = (**int**)(round(simulationDistributionMidData [counter1*11]*(**double**)simProcessDataMiddleHold [5]));

**for** (**int** counter2 = 0; counter2 < countTemp; counter2++){

                expandBDCD [expandBDCDCount] = counter1+1, expandBDCDCount++;

                expandBDCD [expandBDCDCount] = 0, expandBDCDCount++;

            }

        }

**if** (simulationDistributionMidData [counter1*11+1] != 0){

**if** (simProcessDataMiddleHold [6] > 1){

**if** (simulationDistributionMidData [counter1*11+1] > 1) countTemp = (**int**)(round(simulationDistributionMidData [counter1*11+1]+(simulationDistributionData [counter1*11+1]-1)*(**double**)simProcessDataMiddleHold [6]));

**else** countTemp = simulationDistributionMidData [counter1*11+1];

            }

**else** countTemp = (**int**)(round(simulationDistributionMidData [counter1*11+1]*(**double**)simProcessDataMiddleHold [6]));

**for** (**int** counter2 = 0; counter2 < countTemp; counter2++){

                expandNonCD [expandNonCDCount] = counter1+1, expandNonCDCount++;

                expandNonCD [expandNonCDCount] = 0, expandNonCDCount++;

            }

        }

**if** (simulationDistributionMidData [counter1*11+2] != 0){

**if** (simProcessDataMiddleHold [7] > 1){

**if** (simulationDistributionMidData [counter1*11+2] > 1) countTemp = (**int**)(round(simulationDistributionMidData [counter1*11+2]+(simulationDistributionMidData [counter1*11+2]-1)*(**double**)simProcessDataMiddleHold [7]));

**else** countTemp = simulationDistributionMidData [counter1*11+2];

            }

**else** countTemp = (**int**)(round(simulationDistributionMidData [counter1*11+2]*(**double**)simProcessDataMiddleHold [7]));

**for** (**int** counter2 = 0; counter2 < countTemp; counter2++){

                expandBDCF [expandBDCFCount] = counter1+1, expandBDCFCount++;

                expandBDCF [expandBDCFCount] = 0, expandBDCFCount++;

            }

        }

**if** (simulationDistributionMidData [counter1*11+3] != 0){

**if** (simProcessDataMiddleHold [10] > 1){

**if** (simulationDistributionMidData [counter1*11+3] > 1) countTemp = (**int**)(round(simulationDistributionMidData [counter1*11+3]+(simulationDistributionMidData [counter1*11+3]-1)*(**double**)simProcessDataMiddleHold [10]));

**else** countTemp = simulationDistributionMidData [counter1*11+3];

            }

**else** countTemp = (**int**)(round(simulationDistributionMidData [counter1*11+3]*(**double**)simProcessDataMiddleHold [10]));

**for** (**int** counter2 = 0; counter2 < countTemp; counter2++){

                expandBDCFCD [expandBDCFCDCount] = counter1+1, expandBDCFCDCount++;

                expandBDCFCD [expandBDCFCDCount] = 0, expandBDCFCDCount++;

            }

        }

**if** (simulationDistributionMidData [counter1*11+4] != 0){

**if** (simProcessDataMiddleHold [11] > 1){

**if** (simulationDistributionMidData [counter1*11+4] > 1) countTemp = (**int**)(round(simulationDistributionMidData [counter1*11+4]+(simulationDistributionMidData [counter1*11+4]-1)*(**double**)simProcessDataMiddleHold [11]));

**else** countTemp = simulationDistributionMidData [counter1*11+4];

            }

**else** countTemp = (**int**)(round(simulationDistributionMidData [counter1*11+4]*(**double**)simProcessDataMiddleHold [11]));

**for** (**int** counter2 = 0; counter2 < countTemp; counter2++){

                expandTDCF [expandTDCFCount] = counter1+1, expandTDCFCount++;

                expandTDCF [expandTDCFCount] = 0, expandTDCFCount++;

            }

        }

**if** (simulationDistributionMidData [counter1*11+5] != 0){

**if** (simProcessDataMiddleHold [14] > 1){

**if** (simulationDistributionMidData [counter1*11+5] > 1) countTemp = (**int**)(round(simulationDistributionMidData [counter1*11+5]+(simulationDistributionMidData [counter1*11+5]-1)*(**double**)simProcessDataMiddleHold [14]));

**else** countTemp = simulationDistributionMidData [counter1*11+5];

            }

**else** countTemp = (**int**)(round(simulationDistributionMidData [counter1*11+5]*(**double**)simProcessDataMiddleHold [14]));

**for** (**int** counter2 = 0; counter2 < countTemp; counter2++){

                expandTDCFCD [expandTDCFCDCount] = counter1+1, expandTDCFCDCount++;

                expandTDCFCD [expandTDCFCDCount] = 0, expandTDCFCDCount++;

            }

        }

**if** (simulationDistributionMidData [counter1*11+6] != 0){

**if** (simProcessDataMiddleHold [17] > 1){

**if** (simulationDistributionMidData [counter1*11+6] > 1) countTemp = (**int**)(round(simulationDistributionMidData [counter1*11+6]+(simulationDistributionMidData [counter1*11+6]-1)*(**double**)simProcessDataMiddleHold [17]));

**else** countTemp = simulationDistributionMidData [counter1*11+6];

            }

**else** countTemp = (**int**)(round(simulationDistributionMidData [counter1*11+6]*(**double**)simProcessDataMiddleHold [17]));

**for** (**int** counter2 = 0; counter2 < countTemp; counter2++){

                expandTDCD [expandTDCDCount] = counter1+1, expandTDCDCount++;

                expandTDCD [expandTDCDCount] = 0, expandTDCDCount++;

            }

        }

**if** (simulationDistributionMidData [counter1*11+7] != 0){

**if** (simProcessDataMiddleHold [0] > 1){

**if** (simulationDistributionMidData [counter1*11+7] > 1) countTemp = (**int**)(round(simulationDistributionMidData [counter1*11+7]+(simulationDistributionMidData [counter1*11+7]-1)*(**double**)simProcessDataMiddleHold [0]));

**else** countTemp = simulationDistributionMidData [counter1*11+7];

            }

**else** countTemp = (**int**)(round(simulationDistributionMidData [counter1*11+7]*(**double**)simProcessDataMiddleHold [0]));

**for** (**int** counter2 = 0; counter2 < countTemp; counter2++){

                expandDoublingDoubBD [expandDoublingDoubBDCount] = counter1+1, expandDoublingDoubBDCount++;

                expandDoublingDoubBD [expandDoublingDoubBDCount] = 0, expandDoublingDoubBDCount++;

            }

        }

**if** (simulationDistributionMidData [counter1*11+8] != 0){

**if** (simProcessDataMiddleHold [1] > 1){

**if** (simulationDistributionMidData [counter1*11+8] > 1) countTemp = (**int**)(round(simulationDistributionMidData [counter1*11+8]+(simulationDistributionMidData [counter1*11+8]-1)*(**double**)simProcessDataMiddleHold [1]));

**else** countTemp = simulationDistributionMidData [counter1*11+8];

            }

**else** countTemp = (**int**)(round(simulationDistributionMidData [counter1*11+8]*(**double**)simProcessDataMiddleHold [1]));

**for** (**int** counter2 = 0; counter2 < countTemp; counter2++){

                expandDoublingDoubTD [expandDoublingDoubTDCount] = counter1+1, expandDoublingDoubTDCount++;

                expandDoublingDoubTD [expandDoublingDoubTDCount] = 0, expandDoublingDoubTDCount++;

            }

        }

**if** (simulationDistributionMidData [counter1*11+9] != 0){

**if** (simProcessDataMiddleHold [2] > 1){

**if** (simulationDistributionMidData [counter1*11+9] > 1) countTemp = (**int**)(round(simulationDistributionMidData [counter1*11+9]+(simulationDistributionMidData [counter1*11+9]-1)*(**double**)simProcessDataMiddleHold [2]));

**else** countTemp = simulationDistributionMidData [counter1*11+9];

            }

**else** countTemp = (**int**)(round(simulationDistributionMidData [counter1*11+9]*(**double**)simProcessDataMiddleHold [2]));

**for** (**int** counter2 = 0; counter2 < countTemp; counter2++){

                expandDoublingDoubCF [expandDoublingDoubCFCount] = counter1+1, expandDoublingDoubCFCount++;

                expandDoublingDoubCF [expandDoublingDoubCFCount] = 0, expandDoublingDoubCFCount++;

            }

        }

**if** (simulationDistributionMidData [counter1*11+10] != 0){

**for** (**int** counter2 = 0; counter2 < simulationDistributionMidData [counter1*11+10]; counter2++){

                expandFirsDVList [expandFirsDVListCount] = counter1+1, expandFirsDVListCount++;

                expandFirsDVList [expandFirsDVListCount] = 0, expandFirsDVListCount++;

            }

        }

    }

**int** *firstEventList = **new** **int** [150];

**int** *secondEventBDList = **new** **int** [150];

**int** *secondEventBDCFList = **new** **int** [150];

**int** *secondEventTDList = **new** **int** [150];

**int** *secondEventTDCFList = **new** **int** [150];

**for** (**int** counter1 = 0; counter1 < 150; counter1++){

        firstEventList [counter1] = 0;

        secondEventBDList [counter1] = 0;

        secondEventBDCFList [counter1] = 0;

        secondEventTDList [counter1] = 0;

        secondEventTDCFList [counter1] = 0;

    }

**int** totalNoOfNonDivCDWithBias = (**int**)(round(totalNoOfNonDivCD*(**double**)simProcessDataMiddleHold [6]));

**int** totalNumberOfnonDivLingCD = (**int**)simProcessDataMiddleHold [20]+totalNoOfNonDivCDWithBias;

**int** remainingLingNo = (**int**)simProcessDataMiddleHold [24]-totalNumberOfnonDivLingCD;

**if** (remainingLingNo < 0) remainingLingNo = 0;

**int** totalNoOfBDTD = (**int**)(simProcessDataMiddleHold [3]+simProcessDataMiddleHold [4]);

**double** nonDivIn100 = totalNumberOfnonDivLingCD/(**double**)(remainingLingNo+totalNumberOfnonDivLingCD);

**double** divIn100 = remainingLingNo/(**double**)(remainingLingNo+totalNumberOfnonDivLingCD);

**int** percentBD = 0;

**int** percentTD = 0;

**int** percentCD = 0;

**int** nonDivPercent = 0;

**double** percentTemp = 0;

**if** (totalNoOfBDTD != 0){

        percentTemp = (simProcessDataMiddleHold [3]/(**double**)totalNoOfBDTD)*100;

        percentBD = (**int**)(round((percentTemp*divIn100)));

        percentTemp = (simProcessDataMiddleHold [4]/(**double**)totalNoOfBDTD)*100;

        percentTD = (**int**)(round((percentTemp*divIn100)));

    }

**if** (totalNumberOfnonDivLingCD != 0){

        percentTemp = (totalNoOfNonDivCDWithBias/(**double**)totalNumberOfnonDivLingCD)*100;

        percentCD = (**int**)(round(percentTemp*nonDivIn100));

        percentTemp = (simProcessDataMiddleHold [20]/(**double**)totalNumberOfnonDivLingCD)*100;

        nonDivPercent = (**int**)(round(percentTemp*nonDivIn100));

    }

**if** (totalNoOfNonDivCD > simProcessDataMiddleHold [4] && percentCD < percentTD){

        percentBD = (percentBD+percentTD)-percentCD;

        percentTD = percentCD;

    }

**else** **if** (totalNoOfNonDivCD < simProcessDataMiddleHold [4] && percentCD > percentTD){

        nonDivPercent = (percentCD+nonDivPercent)-percentTD;

        percentCD = percentTD;

    }

**int** entryCount = 0;

**for** (**int** counter1 = 0; counter1 < nonDivPercent; counter1++){

**if** (entryCount < 100) firstEventList [entryCount] = 4, entryCount++;

    }

**for** (**int** counter1 = 0; counter1 < percentCD; counter1++){

**if** (entryCount < 100) firstEventList [entryCount] = 3, entryCount++;

    }

**for** (**int** counter1 = 0; counter1 < percentTD; counter1++){

**if** (entryCount < 100) firstEventList [entryCount] = 2, entryCount++;

    }

**for** (**int** counter1 = 0; counter1 < percentBD; counter1++){

**if** (entryCount < 100) firstEventList [entryCount] = 1, entryCount++;

    }

**if** (entryCount != 100){

**for** (**int** counter1 = 0; counter1 < 100; counter1++){

**if** (entryCount < 100) firstEventList [entryCount] = 1, entryCount++;

        }

    }

**int** totalNoOfAfterBD = (**int**)(simProcessDataMiddleHold [3]+simProcessDataMiddleHold [4])+totalNoOfBDCD+totalNoOfBDCF;

    percentBD = 0;

    percentTD = 0;

**int** percentBDCD = 0;

**int** percentBDCF = 0;

**if** (totalNoOfAfterBD != 0) percentBD = (**int**)(round((simProcessDataMiddleHold [3]/(**double**)totalNoOfAfterBD)*100));

**if** (totalNoOfAfterBD != 0) percentTD = (**int**)(round((simProcessDataMiddleHold [4]/(**double**)totalNoOfAfterBD)*100));

**if** (totalNoOfAfterBD != 0) percentBDCD = (**int**)(round((totalNoOfBDCD/(**double**)totalNoOfAfterBD)*100));

**if** (totalNoOfAfterBD != 0) percentBDCF = (**int**)(round((totalNoOfBDCF/(**double**)totalNoOfAfterBD)*100));

**if** (totalNoOfAfterBD != 0){

**if** ((simProcessDataMiddleHold [3]/(**double**)totalNoOfAfterBD)*100 != 0 && percentBD == 0){

            percentBD = 1;

**if** (percentTD > 2) percentTD--;

**else** **if** (percentBDCD > 2) percentBDCD--;

**else** **if** (percentBDCF > 2) percentBDCF--;

        }

**if** ((simProcessDataMiddleHold [4]/(**double**)totalNoOfAfterBD)*100 != 0 && percentTD == 0){

            percentTD = 1;

**if** (percentBD > 2) percentBD--;

**else** **if** (percentBDCD > 2) percentBDCD--;

**else** **if** (percentBDCF > 2) percentBDCF--;

        }

**if** ((totalNoOfBDCD/(**double**)totalNoOfAfterBD)*100 != 0 && percentBDCD == 0){

            percentBDCD = 1;

**if** (percentBD > 2) percentBD--;

**else** **if** (percentTD > 2) percentTD--;

**else** **if** (percentBDCF > 2) percentBDCF--;

        }

**if** ((totalNoOfBDCF/(**double**)totalNoOfAfterBD)*100 != 0 && percentBDCF == 0){

            percentBDCF = 1;

**if** (percentBD > 2) percentBD--;

**else** **if** (percentTD > 2) percentTD--;

**else** **if** (percentBDCD > 2) percentBDCD--;

        }

    }

    entryCount = 0;

**for** (**int** counter1 = 0; counter1 < percentBDCF; counter1++){

**if** (entryCount < 100) secondEventBDList [entryCount] = 6, entryCount++;

    }

**for** (**int** counter1 = 0; counter1 < percentBDCD; counter1++){

**if** (entryCount < 100) secondEventBDList [entryCount] = 5, entryCount++;

    }

**for** (**int** counter1 = 0; counter1 < percentTD; counter1++){

**if** (entryCount < 100) secondEventBDList [entryCount] = 2, entryCount++;

    }

**for** (**int** counter1 = 0; counter1 < percentBD; counter1++){

**if** (entryCount < 100) secondEventBDList [entryCount] = 1, entryCount++;

    }

**if** (entryCount != 100){

**for** (**int** counter1 = 0; counter1 < 100; counter1++){

**if** (entryCount < 100) secondEventBDList [entryCount] = 1, entryCount++;

        }

    }

**int** totalNoOfAfterBDCF = (**int**)(simProcessDataMiddleHold [8]+simProcessDataMiddleHold [9])+totalNoOfBDCFCD;

**int** percentBDCFBD = 0;

**int** percentBDCFTD = 0;

**int** percentBDCFCD = 0;

**if** (totalNoOfAfterBDCF != 0) percentBDCFBD = (**int**)(round((simProcessDataMiddleHold [8]/(**double**)totalNoOfAfterBDCF)*100));

**if** (totalNoOfAfterBDCF != 0) percentBDCFTD = (**int**)(round((simProcessDataMiddleHold [9]/(**double**)totalNoOfAfterBDCF)*100));

**if** (totalNoOfAfterBDCF != 0) percentBDCFCD = (**int**)(round((totalNoOfBDCFCD/(**double**)totalNoOfAfterBDCF)*100));

**if** (totalNoOfAfterBDCF != 0){

**if** ((simProcessDataMiddleHold [8]/(**double**)totalNoOfAfterBDCF)*100 != 0 && percentBDCFBD == 0){

            percentBDCFBD = 1;

**if** (percentBDCFTD > 2) percentBDCFTD--;

**else** **if** (percentBDCFCD > 2) percentBDCFCD--;

        }

**if** ((simProcessDataMiddleHold [9]/(**double**)totalNoOfAfterBDCF)*100 != 0&& percentBDCFTD == 0){

            percentBDCFTD = 1;

**if** (percentBDCFBD > 2) percentBDCFBD--;

**else** **if** (percentBDCFCD > 2) percentBDCFCD--;

        }

**if** ((totalNoOfBDCFCD/(**double**)totalNoOfAfterBDCF)*100 != 0 && percentBDCFCD == 0){

            percentBDCFCD = 1;

**if** (percentBDCFBD > 2) percentBDCFBD--;

**else** **if** (percentBDCFTD > 2) percentBDCFTD--;

        }

    }

    entryCount = 0;

**int** lastEntry = 0;

**for** (**int** counter1 = 0; counter1 < percentBDCFBD; counter1++){

**if** (entryCount < 100){

            secondEventBDCFList [entryCount] = 7, entryCount++;

            lastEntry = 7;

        }

    }

**for** (**int** counter1 = 0; counter1 < percentBDCFTD; counter1++){

**if** (entryCount < 100){

            secondEventBDCFList [entryCount] = 8, entryCount++;

            lastEntry = 8;

        }

    }

**for** (**int** counter1 = 0; counter1 < percentBDCFCD; counter1++){

**if** (entryCount < 100){

            secondEventBDCFList [entryCount] = 9, entryCount++;

            lastEntry = 9;

        }

    }

**if** (entryCount != 0 && entryCount != 100){

**for** (**int** counter1 = 0; counter1 < 100; counter1++){

**if** (entryCount < 100) secondEventBDCFList [entryCount] = lastEntry, entryCount++;

        }

    }

**int** totalNoOfAfterTD = (**int**)(simProcessDataMiddleHold [15]+simProcessDataMiddleHold [16])+totalNoOfTDCD+totalNoOfTDCF;

**int** percentTDCF = 0;

**int** percentTDBD = 0;

**int** percentTDTD = 0;

**int** percentTDCD = 0;

**if** (totalNoOfAfterTD != 0) percentTDBD = (**int**)(round((simProcessDataMiddleHold [15]/(**double**)totalNoOfAfterTD)*100));

**if** (totalNoOfAfterTD != 0) percentTDTD = (**int**)(round((simProcessDataMiddleHold [16]/(**double**)totalNoOfAfterTD)*100));

**if** (totalNoOfAfterTD != 0) percentTDCD = (**int**)(round((totalNoOfTDCD/(**double**)totalNoOfAfterTD)*100));

**if** (totalNoOfAfterTD != 0) percentTDCF = (**int**)(round((totalNoOfTDCF/(**double**)totalNoOfAfterTD)*100));

**if** (totalNoOfAfterTD != 0){

**if** ((simProcessDataMiddleHold [15]/(**double**)totalNoOfAfterTD)*100 != 0 && percentTDBD == 0){

            percentTDBD = 1;

**if** (percentTDTD > 2) percentTDTD--;

**else** **if** (percentTDCD > 2) percentTDCD--;

**else** **if** (percentTDCF > 2) percentTDCF--;

        }

**if** ((simProcessDataMiddleHold [16]/(**double**)totalNoOfAfterTD)*100 != 0 && percentTDTD == 0){

            percentTDTD = 1;

**if** (percentTDBD > 2) percentTDBD--;

**else** **if** (percentTDCD > 2) percentTDCD--;

**else** **if** (percentTDCF > 2) percentTDCF--;

        }

**if** ((totalNoOfTDCD/(**double**)totalNoOfAfterTD)*100 != 0 && percentTDCD == 0){

            percentTDCD = 1;

**if** (percentTDBD > 2) percentTDBD--;

**else** **if** (percentTDTD > 2) percentTDTD--;

**else** **if** (percentTDCF > 2) percentTDCF--;

        }

**if** ((totalNoOfTDCF/(**double**)totalNoOfAfterTD)*100 != 0 && percentTDCF == 0){

            percentTDCF = 1;

**if** (percentTDBD > 2) percentTDBD--;

**else** **if** (percentTDTD > 2) percentTDTD--;

**else** **if** (percentTDCD > 2) percentTDCD--;

        }

    }

    entryCount = 0;

    lastEntry = 0;

**for** (**int** counter1 = 0; counter1 < percentTDCF; counter1++){

**if** (entryCount < 100){

            secondEventTDList [entryCount] = 10, entryCount++;

            lastEntry = 10;

        }

    }

**for** (**int** counter1 = 0; counter1 < percentTDBD; counter1++){

**if** (entryCount < 100){

            secondEventTDList [entryCount] = 11, entryCount++;

            lastEntry = 11;

        }

    }

**for** (**int** counter1 = 0; counter1 < percentTDTD; counter1++){

**if** (entryCount < 100){

            secondEventTDList [entryCount] = 12, entryCount++;

            lastEntry = 12;

        }

    }

**for** (**int** counter1 = 0; counter1 < percentTDCD; counter1++){

**if** (entryCount < 100){

            secondEventTDList [entryCount] = 13, entryCount++;

            lastEntry = 13;

        }

    }

**if** (entryCount != 0 && entryCount != 100){

**for** (**int** counter1 = 0; counter1 < 100; counter1++){

**if** (entryCount < 100) secondEventTDList [entryCount] = lastEntry, entryCount++;

        }

    }

**int** totalNoOfAfterTDCF = (**int**)(simProcessDataMiddleHold [12]+simProcessDataMiddleHold [13])+totalNoOfTDCFCD;

**int** percentTDCFBD = 0;

**int** percentTDCFTD = 0;

**int** percentTDCFCD = 0;

**if** (totalNoOfAfterTDCF != 0) percentTDCFBD = (**int**)(round((simProcessDataMiddleHold [12]/(**double**)totalNoOfAfterTDCF)*100));

**if** (totalNoOfAfterTDCF != 0) percentTDCFTD = (**int**)(round((simProcessDataMiddleHold [13]/(**double**)totalNoOfAfterTDCF)*100));

**if** (totalNoOfAfterTDCF != 0) percentTDCFCD = (**int**)(round((totalNoOfTDCFCD/(**double**)totalNoOfAfterTDCF)*100));

**if** (totalNoOfAfterTDCF != 0){

**if** ((simProcessDataMiddleHold [12]/(**double**)totalNoOfAfterTDCF)*100 != 0 && percentTDCFBD == 0){

            percentTDCFBD = 1;

**if** (percentTDCFTD > 2) percentTDCFTD--;

**else** **if** (percentTDCFCD > 2) percentTDCFCD--;

        }

**if** ((simProcessDataMiddleHold [13]/(**double**)totalNoOfAfterTDCF)*100 != 0&& percentTDCFTD == 0){

            percentTDCFTD = 1;

**if** (percentTDCFBD > 2) percentTDCFBD--;

**else** **if** (percentTDCFCD > 2) percentTDCFCD--;

        }

**if** ((totalNoOfTDCFCD/(**double**)totalNoOfAfterTDCF)*100 != 0 && percentTDCFCD == 0){

            percentTDCFCD = 1;

**if** (percentTDCFBD > 2) percentTDCFBD--;

**else** **if** (percentTDCFTD > 2) percentTDCFTD--;

        }

    }

    entryCount = 0;

    lastEntry = 0;

**for** (**int** counter1 = 0; counter1 < percentTDCFBD; counter1++){

**if** (entryCount < 100){

            secondEventTDCFList [entryCount] = 14, entryCount++;

            lastEntry = 14;

        }

    }

**for** (**int** counter1 = 0; counter1 < percentTDCFTD; counter1++){

**if** (entryCount < 100){

            secondEventTDCFList [entryCount] = 15, entryCount++;

            lastEntry = 15;

        }

    }

**for** (**int** counter1 = 0; counter1 < percentTDCFCD; counter1++){

**if** (entryCount < 100){

            secondEventTDCFList [entryCount] = 16, entryCount++;

            lastEntry = 16;

        }

    }

**if** (entryCount != 0 && entryCount != 100){

**for** (**int** counter1 = 0; counter1 < 100; counter1++){

**if** (entryCount < 100) secondEventTDCFList [entryCount] = lastEntry, entryCount++;

        }

    }

**int** randInit = 0;

**int** extendEnd = 0;

**int** newCellNumber1 = 0;

**int** newCellNumber2 = 0;

**int** newCellNumber3 = 0;

    activeCellStatusListKeepCount = 0;

    string cellNumberExtract;

    string lineageNumberExtract;

    string newExtensionEntryNo;

    string degitExtract;

**if** (doseSimStatusHold != 2){

**int** totalEvent = (**int**)(simProcessDataMiddleHold [3]+simProcessDataMiddleHold [4])+totalNoOfBDCD+totalNoOfBDCF;

**int** percentTotalBD = (**int**)(round(simProcessDataMiddleHold [3]/(**double**)totalEvent));

**int** percentTotalTD = (**int**)(round(simProcessDataMiddleHold [4]/(**double**)totalEvent));

**int** percentTotalCD = (**int**)(round(totalNoOfBDCD/(**double**)totalEvent));

**int** percentTotalCF = (**int**)(round(totalNoOfBDCF/(**double**)totalEvent));

**int** countBD = 0;

**int** countTD = 0;

**int** countCD = 0;

**int** countCF = 0;

**for** (**int** counter2 = 0; counter2 < 1000; counter2++){

            randInit = rand() % 100 + 0;

**if** (secondEventBDList [randInit] == 1) countBD++;

**else** **if** (secondEventBDList [randInit] == 2) countTD++;

**else** **if** (secondEventBDList [randInit] == 5) countCD++;

**else** **if** (secondEventBDList [randInit] == 6) countCF++;

        }

**int** totalCheck = countBD+countTD+countCD+countCF;

**int** percentCheckTD = 0;

**int** percentCheckCD = 0;

**int** percentCheckCF = 0;

**if** (totalCheck != 0){

            percentCheckTD = (**int**)(round(countTD/(**double**)totalCheck));

            percentCheckCD = (**int**)(round(countCD/(**double**)totalCheck));

            percentCheckCF = (**int**)(round(countCF/(**double**)totalCheck));

**int** checkCount = 0;

**if** (percentCheckTD-percentTotalTD < 0 && percentTotalTD-percentCheckTD < percentTotalBD){

                checkCount = percentTotalTD-percentCheckTD;

**for** (**int** counter2 = 0; counter2 < 100; counter2++){

**if** (secondEventBDList [counter2] == 1){

                        secondEventBDList [counter2] = 2;

                        checkCount--;

**if** (checkCount == 0){

**break**;

                        }

                    }

                }

            }

**if** (percentCheckCD-percentTotalCD < 0 && percentTotalCD-percentCheckCD < percentTotalBD-(percentTotalTD-percentCheckTD)){

                checkCount = percentTotalCD-percentCheckCD;

**for** (**int** counter2 = 0; counter2 < 100; counter2++){

**if** (secondEventBDList [counter2] == 1){

                        secondEventBDList [counter2] = 5;

                        checkCount--;

**if** (checkCount == 0){

**break**;

                        }

                    }

                }

            }

**if** (percentCheckCF-percentTotalCF < 0 && percentTotalCF-percentCheckCF < percentTotalBD-(percentTotalTD-percentCheckTD)-(percentTotalCD-percentCheckCD)){

                checkCount = percentTotalCF-percentCheckCF;

**for** (**int** counter2 = 0; counter2 < 100; counter2++){

**if** (secondEventBDList [counter2] == 1){

                        secondEventBDList [counter2] = 6;

                        checkCount--;

**if** (checkCount == 0){

**break**;

                        }

                    }

                }

            }

        }

    }

**else**{

        [**self** secondArraySet];

**int** totalEvent = (**int**)(round(simProcessDataAddHold [3]+simProcessDataAddHold [4]))+totalNoOfBDCD+totalNoOfBDCF;

**int** percentTotalBD = (**int**)(round(simProcessDataAddHold [3]/(**double**)totalEvent));

**int** percentTotalTD = (**int**)(round(simProcessDataAddHold [4]/(**double**)totalEvent));

**int** percentTotalCD = (**int**)(round(totalNoOfBDCD/(**double**)totalEvent));

**int** percentTotalCF = (**int**)(round(totalNoOfBDCF/(**double**)totalEvent));

**int** countBD = 0;

**int** countTD = 0;

**int** countCD = 0;

**int** countCF = 0;

**for** (**int** counter2 = 0; counter2 < 1000; counter2++){

            randInit = rand() % 100 + 0;

**if** (secondEventBDListSel [randInit] == 1) countBD++;

**else** **if** (secondEventBDListSel [randInit] == 2) countTD++;

**else** **if** (secondEventBDListSel [randInit] == 5) countCD++;

**else** **if** (secondEventBDListSel [randInit] == 6) countCF++;

        }

**int** totalCheck = countBD+countTD+countCD+countCF;

**int** percentCheckTD = 0;

**int** percentCheckCD = 0;

**int** percentCheckCF = 0;

**if** (totalCheck != 0){

            percentCheckTD = (**int**)(round(countTD/(**double**)totalCheck));

            percentCheckCD = (**int**)(round(countCD/(**double**)totalCheck));

            percentCheckCF = (**int**)(round(countCF/(**double**)totalCheck));

**int** checkCount = 0;

**if** (percentCheckTD-percentTotalTD < 0 && percentTotalTD-percentCheckTD < percentTotalBD){

                checkCount = percentTotalTD-percentCheckTD;

**for** (**int** counter2 = 0; counter2 < 100; counter2++){

**if** (secondEventBDListSel [counter2] == 1){

                        secondEventBDListSel [counter2] = 2;

                        checkCount--;

**if** (checkCount == 0){

**break**;

                        }

                    }

                }

            }

**if** (percentCheckCD-percentTotalCD < 0 && percentTotalCD-percentCheckCD < percentTotalBD-(percentTotalTD-percentCheckTD)){

                checkCount = percentTotalCD-percentCheckCD;

**for** (**int** counter2 = 0; counter2 < 100; counter2++){

**if** (secondEventBDListSel [counter2] == 1){

                        secondEventBDListSel [counter2] = 5;

                        checkCount--;

**if** (checkCount == 0){

**break**;

                        }

                    }

                }

            }

**if** (percentCheckCF-percentTotalCF < 0 && percentTotalCF-percentCheckCF < percentTotalBD-(percentTotalTD-percentCheckTD)-(percentTotalCD-percentCheckCD)){

                checkCount = percentTotalCF-percentCheckCF;

**for** (**int** counter2 = 0; counter2 < 100; counter2++){

**if** (secondEventBDListSel [counter2] == 1){

                        secondEventBDListSel [counter2] = 6;

                        checkCount--;

**if** (checkCount == 0){

**break**;

                        }

                    }

                }

            }

        }

    }

**int** *activeCellStatusListHold = **new** **int** [activeCellStatusListCount+10];

**int** activeCellStatusListHoldCount = 0;

**for** (**int** counter1 = 0; counter1 < activeCellStatusListCount; counter1++) activeCellStatusListHold [activeCellStatusListHoldCount] = activeCellStatusList [counter1], activeCellStatusListHoldCount++;

**int** terminationFlag = 0;

**int** lowextValue = 0;

**int** highestValue = 0;

**int** eventCount = 0;

**int** eventType = 0;

**int** loopCheck = 0;

**int** siblinFusionCheck1 = 0;

**int** siblinFusionCheck2 = 0;

**int** siblinFusionDoub1 = 0;

**int** siblinFusionDoub2 = 0;

**int** siblinFusionDoubLarge = 0;

**int** siblingCellNo1 = 0;

**int** siblingCellNo2 = 0;

**int** cellDoubLimitCheck = 0;

**int** siblingCellPosition1 = 0;

**int** siblingCellPosition2 = 0;

**int** siblingCellPositionSelect = 0;

**int** siblingCellNoSelect = 0;

**int** siblingCellDoubSelect = 0;

**int** lineageAddTempCount = 0;

**int** lineageAddTempLimit = 0;

**int** parentTDCount = 0;

**int** parentTDTD = 0;

**int** parentTDBD = 0;

**int** fusionCount = 0;

**int** reachMaxDivision = 1000000000;

**int** timeKeep = 0;

**int** arrayOverflow = 0;

**int** newLimit = 0;

**int** startPositionListCount = 0;

**int** cellNoFusionCheckHold = 0;

**int** missingPartnerCheck = 0;

**int** selectChange = 0;

**int** firstTimeAssiginment = 0;

**int** cellNoforSummary = 0;

**int** clingNoforSummary = 0;

**int** fusionForSummary = 0;

**int** cycleMaxReachFlag = 0;

**int** nonDivAssign = 0;

**int** otherDivAssign = 0;

**int** callCount = 0;

**unsigned** **long** setTime = 0;

**double** dataTempDouble = 0;

**unsigned** **long** fusionPartnerListCount = 0;

**unsigned** **long** entryCount2 = 0;

    string cellNumberString;

**int** activeCellStatusListTempCount = 0;

**int** randBDRangeA = (**int**)(round(simProcessDataMiddleHold [26]-simProcessDataMiddleHold [26]*0.25));

**int** randBDRangeB = (**int**)(round(simProcessDataMiddleHold [26]+simProcessDataMiddleHold [26]*0.25));

**int** randCDRangeA = (**int**)(round(simProcessDataMiddleHold [27]-simProcessDataMiddleHold [27]*0.25));

**int** randCDRangeB = (**int**)(round(simProcessDataMiddleHold [27]+simProcessDataMiddleHold [27]*0.25));

**if** (randBDRangeA == 0 && randBDRangeB == 0){

        randBDRangeA = (**int**)(round(simProcessDataMiddleHold [25]*0.8-simProcessDataMiddleHold [25]*0.8*0.25));

        randBDRangeB = (**int**)(round(simProcessDataMiddleHold [25]*0.8));

    }

**if** (randCDRangeA == 0 && randCDRangeB == 0){

        randCDRangeA = (**int**)(round(simProcessDataMiddleHold [25]*0.5-simProcessDataMiddleHold [25]*0.5*0.25));

        randCDRangeB = (**int**)(round(simProcessDataMiddleHold [25]*0.5));

    }

**int** loopCount = 0;

**int** terminate2 = 0;

**int** selectCheck = 0;

**do**{

        processingStatusCall = 1;

        loopCount++;

        processingStatus = "Mid: C"+to_string (loopCount);

        terminationFlag = 1;

        fusionCount = 0;

**for** (**int** counter1 = 0; counter1 < activeCellStatusListCount/12; counter1++){

**if** (terminateSimFlag == 1){

                terminate2 = 1;

**break**;

            }

            selectCheck = 0;

**if** (doseSimStatusHold == 2){

**for** (**int** counter3 = 0; counter3 < lingNoAssigineSimCount; counter3++){

**if** (lingNoAssigineSim [counter3] == activeCellStatusList [counter1*12+3]){

                        selectCheck = 1;

**break**;

                    }

                }

            }

            cellDoubLimitCheck = activeCellStatusList [counter1*12];

**if** (cellDoubLimitCheck < 0) cellDoubLimitCheck = cellDoubLimitCheck*-1;

            cellNumberString = to_string(cellDoubLimitCheck);

**if** (((**int**)cellNumberString.length() == 9 && (cellNumberString.substr(0, 1) == "5" || cellNumberString.substr(0, 1) == "6") && cellNumberString.substr(cellNumberString.length()-1) != "0") || cycleMaxReachFlag == 1){

                activeCellStatusList [counter1*12+4] = 50;

                activeCellStatusList [counter1*12+5] = 9;

                activeCellStatusList [counter1*12+6] = 1;

                cycleMaxReachFlag = 1;

            }

**else**{

**if** (selectCheck == 0){

**if** (activeCellStatusList [counter1*12+5] == 5){

                        dataTempDouble = ((expandNonCDCount/(**double**)2)/(**double**)simProcessDataMiddleHold [24])*100;

                        randInit = rand() % 100 + 0;

**if** (randInit < dataTempDouble && expandNonCDCount != 0){

                            randInit = rand() % expandNonCDCount/2 + 0;

                            activeCellStatusList [counter1*12+4] = expandNonCD [randInit*2];

                            activeCellStatusList [counter1*12+5] = 30;

                            activeCellStatusList [counter1*12+6] = 1;

                        }

**else**{

                            randInit = rand() % (**int**)(round(simProcessDataMiddleHold [25]*(endVariationtHold/(**double**)100))) + 0;

**if** (randInit%2 == 0) activeCellStatusList [counter1*12+4] = (**int**)simProcessDataMiddleHold [25]+randInit;

**else** activeCellStatusList [counter1*12+4] = (**int**)simProcessDataMiddleHold [25]-randInit;

                            activeCellStatusList [counter1*12+5] = 5;

                            activeCellStatusList [counter1*12+6] = 1;

                        }

                    }

**else** **if** (activeCellStatusList [counter1*12+5] == 7){

                        eventType = 1;

**if** (eventType == 1){

**if** (activeCellStatusList [counter1*12+11] == 0){

**if** (expandDoublingDoubBDCount != 0){

                                    randInit = rand() % expandDoublingDoubBDCount/2 + 0;

                                    activeCellStatusList [counter1*12+4] = expandDoublingDoubBD [randInit*2];

                                    activeCellStatusList [counter1*12+5] = 1;

                                    activeCellStatusList [counter1*12+6] = 1;

                                }

**else**{

                                    randInit = rand() % randBDRangeB + randBDRangeA;

                                    activeCellStatusList [counter1*12+4] = randInit;

                                    activeCellStatusList [counter1*12+5] = 7;

                                    activeCellStatusList [counter1*12+6] = 1;

                                }

                            }

**else**{

                                loopCheck = 0;

**for** (**int** counter2 = 0; counter2 < 100; counter2++){

**if** (expandDoublingDoubBDCount != 0){

                                        randInit = rand() % expandDoublingDoubBDCount/2 + 0;

**if** (expandDoublingDoubBD [randInit*2] > activeCellStatusList [counter1*12+11]-50 && expandDoublingDoubBD [randInit*2] < activeCellStatusList [counter1*12+11]+50){

                                            activeCellStatusList [counter1*12+4] = expandDoublingDoubBD [randInit*2];

                                            activeCellStatusList [counter1*12+5] = 1;

                                            activeCellStatusList [counter1*12+6] = 1;

                                            loopCheck = 1;

**break**;

                                        }

                                    }

                                }

**if** (loopCheck == 0){

                                    activeCellStatusList [counter1*12+4] = activeCellStatusList [counter1*12+11];

                                    activeCellStatusList [counter1*12+5] = 7;

                                    activeCellStatusList [counter1*12+6] = 1;

                                }

                            }

                        }

                    }

**else** **if** (activeCellStatusList [counter1*12+5] == 1){

                        siblinFusionCheck1 = 0;

                        siblinFusionDoub1 = 0;

                        siblingCellNo1 = 0;

**for** (**int** counter2 = 0; counter2 < activeCellStatusListCount/12; counter2++){

**if** (activeCellStatusList [counter2*12+6] == 1 && activeCellStatusList [counter2*12+1] == activeCellStatusList [counter1*12] && activeCellStatusList [counter2*12+3] == activeCellStatusList [counter1*12+3]){

                                siblingCellNo1 = counter2;

**if** (activeCellStatusList [counter2*12+5] != 0){

                                    siblinFusionCheck1 = activeCellStatusList [counter2*12+5];

                                    siblinFusionDoub1 = activeCellStatusList [counter2*12+4];

                                }

**break**;

                            }

                        }

                        eventType = 0;

**if** (activeCellStatusList [counter1*12+9] == 0){

**if** (siblinFusionCheck1 == 6){

                                loopCheck = 0;

**for** (**int** counter2 = 0; counter2 < 100; counter2++){

                                    randInit = rand() % 100 + 0;

**if** (secondEventBDList [randInit] != 6){

                                        eventType = secondEventBDList [randInit];

                                        loopCheck = 1;

**break**;

                                    }

                                }

**if** (loopCheck == 0) eventType = 1;

                            }

**else**{

                                randInit = rand() % 100 + 0;

**if** (secondEventBDList [randInit] != 0) eventType = secondEventBDList [randInit];

**else** eventType = 1;

                            }

                        }

**else** **if** (activeCellStatusList [counter1*12+9] != 0){

                            eventCount = 0;

                            eventType = 0;

**if** (siblinFusionCheck1 == 6) eventType = 5;

**else**{

**for** (**int** counter2 = 0; counter2 < 5; counter2++){

                                    randInit = rand() % 100 + 0;

**if** (secondEventBDList [randInit] == 1) eventCount++;

**else**{

                                        eventType = secondEventBDList [randInit];

**break**;

                                    }

                                }

**if** (eventCount == 5 || eventType == 0) eventType = 1;

                            }

                        }

**if** (firstTimeAssiginment == 0 && eventType == 1){

                            randInit = rand() % 100 + 0;

**if** (firstEventList [randInit] != 0){

**if** (firstEventList [randInit] == 3) eventType = 5;

**else** **if** (firstEventList [randInit] == 4) eventType = 10;

**else** eventType = firstEventList [randInit];

                            }

                        }

**if** (eventType == 1){

**if** (activeCellStatusList [counter1*12+11] == 0){

**if** (siblinFusionCheck1 != 6){

**if** (expandDoublingDoubBDCount != 0){

                                        randInit = rand() % expandDoublingDoubBDCount/2 + 0;

                                        activeCellStatusList [counter1*12+4] = expandDoublingDoubBD [randInit*2];

                                        activeCellStatusList [counter1*12+5] = 1;

                                        activeCellStatusList [counter1*12+6] = 1;

**if** (activeCellStatusList [counter1*12+9] != 0) activeCellStatusList [counter1*12+9]--;

                                    }

**else**{

                                        randInit = rand() % randBDRangeB + randBDRangeA;

                                        activeCellStatusList [counter1*12+4] = randInit;

                                        activeCellStatusList [counter1*12+5] = 1;

                                        activeCellStatusList [counter1*12+6] = 1;

**if** (activeCellStatusList [counter1*12+9] != 0) activeCellStatusList [counter1*12+9]--;

                                    }

                                }

**else**{

**if** (siblinFusionDoub1 < randBDRangeA) siblinFusionDoub1 = randBDRangeA;

                                    activeCellStatusList [counter1*12+4] = siblinFusionDoub1+10;

                                    activeCellStatusList [counter1*12+5] = 1;

                                    activeCellStatusList [counter1*12+6] = 1;

**if** (activeCellStatusList [counter1*12+9] != 0) activeCellStatusList [counter1*12+9]--;

                                }

                            }

**else**{

                                loopCheck = 0;

**for** (**int** counter2 = 0; counter2 < 100; counter2++){

**if** (siblinFusionCheck1 != 6){

**if** (expandDoublingDoubBDCount != 0){

                                            randInit = rand() % expandDoublingDoubBDCount/2 + 0;

**if** (expandDoublingDoubBD [randInit*2] > activeCellStatusList [counter1*12+11]-50 && expandDoublingDoubBD [randInit*2] < activeCellStatusList [counter1*12+11]+50){

                                                activeCellStatusList [counter1*12+4] = expandDoublingDoubBD [randInit*2];

                                                activeCellStatusList [counter1*12+5] = 1;

                                                activeCellStatusList [counter1*12+6] = 1;

**if** (activeCellStatusList [counter1*12+9] != 0) activeCellStatusList [counter1*12+9]--;

                                                loopCheck = 1;

**break**;

                                            }

                                        }

                                    }

**else** **if** (siblinFusionCheck1 == 6){

**if** (expandDoublingDoubBDCount != 0){

                                            randInit = rand() % expandDoublingDoubBDCount/2 + 0;

**if** (expandDoublingDoubBD [randInit*2] > activeCellStatusList [counter1*12+11]-50 && expandDoublingDoubBD [randInit*2] < activeCellStatusList [counter1*12+11]+50 && siblinFusionDoub1+10 < expandDoublingDoubBD [randInit*2]){

                                                activeCellStatusList [counter1*12+4] = expandDoublingDoubBD [randInit*2];

                                                activeCellStatusList [counter1*12+5] = 1;

                                                activeCellStatusList [counter1*12+6] = 1;

**if** (activeCellStatusList [counter1*12+9] != 0) activeCellStatusList [counter1*12+9]--;

                                                loopCheck = 1;

**break**;

                                            }

                                        }

                                    }

                                }

**if** (loopCheck == 0){

                                    lowextValue = 100000;

                                    highestValue = 0;

**for** (**int** counter2 = 0; counter2 < expandDoublingDoubBDCount/2; counter2++){

**if** (lowextValue > expandDoublingDoubBD [counter2*2]) lowextValue = expandDoublingDoubBD [counter2*2];

**if** (highestValue < expandDoublingDoubBD [counter2*2]) highestValue = expandDoublingDoubBD [counter2*2];

                                    }

**if** (siblinFusionDoub1 < randBDRangeA) siblinFusionDoub1 = randBDRangeA;

**if** (lowextValue != 100000 && highestValue != 0){

**if** ((highestValue+lowextValue)/(**double**)2 > siblinFusionDoub1+10){

                                            activeCellStatusList [counter1*12+4] = (**int**)(round((highestValue+lowextValue)/(**double**)2));

                                            activeCellStatusList [counter1*12+5] = 1;

                                            activeCellStatusList [counter1*12+6] = 1;

**if** (activeCellStatusList [counter1*12+9] != 0) activeCellStatusList [counter1*12+9]--;

                                        }

**else**{

                                            activeCellStatusList [counter1*12+4] = siblinFusionDoub1+10;

                                            activeCellStatusList [counter1*12+5] = 1;

                                            activeCellStatusList [counter1*12+6] = 1;

**if** (activeCellStatusList [counter1*12+9] != 0) activeCellStatusList [counter1*12+9]--;

                                        }

                                    }

**else**{

                                        activeCellStatusList [counter1*12+4] = siblinFusionDoub1+10;

                                        activeCellStatusList [counter1*12+5] = 1;

                                        activeCellStatusList [counter1*12+6] = 1;

**if** (activeCellStatusList [counter1*12+9] != 0) activeCellStatusList [counter1*12+9]--;

                                    }

                                }

                            }

                        }

**else** **if** (eventType == 2){

**if** (activeCellStatusList [counter1*12+11] == 0){

**if** (siblinFusionCheck1 != 6){

**if** (expandDoublingDoubBDCount != 0){

                                        randInit = rand() % expandDoublingDoubBDCount/2 + 0;

                                        activeCellStatusList [counter1*12+4] = expandDoublingDoubBD [randInit*2];

                                        activeCellStatusList [counter1*12+5] = 2;

                                        activeCellStatusList [counter1*12+6] = 1;

                                        activeCellStatusList [counter1*12+7] = 1;

                                        activeCellStatusList [counter1*12+9] = 0;

                                        activeCellStatusList [counter1*12+8] = (**int**)simProcessDataMiddleHold [18];

                                    }

**else**{

                                        randInit = rand() % randBDRangeB + randBDRangeA;

                                        activeCellStatusList [counter1*12+4] = randInit;

                                        activeCellStatusList [counter1*12+5] = 2;

                                        activeCellStatusList [counter1*12+6] = 1;

                                        activeCellStatusList [counter1*12+7] = 1;

                                        activeCellStatusList [counter1*12+9] = 0;

                                        activeCellStatusList [counter1*12+8] = (**int**)simProcessDataMiddleHold [18];

                                    }

                                }

**else**{

**if** (siblinFusionDoub1 < randBDRangeA) siblinFusionDoub1 = randBDRangeA;

                                    activeCellStatusList [counter1*12+4] = siblinFusionDoub1+10;

                                    activeCellStatusList [counter1*12+5] = 2;

                                    activeCellStatusList [counter1*12+6] = 1;

                                    activeCellStatusList [counter1*12+7] = 1;

                                    activeCellStatusList [counter1*12+9] = 0;

                                    activeCellStatusList [counter1*12+8] = (**int**)simProcessDataMiddleHold [18];

                                }

                            }

**else**{

                                loopCheck = 0;

**for** (**int** counter2 = 0; counter2 < 100; counter2++){

**if** (siblinFusionCheck1 != 6){

**if** (expandDoublingDoubBDCount != 0){

                                            randInit = rand() % expandDoublingDoubBDCount/2 + 0;

**if** (expandDoublingDoubBD [randInit*2] > activeCellStatusList [counter1*12+11]-50 && expandDoublingDoubBD [randInit*2] < activeCellStatusList [counter1*12+11]+50){

                                                activeCellStatusList [counter1*12+4] = expandDoublingDoubBD [randInit*2];

                                                activeCellStatusList [counter1*12+5] = 2;

                                                activeCellStatusList [counter1*12+6] = 1;

                                                activeCellStatusList [counter1*12+7] = 1;

                                                activeCellStatusList [counter1*12+9] = 0;

                                                activeCellStatusList [counter1*12+8] = (**int**)simProcessDataMiddleHold [18];

                                                loopCheck = 1;

**break**;

                                            }

                                        }

                                    }

**else** **if** (siblinFusionCheck1 == 6){

**if** (expandDoublingDoubBDCount != 0){

                                            randInit = rand() % expandDoublingDoubBDCount/2 + 0;

**if** (expandDoublingDoubBD [randInit*2] > activeCellStatusList [counter1*12+11]-50 && expandDoublingDoubBD [randInit*2] < activeCellStatusList [counter1*12+11]+50 && siblinFusionDoub1+10 < expandDoublingDoubBD [randInit*2]){

                                                activeCellStatusList [counter1*12+4] = expandDoublingDoubBD [randInit*2];

                                                activeCellStatusList [counter1*12+5] = 2;

                                                activeCellStatusList [counter1*12+6] = 1;

                                                activeCellStatusList [counter1*12+7] = 1;

                                                activeCellStatusList [counter1*12+9] = 0;

                                                activeCellStatusList [counter1*12+8] = (**int**)simProcessDataMiddleHold [18];

                                                loopCheck = 1;

**break**;

                                            }

                                        }

                                    }

                                }

**if** (loopCheck == 0){

                                    lowextValue = 100000;

                                    highestValue = 0;

**for** (**int** counter2 = 0; counter2 < expandDoublingDoubBDCount/2; counter2++){

**if** (lowextValue > expandDoublingDoubBD [counter2*2]) lowextValue = expandDoublingDoubBD [counter2*2];

**if** (highestValue < expandDoublingDoubBD [counter2*2]) highestValue = expandDoublingDoubBD [counter2*2];

                                    }

**if** (siblinFusionDoub1 < randBDRangeA) siblinFusionDoub1 = randBDRangeA;

**if** (lowextValue != 100000 && highestValue != 0){

**if** ((highestValue+lowextValue)/(**double**)2 > siblinFusionDoub1+10){

                                            activeCellStatusList [counter1*12+4] = (**int**)(round((highestValue+lowextValue)/(**double**)2));

                                            activeCellStatusList [counter1*12+5] = 2;

                                            activeCellStatusList [counter1*12+6] = 1;

                                            activeCellStatusList [counter1*12+7] = 1;

                                            activeCellStatusList [counter1*12+9] = 0;

                                            activeCellStatusList [counter1*12+8] = (**int**)simProcessDataMiddleHold [18];

                                        }

**else**{

                                            activeCellStatusList [counter1*12+4] = siblinFusionDoub1+10;

                                            activeCellStatusList [counter1*12+5] = 2;

                                            activeCellStatusList [counter1*12+6] = 1;

                                            activeCellStatusList [counter1*12+7] = 1;

                                            activeCellStatusList [counter1*12+9] = 0;

                                            activeCellStatusList [counter1*12+8] = (**int**)simProcessDataMiddleHold [18];

                                        }

                                    }

**else**{

                                        activeCellStatusList [counter1*12+4] = siblinFusionDoub1+10;

                                        activeCellStatusList [counter1*12+5] = 2;

                                        activeCellStatusList [counter1*12+6] = 1;

                                        activeCellStatusList [counter1*12+7] = 1;

                                        activeCellStatusList [counter1*12+9] = 0;

                                        activeCellStatusList [counter1*12+8] = (**int**)simProcessDataMiddleHold [18];

                                    }

                                }

                            }

                        }

**else** **if** (eventType == 5){

**if** (expandBDCDCount == 0){

                                randInit = rand() % randCDRangeB + randCDRangeA;

**if** (siblinFusionCheck1 != 6){

                                    activeCellStatusList [counter1*12+4] = randInit;

                                    activeCellStatusList [counter1*12+5] = 3;

                                    activeCellStatusList [counter1*12+6] = 1;

                                    activeCellStatusList [counter1*12+9] = 0;

                                }

**else**{

                                    activeCellStatusList [counter1*12+4] = siblinFusionDoub1+10;

                                    activeCellStatusList [counter1*12+5] = 3;

                                    activeCellStatusList [counter1*12+6] = 1;

                                    activeCellStatusList [counter1*12+9] = 0;

                                }

                            }

**else**{

**if** (siblinFusionCheck1 != 6){

**if** (expandBDCDCount != 0){

                                        randInit = rand() % expandBDCDCount/2 + 0;

                                        activeCellStatusList [counter1*12+4] = expandBDCD [randInit*2];

                                        activeCellStatusList [counter1*12+5] = 3;

                                        activeCellStatusList [counter1*12+6] = 1;

                                        activeCellStatusList [counter1*12+9] = 0;

                                    }

**else**{

                                        activeCellStatusList [counter1*12+4] = siblinFusionDoub1+10;

                                        activeCellStatusList [counter1*12+5] = 3;

                                        activeCellStatusList [counter1*12+6] = 1;

                                        activeCellStatusList [counter1*12+9] = 0;

                                    }

                                }

**else**{

**if** (expandBDCDCount != 0){

                                        randInit = rand() % expandBDCDCount/2 + 0;

**if**(expandBDCD [randInit*2] > siblinFusionDoub1){

                                            activeCellStatusList [counter1*12+4] = expandBDCD [randInit*2]+10;

                                            activeCellStatusList [counter1*12+5] = 3;

                                            activeCellStatusList [counter1*12+6] = 1;

                                            activeCellStatusList [counter1*12+9] = 0;

                                        }

**else**{

                                            activeCellStatusList [counter1*12+4] = siblinFusionDoub1+10;

                                            activeCellStatusList [counter1*12+5] = 3;

                                            activeCellStatusList [counter1*12+6] = 1;

                                            activeCellStatusList [counter1*12+9] = 0;

                                        }

                                    }

**else**{

                                        activeCellStatusList [counter1*12+4] = siblinFusionDoub1+10;

                                        activeCellStatusList [counter1*12+5] = 3;

                                        activeCellStatusList [counter1*12+6] = 1;

                                        activeCellStatusList [counter1*12+9] = 0;

                                    }

                                }

                            }

                        }

**else** **if** (eventType == 6){

**if** (expandBDCFCount == 0){

                                randInit = rand() % randCDRangeB + randCDRangeA;

**if** (siblinFusionCheck1 != 6){

                                    activeCellStatusList [counter1*12+4] = randInit;

                                    activeCellStatusList [counter1*12+5] = 6;

                                    activeCellStatusList [counter1*12+6] = 1;

                                    activeCellStatusList [counter1*12+9] = 0;

                                }

**else**{

**if** (siblinFusionDoub1 < randBDRangeA) siblinFusionDoub1 = randBDRangeA;

                                    activeCellStatusList [siblingCellNo1*12+4] = siblinFusionDoub1+10;

                                    activeCellStatusList [counter1*12+4] = siblinFusionDoub1;

                                    activeCellStatusList [counter1*12+5] = 6;

                                    activeCellStatusList [counter1*12+6] = 1;

                                    activeCellStatusList [counter1*12+9] = 0;

                                }

                            }

**else**{

**if** (siblinFusionCheck1 == 0){

**if** (expandBDCFCount != 0){

                                        randInit = rand() % expandBDCFCount/2 + 0;

                                        activeCellStatusList [counter1*12+4] = expandBDCF [randInit*2];

                                        activeCellStatusList [counter1*12+5] = 6;

                                        activeCellStatusList [counter1*12+6] = 1;

                                        activeCellStatusList [counter1*12+9] = 0;

                                    }

**else** {

                                        activeCellStatusList [counter1*12+4] = siblinFusionDoub1+10;

                                        activeCellStatusList [counter1*12+5] = 6;

                                        activeCellStatusList [counter1*12+6] = 1;

                                        activeCellStatusList [counter1*12+9] = 0;

                                    }

                                }

**else**{

**if** (expandBDCDCount != 0){

                                        randInit = rand() % expandBDCDCount/2 + 0;

**if**(expandBDCD [randInit*2] > siblinFusionDoub1){

                                            activeCellStatusList [counter1*12+4] = expandBDCD [randInit*2]+10;

                                            activeCellStatusList [counter1*12+5] = 3;

                                            activeCellStatusList [counter1*12+6] = 1;

                                            activeCellStatusList [counter1*12+9] = 0;

                                        }

**else**{

                                            activeCellStatusList [counter1*12+4] = siblinFusionDoub1+10;

                                            activeCellStatusList [counter1*12+5] = 3;

                                            activeCellStatusList [counter1*12+6] = 1;

                                            activeCellStatusList [counter1*12+9] = 0;

                                        }

                                    }

**else**{

                                        activeCellStatusList [siblingCellNo1*12+4] = siblinFusionDoub1+10;

                                        activeCellStatusList [counter1*12+4] = siblinFusionDoub1;

                                        activeCellStatusList [counter1*12+5] = 6;

                                        activeCellStatusList [counter1*12+6] = 1;

                                        activeCellStatusList [counter1*12+9] = 0;

                                    }

                                }

                            }

                        }

**else** **if** (eventType == 10){

                            dataTempDouble = ((expandNonCDCount/(**double**)2)/(**double**)simProcessDataMiddleHold [24])*100;

                            randInit = rand() % 100 + 0;

**if** (randInit < dataTempDouble && expandNonCDCount != 0){

                                randInit = rand() % expandNonCDCount/2 + 0;

                                activeCellStatusList [counter1*12+4] = expandNonCD [randInit*2];

                                activeCellStatusList [counter1*12+5] = 30;

                                activeCellStatusList [counter1*12+6] = 1;

                            }

**else**{

                                randInit = rand() % (**int**)(round(simProcessDataMiddleHold [25]*(endVariationtHold/(**double**)100))) + 0;

**if** (randInit%2 == 0) activeCellStatusList [counter1*12+4] = (**int**)simProcessDataMiddleHold [25]+randInit;

**else** activeCellStatusList [counter1*12+4] = (**int**)simProcessDataMiddleHold [25]-randInit;

                                activeCellStatusList [counter1*12+5] = 5;

                                activeCellStatusList [counter1*12+6] = 1;

                            }

                        }

                    }

**else** **if** (activeCellStatusList [counter1*12+5] == 2){

                        siblinFusionCheck1 = 0;

                        siblinFusionCheck2 = 0;

                        siblinFusionDoub1 = 0;

                        siblinFusionDoub2 = 0;

                        siblingCellNo1 = 0;

                        siblingCellNo2 = 0;

**for** (**int** counter2 = 0; counter2 < activeCellStatusListCount/12; counter2++){

**if** (activeCellStatusList [counter2*12+6] == 1 && (activeCellStatusList [counter2*12+1] == activeCellStatusList [counter1*12] || activeCellStatusList [counter2*12+2] == activeCellStatusList [counter1*12]) && activeCellStatusList [counter2*12+3] == activeCellStatusList [counter1*12+3]){

**if** (siblinFusionCheck1 == 0 && activeCellStatusList [counter2*12+5] != 0){

                                    siblinFusionCheck1 = activeCellStatusList [counter2*12+5];

                                    siblinFusionDoub1 = activeCellStatusList [counter2*12+4];

                                    siblingCellNo1 = counter2;

                                }

**else** **if** (siblinFusionCheck1 != 0 && siblinFusionCheck2 == 0 && activeCellStatusList [counter2*12+5] != 0){

                                    siblinFusionCheck2 = activeCellStatusList [counter2*12+5];

                                    siblinFusionDoub2 = activeCellStatusList [counter2*12+4];

                                    siblingCellNo2 = counter2;

**break**;

                                }

                            }

                        }

                        eventType = 0;

**if** (siblinFusionCheck1 == 6 && siblinFusionCheck2 == 6){

**for** (**int** counter2 = 0; counter2 < 100; counter2++){

                                randInit = rand() % 100 + 0;

**if** (secondEventTDList [randInit] != 10){

                                    eventType = secondEventTDList [randInit];

                                    loopCheck = 1;

**break**;

                                }

                            }

**if** (loopCheck == 0) eventType = 13;

                        }

**else**{

                            randInit = rand() % 100 + 0;

                            eventType = secondEventTDList [randInit];

**if** (eventType == 0) eventType = 13;

                        }

**if** ((activeCellStatusList [counter1*12+8] == 2 || activeCellStatusList [counter1*12+8] == 3) && activeCellStatusList [counter1*12+8] == activeCellStatusList [counter1*12+7] && eventType == 12){

                            eventType = 13;

                        }

**if** (siblinFusionDoub1 < siblinFusionDoub2) siblinFusionDoubLarge = siblinFusionDoub2;

**else** siblinFusionDoubLarge = siblinFusionDoub1;

**if** (siblinFusionCheck1 == 6 && siblinFusionCheck2 == 6 && eventType == 10){

                            eventType = 13;

                        }

**if** (eventType == 10){

                            loopCheck = 0;

**if** (siblinFusionCheck1 != 0 && siblinFusionCheck2 != 0){

**if** (siblinFusionCheck1 == 6 && siblinFusionCheck2 != 6){

**if** (expandTDCFCount == 0){

                                        randInit = rand() % randCDRangeB + randCDRangeA;

**if** (siblinFusionDoub2 < randBDRangeA) siblinFusionDoub2 = randBDRangeA;

**if** (siblinFusionDoub1 > siblinFusionDoub2){

**if** (randInit > siblinFusionDoub1){

                                                activeCellStatusList [siblingCellNo2*12+4] = randInit+10;

                                                activeCellStatusList [counter1*12+4] = siblinFusionDoub2;

                                                activeCellStatusList [counter1*12+5] = 6;

                                                activeCellStatusList [counter1*12+6] = 1;

                                            }

**else**{

                                                activeCellStatusList [siblingCellNo2*12+4] = siblinFusionDoub1+10;

                                                activeCellStatusList [counter1*12+4] = siblinFusionDoub2;

                                                activeCellStatusList [counter1*12+5] = 6;

                                                activeCellStatusList [counter1*12+6] = 1;

                                            }

                                        }

**else** **if** (siblinFusionDoub1 < siblinFusionDoub2){

**if** (randInit > siblinFusionDoub2){

                                                activeCellStatusList [siblingCellNo2*12+4] = randInit+10;

                                                activeCellStatusList [counter1*12+4] = siblinFusionDoub2;

                                                activeCellStatusList [counter1*12+5] = 6;

                                                activeCellStatusList [counter1*12+6] = 1;

                                            }

**else**{

                                                activeCellStatusList [siblingCellNo2*12+4] = siblinFusionDoub2+10;

                                                activeCellStatusList [counter1*12+4] = siblinFusionDoub2;

                                                activeCellStatusList [counter1*12+5] = 6;

                                                activeCellStatusList [counter1*12+6] = 1;

                                            }

                                        }

                                    }

**else**{

**if** (siblinFusionDoub1 > siblinFusionDoub2){

**if** (expandTDCFCount != 0){

                                                randInit = rand() % expandTDCFCount/2 + 0;

**if** (expandTDCF [randInit*2] > siblinFusionDoub1){

                                                    activeCellStatusList [siblingCellNo2*12+4] = expandTDCF [randInit*2]+10;

                                                    activeCellStatusList [counter1*12+4] = siblinFusionDoub2;

                                                    activeCellStatusList [counter1*12+5] = 6;

                                                    activeCellStatusList [counter1*12+6] = 1;

                                                }

**else**{

                                                    activeCellStatusList [siblingCellNo2*12+4] = siblinFusionDoub1+10;

                                                    activeCellStatusList [counter1*12+4] = siblinFusionDoub2;

                                                    activeCellStatusList [counter1*12+5] = 6;

                                                    activeCellStatusList [counter1*12+6] = 1;

                                                }

                                            }

**else**{

                                                activeCellStatusList [siblingCellNo2*12+4] = siblinFusionDoub1+10;

                                                activeCellStatusList [counter1*12+4] = siblinFusionDoub2;

                                                activeCellStatusList [counter1*12+5] = 6;

                                                activeCellStatusList [counter1*12+6] = 1;

                                            }

                                        }

**else** **if** (siblinFusionDoub1 < siblinFusionDoub2){

**if** (expandTDCFCount != 0){

                                                randInit = rand() % expandTDCFCount/2 + 0;

**if** (expandTDCF [randInit*2] > siblinFusionDoub2){

                                                    activeCellStatusList [siblingCellNo2*12+4] = expandTDCF [randInit*2]+10;

                                                    activeCellStatusList [counter1*12+4] = siblinFusionDoub2;

                                                    activeCellStatusList [counter1*12+5] = 6;

                                                    activeCellStatusList [counter1*12+6] = 1;

                                                }

**else**{

                                                    activeCellStatusList [siblingCellNo2*12+4] = siblinFusionDoub2+10;

                                                    activeCellStatusList [counter1*12+4] = siblinFusionDoub2;

                                                    activeCellStatusList [counter1*12+5] = 6;

                                                    activeCellStatusList [counter1*12+6] = 1;

                                                }

                                            }

**else**{

                                                activeCellStatusList [siblingCellNo2*12+4] = siblinFusionDoub2+10;

                                                activeCellStatusList [counter1*12+4] = siblinFusionDoub2;

                                                activeCellStatusList [counter1*12+5] = 6;

                                                activeCellStatusList [counter1*12+6] = 1;

                                            }

                                        }

                                    }

                                }

**else** **if** (siblinFusionCheck1 != 6 && siblinFusionCheck2 == 6){

**if** (expandTDCFCount == 0){

                                        randInit = rand() % randCDRangeB + randCDRangeA;

**if** (siblinFusionDoub1 > siblinFusionDoub2){

**if** (randInit > siblinFusionDoub1){

                                                activeCellStatusList [siblingCellNo1*12+4] = randInit+10;

                                                activeCellStatusList [counter1*12+4] = siblinFusionDoub1;

                                                activeCellStatusList [counter1*12+5] = 6;

                                                activeCellStatusList [counter1*12+6] = 1;

                                            }

**else**{

                                                activeCellStatusList [siblingCellNo1*12+4] = siblinFusionDoub1+10;

                                                activeCellStatusList [counter1*12+4] = siblinFusionDoub1;

                                                activeCellStatusList [counter1*12+5] = 6;

                                                activeCellStatusList [counter1*12+6] = 1;

                                            }

                                        }

**else** **if** (siblinFusionDoub1 < siblinFusionDoub2){

**if** (randInit > siblinFusionDoub2){

                                                activeCellStatusList [siblingCellNo1*12+4] = randInit+10;

                                                activeCellStatusList [counter1*12+4] = siblinFusionDoub1;

                                                activeCellStatusList [counter1*12+5] = 6;

                                                activeCellStatusList [counter1*12+6] = 1;

                                            }

**else**{

                                                activeCellStatusList [siblingCellNo1*12+4] = siblinFusionDoub2+10;

                                                activeCellStatusList [counter1*12+4] = siblinFusionDoub1;

                                                activeCellStatusList [counter1*12+5] = 6;

                                                activeCellStatusList [counter1*12+6] = 1;

                                            }

                                        }

                                    }

**else**{

**if** (siblinFusionDoub1 > siblinFusionDoub2){

**if** (expandTDCFCount != 0){

                                                randInit = rand() % expandTDCFCount/2 + 0;

**if** (expandTDCF [randInit*2] > siblinFusionDoub1){

                                                    activeCellStatusList [siblingCellNo1*12+4] = expandTDCF [randInit*2]+10;

                                                    activeCellStatusList [counter1*12+4] = siblinFusionDoub1;

                                                    activeCellStatusList [counter1*12+5] = 6;

                                                    activeCellStatusList [counter1*12+6] = 1;

                                                }

**else**{

                                                    activeCellStatusList [siblingCellNo1*12+4] = siblinFusionDoub1+10;

                                                    activeCellStatusList [counter1*12+4] = siblinFusionDoub1;

                                                    activeCellStatusList [counter1*12+5] = 6;

                                                    activeCellStatusList [counter1*12+6] = 1;

                                                }

                                            }

**else**{

                                                activeCellStatusList [siblingCellNo1*12+4] = siblinFusionDoub1+10;

                                                activeCellStatusList [counter1*12+4] = siblinFusionDoub1;

                                                activeCellStatusList [counter1*12+5] = 6;

                                                activeCellStatusList [counter1*12+6] = 1;

                                            }

                                        }

**else** **if** (siblinFusionDoub1 < siblinFusionDoub2){

**if** (expandTDCFCount != 0){

                                                randInit = rand() % expandTDCFCount/2 + 0;

**if** (expandTDCF [randInit*2] >= siblinFusionDoub2){

                                                    activeCellStatusList [siblingCellNo1*12+4] = expandTDCF [randInit*2]+10;

                                                    activeCellStatusList [counter1*12+4] = siblinFusionDoub1;

                                                    activeCellStatusList [counter1*12+5] = 6;

                                                    activeCellStatusList [counter1*12+6] = 1;

                                                }

**else**{

                                                    activeCellStatusList [siblingCellNo1*12+4] = siblinFusionDoub2+10;

                                                    activeCellStatusList [counter1*12+4] = siblinFusionDoub1;

                                                    activeCellStatusList [counter1*12+5] = 6;

                                                    activeCellStatusList [counter1*12+6] = 1;

                                                }

                                            }

**else**{

                                                activeCellStatusList [siblingCellNo1*12+4] = siblinFusionDoub2+10;

                                                activeCellStatusList [counter1*12+4] = siblinFusionDoub1;

                                                activeCellStatusList [counter1*12+5] = 6;

                                                activeCellStatusList [counter1*12+6] = 1;

                                            }

                                        }

                                    }

                                }

**else** **if** (siblinFusionCheck1 != 6 && siblinFusionCheck2 != 6){

                                    activeCellStatusList [siblingCellNo1*12+4] = siblinFusionDoub1+10;

                                    activeCellStatusList [siblingCellNo2*12+4] = siblinFusionDoub2+10;

                                    activeCellStatusList [counter1*12+4] = siblinFusionDoub2;

                                    activeCellStatusList [counter1*12+5] = 6;

                                    activeCellStatusList [counter1*12+6] = 1;

                                }

                            }

**else** **if** (siblinFusionCheck1 != 0 && siblinFusionCheck2 == 0){

**if** (expandTDCFCount == 0){

                                    randInit = rand() % randCDRangeB + randCDRangeA;

**if** (randInit > siblinFusionDoub1+10){

                                        activeCellStatusList [counter1*12+4] = randInit;

                                        activeCellStatusList [counter1*12+5] = 6;

                                        activeCellStatusList [counter1*12+6] = 1;

                                    }

**else**{

                                        activeCellStatusList [counter1*12+4] = siblinFusionDoub1+5;

                                        activeCellStatusList [counter1*12+5] = 6;

                                        activeCellStatusList [counter1*12+6] = 1;

                                    }

                                }

**else**{

**if** (expandTDCFCount != 0){

                                        randInit = rand() % expandTDCFCount/2 + 0;

**if** (expandTDCF [randInit*2] > siblinFusionDoub1+10){

                                            activeCellStatusList [counter1*12+4] = expandTDCF [randInit*2];

                                            activeCellStatusList [counter1*12+5] = 6;

                                            activeCellStatusList [counter1*12+6] = 1;

                                        }

**else**{

                                            activeCellStatusList [counter1*12+4] = siblinFusionDoub1+5;

                                            activeCellStatusList [counter1*12+5] = 6;

                                            activeCellStatusList [counter1*12+6] = 1;

                                        }

                                    }

**else**{

                                        activeCellStatusList [counter1*12+4] = siblinFusionDoub1+5;

                                        activeCellStatusList [counter1*12+5] = 6;

                                        activeCellStatusList [counter1*12+6] = 1;

                                    }

                                }

                            }

**else** **if** (siblinFusionCheck1 == 0 && siblinFusionCheck2 == 0){

**if** (expandTDCFCount == 0){

                                    randInit = rand() % randCDRangeB + randCDRangeA;

                                    activeCellStatusList [counter1*12+4] = randInit;

                                    activeCellStatusList [counter1*12+5] = 6;

                                    activeCellStatusList [counter1*12+6] = 1;

                                }

**else**{

**if** (activeCellStatusList [counter1*12+11] == 0){

                                        randInit = rand() % expandTDCFCount/2 + 0;

                                        activeCellStatusList [counter1*12+4] = expandTDCF [randInit*2];

                                        activeCellStatusList [counter1*12+5] = 6;

                                        activeCellStatusList [counter1*12+6] = 1;

                                    }

**else**{

                                        randInit = rand() % expandTDCFCount/2 + 0;

                                        activeCellStatusList [counter1*12+4] = expandTDCF [randInit*2];

                                        activeCellStatusList [counter1*12+5] = 6;

                                        activeCellStatusList [counter1*12+6] = 1;

                                    }

                                }

                            }

                        }

**else** **if** (eventType == 11){

**if** (expandDoublingDoubTDCount == 0){

                                randInit = rand() % randBDRangeB + randBDRangeA;

**if** (randInit > siblinFusionDoubLarge+10){

                                    activeCellStatusList [counter1*12+4] = randInit;

                                    activeCellStatusList [counter1*12+5] = 1;

                                    activeCellStatusList [counter1*12+6] = 1;

                                    activeCellStatusList [counter1*12+9] = (**int**)simProcessDataMiddleHold [19];

                                    activeCellStatusList [counter1*12+7] = 0;

                                }

**else**{

**if** (siblinFusionDoubLarge < randBDRangeA) siblinFusionDoubLarge = randBDRangeA;

                                    activeCellStatusList [counter1*12+4] = siblinFusionDoubLarge+10;

                                    activeCellStatusList [counter1*12+5] = 1;

                                    activeCellStatusList [counter1*12+6] = 1;

                                    activeCellStatusList [counter1*12+9] = (**int**)simProcessDataMiddleHold [19];

                                    activeCellStatusList [counter1*12+7] = 0;

                                }

                            }

**else**{

**if** (activeCellStatusList [counter1*12+11] == 0){

**if** (expandDoublingDoubTDCount != 0){

                                        randInit = rand() % expandDoublingDoubTDCount/2 + 0;

**if** (randInit > siblinFusionDoubLarge+10){

                                            activeCellStatusList [counter1*12+4] = expandDoublingDoubTD [randInit*2];

                                            activeCellStatusList [counter1*12+5] = 1;

                                            activeCellStatusList [counter1*12+6] = 1;

                                            activeCellStatusList [counter1*12+9] = (**int**)simProcessDataMiddleHold [19];

                                            activeCellStatusList [counter1*12+7] = 0;

                                        }

**else**{

**if** (siblinFusionDoubLarge < randBDRangeA) siblinFusionDoubLarge = randBDRangeA;

                                            activeCellStatusList [counter1*12+4] = siblinFusionDoubLarge+10;

                                            activeCellStatusList [counter1*12+5] = 1;

                                            activeCellStatusList [counter1*12+6] = 1;

                                            activeCellStatusList [counter1*12+9] = (**int**)simProcessDataMiddleHold [19];

                                            activeCellStatusList [counter1*12+7] = 0;

                                        }

                                    }

**else**{

**if** (siblinFusionDoubLarge < randBDRangeA) siblinFusionDoubLarge = randBDRangeA;

                                        activeCellStatusList [counter1*12+4] = siblinFusionDoubLarge+10;

                                        activeCellStatusList [counter1*12+5] = 1;

                                        activeCellStatusList [counter1*12+6] = 1;

                                        activeCellStatusList [counter1*12+9] = (**int**)simProcessDataMiddleHold [19];

                                        activeCellStatusList [counter1*12+7] = 0;

                                    }

                                }

**else**{

**if** (expandDoublingDoubTDCount != 0){

                                        randInit = rand() % expandDoublingDoubTDCount/2 + 0;

**if** (expandDoublingDoubTD [randInit*2] > siblinFusionDoubLarge+10){

                                            activeCellStatusList [counter1*12+4] = expandDoublingDoubTD [randInit*2];

                                            activeCellStatusList [counter1*12+5] = 1;

                                            activeCellStatusList [counter1*12+6] = 1;

                                            activeCellStatusList [counter1*12+9] = (**int**)simProcessDataMiddleHold [19];

                                            activeCellStatusList [counter1*12+7] = 0;

                                        }

**else**{

**if** (siblinFusionDoubLarge < randBDRangeA) siblinFusionDoubLarge = randBDRangeA;

                                            activeCellStatusList [counter1*12+4] = siblinFusionDoubLarge+10;

                                            activeCellStatusList [counter1*12+5] = 1;

                                            activeCellStatusList [counter1*12+6] = 1;

                                            activeCellStatusList [counter1*12+9] = (**int**)simProcessDataMiddleHold [19];

                                            activeCellStatusList [counter1*12+7] = 0;

                                        }

                                    }

**else**{

**if** (siblinFusionDoubLarge < randBDRangeA) siblinFusionDoubLarge = randBDRangeA;

                                        activeCellStatusList [counter1*12+4] = siblinFusionDoubLarge+10;

                                        activeCellStatusList [counter1*12+5] = 1;

                                        activeCellStatusList [counter1*12+6] = 1;

                                        activeCellStatusList [counter1*12+9] = (**int**)simProcessDataMiddleHold [19];

                                        activeCellStatusList [counter1*12+7] = 0;

                                    }

                                }

                            }

                        }

**else** **if** (eventType == 12){

**if** (expandDoublingDoubTDCount == 0){

                                randInit = rand() % randBDRangeB + randBDRangeA;

**if** (randInit > siblinFusionDoubLarge+10){

                                    activeCellStatusList [counter1*12+4] = randInit;

                                    activeCellStatusList [counter1*12+5] = 2;

                                    activeCellStatusList [counter1*12+6] = 1;

                                    activeCellStatusList [counter1*12+7]++;

                                }

**else**{

**if** (siblinFusionDoubLarge < randBDRangeA) siblinFusionDoubLarge = randBDRangeA;

                                    activeCellStatusList [counter1*12+4] = siblinFusionDoubLarge+10;

                                    activeCellStatusList [counter1*12+5] = 2;

                                    activeCellStatusList [counter1*12+6] = 1;

                                    activeCellStatusList [counter1*12+7]++;

                                }

                            }

**else**{

**if** (activeCellStatusList [counter1*12+11] == 0){

**if** (expandDoublingDoubTDCount != 0){

                                        randInit = rand() % expandDoublingDoubTDCount/2 + 0;

**if** (randInit >= siblinFusionDoubLarge+10){

                                            activeCellStatusList [counter1*12+4] = expandDoublingDoubTD [randInit*2];

                                            activeCellStatusList [counter1*12+5] = 2;

                                            activeCellStatusList [counter1*12+6] = 1;

                                            activeCellStatusList [counter1*12+7]++;

                                        }

**else**{

**if** (siblinFusionDoubLarge < randBDRangeA) siblinFusionDoubLarge = randBDRangeA;

                                            activeCellStatusList [counter1*12+4] = siblinFusionDoubLarge+10;

                                            activeCellStatusList [counter1*12+5] = 2;

                                            activeCellStatusList [counter1*12+6] = 1;

                                            activeCellStatusList [counter1*12+7]++;

                                        }

                                    }

**else**{

**if** (siblinFusionDoubLarge < randBDRangeA) siblinFusionDoubLarge = randBDRangeA;

                                        activeCellStatusList [counter1*12+4] = siblinFusionDoubLarge+10;

                                        activeCellStatusList [counter1*12+5] = 2;

                                        activeCellStatusList [counter1*12+6] = 1;

                                        activeCellStatusList [counter1*12+7]++;

                                    }

                                }

**else**{

**if** (expandDoublingDoubTDCount != 0){

                                        randInit = rand() % expandDoublingDoubTDCount/2 + 0;

**if** (expandDoublingDoubTD [randInit*2] > siblinFusionDoubLarge+10){

                                            activeCellStatusList [counter1*12+4] = expandDoublingDoubTD [randInit*2];

                                            activeCellStatusList [counter1*12+5] = 2;

                                            activeCellStatusList [counter1*12+7]++;

                                        }

**else**{

**if** (siblinFusionDoubLarge < randBDRangeA) siblinFusionDoubLarge = randBDRangeA;

                                            activeCellStatusList [counter1*12+4] = siblinFusionDoubLarge+10;

                                            activeCellStatusList [counter1*12+5] = 2;

                                            activeCellStatusList [counter1*12+7]++;

                                        }

                                    }

**else**{

**if** (siblinFusionDoubLarge < randBDRangeA) siblinFusionDoubLarge = randBDRangeA;

                                        activeCellStatusList [counter1*12+4] = siblinFusionDoubLarge+10;

                                        activeCellStatusList [counter1*12+5] = 2;

                                        activeCellStatusList [counter1*12+7]++;

                                    }

                                }

                            }

                        }

**else** **if** (eventType == 13){

**if** (expandTDCDCount == 0){

                                randInit = rand() % randCDRangeB + randCDRangeA;

**if** (randInit > siblinFusionDoubLarge+10){

                                    activeCellStatusList [counter1*12+4] = randInit;

                                    activeCellStatusList [counter1*12+5] = 3;

                                    activeCellStatusList [counter1*12+6] = 1;

                                }

**else**{

                                    activeCellStatusList [counter1*12+4] = siblinFusionDoubLarge+10;

                                    activeCellStatusList [counter1*12+5] = 3;

                                    activeCellStatusList [counter1*12+6] = 1;

                                }

                            }

**else**{

**if** (expandTDCDCount != 0){

                                    randInit = rand() % expandTDCDCount/2 + 0;

**if** (expandTDCD [randInit*2] > siblinFusionDoubLarge+10){

                                        activeCellStatusList [counter1*12+4] = expandTDCD [randInit*2];

                                        activeCellStatusList [counter1*12+5] = 3;

                                        activeCellStatusList [counter1*12+6] = 1;

                                    }

**else**{

                                        activeCellStatusList [counter1*12+4] = siblinFusionDoubLarge+10;

                                        activeCellStatusList [counter1*12+5] = 3;

                                        activeCellStatusList [counter1*12+6] = 1;

                                    }

                                }

**else**{

                                    activeCellStatusList [counter1*12+4] = siblinFusionDoubLarge+10;

                                    activeCellStatusList [counter1*12+5] = 3;

                                    activeCellStatusList [counter1*12+6] = 1;

                                }

                            }

                        }

                    }

                }

**else**{

**if** (activeCellStatusList [counter1*12+5] == 5){

                        dataTempDouble = ((expandNonCDSelCount/(**double**)2)/(**double**)simProcessDataAddHold [24])*100;

                        randInit = rand() % 100 + 0;

**if** (randInit < dataTempDouble && expandNonCDSelCount != 0){

                            randInit = rand() % expandNonCDSelCount/2 + 0;

                            activeCellStatusList [counter1*12+4] = expandNonCDSel [randInit*2];

                            activeCellStatusList [counter1*12+5] = 30;

                            activeCellStatusList [counter1*12+6] = 1;

                        }

**else**{

                            randInit = rand() % (**int**)(round(simProcessDataAddHold [25]*(endVariationtHold/(**double**)100))) + 0;

**if** (randInit%2 == 0) activeCellStatusList [counter1*12+4] = (**int**)simProcessDataAddHold [25]+randInit;

**else** activeCellStatusList [counter1*12+4] = (**int**)simProcessDataAddHold [25]-randInit;

                            activeCellStatusList [counter1*12+5] = 5;

                            activeCellStatusList [counter1*12+6] = 1;

                        }

                    }

**else** **if** (activeCellStatusList [counter1*12+5] == 7){

                        eventType = 1;

**if** (eventType == 1){

**if** (activeCellStatusList [counter1*12+11] == 0){

**if** (expandDoublingDoubBDSelCount != 0){

                                    randInit = rand() % expandDoublingDoubBDSelCount/2 + 0;

                                    activeCellStatusList [counter1*12+4] = expandDoublingDoubBDSel [randInit*2];

                                    activeCellStatusList [counter1*12+5] = 1;

                                    activeCellStatusList [counter1*12+6] = 1;

                                }

**else**{

                                    randInit = rand() % randBDRangeSelB + randBDRangeSelA;

                                    activeCellStatusList [counter1*12+4] = randInit;

                                    activeCellStatusList [counter1*12+5] = 7;

                                    activeCellStatusList [counter1*12+6] = 1;

                                }

                            }

**else**{

                                loopCheck = 0;

**for** (**int** counter2 = 0; counter2 < 100; counter2++){

**if** (expandDoublingDoubBDSelCount != 0){

                                        randInit = rand() % expandDoublingDoubBDSelCount/2 + 0;

**if** (expandDoublingDoubBDSel [randInit*2] > activeCellStatusList [counter1*12+11]-50 && expandDoublingDoubBDSel [randInit*2] < activeCellStatusList [counter1*12+11]+50){

                                            activeCellStatusList [counter1*12+4] = expandDoublingDoubBDSel [randInit*2];

                                            activeCellStatusList [counter1*12+5] = 1;

                                            activeCellStatusList [counter1*12+6] = 1;

                                            loopCheck = 1;

**break**;

                                        }

                                    }

                                }

**if** (loopCheck == 0){

                                    activeCellStatusList [counter1*12+4] = activeCellStatusList [counter1*12+11];

                                    activeCellStatusList [counter1*12+5] = 7;

                                    activeCellStatusList [counter1*12+6] = 1;

                                }

                            }

                        }

                    }

**else** **if** (activeCellStatusList [counter1*12+5] == 1){

                        siblinFusionCheck1 = 0;

                        siblinFusionDoub1 = 0;

                        siblingCellNo1 = 0;

**for** (**int** counter2 = 0; counter2 < activeCellStatusListCount/12; counter2++){

**if** (activeCellStatusList [counter2*12+6] == 1 && activeCellStatusList [counter2*12+1] == activeCellStatusList [counter1*12] && activeCellStatusList [counter2*12+3] == activeCellStatusList [counter1*12+3]){

                                siblingCellNo1 = counter2;

**if** (activeCellStatusList [counter2*12+5] != 0){

                                    siblinFusionCheck1 = activeCellStatusList [counter2*12+5];

                                    siblinFusionDoub1 = activeCellStatusList [counter2*12+4];

                                }

**break**;

                            }

                        }

                        eventType = 0;

**if** (activeCellStatusList [counter1*12+9] == 0){

**if** (siblinFusionCheck1 == 6){

                                loopCheck = 0;

**for** (**int** counter2 = 0; counter2 < 100; counter2++){

                                    randInit = rand() % 100 + 0;

**if** (secondEventBDListSel [randInit] != 6){

                                        eventType = secondEventBDListSel [randInit];

                                        loopCheck = 1;

**break**;

                                    }

                                }

**if** (loopCheck == 0) eventType = 1;

                            }

**else**{

                                randInit = rand() % 100 + 0;

**if** (secondEventBDListSel [randInit] != 0) eventType = secondEventBDListSel [randInit];

**else** eventType = 1;

                            }

                        }

**else** **if** (activeCellStatusList [counter1*12+9] != 0){

                            eventCount = 0;

                            eventType = 0;

**if** (siblinFusionCheck1 == 6) eventType = 5;

**else**{

**for** (**int** counter2 = 0; counter2 < 5; counter2++){

                                    randInit = rand() % 100 + 0;

**if** (secondEventBDListSel [randInit] == 1) eventCount++;

**else**{

                                        eventType = secondEventBDListSel [randInit];

**break**;

                                    }

                                }

**if** (eventCount == 5 || eventType == 0) eventType = 1;

                            }

                        }

**if** (eventType == 1){

**if** (activeCellStatusList [counter1*12+11] == 0){

**if** (siblinFusionCheck1 != 6){

**if** (expandDoublingDoubBDSelCount != 0){

                                        randInit = rand() % expandDoublingDoubBDSelCount/2 + 0;

                                        activeCellStatusList [counter1*12+4] = expandDoublingDoubBDSel [randInit*2];

                                        activeCellStatusList [counter1*12+5] = 1;

                                        activeCellStatusList [counter1*12+6] = 1;

**if** (activeCellStatusList [counter1*12+9] != 0) activeCellStatusList [counter1*12+9]--;

                                    }

**else**{

                                        randInit = rand() % randBDRangeSelB + randBDRangeSelA;

                                        activeCellStatusList [counter1*12+4] = randInit;

                                        activeCellStatusList [counter1*12+5] = 1;

                                        activeCellStatusList [counter1*12+6] = 1;

**if** (activeCellStatusList [counter1*12+9] != 0) activeCellStatusList [counter1*12+9]--;

                                    }

                                }

**else**{

**if** (siblinFusionDoub1 < randBDRangeSelA) siblinFusionDoub1 = randBDRangeSelA;

                                    activeCellStatusList [counter1*12+4] = siblinFusionDoub1+10;

                                    activeCellStatusList [counter1*12+5] = 1;

                                    activeCellStatusList [counter1*12+6] = 1;

**if** (activeCellStatusList [counter1*12+9] != 0) activeCellStatusList [counter1*12+9]--;

                                }

                            }

**else**{

                                loopCheck = 0;

**for** (**int** counter2 = 0; counter2 < 100; counter2++){

**if** (siblinFusionCheck1 != 6){

**if** (expandDoublingDoubBDSelCount != 0){

                                            randInit = rand() % expandDoublingDoubBDSelCount/2 + 0;

**if** (expandDoublingDoubBDSel [randInit*2] > activeCellStatusList [counter1*12+11]-50 && expandDoublingDoubBDSel [randInit*2] < activeCellStatusList [counter1*12+11]+50){

                                                activeCellStatusList [counter1*12+4] = expandDoublingDoubBDSel [randInit*2];

                                                activeCellStatusList [counter1*12+5] = 1;

                                                activeCellStatusList [counter1*12+6] = 1;

**if** (activeCellStatusList [counter1*12+9] != 0) activeCellStatusList [counter1*12+9]--;

                                                loopCheck = 1;

**break**;

                                            }

                                        }

                                    }

**else** **if** (siblinFusionCheck1 == 6){

**if** (expandDoublingDoubBDSelCount != 0){

                                            randInit = rand() % expandDoublingDoubBDSelCount/2 + 0;

**if** (expandDoublingDoubBDSel [randInit*2] > activeCellStatusList [counter1*12+11]-50 && expandDoublingDoubBDSel [randInit*2] < activeCellStatusList [counter1*12+11]+50 && siblinFusionDoub1+10 < expandDoublingDoubBDSel [randInit*2]){

                                                activeCellStatusList [counter1*12+4] = expandDoublingDoubBDSel [randInit*2];

                                                activeCellStatusList [counter1*12+5] = 1;

                                                activeCellStatusList [counter1*12+6] = 1;

**if** (activeCellStatusList [counter1*12+9] != 0) activeCellStatusList [counter1*12+9]--;

                                                loopCheck = 1;

**break**;

                                            }

                                        }

                                    }

                                }

**if** (loopCheck == 0){

                                    lowextValue = 100000;

                                    highestValue = 0;

**for** (**int** counter2 = 0; counter2 < expandDoublingDoubBDSelCount/2; counter2++){

**if** (lowextValue > expandDoublingDoubBDSel [counter2*2]) lowextValue = expandDoublingDoubBDSel [counter2*2];

**if** (highestValue < expandDoublingDoubBDSel [counter2*2]) highestValue = expandDoublingDoubBDSel [counter2*2];

                                    }

**if** (siblinFusionDoub1 < randBDRangeSelA) siblinFusionDoub1 = randBDRangeSelA;

**if** (lowextValue != 100000 && highestValue != 0){

**if** ((highestValue+lowextValue)/(**double**)2 > siblinFusionDoub1+10){

                                            activeCellStatusList [counter1*12+4] = (**int**)(round((highestValue+lowextValue)/(**double**)2));

                                            activeCellStatusList [counter1*12+5] = 1;

                                            activeCellStatusList [counter1*12+6] = 1;

**if** (activeCellStatusList [counter1*12+9] != 0) activeCellStatusList [counter1*12+9]--;

                                        }

**else**{

                                            activeCellStatusList [counter1*12+4] = siblinFusionDoub1+10;

                                            activeCellStatusList [counter1*12+5] = 1;

                                            activeCellStatusList [counter1*12+6] = 1;

**if** (activeCellStatusList [counter1*12+9] != 0) activeCellStatusList [counter1*12+9]--;

                                        }

                                    }

**else**{

                                        activeCellStatusList [counter1*12+4] = siblinFusionDoub1+10;

                                        activeCellStatusList [counter1*12+5] = 1;

                                        activeCellStatusList [counter1*12+6] = 1;

**if** (activeCellStatusList [counter1*12+9] != 0) activeCellStatusList [counter1*12+9]--;

                                    }

                                }

                            }

                        }

**else** **if** (eventType == 2){

**if** (activeCellStatusList [counter1*12+11] == 0){

**if** (siblinFusionCheck1 != 6){

**if** (expandDoublingDoubBDSelCount != 0){

                                        randInit = rand() % expandDoublingDoubBDSelCount/2 + 0;

                                        activeCellStatusList [counter1*12+4] = expandDoublingDoubBDSel [randInit*2];

                                        activeCellStatusList [counter1*12+5] = 2;

                                        activeCellStatusList [counter1*12+6] = 1;

                                        activeCellStatusList [counter1*12+7] = 1;

                                        activeCellStatusList [counter1*12+9] = 0;

                                        activeCellStatusList [counter1*12+8] = (**int**)simProcessDataAddHold [18];

                                    }

**else**{

                                        randInit = rand() % randBDRangeSelB + randBDRangeSelA;

                                        activeCellStatusList [counter1*12+4] = randInit;

                                        activeCellStatusList [counter1*12+5] = 2;

                                        activeCellStatusList [counter1*12+6] = 1;

                                        activeCellStatusList [counter1*12+7] = 1;

                                        activeCellStatusList [counter1*12+9] = 0;

                                        activeCellStatusList [counter1*12+8] = (**int**)simProcessDataAddHold [18];

                                    }

                                }

**else**{

**if** (siblinFusionDoub1 < randBDRangeSelA) siblinFusionDoub1 = randBDRangeSelA;

                                    activeCellStatusList [counter1*12+4] = siblinFusionDoub1+10;

                                    activeCellStatusList [counter1*12+5] = 2;

                                    activeCellStatusList [counter1*12+6] = 1;

                                    activeCellStatusList [counter1*12+7] = 1;

                                    activeCellStatusList [counter1*12+9] = 0;

                                    activeCellStatusList [counter1*12+8] = (**int**)simProcessDataAddHold [18];

                                }

                            }

**else**{

                                loopCheck = 0;

**for** (**int** counter2 = 0; counter2 < 100; counter2++){

**if** (siblinFusionCheck1 != 6){

**if** (expandDoublingDoubBDSelCount != 0){

                                            randInit = rand() % expandDoublingDoubBDSelCount/2 + 0;

**if** (expandDoublingDoubBDSel [randInit*2] > activeCellStatusList [counter1*12+11]-50 && expandDoublingDoubBDSel [randInit*2] < activeCellStatusList [counter1*12+11]+50){

                                                activeCellStatusList [counter1*12+4] = expandDoublingDoubBDSel [randInit*2];

                                                activeCellStatusList [counter1*12+5] = 2;

                                                activeCellStatusList [counter1*12+6] = 1;

                                                activeCellStatusList [counter1*12+7] = 1;

                                                activeCellStatusList [counter1*12+9] = 0;

                                                activeCellStatusList [counter1*12+8] = (**int**)simProcessDataAddHold [18];

                                                loopCheck = 1;

**break**;

                                            }

                                        }

                                    }

**else** **if** (siblinFusionCheck1 == 6){

**if** (expandDoublingDoubBDSelCount != 0){

                                            randInit = rand() % expandDoublingDoubBDSelCount/2 + 0;

**if** (expandDoublingDoubBDSel [randInit*2] > activeCellStatusList [counter1*12+11]-50 && expandDoublingDoubBDSel [randInit*2] < activeCellStatusList [counter1*12+11]+50 && siblinFusionDoub1+10 < expandDoublingDoubBDSel [randInit*2]){

                                                activeCellStatusList [counter1*12+4] = expandDoublingDoubBDSel [randInit*2];

                                                activeCellStatusList [counter1*12+5] = 2;

                                                activeCellStatusList [counter1*12+6] = 1;

                                                activeCellStatusList [counter1*12+7] = 1;

                                                activeCellStatusList [counter1*12+9] = 0;

                                                activeCellStatusList [counter1*12+8] = (**int**)simProcessDataAddHold [18];

                                                loopCheck = 1;

**break**;

                                            }

                                        }

                                    }

                                }

**if** (loopCheck == 0){

                                    lowextValue = 100000;

                                    highestValue = 0;

**for** (**int** counter2 = 0; counter2 < expandDoublingDoubBDSelCount/2; counter2++){

**if** (lowextValue > expandDoublingDoubBDSel [counter2*2]) lowextValue = expandDoublingDoubBDSel [counter2*2];

**if** (highestValue < expandDoublingDoubBDSel [counter2*2]) highestValue = expandDoublingDoubBDSel [counter2*2];

                                    }

**if** (siblinFusionDoub1 < randBDRangeSelA) siblinFusionDoub1 = randBDRangeSelA;

**if** (lowextValue != 100000 && highestValue != 0){

**if** ((highestValue+lowextValue)/(**double**)2 > siblinFusionDoub1+10){

                                            activeCellStatusList [counter1*12+4] = (**int**)(round((highestValue+lowextValue)/(**double**)2));

                                            activeCellStatusList [counter1*12+5] = 2;

                                            activeCellStatusList [counter1*12+6] = 1;

                                            activeCellStatusList [counter1*12+7] = 1;

                                            activeCellStatusList [counter1*12+9] = 0;

                                            activeCellStatusList [counter1*12+8] = (**int**)simProcessDataAddHold [18];

                                        }

**else**{

                                            activeCellStatusList [counter1*12+4] = siblinFusionDoub1+10;

                                            activeCellStatusList [counter1*12+5] = 2;

                                            activeCellStatusList [counter1*12+6] = 1;

                                            activeCellStatusList [counter1*12+7] = 1;

                                            activeCellStatusList [counter1*12+9] = 0;

                                            activeCellStatusList [counter1*12+8] = (**int**)simProcessDataAddHold [18];

                                        }

                                    }

**else**{

                                        activeCellStatusList [counter1*12+4] = siblinFusionDoub1+10;

                                        activeCellStatusList [counter1*12+5] = 2;

                                        activeCellStatusList [counter1*12+6] = 1;

                                        activeCellStatusList [counter1*12+7] = 1;

                                        activeCellStatusList [counter1*12+9] = 0;

                                        activeCellStatusList [counter1*12+8] = (**int**)simProcessDataAddHold [18];

                                    }

                                }

                            }

                        }

**else** **if** (eventType == 5){

**if** (expandBDCDSelCount == 0){

                                randInit = rand() % randCDRangeSelB + randCDRangeSelA;

**if** (siblinFusionCheck1 != 6){

                                    activeCellStatusList [counter1*12+4] = randInit;

                                    activeCellStatusList [counter1*12+5] = 3;

                                    activeCellStatusList [counter1*12+6] = 1;

                                    activeCellStatusList [counter1*12+9] = 0;

                                }

**else**{

                                    activeCellStatusList [counter1*12+4] = siblinFusionDoub1+10;

                                    activeCellStatusList [counter1*12+5] = 3;

                                    activeCellStatusList [counter1*12+6] = 1;

                                    activeCellStatusList [counter1*12+9] = 0;

                                }

                            }

**else**{

**if** (siblinFusionCheck1 != 6){

**if** (expandBDCDSelCount != 0){

                                        randInit = rand() % expandBDCDSelCount/2 + 0;

                                        activeCellStatusList [counter1*12+4] = expandBDCDSel [randInit*2];

                                        activeCellStatusList [counter1*12+5] = 3;

                                        activeCellStatusList [counter1*12+6] = 1;

                                        activeCellStatusList [counter1*12+9] = 0;

                                    }

**else**{

                                        activeCellStatusList [counter1*12+4] = siblinFusionDoub1+10;

                                        activeCellStatusList [counter1*12+5] = 3;

                                        activeCellStatusList [counter1*12+6] = 1;

                                        activeCellStatusList [counter1*12+9] = 0;

                                    }

                                }

**else**{

**if** (expandBDCDSelCount != 0){

                                        randInit = rand() % expandBDCDSelCount/2 + 0;

**if**(expandBDCDSel [randInit*2] > siblinFusionDoub1){

                                            activeCellStatusList [counter1*12+4] = expandBDCDSel [randInit*2]+10;

                                            activeCellStatusList [counter1*12+5] = 3;

                                            activeCellStatusList [counter1*12+6] = 1;

                                            activeCellStatusList [counter1*12+9] = 0;

                                        }

**else**{

                                            activeCellStatusList [counter1*12+4] = siblinFusionDoub1+10;

                                            activeCellStatusList [counter1*12+5] = 3;

                                            activeCellStatusList [counter1*12+6] = 1;

                                            activeCellStatusList [counter1*12+9] = 0;

                                        }

                                    }

**else**{

                                        activeCellStatusList [counter1*12+4] = siblinFusionDoub1+10;

                                        activeCellStatusList [counter1*12+5] = 3;

                                        activeCellStatusList [counter1*12+6] = 1;

                                        activeCellStatusList [counter1*12+9] = 0;

                                    }

                                }

                            }

                        }

**else** **if** (eventType == 6){

**if** (expandBDCFSelCount == 0){

                                randInit = rand() % randCDRangeSelB + randCDRangeSelA;

**if** (siblinFusionCheck1 != 6){

                                    activeCellStatusList [counter1*12+4] = randInit;

                                    activeCellStatusList [counter1*12+5] = 6;

                                    activeCellStatusList [counter1*12+6] = 1;

                                    activeCellStatusList [counter1*12+9] = 0;

                                }

**else**{

**if** (siblinFusionDoub1 < randBDRangeSelA) siblinFusionDoub1 = randBDRangeSelA;

                                    activeCellStatusList [siblingCellNo1*12+4] = siblinFusionDoub1+10;

                                    activeCellStatusList [counter1*12+4] = siblinFusionDoub1;

                                    activeCellStatusList [counter1*12+5] = 6;

                                    activeCellStatusList [counter1*12+6] = 1;

                                    activeCellStatusList [counter1*12+9] = 0;

                                }

                            }

**else**{

**if** (siblinFusionCheck1 == 0){

**if** (expandBDCFSelCount != 0){

                                        randInit = rand() % expandBDCFSelCount/2 + 0;

                                        activeCellStatusList [counter1*12+4] = expandBDCFSel [randInit*2];

                                        activeCellStatusList [counter1*12+5] = 6;

                                        activeCellStatusList [counter1*12+6] = 1;

                                        activeCellStatusList [counter1*12+9] = 0;

                                    }

**else** {

                                        activeCellStatusList [counter1*12+4] = siblinFusionDoub1+10;

                                        activeCellStatusList [counter1*12+5] = 6;

                                        activeCellStatusList [counter1*12+6] = 1;

                                        activeCellStatusList [counter1*12+9] = 0;

                                    }

                                }

**else**{

**if** (expandBDCDSelCount != 0){

                                        randInit = rand() % expandBDCDSelCount/2 + 0;

**if**(expandBDCDSel [randInit*2] > siblinFusionDoub1){

                                            activeCellStatusList [counter1*12+4] = expandBDCDSel [randInit*2]+10;

                                            activeCellStatusList [counter1*12+5] = 3;

                                            activeCellStatusList [counter1*12+6] = 1;

                                            activeCellStatusList [counter1*12+9] = 0;

                                        }

**else**{

                                            activeCellStatusList [counter1*12+4] = siblinFusionDoub1+10;

                                            activeCellStatusList [counter1*12+5] = 3;

                                            activeCellStatusList [counter1*12+6] = 1;

                                            activeCellStatusList [counter1*12+9] = 0;

                                        }

                                    }

**else**{

                                        activeCellStatusList [siblingCellNo1*12+4] = siblinFusionDoub1+10;

                                        activeCellStatusList [counter1*12+4] = siblinFusionDoub1;

                                        activeCellStatusList [counter1*12+5] = 6;

                                        activeCellStatusList [counter1*12+6] = 1;

                                        activeCellStatusList [counter1*12+9] = 0;

                                    }

                                }

                            }

                        }

                    }

**else** **if** (activeCellStatusList [counter1*12+5] == 2){

                        siblinFusionCheck1 = 0;

                        siblinFusionCheck2 = 0;

                        siblinFusionDoub1 = 0;

                        siblinFusionDoub2 = 0;

                        siblingCellNo1 = 0;

                        siblingCellNo2 = 0;

**for** (**int** counter2 = 0; counter2 < activeCellStatusListCount/12; counter2++){

**if** (activeCellStatusList [counter2*12+6] == 1 && (activeCellStatusList [counter2*12+1] == activeCellStatusList [counter1*12] || activeCellStatusList [counter2*12+2] == activeCellStatusList [counter1*12]) && activeCellStatusList [counter2*12+3] == activeCellStatusList [counter1*12+3]){

**if** (siblinFusionCheck1 == 0 && activeCellStatusList [counter2*12+5] != 0){

                                    siblinFusionCheck1 = activeCellStatusList [counter2*12+5];

                                    siblinFusionDoub1 = activeCellStatusList [counter2*12+4];

                                    siblingCellNo1 = counter2;

                                }

**else** **if** (siblinFusionCheck1 != 0 && siblinFusionCheck2 == 0 && activeCellStatusList [counter2*12+5] != 0){

                                    siblinFusionCheck2 = activeCellStatusList [counter2*12+5];

                                    siblinFusionDoub2 = activeCellStatusList [counter2*12+4];

                                    siblingCellNo2 = counter2;

**break**;

                                }

                            }

                        }

                        eventType = 0;

**if** (siblinFusionCheck1 == 6 && siblinFusionCheck2 == 6){

**for** (**int** counter2 = 0; counter2 < 100; counter2++){

                                randInit = rand() % 100 + 0;

**if** (secondEventTDListSel [randInit] != 10){

                                    eventType = secondEventTDListSel [randInit];

                                    loopCheck = 1;

**break**;

                                }

                            }

**if** (loopCheck == 0) eventType = 13;

                        }

**else**{

                            randInit = rand() % 100 + 0;

                            eventType = secondEventTDListSel [randInit];

**if** (eventType == 0) eventType = 13;

                        }

**if** ((activeCellStatusList [counter1*12+8] == 2 || activeCellStatusList [counter1*12+8] == 3) && activeCellStatusList [counter1*12+8] == activeCellStatusList [counter1*12+7] && eventType == 12){

                            eventType = 13;

                        }

**if** (siblinFusionDoub1 < siblinFusionDoub2) siblinFusionDoubLarge = siblinFusionDoub2;

**else** siblinFusionDoubLarge = siblinFusionDoub1;

**if** (siblinFusionCheck1 == 6 && siblinFusionCheck2 == 6 && eventType == 10){

                            eventType = 13;

                        }

**if** (eventType == 10){

                            loopCheck = 0;

**if** (siblinFusionCheck1 != 0 && siblinFusionCheck2 != 0){

**if** (siblinFusionCheck1 == 6 && siblinFusionCheck2 != 6){

**if** (expandTDCFSelCount == 0){

                                        randInit = rand() % randCDRangeSelB + randCDRangeSelA;

**if** (siblinFusionDoub2 < randBDRangeSelA) siblinFusionDoub2 = randBDRangeSelA;

**if** (siblinFusionDoub1 > siblinFusionDoub2){

**if** (randInit > siblinFusionDoub1){

                                                activeCellStatusList [siblingCellNo2*12+4] = randInit+10;

                                                activeCellStatusList [counter1*12+4] = siblinFusionDoub2;

                                                activeCellStatusList [counter1*12+5] = 6;

                                                activeCellStatusList [counter1*12+6] = 1;

                                            }

**else**{

                                                activeCellStatusList [siblingCellNo2*12+4] = siblinFusionDoub1+10;

                                                activeCellStatusList [counter1*12+4] = siblinFusionDoub2;

                                                activeCellStatusList [counter1*12+5] = 6;

                                                activeCellStatusList [counter1*12+6] = 1;

                                            }

                                        }

**else** **if** (siblinFusionDoub1 < siblinFusionDoub2){

**if** (randInit > siblinFusionDoub2){

                                                activeCellStatusList [siblingCellNo2*12+4] = randInit+10;

                                                activeCellStatusList [counter1*12+4] = siblinFusionDoub2;

                                                activeCellStatusList [counter1*12+5] = 6;

                                                activeCellStatusList [counter1*12+6] = 1;

                                            }

**else**{

                                                activeCellStatusList [siblingCellNo2*12+4] = siblinFusionDoub2+10;

                                                activeCellStatusList [counter1*12+4] = siblinFusionDoub2;

                                                activeCellStatusList [counter1*12+5] = 6;

                                                activeCellStatusList [counter1*12+6] = 1;

                                            }

                                        }

                                    }

**else**{

**if** (siblinFusionDoub1 > siblinFusionDoub2){

**if** (expandTDCFSelCount != 0){

                                                randInit = rand() % expandTDCFSelCount/2 + 0;

**if** (expandTDCFSel [randInit*2] > siblinFusionDoub1){

                                                    activeCellStatusList [siblingCellNo2*12+4] = expandTDCFSel [randInit*2]+10;

                                                    activeCellStatusList [counter1*12+4] = siblinFusionDoub2;

                                                    activeCellStatusList [counter1*12+5] = 6;

                                                    activeCellStatusList [counter1*12+6] = 1;

                                                }

**else**{

                                                    activeCellStatusList [siblingCellNo2*12+4] = siblinFusionDoub1+10;

                                                    activeCellStatusList [counter1*12+4] = siblinFusionDoub2;

                                                    activeCellStatusList [counter1*12+5] = 6;

                                                    activeCellStatusList [counter1*12+6] = 1;

                                                }

                                            }

**else**{

                                                activeCellStatusList [siblingCellNo2*12+4] = siblinFusionDoub1+10;

                                                activeCellStatusList [counter1*12+4] = siblinFusionDoub2;

                                                activeCellStatusList [counter1*12+5] = 6;

                                                activeCellStatusList [counter1*12+6] = 1;

                                            }

                                        }

**else** **if** (siblinFusionDoub1 < siblinFusionDoub2){

**if** (expandTDCFSelCount != 0){

                                                randInit = rand() % expandTDCFSelCount/2 + 0;

**if** (expandTDCFSel [randInit*2] > siblinFusionDoub2){

                                                    activeCellStatusList [siblingCellNo2*12+4] = expandTDCFSel [randInit*2]+10;

                                                    activeCellStatusList [counter1*12+4] = siblinFusionDoub2;

                                                    activeCellStatusList [counter1*12+5] = 6;

                                                    activeCellStatusList [counter1*12+6] = 1;

                                                }

**else**{

                                                    activeCellStatusList [siblingCellNo2*12+4] = siblinFusionDoub2+10;

                                                    activeCellStatusList [counter1*12+4] = siblinFusionDoub2;

                                                    activeCellStatusList [counter1*12+5] = 6;

                                                    activeCellStatusList [counter1*12+6] = 1;

                                                }

                                            }

**else**{

                                                activeCellStatusList [siblingCellNo2*12+4] = siblinFusionDoub2+10;

                                                activeCellStatusList [counter1*12+4] = siblinFusionDoub2;

                                                activeCellStatusList [counter1*12+5] = 6;

                                                activeCellStatusList [counter1*12+6] = 1;

                                            }

                                        }

                                    }

                                }

**else** **if** (siblinFusionCheck1 != 6 && siblinFusionCheck2 == 6){

**if** (expandTDCFSelCount == 0){

                                        randInit = rand() % randCDRangeSelB + randCDRangeSelA;

**if** (siblinFusionDoub1 > siblinFusionDoub2){

**if** (randInit > siblinFusionDoub1){

                                                activeCellStatusList [siblingCellNo1*12+4] = randInit+10;

                                                activeCellStatusList [counter1*12+4] = siblinFusionDoub1;

                                                activeCellStatusList [counter1*12+5] = 6;

                                                activeCellStatusList [counter1*12+6] = 1;

                                            }

**else**{

                                                activeCellStatusList [siblingCellNo1*12+4] = siblinFusionDoub1+10;

                                                activeCellStatusList [counter1*12+4] = siblinFusionDoub1;

                                                activeCellStatusList [counter1*12+5] = 6;

                                                activeCellStatusList [counter1*12+6] = 1;

                                            }

                                        }

**else** **if** (siblinFusionDoub1 < siblinFusionDoub2){

**if** (randInit > siblinFusionDoub2){

                                                activeCellStatusList [siblingCellNo1*12+4] = randInit+10;

                                                activeCellStatusList [counter1*12+4] = siblinFusionDoub1;

                                                activeCellStatusList [counter1*12+5] = 6;

                                                activeCellStatusList [counter1*12+6] = 1;

                                            }

**else**{

                                                activeCellStatusList [siblingCellNo1*12+4] = siblinFusionDoub2+10;

                                                activeCellStatusList [counter1*12+4] = siblinFusionDoub1;

                                                activeCellStatusList [counter1*12+5] = 6;

                                                activeCellStatusList [counter1*12+6] = 1;

                                            }

                                        }

                                    }

**else**{

**if** (siblinFusionDoub1 > siblinFusionDoub2){

**if** (expandTDCFSelCount != 0){

                                                randInit = rand() % expandTDCFSelCount/2 + 0;

**if** (expandTDCFSel [randInit*2] > siblinFusionDoub1){

                                                    activeCellStatusList [siblingCellNo1*12+4] = expandTDCFSel [randInit*2]+10;

                                                    activeCellStatusList [counter1*12+4] = siblinFusionDoub1;

                                                    activeCellStatusList [counter1*12+5] = 6;

                                                    activeCellStatusList [counter1*12+6] = 1;

                                                }

**else**{

                                                    activeCellStatusList [siblingCellNo1*12+4] = siblinFusionDoub1+10;

                                                    activeCellStatusList [counter1*12+4] = siblinFusionDoub1;

                                                    activeCellStatusList [counter1*12+5] = 6;

                                                    activeCellStatusList [counter1*12+6] = 1;

                                                }

                                            }

**else**{

                                                activeCellStatusList [siblingCellNo1*12+4] = siblinFusionDoub1+10;

                                                activeCellStatusList [counter1*12+4] = siblinFusionDoub1;

                                                activeCellStatusList [counter1*12+5] = 6;

                                                activeCellStatusList [counter1*12+6] = 1;

                                            }

                                        }

**else** **if** (siblinFusionDoub1 < siblinFusionDoub2){

**if** (expandTDCFSelCount != 0){

                                                randInit = rand() % expandTDCFSelCount/2 + 0;

**if** (expandTDCF [randInit*2] >= siblinFusionDoub2){

                                                    activeCellStatusList [siblingCellNo1*12+4] = expandTDCFSel [randInit*2]+10;

                                                    activeCellStatusList [counter1*12+4] = siblinFusionDoub1;

                                                    activeCellStatusList [counter1*12+5] = 6;

                                                    activeCellStatusList [counter1*12+6] = 1;

                                                }

**else**{

                                                    activeCellStatusList [siblingCellNo1*12+4] = siblinFusionDoub2+10;

                                                    activeCellStatusList [counter1*12+4] = siblinFusionDoub1;

                                                    activeCellStatusList [counter1*12+5] = 6;

                                                    activeCellStatusList [counter1*12+6] = 1;

                                                }

                                            }

**else**{

                                                activeCellStatusList [siblingCellNo1*12+4] = siblinFusionDoub2+10;

                                                activeCellStatusList [counter1*12+4] = siblinFusionDoub1;

                                                activeCellStatusList [counter1*12+5] = 6;

                                                activeCellStatusList [counter1*12+6] = 1;

                                            }

                                        }

                                    }

                                }

**else** **if** (siblinFusionCheck1 != 6 && siblinFusionCheck2 != 6){

                                    activeCellStatusList [siblingCellNo1*12+4] = siblinFusionDoub1+10;

                                    activeCellStatusList [siblingCellNo2*12+4] = siblinFusionDoub2+10;

                                    activeCellStatusList [counter1*12+4] = siblinFusionDoub2;

                                    activeCellStatusList [counter1*12+5] = 6;

                                    activeCellStatusList [counter1*12+6] = 1;

                                }

                            }

**else** **if** (siblinFusionCheck1 != 0 && siblinFusionCheck2 == 0){

**if** (expandTDCFSelCount == 0){

                                    randInit = rand() % randCDRangeSelB + randCDRangeSelA;

**if** (randInit > siblinFusionDoub1+10){

                                        activeCellStatusList [counter1*12+4] = randInit;

                                        activeCellStatusList [counter1*12+5] = 6;

                                        activeCellStatusList [counter1*12+6] = 1;

                                    }

**else**{

                                        activeCellStatusList [counter1*12+4] = siblinFusionDoub1+5;

                                        activeCellStatusList [counter1*12+5] = 6;

                                        activeCellStatusList [counter1*12+6] = 1;

                                    }

                                }

**else**{

**if** (expandTDCFSelCount != 0){

                                        randInit = rand() % expandTDCFSelCount/2 + 0;

**if** (expandTDCFSel [randInit*2] > siblinFusionDoub1+10){

                                            activeCellStatusList [counter1*12+4] = expandTDCFSel [randInit*2];

                                            activeCellStatusList [counter1*12+5] = 6;

                                            activeCellStatusList [counter1*12+6] = 1;

                                        }

**else**{

                                            activeCellStatusList [counter1*12+4] = siblinFusionDoub1+5;

                                            activeCellStatusList [counter1*12+5] = 6;

                                            activeCellStatusList [counter1*12+6] = 1;

                                        }

                                    }

**else**{

                                        activeCellStatusList [counter1*12+4] = siblinFusionDoub1+5;

                                        activeCellStatusList [counter1*12+5] = 6;

                                        activeCellStatusList [counter1*12+6] = 1;

                                    }

                                }

                            }

**else** **if** (siblinFusionCheck1 == 0 && siblinFusionCheck2 == 0){

**if** (expandTDCFSelCount == 0){

                                    randInit = rand() % randCDRangeSelB + randCDRangeSelA;

                                    activeCellStatusList [counter1*12+4] = randInit;

                                    activeCellStatusList [counter1*12+5] = 6;

                                    activeCellStatusList [counter1*12+6] = 1;

                                }

**else**{

**if** (activeCellStatusList [counter1*12+11] == 0){

                                        randInit = rand() % expandTDCFSelCount/2 + 0;

                                        activeCellStatusList [counter1*12+4] = expandTDCFSel [randInit*2];

                                        activeCellStatusList [counter1*12+5] = 6;

                                        activeCellStatusList [counter1*12+6] = 1;

                                    }

**else**{

                                        randInit = rand() % expandTDCFSelCount/2 + 0;

                                        activeCellStatusList [counter1*12+4] = expandTDCFSel [randInit*2];

                                        activeCellStatusList [counter1*12+5] = 6;

                                        activeCellStatusList [counter1*12+6] = 1;

                                    }

                                }

                            }

                        }

**else** **if** (eventType == 11){

**if** (expandDoublingDoubTDSelCount == 0){

                                randInit = rand() % randBDRangeSelB + randBDRangeSelA;

**if** (randInit > siblinFusionDoubLarge+10){

                                    activeCellStatusList [counter1*12+4] = randInit;

                                    activeCellStatusList [counter1*12+5] = 1;

                                    activeCellStatusList [counter1*12+6] = 1;

                                    activeCellStatusList [counter1*12+9] = (**int**)simProcessDataAddHold [19];

                                    activeCellStatusList [counter1*12+7] = 0;

                                }

**else**{

**if** (siblinFusionDoubLarge < randBDRangeSelA) siblinFusionDoubLarge = randBDRangeSelA;

                                    activeCellStatusList [counter1*12+4] = siblinFusionDoubLarge+10;

                                    activeCellStatusList [counter1*12+5] = 1;

                                    activeCellStatusList [counter1*12+6] = 1;

                                    activeCellStatusList [counter1*12+9] = (**int**)simProcessDataAddHold [19];

                                    activeCellStatusList [counter1*12+7] = 0;

                                }

                            }

**else**{

**if** (activeCellStatusList [counter1*12+11] == 0){

**if** (expandDoublingDoubTDSelCount != 0){

                                        randInit = rand() % expandDoublingDoubTDSelCount/2 + 0;

**if** (randInit > siblinFusionDoubLarge+10){

                                            activeCellStatusList [counter1*12+4] = expandDoublingDoubTDSel [randInit*2];

                                            activeCellStatusList [counter1*12+5] = 1;

                                            activeCellStatusList [counter1*12+6] = 1;

                                            activeCellStatusList [counter1*12+9] = (**int**)simProcessDataAddHold [19];

                                            activeCellStatusList [counter1*12+7] = 0;

                                        }

**else**{

**if** (siblinFusionDoubLarge < randBDRangeSelA) siblinFusionDoubLarge = randBDRangeSelA;

                                            activeCellStatusList [counter1*12+4] = siblinFusionDoubLarge+10;

                                            activeCellStatusList [counter1*12+5] = 1;

                                            activeCellStatusList [counter1*12+6] = 1;

                                            activeCellStatusList [counter1*12+9] = (**int**)simProcessDataAddHold [19];

                                            activeCellStatusList [counter1*12+7] = 0;

                                        }

                                    }

**else**{

**if** (siblinFusionDoubLarge < randBDRangeSelA) siblinFusionDoubLarge = randBDRangeSelA;

                                        activeCellStatusList [counter1*12+4] = siblinFusionDoubLarge+10;

                                        activeCellStatusList [counter1*12+5] = 1;

                                        activeCellStatusList [counter1*12+6] = 1;

                                        activeCellStatusList [counter1*12+9] = (**int**)simProcessDataAddHold [19];

                                        activeCellStatusList [counter1*12+7] = 0;

                                    }

                                }

**else**{

**if** (expandDoublingDoubTDSelCount != 0){

                                        randInit = rand() % expandDoublingDoubTDSelCount/2 + 0;

**if** (expandDoublingDoubTDSel [randInit*2] > siblinFusionDoubLarge+10){

                                            activeCellStatusList [counter1*12+4] = expandDoublingDoubTDSel [randInit*2];

                                            activeCellStatusList [counter1*12+5] = 1;

                                            activeCellStatusList [counter1*12+6] = 1;

                                            activeCellStatusList [counter1*12+9] = (**int**)simProcessDataAddHold [19];

                                            activeCellStatusList [counter1*12+7] = 0;

                                        }

**else**{

**if** (siblinFusionDoubLarge < randBDRangeSelA) siblinFusionDoubLarge = randBDRangeSelA;

                                            activeCellStatusList [counter1*12+4] = siblinFusionDoubLarge+10;

                                            activeCellStatusList [counter1*12+5] = 1;

                                            activeCellStatusList [counter1*12+6] = 1;

                                            activeCellStatusList [counter1*12+9] = (**int**)simProcessDataAddHold [19];

                                            activeCellStatusList [counter1*12+7] = 0;

                                        }

                                    }

**else**{

**if** (siblinFusionDoubLarge < randBDRangeSelA) siblinFusionDoubLarge = randBDRangeSelA;

                                        activeCellStatusList [counter1*12+4] = siblinFusionDoubLarge+10;

                                        activeCellStatusList [counter1*12+5] = 1;

                                        activeCellStatusList [counter1*12+6] = 1;

                                        activeCellStatusList [counter1*12+9] = (**int**)simProcessDataAddHold [19];

                                        activeCellStatusList [counter1*12+7] = 0;

                                    }

                                }

                            }

                        }

**else** **if** (eventType == 12){

**if** (expandDoublingDoubTDSelCount == 0){

                                randInit = rand() % randBDRangeSelB + randBDRangeSelA;

**if** (randInit > siblinFusionDoubLarge+10){

                                    activeCellStatusList [counter1*12+4] = randInit;

                                    activeCellStatusList [counter1*12+5] = 2;

                                    activeCellStatusList [counter1*12+6] = 1;

                                    activeCellStatusList [counter1*12+7]++;

                                }

**else**{

**if** (siblinFusionDoubLarge < randBDRangeSelA) siblinFusionDoubLarge = randBDRangeSelA;

                                    activeCellStatusList [counter1*12+4] = siblinFusionDoubLarge+10;

                                    activeCellStatusList [counter1*12+5] = 2;

                                    activeCellStatusList [counter1*12+6] = 1;

                                    activeCellStatusList [counter1*12+7]++;

                                }

                            }

**else**{

**if** (activeCellStatusList [counter1*12+11] == 0){

**if** (expandDoublingDoubTDSelCount != 0){

                                        randInit = rand() % expandDoublingDoubTDSelCount/2 + 0;

**if** (randInit >= siblinFusionDoubLarge+10){

                                            activeCellStatusList [counter1*12+4] = expandDoublingDoubTDSel [randInit*2];

                                            activeCellStatusList [counter1*12+5] = 2;

                                            activeCellStatusList [counter1*12+6] = 1;

                                            activeCellStatusList [counter1*12+7]++;

                                        }

**else**{

**if** (siblinFusionDoubLarge < randBDRangeSelA) siblinFusionDoubLarge = randBDRangeSelA;

                                            activeCellStatusList [counter1*12+4] = siblinFusionDoubLarge+10;

                                            activeCellStatusList [counter1*12+5] = 2;

                                            activeCellStatusList [counter1*12+6] = 1;

                                            activeCellStatusList [counter1*12+7]++;

                                        }

                                    }

**else**{

**if** (siblinFusionDoubLarge < randBDRangeSelA) siblinFusionDoubLarge = randBDRangeSelA;

                                        activeCellStatusList [counter1*12+4] = siblinFusionDoubLarge+10;

                                        activeCellStatusList [counter1*12+5] = 2;

                                        activeCellStatusList [counter1*12+6] = 1;

                                        activeCellStatusList [counter1*12+7]++;

                                    }

                                }

**else**{

**if** (expandDoublingDoubTDSelCount != 0){

                                        randInit = rand() % expandDoublingDoubTDSelCount/2 + 0;

**if** (expandDoublingDoubTDSel [randInit*2] > siblinFusionDoubLarge+10){

                                            activeCellStatusList [counter1*12+4] = expandDoublingDoubTDSel [randInit*2];

                                            activeCellStatusList [counter1*12+5] = 2;

                                            activeCellStatusList [counter1*12+7]++;

                                        }

**else**{

**if** (siblinFusionDoubLarge < randBDRangeSelA) siblinFusionDoubLarge = randBDRangeSelA;

                                            activeCellStatusList [counter1*12+4] = siblinFusionDoubLarge+10;

                                            activeCellStatusList [counter1*12+5] = 2;

                                            activeCellStatusList [counter1*12+7]++;

                                        }

                                    }

**else**{

**if** (siblinFusionDoubLarge < randBDRangeSelA) siblinFusionDoubLarge = randBDRangeSelA;

                                        activeCellStatusList [counter1*12+4] = siblinFusionDoubLarge+10;

                                        activeCellStatusList [counter1*12+5] = 2;

                                        activeCellStatusList [counter1*12+7]++;

                                    }

                                }

                            }

                        }

**else** **if** (eventType == 13){

**if** (expandTDCDSelCount == 0){

                                randInit = rand() % randCDRangeSelB + randCDRangeSelA;

**if** (randInit > siblinFusionDoubLarge+10){

                                    activeCellStatusList [counter1*12+4] = randInit;

                                    activeCellStatusList [counter1*12+5] = 3;

                                    activeCellStatusList [counter1*12+6] = 1;

                                }

**else**{

                                    activeCellStatusList [counter1*12+4] = siblinFusionDoubLarge+10;

                                    activeCellStatusList [counter1*12+5] = 3;

                                    activeCellStatusList [counter1*12+6] = 1;

                                }

                            }

**else**{

**if** (expandTDCDSelCount != 0){

                                    randInit = rand() % expandTDCDSelCount/2 + 0;

**if** (expandTDCDSel [randInit*2] > siblinFusionDoubLarge+10){

                                        activeCellStatusList [counter1*12+4] = expandTDCDSel [randInit*2];

                                        activeCellStatusList [counter1*12+5] = 3;

                                        activeCellStatusList [counter1*12+6] = 1;

                                    }

**else**{

                                        activeCellStatusList [counter1*12+4] = siblinFusionDoubLarge+10;

                                        activeCellStatusList [counter1*12+5] = 3;

                                        activeCellStatusList [counter1*12+6] = 1;

                                    }

                                }

**else**{

                                    activeCellStatusList [counter1*12+4] = siblinFusionDoubLarge+10;

                                    activeCellStatusList [counter1*12+5] = 3;

                                    activeCellStatusList [counter1*12+6] = 1;

                                }

                            }

                        }

                    }

                }

            }

        }

**if** (callCount == 0){

            nonDivAssign = 0;

            otherDivAssign = 0;

**for** (**int** counterA = 0; counterA < activeCellStatusListCount/12; counterA++){

**if** (activeCellStatusList [counterA*12+5] == 5 || activeCellStatusList [counterA*12+5] == 4){

                    nonDivAssign++;

                }

**else** otherDivAssign++;

            }

            processingStatusCall4 = 1;

            processingStatus4 = to_string (nonDivAssign)+"/"+to_string (otherDivAssign);

            callCount = 1;

        }

**if** (terminate2 == 0){

**for** (**int** counter1 = 0; counter1 < activeCellStatusListCount/12; counter1++){

                activeCellStatusList [counter1*12+6] = 0;

            }

            siblingCellPosition1 = 0;

            siblingCellPosition2 = 0;

**for** (**int** counter1 = 0; counter1 < activeCellStatusListCount/12; counter1++){

**if** (terminateSimFlag == 1){

                    terminate2 = 1;

**break**;

                }

                selectCheck = 0;

**if** (doseSimStatusHold == 2){

**for** (**int** counter3 = 0; counter3 < lingNoAssigineSimCount; counter3++){

**if** (lingNoAssigineSim [counter3] == activeCellStatusList [counter1*12+3]){

                            selectCheck = 1;

**break**;

                        }

                    }

                }

**if** (selectCheck == 0){

**if** (activeCellStatusList [counter1*12+5] == 6){

**if** (activeCellStatusList [counter1*12+2] == 0){

                            siblinFusionDoub1 = 0;

                            siblingCellNo1 = 0;

**for** (**int** counter2 = 0; counter2 < activeCellStatusListCount/12; counter2++){

**if** (activeCellStatusList [counter2*12+1] == activeCellStatusList [counter1*12] && activeCellStatusList [counter2*12+3] == activeCellStatusList [counter1*12+3]){

                                    siblingCellNo1 = activeCellStatusList [counter2*12];

                                    siblinFusionDoub1 = activeCellStatusList [counter2*12+4];

                                    siblingCellPosition1 = counter2;

                                    fusionCount++;

**break**;

                                }

                            }

                            randInit = rand() % 100 + 0;

**if** (secondEventBDCFList [randInit] == 0){

                                activeCellStatusList [counter1*12+6] = siblingCellNo1;

                                fusionCount++;

                            }

**else** **if** (secondEventBDCFList [randInit] == 7){

                                loopCheck = 0;

**if** (expandDoublingDoubCFCount != 0){

                                    randInit = rand() % expandDoublingDoubCFCount/2 + 0;

**if** (expandDoublingDoubCF [randInit*2] > siblinFusionDoub1){

                                        activeCellStatusList [siblingCellPosition1*12+4] = expandDoublingDoubCF [randInit*2];

                                        activeCellStatusList [siblingCellPosition1*12+5] = 1;

                                        activeCellStatusList [counter1*12+6] = siblingCellNo1;

                                        activeCellStatusList [siblingCellPosition1*12+7] = 0;

                                        activeCellStatusList [siblingCellPosition1*12+8] = 0;

                                        fusionCount++;

                                        loopCheck = 1;

                                    }

**else**{

                                        activeCellStatusList [siblingCellPosition1*12+5] = 1;

                                        activeCellStatusList [counter1*12+6] = siblingCellNo1;

                                        activeCellStatusList [siblingCellPosition1*12+7] = 0;

                                        activeCellStatusList [siblingCellPosition1*12+8] = 0;

                                        fusionCount++;

                                        loopCheck = 1;

                                    }

                                }

**if** (loopCheck == 0){

                                    activeCellStatusList [siblingCellPosition1*12+5] = 1;

                                    activeCellStatusList [counter1*12+6] = siblingCellNo1;

                                    activeCellStatusList [siblingCellPosition1*12+7] = 0;

                                    activeCellStatusList [siblingCellPosition1*12+8] = 0;

                                    fusionCount++;

                                }

                            }

**else** **if** (secondEventBDCFList [randInit] == 8){

                                loopCheck = 0;

**if** (expandDoublingDoubCFCount != 0){

                                    randInit = rand() % expandDoublingDoubCFCount/2 + 0;

**if** (expandDoublingDoubCF [randInit*2] > siblinFusionDoub1){

**if** (activeCellStatusList [siblingCellPosition1*12+5] != 2) activeCellStatusList [siblingCellPosition1*12+7]++;

                                        activeCellStatusList [siblingCellPosition1*12+4] = expandDoublingDoubCF [randInit*2];

                                        activeCellStatusList [siblingCellPosition1*12+5] = 2;

                                        activeCellStatusList [counter1*12+6] = siblingCellNo1;

                                        activeCellStatusList [siblingCellPosition1*12+8] = (**int**)simProcessDataMiddleHold [18];

                                        fusionCount++;

                                        loopCheck = 1;

                                    }

**else**{

**if** (activeCellStatusList [siblingCellPosition1*12+5] != 2) activeCellStatusList [siblingCellPosition1*12+7]++;

                                        activeCellStatusList [siblingCellPosition1*12+5] = 2;

                                        activeCellStatusList [counter1*12+6] = siblingCellNo1;

                                        activeCellStatusList [siblingCellPosition1*12+8] = (**int**)simProcessDataMiddleHold [18];

                                        fusionCount++;

                                        loopCheck = 1;

                                    }

                                }

**if** (loopCheck == 0){

**if** (activeCellStatusList [siblingCellPosition1*12+5] != 2) activeCellStatusList [siblingCellPosition1*12+7]++;

                                    activeCellStatusList [siblingCellPosition1*12+5] = 2;

                                    activeCellStatusList [counter1*12+6] = siblingCellNo1;

                                    activeCellStatusList [siblingCellPosition1*12+8] = (**int**)simProcessDataMiddleHold [18];

                                    fusionCount++;

                                }

                            }

**else** **if** (secondEventBDCFList [randInit] == 9){

**if** (expandBDCFCDCount != 0){

                                    randInit = rand() % expandBDCFCDCount/2 + 0;

**if** (expandBDCFCD [randInit*2] > siblinFusionDoub1){

                                        activeCellStatusList [siblingCellPosition1*12+4] = expandBDCFCD [randInit*2];

                                        activeCellStatusList [siblingCellPosition1*12+5] = 3;

                                        activeCellStatusList [counter1*12+6] = siblingCellNo1;

                                        fusionCount++;

                                    }

**else**{

                                        activeCellStatusList [siblingCellPosition1*12+5] = 3;

                                        activeCellStatusList [counter1*12+6] = siblingCellNo1;

                                        fusionCount++;

                                    }

                                }

**else**{

                                    activeCellStatusList [siblingCellPosition1*12+5] = 3;

                                    activeCellStatusList [counter1*12+6] = siblingCellNo1;

                                    fusionCount++;

                                }

                            }

                        }

**else**{

                            siblinFusionCheck1 = 0;

                            siblinFusionCheck2 = 0;

                            siblinFusionDoub1 = 0;

                            siblinFusionDoub2 = 0;

                            siblingCellNo1 = 0;

                            siblingCellNo2 = 0;

**for** (**int** counter2 = 0; counter2 < activeCellStatusListCount/12; counter2++){

**if** ((activeCellStatusList [counter2*12+1] == activeCellStatusList [counter1*12] || activeCellStatusList [counter2*12+2] == activeCellStatusList [counter1*12]) && activeCellStatusList [counter2*12+3] == activeCellStatusList [counter1*12+3]){

**if** (siblinFusionCheck1 == 0 && activeCellStatusList [counter2*12+5] != 0){

                                        siblinFusionCheck1 = activeCellStatusList [counter2*12+5];

                                        siblinFusionDoub1 = activeCellStatusList [counter2*12+4];

                                        siblingCellNo1 = activeCellStatusList [counter2*12];

                                        siblingCellPosition1 = counter2;

                                    }

**else** **if** (siblinFusionCheck1 != 0 && siblinFusionCheck2 == 0 && activeCellStatusList [counter2*12+5] != 0){

                                        siblinFusionCheck2 = activeCellStatusList [counter2*12+5];

                                        siblinFusionDoub2 = activeCellStatusList [counter2*12+4];

                                        siblingCellNo2 = activeCellStatusList [counter2*12];

                                        siblingCellPosition2 = counter2;

**break**;

                                    }

                                }

                            }

**if** (siblinFusionCheck1 == 6 && siblinFusionCheck2 != 6){

                                randInit = rand() % 100 + 0;

**if** (secondEventTDCFList [randInit] == 0){

                                    activeCellStatusList [siblingCellPosition1*12+6] = siblingCellNo2;

                                    activeCellStatusList [counter1*12+6] = siblingCellNo2;

                                    fusionCount++;

                                }

**else**{

**if** ((activeCellStatusList [counter1*12+8] == 2 || activeCellStatusList [counter1*12+8] == 3) && activeCellStatusList [counter1*12+8] == activeCellStatusList [counter1*12+7] && secondEventTDCFList [randInit] == 15){

                                        selectChange = 16;

                                    }

**else** selectChange = secondEventTDCFList [randInit];

**if** (selectChange == 14){

                                        loopCheck = 0;

**if** (expandDoublingDoubCFCount != 0){

                                            randInit = rand() % expandDoublingDoubCFCount/2 + 0;

**if** (expandDoublingDoubCF [randInit*2] > siblinFusionDoub2){

                                                activeCellStatusList [siblingCellPosition2*12+4] = expandDoublingDoubCF [randInit*2];

                                                activeCellStatusList [siblingCellPosition2*12+5] = 1;

                                                activeCellStatusList [siblingCellPosition1*12+6] = siblingCellNo2;

                                                activeCellStatusList [counter1*12+6] = siblingCellNo2;

                                                activeCellStatusList [siblingCellPosition2*12+7] = 0;

                                                activeCellStatusList [siblingCellPosition2*12+8] = 0;

                                                fusionCount++;

                                                loopCheck = 1;

                                            }

**else**{

                                                activeCellStatusList [siblingCellPosition2*12+5] = 1;

                                                activeCellStatusList [siblingCellPosition1*12+6] = siblingCellNo2;

                                                activeCellStatusList [counter1*12+6] = siblingCellNo2;

                                                activeCellStatusList [siblingCellPosition2*12+7] = 0;

                                                activeCellStatusList [siblingCellPosition2*12+8] = 0;

                                                fusionCount++;

                                                loopCheck = 1;

                                            }

                                        }

**if** (loopCheck == 0){

                                            activeCellStatusList [siblingCellPosition2*12+5] = 1;

                                            activeCellStatusList [siblingCellPosition1*12+6] = siblingCellNo2;

                                            activeCellStatusList [counter1*12+6] = siblingCellNo2;

                                            activeCellStatusList [siblingCellPosition2*12+7] = 0;

                                            activeCellStatusList [siblingCellPosition2*12+8] = 0;

                                            fusionCount++;

                                        }

                                    }

**else** **if** (selectChange == 15){

                                        loopCheck = 0;

**if** (expandDoublingDoubCFCount != 0){

                                            randInit = rand() % expandDoublingDoubCFCount/2 + 0;

**if** (expandDoublingDoubCF [randInit*2] > siblinFusionDoub2){

**if** (activeCellStatusList [siblingCellPosition2*12+5] != 2) activeCellStatusList [siblingCellPosition2*12+7]++;

                                                activeCellStatusList [siblingCellPosition2*12+4] = expandDoublingDoubCF [randInit*2];

                                                activeCellStatusList [siblingCellPosition2*12+5] = 2;

                                                activeCellStatusList [siblingCellPosition1*12+6] = siblingCellNo2;

                                                activeCellStatusList [counter1*12+6] = siblingCellNo2;

                                                activeCellStatusList [siblingCellPosition2*12+8] = (**int**)simProcessDataMiddleHold [18];

                                                fusionCount++;

                                                loopCheck = 1;

                                            }

**else**{

**if** (activeCellStatusList [siblingCellPosition2*12+5] != 2) activeCellStatusList [siblingCellPosition2*12+7]++;

                                                activeCellStatusList [siblingCellPosition2*12+5] = 2;

                                                activeCellStatusList [siblingCellPosition1*12+6] = siblingCellNo2;

                                                activeCellStatusList [counter1*12+6] = siblingCellNo2;

                                                activeCellStatusList [siblingCellPosition2*12+8] = (**int**)simProcessDataMiddleHold [18];

                                                fusionCount++;

                                                loopCheck = 1;

                                            }

                                        }

**if** (loopCheck == 0){

**if** (activeCellStatusList [siblingCellPosition2*12+5] != 2) activeCellStatusList [siblingCellPosition2*12+7]++;

                                            activeCellStatusList [siblingCellPosition2*12+5] = 2;

                                            activeCellStatusList [siblingCellPosition1*12+6] = siblingCellNo2;

                                            activeCellStatusList [counter1*12+6] = siblingCellNo2;

                                            activeCellStatusList [siblingCellPosition2*12+8] = (**int**)simProcessDataMiddleHold [18];

                                            fusionCount++;

                                        }

                                    }

**else** **if** (selectChange == 16){

**if** (expandTDCFCDCount != 0){

                                            randInit = rand() % expandTDCFCDCount/2 + 0;

**if** (expandTDCFCD [randInit*2] > siblinFusionDoub2){

                                                activeCellStatusList [siblingCellPosition2*12+4] = expandTDCFCD [randInit*2];

                                                activeCellStatusList [siblingCellPosition2*12+5] = 3;

                                                activeCellStatusList [siblingCellPosition1*12+6] = siblingCellNo2;

                                                activeCellStatusList [counter1*12+6] = siblingCellNo2;

                                                fusionCount++;

                                            }

**else**{

                                                activeCellStatusList [siblingCellPosition2*12+5] = 3;

                                                activeCellStatusList [siblingCellPosition1*12+6] = siblingCellNo2;

                                                activeCellStatusList [counter1*12+6] = siblingCellNo2;

                                                fusionCount++;

                                            }

                                        }

**else**{

                                            activeCellStatusList [siblingCellPosition2*12+5] = 3;

                                            activeCellStatusList [siblingCellPosition1*12+6] = siblingCellNo2;

                                            activeCellStatusList [counter1*12+6] = siblingCellNo2;

                                            fusionCount++;

                                        }

                                    }

                                }

                            }

**else** **if** (siblinFusionCheck1 != 6 && siblinFusionCheck2 == 6){

                                randInit = rand() % 100 + 0;

**if** (secondEventTDCFList [randInit] == 0){

                                    activeCellStatusList [siblingCellPosition2*12+6] = siblingCellNo1;

                                    activeCellStatusList [counter1*12+6] = siblingCellNo1;

                                    fusionCount++;

                                }

**else**{

**if** ((activeCellStatusList [counter1*12+8] == 2 || activeCellStatusList [counter1*12+8] == 3) && activeCellStatusList [counter1*12+8] == activeCellStatusList [counter1*12+7] && secondEventTDCFList [randInit] == 15){

                                        selectChange = 16;

                                    }

**else** selectChange = secondEventTDCFList [randInit];

**if** (selectChange == 14){

                                        loopCheck = 0;

**if** (expandDoublingDoubCFCount != 0){

                                            randInit = rand() % expandDoublingDoubCFCount/2 + 0;

**if** (expandDoublingDoubCF [randInit*2] > siblinFusionDoub1){

                                                activeCellStatusList [siblingCellPosition1*12+4] = expandDoublingDoubCF [randInit*2];

                                                activeCellStatusList [siblingCellPosition1*12+5] = 1;

                                                activeCellStatusList [siblingCellPosition2*12+6] = siblingCellNo1;

                                                activeCellStatusList [counter1*12+6] = siblingCellNo1;

                                                activeCellStatusList [siblingCellPosition1*12+7] = 0;

                                                activeCellStatusList [siblingCellPosition1*12+8] = 0;

                                                fusionCount++;

                                                loopCheck = 1;

                                            }

**else**{

                                                activeCellStatusList [siblingCellPosition1*12+5] = 1;

                                                activeCellStatusList [siblingCellPosition2*12+6] = siblingCellNo1;

                                                activeCellStatusList [counter1*12+6] = siblingCellNo1;

                                                activeCellStatusList [siblingCellPosition1*12+7] = 0;

                                                activeCellStatusList [siblingCellPosition1*12+8] = 0;

                                                fusionCount++;

                                                loopCheck = 1;

                                            }

                                        }

**if** (loopCheck == 0){

                                            activeCellStatusList [siblingCellPosition1*12+5] = 1;

                                            activeCellStatusList [siblingCellPosition2*12+6] = siblingCellNo1;

                                            activeCellStatusList [counter1*12+6] = siblingCellNo1;

                                            activeCellStatusList [siblingCellPosition1*12+7] = 0;

                                            activeCellStatusList [siblingCellPosition1*12+8] = 0;

                                            fusionCount++;

                                        }

                                    }

**else** **if** (selectChange == 15){

                                        loopCheck = 0;

**if** (expandDoublingDoubCFCount != 0){

                                            randInit = rand() % expandDoublingDoubCFCount/2 + 0;

**if** (expandDoublingDoubCF [randInit*2] > siblinFusionDoub1){

**if** (activeCellStatusList [siblingCellPosition1*12+5] != 2) activeCellStatusList [siblingCellPosition1*12+7]++;

                                                activeCellStatusList [siblingCellPosition1*12+4] = expandDoublingDoubCF [randInit*2];

                                                activeCellStatusList [siblingCellPosition1*12+5] = 2;

                                                activeCellStatusList [siblingCellPosition2*12+6] = siblingCellNo1;

                                                activeCellStatusList [counter1*12+6] = siblingCellNo1;

                                                activeCellStatusList [siblingCellPosition1*12+8] = (**int**)simProcessDataMiddleHold [18];

                                                fusionCount++;

                                                loopCheck = 1;

                                            }

**else**{

**if** (activeCellStatusList [siblingCellPosition1*12+5] != 2) activeCellStatusList [siblingCellPosition1*12+7]++;

                                                activeCellStatusList [siblingCellPosition1*12+5] = 2;

                                                activeCellStatusList [siblingCellPosition2*12+6] = siblingCellNo1;

                                                activeCellStatusList [counter1*12+6] = siblingCellNo1;

                                                activeCellStatusList [siblingCellPosition1*12+8] = (**int**)simProcessDataMiddleHold [18];

                                                fusionCount++;

                                                loopCheck = 1;

                                            }

                                        }

**if** (loopCheck == 0){

**if** (activeCellStatusList [siblingCellPosition1*12+5] != 2) activeCellStatusList [siblingCellPosition1*12+7]++;

                                            activeCellStatusList [siblingCellPosition1*12+5] = 2;

                                            activeCellStatusList [siblingCellPosition2*12+6] = siblingCellNo1;

                                            activeCellStatusList [counter1*12+6] = siblingCellNo1;

                                            activeCellStatusList [siblingCellPosition1*12+8] = (**int**)simProcessDataMiddleHold [18];

                                            fusionCount++;

                                        }

                                    }

**else** **if** (selectChange == 16){

**if** (expandTDCFCDCount != 0){

                                            randInit = rand() % expandTDCFCDCount/2 + 0;

**if** (expandTDCFCD [randInit*2] > siblinFusionDoub1){

                                                activeCellStatusList [siblingCellPosition1*12+4] = expandTDCFCD [randInit*2];

                                                activeCellStatusList [siblingCellPosition1*12+5] = 3;

                                                activeCellStatusList [siblingCellPosition2*12+6] = siblingCellNo1;

                                                activeCellStatusList [counter1*12+6] = siblingCellNo1;

                                                fusionCount++;

                                            }

**else**{

                                                activeCellStatusList [siblingCellPosition1*12+5] = 3;

                                                activeCellStatusList [siblingCellPosition2*12+6] = siblingCellNo1;

                                                activeCellStatusList [counter1*12+6] = siblingCellNo1;

                                                fusionCount++;

                                            }

                                        }

**else**{

                                            activeCellStatusList [siblingCellPosition1*12+5] = 3;

                                            activeCellStatusList [siblingCellPosition2*12+6] = siblingCellNo1;

                                            activeCellStatusList [counter1*12+6] = siblingCellNo1;

                                            fusionCount++;

                                        }

                                    }

                                }

                            }

**else** **if** (siblinFusionCheck1 != 6 && siblinFusionCheck2 != 6){

                                randInit = rand() % 1 + 0;

**if** (randInit == 1){

                                    siblingCellPositionSelect = siblingCellPosition1;

                                    siblingCellNoSelect = siblingCellNo1;

                                    siblingCellDoubSelect = siblinFusionDoub1;

                                    fusionCount++;

                                }

**else**{

                                    siblingCellPositionSelect = siblingCellPosition2;

                                    siblingCellNoSelect = siblingCellNo2;

                                    siblingCellDoubSelect = siblinFusionDoub2;

                                    fusionCount++;

                                }

                                randInit = rand() % 100 + 0;

**if** (secondEventTDCFList [randInit] == 0){

                                    activeCellStatusList [counter1*12+6] = siblingCellNoSelect;

                                    fusionCount++;

                                }

**else**{

**if** ((activeCellStatusList [counter1*12+8] == 2 || activeCellStatusList [counter1*12+8] == 3) && activeCellStatusList [counter1*12+8] == activeCellStatusList [counter1*12+7] && secondEventTDCFList [randInit] == 15){

                                        selectChange = 16;

                                    }

**else** selectChange = secondEventTDCFList [randInit];

**if** (selectChange == 14){

                                        loopCheck = 0;

**if** (expandDoublingDoubCFCount != 0){

                                            randInit = rand() % expandDoublingDoubCFCount/2 + 0;

**if** (expandDoublingDoubCF [randInit*2] > siblingCellDoubSelect){

                                                activeCellStatusList [siblingCellPositionSelect*12+4] = expandDoublingDoubCF [randInit*2];

                                                activeCellStatusList [siblingCellPositionSelect*12+5] = 1;

                                                activeCellStatusList [counter1*12+6] = siblingCellNoSelect;

                                                activeCellStatusList [siblingCellPositionSelect*12+7] = 0;

                                                activeCellStatusList [siblingCellPositionSelect*12+8] = 0;

                                                fusionCount++;

                                                loopCheck = 1;

                                            }

**else**{

                                                activeCellStatusList [siblingCellPositionSelect*12+5] = 1;

                                                activeCellStatusList [counter1*12+6] = siblingCellNoSelect;

                                                activeCellStatusList [siblingCellPositionSelect*12+7] = 0;

                                                activeCellStatusList [siblingCellPositionSelect*12+8] = 0;

                                                fusionCount++;

                                                loopCheck = 1;

                                            }

                                        }

**if** (loopCheck == 0){

                                            activeCellStatusList [siblingCellPositionSelect*12+5] = 1;

                                            activeCellStatusList [counter1*12+6] = siblingCellNoSelect;

                                            activeCellStatusList [siblingCellPositionSelect*12+7] = 0;

                                            activeCellStatusList [siblingCellPositionSelect*12+8] = 0;

                                            fusionCount++;

                                        }

                                    }

**else** **if** (selectChange == 15){

                                        loopCheck = 0;

**if** (expandDoublingDoubCFCount != 0){

                                            randInit = rand() % expandDoublingDoubCFCount/2 + 0;

**if** (expandDoublingDoubCF [randInit*2] > siblingCellDoubSelect){

**if** (activeCellStatusList [siblingCellPositionSelect*12+5] != 2) activeCellStatusList [siblingCellPositionSelect*12+7]++;

                                                activeCellStatusList [siblingCellPositionSelect*12+4] = expandDoublingDoubCF [randInit*2];

                                                activeCellStatusList [siblingCellPositionSelect*12+5] = 2;

                                                activeCellStatusList [counter1*12+6] = siblingCellNoSelect;

                                                activeCellStatusList [siblingCellPositionSelect*12+8] = (**int**)simProcessDataMiddleHold [18];

                                                fusionCount++;

                                                loopCheck = 1;

                                            }

**else**{

**if** (activeCellStatusList [siblingCellPositionSelect*12+5] != 2) activeCellStatusList [siblingCellPositionSelect*12+7]++;

                                                activeCellStatusList [siblingCellPosition1*12+5] = 2;

                                                activeCellStatusList [counter1*12+6] = siblingCellNoSelect;

                                                activeCellStatusList [siblingCellPositionSelect*12+8] = (**int**)simProcessDataMiddleHold [18];

                                                fusionCount++;

                                                loopCheck = 1;

                                            }

                                        }

**if** (loopCheck == 0){

**if** (activeCellStatusList [siblingCellPositionSelect*12+5] != 2) activeCellStatusList [siblingCellPositionSelect*12+7]++;

                                            activeCellStatusList [siblingCellPositionSelect*12+5] = 2;

                                            activeCellStatusList [counter1*12+6] = siblingCellNoSelect;

                                            activeCellStatusList [siblingCellPositionSelect*12+8] = (**int**)simProcessDataMiddleHold [18];

                                            fusionCount++;

                                        }

                                    }

**else** **if** (selectChange == 16){

**if** (expandTDCFCDCount != 0){

                                            randInit = rand() % expandTDCFCDCount/2 + 0;

**if** (expandTDCFCD [randInit*2] > siblingCellDoubSelect){

                                                activeCellStatusList [siblingCellPositionSelect*12+4] = expandTDCFCD [randInit*2];

                                                activeCellStatusList [siblingCellPositionSelect*12+5] = 3;

                                                activeCellStatusList [counter1*12+6] = siblingCellNoSelect;

                                                fusionCount++;

                                            }

**else**{

                                                activeCellStatusList [siblingCellPositionSelect*12+5] = 3;

                                                activeCellStatusList [counter1*12+6] = siblingCellNoSelect;

                                                fusionCount++;

                                            }

                                        }

**else**{

                                            activeCellStatusList [siblingCellPositionSelect*12+5] = 3;

                                            activeCellStatusList [counter1*12+6] = siblingCellNoSelect;

                                            fusionCount++;

                                        }

                                    }

                                }

                            }

                        }

                    }

                }

**else**{

**if** (activeCellStatusList [counter1*12+5] == 6){

**if** (activeCellStatusList [counter1*12+2] == 0){

                            siblinFusionDoub1 = 0;

                            siblingCellNo1 = 0;

**for** (**int** counter2 = 0; counter2 < activeCellStatusListCount/12; counter2++){

**if** (activeCellStatusList [counter2*12+1] == activeCellStatusList [counter1*12] && activeCellStatusList [counter2*12+3] == activeCellStatusList [counter1*12+3]){

                                    siblingCellNo1 = activeCellStatusList [counter2*12];

                                    siblinFusionDoub1 = activeCellStatusList [counter2*12+4];

                                    siblingCellPosition1 = counter2;

                                    fusionCount++;

**break**;

                                }

                            }

                            randInit = rand() % 100 + 0;

**if** (secondEventBDCFListSel [randInit] == 0){

                                activeCellStatusList [counter1*12+6] = siblingCellNo1;

                                fusionCount++;

                            }

**else** **if** (secondEventBDCFListSel [randInit] == 7){

                                loopCheck = 0;

**if** (expandDoublingDoubCFSelCount != 0){

                                    randInit = rand() % expandDoublingDoubCFSelCount/2 + 0;

**if** (expandDoublingDoubCFSel [randInit*2] > siblinFusionDoub1){

                                        activeCellStatusList [siblingCellPosition1*12+4] = expandDoublingDoubCFSel [randInit*2];

                                        activeCellStatusList [siblingCellPosition1*12+5] = 1;

                                        activeCellStatusList [counter1*12+6] = siblingCellNo1;

                                        activeCellStatusList [siblingCellPosition1*12+7] = 0;

                                        activeCellStatusList [siblingCellPosition1*12+8] = 0;

                                        fusionCount++;

                                        loopCheck = 1;

                                    }

**else**{

                                        activeCellStatusList [siblingCellPosition1*12+5] = 1;

                                        activeCellStatusList [counter1*12+6] = siblingCellNo1;

                                        activeCellStatusList [siblingCellPosition1*12+7] = 0;

                                        activeCellStatusList [siblingCellPosition1*12+8] = 0;

                                        fusionCount++;

                                        loopCheck = 1;

                                    }

                                }

**if** (loopCheck == 0){

                                    activeCellStatusList [siblingCellPosition1*12+5] = 1;

                                    activeCellStatusList [counter1*12+6] = siblingCellNo1;

                                    activeCellStatusList [siblingCellPosition1*12+7] = 0;

                                    activeCellStatusList [siblingCellPosition1*12+8] = 0;

                                    fusionCount++;

                                }

                            }

**else** **if** (secondEventBDCFListSel [randInit] == 8){

                                loopCheck = 0;

**if** (expandDoublingDoubCFSelCount != 0){

                                    randInit = rand() % expandDoublingDoubCFSelCount/2 + 0;

**if** (expandDoublingDoubCFSel [randInit*2] > siblinFusionDoub1){

**if** (activeCellStatusList [siblingCellPosition1*12+5] != 2) activeCellStatusList [siblingCellPosition1*12+7]++;

                                        activeCellStatusList [siblingCellPosition1*12+4] = expandDoublingDoubCFSel [randInit*2];

                                        activeCellStatusList [siblingCellPosition1*12+5] = 2;

                                        activeCellStatusList [counter1*12+6] = siblingCellNo1;

                                        activeCellStatusList [siblingCellPosition1*12+8] = (**int**)simProcessDataAddHold [18];

                                        fusionCount++;

                                        loopCheck = 1;

                                    }

**else**{

**if** (activeCellStatusList [siblingCellPosition1*12+5] != 2) activeCellStatusList [siblingCellPosition1*12+7]++;

                                        activeCellStatusList [siblingCellPosition1*12+5] = 2;

                                        activeCellStatusList [counter1*12+6] = siblingCellNo1;

                                        activeCellStatusList [siblingCellPosition1*12+8] = (**int**)simProcessDataAddHold [18];

                                        fusionCount++;

                                        loopCheck = 1;

                                    }

                                }

**if** (loopCheck == 0){

**if** (activeCellStatusList [siblingCellPosition1*12+5] != 2) activeCellStatusList [siblingCellPosition1*12+7]++;

                                    activeCellStatusList [siblingCellPosition1*12+5] = 2;

                                    activeCellStatusList [counter1*12+6] = siblingCellNo1;

                                    activeCellStatusList [siblingCellPosition1*12+8] = (**int**)simProcessDataAddHold [18];

                                    fusionCount++;

                                }

                            }

**else** **if** (secondEventBDCFListSel [randInit] == 9){

**if** (expandBDCFCDSelCount != 0){

                                    randInit = rand() % expandBDCFCDSelCount/2 + 0;

**if** (expandBDCFCDSel [randInit*2] > siblinFusionDoub1){

                                        activeCellStatusList [siblingCellPosition1*12+4] = expandBDCFCDSel [randInit*2];

                                        activeCellStatusList [siblingCellPosition1*12+5] = 3;

                                        activeCellStatusList [counter1*12+6] = siblingCellNo1;

                                        fusionCount++;

                                    }

**else**{

                                        activeCellStatusList [siblingCellPosition1*12+5] = 3;

                                        activeCellStatusList [counter1*12+6] = siblingCellNo1;

                                        fusionCount++;

                                    }

                                }

**else**{

                                    activeCellStatusList [siblingCellPosition1*12+5] = 3;

                                    activeCellStatusList [counter1*12+6] = siblingCellNo1;

                                    fusionCount++;

                                }

                            }

                        }

**else**{

                            siblinFusionCheck1 = 0;

                            siblinFusionCheck2 = 0;

                            siblinFusionDoub1 = 0;

                            siblinFusionDoub2 = 0;

                            siblingCellNo1 = 0;

                            siblingCellNo2 = 0;

**for** (**int** counter2 = 0; counter2 < activeCellStatusListCount/12; counter2++){

**if** ((activeCellStatusList [counter2*12+1] == activeCellStatusList [counter1*12] || activeCellStatusList [counter2*12+2] == activeCellStatusList [counter1*12]) && activeCellStatusList [counter2*12+3] == activeCellStatusList [counter1*12+3]){

**if** (siblinFusionCheck1 == 0 && activeCellStatusList [counter2*12+5] != 0){

                                        siblinFusionCheck1 = activeCellStatusList [counter2*12+5];

                                        siblinFusionDoub1 = activeCellStatusList [counter2*12+4];

                                        siblingCellNo1 = activeCellStatusList [counter2*12];

                                        siblingCellPosition1 = counter2;

                                    }

**else** **if** (siblinFusionCheck1 != 0 && siblinFusionCheck2 == 0 && activeCellStatusList [counter2*12+5] != 0){

                                        siblinFusionCheck2 = activeCellStatusList [counter2*12+5];

                                        siblinFusionDoub2 = activeCellStatusList [counter2*12+4];

                                        siblingCellNo2 = activeCellStatusList [counter2*12];

                                        siblingCellPosition2 = counter2;

**break**;

                                    }

                                }

                            }

**if** (siblinFusionCheck1 == 6 && siblinFusionCheck2 != 6){

                                randInit = rand() % 100 + 0;

**if** (secondEventTDCFListSel [randInit] == 0){

                                    activeCellStatusList [siblingCellPosition1*12+6] = siblingCellNo2;

                                    activeCellStatusList [counter1*12+6] = siblingCellNo2;

                                    fusionCount++;

                                }

**else**{

**if** ((activeCellStatusList [counter1*12+8] == 2 || activeCellStatusList [counter1*12+8] == 3) && activeCellStatusList [counter1*12+8] == activeCellStatusList [counter1*12+7] && secondEventTDCFListSel [randInit] == 15){

                                        selectChange = 16;

                                    }

**else** selectChange = secondEventTDCFListSel [randInit];

**if** (selectChange == 14){

                                        loopCheck = 0;

**if** (expandDoublingDoubCFSelCount != 0){

                                            randInit = rand() % expandDoublingDoubCFSelCount/2 + 0;

**if** (expandDoublingDoubCFSel [randInit*2] > siblinFusionDoub2){

                                                activeCellStatusList [siblingCellPosition2*12+4] = expandDoublingDoubCFSel [randInit*2];

                                                activeCellStatusList [siblingCellPosition2*12+5] = 1;

                                                activeCellStatusList [siblingCellPosition1*12+6] = siblingCellNo2;

                                                activeCellStatusList [counter1*12+6] = siblingCellNo2;

                                                activeCellStatusList [siblingCellPosition2*12+7] = 0;

                                                activeCellStatusList [siblingCellPosition2*12+8] = 0;

                                                fusionCount++;

                                                loopCheck = 1;

                                            }

**else**{

                                                activeCellStatusList [siblingCellPosition2*12+5] = 1;

                                                activeCellStatusList [siblingCellPosition1*12+6] = siblingCellNo2;

                                                activeCellStatusList [counter1*12+6] = siblingCellNo2;

                                                activeCellStatusList [siblingCellPosition2*12+7] = 0;

                                                activeCellStatusList [siblingCellPosition2*12+8] = 0;

                                                fusionCount++;

                                                loopCheck = 1;

                                            }

                                        }

**if** (loopCheck == 0){

                                            activeCellStatusList [siblingCellPosition2*12+5] = 1;

                                            activeCellStatusList [siblingCellPosition1*12+6] = siblingCellNo2;

                                            activeCellStatusList [counter1*12+6] = siblingCellNo2;

                                            activeCellStatusList [siblingCellPosition2*12+7] = 0;

                                            activeCellStatusList [siblingCellPosition2*12+8] = 0;

                                            fusionCount++;

                                        }

                                    }

**else** **if** (selectChange == 15){

                                        loopCheck = 0;

**if** (expandDoublingDoubCFSelCount != 0){

                                            randInit = rand() % expandDoublingDoubCFSelCount/2 + 0;

**if** (expandDoublingDoubCFSel [randInit*2] > siblinFusionDoub2){

**if** (activeCellStatusList [siblingCellPosition2*12+5] != 2) activeCellStatusList [siblingCellPosition2*12+7]++;

                                                activeCellStatusList [siblingCellPosition2*12+4] = expandDoublingDoubCFSel [randInit*2];

                                                activeCellStatusList [siblingCellPosition2*12+5] = 2;

                                                activeCellStatusList [siblingCellPosition1*12+6] = siblingCellNo2;

                                                activeCellStatusList [counter1*12+6] = siblingCellNo2;

                                                activeCellStatusList [siblingCellPosition2*12+8] = (**int**)simProcessDataAddHold [18];

                                                fusionCount++;

                                                loopCheck = 1;

                                            }

**else**{

**if** (activeCellStatusList [siblingCellPosition2*12+5] != 2) activeCellStatusList [siblingCellPosition2*12+7]++;

                                                activeCellStatusList [siblingCellPosition2*12+5] = 2;

                                                activeCellStatusList [siblingCellPosition1*12+6] = siblingCellNo2;

                                                activeCellStatusList [counter1*12+6] = siblingCellNo2;

                                                activeCellStatusList [siblingCellPosition2*12+8] = (**int**)simProcessDataAddHold [18];

                                                fusionCount++;

                                                loopCheck = 1;

                                            }

                                        }

**if** (loopCheck == 0){

**if** (activeCellStatusList [siblingCellPosition2*12+5] != 2) activeCellStatusList [siblingCellPosition2*12+7]++;

                                            activeCellStatusList [siblingCellPosition2*12+5] = 2;

                                            activeCellStatusList [siblingCellPosition1*12+6] = siblingCellNo2;

                                            activeCellStatusList [counter1*12+6] = siblingCellNo2;

                                            activeCellStatusList [siblingCellPosition2*12+8] = (**int**)simProcessDataAddHold [18];

                                            fusionCount++;

                                        }

                                    }

**else** **if** (selectChange == 16){

**if** (expandTDCFCDSelCount != 0){

                                            randInit = rand() % expandTDCFCDSelCount/2 + 0;

**if** (expandTDCFCDSel [randInit*2] > siblinFusionDoub2){

                                                activeCellStatusList [siblingCellPosition2*12+4] = expandTDCFCDSel [randInit*2];

                                                activeCellStatusList [siblingCellPosition2*12+5] = 3;

                                                activeCellStatusList [siblingCellPosition1*12+6] = siblingCellNo2;

                                                activeCellStatusList [counter1*12+6] = siblingCellNo2;

                                                fusionCount++;

                                            }

**else**{

                                                activeCellStatusList [siblingCellPosition2*12+5] = 3;

                                                activeCellStatusList [siblingCellPosition1*12+6] = siblingCellNo2;

                                                activeCellStatusList [counter1*12+6] = siblingCellNo2;

                                                fusionCount++;

                                            }

                                        }

**else**{

                                            activeCellStatusList [siblingCellPosition2*12+5] = 3;

                                            activeCellStatusList [siblingCellPosition1*12+6] = siblingCellNo2;

                                            activeCellStatusList [counter1*12+6] = siblingCellNo2;

                                            fusionCount++;

                                        }

                                    }

                                }

                            }

**else** **if** (siblinFusionCheck1 != 6 && siblinFusionCheck2 == 6){

                                randInit = rand() % 100 + 0;

**if** (secondEventTDCFListSel [randInit] == 0){

                                    activeCellStatusList [siblingCellPosition2*12+6] = siblingCellNo1;

                                    activeCellStatusList [counter1*12+6] = siblingCellNo1;

                                    fusionCount++;

                                }

**else**{

**if** ((activeCellStatusList [counter1*12+8] == 2 || activeCellStatusList [counter1*12+8] == 3) && activeCellStatusList [counter1*12+8] == activeCellStatusList [counter1*12+7] && secondEventTDCFListSel [randInit] == 15){

                                        selectChange = 16;

                                    }

**else** selectChange = secondEventTDCFListSel [randInit];

**if** (selectChange == 14){

                                        loopCheck = 0;

**if** (expandDoublingDoubCFSelCount != 0){

                                            randInit = rand() % expandDoublingDoubCFSelCount/2 + 0;

**if** (expandDoublingDoubCFSel [randInit*2] > siblinFusionDoub1){

                                                activeCellStatusList [siblingCellPosition1*12+4] = expandDoublingDoubCFSel [randInit*2];

                                                activeCellStatusList [siblingCellPosition1*12+5] = 1;

                                                activeCellStatusList [siblingCellPosition2*12+6] = siblingCellNo1;

                                                activeCellStatusList [counter1*12+6] = siblingCellNo1;

                                                activeCellStatusList [siblingCellPosition1*12+7] = 0;

                                                activeCellStatusList [siblingCellPosition1*12+8] = 0;

                                                fusionCount++;

                                                loopCheck = 1;

                                            }

**else**{

                                                activeCellStatusList [siblingCellPosition1*12+5] = 1;

                                                activeCellStatusList [siblingCellPosition2*12+6] = siblingCellNo1;

                                                activeCellStatusList [counter1*12+6] = siblingCellNo1;

                                                activeCellStatusList [siblingCellPosition1*12+7] = 0;

                                                activeCellStatusList [siblingCellPosition1*12+8] = 0;

                                                fusionCount++;

                                                loopCheck = 1;

                                            }

                                        }

**if** (loopCheck == 0){

                                            activeCellStatusList [siblingCellPosition1*12+5] = 1;

                                            activeCellStatusList [siblingCellPosition2*12+6] = siblingCellNo1;

                                            activeCellStatusList [counter1*12+6] = siblingCellNo1;

                                            activeCellStatusList [siblingCellPosition1*12+7] = 0;

                                            activeCellStatusList [siblingCellPosition1*12+8] = 0;

                                            fusionCount++;

                                        }

                                    }

**else** **if** (selectChange == 15){

                                        loopCheck = 0;

**if** (expandDoublingDoubCFSelCount != 0){

                                            randInit = rand() % expandDoublingDoubCFSelCount/2 + 0;

**if** (expandDoublingDoubCFSel [randInit*2] > siblinFusionDoub1){

**if** (activeCellStatusList [siblingCellPosition1*12+5] != 2) activeCellStatusList [siblingCellPosition1*12+7]++;

                                                activeCellStatusList [siblingCellPosition1*12+4] = expandDoublingDoubCFSel [randInit*2];

                                                activeCellStatusList [siblingCellPosition1*12+5] = 2;

                                                activeCellStatusList [siblingCellPosition2*12+6] = siblingCellNo1;

                                                activeCellStatusList [counter1*12+6] = siblingCellNo1;

                                                activeCellStatusList [siblingCellPosition1*12+8] = (**int**)simProcessDataAddHold [18];

                                                fusionCount++;

                                                loopCheck = 1;

                                            }

**else**{

**if** (activeCellStatusList [siblingCellPosition1*12+5] != 2) activeCellStatusList [siblingCellPosition1*12+7]++;

                                                activeCellStatusList [siblingCellPosition1*12+5] = 2;

                                                activeCellStatusList [siblingCellPosition2*12+6] = siblingCellNo1;

                                                activeCellStatusList [counter1*12+6] = siblingCellNo1;

                                                activeCellStatusList [siblingCellPosition1*12+8] = (**int**)simProcessDataAddHold [18];

                                                fusionCount++;

                                                loopCheck = 1;

                                            }

                                        }

**if** (loopCheck == 0){

**if** (activeCellStatusList [siblingCellPosition1*12+5] != 2) activeCellStatusList [siblingCellPosition1*12+7]++;

                                            activeCellStatusList [siblingCellPosition1*12+5] = 2;

                                            activeCellStatusList [siblingCellPosition2*12+6] = siblingCellNo1;

                                            activeCellStatusList [counter1*12+6] = siblingCellNo1;

                                            activeCellStatusList [siblingCellPosition1*12+8] = (**int**)simProcessDataAddHold [18];

                                            fusionCount++;

                                        }

                                    }

**else** **if** (selectChange == 16){

**if** (expandTDCFCDSelCount != 0){

                                            randInit = rand() % expandTDCFCDSelCount/2 + 0;

**if** (expandTDCFCD [randInit*2] > siblinFusionDoub1){

                                                activeCellStatusList [siblingCellPosition1*12+4] = expandTDCFCDSel [randInit*2];

                                                activeCellStatusList [siblingCellPosition1*12+5] = 3;

                                                activeCellStatusList [siblingCellPosition2*12+6] = siblingCellNo1;

                                                activeCellStatusList [counter1*12+6] = siblingCellNo1;

                                                fusionCount++;

                                            }

**else**{

                                                activeCellStatusList [siblingCellPosition1*12+5] = 3;

                                                activeCellStatusList [siblingCellPosition2*12+6] = siblingCellNo1;

                                                activeCellStatusList [counter1*12+6] = siblingCellNo1;

                                                fusionCount++;

                                            }

                                        }

**else**{

                                            activeCellStatusList [siblingCellPosition1*12+5] = 3;

                                            activeCellStatusList [siblingCellPosition2*12+6] = siblingCellNo1;

                                            activeCellStatusList [counter1*12+6] = siblingCellNo1;

                                            fusionCount++;

                                        }

                                    }

                                }

                            }

**else** **if** (siblinFusionCheck1 != 6 && siblinFusionCheck2 != 6){

                                randInit = rand() % 1 + 0;

**if** (randInit == 1){

                                    siblingCellPositionSelect = siblingCellPosition1;

                                    siblingCellNoSelect = siblingCellNo1;

                                    siblingCellDoubSelect = siblinFusionDoub1;

                                    fusionCount++;

                                }

**else**{

                                    siblingCellPositionSelect = siblingCellPosition2;

                                    siblingCellNoSelect = siblingCellNo2;

                                    siblingCellDoubSelect = siblinFusionDoub2;

                                    fusionCount++;

                                }

                                randInit = rand() % 100 + 0;

**if** (secondEventTDCFListSel [randInit] == 0){

                                    activeCellStatusList [counter1*12+6] = siblingCellNoSelect;

                                    fusionCount++;

                                }

**else**{

**if** ((activeCellStatusList [counter1*12+8] == 2 || activeCellStatusList [counter1*12+8] == 3) && activeCellStatusList [counter1*12+8] == activeCellStatusList [counter1*12+7] && secondEventTDCFListSel [randInit] == 15){

                                        selectChange = 16;

                                    }

**else** selectChange = secondEventTDCFListSel [randInit];

**if** (selectChange == 14){

                                        loopCheck = 0;

**if** (expandDoublingDoubCFSelCount != 0){

                                            randInit = rand() % expandDoublingDoubCFSelCount/2 + 0;

**if** (expandDoublingDoubCFSel [randInit*2] > siblingCellDoubSelect){

                                                activeCellStatusList [siblingCellPositionSelect*12+4] = expandDoublingDoubCFSel [randInit*2];

                                                activeCellStatusList [siblingCellPositionSelect*12+5] = 1;

                                                activeCellStatusList [counter1*12+6] = siblingCellNoSelect;

                                                activeCellStatusList [siblingCellPositionSelect*12+7] = 0;

                                                activeCellStatusList [siblingCellPositionSelect*12+8] = 0;

                                                fusionCount++;

                                                loopCheck = 1;

                                            }

**else**{

                                                activeCellStatusList [siblingCellPositionSelect*12+5] = 1;

                                                activeCellStatusList [counter1*12+6] = siblingCellNoSelect;

                                                activeCellStatusList [siblingCellPositionSelect*12+7] = 0;

                                                activeCellStatusList [siblingCellPositionSelect*12+8] = 0;

                                                fusionCount++;

                                                loopCheck = 1;

                                            }

                                        }

**if** (loopCheck == 0){

                                            activeCellStatusList [siblingCellPositionSelect*12+5] = 1;

                                            activeCellStatusList [counter1*12+6] = siblingCellNoSelect;

                                            activeCellStatusList [siblingCellPositionSelect*12+7] = 0;

                                            activeCellStatusList [siblingCellPositionSelect*12+8] = 0;

                                            fusionCount++;

                                        }

                                    }

**else** **if** (selectChange == 15){

                                        loopCheck = 0;

**if** (expandDoublingDoubCFSelCount != 0){

                                            randInit = rand() % expandDoublingDoubCFSelCount/2 + 0;

**if** (expandDoublingDoubCFSel [randInit*2] > siblingCellDoubSelect){

**if** (activeCellStatusList [siblingCellPositionSelect*12+5] != 2) activeCellStatusList [siblingCellPositionSelect*12+7]++;

                                                activeCellStatusList [siblingCellPositionSelect*12+4] = expandDoublingDoubCFSel [randInit*2];

                                                activeCellStatusList [siblingCellPositionSelect*12+5] = 2;

                                                activeCellStatusList [counter1*12+6] = siblingCellNoSelect;

                                                activeCellStatusList [siblingCellPositionSelect*12+8] = (**int**)simProcessDataAddHold [18];

                                                fusionCount++;

                                                loopCheck = 1;

                                            }

**else**{

**if** (activeCellStatusList [siblingCellPositionSelect*12+5] != 2) activeCellStatusList [siblingCellPositionSelect*12+7]++;

                                                activeCellStatusList [siblingCellPosition1*12+5] = 2;

                                                activeCellStatusList [counter1*12+6] = siblingCellNoSelect;

                                                activeCellStatusList [siblingCellPositionSelect*12+8] = (**int**)simProcessDataAddHold [18];

                                                fusionCount++;

                                                loopCheck = 1;

                                            }

                                        }

**if** (loopCheck == 0){

**if** (activeCellStatusList [siblingCellPositionSelect*12+5] != 2) activeCellStatusList [siblingCellPositionSelect*12+7]++;

                                            activeCellStatusList [siblingCellPositionSelect*12+5] = 2;

                                            activeCellStatusList [counter1*12+6] = siblingCellNoSelect;

                                            activeCellStatusList [siblingCellPositionSelect*12+8] = (**int**)simProcessDataAddHold [18];

                                            fusionCount++;

                                        }

                                    }

**else** **if** (selectChange == 16){

**if** (expandTDCFCDSelCount != 0){

                                            randInit = rand() % expandTDCFCDSelCount/2 + 0;

**if** (expandTDCFCDSel [randInit*2] > siblingCellDoubSelect){

                                                activeCellStatusList [siblingCellPositionSelect*12+4] = expandTDCFCDSel [randInit*2];

                                                activeCellStatusList [siblingCellPositionSelect*12+5] = 3;

                                                activeCellStatusList [counter1*12+6] = siblingCellNoSelect;

                                                fusionCount++;

                                            }

**else**{

                                                activeCellStatusList [siblingCellPositionSelect*12+5] = 3;

                                                activeCellStatusList [counter1*12+6] = siblingCellNoSelect;

                                                fusionCount++;

                                            }

                                        }

**else**{

                                            activeCellStatusList [siblingCellPositionSelect*12+5] = 3;

                                            activeCellStatusList [counter1*12+6] = siblingCellNoSelect;

                                            fusionCount++;

                                        }

                                    }

                                }

                            }

                        }

                    }

                }

            }

**if** (terminate2 == 0){

**int** *lineageAddTemp = **new** **int** [10000000];

                lineageAddTempLimit = 10000000;

**int** *tempListOfCells = **new** **int** [10000000];

**unsigned** **long** tempListOfCellsCount = 0;

**unsigned** **long** tempListOfCellsLimit = 10000000;

**long** *fusionPartnerList = **new** **long** [fusionCount*5+100];

                fusionPartnerListCount = 0;

**long** *startPositionList = **new** **long** [(activeCellStatusListCount/12)*3+10];

                startPositionListCount = 0;

**for** (**int** counter1 = 0; counter1 < activeCellStatusListCount/12; counter1++){

**if** (terminateSimFlag == 1){

                        terminate2 = 1;

**break**;

                    }

                    processingStatusCall2 = 1;

                    processingStatus2 = to_string(counter1)+"/"+to_string(activeCellStatusListCount/12);

                    lineageAddTempCount = 0;

                    timeKeep = 0;

                    cellNoFusionCheckHold = -1;

**if** (activeCellStatusList [counter1*12+5] == 5 || activeCellStatusList [counter1*12+5] == 9 || activeCellStatusList [counter1*12+5] == 70 || activeCellStatusList [counter1*12+5] == 30){

**for** (**unsigned** **long** counter2 = 0; counter2 < cellLineageSummaryArrayCount/9; counter2++){

**if** (cellLineageSummaryArray [counter2*9+2] == activeCellStatusList [counter1*12+3] && cellLineageSummaryArray [counter2*9+3] == activeCellStatusList [counter1*12]){

**for** (**unsigned** **long** counter3 = (**unsigned** **long**)cellLineageSummaryArray [counter2*9]/9; counter3 <= cellLineageSummaryArray [counter2*9+1]/9; counter3++){

**if** (lineageAddTempCount+9 > lineageAddTempLimit){

**int** *arrayUpDate = **new** **int** [lineageAddTempCount+10];

**for** (**int** counter4 = 0; counter4 < lineageAddTempCount; counter4++) arrayUpDate [counter4] = lineageAddTemp [counter4];

**delete** [] lineageAddTemp;

                                        lineageAddTemp = **new** **int** [lineageAddTempLimit+1000000];

                                        lineageAddTempLimit = lineageAddTempLimit+1000000;

**for** (**int** counter4 = 0; counter4 < lineageAddTempCount; counter4++) lineageAddTemp [counter4] = arrayUpDate [counter4];

**delete** [] arrayUpDate;

                                    }

                                    lineageAddTemp [lineageAddTempCount] = cellLineageTempArray [counter3*9], lineageAddTempCount++;

                                    lineageAddTemp [lineageAddTempCount] = cellLineageTempArray [counter3*9+1], lineageAddTempCount++;

                                    lineageAddTemp [lineageAddTempCount] = cellLineageTempArray [counter3*9+2], lineageAddTempCount++;

                                    lineageAddTemp [lineageAddTempCount] = cellLineageTempArray [counter3*9+3], lineageAddTempCount++;

                                    lineageAddTemp [lineageAddTempCount] = cellLineageTempArray [counter3*9+4], lineageAddTempCount++;

                                    lineageAddTemp [lineageAddTempCount] = cellLineageTempArray [counter3*9+5], lineageAddTempCount++;

                                    lineageAddTemp [lineageAddTempCount] = cellLineageTempArray [counter3*9+6], lineageAddTempCount++;

                                    lineageAddTemp [lineageAddTempCount] = cellLineageTempArray [counter3*9+7], lineageAddTempCount++;

                                    lineageAddTemp [lineageAddTempCount] = cellLineageTempArray [counter3*9+8], lineageAddTempCount++;

                                    timeKeep = cellLineageTempArray [counter3*9+2];

                                    cellLineageTempArray [counter3*9+8] = -1;

**if** (activeCellStatusList [counter1*12+5] == 70) activeCellStatusList [counter1*12+5] = 7;

**else** **if** (activeCellStatusList [counter1*12+5] == 30) activeCellStatusList [counter1*12+5] = 3;

                                }

**break**;

                            }

                        }

                    }

**else**{

**for** (**unsigned** **long** counter2 = 0; counter2 < cellLineageSummaryArrayCount/9; counter2++){

**if** (cellLineageSummaryArray [counter2*9+2] == activeCellStatusList [counter1*12+3] && cellLineageSummaryArray [counter2*9+3] == activeCellStatusList [counter1*12]){

**if** (lineageAddTempCount+9 > lineageAddTempLimit){

**int** *arrayUpDate = **new** **int** [lineageAddTempCount+10];

**for** (**int** counter4 = 0; counter4 < lineageAddTempCount; counter4++) arrayUpDate [counter4] = lineageAddTemp [counter4];

**delete** [] lineageAddTemp;

                                    lineageAddTemp = **new** **int** [lineageAddTempLimit+1000000];

                                    lineageAddTempLimit = lineageAddTempLimit+1000000;

**for** (**int** counter4 = 0; counter4 < lineageAddTempCount; counter4++) lineageAddTemp [counter4] = arrayUpDate [counter4];

**delete** [] arrayUpDate;

                                }

                                setTime = (**unsigned** **long** )(cellLineageSummaryArray [counter2*9]);

                                lineageAddTemp [lineageAddTempCount] = cellLineageTempArray [setTime], lineageAddTempCount++;

                                lineageAddTemp [lineageAddTempCount] = cellLineageTempArray [setTime+1], lineageAddTempCount++;

                                lineageAddTemp [lineageAddTempCount] = cellLineageTempArray [setTime+2], lineageAddTempCount++;

                                lineageAddTemp [lineageAddTempCount] = cellLineageTempArray [setTime+3], lineageAddTempCount++;

                                lineageAddTemp [lineageAddTempCount] = cellLineageTempArray [setTime+4], lineageAddTempCount++;

                                lineageAddTemp [lineageAddTempCount] = cellLineageTempArray [setTime+5], lineageAddTempCount++;

                                lineageAddTemp [lineageAddTempCount] = cellLineageTempArray [setTime+6], lineageAddTempCount++;

                                lineageAddTemp [lineageAddTempCount] = cellLineageTempArray [setTime+7], lineageAddTempCount++;

                                lineageAddTemp [lineageAddTempCount] = cellLineageTempArray [setTime+8], lineageAddTempCount++;

                                timeKeep = cellLineageTempArray [setTime+2];

                                cellLineageTempArray [setTime+8] = -1;

**break**;

                            }

                        }

                    }

**if** (activeCellStatusList [counter1*12+4]+timeKeep > growthCycleBase) extendEnd = growthCycleBase-timeKeep;

**else** extendEnd = (**int**)activeCellStatusList [counter1*12+4];

**if** (timeKeep <= growthCycleBase){

                        timeKeep++;

**if** (activeCellStatusListKeepCount+100 > activeCellStatusListKeepLimit){

**int** *arrayUpDate = **new** **int** [activeCellStatusListKeepCount+50];

**for** (**int** counter3 = 0; counter3 < activeCellStatusListKeepCount; counter3++) arrayUpDate [counter3] = activeCellStatusListKeep [counter3];

**delete** [] activeCellStatusListKeep;

                            activeCellStatusListKeep = **new** **int** [activeCellStatusListKeepLimit+100000];

                            activeCellStatusListKeepLimit = activeCellStatusListKeepLimit+100000;

**for** (**int** counter3 = 0; counter3 < activeCellStatusListKeepCount; counter3++) activeCellStatusListKeep [counter3] = arrayUpDate [counter3];

**delete** [] arrayUpDate;

                        }

**for** (**int** counter2 = 0; counter2 < extendEnd; counter2++){

**if** (lineageAddTempCount+9 > lineageAddTempLimit){

**int** *arrayUpDate = **new** **int** [lineageAddTempCount+10];

**for** (**int** counter3 = 0; counter3 < lineageAddTempCount; counter3++) arrayUpDate [counter3] = lineageAddTemp [counter3];

**delete** [] lineageAddTemp;

                                lineageAddTemp = **new** **int** [lineageAddTempLimit+1000000];

                                lineageAddTempLimit = lineageAddTempLimit+1000000;

**for** (**int** counter3 = 0; counter3 < lineageAddTempCount; counter3++) lineageAddTemp [counter3] = arrayUpDate [counter3];

**delete** [] arrayUpDate;

                            }

                            lineageAddTemp [lineageAddTempCount] = 1, lineageAddTempCount++;

                            lineageAddTemp [lineageAddTempCount] = 1, lineageAddTempCount++;

                            lineageAddTemp [lineageAddTempCount] = timeKeep, lineageAddTempCount++;

                            lineageAddTemp [lineageAddTempCount] = 2, lineageAddTempCount++;

                            lineageAddTemp [lineageAddTempCount] = 0, lineageAddTempCount++;

                            lineageAddTemp [lineageAddTempCount] = activeCellStatusList [counter1*12], lineageAddTempCount++;

                            lineageAddTemp [lineageAddTempCount] = activeCellStatusList [counter1*12+3], lineageAddTempCount++;

                            lineageAddTemp [lineageAddTempCount] = 0, lineageAddTempCount++;

                            lineageAddTemp [lineageAddTempCount] = 0, lineageAddTempCount++;

**if** (timeKeep == growthCycleBase){

                                activeCellStatusListKeep [activeCellStatusListKeepCount] = activeCellStatusListHold [counter1*12], activeCellStatusListKeepCount++;

                                activeCellStatusListKeep [activeCellStatusListKeepCount] = activeCellStatusListHold [counter1*12+1], activeCellStatusListKeepCount++;

                                activeCellStatusListKeep [activeCellStatusListKeepCount] = activeCellStatusListHold [counter1*12+2], activeCellStatusListKeepCount++;

                                activeCellStatusListKeep [activeCellStatusListKeepCount] = activeCellStatusListHold [counter1*12+3], activeCellStatusListKeepCount++;

                                activeCellStatusListKeep [activeCellStatusListKeepCount] = activeCellStatusListHold [counter1*12+4], activeCellStatusListKeepCount++;

                                activeCellStatusListKeep [activeCellStatusListKeepCount] = activeCellStatusListHold [counter1*12+5], activeCellStatusListKeepCount++;

                                activeCellStatusListKeep [activeCellStatusListKeepCount] = activeCellStatusListHold [counter1*12+6], activeCellStatusListKeepCount++;

                                activeCellStatusListKeep [activeCellStatusListKeepCount] = activeCellStatusListHold [counter1*12+7], activeCellStatusListKeepCount++;

                                activeCellStatusListKeep [activeCellStatusListKeepCount] = activeCellStatusListHold [counter1*12+8], activeCellStatusListKeepCount++;

                                activeCellStatusListKeep [activeCellStatusListKeepCount] = activeCellStatusListHold [counter1*12+9], activeCellStatusListKeepCount++;

                                activeCellStatusListKeep [activeCellStatusListKeepCount] = activeCellStatusListHold [counter1*12+10], activeCellStatusListKeepCount++;

                                activeCellStatusListKeep [activeCellStatusListKeepCount] = activeCellStatusListHold [counter1*12+11], activeCellStatusListKeepCount++;

                                cellNoFusionCheckHold = activeCellStatusList [counter1*12];

                                activeCellStatusList [counter1*12+1] = -1;

                                timeKeep++;

**break**;

                            }

**else** timeKeep++;

                        }

**if** (activeCellStatusList [counter1*12+5] == 9){

                            cellNoFusionCheckHold = activeCellStatusList [counter1*12];

                            activeCellStatusList [counter1*12+1] = -1;

**if** (reachMaxDivision > timeKeep) reachMaxDivision = timeKeep;

                        }

**if** (activeCellStatusList [counter1*12+5] == 5 && activeCellStatusList [counter1*12+1] != -1){

                            cellNoFusionCheckHold = activeCellStatusList [counter1*12];

                        }

**if** (tempListOfCellsCount+20 > tempListOfCellsLimit){

**int** *arrayUpDate = **new** **int** [tempListOfCellsCount+10];

**for** (**int** counter2 = 0; counter2 < tempListOfCellsCount; counter2++) arrayUpDate [counter2] = tempListOfCells [counter2];

**delete** [] tempListOfCells;

                            tempListOfCells = **new** **int** [tempListOfCellsLimit+1000000];

                            tempListOfCellsLimit = tempListOfCellsLimit+1000000;

**for** (**int** counter2 = 0; counter2 < tempListOfCellsCount; counter2++) tempListOfCells [counter2] = arrayUpDate [counter2];

**delete** [] arrayUpDate;

                        }

**if** (lineageAddTempCount+50 > lineageAddTempLimit){

**int** *arrayUpDate = **new** **int** [lineageAddTempCount+10];

**for** (**int** counter3 = 0; counter3 < lineageAddTempCount; counter3++) arrayUpDate [counter3] = lineageAddTemp [counter3];

**delete** [] lineageAddTemp;

                            lineageAddTemp = **new** **int** [lineageAddTempLimit+1000000];

                            lineageAddTempLimit = lineageAddTempLimit+1000000;

**for** (**int** counter3 = 0; counter3 < lineageAddTempCount; counter3++) lineageAddTemp [counter3] = arrayUpDate [counter3];

**delete** [] arrayUpDate;

                        }

**if** (cellNoLingNoListCount+10 > cellNoLingNoListLimit){

**int** *arrayUpDate = **new** **int** [cellNoLingNoListCount+10];

**for** (**int** counter3 = 0; counter3 < cellNoLingNoListCount; counter3++) arrayUpDate [counter3] = cellNoLingNoList [counter3];

**delete** [] cellNoLingNoList;

                            cellNoLingNoList = **new** **int** [cellNoLingNoListLimit+100000];

                            cellNoLingNoListLimit = cellNoLingNoListLimit+100000;

**for** (**int** counter3 = 0; counter3 < cellNoLingNoListCount; counter3++) cellNoLingNoList [counter3] = arrayUpDate [counter3];

**delete** [] arrayUpDate;

                        }

**if** (activeCellStatusList [counter1*12+5] == 1 || activeCellStatusList [counter1*12+5] == 7){

**if** (timeKeep+2 <= growthCycleBase){

                                lineageAddTemp [lineageAddTempCount] = 1, lineageAddTempCount++;

                                lineageAddTemp [lineageAddTempCount] = 1, lineageAddTempCount++;

                                lineageAddTemp [lineageAddTempCount] = timeKeep, lineageAddTempCount++;

                                lineageAddTemp [lineageAddTempCount] = 6, lineageAddTempCount++;

                                lineageAddTemp [lineageAddTempCount] = 0, lineageAddTempCount++;

                                lineageAddTemp [lineageAddTempCount] = activeCellStatusList [counter1*12], lineageAddTempCount++;

                                lineageAddTemp [lineageAddTempCount] = activeCellStatusList [counter1*12+3], lineageAddTempCount++;

                                lineageAddTemp [lineageAddTempCount] = 0, lineageAddTempCount++;

                                lineageAddTemp [lineageAddTempCount] = 0, lineageAddTempCount++;

                                lineageAddTemp [lineageAddTempCount] = 1, lineageAddTempCount++;

                                lineageAddTemp [lineageAddTempCount] = 1, lineageAddTempCount++;

                                lineageAddTemp [lineageAddTempCount] = timeKeep+1, lineageAddTempCount++;

                                lineageAddTemp [lineageAddTempCount] = 32, lineageAddTempCount++;

                                lineageAddTemp [lineageAddTempCount] = 0, lineageAddTempCount++;

                                lineageAddTemp [lineageAddTempCount] = activeCellStatusList [counter1*12], lineageAddTempCount++;

                                lineageAddTemp [lineageAddTempCount] = activeCellStatusList [counter1*12+3], lineageAddTempCount++;

                                lineageAddTemp [lineageAddTempCount] = 0, lineageAddTempCount++;

                                lineageAddTemp [lineageAddTempCount] = 0, lineageAddTempCount++;

                                createNewCellNo = [[CreateNewCellNo alloc] init];

                                newCellNumber1 = [createNewCellNo cellNumberAddition:counter1];

                                [CreateNewCellNo release];

                                cellNoLingNoList [cellNoLingNoListCount] = activeCellStatusList [counter1*12+3], cellNoLingNoListCount++;

                                cellNoLingNoList [cellNoLingNoListCount] = newCellNumber1, cellNoLingNoListCount++;

                                createNewCellNo = [[CreateNewCellNo alloc] init];

                                newCellNumber2 = [createNewCellNo cellNumberSubtraction:counter1];

                                [CreateNewCellNo release];

                                cellNoLingNoList [cellNoLingNoListCount] = activeCellStatusList [counter1*12+3], cellNoLingNoListCount++;

                                cellNoLingNoList [cellNoLingNoListCount] = newCellNumber2, cellNoLingNoListCount++;

                                lineageAddTemp [lineageAddTempCount] = 1, lineageAddTempCount++;

                                lineageAddTemp [lineageAddTempCount] = 1, lineageAddTempCount++;

                                lineageAddTemp [lineageAddTempCount] = timeKeep+2, lineageAddTempCount++;

                                lineageAddTemp [lineageAddTempCount] = 31, lineageAddTempCount++;

                                lineageAddTemp [lineageAddTempCount] = activeCellStatusList [counter1*12], lineageAddTempCount++;

                                lineageAddTemp [lineageAddTempCount] = newCellNumber1, lineageAddTempCount++;

                                lineageAddTemp [lineageAddTempCount] = activeCellStatusList [counter1*12+3], lineageAddTempCount++;

                                lineageAddTemp [lineageAddTempCount] = 0, lineageAddTempCount++;

                                lineageAddTemp [lineageAddTempCount] = 0, lineageAddTempCount++;

                                lineageAddTemp [lineageAddTempCount] = 1, lineageAddTempCount++;

                                lineageAddTemp [lineageAddTempCount] = 1, lineageAddTempCount++;

                                lineageAddTemp [lineageAddTempCount] = timeKeep+2, lineageAddTempCount++;

                                lineageAddTemp [lineageAddTempCount] = 31, lineageAddTempCount++;

                                lineageAddTemp [lineageAddTempCount] = activeCellStatusList [counter1*12], lineageAddTempCount++;

                                lineageAddTemp [lineageAddTempCount] = newCellNumber2, lineageAddTempCount++;

                                lineageAddTemp [lineageAddTempCount] = activeCellStatusList [counter1*12+3], lineageAddTempCount++;

                                lineageAddTemp [lineageAddTempCount] = 0, lineageAddTempCount++;

                                lineageAddTemp [lineageAddTempCount] = 0, lineageAddTempCount++;

**if** (timeKeep+2 < growthCycleBase){

                                    tempListOfCells [tempListOfCellsCount] = activeCellStatusList [counter1*12+3], tempListOfCellsCount++;

                                    tempListOfCells [tempListOfCellsCount] = activeCellStatusList [counter1*12], tempListOfCellsCount++;

                                    tempListOfCells [tempListOfCellsCount] = newCellNumber1, tempListOfCellsCount++;

                                    tempListOfCells [tempListOfCellsCount] = newCellNumber2, tempListOfCellsCount++;

                                    tempListOfCells [tempListOfCellsCount] = 0, tempListOfCellsCount++;

                                    tempListOfCells [tempListOfCellsCount] = activeCellStatusList [counter1*12+5], tempListOfCellsCount++;

                                    tempListOfCells [tempListOfCellsCount] = activeCellStatusList [counter1*12+4], tempListOfCellsCount++;

                                }

**else**{

                                    activeCellStatusListKeep [activeCellStatusListKeepCount] = newCellNumber1, activeCellStatusListKeepCount++;

                                    activeCellStatusListKeep [activeCellStatusListKeepCount] = newCellNumber2, activeCellStatusListKeepCount++;

                                    activeCellStatusListKeep [activeCellStatusListKeepCount] = 0, activeCellStatusListKeepCount++;

                                    activeCellStatusListKeep [activeCellStatusListKeepCount] = activeCellStatusList [counter1*12+3], activeCellStatusListKeepCount++;

                                    activeCellStatusListKeep [activeCellStatusListKeepCount] = 0, activeCellStatusListKeepCount++;

                                    activeCellStatusListKeep [activeCellStatusListKeepCount] = 1, activeCellStatusListKeepCount++;

                                    activeCellStatusListKeep [activeCellStatusListKeepCount] = 0, activeCellStatusListKeepCount++;

                                    activeCellStatusListKeep [activeCellStatusListKeepCount] = activeCellStatusList [counter1*12+7], activeCellStatusListKeepCount++;

                                    activeCellStatusListKeep [activeCellStatusListKeepCount] = activeCellStatusList [counter1*12+8], activeCellStatusListKeepCount++;

                                    activeCellStatusListKeep [activeCellStatusListKeepCount] = activeCellStatusList [counter1*12+9], activeCellStatusListKeepCount++;

                                    activeCellStatusListKeep [activeCellStatusListKeepCount] = activeCellStatusList [counter1*12], activeCellStatusListKeepCount++;

                                    activeCellStatusListKeep [activeCellStatusListKeepCount] = activeCellStatusList [counter1*12+4], activeCellStatusListKeepCount++;

                                    activeCellStatusListKeep [activeCellStatusListKeepCount] = newCellNumber2, activeCellStatusListKeepCount++;

                                    activeCellStatusListKeep [activeCellStatusListKeepCount] = newCellNumber1, activeCellStatusListKeepCount++;

                                    activeCellStatusListKeep [activeCellStatusListKeepCount] = 0, activeCellStatusListKeepCount++;

                                    activeCellStatusListKeep [activeCellStatusListKeepCount] = activeCellStatusList [counter1*12+3], activeCellStatusListKeepCount++;

                                    activeCellStatusListKeep [activeCellStatusListKeepCount] = 0, activeCellStatusListKeepCount++;

                                    activeCellStatusListKeep [activeCellStatusListKeepCount] = 1, activeCellStatusListKeepCount++;

                                    activeCellStatusListKeep [activeCellStatusListKeepCount] = 0, activeCellStatusListKeepCount++;

                                    activeCellStatusListKeep [activeCellStatusListKeepCount] = activeCellStatusList [counter1*12+7], activeCellStatusListKeepCount++;

                                    activeCellStatusListKeep [activeCellStatusListKeepCount] = activeCellStatusList [counter1*12+8], activeCellStatusListKeepCount++;

                                    activeCellStatusListKeep [activeCellStatusListKeepCount] = activeCellStatusList [counter1*12+9], activeCellStatusListKeepCount++;

                                    activeCellStatusListKeep [activeCellStatusListKeepCount] = activeCellStatusList [counter1*12], activeCellStatusListKeepCount++;

                                    activeCellStatusListKeep [activeCellStatusListKeepCount] = activeCellStatusList [counter1*12+4], activeCellStatusListKeepCount++;

                                }

                                cellNoFusionCheckHold = activeCellStatusList [counter1*12];

                                activeCellStatusList [counter1*12+1] = -1;

                            }

**else** **if** (timeKeep+1 == growthCycleBase){

                                lineageAddTemp [lineageAddTempCount] = 1, lineageAddTempCount++;

                                lineageAddTemp [lineageAddTempCount] = 1, lineageAddTempCount++;

                                lineageAddTemp [lineageAddTempCount] = timeKeep, lineageAddTempCount++;

                                lineageAddTemp [lineageAddTempCount] = 2, lineageAddTempCount++;

                                lineageAddTemp [lineageAddTempCount] = 0, lineageAddTempCount++;

                                lineageAddTemp [lineageAddTempCount] = activeCellStatusList [counter1*12], lineageAddTempCount++;

                                lineageAddTemp [lineageAddTempCount] = activeCellStatusList [counter1*12+3], lineageAddTempCount++;

                                lineageAddTemp [lineageAddTempCount] = 0, lineageAddTempCount++;

                                lineageAddTemp [lineageAddTempCount] = 0, lineageAddTempCount++;

                                lineageAddTemp [lineageAddTempCount] = 1, lineageAddTempCount++;

                                lineageAddTemp [lineageAddTempCount] = 1, lineageAddTempCount++;

                                lineageAddTemp [lineageAddTempCount] = timeKeep+1, lineageAddTempCount++;

                                lineageAddTemp [lineageAddTempCount] = 2, lineageAddTempCount++;

                                lineageAddTemp [lineageAddTempCount] = 0, lineageAddTempCount++;

                                lineageAddTemp [lineageAddTempCount] = activeCellStatusList [counter1*12], lineageAddTempCount++;

                                lineageAddTemp [lineageAddTempCount] = activeCellStatusList [counter1*12+3], lineageAddTempCount++;

                                lineageAddTemp [lineageAddTempCount] = 0, lineageAddTempCount++;

                                lineageAddTemp [lineageAddTempCount] = 0, lineageAddTempCount++;

                                activeCellStatusListKeep [activeCellStatusListKeepCount] = activeCellStatusListHold [counter1*12], activeCellStatusListKeepCount++;

                                activeCellStatusListKeep [activeCellStatusListKeepCount] = activeCellStatusListHold [counter1*12+1], activeCellStatusListKeepCount++;

                                activeCellStatusListKeep [activeCellStatusListKeepCount] = activeCellStatusListHold [counter1*12+2], activeCellStatusListKeepCount++;

                                activeCellStatusListKeep [activeCellStatusListKeepCount] = activeCellStatusListHold [counter1*12+3], activeCellStatusListKeepCount++;

                                activeCellStatusListKeep [activeCellStatusListKeepCount] = activeCellStatusListHold [counter1*12+4], activeCellStatusListKeepCount++;

                                activeCellStatusListKeep [activeCellStatusListKeepCount] = activeCellStatusListHold [counter1*12+5], activeCellStatusListKeepCount++;

                                activeCellStatusListKeep [activeCellStatusListKeepCount] = activeCellStatusListHold [counter1*12+6], activeCellStatusListKeepCount++;

                                activeCellStatusListKeep [activeCellStatusListKeepCount] = activeCellStatusListHold [counter1*12+7], activeCellStatusListKeepCount++;

                                activeCellStatusListKeep [activeCellStatusListKeepCount] = activeCellStatusListHold [counter1*12+8], activeCellStatusListKeepCount++;

                                activeCellStatusListKeep [activeCellStatusListKeepCount] = activeCellStatusListHold [counter1*12+9], activeCellStatusListKeepCount++;

                                activeCellStatusListKeep [activeCellStatusListKeepCount] = activeCellStatusListHold [counter1*12+10], activeCellStatusListKeepCount++;

                                activeCellStatusListKeep [activeCellStatusListKeepCount] = activeCellStatusListHold [counter1*12+11], activeCellStatusListKeepCount++;

                                cellNoFusionCheckHold = activeCellStatusList [counter1*12];

                                activeCellStatusList [counter1*12+1] = -1;

                            }

**else** **if** (timeKeep == growthCycleBase){

                                lineageAddTemp [lineageAddTempCount] = 1, lineageAddTempCount++;

                                lineageAddTemp [lineageAddTempCount] = 1, lineageAddTempCount++;

                                lineageAddTemp [lineageAddTempCount] = timeKeep, lineageAddTempCount++;

                                lineageAddTemp [lineageAddTempCount] = 2, lineageAddTempCount++;

                                lineageAddTemp [lineageAddTempCount] = 0, lineageAddTempCount++;

                                lineageAddTemp [lineageAddTempCount] = activeCellStatusList [counter1*12], lineageAddTempCount++;

                                lineageAddTemp [lineageAddTempCount] = activeCellStatusList [counter1*12+3], lineageAddTempCount++;

                                lineageAddTemp [lineageAddTempCount] = 0, lineageAddTempCount++;

                                lineageAddTemp [lineageAddTempCount] = 0, lineageAddTempCount++;

                                activeCellStatusListKeep [activeCellStatusListKeepCount] = activeCellStatusListHold [counter1*12], activeCellStatusListKeepCount++;

                                activeCellStatusListKeep [activeCellStatusListKeepCount] = activeCellStatusListHold [counter1*12+1], activeCellStatusListKeepCount++;

                                activeCellStatusListKeep [activeCellStatusListKeepCount] = activeCellStatusListHold [counter1*12+2], activeCellStatusListKeepCount++;

                                activeCellStatusListKeep [activeCellStatusListKeepCount] = activeCellStatusListHold [counter1*12+3], activeCellStatusListKeepCount++;

                                activeCellStatusListKeep [activeCellStatusListKeepCount] = activeCellStatusListHold [counter1*12+4], activeCellStatusListKeepCount++;

                                activeCellStatusListKeep [activeCellStatusListKeepCount] = activeCellStatusListHold [counter1*12+5], activeCellStatusListKeepCount++;

                                activeCellStatusListKeep [activeCellStatusListKeepCount] = activeCellStatusListHold [counter1*12+6], activeCellStatusListKeepCount++;

                                activeCellStatusListKeep [activeCellStatusListKeepCount] = activeCellStatusListHold [counter1*12+7], activeCellStatusListKeepCount++;

                                activeCellStatusListKeep [activeCellStatusListKeepCount] = activeCellStatusListHold [counter1*12+8], activeCellStatusListKeepCount++;

                                activeCellStatusListKeep [activeCellStatusListKeepCount] = activeCellStatusListHold [counter1*12+9], activeCellStatusListKeepCount++;

                                activeCellStatusListKeep [activeCellStatusListKeepCount] = activeCellStatusListHold [counter1*12+10], activeCellStatusListKeepCount++;

                                activeCellStatusListKeep [activeCellStatusListKeepCount] = activeCellStatusListHold [counter1*12+11], activeCellStatusListKeepCount++;

                                cellNoFusionCheckHold = activeCellStatusList [counter1*12];

                                activeCellStatusList [counter1*12+1] = -1;

                            }

                        }

**if** (activeCellStatusList [counter1*12+5] == 2){

**if** (timeKeep+2 <= growthCycleBase){

                                lineageAddTemp [lineageAddTempCount] = 1, lineageAddTempCount++;

                                lineageAddTemp [lineageAddTempCount] = 1, lineageAddTempCount++;

                                lineageAddTemp [lineageAddTempCount] = timeKeep, lineageAddTempCount++;

                                lineageAddTemp [lineageAddTempCount] = 6, lineageAddTempCount++;

                                lineageAddTemp [lineageAddTempCount] = 0, lineageAddTempCount++;

                                lineageAddTemp [lineageAddTempCount] = activeCellStatusList [counter1*12], lineageAddTempCount++;

                                lineageAddTemp [lineageAddTempCount] = activeCellStatusList [counter1*12+3], lineageAddTempCount++;

                                lineageAddTemp [lineageAddTempCount] = 0, lineageAddTempCount++;

                                lineageAddTemp [lineageAddTempCount] = 0, lineageAddTempCount++;

                                lineageAddTemp [lineageAddTempCount] = 1, lineageAddTempCount++;

                                lineageAddTemp [lineageAddTempCount] = 1, lineageAddTempCount++;

                                lineageAddTemp [lineageAddTempCount] = timeKeep+1, lineageAddTempCount++;

                                lineageAddTemp [lineageAddTempCount] = 42, lineageAddTempCount++;

                                lineageAddTemp [lineageAddTempCount] = 0, lineageAddTempCount++;

                                lineageAddTemp [lineageAddTempCount] = activeCellStatusList [counter1*12], lineageAddTempCount++;

                                lineageAddTemp [lineageAddTempCount] = activeCellStatusList [counter1*12+3], lineageAddTempCount++;

                                lineageAddTemp [lineageAddTempCount] = 0, lineageAddTempCount++;

                                lineageAddTemp [lineageAddTempCount] = 0, lineageAddTempCount++;

                                createNewCellNo = [[CreateNewCellNo alloc] init];

                                newCellNumber1 = [createNewCellNo cellNumberAddition:counter1];

                                [CreateNewCellNo release];

                                cellNoLingNoList [cellNoLingNoListCount] = activeCellStatusList [counter1*12+3], cellNoLingNoListCount++;

                                cellNoLingNoList [cellNoLingNoListCount] = newCellNumber1, cellNoLingNoListCount++;

                                createNewCellNo = [[CreateNewCellNo alloc] init];

                                newCellNumber2 = [createNewCellNo cellNumberSubtraction:counter1];

                                [CreateNewCellNo release];

                                cellNoLingNoList [cellNoLingNoListCount] = activeCellStatusList [counter1*12+3], cellNoLingNoListCount++;

                                cellNoLingNoList [cellNoLingNoListCount] = newCellNumber2, cellNoLingNoListCount++;

                                createNewCellNo = [[CreateNewCellNo alloc] init];

                                newCellNumber3 = [createNewCellNo cellNumberAdditionSecond:counter1];

                                [CreateNewCellNo release];

                                cellNoLingNoList [cellNoLingNoListCount] = activeCellStatusList [counter1*12+3], cellNoLingNoListCount++;

                                cellNoLingNoList [cellNoLingNoListCount] = newCellNumber3, cellNoLingNoListCount++;

                                lineageAddTemp [lineageAddTempCount] = 1, lineageAddTempCount++;

                                lineageAddTemp [lineageAddTempCount] = 1, lineageAddTempCount++;

                                lineageAddTemp [lineageAddTempCount] = timeKeep+2, lineageAddTempCount++;

                                lineageAddTemp [lineageAddTempCount] = 41, lineageAddTempCount++;

                                lineageAddTemp [lineageAddTempCount] = activeCellStatusList [counter1*12], lineageAddTempCount++;

                                lineageAddTemp [lineageAddTempCount] = newCellNumber1, lineageAddTempCount++;

                                lineageAddTemp [lineageAddTempCount] = activeCellStatusList [counter1*12+3], lineageAddTempCount++;

                                lineageAddTemp [lineageAddTempCount] = 0, lineageAddTempCount++;

                                lineageAddTemp [lineageAddTempCount] = 0, lineageAddTempCount++;

                                lineageAddTemp [lineageAddTempCount] = 1, lineageAddTempCount++;

                                lineageAddTemp [lineageAddTempCount] = 1, lineageAddTempCount++;

                                lineageAddTemp [lineageAddTempCount] = timeKeep+2, lineageAddTempCount++;

                                lineageAddTemp [lineageAddTempCount] = 41, lineageAddTempCount++;

                                lineageAddTemp [lineageAddTempCount] = activeCellStatusList [counter1*12], lineageAddTempCount++;

                                lineageAddTemp [lineageAddTempCount] = newCellNumber2, lineageAddTempCount++;

                                lineageAddTemp [lineageAddTempCount] = activeCellStatusList [counter1*12+3], lineageAddTempCount++;

                                lineageAddTemp [lineageAddTempCount] = 0, lineageAddTempCount++;

                                lineageAddTemp [lineageAddTempCount] = 0, lineageAddTempCount++;

                                lineageAddTemp [lineageAddTempCount] = 1, lineageAddTempCount++;

                                lineageAddTemp [lineageAddTempCount] = 1, lineageAddTempCount++;

                                lineageAddTemp [lineageAddTempCount] = timeKeep+2, lineageAddTempCount++;

                                lineageAddTemp [lineageAddTempCount] = 41, lineageAddTempCount++;

                                lineageAddTemp [lineageAddTempCount] = activeCellStatusList [counter1*12], lineageAddTempCount++;

                                lineageAddTemp [lineageAddTempCount] = newCellNumber3, lineageAddTempCount++;

                                lineageAddTemp [lineageAddTempCount] = activeCellStatusList [counter1*12+3], lineageAddTempCount++;

                                lineageAddTemp [lineageAddTempCount] = 0, lineageAddTempCount++;

                                lineageAddTemp [lineageAddTempCount] = 0, lineageAddTempCount++;

**if** (timeKeep+2 < growthCycleBase){

                                    tempListOfCells [tempListOfCellsCount] = activeCellStatusList [counter1*12+3], tempListOfCellsCount++;

                                    tempListOfCells [tempListOfCellsCount] = activeCellStatusList [counter1*12], tempListOfCellsCount++;

                                    tempListOfCells [tempListOfCellsCount] = newCellNumber1, tempListOfCellsCount++;

                                    tempListOfCells [tempListOfCellsCount] = newCellNumber2, tempListOfCellsCount++;

                                    tempListOfCells [tempListOfCellsCount] = newCellNumber3, tempListOfCellsCount++;

                                    tempListOfCells [tempListOfCellsCount] = activeCellStatusList [counter1*12+5], tempListOfCellsCount++;

                                    tempListOfCells [tempListOfCellsCount] = activeCellStatusList [counter1*12+4], tempListOfCellsCount++;

                                }

**else**{

                                    activeCellStatusListKeep [activeCellStatusListKeepCount] = newCellNumber1, activeCellStatusListKeepCount++;

                                    activeCellStatusListKeep [activeCellStatusListKeepCount] = newCellNumber2, activeCellStatusListKeepCount++;

                                    activeCellStatusListKeep [activeCellStatusListKeepCount] = newCellNumber3, activeCellStatusListKeepCount++;

                                    activeCellStatusListKeep [activeCellStatusListKeepCount] = activeCellStatusList [counter1*12+3], activeCellStatusListKeepCount++;

                                    activeCellStatusListKeep [activeCellStatusListKeepCount] = 0, activeCellStatusListKeepCount++;

                                    activeCellStatusListKeep [activeCellStatusListKeepCount] = 1, activeCellStatusListKeepCount++;

                                    activeCellStatusListKeep [activeCellStatusListKeepCount] = 0, activeCellStatusListKeepCount++;

                                    activeCellStatusListKeep [activeCellStatusListKeepCount] = activeCellStatusList [counter1*12+7], activeCellStatusListKeepCount++;

                                    activeCellStatusListKeep [activeCellStatusListKeepCount] = activeCellStatusList [counter1*12+8], activeCellStatusListKeepCount++;

                                    activeCellStatusListKeep [activeCellStatusListKeepCount] = activeCellStatusList [counter1*12+9], activeCellStatusListKeepCount++;

                                    activeCellStatusListKeep [activeCellStatusListKeepCount] = activeCellStatusList [counter1*12], activeCellStatusListKeepCount++;

                                    activeCellStatusListKeep [activeCellStatusListKeepCount] = activeCellStatusList [counter1*12+4], activeCellStatusListKeepCount++;

                                    activeCellStatusListKeep [activeCellStatusListKeepCount] = newCellNumber2, activeCellStatusListKeepCount++;

                                    activeCellStatusListKeep [activeCellStatusListKeepCount] = newCellNumber3, activeCellStatusListKeepCount++;

                                    activeCellStatusListKeep [activeCellStatusListKeepCount] = newCellNumber1, activeCellStatusListKeepCount++;

                                    activeCellStatusListKeep [activeCellStatusListKeepCount] = activeCellStatusList [counter1*12+3], activeCellStatusListKeepCount++;

                                    activeCellStatusListKeep [activeCellStatusListKeepCount] = 0, activeCellStatusListKeepCount++;

                                    activeCellStatusListKeep [activeCellStatusListKeepCount] = 1, activeCellStatusListKeepCount++;

                                    activeCellStatusListKeep [activeCellStatusListKeepCount] = 0, activeCellStatusListKeepCount++;

                                    activeCellStatusListKeep [activeCellStatusListKeepCount] = activeCellStatusList [counter1*12+7], activeCellStatusListKeepCount++;

                                    activeCellStatusListKeep [activeCellStatusListKeepCount] = activeCellStatusList [counter1*12+8], activeCellStatusListKeepCount++;

                                    activeCellStatusListKeep [activeCellStatusListKeepCount] = activeCellStatusList [counter1*12+9], activeCellStatusListKeepCount++;

                                    activeCellStatusListKeep [activeCellStatusListKeepCount] = activeCellStatusList [counter1*12], activeCellStatusListKeepCount++;

                                    activeCellStatusListKeep [activeCellStatusListKeepCount] = activeCellStatusList [counter1*12+4], activeCellStatusListKeepCount++;

                                    activeCellStatusListKeep [activeCellStatusListKeepCount] = newCellNumber3, activeCellStatusListKeepCount++;

                                    activeCellStatusListKeep [activeCellStatusListKeepCount] = newCellNumber1, activeCellStatusListKeepCount++;

                                    activeCellStatusListKeep [activeCellStatusListKeepCount] = newCellNumber2, activeCellStatusListKeepCount++;

                                    activeCellStatusListKeep [activeCellStatusListKeepCount] = activeCellStatusList [counter1*12+3], activeCellStatusListKeepCount++;

                                    activeCellStatusListKeep [activeCellStatusListKeepCount] = 0, activeCellStatusListKeepCount++;

                                    activeCellStatusListKeep [activeCellStatusListKeepCount] = 1, activeCellStatusListKeepCount++;

                                    activeCellStatusListKeep [activeCellStatusListKeepCount] = 0, activeCellStatusListKeepCount++;

                                    activeCellStatusListKeep [activeCellStatusListKeepCount] = activeCellStatusList [counter1*12+7], activeCellStatusListKeepCount++;

                                    activeCellStatusListKeep [activeCellStatusListKeepCount] = activeCellStatusList [counter1*12+8], activeCellStatusListKeepCount++;

                                    activeCellStatusListKeep [activeCellStatusListKeepCount] = activeCellStatusList [counter1*12+9], activeCellStatusListKeepCount++;

                                    activeCellStatusListKeep [activeCellStatusListKeepCount] = activeCellStatusList [counter1*12], activeCellStatusListKeepCount++;

                                    activeCellStatusListKeep [activeCellStatusListKeepCount] = activeCellStatusList [counter1*12+4], activeCellStatusListKeepCount++;

                                }

                                cellNoFusionCheckHold = activeCellStatusList [counter1*12];

                                activeCellStatusList [counter1*12+1] = -1;

                            }

**else** **if** (timeKeep+1 == growthCycleBase){

                                lineageAddTemp [lineageAddTempCount] = 1, lineageAddTempCount++;

                                lineageAddTemp [lineageAddTempCount] = 1, lineageAddTempCount++;

                                lineageAddTemp [lineageAddTempCount] = timeKeep, lineageAddTempCount++;

                                lineageAddTemp [lineageAddTempCount] = 2, lineageAddTempCount++;

                                lineageAddTemp [lineageAddTempCount] = 0, lineageAddTempCount++;

                                lineageAddTemp [lineageAddTempCount] = activeCellStatusList [counter1*12], lineageAddTempCount++;

                                lineageAddTemp [lineageAddTempCount] = activeCellStatusList [counter1*12+3], lineageAddTempCount++;

                                lineageAddTemp [lineageAddTempCount] = 0, lineageAddTempCount++;

                                lineageAddTemp [lineageAddTempCount] = 0, lineageAddTempCount++;

                                lineageAddTemp [lineageAddTempCount] = 1, lineageAddTempCount++;

                                lineageAddTemp [lineageAddTempCount] = 1, lineageAddTempCount++;

                                lineageAddTemp [lineageAddTempCount] = timeKeep+1, lineageAddTempCount++;

                                lineageAddTemp [lineageAddTempCount] = 2, lineageAddTempCount++;

                                lineageAddTemp [lineageAddTempCount] = 0, lineageAddTempCount++;

                                lineageAddTemp [lineageAddTempCount] = activeCellStatusList [counter1*12], lineageAddTempCount++;

                                lineageAddTemp [lineageAddTempCount] = activeCellStatusList [counter1*12+3], lineageAddTempCount++;

                                lineageAddTemp [lineageAddTempCount] = 0, lineageAddTempCount++;

                                lineageAddTemp [lineageAddTempCount] = 0, lineageAddTempCount++;

                                activeCellStatusListKeep [activeCellStatusListKeepCount] = activeCellStatusListHold [counter1*12], activeCellStatusListKeepCount++;

                                activeCellStatusListKeep [activeCellStatusListKeepCount] = activeCellStatusListHold [counter1*12+1], activeCellStatusListKeepCount++;

                                activeCellStatusListKeep [activeCellStatusListKeepCount] = activeCellStatusListHold [counter1*12+2], activeCellStatusListKeepCount++;

                                activeCellStatusListKeep [activeCellStatusListKeepCount] = activeCellStatusListHold [counter1*12+3], activeCellStatusListKeepCount++;

                                activeCellStatusListKeep [activeCellStatusListKeepCount] = activeCellStatusListHold [counter1*12+4], activeCellStatusListKeepCount++;

                                activeCellStatusListKeep [activeCellStatusListKeepCount] = activeCellStatusListHold [counter1*12+5], activeCellStatusListKeepCount++;

                                activeCellStatusListKeep [activeCellStatusListKeepCount] = activeCellStatusListHold [counter1*12+6], activeCellStatusListKeepCount++;

                                activeCellStatusListKeep [activeCellStatusListKeepCount] = activeCellStatusListHold [counter1*12+7], activeCellStatusListKeepCount++;

                                activeCellStatusListKeep [activeCellStatusListKeepCount] = activeCellStatusListHold [counter1*12+8], activeCellStatusListKeepCount++;

                                activeCellStatusListKeep [activeCellStatusListKeepCount] = activeCellStatusListHold [counter1*12+9], activeCellStatusListKeepCount++;

                                activeCellStatusListKeep [activeCellStatusListKeepCount] = activeCellStatusListHold [counter1*12+10], activeCellStatusListKeepCount++;

                                activeCellStatusListKeep [activeCellStatusListKeepCount] = activeCellStatusListHold [counter1*12+11], activeCellStatusListKeepCount++;

                                cellNoFusionCheckHold = activeCellStatusList [counter1*12];

                                activeCellStatusList [counter1*12+1] = -1;

                            }

**else** **if** (timeKeep == growthCycleBase){

                                lineageAddTemp [lineageAddTempCount] = 1, lineageAddTempCount++;

                                lineageAddTemp [lineageAddTempCount] = 1, lineageAddTempCount++;

                                lineageAddTemp [lineageAddTempCount] = timeKeep, lineageAddTempCount++;

                                lineageAddTemp [lineageAddTempCount] = 2, lineageAddTempCount++;

                                lineageAddTemp [lineageAddTempCount] = 0, lineageAddTempCount++;

                                lineageAddTemp [lineageAddTempCount] = activeCellStatusList [counter1*12], lineageAddTempCount++;

                                lineageAddTemp [lineageAddTempCount] = activeCellStatusList [counter1*12+3], lineageAddTempCount++;

                                lineageAddTemp [lineageAddTempCount] = 0, lineageAddTempCount++;

                                lineageAddTemp [lineageAddTempCount] = 0, lineageAddTempCount++;

                                activeCellStatusListKeep [activeCellStatusListKeepCount] = activeCellStatusListHold [counter1*12], activeCellStatusListKeepCount++;

                                activeCellStatusListKeep [activeCellStatusListKeepCount] = activeCellStatusListHold [counter1*12+1], activeCellStatusListKeepCount++;

                                activeCellStatusListKeep [activeCellStatusListKeepCount] = activeCellStatusListHold [counter1*12+2], activeCellStatusListKeepCount++;

                                activeCellStatusListKeep [activeCellStatusListKeepCount] = activeCellStatusListHold [counter1*12+3], activeCellStatusListKeepCount++;

                                activeCellStatusListKeep [activeCellStatusListKeepCount] = activeCellStatusListHold [counter1*12+4], activeCellStatusListKeepCount++;

                                activeCellStatusListKeep [activeCellStatusListKeepCount] = activeCellStatusListHold [counter1*12+5], activeCellStatusListKeepCount++;

                                activeCellStatusListKeep [activeCellStatusListKeepCount] = activeCellStatusListHold [counter1*12+6], activeCellStatusListKeepCount++;

                                activeCellStatusListKeep [activeCellStatusListKeepCount] = activeCellStatusListHold [counter1*12+7], activeCellStatusListKeepCount++;

                                activeCellStatusListKeep [activeCellStatusListKeepCount] = activeCellStatusListHold [counter1*12+8], activeCellStatusListKeepCount++;

                                activeCellStatusListKeep [activeCellStatusListKeepCount] = activeCellStatusListHold [counter1*12+9], activeCellStatusListKeepCount++;

                                activeCellStatusListKeep [activeCellStatusListKeepCount] = activeCellStatusListHold [counter1*12+10], activeCellStatusListKeepCount++;

                                activeCellStatusListKeep [activeCellStatusListKeepCount] = activeCellStatusListHold [counter1*12+11], activeCellStatusListKeepCount++;

                                cellNoFusionCheckHold = activeCellStatusList [counter1*12];

                                activeCellStatusList [counter1*12+1] = -1;

                            }

                        }

**if** (activeCellStatusList [counter1*12+5] == 3){

**if** (timeKeep <= growthCycleBase){

                                lineageAddTemp [lineageAddTempCount] = 1, lineageAddTempCount++;

                                lineageAddTemp [lineageAddTempCount] = 1, lineageAddTempCount++;

                                lineageAddTemp [lineageAddTempCount] = timeKeep, lineageAddTempCount++;

                                lineageAddTemp [lineageAddTempCount] = 7, lineageAddTempCount++;

                                lineageAddTemp [lineageAddTempCount] = 0, lineageAddTempCount++;

                                lineageAddTemp [lineageAddTempCount] = activeCellStatusList [counter1*12], lineageAddTempCount++;

                                lineageAddTemp [lineageAddTempCount] = activeCellStatusList [counter1*12+3], lineageAddTempCount++;

                                lineageAddTemp [lineageAddTempCount] = 0, lineageAddTempCount++;

                                lineageAddTemp [lineageAddTempCount] = 0, lineageAddTempCount++;

                                cellNoFusionCheckHold = activeCellStatusList [counter1*12];

                                activeCellStatusList [counter1*12+1] = -1;

                            }

                        }

**if** (activeCellStatusList [counter1*12+5] == 6){

**if** (timeKeep+1 <= growthCycleBase){

**if** (activeCellStatusList [counter1*12+6] != 0){

                                    fusionPartnerList [fusionPartnerListCount] = (**long**)activeCellStatusList [counter1*12+6], fusionPartnerListCount++;

                                    fusionPartnerList [fusionPartnerListCount] = (**long**)activeCellStatusList [counter1*12+3], fusionPartnerListCount++;

                                    fusionPartnerList [fusionPartnerListCount] = (**long**)timeKeep, fusionPartnerListCount++;

                                    fusionPartnerList [fusionPartnerListCount] = (**long**)activeCellStatusList [counter1*12], fusionPartnerListCount++;

                                    fusionPartnerList [fusionPartnerListCount] = 0, fusionPartnerListCount++;

                                }

                                lineageAddTemp [lineageAddTempCount] = 1, lineageAddTempCount++;

                                lineageAddTemp [lineageAddTempCount] = 1, lineageAddTempCount++;

                                lineageAddTemp [lineageAddTempCount] = timeKeep, lineageAddTempCount++;

                                lineageAddTemp [lineageAddTempCount] = 91, lineageAddTempCount++;

                                lineageAddTemp [lineageAddTempCount] = activeCellStatusList [counter1*12+6], lineageAddTempCount++;

                                lineageAddTemp [lineageAddTempCount] = activeCellStatusList [counter1*12], lineageAddTempCount++;

                                lineageAddTemp [lineageAddTempCount] = activeCellStatusList [counter1*12+3], lineageAddTempCount++;

                                lineageAddTemp [lineageAddTempCount] = activeCellStatusList [counter1*12+3], lineageAddTempCount++;

                                lineageAddTemp [lineageAddTempCount] = 0, lineageAddTempCount++;

                                cellNoFusionCheckHold = activeCellStatusList [counter1*12];

                                activeCellStatusList [counter1*12+1] = -1;

                            }

**else** **if** (timeKeep == growthCycleBase){

                                lineageAddTemp [lineageAddTempCount] = 1, lineageAddTempCount++;

                                lineageAddTemp [lineageAddTempCount] = 1, lineageAddTempCount++;

                                lineageAddTemp [lineageAddTempCount] = timeKeep, lineageAddTempCount++;

                                lineageAddTemp [lineageAddTempCount] = 7, lineageAddTempCount++;

                                lineageAddTemp [lineageAddTempCount] = activeCellStatusList [counter1*12+6], lineageAddTempCount++;

                                lineageAddTemp [lineageAddTempCount] = activeCellStatusList [counter1*12], lineageAddTempCount++;

                                lineageAddTemp [lineageAddTempCount] = activeCellStatusList [counter1*12+3], lineageAddTempCount++;

                                lineageAddTemp [lineageAddTempCount] = activeCellStatusList [counter1*12+3], lineageAddTempCount++;

                                lineageAddTemp [lineageAddTempCount] = 0, lineageAddTempCount++;

                                cellNoFusionCheckHold = activeCellStatusList [counter1*12];

                                activeCellStatusList [counter1*12+1] = -1;

                            }

                        }

                        newLimit = 0;

                        startPositionList [startPositionListCount] = (**long**)cellNoFusionCheckHold, startPositionListCount++;

                        startPositionList [startPositionListCount] = (**long**)activeCellStatusList [counter1*12+3], startPositionListCount++;

                        startPositionList [startPositionListCount] = (**long**)cellLineageTempArrayCount, startPositionListCount++;

**if** (activeCellStatusList [counter1*12+5] == 6){

**if** (timeKeep+1 <= growthCycleBase){

**if** (activeCellStatusList [counter1*12+6] != 0){

                                    fusionPartnerList [fusionPartnerListCount-1] = (**long**)cellLineageTempArrayCount;

                                }

                            }

                        }

**for** (**int** counter2 = 0; counter2 < lineageAddTempCount/9; counter2++){

**if** (cellLineageTempArrayLimit+(**unsigned** **long**)activeCellStatusListCount*(**unsigned** **long**)simProcessDataMiddleHold [25]*10+10 > 550000000000000000){

**if** (cellLineageTempArrayLimit < 550000000000000000) newLimit = 1;

**else**{

                                    arrayOverflow = 1;

**break**;

                                }

                            }

**if** (cellLineageTempArrayCount+9 > cellLineageTempArrayLimit){

**int** *arrayUpDate = **new** **int** [cellLineageTempArrayCount+10];

**for** (**int** counter3 = 0; counter3 < cellLineageTempArrayCount; counter3++) arrayUpDate [counter3] = cellLineageTempArray [counter3];

**delete** [] cellLineageTempArray;

**if** (newLimit == 0){

                                    cellLineageTempArray = **new** **int** [cellLineageTempArrayLimit+(**unsigned** **long**)activeCellStatusListCount*(**unsigned** **long**)simProcessDataMiddleHold [25]*10+10];

                                    cellLineageTempArrayLimit = cellLineageTempArrayLimit+(**unsigned** **long**)activeCellStatusListCount*(**unsigned** **long**)simProcessDataMiddleHold [25]*10+10;

                                }

**else**{

                                    cellLineageTempArray = **new** **int** [550000000000000000];

                                    cellLineageTempArrayLimit = 550000000000000000;

                                }

**for** (**int** counter3 = 0; counter3 < cellLineageTempArrayCount; counter3++) cellLineageTempArray [counter3] = arrayUpDate [counter3];

**delete** [] arrayUpDate;

                            }

                            cellLineageTempArray [cellLineageTempArrayCount] = lineageAddTemp [counter2*9], cellLineageTempArrayCount++;

                            cellLineageTempArray [cellLineageTempArrayCount] = lineageAddTemp [counter2*9+1], cellLineageTempArrayCount++;

                            cellLineageTempArray [cellLineageTempArrayCount] = lineageAddTemp [counter2*9+2], cellLineageTempArrayCount++;

                            cellLineageTempArray [cellLineageTempArrayCount] = lineageAddTemp [counter2*9+3], cellLineageTempArrayCount++;

                            cellLineageTempArray [cellLineageTempArrayCount] = lineageAddTemp [counter2*9+4], cellLineageTempArrayCount++;

                            cellLineageTempArray [cellLineageTempArrayCount] = lineageAddTemp [counter2*9+5], cellLineageTempArrayCount++;

                            cellLineageTempArray [cellLineageTempArrayCount] = lineageAddTemp [counter2*9+6], cellLineageTempArrayCount++;

                            cellLineageTempArray [cellLineageTempArrayCount] = lineageAddTemp [counter2*9+7], cellLineageTempArrayCount++;

                            cellLineageTempArray [cellLineageTempArrayCount] = lineageAddTemp [counter2*9+8], cellLineageTempArrayCount++;

                        }

                    }

**if** (arrayOverflow == 1){

**break**;

                    }

                }

**delete** [] lineageAddTemp;

**if** (reachMaxDivision != 1000000000 && reachMaxDivision < growthCycleBase){

                    growthCycleBase = reachMaxDivision;

                }

**if** (terminate2 == 0){

**for** (**int** counter1 = 0; counter1 < fusionPartnerListCount/5; counter1++){

                        missingPartnerCheck = 0;

**for** (**int** counter2 = 0; counter2 < startPositionListCount/3; counter2++){

**if** (fusionPartnerList [counter1*5] == startPositionList [counter2*3] && fusionPartnerList [counter1*5+1] == startPositionList [counter2*3+1]){

                                missingPartnerCheck = 1;

**for** (**unsigned** **long** counter3 = (**unsigned** **long**)startPositionList [counter2*3+2]/9; counter3 < cellLineageTempArrayCount/9; counter3++){

**if** (cellLineageTempArray [counter3*9+5] == startPositionList [counter2*3] && cellLineageTempArray [counter3*9+6] == startPositionList [counter2*3+1] && cellLineageTempArray [counter3*9+2] == fusionPartnerList [counter1*5+2]){

                                        cellLineageTempArray [counter3*9+4] = (**int**)fusionPartnerList [counter1*5+3];

                                        cellLineageTempArray [counter3*9+7] = (**int**)fusionPartnerList [counter1*5+1];

                                        cellLineageTempArray [counter3*9+3] = 92;

**break**;

                                    }

**else** **if** (cellLineageTempArray [counter3*9+5] != startPositionList [counter2*3] || cellLineageTempArray [counter3*9+6] != startPositionList [counter2*3+1]){

**break**;

                                    }

                                }

**break**;

                            }

                        }

**if** (missingPartnerCheck == 0){

**for** (**unsigned** **long** counter3 = (**unsigned** **long**)(fusionPartnerList [counter1*5+4]/9); counter3 < cellLineageTempArrayCount/9; counter3++){

**if** (cellLineageTempArray [counter3*9+5] == fusionPartnerList [counter1*5+1] && cellLineageTempArray [counter3*9+6] == fusionPartnerList [counter1*5+3] && cellLineageTempArray [counter3*9+3] == 91){

                                    cellLineageTempArray [counter3*9+4] = 0;

                                    cellLineageTempArray [counter3*9+7] = 0;

                                    cellLineageTempArray [counter3*9+3] = 7;

**break**;

                                }

                            }

                        }

                    }

                }

**delete** [] fusionPartnerList;

**delete** [] startPositionList;

**if** (terminate2 == 0){

                    entryCount2 = 0;

                    cellNoforSummary = 0;

                    clingNoforSummary = 0;

                    fusionForSummary = 0;

                    cellLineageSummaryArrayCount = 0;

**for** (**unsigned** **long** counter2 = 0; counter2 < cellLineageTempArrayCount/9; counter2++){

**if** (cellLineageTempArray [counter2*9+8] != -1){

                            cellLineageTempArray [entryCount2] = cellLineageTempArray [counter2*9], entryCount2++;

                            cellLineageTempArray [entryCount2] = cellLineageTempArray [counter2*9+1], entryCount2++;

                            cellLineageTempArray [entryCount2] = cellLineageTempArray [counter2*9+2], entryCount2++;

                            cellLineageTempArray [entryCount2] = cellLineageTempArray [counter2*9+3], entryCount2++;

                            cellLineageTempArray [entryCount2] = cellLineageTempArray [counter2*9+4], entryCount2++;

                            cellLineageTempArray [entryCount2] = cellLineageTempArray [counter2*9+5], entryCount2++;

                            cellLineageTempArray [entryCount2] = cellLineageTempArray [counter2*9+6], entryCount2++;

                            cellLineageTempArray [entryCount2] = cellLineageTempArray [counter2*9+7], entryCount2++;

                            cellLineageTempArray [entryCount2] = cellLineageTempArray [counter2*9+8], entryCount2++;

**if** (cellLineageTempArray [entryCount2-6] == 92) fusionForSummary = 1;

**if** (cellLineageTempArray [entryCount2-4] != cellNoforSummary || cellLineageTempArray [entryCount2-3] != clingNoforSummary || counter2 == cellLineageTempArrayCount/9-1){

**if** (cellLineageSummaryArrayCount+50 > cellLineageSummaryArrayLimit){

**long** *arrayUpDate = **new** **long** [cellLineageSummaryArrayCount+10];

**for** (**unsigned** **long** counter3 = 0; counter3 < cellLineageSummaryArrayCount; counter3++) arrayUpDate [counter3] = cellLineageSummaryArray [counter3];

**delete** [] cellLineageSummaryArray;

                                    cellLineageSummaryArray = **new** **long** [cellLineageSummaryArrayLimit+100000000];

                                    cellLineageSummaryArrayLimit = cellLineageSummaryArrayLimit+100000000;

**for** (**unsigned** **long** counter3 = 0; counter3 < cellLineageSummaryArrayCount; counter3++) cellLineageSummaryArray [counter3] = arrayUpDate [counter3];

**delete** [] arrayUpDate;

                                }

**if** (cellLineageSummaryArrayCount == 0){

                                    cellNoforSummary = cellLineageTempArray [entryCount2-4];

                                    clingNoforSummary = cellLineageTempArray [entryCount2-3];

                                    fusionForSummary = 0;

                                    cellLineageSummaryArray [cellLineageSummaryArrayCount] = 0, cellLineageSummaryArrayCount++;

                                    cellLineageSummaryArray [cellLineageSummaryArrayCount] = 0, cellLineageSummaryArrayCount++;

                                    cellLineageSummaryArray [cellLineageSummaryArrayCount] = (**long**)clingNoforSummary, cellLineageSummaryArrayCount++;

                                    cellLineageSummaryArray [cellLineageSummaryArrayCount] = (**long**)cellNoforSummary, cellLineageSummaryArrayCount++;

                                    cellLineageSummaryArray [cellLineageSummaryArrayCount] = (**long**)cellLineageTempArray [entryCount2-5], cellLineageSummaryArrayCount++;

                                    cellLineageSummaryArray [cellLineageSummaryArrayCount] = (**long**)clingNoforSummary, cellLineageSummaryArrayCount++;

                                    cellLineageSummaryArray [cellLineageSummaryArrayCount] = (**long**)cellLineageTempArray [entryCount2-6], cellLineageSummaryArrayCount++;

                                    cellLineageSummaryArray [cellLineageSummaryArrayCount] = 0, cellLineageSummaryArrayCount++;

                                    cellLineageSummaryArray [cellLineageSummaryArrayCount] = 0, cellLineageSummaryArrayCount++;

                                }

**else** **if** (cellLineageSummaryArrayCount != 0 && counter2 != cellLineageTempArrayCount/9-1){

                                    cellLineageSummaryArray [cellLineageSummaryArrayCount-8] = (**long**)entryCount2-18;

**if** ((**long**)cellLineageTempArray [entryCount2-15] == 31 || (**long**)cellLineageTempArray [entryCount2-15] == 41){

                                        cellLineageSummaryArray [cellLineageSummaryArrayCount-2] = 2;

                                    }

**else** cellLineageSummaryArray [cellLineageSummaryArrayCount-2] = (**long**)cellLineageTempArray [entryCount2-15];

**if** (fusionForSummary == 1) cellLineageSummaryArray [cellLineageSummaryArrayCount-1] = 1;

                                    cellNoforSummary = cellLineageTempArray [entryCount2-4];

                                    clingNoforSummary = cellLineageTempArray [entryCount2-3];

                                    fusionForSummary = 0;

                                    cellLineageSummaryArray [cellLineageSummaryArrayCount] = (**long**)entryCount2-9, cellLineageSummaryArrayCount++;

                                    cellLineageSummaryArray [cellLineageSummaryArrayCount] = 0, cellLineageSummaryArrayCount++;

                                    cellLineageSummaryArray [cellLineageSummaryArrayCount] = (**long**)clingNoforSummary, cellLineageSummaryArrayCount++;

                                    cellLineageSummaryArray [cellLineageSummaryArrayCount] = (**long**)cellNoforSummary, cellLineageSummaryArrayCount++;

                                    cellLineageSummaryArray [cellLineageSummaryArrayCount] = (**long**)cellLineageTempArray [entryCount2-5], cellLineageSummaryArrayCount++;

                                    cellLineageSummaryArray [cellLineageSummaryArrayCount] = (**long**)clingNoforSummary, cellLineageSummaryArrayCount++;

                                    cellLineageSummaryArray [cellLineageSummaryArrayCount] = (**long**)cellLineageTempArray [entryCount2-6], cellLineageSummaryArrayCount++;

                                    cellLineageSummaryArray [cellLineageSummaryArrayCount] = 0, cellLineageSummaryArrayCount++;

                                    cellLineageSummaryArray [cellLineageSummaryArrayCount] = 0, cellLineageSummaryArrayCount++;

                                }

**else** **if** (cellLineageSummaryArrayCount != 0 && counter2 == cellLineageTempArrayCount/9-1){

**if** (cellLineageTempArray [entryCount2-13] != cellLineageTempArray [entryCount2-4] || cellLineageTempArray [entryCount2-12] != cellLineageTempArray [entryCount2-3]){

                                        cellLineageSummaryArray [cellLineageSummaryArrayCount-8] = (**long**)entryCount2-18;

                                        cellLineageSummaryArray [cellLineageSummaryArrayCount-2] = 2;

**if** (fusionForSummary == 1) cellLineageSummaryArray [cellLineageSummaryArrayCount-1] = 1;

                                        cellLineageSummaryArray [cellLineageSummaryArrayCount] = (**long**)entryCount2-9, cellLineageSummaryArrayCount++;

                                        cellLineageSummaryArray [cellLineageSummaryArrayCount] = (**long**)entryCount2-9, cellLineageSummaryArrayCount++;

                                        cellLineageSummaryArray [cellLineageSummaryArrayCount] = (**long**)cellLineageTempArray [entryCount2-3], cellLineageSummaryArrayCount++;

                                        cellLineageSummaryArray [cellLineageSummaryArrayCount] = (**long**)cellLineageTempArray [entryCount2-4], cellLineageSummaryArrayCount++;

                                        cellLineageSummaryArray [cellLineageSummaryArrayCount] = (**long**)cellLineageTempArray [entryCount2-5], cellLineageSummaryArrayCount++;

                                        cellLineageSummaryArray [cellLineageSummaryArrayCount] = (**long**)cellLineageTempArray [entryCount2-3], cellLineageSummaryArrayCount++;

                                        cellLineageSummaryArray [cellLineageSummaryArrayCount] = (**long**)cellLineageTempArray [entryCount2-6], cellLineageSummaryArrayCount++;

                                        cellLineageSummaryArray [cellLineageSummaryArrayCount] = 2, cellLineageSummaryArrayCount++;

                                        cellLineageSummaryArray [cellLineageSummaryArrayCount] = 0, cellLineageSummaryArrayCount++;

                                    }

**else**{

                                        cellLineageSummaryArray [cellLineageSummaryArrayCount-8] = (**long**)entryCount2-9;

                                        cellLineageSummaryArray [cellLineageSummaryArrayCount-2] = 2;

**if** (fusionForSummary == 1) cellLineageSummaryArray [cellLineageSummaryArrayCount-1] = 1;

                                    }

                                }

                            }

                        }

                    }

                    cellLineageTempArrayCount = entryCount2;

**for** (**int** counter1 = 0; counter1 < tempListOfCellsCount/7; counter1++){

                        parentTDCount = 0;

                        parentTDTD = 0;

                        parentTDBD = 0;

**for** (**int** counter2 = 0; counter2 < activeCellStatusListCount/12; counter2++){

**if** (activeCellStatusList [counter2*12] == tempListOfCells [counter1*7+1] && activeCellStatusList [counter2*12+3] == tempListOfCells [counter1*7]){

                                parentTDCount = activeCellStatusList [counter2*12+7];

                                parentTDTD = activeCellStatusList [counter2*12+8];

                                parentTDBD = activeCellStatusList [counter2*12+9];

**break**;

                            }

                        }

**if** (activeCellStatusListCount+50 > activeCellStatusListLimitHold){

**int** *arrayUpDate = **new** **int** [activeCellStatusListCount+10];

**for** (**int** counter2 = 0; counter2 < activeCellStatusListCount; counter2++) arrayUpDate [counter2] = activeCellStatusList [counter2];

**delete** [] activeCellStatusList;

                            activeCellStatusList = **new** **int** [activeCellStatusListLimitHold+10000];

                            activeCellStatusListLimitHold = activeCellStatusListLimitHold+10000;

**for** (**int** counter2 = 0; counter2 < activeCellStatusListCount; counter2++) activeCellStatusList [counter2] = arrayUpDate [counter2];

**delete** [] arrayUpDate;

                        }

**if** (tempListOfCells [counter1*7+5] == 1 || tempListOfCells [counter1*7+5] == 7){

                            activeCellStatusList [activeCellStatusListCount] = tempListOfCells [counter1*7+2], activeCellStatusListCount++;

                            activeCellStatusList [activeCellStatusListCount] = tempListOfCells [counter1*7+3], activeCellStatusListCount++;

                            activeCellStatusList [activeCellStatusListCount] = 0, activeCellStatusListCount++;

                            activeCellStatusList [activeCellStatusListCount] = tempListOfCells [counter1*7], activeCellStatusListCount++;

                            activeCellStatusList [activeCellStatusListCount] = 0, activeCellStatusListCount++;

                            activeCellStatusList [activeCellStatusListCount] = tempListOfCells [counter1*7+5], activeCellStatusListCount++;

                            activeCellStatusList [activeCellStatusListCount] = 0, activeCellStatusListCount++;

                            activeCellStatusList [activeCellStatusListCount] = parentTDCount, activeCellStatusListCount++;

                            activeCellStatusList [activeCellStatusListCount] = parentTDTD, activeCellStatusListCount++;

                            activeCellStatusList [activeCellStatusListCount] = parentTDBD, activeCellStatusListCount++;

                            activeCellStatusList [activeCellStatusListCount] = tempListOfCells [counter1*7+1], activeCellStatusListCount++;

                            activeCellStatusList [activeCellStatusListCount] = tempListOfCells [counter1*7+6], activeCellStatusListCount++;

                            activeCellStatusList [activeCellStatusListCount] = tempListOfCells [counter1*7+3], activeCellStatusListCount++;

                            activeCellStatusList [activeCellStatusListCount] = tempListOfCells [counter1*7+2], activeCellStatusListCount++;

                            activeCellStatusList [activeCellStatusListCount] = 0, activeCellStatusListCount++;

                            activeCellStatusList [activeCellStatusListCount] = tempListOfCells [counter1*7], activeCellStatusListCount++;

                            activeCellStatusList [activeCellStatusListCount] = 0, activeCellStatusListCount++;

                            activeCellStatusList [activeCellStatusListCount] = tempListOfCells [counter1*7+5], activeCellStatusListCount++;

                            activeCellStatusList [activeCellStatusListCount] = 0, activeCellStatusListCount++;

                            activeCellStatusList [activeCellStatusListCount] = parentTDCount, activeCellStatusListCount++;

                            activeCellStatusList [activeCellStatusListCount] = parentTDTD, activeCellStatusListCount++;

                            activeCellStatusList [activeCellStatusListCount] = parentTDBD, activeCellStatusListCount++;

                            activeCellStatusList [activeCellStatusListCount] = tempListOfCells [counter1*7+1], activeCellStatusListCount++;

                            activeCellStatusList [activeCellStatusListCount] = tempListOfCells [counter1*7+6], activeCellStatusListCount++;

                        }

**if** (tempListOfCells [counter1*7+5]== 2){

                            activeCellStatusList [activeCellStatusListCount] = tempListOfCells [counter1*7+2], activeCellStatusListCount++;

                            activeCellStatusList [activeCellStatusListCount] = tempListOfCells [counter1*7+3], activeCellStatusListCount++;

                            activeCellStatusList [activeCellStatusListCount] = tempListOfCells [counter1*7+4], activeCellStatusListCount++;

                            activeCellStatusList [activeCellStatusListCount] = tempListOfCells [counter1*7], activeCellStatusListCount++;

                            activeCellStatusList [activeCellStatusListCount] = 0, activeCellStatusListCount++;

                            activeCellStatusList [activeCellStatusListCount] = 2, activeCellStatusListCount++;

                            activeCellStatusList [activeCellStatusListCount] = 0, activeCellStatusListCount++;

                            activeCellStatusList [activeCellStatusListCount] = parentTDCount, activeCellStatusListCount++;

                            activeCellStatusList [activeCellStatusListCount] = parentTDTD, activeCellStatusListCount++;

                            activeCellStatusList [activeCellStatusListCount] = parentTDBD, activeCellStatusListCount++;

                            activeCellStatusList [activeCellStatusListCount] = tempListOfCells [counter1*7+1], activeCellStatusListCount++;

                            activeCellStatusList [activeCellStatusListCount] = tempListOfCells [counter1*7+6], activeCellStatusListCount++;

                            activeCellStatusList [activeCellStatusListCount] = tempListOfCells [counter1*7+3], activeCellStatusListCount++;

                            activeCellStatusList [activeCellStatusListCount] = tempListOfCells [counter1*7+2], activeCellStatusListCount++;

                            activeCellStatusList [activeCellStatusListCount] = tempListOfCells [counter1*7+4], activeCellStatusListCount++;

                            activeCellStatusList [activeCellStatusListCount] = tempListOfCells [counter1*7], activeCellStatusListCount++;

                            activeCellStatusList [activeCellStatusListCount] = 0, activeCellStatusListCount++;

                            activeCellStatusList [activeCellStatusListCount] = 2, activeCellStatusListCount++;

                            activeCellStatusList [activeCellStatusListCount] = 0, activeCellStatusListCount++;

                            activeCellStatusList [activeCellStatusListCount] = parentTDCount, activeCellStatusListCount++;

                            activeCellStatusList [activeCellStatusListCount] = parentTDTD, activeCellStatusListCount++;

                            activeCellStatusList [activeCellStatusListCount] = parentTDBD, activeCellStatusListCount++;

                            activeCellStatusList [activeCellStatusListCount] = tempListOfCells [counter1*7+1], activeCellStatusListCount++;

                            activeCellStatusList [activeCellStatusListCount] = tempListOfCells [counter1*7+6], activeCellStatusListCount++;

                            activeCellStatusList [activeCellStatusListCount] = tempListOfCells [counter1*7+4], activeCellStatusListCount++;

                            activeCellStatusList [activeCellStatusListCount] = tempListOfCells [counter1*7+2], activeCellStatusListCount++;

                            activeCellStatusList [activeCellStatusListCount] = tempListOfCells [counter1*7+3], activeCellStatusListCount++;

                            activeCellStatusList [activeCellStatusListCount] = tempListOfCells [counter1*7], activeCellStatusListCount++;

                            activeCellStatusList [activeCellStatusListCount] = 0, activeCellStatusListCount++;

                            activeCellStatusList [activeCellStatusListCount] = 2, activeCellStatusListCount++;

                            activeCellStatusList [activeCellStatusListCount] = 0, activeCellStatusListCount++;

                            activeCellStatusList [activeCellStatusListCount] = parentTDCount, activeCellStatusListCount++;

                            activeCellStatusList [activeCellStatusListCount] = parentTDTD, activeCellStatusListCount++;

                            activeCellStatusList [activeCellStatusListCount] = parentTDBD, activeCellStatusListCount++;

                            activeCellStatusList [activeCellStatusListCount] = tempListOfCells [counter1*7+1], activeCellStatusListCount++;

                            activeCellStatusList [activeCellStatusListCount] = tempListOfCells [counter1*7+6], activeCellStatusListCount++;

                        }

                    }

**int** *activeCellStatusListTemp = **new** **int** [activeCellStatusListCount+100];

                    activeCellStatusListTempCount = 0;

**for** (**int** counter1 = 0; counter1 < activeCellStatusListCount/12; counter1++){

**if** (activeCellStatusList [counter1*12+1] != -1){

                            activeCellStatusListTemp [activeCellStatusListTempCount] = activeCellStatusList [counter1*12], activeCellStatusListTempCount++;

                            activeCellStatusListTemp [activeCellStatusListTempCount] = activeCellStatusList [counter1*12+1], activeCellStatusListTempCount++;

                            activeCellStatusListTemp [activeCellStatusListTempCount] = activeCellStatusList [counter1*12+2], activeCellStatusListTempCount++;

                            activeCellStatusListTemp [activeCellStatusListTempCount] = activeCellStatusList [counter1*12+3], activeCellStatusListTempCount++;

                            activeCellStatusListTemp [activeCellStatusListTempCount] = activeCellStatusList [counter1*12+4], activeCellStatusListTempCount++;

                            activeCellStatusListTemp [activeCellStatusListTempCount] = activeCellStatusList [counter1*12+5], activeCellStatusListTempCount++;

                            activeCellStatusListTemp [activeCellStatusListTempCount] = 0, activeCellStatusListTempCount++;

                            activeCellStatusListTemp [activeCellStatusListTempCount] = activeCellStatusList [counter1*12+7], activeCellStatusListTempCount++;

                            activeCellStatusListTemp [activeCellStatusListTempCount] = activeCellStatusList [counter1*12+8], activeCellStatusListTempCount++;

                            activeCellStatusListTemp [activeCellStatusListTempCount] = activeCellStatusList [counter1*12+9], activeCellStatusListTempCount++;

                            activeCellStatusListTemp [activeCellStatusListTempCount] = activeCellStatusList [counter1*12+10], activeCellStatusListTempCount++;

                            activeCellStatusListTemp [activeCellStatusListTempCount] = activeCellStatusList [counter1*12+11], activeCellStatusListTempCount++;

                        }

                    }

                    activeCellStatusListCount = 0;

**for** (**int** counter1 = 0; counter1 < activeCellStatusListTempCount; counter1++) activeCellStatusList [activeCellStatusListCount] = activeCellStatusListTemp [counter1], activeCellStatusListCount++;

**delete** [] activeCellStatusListTemp;

                }

**delete** [] tempListOfCells;

**delete** [] activeCellStatusListHold;

                activeCellStatusListHold = **new** **int** [activeCellStatusListCount+10];

                activeCellStatusListHoldCount = 0;

                firstTimeAssiginment = 1;

**for** (**int** counter1 = 0; counter1 < activeCellStatusListCount; counter1++) activeCellStatusListHold [activeCellStatusListHoldCount] = activeCellStatusList [counter1], activeCellStatusListHoldCount++;

**if** (arrayOverflow == 1){

**break**;

                }

**if** (activeCellStatusListCount == 0){

                    terminationFlag = 0;

                }

            }

        }

**if** (terminateSimFlag == 1) terminationFlag = 0;

    } **while** (terminationFlag == 1);

**delete** [] expandFirsDVList;

**delete** [] expandDoublingDoubBD;

**delete** [] expandDoublingDoubTD;

**delete** [] expandDoublingDoubCF;

**delete** [] expandBDCD;

**delete** [] expandBDCF;

**delete** [] expandNonCD;

**delete** [] expandBDCFCD;

**delete** [] expandTDCF;

**delete** [] expandTDCFCD;

**delete** [] expandTDCD;

**delete** [] firstEventList;

**delete** [] secondEventBDList;

**delete** [] secondEventBDCFList;

**delete** [] secondEventTDList;

**delete** [] secondEventTDCFList;

**delete** [] activeCellStatusListHold;

**if** (selArraySet == 1){

**delete** [] expandFirsDVListSel;

**delete** [] expandDoublingDoubBDSel;

**delete** [] expandDoublingDoubTDSel;

**delete** [] expandDoublingDoubCFSel;

**delete** [] expandBDCDSel;

**delete** [] expandBDCFSel;

**delete** [] expandNonCDSel;

**delete** [] expandBDCFCDSel;

**delete** [] expandTDCFSel;

**delete** [] expandTDCFCDSel;

**delete** [] expandTDCDSel;

**delete** [] firstEventListSel;

**delete** [] secondEventBDListSel;

**delete** [] secondEventBDCFListSel;

**delete** [] secondEventTDListSel;

**delete** [] secondEventTDCFListSel;

    }

}

-(**void**)secondArraySet{

**int** totalNoOfEntryFirstDV = 0;

**int** totalNoOfEntryDoubBD = 0;

**int** totalNoOfEntryDoubTD = 0;

**int** totalNoOfEntryDoubCF = 0;

**int** totalNoOfNonDivCDSel = 0;

**int** totalNoOfBDCD = 0;

**int** totalNoOfBDCF = 0;

**int** totalNoOfBDCFCD = 0;

**int** totalNoOfTDCF = 0;

**int** totalNoOfTDCFCD = 0;

**int** totalNoOfTDCD = 0;

**for** (**int** counter1 = 0; counter1 < simulationDistributionAddDataCount/11; counter1++){

**if** (simulationDistributionAddData [counter1*11] != 0) totalNoOfBDCD = totalNoOfBDCD+simulationDistributionAddData [counter1*11];

**if** (simulationDistributionAddData [counter1*11+1] != 0) totalNoOfNonDivCDSel = totalNoOfNonDivCDSel+simulationDistributionAddData [counter1*11+1];

**if** (simulationDistributionAddData [counter1*11+2] != 0) totalNoOfBDCF = totalNoOfBDCF+simulationDistributionAddData [counter1*11+2];

**if** (simulationDistributionAddData [counter1*11+3] != 0) totalNoOfBDCFCD = totalNoOfBDCFCD+simulationDistributionAddData [counter1*11+3];

**if** (simulationDistributionAddData [counter1*11+4] != 0) totalNoOfTDCF = totalNoOfTDCF+simulationDistributionAddData [counter1*11+4];

**if** (simulationDistributionAddData [counter1*11+5] != 0) totalNoOfTDCFCD = totalNoOfTDCFCD+simulationDistributionAddData [counter1*11+5];

**if** (simulationDistributionAddData [counter1*11+6] != 0) totalNoOfTDCD = totalNoOfTDCD+simulationDistributionAddData [counter1*11+6];

**if** (simulationDistributionAddData [counter1*11+7] != 0) totalNoOfEntryDoubBD = totalNoOfEntryDoubBD+simulationDistributionAddData [counter1*11+7];

**if** (simulationDistributionAddData [counter1*11+8] != 0) totalNoOfEntryDoubTD = totalNoOfEntryDoubTD+simulationDistributionAddData [counter1*11+8];

**if** (simulationDistributionAddData [counter1*11+9] != 0) totalNoOfEntryDoubCF = totalNoOfEntryDoubCF+simulationDistributionAddData [counter1*11+9];

**if** (simulationDistributionAddData [counter1*11+10] != 0) totalNoOfEntryFirstDV = totalNoOfEntryFirstDV+simulationDistributionAddData [counter1*11+10];

    }

    //--------Apply the bias--------

    totalNoOfEntryDoubBD = (**int**)(round(totalNoOfEntryDoubBD*(**double**)simProcessDataAddHold [0]));

    totalNoOfEntryDoubTD = (**int**)(round(totalNoOfEntryDoubTD*(**double**)simProcessDataAddHold [1]));

    totalNoOfEntryDoubCF = (**int**)(round(totalNoOfEntryDoubCF*(**double**)simProcessDataAddHold [2]));

    totalNoOfBDCD = (**int**)(round(totalNoOfBDCD*(**double**)simProcessDataAddHold [5]));

    totalNoOfNonDivCDSel = (**int**)(round(totalNoOfNonDivCDSel*(**double**)simProcessDataAddHold [6]));

    totalNoOfBDCF = (**int**)(round(totalNoOfBDCF*(**double**)simProcessDataAddHold [7]));

    totalNoOfBDCFCD = (**int**)(round(totalNoOfBDCFCD*(**double**)simProcessDataAddHold [10]));

    totalNoOfTDCF = (**int**)(round(totalNoOfTDCF*(**double**)simProcessDataAddHold [11]));

    totalNoOfTDCFCD = (**int**)(round(totalNoOfTDCFCD*(**double**)simProcessDataAddHold [14]));

    totalNoOfTDCD = (**int**)(round(totalNoOfTDCD*(**double**)simProcessDataAddHold [17]));

    //--------Expand the content, e.g. Time 33:3, 33,33,33--------

    expandFirsDVListSel = **new** **int** [totalNoOfEntryFirstDV*2+1];

    expandFirsDVListSelCount = 0;

    expandDoublingDoubBDSel = **new** **int** [totalNoOfEntryDoubBD*2+1];

    expandDoublingDoubBDSelCount = 0;

    expandDoublingDoubTDSel = **new** **int** [totalNoOfEntryDoubTD*2+1];

    expandDoublingDoubTDSelCount = 0;

    expandDoublingDoubCFSel = **new** **int** [totalNoOfEntryDoubCF*2+1];

    expandDoublingDoubCFSelCount = 0;

    expandBDCDSel = **new** **int** [totalNoOfBDCD*2+1];

    expandBDCDSelCount = 0;

    expandBDCFSel = **new** **int** [totalNoOfBDCF*2+1];

    expandBDCFSelCount = 0;

    expandNonCDSel = **new** **int** [totalNoOfNonDivCDSel*2+1];

    expandNonCDSelCount = 0;

    expandBDCFCDSel = **new** **int** [totalNoOfBDCFCD*2+1];

    expandBDCFCDSelCount = 0;

    expandTDCFSel = **new** **int** [totalNoOfTDCF*2+1];

    expandTDCFSelCount = 0;

    expandTDCFCDSel = **new** **int** [totalNoOfTDCFCD*2+1];

    expandTDCFCDSelCount = 0;

    expandTDCDSel = **new** **int** [totalNoOfTDCD*2+1];

    expandTDCDSelCount = 0;

**int** countTemp = 0;

**for** (**int** counter1 = 0; counter1 < simulationDistributionAddDataCount/11; counter1++){

**if** (simulationDistributionAddData [counter1*11] != 0){

**if** (simProcessDataAddHold [5] > 1){

**if** (simulationDistributionAddData [counter1*11] > 1) countTemp = (**int**)(round(simulationDistributionAddData [counter1*11]+(simulationDistributionAddData [counter1*11]-1)*(**double**)simProcessDataAddHold [5]));

**else** countTemp = simulationDistributionAddData [counter1*11];

            }

**else** countTemp = (**int**)(round(simulationDistributionAddData [counter1*11]*(**double**)simProcessDataAddHold [5]));

**for** (**int** counter2 = 0; counter2 < countTemp; counter2++){

                expandBDCDSel [expandBDCDSelCount] = counter1+1, expandBDCDSelCount++;

                expandBDCDSel [expandBDCDSelCount] = 0, expandBDCDSelCount++;

            }

        }

**if** (simulationDistributionAddData [counter1*11+1] != 0){

**if** (simProcessDataAddHold [6] > 1){

**if** (simulationDistributionAddData [counter1*11+1] > 1) countTemp = (**int**)(round(simulationDistributionAddData [counter1*11+1]+(simulationDistributionAddData [counter1*11+1]-1)*(**double**)simProcessDataAddHold [6]));

**else** countTemp = simulationDistributionAddData [counter1*11+1];

            }

**else** countTemp = (**int**)(round(simulationDistributionAddData [counter1*11+1]*(**double**)simProcessDataAddHold [6]));

**for** (**int** counter2 = 0; counter2 < countTemp; counter2++){

                expandNonCDSel [expandNonCDSelCount] = counter1+1, expandNonCDSelCount++;

                expandNonCDSel [expandNonCDSelCount] = 0, expandNonCDSelCount++;

            }

        }

**if** (simulationDistributionAddData [counter1*11+2] != 0){

**if** (simProcessDataAddHold [7] > 1){

**if** (simulationDistributionAddData [counter1*11+2] > 1) countTemp = (**int**)(round(simulationDistributionAddData [counter1*11+2]+(simulationDistributionAddData [counter1*11+2]-1)*(**double**)simProcessDataAddHold [7]));

**else** countTemp = simulationDistributionAddData [counter1*11+2];

            }

**else** countTemp = (**int**)(round(simulationDistributionAddData [counter1*11+2]*(**double**)simProcessDataAddHold [7]));

**for** (**int** counter2 = 0; counter2 < countTemp; counter2++){

                expandBDCFSel [expandBDCFSelCount] = counter1+1, expandBDCFSelCount++;

                expandBDCFSel [expandBDCFSelCount] = 0, expandBDCFSelCount++;

            }

        }

**if** (simulationDistributionAddData [counter1*11+3] != 0){

**if** (simProcessDataAddHold [10] > 1){

**if** (simulationDistributionAddData [counter1*11+3] > 1) countTemp = (**int**)(round(simulationDistributionAddData [counter1*11+3]+(simulationDistributionAddData [counter1*11+3]-1)*(**double**)simProcessDataAddHold [10]));

**else** countTemp = simulationDistributionAddData [counter1*11+3];

            }

**else** countTemp = (**int**)(round(simulationDistributionAddData [counter1*11+3]*(**double**)simProcessDataAddHold [10]));

**for** (**int** counter2 = 0; counter2 < countTemp; counter2++){

                expandBDCFCDSel [expandBDCFCDSelCount] = counter1+1, expandBDCFCDSelCount++;

                expandBDCFCDSel [expandBDCFCDSelCount] = 0, expandBDCFCDSelCount++;

            }

        }

**if** (simulationDistributionAddData [counter1*11+4] != 0){

**if** (simProcessDataAddHold [11] > 1){

**if** (simulationDistributionAddData [counter1*11+4] > 1) countTemp = (**int**)(round(simulationDistributionAddData [counter1*11+4]+(simulationDistributionAddData [counter1*11+4]-1)*(**double**)simProcessDataAddHold [11]));

**else** countTemp = simulationDistributionAddData [counter1*11+4];

            }

**else** countTemp = (**int**)(round(simulationDistributionAddData [counter1*11+4]*(**double**)simProcessDataAddHold [11]));

**for** (**int** counter2 = 0; counter2 < countTemp; counter2++){

                expandTDCFSel [expandTDCFSelCount] = counter1+1, expandTDCFSelCount++;

                expandTDCFSel [expandTDCFSelCount] = 0, expandTDCFSelCount++;

            }

        }

**if** (simulationDistributionAddData [counter1*11+5] != 0){

**if** (simProcessDataAddHold [14] > 1){

**if** (simulationDistributionAddData [counter1*11+5] > 1) countTemp = (**int**)(round(simulationDistributionAddData [counter1*11+5]+(simulationDistributionAddData [counter1*11+5]-1)*(**double**)simProcessDataAddHold [14]));

**else** countTemp = simulationDistributionAddData [counter1*11+5];

            }

**else** countTemp = (**int**)(round(simulationDistributionAddData [counter1*11+5]*(**double**)simProcessDataAddHold [14]));

**for** (**int** counter2 = 0; counter2 < countTemp; counter2++){

                expandTDCFCDSel [expandTDCFCDSelCount] = counter1+1, expandTDCFCDSelCount++;

                expandTDCFCDSel [expandTDCFCDSelCount] = 0, expandTDCFCDSelCount++;

            }

        }

**if** (simulationDistributionAddData [counter1*11+6] != 0){

**if** (simProcessDataAddHold [17] > 1){

**if** (simulationDistributionAddData [counter1*11+6] > 1) countTemp = (**int**)(round(simulationDistributionAddData [counter1*11+6]+(simulationDistributionAddData [counter1*11+6]-1)*(**double**)simProcessDataAddHold [17]));

**else** countTemp = simulationDistributionAddData [counter1*11+6];

            }

**else** countTemp = (**int**)(round(simulationDistributionAddData [counter1*11+6]*(**double**)simProcessDataAddHold [17]));

**for** (**int** counter2 = 0; counter2 < countTemp; counter2++){

                expandTDCDSel [expandTDCDSelCount] = counter1+1, expandTDCDSelCount++;

                expandTDCDSel [expandTDCDSelCount] = 0, expandTDCDSelCount++;

            }

        }

**if** (simulationDistributionAddData [counter1*11+7] != 0){ //--------

**if** (simProcessDataAddHold [0] > 1){

**if** (simulationDistributionAddData [counter1*11+7] > 1) countTemp = (**int**)(round(simulationDistributionAddData [counter1*11+7]+(simulationDistributionAddData [counter1*11+7]-1)*(**double**)simProcessDataAddHold [0]));

**else** countTemp = simulationDistributionAddData [counter1*11+7];

            }

**else** countTemp = (**int**)(round(simulationDistributionAddData [counter1*11+7]*(**double**)simProcessDataAddHold [0]));

**for** (**int** counter2 = 0; counter2 < countTemp; counter2++){

                expandDoublingDoubBDSel [expandDoublingDoubBDSelCount] = counter1+1, expandDoublingDoubBDSelCount++;

                expandDoublingDoubBDSel [expandDoublingDoubBDSelCount] = 0, expandDoublingDoubBDSelCount++;

            }

        }

**if** (simulationDistributionAddData [counter1*11+8] != 0){

**if** (simProcessDataAddHold [1] > 1){

**if** (simulationDistributionAddData [counter1*11+8] > 1) countTemp = (**int**)(round(simulationDistributionAddData [counter1*11+8]+(simulationDistributionAddData [counter1*11+8]-1)*(**double**)simProcessDataAddHold [1]));

**else** countTemp = simulationDistributionAddData [counter1*11+8];

            }

**else** countTemp = (**int**)(round(simulationDistributionAddData [counter1*11+8]*(**double**)simProcessDataAddHold [1]));

**for** (**int** counter2 = 0; counter2 < countTemp; counter2++){

                expandDoublingDoubTDSel [expandDoublingDoubTDSelCount] = counter1+1, expandDoublingDoubTDSelCount++;

                expandDoublingDoubTDSel [expandDoublingDoubTDSelCount] = 0, expandDoublingDoubTDSelCount++;

            }

        }

**if** (simulationDistributionAddData [counter1*11+9] != 0){

**if** (simProcessDataAddHold [2] > 1){

**if** (simulationDistributionAddData [counter1*11+9] > 1) countTemp = (**int**)(round(simulationDistributionAddData [counter1*11+9]+(simulationDistributionAddData [counter1*11+9]-1)*(**double**)simProcessDataAddHold [2]));

**else** countTemp = simulationDistributionAddData [counter1*11+9];

            }

**else** countTemp = (**int**)(round(simulationDistributionAddData [counter1*11+9]*(**double**)simProcessDataAddHold [2]));

**for** (**int** counter2 = 0; counter2 < countTemp; counter2++){

                expandDoublingDoubCFSel [expandDoublingDoubCFSelCount] = counter1+1, expandDoublingDoubCFSelCount++;

                expandDoublingDoubCFSel [expandDoublingDoubCFSelCount] = 0, expandDoublingDoubCFSelCount++;

            }

        }

**if** (simulationDistributionAddData [counter1*11+10] != 0){

**for** (**int** counter2 = 0; counter2 < simulationDistributionAddData [counter1*11+10]; counter2++){

                expandFirsDVListSel [expandFirsDVListSelCount] = counter1+1, expandFirsDVListSelCount++;

                expandFirsDVListSel [expandFirsDVListSelCount] = 0, expandFirsDVListSelCount++;

            }

        }

    }

    firstEventListSel = **new** **int** [150];

    secondEventBDListSel = **new** **int** [150];

    secondEventBDCFListSel = **new** **int** [150];

    secondEventTDListSel = **new** **int** [150];

    secondEventTDCFListSel = **new** **int** [150];

**for** (**int** counter1 = 0; counter1 < 150; counter1++){

        firstEventListSel [counter1] = 0;

        secondEventBDListSel [counter1] = 0;

        secondEventBDCFListSel [counter1] = 0;

        secondEventTDListSel [counter1] = 0;

        secondEventTDCFListSel [counter1] = 0;

    }

    selArraySet = 1;

**int** totalNoOfNonDivCDWithBias = (**int**)(round(totalNoOfNonDivCDSel*(**double**)simProcessDataAddHold [6]));

**int** totalNumberOfnonDivLingCD = (**int**)simProcessDataAddHold [20]+totalNoOfNonDivCDWithBias;

**int** remainingLingNo = (**int**)simProcessDataAddHold [24]-totalNumberOfnonDivLingCD;

**if** (remainingLingNo < 0) remainingLingNo = 0;

**int** totalNoOfBDTD = (**int**)(simProcessDataAddHold [3]+simProcessDataAddHold [4]);

**double** nonDivIn100 = totalNumberOfnonDivLingCD/(**double**)(remainingLingNo+totalNumberOfnonDivLingCD);

**double** divIn100 = remainingLingNo/(**double**)(remainingLingNo+totalNumberOfnonDivLingCD);

**int** percentBD = 0;

**int** percentTD = 0;

**int** percentCD = 0;

**int** nonDivPercent = 0;

**double** percentTemp = 0;

**if** (totalNoOfBDTD != 0){

        percentTemp = (simProcessDataAddHold [3]/(**double**)totalNoOfBDTD)*100;

        percentBD = (**int**)(round((percentTemp*divIn100)));

        percentTemp = (simProcessDataAddHold [4]/(**double**)totalNoOfBDTD)*100;

        percentTD = (**int**)(round((percentTemp*divIn100)));

    }

**if** (totalNumberOfnonDivLingCD != 0){

        percentTemp = (totalNoOfNonDivCDWithBias/(**double**)totalNumberOfnonDivLingCD)*100;

        percentCD = (**int**)(round(percentTemp*nonDivIn100));

        percentTemp = (simProcessDataAddHold [20]/(**double**)totalNumberOfnonDivLingCD)*100;

        nonDivPercent = (**int**)(round(percentTemp*nonDivIn100));

    }

**if** (totalNoOfNonDivCDSel > simProcessDataAddHold [4] && percentCD < percentTD){

        percentBD = (percentBD+percentTD)-percentCD;

        percentTD = percentCD;

    }

**else** **if** (totalNoOfNonDivCDSel < simProcessDataAddHold [4] && percentCD > percentTD){

        nonDivPercent = (percentCD+nonDivPercent)-percentTD;

        percentCD = percentTD;

    }

**int** entryCount = 0; //--------Enter type values floowing the %

**for** (**int** counter1 = 0; counter1 < nonDivPercent; counter1++){

**if** (entryCount < 100) firstEventListSel [entryCount] = 4, entryCount++;

    }

**for** (**int** counter1 = 0; counter1 < percentCD; counter1++){

**if** (entryCount < 100) firstEventListSel [entryCount] = 3, entryCount++;

    }

**for** (**int** counter1 = 0; counter1 < percentTD; counter1++){

**if** (entryCount < 100) firstEventListSel [entryCount] = 2, entryCount++;

    }

**for** (**int** counter1 = 0; counter1 < percentBD; counter1++){

**if** (entryCount < 100) firstEventListSel [entryCount] = 1, entryCount++;

    }

**if** (entryCount != 100){

**for** (**int** counter1 = 0; counter1 < 100; counter1++){

**if** (entryCount < 100) firstEventListSel [entryCount] = 1, entryCount++;

        }

    }

**int** totalNoOfAfterBD = (**int**)(simProcessDataAddHold [3]+simProcessDataAddHold [4])+totalNoOfBDCD+totalNoOfBDCF;

    percentBD = 0;

    percentTD = 0;

**int** percentBDCD = 0;

**int** percentBDCF = 0;

**if** (totalNoOfAfterBD != 0) percentBD = (**int**)(round((simProcessDataAddHold [3]/(**double**)totalNoOfAfterBD)*100));

**if** (totalNoOfAfterBD != 0) percentTD = (**int**)(round((simProcessDataAddHold [4]/(**double**)totalNoOfAfterBD)*100));

**if** (totalNoOfAfterBD != 0) percentBDCD = (**int**)(round((totalNoOfBDCD/(**double**)totalNoOfAfterBD)*100));

**if** (totalNoOfAfterBD != 0) percentBDCF = (**int**)(round((totalNoOfBDCF/(**double**)totalNoOfAfterBD)*100));

**if** (totalNoOfAfterBD != 0){

**if** ((simProcessDataAddHold [3]/(**double**)totalNoOfAfterBD)*100 != 0 && percentBD == 0){

            percentBD = 1;

**if** (percentTD > 2) percentTD--;

**else** **if** (percentBDCD > 2) percentBDCD--;

**else** **if** (percentBDCF > 2) percentBDCF--;

        }

**if** ((simProcessDataAddHold [4]/(**double**)totalNoOfAfterBD)*100 != 0 && percentTD == 0){

            percentTD = 1;

**if** (percentBD > 2) percentBD--;

**else** **if** (percentBDCD > 2) percentBDCD--;

**else** **if** (percentBDCF > 2) percentBDCF--;

        }

**if** ((totalNoOfBDCD/(**double**)totalNoOfAfterBD)*100 != 0 && percentBDCD == 0){

            percentBDCD = 1;

**if** (percentBD > 2) percentBD--;

**else** **if** (percentTD > 2) percentTD--;

**else** **if** (percentBDCF > 2) percentBDCF--;

        }

**if** ((totalNoOfBDCF/(**double**)totalNoOfAfterBD)*100 != 0 && percentBDCF == 0){

            percentBDCF = 1;

**if** (percentBD > 2) percentBD--;

**else** **if** (percentTD > 2) percentTD--;

**else** **if** (percentBDCD > 2) percentBDCD--;

        }

    }

    entryCount = 0;

**for** (**int** counter1 = 0; counter1 < percentBDCF; counter1++){

**if** (entryCount < 100) secondEventBDListSel [entryCount] = 6, entryCount++;

    }

**for** (**int** counter1 = 0; counter1 < percentBDCD; counter1++){

**if** (entryCount < 100) secondEventBDListSel [entryCount] = 5, entryCount++;

    }

**for** (**int** counter1 = 0; counter1 < percentTD; counter1++){

**if** (entryCount < 100) secondEventBDListSel [entryCount] = 2, entryCount++;

    }

**for** (**int** counter1 = 0; counter1 < percentBD; counter1++){

**if** (entryCount < 100) secondEventBDListSel [entryCount] = 1, entryCount++;

    }

**if** (entryCount != 100){

**for** (**int** counter1 = 0; counter1 < 100; counter1++){

**if** (entryCount < 100) secondEventBDListSel [entryCount] = 1, entryCount++;

        }

    }

**int** totalNoOfAfterBDCF = (**int**)(simProcessDataAddHold [8]+simProcessDataAddHold [9])+totalNoOfBDCFCD;

**int** percentBDCFBD = 0;

**int** percentBDCFTD = 0;

**int** percentBDCFCD = 0;

**if** (totalNoOfAfterBDCF != 0) percentBDCFBD = (**int**)(round((simProcessDataAddHold [8]/(**double**)totalNoOfAfterBDCF)*100));

**if** (totalNoOfAfterBDCF != 0) percentBDCFTD = (**int**)(round((simProcessDataAddHold [9]/(**double**)totalNoOfAfterBDCF)*100));

**if** (totalNoOfAfterBDCF != 0) percentBDCFCD = (**int**)(round((totalNoOfBDCFCD/(**double**)totalNoOfAfterBDCF)*100));

**if** (totalNoOfAfterBDCF != 0){

**if** ((simProcessDataAddHold [8]/(**double**)totalNoOfAfterBDCF)*100 != 0 && percentBDCFBD == 0){

            percentBDCFBD = 1;

**if** (percentBDCFTD > 2) percentBDCFTD--;

**else** **if** (percentBDCFCD > 2) percentBDCFCD--;

        }

**if** ((simProcessDataAddHold [9]/(**double**)totalNoOfAfterBDCF)*100 != 0 && percentBDCFTD == 0){

            percentBDCFTD = 1;

**if** (percentBDCFBD > 2) percentBDCFBD--;

**else** **if** (percentBDCFCD > 2) percentBDCFCD--;

        }

**if** ((totalNoOfBDCFCD/(**double**)totalNoOfAfterBDCF)*100 != 0 && percentBDCFCD == 0){

            percentBDCFCD = 1;

**if** (percentBDCFBD > 2) percentBDCFBD--;

**else** **if** (percentBDCFTD > 2) percentBDCFTD--;

        }

    }

    entryCount = 0;

**int** lastEntry = 0;

**for** (**int** counter1 = 0; counter1 < percentBDCFBD; counter1++){

**if** (entryCount < 100){

            secondEventBDCFListSel [entryCount] = 7, entryCount++;

            lastEntry = 7;

        }

    }

**for** (**int** counter1 = 0; counter1 < percentBDCFTD; counter1++){

**if** (entryCount < 100){

            secondEventBDCFListSel [entryCount] = 8, entryCount++;

            lastEntry = 8;

        }

    }

**for** (**int** counter1 = 0; counter1 < percentBDCFCD; counter1++){

**if** (entryCount < 100){

            secondEventBDCFListSel [entryCount] = 9, entryCount++;

            lastEntry = 9;

        }

    }

**if** (entryCount != 0 && entryCount != 100){

**for** (**int** counter1 = 0; counter1 < 100; counter1++){

**if** (entryCount < 100) secondEventBDCFListSel [entryCount] = lastEntry, entryCount++;

        }

    }

**int** totalNoOfAfterTD = (**int**)(simProcessDataAddHold [15]+simProcessDataAddHold [16])+totalNoOfTDCD+totalNoOfTDCF;

**int** percentTDCF = 0;

**int** percentTDBD = 0;

**int** percentTDTD = 0;

**int** percentTDCD = 0;

**if** (totalNoOfAfterTD != 0) percentTDBD = (**int**)(round((simProcessDataAddHold [15]/(**double**)totalNoOfAfterTD)*100));

**if** (totalNoOfAfterTD != 0) percentTDTD = (**int**)(round((simProcessDataAddHold [16]/(**double**)totalNoOfAfterTD)*100));

**if** (totalNoOfAfterTD != 0) percentTDCD = (**int**)(round((totalNoOfTDCD/(**double**)totalNoOfAfterTD)*100));

**if** (totalNoOfAfterTD != 0) percentTDCF = (**int**)(round((totalNoOfTDCF/(**double**)totalNoOfAfterTD)*100));

**if** (totalNoOfAfterTD != 0){

**if** ((simProcessDataAddHold [15]/(**double**)totalNoOfAfterTD)*100 != 0 && percentTDBD == 0){

            percentTDBD = 1;

**if** (percentTDTD > 2) percentTDTD--;

**else** **if** (percentTDCD > 2) percentTDCD--;

**else** **if** (percentTDCF > 2) percentTDCF--;

        }

**if** ((simProcessDataAddHold [16]/(**double**)totalNoOfAfterTD)*100 != 0 && percentTDTD == 0){

            percentTDTD = 1;

**if** (percentTDBD > 2) percentTDBD--;

**else** **if** (percentTDCD > 2) percentTDCD--;

**else** **if** (percentTDCF > 2) percentTDCF--;

        }

**if** ((totalNoOfTDCD/(**double**)totalNoOfAfterTD)*100 != 0 && percentTDCD == 0){

            percentTDCD = 1;

**if** (percentTDBD > 2) percentTDBD--;

**else** **if** (percentTDTD > 2) percentTDTD--;

**else** **if** (percentTDCF > 2) percentTDCF--;

        }

**if** ((totalNoOfTDCF/(**double**)totalNoOfAfterTD)*100 != 0 && percentTDCF == 0){

            percentTDCF = 1;

**if** (percentTDBD > 2) percentTDBD--;

**else** **if** (percentTDTD > 2) percentTDTD--;

**else** **if** (percentTDCD > 2) percentTDCD--;

        }

    }

    entryCount = 0;

    lastEntry = 0;

**for** (**int** counter1 = 0; counter1 < percentTDCF; counter1++){

**if** (entryCount < 100){

            secondEventTDListSel [entryCount] = 10, entryCount++;

            lastEntry = 10;

        }

    }

**for** (**int** counter1 = 0; counter1 < percentTDBD; counter1++){

**if** (entryCount < 100){

            secondEventTDListSel [entryCount] = 11, entryCount++;

            lastEntry = 11;

        }

    }

**for** (**int** counter1 = 0; counter1 < percentTDTD; counter1++){

**if** (entryCount < 100){

            secondEventTDListSel [entryCount] = 12, entryCount++;

            lastEntry = 12;

        }

    }

**for** (**int** counter1 = 0; counter1 < percentTDCD; counter1++){

**if** (entryCount < 100){

            secondEventTDListSel [entryCount] = 13, entryCount++;

            lastEntry = 13;

        }

    }

**if** (entryCount != 0 && entryCount != 100){

**for** (**int** counter1 = 0; counter1 < 100; counter1++){

**if** (entryCount < 100) secondEventTDListSel [entryCount] = lastEntry, entryCount++;

        }

    }

**int** totalNoOfAfterTDCF = (**int**)(simProcessDataAddHold [12]+simProcessDataAddHold [13])+totalNoOfTDCFCD;

**int** percentTDCFBD = 0;

**int** percentTDCFTD = 0;

**int** percentTDCFCD = 0;

**if** (totalNoOfAfterTDCF != 0) percentTDCFBD = (**int**)(round((simProcessDataAddHold [12]/(**double**)totalNoOfAfterTDCF)*100));

**if** (totalNoOfAfterTDCF != 0) percentTDCFTD = (**int**)(round((simProcessDataAddHold [13]/(**double**)totalNoOfAfterTDCF)*100));

**if** (totalNoOfAfterTDCF != 0) percentTDCFCD = (**int**)(round((totalNoOfTDCFCD/(**double**)totalNoOfAfterTDCF)*100));

**if** (totalNoOfAfterTDCF != 0){

**if** ((simProcessDataAddHold [12]/(**double**)totalNoOfAfterTDCF)*100 != 0 && percentTDCFBD == 0){

            percentTDCFBD = 1;

**if** (percentTDCFTD > 2) percentTDCFTD--;

**else** **if** (percentTDCFCD > 2) percentTDCFCD--;

        }

**if** ((simProcessDataAddHold [13]/(**double**)totalNoOfAfterTDCF)*100 != 0&& percentTDCFTD == 0){

            percentTDCFTD = 1;

**if** (percentTDCFBD > 2) percentTDCFBD--;

**else** **if** (percentTDCFCD > 2) percentTDCFCD--;

        }

**if** ((totalNoOfTDCFCD/(**double**)totalNoOfAfterTDCF)*100 != 0 && percentTDCFCD == 0){

            percentTDCFCD = 1;

**if** (percentTDCFBD > 2) percentTDCFBD--;

**else** **if** (percentTDCFTD > 2) percentTDCFTD--;

        }

    }

    entryCount = 0;

    lastEntry = 0;

**for** (**int** counter1 = 0; counter1 < percentTDCFBD; counter1++){

**if** (entryCount < 100){

            secondEventTDCFListSel [entryCount] = 14, entryCount++;

            lastEntry = 14;

        }

    }

**for** (**int** counter1 = 0; counter1 < percentTDCFTD; counter1++){

**if** (entryCount < 100){

            secondEventTDCFListSel [entryCount] = 15, entryCount++;

            lastEntry = 15;

        }

    }

**for** (**int** counter1 = 0; counter1 < percentTDCFCD; counter1++){

**if** (entryCount < 100){

            secondEventTDCFListSel [entryCount] = 16, entryCount++;

            lastEntry = 16;

        }

    }

**if** (entryCount != 0 && entryCount != 100){

**for** (**int** counter1 = 0; counter1 < 100; counter1++){

**if** (entryCount < 100) secondEventTDCFListSel [entryCount] = lastEntry, entryCount++;

        }

    }

    randBDRangeSelA = (**int**)(round(simProcessDataAddHold [26]-simProcessDataAddHold [26]*0.25));

    randBDRangeSelB = (**int**)(round(simProcessDataAddHold [26]+simProcessDataAddHold [26]*0.25));

    randCDRangeSelA = (**int**)(round(simProcessDataAddHold [27]-simProcessDataAddHold [27]*0.25));

    randCDRangeSelB = (**int**)(round(simProcessDataAddHold [27]+simProcessDataAddHold [27]*0.25));

**if** (randBDRangeSelA == 0 && randBDRangeSelB == 0){

        randBDRangeSelA = (**int**)(round(simProcessDataAddHold [25]*0.8-simProcessDataAddHold [25]*0.8*0.25));

        randBDRangeSelB = (**int**)(round(simProcessDataAddHold [25]*0.8));

    }

**if** (randCDRangeSelA == 0 && randCDRangeSelB == 0){

        randCDRangeSelA = (**int**)(round(simProcessDataAddHold [25]*0.5-simProcessDataAddHold [25]*0.5*0.25));

        randCDRangeSelB = (**int**)(round(simProcessDataAddHold [25]*0.5));

    }

}

**@end**

//

//  SimulationPerformMid.m

//  Lineage_Analysis

//

//  Created by Masahiko Sato on 2020-11-27.

//

#import "SimulationPerformMid.h"

/*

 Simulation: the second condition

 The following code is written in Objective-C/C++ using Xcode.

 Comments: see SimulationPerform

 */

**@implementation** SimulationPerformMid

-(**void**)midPerform:(**int**)growthCycleMid :(**int**)growthCycleProgEnd :(**int**)growthCycleProgEnd2{

**int** growthCycleBase = growthCycleMid;

    selArraySet = 0;

**int** totalNoOfEntryFirstDV = 0;

**int** totalNoOfEntryDoubBD = 0;

**int** totalNoOfEntryDoubTD = 0;

**int** totalNoOfEntryDoubCF = 0;

**int** totalNoOfNonDivCD = 0;

**int** totalNoOfBDCD = 0;

**int** totalNoOfBDCF = 0;

**int** totalNoOfBDCFCD = 0;

**int** totalNoOfTDCF = 0;

**int** totalNoOfTDCFCD = 0;

**int** totalNoOfTDCD = 0;

**for** (**int** counter1 = 0; counter1 < simulationDistributionMidDataCount/11; counter1++){

**if** (simulationDistributionMidData [counter1*11] != 0) totalNoOfBDCD = totalNoOfBDCD+simulationDistributionMidData [counter1*11];

**if** (simulationDistributionMidData [counter1*11+1] != 0) totalNoOfNonDivCD = totalNoOfNonDivCD+simulationDistributionMidData [counter1*11+1];

**if** (simulationDistributionMidData [counter1*11+2] != 0) totalNoOfBDCF = totalNoOfBDCF+simulationDistributionMidData [counter1*11+2];

**if** (simulationDistributionMidData [counter1*11+3] != 0) totalNoOfBDCFCD = totalNoOfBDCFCD+simulationDistributionMidData [counter1*11+3];

**if** (simulationDistributionMidData [counter1*11+4] != 0) totalNoOfTDCF = totalNoOfTDCF+simulationDistributionMidData [counter1*11+4];

**if** (simulationDistributionMidData [counter1*11+5] != 0) totalNoOfTDCFCD = totalNoOfTDCFCD+simulationDistributionMidData [counter1*11+5];

**if** (simulationDistributionMidData [counter1*11+6] != 0) totalNoOfTDCD = totalNoOfTDCD+simulationDistributionMidData [counter1*11+6];

**if** (simulationDistributionMidData [counter1*11+7] != 0) totalNoOfEntryDoubBD = totalNoOfEntryDoubBD+simulationDistributionMidData [counter1*11+7];

**if** (simulationDistributionMidData [counter1*11+8] != 0) totalNoOfEntryDoubTD = totalNoOfEntryDoubTD+simulationDistributionMidData [counter1*11+8];

**if** (simulationDistributionMidData [counter1*11+9] != 0) totalNoOfEntryDoubCF = totalNoOfEntryDoubCF+simulationDistributionMidData [counter1*11+9];

**if** (simulationDistributionMidData [counter1*11+10] != 0) totalNoOfEntryFirstDV = totalNoOfEntryFirstDV+simulationDistributionMidData [counter1*11+10];

    }

    totalNoOfEntryDoubBD = (**int**)(round(totalNoOfEntryDoubBD*(**double**)simProcessDataMiddleHold [0]));

    totalNoOfEntryDoubTD = (**int**)(round(totalNoOfEntryDoubTD*(**double**)simProcessDataMiddleHold [1]));

    totalNoOfEntryDoubCF = (**int**)(round(totalNoOfEntryDoubCF*(**double**)simProcessDataMiddleHold [2]));

    totalNoOfBDCD = (**int**)(round(totalNoOfBDCD*(**double**)simProcessDataMiddleHold [5]));

    totalNoOfNonDivCD = (**int**)(round(totalNoOfNonDivCD*(**double**)simProcessDataMiddleHold [6]));

    totalNoOfBDCF = (**int**)(round(totalNoOfBDCF*(**double**)simProcessDataMiddleHold [7]));

    totalNoOfBDCFCD = (**int**)(round(totalNoOfBDCFCD*(**double**)simProcessDataMiddleHold [10]));

    totalNoOfTDCF = (**int**)(round(totalNoOfTDCF*(**double**)simProcessDataMiddleHold [11]));

    totalNoOfTDCFCD = (**int**)(round(totalNoOfTDCFCD*(**double**)simProcessDataMiddleHold [14]));

    totalNoOfTDCD = (**int**)(round(totalNoOfTDCD*(**double**)simProcessDataMiddleHold [17]));

**int** *expandFirsDVList = **new** **int** [totalNoOfEntryFirstDV*2+1];

**int** expandFirsDVListCount = 0;

**int** *expandDoublingDoubBD = **new** **int** [totalNoOfEntryDoubBD*2+1];

**int** expandDoublingDoubBDCount = 0;

**int** *expandDoublingDoubTD = **new** **int** [totalNoOfEntryDoubTD*2+1];

**int** expandDoublingDoubTDCount = 0;

**int** *expandDoublingDoubCF = **new** **int** [totalNoOfEntryDoubCF*2+1];

**int** expandDoublingDoubCFCount = 0;

**int** *expandBDCD = **new** **int** [totalNoOfBDCD*2+1];

**int** expandBDCDCount = 0;

**int** *expandBDCF = **new** **int** [totalNoOfBDCF*2+1];

**int** expandBDCFCount = 0;

**int** *expandNonCD = **new** **int** [totalNoOfNonDivCD*2+1];

**int** expandNonCDCount = 0;

**int** *expandBDCFCD = **new** **int** [totalNoOfBDCFCD*2+1];

**int** expandBDCFCDCount = 0;

**int** *expandTDCF = **new** **int** [totalNoOfTDCF*2+1];

**int** expandTDCFCount = 0;

**int** *expandTDCFCD = **new** **int** [totalNoOfTDCFCD*2+1];

**int** expandTDCFCDCount = 0;

**int** *expandTDCD = **new** **int** [totalNoOfTDCD*2+1];

**int** expandTDCDCount = 0;

**int** countTemp = 0;

**for** (**int** counter1 = 0; counter1 < simulationDistributionMidDataCount/11; counter1++){

**if** (simulationDistributionMidData [counter1*11] != 0){

**if** (simProcessDataMiddleHold [5] > 1){

**if** (simulationDistributionMidData [counter1*11] > 1) countTemp = (**int**)(round(simulationDistributionMidData [counter1*11]+(simulationDistributionMidData [counter1*11]-1)*(**double**)simProcessDataMiddleHold [5]));

**else** countTemp = simulationDistributionMidData [counter1*11];

            }

**else** countTemp = (**int**)(round(simulationDistributionMidData [counter1*11]*(**double**)simProcessDataMiddleHold [5]));

**for** (**int** counter2 = 0; counter2 < countTemp; counter2++){

                expandBDCD [expandBDCDCount] = counter1+1, expandBDCDCount++;

                expandBDCD [expandBDCDCount] = 0, expandBDCDCount++;

            }

        }

**if** (simulationDistributionMidData [counter1*11+1] != 0){

**if** (simProcessDataMiddleHold [6] > 1){

**if** (simulationDistributionMidData [counter1*11+1] > 1) countTemp = (**int**)(round(simulationDistributionMidData [counter1*11+1]+(simulationDistributionData [counter1*11+1]-1)*(**double**)simProcessDataMiddleHold [6]));

**else** countTemp = simulationDistributionMidData [counter1*11+1];

            }

**else** countTemp = (**int**)(round(simulationDistributionMidData [counter1*11+1]*(**double**)simProcessDataMiddleHold [6]));

**for** (**int** counter2 = 0; counter2 < countTemp; counter2++){

                expandNonCD [expandNonCDCount] = counter1+1, expandNonCDCount++;

                expandNonCD [expandNonCDCount] = 0, expandNonCDCount++;

            }

        }

**if** (simulationDistributionMidData [counter1*11+2] != 0){

**if** (simProcessDataMiddleHold [7] > 1){

**if** (simulationDistributionMidData [counter1*11+2] > 1) countTemp = (**int**)(round(simulationDistributionMidData [counter1*11+2]+(simulationDistributionMidData [counter1*11+2]-1)*(**double**)simProcessDataMiddleHold [7]));

**else** countTemp = simulationDistributionMidData [counter1*11+2];

            }

**else** countTemp = (**int**)(round(simulationDistributionMidData [counter1*11+2]*(**double**)simProcessDataMiddleHold [7]));

**for** (**int** counter2 = 0; counter2 < countTemp; counter2++){

                expandBDCF [expandBDCFCount] = counter1+1, expandBDCFCount++;

                expandBDCF [expandBDCFCount] = 0, expandBDCFCount++;

            }

        }

**if** (simulationDistributionMidData [counter1*11+3] != 0){

**if** (simProcessDataMiddleHold [10] > 1){

**if** (simulationDistributionMidData [counter1*11+3] > 1) countTemp = (**int**)(round(simulationDistributionMidData [counter1*11+3]+(simulationDistributionMidData [counter1*11+3]-1)*(**double**)simProcessDataMiddleHold [10]));

**else** countTemp = simulationDistributionMidData [counter1*11+3];

            }

**else** countTemp = (**int**)(round(simulationDistributionMidData [counter1*11+3]*(**double**)simProcessDataMiddleHold [10]));

**for** (**int** counter2 = 0; counter2 < countTemp; counter2++){

                expandBDCFCD [expandBDCFCDCount] = counter1+1, expandBDCFCDCount++;

                expandBDCFCD [expandBDCFCDCount] = 0, expandBDCFCDCount++;

            }

        }

**if** (simulationDistributionMidData [counter1*11+4] != 0){

**if** (simProcessDataMiddleHold [11] > 1){

**if** (simulationDistributionMidData [counter1*11+4] > 1) countTemp = (**int**)(round(simulationDistributionMidData [counter1*11+4]+(simulationDistributionMidData [counter1*11+4]-1)*(**double**)simProcessDataMiddleHold [11]));

**else** countTemp = simulationDistributionMidData [counter1*11+4];

            }

**else** countTemp = (**int**)(round(simulationDistributionMidData [counter1*11+4]*(**double**)simProcessDataMiddleHold [11]));

**for** (**int** counter2 = 0; counter2 < countTemp; counter2++){

                expandTDCF [expandTDCFCount] = counter1+1, expandTDCFCount++;

                expandTDCF [expandTDCFCount] = 0, expandTDCFCount++;

            }

        }

**if** (simulationDistributionMidData [counter1*11+5] != 0){

**if** (simProcessDataMiddleHold [14] > 1){

**if** (simulationDistributionMidData [counter1*11+5] > 1) countTemp = (**int**)(round(simulationDistributionMidData [counter1*11+5]+(simulationDistributionMidData [counter1*11+5]-1)*(**double**)simProcessDataMiddleHold [14]));

**else** countTemp = simulationDistributionMidData [counter1*11+5];

            }

**else** countTemp = (**int**)(round(simulationDistributionMidData [counter1*11+5]*(**double**)simProcessDataMiddleHold [14]));

**for** (**int** counter2 = 0; counter2 < countTemp; counter2++){

                expandTDCFCD [expandTDCFCDCount] = counter1+1, expandTDCFCDCount++;

                expandTDCFCD [expandTDCFCDCount] = 0, expandTDCFCDCount++;

            }

        }

**if** (simulationDistributionMidData [counter1*11+6] != 0){

**if** (simProcessDataMiddleHold [17] > 1){

**if** (simulationDistributionMidData [counter1*11+6] > 1) countTemp = (**int**)(round(simulationDistributionMidData [counter1*11+6]+(simulationDistributionMidData [counter1*11+6]-1)*(**double**)simProcessDataMiddleHold [17]));

**else** countTemp = simulationDistributionMidData [counter1*11+6];

            }

**else** countTemp = (**int**)(round(simulationDistributionMidData [counter1*11+6]*(**double**)simProcessDataMiddleHold [17]));

**for** (**int** counter2 = 0; counter2 < countTemp; counter2++){

                expandTDCD [expandTDCDCount] = counter1+1, expandTDCDCount++;

                expandTDCD [expandTDCDCount] = 0, expandTDCDCount++;

            }

        }

**if** (simulationDistributionMidData [counter1*11+7] != 0){

**if** (simProcessDataMiddleHold [0] > 1){

**if** (simulationDistributionMidData [counter1*11+7] > 1) countTemp = (**int**)(round(simulationDistributionMidData [counter1*11+7]+(simulationDistributionMidData [counter1*11+7]-1)*(**double**)simProcessDataMiddleHold [0]));

**else** countTemp = simulationDistributionMidData [counter1*11+7];

            }

**else** countTemp = (**int**)(round(simulationDistributionMidData [counter1*11+7]*(**double**)simProcessDataMiddleHold [0]));

**for** (**int** counter2 = 0; counter2 < countTemp; counter2++){

                expandDoublingDoubBD [expandDoublingDoubBDCount] = counter1+1, expandDoublingDoubBDCount++;

                expandDoublingDoubBD [expandDoublingDoubBDCount] = 0, expandDoublingDoubBDCount++;

            }

        }

**if** (simulationDistributionMidData [counter1*11+8] != 0){

**if** (simProcessDataMiddleHold [1] > 1){

**if** (simulationDistributionMidData [counter1*11+8] > 1) countTemp = (**int**)(round(simulationDistributionMidData [counter1*11+8]+(simulationDistributionMidData [counter1*11+8]-1)*(**double**)simProcessDataMiddleHold [1]));

**else** countTemp = simulationDistributionMidData [counter1*11+8];

            }

**else** countTemp = (**int**)(round(simulationDistributionMidData [counter1*11+8]*(**double**)simProcessDataMiddleHold [1]));

**for** (**int** counter2 = 0; counter2 < countTemp; counter2++){

                expandDoublingDoubTD [expandDoublingDoubTDCount] = counter1+1, expandDoublingDoubTDCount++;

                expandDoublingDoubTD [expandDoublingDoubTDCount] = 0, expandDoublingDoubTDCount++;

            }

        }

**if** (simulationDistributionMidData [counter1*11+9] != 0){

**if** (simProcessDataMiddleHold [2] > 1){

**if** (simulationDistributionMidData [counter1*11+9] > 1) countTemp = (**int**)(round(simulationDistributionMidData [counter1*11+9]+(simulationDistributionMidData [counter1*11+9]-1)*(**double**)simProcessDataMiddleHold [2]));

**else** countTemp = simulationDistributionMidData [counter1*11+9];

            }

**else** countTemp = (**int**)(round(simulationDistributionMidData [counter1*11+9]*(**double**)simProcessDataMiddleHold [2]));

**for** (**int** counter2 = 0; counter2 < countTemp; counter2++){

                expandDoublingDoubCF [expandDoublingDoubCFCount] = counter1+1, expandDoublingDoubCFCount++;

                expandDoublingDoubCF [expandDoublingDoubCFCount] = 0, expandDoublingDoubCFCount++;

            }

        }

**if** (simulationDistributionMidData [counter1*11+10] != 0){

**for** (**int** counter2 = 0; counter2 < simulationDistributionMidData [counter1*11+10]; counter2++){

                expandFirsDVList [expandFirsDVListCount] = counter1+1, expandFirsDVListCount++;

                expandFirsDVList [expandFirsDVListCount] = 0, expandFirsDVListCount++;

            }

        }

    }

**int** *firstEventList = **new** **int** [150];

**int** *secondEventBDList = **new** **int** [150];

**int** *secondEventBDCFList = **new** **int** [150];

**int** *secondEventTDList = **new** **int** [150];

**int** *secondEventTDCFList = **new** **int** [150];

**for** (**int** counter1 = 0; counter1 < 150; counter1++){

        firstEventList [counter1] = 0;

        secondEventBDList [counter1] = 0;

        secondEventBDCFList [counter1] = 0;

        secondEventTDList [counter1] = 0;

        secondEventTDCFList [counter1] = 0;

    }

**int** totalNoOfNonDivCDWithBias = (**int**)(round(totalNoOfNonDivCD*(**double**)simProcessDataMiddleHold [6]));

**int** totalNumberOfnonDivLingCD = (**int**)simProcessDataMiddleHold [20]+totalNoOfNonDivCDWithBias;

**int** remainingLingNo = (**int**)simProcessDataMiddleHold [24]-totalNumberOfnonDivLingCD;

**if** (remainingLingNo < 0) remainingLingNo = 0;

**int** totalNoOfBDTD = (**int**)(simProcessDataMiddleHold [3]+simProcessDataMiddleHold [4]);

**double** nonDivIn100 = totalNumberOfnonDivLingCD/(**double**)(remainingLingNo+totalNumberOfnonDivLingCD);

**double** divIn100 = remainingLingNo/(**double**)(remainingLingNo+totalNumberOfnonDivLingCD);

**int** percentBD = 0;

**int** percentTD = 0;

**int** percentCD = 0;

**int** nonDivPercent = 0;

**double** percentTemp = 0;

**if** (totalNoOfBDTD != 0){

        percentTemp = (simProcessDataMiddleHold [3]/(**double**)totalNoOfBDTD)*100;

        percentBD = (**int**)(round((percentTemp*divIn100)));

        percentTemp = (simProcessDataMiddleHold [4]/(**double**)totalNoOfBDTD)*100;

        percentTD = (**int**)(round((percentTemp*divIn100)));

    }

**if** (totalNumberOfnonDivLingCD != 0){

        percentTemp = (totalNoOfNonDivCDWithBias/(**double**)totalNumberOfnonDivLingCD)*100;

        percentCD = (**int**)(round(percentTemp*nonDivIn100));

        percentTemp = (simProcessDataMiddleHold [20]/(**double**)totalNumberOfnonDivLingCD)*100;

        nonDivPercent = (**int**)(round(percentTemp*nonDivIn100));

    }

**if** (totalNoOfNonDivCD > simProcessDataMiddleHold [4] && percentCD < percentTD){

        percentBD = (percentBD+percentTD)-percentCD;

        percentTD = percentCD;

    }

**else** **if** (totalNoOfNonDivCD < simProcessDataMiddleHold [4] && percentCD > percentTD){

        nonDivPercent = (percentCD+nonDivPercent)-percentTD;

        percentCD = percentTD;

    }

**int** entryCount = 0;

**for** (**int** counter1 = 0; counter1 < nonDivPercent; counter1++){

**if** (entryCount < 100) firstEventList [entryCount] = 4, entryCount++;

    }

**for** (**int** counter1 = 0; counter1 < percentCD; counter1++){

**if** (entryCount < 100) firstEventList [entryCount] = 3, entryCount++;

    }

**for** (**int** counter1 = 0; counter1 < percentTD; counter1++){

**if** (entryCount < 100) firstEventList [entryCount] = 2, entryCount++;

    }

**for** (**int** counter1 = 0; counter1 < percentBD; counter1++){

**if** (entryCount < 100) firstEventList [entryCount] = 1, entryCount++;

    }

**if** (entryCount != 100){

**for** (**int** counter1 = 0; counter1 < 100; counter1++){

**if** (entryCount < 100) firstEventList [entryCount] = 1, entryCount++;

        }

    }

**int** totalNoOfAfterBD = (**int**)(simProcessDataMiddleHold [3]+simProcessDataMiddleHold [4])+totalNoOfBDCD+totalNoOfBDCF;

    percentBD = 0;

    percentTD = 0;

**int** percentBDCD = 0;

**int** percentBDCF = 0;

**if** (totalNoOfAfterBD != 0) percentBD = (**int**)(round((simProcessDataMiddleHold [3]/(**double**)totalNoOfAfterBD)*100));

**if** (totalNoOfAfterBD != 0) percentTD = (**int**)(round((simProcessDataMiddleHold [4]/(**double**)totalNoOfAfterBD)*100));

**if** (totalNoOfAfterBD != 0) percentBDCD = (**int**)(round((totalNoOfBDCD/(**double**)totalNoOfAfterBD)*100));

**if** (totalNoOfAfterBD != 0) percentBDCF = (**int**)(round((totalNoOfBDCF/(**double**)totalNoOfAfterBD)*100));

**if** (totalNoOfAfterBD != 0){

**if** ((simProcessDataMiddleHold [3]/(**double**)totalNoOfAfterBD)*100 != 0 && percentBD == 0){

            percentBD = 1;

**if** (percentTD > 2) percentTD--;

**else** **if** (percentBDCD > 2) percentBDCD--;

**else** **if** (percentBDCF > 2) percentBDCF--;

        }

**if** ((simProcessDataMiddleHold [4]/(**double**)totalNoOfAfterBD)*100 != 0 && percentTD == 0){

            percentTD = 1;

**if** (percentBD > 2) percentBD--;

**else** **if** (percentBDCD > 2) percentBDCD--;

**else** **if** (percentBDCF > 2) percentBDCF--;

        }

**if** ((totalNoOfBDCD/(**double**)totalNoOfAfterBD)*100 != 0 && percentBDCD == 0){

            percentBDCD = 1;

**if** (percentBD > 2) percentBD--;

**else** **if** (percentTD > 2) percentTD--;

**else** **if** (percentBDCF > 2) percentBDCF--;

        }

**if** ((totalNoOfBDCF/(**double**)totalNoOfAfterBD)*100 != 0 && percentBDCF == 0){

            percentBDCF = 1;

**if** (percentBD > 2) percentBD--;

**else** **if** (percentTD > 2) percentTD--;

**else** **if** (percentBDCD > 2) percentBDCD--;

        }

    }

    entryCount = 0;

**for** (**int** counter1 = 0; counter1 < percentBDCF; counter1++){

**if** (entryCount < 100) secondEventBDList [entryCount] = 6, entryCount++;

    }

**for** (**int** counter1 = 0; counter1 < percentBDCD; counter1++){

**if** (entryCount < 100) secondEventBDList [entryCount] = 5, entryCount++;

    }

**for** (**int** counter1 = 0; counter1 < percentTD; counter1++){

**if** (entryCount < 100) secondEventBDList [entryCount] = 2, entryCount++;

    }

**for** (**int** counter1 = 0; counter1 < percentBD; counter1++){

**if** (entryCount < 100) secondEventBDList [entryCount] = 1, entryCount++;

    }

**if** (entryCount != 100){

**for** (**int** counter1 = 0; counter1 < 100; counter1++){

**if** (entryCount < 100) secondEventBDList [entryCount] = 1, entryCount++;

        }

    }

**int** totalNoOfAfterBDCF = (**int**)(simProcessDataMiddleHold [8]+simProcessDataMiddleHold [9])+totalNoOfBDCFCD;

**int** percentBDCFBD = 0;

**int** percentBDCFTD = 0;

**int** percentBDCFCD = 0;

**if** (totalNoOfAfterBDCF != 0) percentBDCFBD = (**int**)(round((simProcessDataMiddleHold [8]/(**double**)totalNoOfAfterBDCF)*100));

**if** (totalNoOfAfterBDCF != 0) percentBDCFTD = (**int**)(round((simProcessDataMiddleHold [9]/(**double**)totalNoOfAfterBDCF)*100));

**if** (totalNoOfAfterBDCF != 0) percentBDCFCD = (**int**)(round((totalNoOfBDCFCD/(**double**)totalNoOfAfterBDCF)*100));

**if** (totalNoOfAfterBDCF != 0){

**if** ((simProcessDataMiddleHold [8]/(**double**)totalNoOfAfterBDCF)*100 != 0 && percentBDCFBD == 0){

            percentBDCFBD = 1;

**if** (percentBDCFTD > 2) percentBDCFTD--;

**else** **if** (percentBDCFCD > 2) percentBDCFCD--;

        }

**if** ((simProcessDataMiddleHold [9]/(**double**)totalNoOfAfterBDCF)*100 != 0&& percentBDCFTD == 0){

            percentBDCFTD = 1;

**if** (percentBDCFBD > 2) percentBDCFBD--;

**else** **if** (percentBDCFCD > 2) percentBDCFCD--;

        }

**if** ((totalNoOfBDCFCD/(**double**)totalNoOfAfterBDCF)*100 != 0 && percentBDCFCD == 0){

            percentBDCFCD = 1;

**if** (percentBDCFBD > 2) percentBDCFBD--;

**else** **if** (percentBDCFTD > 2) percentBDCFTD--;

        }

    }

    entryCount = 0;

**int** lastEntry = 0;

**for** (**int** counter1 = 0; counter1 < percentBDCFBD; counter1++){

**if** (entryCount < 100){

            secondEventBDCFList [entryCount] = 7, entryCount++;

            lastEntry = 7;

        }

    }

**for** (**int** counter1 = 0; counter1 < percentBDCFTD; counter1++){

**if** (entryCount < 100){

            secondEventBDCFList [entryCount] = 8, entryCount++;

            lastEntry = 8;

        }

    }

**for** (**int** counter1 = 0; counter1 < percentBDCFCD; counter1++){

**if** (entryCount < 100){

            secondEventBDCFList [entryCount] = 9, entryCount++;

            lastEntry = 9;

        }

    }

**if** (entryCount != 0 && entryCount != 100){

**for** (**int** counter1 = 0; counter1 < 100; counter1++){

**if** (entryCount < 100) secondEventBDCFList [entryCount] = lastEntry, entryCount++;

        }

    }

**int** totalNoOfAfterTD = (**int**)(simProcessDataMiddleHold [15]+simProcessDataMiddleHold [16])+totalNoOfTDCD+totalNoOfTDCF;

**int** percentTDCF = 0;

**int** percentTDBD = 0;

**int** percentTDTD = 0;

**int** percentTDCD = 0;

**if** (totalNoOfAfterTD != 0) percentTDBD = (**int**)(round((simProcessDataMiddleHold [15]/(**double**)totalNoOfAfterTD)*100));

**if** (totalNoOfAfterTD != 0) percentTDTD = (**int**)(round((simProcessDataMiddleHold [16]/(**double**)totalNoOfAfterTD)*100));

**if** (totalNoOfAfterTD != 0) percentTDCD = (**int**)(round((totalNoOfTDCD/(**double**)totalNoOfAfterTD)*100));

**if** (totalNoOfAfterTD != 0) percentTDCF = (**int**)(round((totalNoOfTDCF/(**double**)totalNoOfAfterTD)*100));

**if** (totalNoOfAfterTD != 0){

**if** ((simProcessDataMiddleHold [15]/(**double**)totalNoOfAfterTD)*100 != 0 && percentTDBD == 0){

            percentTDBD = 1;

**if** (percentTDTD > 2) percentTDTD--;

**else** **if** (percentTDCD > 2) percentTDCD--;

**else** **if** (percentTDCF > 2) percentTDCF--;

        }

**if** ((simProcessDataMiddleHold [16]/(**double**)totalNoOfAfterTD)*100 != 0 && percentTDTD == 0){

            percentTDTD = 1;

**if** (percentTDBD > 2) percentTDBD--;

**else** **if** (percentTDCD > 2) percentTDCD--;

**else** **if** (percentTDCF > 2) percentTDCF--;

        }

**if** ((totalNoOfTDCD/(**double**)totalNoOfAfterTD)*100 != 0 && percentTDCD == 0){

            percentTDCD = 1;

**if** (percentTDBD > 2) percentTDBD--;

**else** **if** (percentTDTD > 2) percentTDTD--;

**else** **if** (percentTDCF > 2) percentTDCF--;

        }

**if** ((totalNoOfTDCF/(**double**)totalNoOfAfterTD)*100 != 0 && percentTDCF == 0){

            percentTDCF = 1;

**if** (percentTDBD > 2) percentTDBD--;

**else** **if** (percentTDTD > 2) percentTDTD--;

**else** **if** (percentTDCD > 2) percentTDCD--;

        }

    }

    entryCount = 0;

    lastEntry = 0;

**for** (**int** counter1 = 0; counter1 < percentTDCF; counter1++){

**if** (entryCount < 100){

            secondEventTDList [entryCount] = 10, entryCount++;

            lastEntry = 10;

        }

    }

**for** (**int** counter1 = 0; counter1 < percentTDBD; counter1++){

**if** (entryCount < 100){

            secondEventTDList [entryCount] = 11, entryCount++;

            lastEntry = 11;

        }

    }

**for** (**int** counter1 = 0; counter1 < percentTDTD; counter1++){

**if** (entryCount < 100){

            secondEventTDList [entryCount] = 12, entryCount++;

            lastEntry = 12;

        }

    }

**for** (**int** counter1 = 0; counter1 < percentTDCD; counter1++){

**if** (entryCount < 100){

            secondEventTDList [entryCount] = 13, entryCount++;

            lastEntry = 13;

        }

    }

**if** (entryCount != 0 && entryCount != 100){

**for** (**int** counter1 = 0; counter1 < 100; counter1++){

**if** (entryCount < 100) secondEventTDList [entryCount] = lastEntry, entryCount++;

        }

    }

**int** totalNoOfAfterTDCF = (**int**)(simProcessDataMiddleHold [12]+simProcessDataMiddleHold [13])+totalNoOfTDCFCD;

**int** percentTDCFBD = 0;

**int** percentTDCFTD = 0;

**int** percentTDCFCD = 0;

**if** (totalNoOfAfterTDCF != 0) percentTDCFBD = (**int**)(round((simProcessDataMiddleHold [12]/(**double**)totalNoOfAfterTDCF)*100));

**if** (totalNoOfAfterTDCF != 0) percentTDCFTD = (**int**)(round((simProcessDataMiddleHold [13]/(**double**)totalNoOfAfterTDCF)*100));

**if** (totalNoOfAfterTDCF != 0) percentTDCFCD = (**int**)(round((totalNoOfTDCFCD/(**double**)totalNoOfAfterTDCF)*100));

**if** (totalNoOfAfterTDCF != 0){

**if** ((simProcessDataMiddleHold [12]/(**double**)totalNoOfAfterTDCF)*100 != 0 && percentTDCFBD == 0){

            percentTDCFBD = 1;

**if** (percentTDCFTD > 2) percentTDCFTD--;

**else** **if** (percentTDCFCD > 2) percentTDCFCD--;

        }

**if** ((simProcessDataMiddleHold [13]/(**double**)totalNoOfAfterTDCF)*100 != 0&& percentTDCFTD == 0){

            percentTDCFTD = 1;

**if** (percentTDCFBD > 2) percentTDCFBD--;

**else** **if** (percentTDCFCD > 2) percentTDCFCD--;

        }

**if** ((totalNoOfTDCFCD/(**double**)totalNoOfAfterTDCF)*100 != 0 && percentTDCFCD == 0){

            percentTDCFCD = 1;

**if** (percentTDCFBD > 2) percentTDCFBD--;

**else** **if** (percentTDCFTD > 2) percentTDCFTD--;

        }

    }

    entryCount = 0;

    lastEntry = 0;

**for** (**int** counter1 = 0; counter1 < percentTDCFBD; counter1++){

**if** (entryCount < 100){

            secondEventTDCFList [entryCount] = 14, entryCount++;

            lastEntry = 14;

        }

    }

**for** (**int** counter1 = 0; counter1 < percentTDCFTD; counter1++){

**if** (entryCount < 100){

            secondEventTDCFList [entryCount] = 15, entryCount++;

            lastEntry = 15;

        }

    }

**for** (**int** counter1 = 0; counter1 < percentTDCFCD; counter1++){

**if** (entryCount < 100){

            secondEventTDCFList [entryCount] = 16, entryCount++;

            lastEntry = 16;

        }

    }

**if** (entryCount != 0 && entryCount != 100){

**for** (**int** counter1 = 0; counter1 < 100; counter1++){

**if** (entryCount < 100) secondEventTDCFList [entryCount] = lastEntry, entryCount++;

        }

    }

**int** randInit = 0;

**int** extendEnd = 0;

**int** newCellNumber1 = 0;

**int** newCellNumber2 = 0;

**int** newCellNumber3 = 0;

    activeCellStatusListKeepCount = 0;

    string cellNumberExtract;

    string lineageNumberExtract;

    string newExtensionEntryNo;

    string degitExtract;

**if** (doseSimStatusHold != 2){

**int** totalEvent = (**int**)(simProcessDataMiddleHold [3]+simProcessDataMiddleHold [4])+totalNoOfBDCD+totalNoOfBDCF;

**int** percentTotalBD = (**int**)(round(simProcessDataMiddleHold [3]/(**double**)totalEvent));

**int** percentTotalTD = (**int**)(round(simProcessDataMiddleHold [4]/(**double**)totalEvent));

**int** percentTotalCD = (**int**)(round(totalNoOfBDCD/(**double**)totalEvent));

**int** percentTotalCF = (**int**)(round(totalNoOfBDCF/(**double**)totalEvent));

**int** countBD = 0;

**int** countTD = 0;

**int** countCD = 0;

**int** countCF = 0;

**for** (**int** counter2 = 0; counter2 < 1000; counter2++){

            randInit = rand() % 100 + 0;

**if** (secondEventBDList [randInit] == 1) countBD++;

**else** **if** (secondEventBDList [randInit] == 2) countTD++;

**else** **if** (secondEventBDList [randInit] == 5) countCD++;

**else** **if** (secondEventBDList [randInit] == 6) countCF++;

        }

**int** totalCheck = countBD+countTD+countCD+countCF;

**int** percentCheckTD = 0;

**int** percentCheckCD = 0;

**int** percentCheckCF = 0;

**if** (totalCheck != 0){

            percentCheckTD = (**int**)(round(countTD/(**double**)totalCheck));

            percentCheckCD = (**int**)(round(countCD/(**double**)totalCheck));

            percentCheckCF = (**int**)(round(countCF/(**double**)totalCheck));

**int** checkCount = 0;

**if** (percentCheckTD-percentTotalTD < 0 && percentTotalTD-percentCheckTD < percentTotalBD){

                checkCount = percentTotalTD-percentCheckTD;

**for** (**int** counter2 = 0; counter2 < 100; counter2++){

**if** (secondEventBDList [counter2] == 1){

                        secondEventBDList [counter2] = 2;

                        checkCount--;

**if** (checkCount == 0){

**break**;

                        }

                    }

                }

            }

**if** (percentCheckCD-percentTotalCD < 0 && percentTotalCD-percentCheckCD < percentTotalBD-(percentTotalTD-percentCheckTD)){

                checkCount = percentTotalCD-percentCheckCD;

**for** (**int** counter2 = 0; counter2 < 100; counter2++){

**if** (secondEventBDList [counter2] == 1){

                        secondEventBDList [counter2] = 5;

                        checkCount--;

**if** (checkCount == 0){

**break**;

                        }

                    }

                }

            }

**if** (percentCheckCF-percentTotalCF < 0 && percentTotalCF-percentCheckCF < percentTotalBD-(percentTotalTD-percentCheckTD)-(percentTotalCD-percentCheckCD)){

                checkCount = percentTotalCF-percentCheckCF;

**for** (**int** counter2 = 0; counter2 < 100; counter2++){

**if** (secondEventBDList [counter2] == 1){

                        secondEventBDList [counter2] = 6;

                        checkCount--;

**if** (checkCount == 0){

**break**;

                        }

                    }

                }

            }

        }

    }

**else**{

        [**self** secondArraySet];

**int** totalEvent = (**int**)(round(simProcessDataAddHold [3]+simProcessDataAddHold [4]))+totalNoOfBDCD+totalNoOfBDCF;

**int** percentTotalBD = (**int**)(round(simProcessDataAddHold [3]/(**double**)totalEvent));

**int** percentTotalTD = (**int**)(round(simProcessDataAddHold [4]/(**double**)totalEvent));

**int** percentTotalCD = (**int**)(round(totalNoOfBDCD/(**double**)totalEvent));

**int** percentTotalCF = (**int**)(round(totalNoOfBDCF/(**double**)totalEvent));

**int** countBD = 0;

**int** countTD = 0;

**int** countCD = 0;

**int** countCF = 0;

**for** (**int** counter2 = 0; counter2 < 1000; counter2++){

            randInit = rand() % 100 + 0;

**if** (secondEventBDListSel [randInit] == 1) countBD++;

**else** **if** (secondEventBDListSel [randInit] == 2) countTD++;

**else** **if** (secondEventBDListSel [randInit] == 5) countCD++;

**else** **if** (secondEventBDListSel [randInit] == 6) countCF++;

        }

**int** totalCheck = countBD+countTD+countCD+countCF;

**int** percentCheckTD = 0;

**int** percentCheckCD = 0;

**int** percentCheckCF = 0;

**if** (totalCheck != 0){

            percentCheckTD = (**int**)(round(countTD/(**double**)totalCheck));

            percentCheckCD = (**int**)(round(countCD/(**double**)totalCheck));

            percentCheckCF = (**int**)(round(countCF/(**double**)totalCheck));

**int** checkCount = 0;

**if** (percentCheckTD-percentTotalTD < 0 && percentTotalTD-percentCheckTD < percentTotalBD){

                checkCount = percentTotalTD-percentCheckTD;

**for** (**int** counter2 = 0; counter2 < 100; counter2++){

**if** (secondEventBDListSel [counter2] == 1){

                        secondEventBDListSel [counter2] = 2;

                        checkCount--;

**if** (checkCount == 0){

**break**;

                        }

                    }

                }

            }

**if** (percentCheckCD-percentTotalCD < 0 && percentTotalCD-percentCheckCD < percentTotalBD-(percentTotalTD-percentCheckTD)){

                checkCount = percentTotalCD-percentCheckCD;

**for** (**int** counter2 = 0; counter2 < 100; counter2++){

**if** (secondEventBDListSel [counter2] == 1){

                        secondEventBDListSel [counter2] = 5;

                        checkCount--;

**if** (checkCount == 0){

**break**;

                        }

                    }

                }

            }

**if** (percentCheckCF-percentTotalCF < 0 && percentTotalCF-percentCheckCF < percentTotalBD-(percentTotalTD-percentCheckTD)-(percentTotalCD-percentCheckCD)){

                checkCount = percentTotalCF-percentCheckCF;

**for** (**int** counter2 = 0; counter2 < 100; counter2++){

**if** (secondEventBDListSel [counter2] == 1){

                        secondEventBDListSel [counter2] = 6;

                        checkCount--;

**if** (checkCount == 0){

**break**;

                        }

                    }

                }

            }

        }

    }

**int** *activeCellStatusListHold = **new** **int** [activeCellStatusListCount+10];

**int** activeCellStatusListHoldCount = 0;

**for** (**int** counter1 = 0; counter1 < activeCellStatusListCount; counter1++) activeCellStatusListHold [activeCellStatusListHoldCount] = activeCellStatusList [counter1], activeCellStatusListHoldCount++;

**int** terminationFlag = 0;

**int** lowextValue = 0;

**int** highestValue = 0;

**int** eventCount = 0;

**int** eventType = 0;

**int** loopCheck = 0;

**int** siblinFusionCheck1 = 0;

**int** siblinFusionCheck2 = 0;

**int** siblinFusionDoub1 = 0;

**int** siblinFusionDoub2 = 0;

**int** siblinFusionDoubLarge = 0;

**int** siblingCellNo1 = 0;

**int** siblingCellNo2 = 0;

**int** cellDoubLimitCheck = 0;

**int** siblingCellPosition1 = 0;

**int** siblingCellPosition2 = 0;

**int** siblingCellPositionSelect = 0;

**int** siblingCellNoSelect = 0;

**int** siblingCellDoubSelect = 0;

**int** lineageAddTempCount = 0;

**int** lineageAddTempLimit = 0;

**int** parentTDCount = 0;

**int** parentTDTD = 0;

**int** parentTDBD = 0;

**int** fusionCount = 0;

**int** reachMaxDivision = 1000000000;

**int** timeKeep = 0;

**int** arrayOverflow = 0;

**int** newLimit = 0;

**int** startPositionListCount = 0;

**int** cellNoFusionCheckHold = 0;

**int** missingPartnerCheck = 0;

**int** selectChange = 0;

**int** firstTimeAssiginment = 0;

**int** cellNoforSummary = 0;

**int** clingNoforSummary = 0;

**int** fusionForSummary = 0;

**int** cycleMaxReachFlag = 0;

**int** nonDivAssign = 0;

**int** otherDivAssign = 0;

**int** callCount = 0;

**unsigned** **long** setTime = 0;

**double** dataTempDouble = 0;

**unsigned** **long** fusionPartnerListCount = 0;

**unsigned** **long** entryCount2 = 0;

    string cellNumberString;

**int** activeCellStatusListTempCount = 0;

**int** randBDRangeA = (**int**)(round(simProcessDataMiddleHold [26]-simProcessDataMiddleHold [26]*0.25));

**int** randBDRangeB = (**int**)(round(simProcessDataMiddleHold [26]+simProcessDataMiddleHold [26]*0.25));

**int** randCDRangeA = (**int**)(round(simProcessDataMiddleHold [27]-simProcessDataMiddleHold [27]*0.25));

**int** randCDRangeB = (**int**)(round(simProcessDataMiddleHold [27]+simProcessDataMiddleHold [27]*0.25));

**if** (randBDRangeA == 0 && randBDRangeB == 0){

        randBDRangeA = (**int**)(round(simProcessDataMiddleHold [25]*0.8-simProcessDataMiddleHold [25]*0.8*0.25));

        randBDRangeB = (**int**)(round(simProcessDataMiddleHold [25]*0.8));

    }

**if** (randCDRangeA == 0 && randCDRangeB == 0){

        randCDRangeA = (**int**)(round(simProcessDataMiddleHold [25]*0.5-simProcessDataMiddleHold [25]*0.5*0.25));

        randCDRangeB = (**int**)(round(simProcessDataMiddleHold [25]*0.5));

    }

**int** loopCount = 0;

**int** terminate2 = 0;

**int** selectCheck = 0;

**do**{

        processingStatusCall = 1;

        loopCount++;

        processingStatus = "Mid: C"+to_string (loopCount);

        terminationFlag = 1;

        fusionCount = 0;

**for** (**int** counter1 = 0; counter1 < activeCellStatusListCount/12; counter1++){

**if** (terminateSimFlag == 1){

                terminate2 = 1;

**break**;

            }

            selectCheck = 0;

**if** (doseSimStatusHold == 2){

**for** (**int** counter3 = 0; counter3 < lingNoAssigineSimCount; counter3++){

**if** (lingNoAssigineSim [counter3] == activeCellStatusList [counter1*12+3]){

                        selectCheck = 1;

**break**;

                    }

                }

            }

            cellDoubLimitCheck = activeCellStatusList [counter1*12];

**if** (cellDoubLimitCheck < 0) cellDoubLimitCheck = cellDoubLimitCheck*-1;

            cellNumberString = to_string(cellDoubLimitCheck);

**if** (((**int**)cellNumberString.length() == 9 && (cellNumberString.substr(0, 1) == "5" || cellNumberString.substr(0, 1) == "6") && cellNumberString.substr(cellNumberString.length()-1) != "0") || cycleMaxReachFlag == 1){

                activeCellStatusList [counter1*12+4] = 50;

                activeCellStatusList [counter1*12+5] = 9;

                activeCellStatusList [counter1*12+6] = 1;

                cycleMaxReachFlag = 1;

            }

**else**{

**if** (selectCheck == 0){

**if** (activeCellStatusList [counter1*12+5] == 5){

                        dataTempDouble = ((expandNonCDCount/(**double**)2)/(**double**)simProcessDataMiddleHold [24])*100;

                        randInit = rand() % 100 + 0;

**if** (randInit < dataTempDouble && expandNonCDCount != 0){

                            randInit = rand() % expandNonCDCount/2 + 0;

                            activeCellStatusList [counter1*12+4] = expandNonCD [randInit*2];

                            activeCellStatusList [counter1*12+5] = 30;

                            activeCellStatusList [counter1*12+6] = 1;

                        }

**else**{

                            randInit = rand() % (**int**)(round(simProcessDataMiddleHold [25]*(endVariationtHold/(**double**)100))) + 0;

**if** (randInit%2 == 0) activeCellStatusList [counter1*12+4] = (**int**)simProcessDataMiddleHold [25]+randInit;

**else** activeCellStatusList [counter1*12+4] = (**int**)simProcessDataMiddleHold [25]-randInit;

                            activeCellStatusList [counter1*12+5] = 5;

                            activeCellStatusList [counter1*12+6] = 1;

                        }

                    }

**else** **if** (activeCellStatusList [counter1*12+5] == 7){

                        eventType = 1;

**if** (eventType == 1){

**if** (activeCellStatusList [counter1*12+11] == 0){

**if** (expandDoublingDoubBDCount != 0){

                                    randInit = rand() % expandDoublingDoubBDCount/2 + 0;

                                    activeCellStatusList [counter1*12+4] = expandDoublingDoubBD [randInit*2];

                                    activeCellStatusList [counter1*12+5] = 1;

                                    activeCellStatusList [counter1*12+6] = 1;

                                }

**else**{

                                    randInit = rand() % randBDRangeB + randBDRangeA;

                                    activeCellStatusList [counter1*12+4] = randInit;

                                    activeCellStatusList [counter1*12+5] = 7;

                                    activeCellStatusList [counter1*12+6] = 1;

                                }

                            }

**else**{

                                loopCheck = 0;

**for** (**int** counter2 = 0; counter2 < 100; counter2++){

**if** (expandDoublingDoubBDCount != 0){

                                        randInit = rand() % expandDoublingDoubBDCount/2 + 0;

**if** (expandDoublingDoubBD [randInit*2] > activeCellStatusList [counter1*12+11]-50 && expandDoublingDoubBD [randInit*2] < activeCellStatusList [counter1*12+11]+50){

                                            activeCellStatusList [counter1*12+4] = expandDoublingDoubBD [randInit*2];

                                            activeCellStatusList [counter1*12+5] = 1;

                                            activeCellStatusList [counter1*12+6] = 1;

                                            loopCheck = 1;

**break**;

                                        }

                                    }

                                }

**if** (loopCheck == 0){

                                    activeCellStatusList [counter1*12+4] = activeCellStatusList [counter1*12+11];

                                    activeCellStatusList [counter1*12+5] = 7;

                                    activeCellStatusList [counter1*12+6] = 1;

                                }

                            }

                        }

                    }

**else** **if** (activeCellStatusList [counter1*12+5] == 1){

                        siblinFusionCheck1 = 0;

                        siblinFusionDoub1 = 0;

                        siblingCellNo1 = 0;

**for** (**int** counter2 = 0; counter2 < activeCellStatusListCount/12; counter2++){

**if** (activeCellStatusList [counter2*12+6] == 1 && activeCellStatusList [counter2*12+1] == activeCellStatusList [counter1*12] && activeCellStatusList [counter2*12+3] == activeCellStatusList [counter1*12+3]){

                                siblingCellNo1 = counter2;

**if** (activeCellStatusList [counter2*12+5] != 0){

                                    siblinFusionCheck1 = activeCellStatusList [counter2*12+5];

                                    siblinFusionDoub1 = activeCellStatusList [counter2*12+4];

                                }

**break**;

                            }

                        }

                        eventType = 0;

**if** (activeCellStatusList [counter1*12+9] == 0){

**if** (siblinFusionCheck1 == 6){

                                loopCheck = 0;

**for** (**int** counter2 = 0; counter2 < 100; counter2++){

                                    randInit = rand() % 100 + 0;

**if** (secondEventBDList [randInit] != 6){

                                        eventType = secondEventBDList [randInit];

                                        loopCheck = 1;

**break**;

                                    }

                                }

**if** (loopCheck == 0) eventType = 1;

                            }

**else**{

                                randInit = rand() % 100 + 0;

**if** (secondEventBDList [randInit] != 0) eventType = secondEventBDList [randInit];

**else** eventType = 1;

                            }

                        }

**else** **if** (activeCellStatusList [counter1*12+9] != 0){

                            eventCount = 0;

                            eventType = 0;

**if** (siblinFusionCheck1 == 6) eventType = 5;

**else**{

**for** (**int** counter2 = 0; counter2 < 5; counter2++){

                                    randInit = rand() % 100 + 0;

**if** (secondEventBDList [randInit] == 1) eventCount++;

**else**{

                                        eventType = secondEventBDList [randInit];

**break**;

                                    }

                                }

**if** (eventCount == 5 || eventType == 0) eventType = 1;

                            }

                        }

**if** (firstTimeAssiginment == 0 && eventType == 1){

                            randInit = rand() % 100 + 0;

**if** (firstEventList [randInit] != 0){

**if** (firstEventList [randInit] == 3) eventType = 5;

**else** **if** (firstEventList [randInit] == 4) eventType = 10;

**else** eventType = firstEventList [randInit];

                            }

                        }

**if** (eventType == 1){

**if** (activeCellStatusList [counter1*12+11] == 0){

**if** (siblinFusionCheck1 != 6){

**if** (expandDoublingDoubBDCount != 0){

                                        randInit = rand() % expandDoublingDoubBDCount/2 + 0;

                                        activeCellStatusList [counter1*12+4] = expandDoublingDoubBD [randInit*2];

                                        activeCellStatusList [counter1*12+5] = 1;

                                        activeCellStatusList [counter1*12+6] = 1;

**if** (activeCellStatusList [counter1*12+9] != 0) activeCellStatusList [counter1*12+9]--;

                                    }

**else**{

                                        randInit = rand() % randBDRangeB + randBDRangeA;

                                        activeCellStatusList [counter1*12+4] = randInit;

                                        activeCellStatusList [counter1*12+5] = 1;

                                        activeCellStatusList [counter1*12+6] = 1;

**if** (activeCellStatusList [counter1*12+9] != 0) activeCellStatusList [counter1*12+9]--;

                                    }

                                }

**else**{

**if** (siblinFusionDoub1 < randBDRangeA) siblinFusionDoub1 = randBDRangeA;

                                    activeCellStatusList [counter1*12+4] = siblinFusionDoub1+10;

                                    activeCellStatusList [counter1*12+5] = 1;

                                    activeCellStatusList [counter1*12+6] = 1;

**if** (activeCellStatusList [counter1*12+9] != 0) activeCellStatusList [counter1*12+9]--;

                                }

                            }

**else**{

                                loopCheck = 0;

**for** (**int** counter2 = 0; counter2 < 100; counter2++){

**if** (siblinFusionCheck1 != 6){

**if** (expandDoublingDoubBDCount != 0){

                                            randInit = rand() % expandDoublingDoubBDCount/2 + 0;

**if** (expandDoublingDoubBD [randInit*2] > activeCellStatusList [counter1*12+11]-50 && expandDoublingDoubBD [randInit*2] < activeCellStatusList [counter1*12+11]+50){

                                                activeCellStatusList [counter1*12+4] = expandDoublingDoubBD [randInit*2];

                                                activeCellStatusList [counter1*12+5] = 1;

                                                activeCellStatusList [counter1*12+6] = 1;

**if** (activeCellStatusList [counter1*12+9] != 0) activeCellStatusList [counter1*12+9]--;

                                                loopCheck = 1;

**break**;

                                            }

                                        }

                                    }

**else** **if** (siblinFusionCheck1 == 6){

**if** (expandDoublingDoubBDCount != 0){

                                            randInit = rand() % expandDoublingDoubBDCount/2 + 0;

**if** (expandDoublingDoubBD [randInit*2] > activeCellStatusList [counter1*12+11]-50 && expandDoublingDoubBD [randInit*2] < activeCellStatusList [counter1*12+11]+50 && siblinFusionDoub1+10 < expandDoublingDoubBD [randInit*2]){

                                                activeCellStatusList [counter1*12+4] = expandDoublingDoubBD [randInit*2];

                                                activeCellStatusList [counter1*12+5] = 1;

                                                activeCellStatusList [counter1*12+6] = 1;

**if** (activeCellStatusList [counter1*12+9] != 0) activeCellStatusList [counter1*12+9]--;

                                                loopCheck = 1;

**break**;

                                            }

                                        }

                                    }

                                }

**if** (loopCheck == 0){

                                    lowextValue = 100000;

                                    highestValue = 0;

**for** (**int** counter2 = 0; counter2 < expandDoublingDoubBDCount/2; counter2++){

**if** (lowextValue > expandDoublingDoubBD [counter2*2]) lowextValue = expandDoublingDoubBD [counter2*2];

**if** (highestValue < expandDoublingDoubBD [counter2*2]) highestValue = expandDoublingDoubBD [counter2*2];

                                    }

**if** (siblinFusionDoub1 < randBDRangeA) siblinFusionDoub1 = randBDRangeA;

**if** (lowextValue != 100000 && highestValue != 0){

**if** ((highestValue+lowextValue)/(**double**)2 > siblinFusionDoub1+10){

                                            activeCellStatusList [counter1*12+4] = (**int**)(round((highestValue+lowextValue)/(**double**)2));

                                            activeCellStatusList [counter1*12+5] = 1;

                                            activeCellStatusList [counter1*12+6] = 1;

**if** (activeCellStatusList [counter1*12+9] != 0) activeCellStatusList [counter1*12+9]--;

                                        }

**else**{

                                            activeCellStatusList [counter1*12+4] = siblinFusionDoub1+10;

                                            activeCellStatusList [counter1*12+5] = 1;

                                            activeCellStatusList [counter1*12+6] = 1;

**if** (activeCellStatusList [counter1*12+9] != 0) activeCellStatusList [counter1*12+9]--;

                                        }

                                    }

**else**{

                                        activeCellStatusList [counter1*12+4] = siblinFusionDoub1+10;

                                        activeCellStatusList [counter1*12+5] = 1;

                                        activeCellStatusList [counter1*12+6] = 1;

**if** (activeCellStatusList [counter1*12+9] != 0) activeCellStatusList [counter1*12+9]--;

                                    }

                                }

                            }

                        }

**else** **if** (eventType == 2){

**if** (activeCellStatusList [counter1*12+11] == 0){

**if** (siblinFusionCheck1 != 6){

**if** (expandDoublingDoubBDCount != 0){

                                        randInit = rand() % expandDoublingDoubBDCount/2 + 0;

                                        activeCellStatusList [counter1*12+4] = expandDoublingDoubBD [randInit*2];

                                        activeCellStatusList [counter1*12+5] = 2;

                                        activeCellStatusList [counter1*12+6] = 1;

                                        activeCellStatusList [counter1*12+7] = 1;

                                        activeCellStatusList [counter1*12+9] = 0;

                                        activeCellStatusList [counter1*12+8] = (**int**)simProcessDataMiddleHold [18];

                                    }

**else**{

                                        randInit = rand() % randBDRangeB + randBDRangeA;

                                        activeCellStatusList [counter1*12+4] = randInit;

                                        activeCellStatusList [counter1*12+5] = 2;

                                        activeCellStatusList [counter1*12+6] = 1;

                                        activeCellStatusList [counter1*12+7] = 1;

                                        activeCellStatusList [counter1*12+9] = 0;

                                        activeCellStatusList [counter1*12+8] = (**int**)simProcessDataMiddleHold [18];

                                    }

                                }

**else**{

**if** (siblinFusionDoub1 < randBDRangeA) siblinFusionDoub1 = randBDRangeA;

                                    activeCellStatusList [counter1*12+4] = siblinFusionDoub1+10;

                                    activeCellStatusList [counter1*12+5] = 2;

                                    activeCellStatusList [counter1*12+6] = 1;

                                    activeCellStatusList [counter1*12+7] = 1;

                                    activeCellStatusList [counter1*12+9] = 0;

                                    activeCellStatusList [counter1*12+8] = (**int**)simProcessDataMiddleHold [18];

                                }

                            }

**else**{

                                loopCheck = 0;

**for** (**int** counter2 = 0; counter2 < 100; counter2++){

**if** (siblinFusionCheck1 != 6){

**if** (expandDoublingDoubBDCount != 0){

                                            randInit = rand() % expandDoublingDoubBDCount/2 + 0;

**if** (expandDoublingDoubBD [randInit*2] > activeCellStatusList [counter1*12+11]-50 && expandDoublingDoubBD [randInit*2] < activeCellStatusList [counter1*12+11]+50){

                                                activeCellStatusList [counter1*12+4] = expandDoublingDoubBD [randInit*2];

                                                activeCellStatusList [counter1*12+5] = 2;

                                                activeCellStatusList [counter1*12+6] = 1;

                                                activeCellStatusList [counter1*12+7] = 1;

                                                activeCellStatusList [counter1*12+9] = 0;

                                                activeCellStatusList [counter1*12+8] = (**int**)simProcessDataMiddleHold [18];

                                                loopCheck = 1;

**break**;

                                            }

                                        }

                                    }

**else** **if** (siblinFusionCheck1 == 6){

**if** (expandDoublingDoubBDCount != 0){

                                            randInit = rand() % expandDoublingDoubBDCount/2 + 0;

**if** (expandDoublingDoubBD [randInit*2] > activeCellStatusList [counter1*12+11]-50 && expandDoublingDoubBD [randInit*2] < activeCellStatusList [counter1*12+11]+50 && siblinFusionDoub1+10 < expandDoublingDoubBD [randInit*2]){

                                                activeCellStatusList [counter1*12+4] = expandDoublingDoubBD [randInit*2];

                                                activeCellStatusList [counter1*12+5] = 2;

                                                activeCellStatusList [counter1*12+6] = 1;

                                                activeCellStatusList [counter1*12+7] = 1;

                                                activeCellStatusList [counter1*12+9] = 0;

                                                activeCellStatusList [counter1*12+8] = (**int**)simProcessDataMiddleHold [18];

                                                loopCheck = 1;

**break**;

                                            }

                                        }

                                    }

                                }

**if** (loopCheck == 0){

                                    lowextValue = 100000;

                                    highestValue = 0;

**for** (**int** counter2 = 0; counter2 < expandDoublingDoubBDCount/2; counter2++){

**if** (lowextValue > expandDoublingDoubBD [counter2*2]) lowextValue = expandDoublingDoubBD [counter2*2];

**if** (highestValue < expandDoublingDoubBD [counter2*2]) highestValue = expandDoublingDoubBD [counter2*2];

                                    }

**if** (siblinFusionDoub1 < randBDRangeA) siblinFusionDoub1 = randBDRangeA;

**if** (lowextValue != 100000 && highestValue != 0){

**if** ((highestValue+lowextValue)/(**double**)2 > siblinFusionDoub1+10){

                                            activeCellStatusList [counter1*12+4] = (**int**)(round((highestValue+lowextValue)/(**double**)2));

                                            activeCellStatusList [counter1*12+5] = 2;

                                            activeCellStatusList [counter1*12+6] = 1;

                                            activeCellStatusList [counter1*12+7] = 1;

                                            activeCellStatusList [counter1*12+9] = 0;

                                            activeCellStatusList [counter1*12+8] = (**int**)simProcessDataMiddleHold [18];

                                        }

**else**{

                                            activeCellStatusList [counter1*12+4] = siblinFusionDoub1+10;

                                            activeCellStatusList [counter1*12+5] = 2;

                                            activeCellStatusList [counter1*12+6] = 1;

                                            activeCellStatusList [counter1*12+7] = 1;

                                            activeCellStatusList [counter1*12+9] = 0;

                                            activeCellStatusList [counter1*12+8] = (**int**)simProcessDataMiddleHold [18];

                                        }

                                    }

**else**{

                                        activeCellStatusList [counter1*12+4] = siblinFusionDoub1+10;

                                        activeCellStatusList [counter1*12+5] = 2;

                                        activeCellStatusList [counter1*12+6] = 1;

                                        activeCellStatusList [counter1*12+7] = 1;

                                        activeCellStatusList [counter1*12+9] = 0;

                                        activeCellStatusList [counter1*12+8] = (**int**)simProcessDataMiddleHold [18];

                                    }

                                }

                            }

                        }

**else** **if** (eventType == 5){

**if** (expandBDCDCount == 0){

                                randInit = rand() % randCDRangeB + randCDRangeA;

**if** (siblinFusionCheck1 != 6){

                                    activeCellStatusList [counter1*12+4] = randInit;

                                    activeCellStatusList [counter1*12+5] = 3;

                                    activeCellStatusList [counter1*12+6] = 1;

                                    activeCellStatusList [counter1*12+9] = 0;

                                }

**else**{

                                    activeCellStatusList [counter1*12+4] = siblinFusionDoub1+10;

                                    activeCellStatusList [counter1*12+5] = 3;

                                    activeCellStatusList [counter1*12+6] = 1;

                                    activeCellStatusList [counter1*12+9] = 0;

                                }

                            }

**else**{

**if** (siblinFusionCheck1 != 6){

**if** (expandBDCDCount != 0){

                                        randInit = rand() % expandBDCDCount/2 + 0;

                                        activeCellStatusList [counter1*12+4] = expandBDCD [randInit*2];

                                        activeCellStatusList [counter1*12+5] = 3;

                                        activeCellStatusList [counter1*12+6] = 1;

                                        activeCellStatusList [counter1*12+9] = 0;

                                    }

**else**{

                                        activeCellStatusList [counter1*12+4] = siblinFusionDoub1+10;

                                        activeCellStatusList [counter1*12+5] = 3;

                                        activeCellStatusList [counter1*12+6] = 1;

                                        activeCellStatusList [counter1*12+9] = 0;

                                    }

                                }

**else**{

**if** (expandBDCDCount != 0){

                                        randInit = rand() % expandBDCDCount/2 + 0;

**if**(expandBDCD [randInit*2] > siblinFusionDoub1){

                                            activeCellStatusList [counter1*12+4] = expandBDCD [randInit*2]+10;

                                            activeCellStatusList [counter1*12+5] = 3;

                                            activeCellStatusList [counter1*12+6] = 1;

                                            activeCellStatusList [counter1*12+9] = 0;

                                        }

**else**{

                                            activeCellStatusList [counter1*12+4] = siblinFusionDoub1+10;

                                            activeCellStatusList [counter1*12+5] = 3;

                                            activeCellStatusList [counter1*12+6] = 1;

                                            activeCellStatusList [counter1*12+9] = 0;

                                        }

                                    }

**else**{

                                        activeCellStatusList [counter1*12+4] = siblinFusionDoub1+10;

                                        activeCellStatusList [counter1*12+5] = 3;

                                        activeCellStatusList [counter1*12+6] = 1;

                                        activeCellStatusList [counter1*12+9] = 0;

                                    }

                                }

                            }

                        }

**else** **if** (eventType == 6){

**if** (expandBDCFCount == 0){

                                randInit = rand() % randCDRangeB + randCDRangeA;

**if** (siblinFusionCheck1 != 6){

                                    activeCellStatusList [counter1*12+4] = randInit;

                                    activeCellStatusList [counter1*12+5] = 6;

                                    activeCellStatusList [counter1*12+6] = 1;

                                    activeCellStatusList [counter1*12+9] = 0;

                                }

**else**{

**if** (siblinFusionDoub1 < randBDRangeA) siblinFusionDoub1 = randBDRangeA;

                                    activeCellStatusList [siblingCellNo1*12+4] = siblinFusionDoub1+10;

                                    activeCellStatusList [counter1*12+4] = siblinFusionDoub1;

                                    activeCellStatusList [counter1*12+5] = 6;

                                    activeCellStatusList [counter1*12+6] = 1;

                                    activeCellStatusList [counter1*12+9] = 0;

                                }

                            }

**else**{

**if** (siblinFusionCheck1 == 0){

**if** (expandBDCFCount != 0){

                                        randInit = rand() % expandBDCFCount/2 + 0;

                                        activeCellStatusList [counter1*12+4] = expandBDCF [randInit*2];

                                        activeCellStatusList [counter1*12+5] = 6;

                                        activeCellStatusList [counter1*12+6] = 1;

                                        activeCellStatusList [counter1*12+9] = 0;

                                    }

**else** {

                                        activeCellStatusList [counter1*12+4] = siblinFusionDoub1+10;

                                        activeCellStatusList [counter1*12+5] = 6;

                                        activeCellStatusList [counter1*12+6] = 1;

                                        activeCellStatusList [counter1*12+9] = 0;

                                    }

                                }

**else**{

**if** (expandBDCDCount != 0){

                                        randInit = rand() % expandBDCDCount/2 + 0;

**if**(expandBDCD [randInit*2] > siblinFusionDoub1){

                                            activeCellStatusList [counter1*12+4] = expandBDCD [randInit*2]+10;

                                            activeCellStatusList [counter1*12+5] = 3;

                                            activeCellStatusList [counter1*12+6] = 1;

                                            activeCellStatusList [counter1*12+9] = 0;

                                        }

**else**{

                                            activeCellStatusList [counter1*12+4] = siblinFusionDoub1+10;

                                            activeCellStatusList [counter1*12+5] = 3;

                                            activeCellStatusList [counter1*12+6] = 1;

                                            activeCellStatusList [counter1*12+9] = 0;

                                        }

                                    }

**else**{

                                        activeCellStatusList [siblingCellNo1*12+4] = siblinFusionDoub1+10;

                                        activeCellStatusList [counter1*12+4] = siblinFusionDoub1;

                                        activeCellStatusList [counter1*12+5] = 6;

                                        activeCellStatusList [counter1*12+6] = 1;

                                        activeCellStatusList [counter1*12+9] = 0;

                                    }

                                }

                            }

                        }

**else** **if** (eventType == 10){

                            dataTempDouble = ((expandNonCDCount/(**double**)2)/(**double**)simProcessDataMiddleHold [24])*100;

                            randInit = rand() % 100 + 0;

**if** (randInit < dataTempDouble && expandNonCDCount != 0){

                                randInit = rand() % expandNonCDCount/2 + 0;

                                activeCellStatusList [counter1*12+4] = expandNonCD [randInit*2];

                                activeCellStatusList [counter1*12+5] = 30;

                                activeCellStatusList [counter1*12+6] = 1;

                            }

**else**{

                                randInit = rand() % (**int**)(round(simProcessDataMiddleHold [25]*(endVariationtHold/(**double**)100))) + 0;

**if** (randInit%2 == 0) activeCellStatusList [counter1*12+4] = (**int**)simProcessDataMiddleHold [25]+randInit;

**else** activeCellStatusList [counter1*12+4] = (**int**)simProcessDataMiddleHold [25]-randInit;

                                activeCellStatusList [counter1*12+5] = 5;

                                activeCellStatusList [counter1*12+6] = 1;

                            }

                        }

                    }

**else** **if** (activeCellStatusList [counter1*12+5] == 2){

                        siblinFusionCheck1 = 0;

                        siblinFusionCheck2 = 0;

                        siblinFusionDoub1 = 0;

                        siblinFusionDoub2 = 0;

                        siblingCellNo1 = 0;

                        siblingCellNo2 = 0;

**for** (**int** counter2 = 0; counter2 < activeCellStatusListCount/12; counter2++){

**if** (activeCellStatusList [counter2*12+6] == 1 && (activeCellStatusList [counter2*12+1] == activeCellStatusList [counter1*12] || activeCellStatusList [counter2*12+2] == activeCellStatusList [counter1*12]) && activeCellStatusList [counter2*12+3] == activeCellStatusList [counter1*12+3]){

**if** (siblinFusionCheck1 == 0 && activeCellStatusList [counter2*12+5] != 0){

                                    siblinFusionCheck1 = activeCellStatusList [counter2*12+5];

                                    siblinFusionDoub1 = activeCellStatusList [counter2*12+4];

                                    siblingCellNo1 = counter2;

                                }
[truncated: 267,062 more chars]
