# Supplementary material for: Empirical single-cell tracking and cell-fate simulation reveal dual roles of p53 in tumor suppression
Source: eLife. 2022 Sep 20;11:e72498. doi: 10.7554/eLife.72498 (PMC9560164; doi:10.7554/eLife.72498)
Supplement: Figure 7—source code 5. [file elife-72498-fig7-code5.docx]

//

//  CreateNewCellNo.m

//  Lineage_Analysis

//

//  Created by Masahiko Sato

//

/*

 Cell number assignment

 The following code is written in Objective-C/C++ using Xcode.

 */

-(**int**)cellNumberAddition:(**int**)position{

    string cellNumberExtract = to_string(activeCellStatusList [position*12]);

**if** (activeCellStatusList [position*12] < 0){

**if** ((**int**)cellNumberExtract.length() == 9) cellNumberExtract = "-0"+cellNumberExtract.substr(1);

    }

**else** **if** ((**int**)cellNumberExtract.length() == 8) cellNumberExtract = "0"+cellNumberExtract;

**int** cellNumberTempInt = activeCellStatusList [position*12];

**int** numberLength = (**int**)cellNumberExtract.length();

**int** zeroPoint = -1;

**int** checkFlag = 0;

**int** newCellNumber = 0;

**int** degitNumber = 0;

**int** cellExtensionEntry = 0;

    string degitExtract;

    string newExtensionEntryNo;

**if** (cellNumberTempInt == 0) newCellNumber = -100000000;

**else**{

**for** (**int** counter3 = 0; counter3 < numberLength; counter3++){

            degitExtract = cellNumberExtract.substr((**unsigned** **long**)counter3, 1);

**if** (checkFlag == 0 && degitExtract == "0"){

                zeroPoint = counter3;

                checkFlag = 1;

            }

**else** **if** (checkFlag == 1 && degitExtract != "0"){

                zeroPoint = -1;

                checkFlag = 0;

            }

        }

**if** (zeroPoint != -1){

            degitNumber = numberLength-zeroPoint;

**if** (degitNumber == 8) newCellNumber = cellNumberTempInt+10000000;

**else** **if** (degitNumber == 7) newCellNumber = cellNumberTempInt+1000000;

**else** **if** (degitNumber == 6) newCellNumber = cellNumberTempInt+100000;

**else** **if** (degitNumber == 5) newCellNumber = cellNumberTempInt+10000;

**else** **if** (degitNumber == 4) newCellNumber = cellNumberTempInt+1000;

**else** **if** (degitNumber == 3) newCellNumber = cellNumberTempInt+100;

**else** **if** (degitNumber == 2) newCellNumber = cellNumberTempInt+10;

**else** **if** (degitNumber == 1) newCellNumber = cellNumberTempInt+1;

        }

**else**{

            cellExtensionEntry = 0;

**if** (cellNumberTempInt > 0){

**if** (cellNumberExtract.substr(0, 1) == "0"){

**for** (**int** counter3 = 0; counter3 < cellNoLingNoListCount/2; counter3++){

**if** (cellNoLingNoList [counter3*2] == activeCellStatusList [position*12+3]){

**if** ((to_string(cellNoLingNoList [counter3*2+1])).substr(0, 1) == "3"){

**if** (atoi((to_string(cellNoLingNoList [counter3*2+1])).substr(1, 5).c_str()) > cellExtensionEntry){

                                    cellExtensionEntry = atoi((to_string(cellNoLingNoList [counter3*2+1])).substr(1, 5).c_str());

                                }

                            }

                        }

                    }

                    cellExtensionEntry++;

                    newExtensionEntryNo = to_string(cellExtensionEntry);

**if** (newExtensionEntryNo.length() == 1) newExtensionEntryNo = "30000"+newExtensionEntryNo+"700";

**else** **if** (newExtensionEntryNo.length() == 2) newExtensionEntryNo = "3000"+newExtensionEntryNo+"700";

**else** **if** (newExtensionEntryNo.length() == 3) newExtensionEntryNo = "300"+newExtensionEntryNo+"700";

**else** **if** (newExtensionEntryNo.length() == 4) newExtensionEntryNo = "30"+newExtensionEntryNo+"700";

**else** **if** (newExtensionEntryNo.length() == 5) newExtensionEntryNo = "3"+newExtensionEntryNo+"700";

                    newCellNumber = atoi(newExtensionEntryNo.c_str());

                }

**if** (cellNumberExtract.substr(0, 1) == "1" || cellNumberExtract.substr(0, 1) == "2"){

**for** (**int** counter3 = 0; counter3 < cellNoLingNoListCount/2; counter3++){

**if** (cellNoLingNoList [counter3*2] == activeCellStatusList [position*12+3]){

**if** ((to_string(cellNoLingNoList [counter3*2+1])).substr(0, 1) == "4"){

**if** (atoi((to_string(cellNoLingNoList [counter3*2+1])).substr(1, 5).c_str()) > cellExtensionEntry){

                                    cellExtensionEntry = atoi((to_string(cellNoLingNoList [counter3*2+1])).substr(1, 5).c_str());

                                }

                            }

                        }

                    }

                    cellExtensionEntry++;

                    newExtensionEntryNo = to_string(cellExtensionEntry);

**if** (newExtensionEntryNo.length() == 1) newExtensionEntryNo = "40000"+newExtensionEntryNo+"700";

**else** **if** (newExtensionEntryNo.length() == 2) newExtensionEntryNo = "4000"+newExtensionEntryNo+"700";

**else** **if** (newExtensionEntryNo.length() == 3) newExtensionEntryNo = "400"+newExtensionEntryNo+"700";

**else** **if** (newExtensionEntryNo.length() == 4) newExtensionEntryNo = "40"+newExtensionEntryNo+"700";

**else** **if** (newExtensionEntryNo.length() == 5) newExtensionEntryNo = "4"+newExtensionEntryNo+"700";

                    newCellNumber = atoi(newExtensionEntryNo.c_str());

                }

**if** (cellNumberExtract.substr(0, 1) == "3"){

**for** (**int** counter3 = 0; counter3 < cellNoLingNoListCount/2; counter3++){

**if** (cellNoLingNoList [counter3*2] == activeCellStatusList [position*12+3]){

**if** ((to_string(cellNoLingNoList [counter3*2+1])).substr(0, 1) == "5"){

**if** (atoi((to_string(cellNoLingNoList [counter3*2+1])).substr(1, 5).c_str()) > cellExtensionEntry){

                                    cellExtensionEntry = atoi((to_string(cellNoLingNoList [counter3*2+1])).substr(1, 5).c_str());

                                }

                            }

                        }

                    }

                    cellExtensionEntry++;

                    newExtensionEntryNo = to_string(cellExtensionEntry);

**if** (newExtensionEntryNo.length() == 1) newExtensionEntryNo = "50000"+newExtensionEntryNo+"700";

**else** **if** (newExtensionEntryNo.length() == 2) newExtensionEntryNo = "5000"+newExtensionEntryNo+"700";

**else** **if** (newExtensionEntryNo.length() == 3) newExtensionEntryNo = "500"+newExtensionEntryNo+"700";

**else** **if** (newExtensionEntryNo.length() == 4) newExtensionEntryNo = "50"+newExtensionEntryNo+"700";

**else** **if** (newExtensionEntryNo.length() == 5) newExtensionEntryNo = "5"+newExtensionEntryNo+"700";

                    newCellNumber = atoi(newExtensionEntryNo.c_str());

                }

**if** (cellNumberExtract.substr(0, 1) == "4"){

**for** (**int** counter3 = 0; counter3 < cellNoLingNoListCount/2; counter3++){

**if** (cellNoLingNoList [counter3*2] == activeCellStatusList [position*12+3]){

**if** ((to_string(cellNoLingNoList [counter3*2+1])).substr(0, 1) == "6"){

**if** (atoi((to_string(cellNoLingNoList [counter3*2+1])).substr(1, 5).c_str()) > cellExtensionEntry){

                                    cellExtensionEntry = atoi((to_string(cellNoLingNoList [counter3*2+1])).substr(1, 5).c_str());

                                }

                            }

                        }

                    }

                    cellExtensionEntry++;

                    newExtensionEntryNo = to_string(cellExtensionEntry);

**if** (newExtensionEntryNo.length() == 1) newExtensionEntryNo = "60000"+newExtensionEntryNo+"700";

**else** **if** (newExtensionEntryNo.length() == 2) newExtensionEntryNo = "6000"+newExtensionEntryNo+"700";

**else** **if** (newExtensionEntryNo.length() == 3) newExtensionEntryNo = "600"+newExtensionEntryNo+"700";

**else** **if** (newExtensionEntryNo.length() == 4) newExtensionEntryNo = "60"+newExtensionEntryNo+"700";

**else** **if** (newExtensionEntryNo.length() == 5) newExtensionEntryNo = "6"+newExtensionEntryNo+"700";

                    newCellNumber = atoi(newExtensionEntryNo.c_str());

                }

            }

**else**{

**if** (cellNumberExtract.substr(1, 1) == "0"){

**for** (**int** counter3 = 0; counter3 < cellNoLingNoListCount/2; counter3++){

**if** (cellNoLingNoList [counter3*2] == activeCellStatusList [position*12+3]){

**if** ((to_string(cellNoLingNoList [counter3*2+1])).substr(1, 1) == "3"){

**if** (atoi((to_string(cellNoLingNoList [counter3*2+1])).substr(2, 5).c_str()) > cellExtensionEntry){

                                    cellExtensionEntry = atoi((to_string(cellNoLingNoList [counter3*2+1])).substr(2, 5).c_str());

                                }

                            }

                        }

                    }

                    cellExtensionEntry++;

                    newExtensionEntryNo = to_string(cellExtensionEntry);

**if** (newExtensionEntryNo.length() == 1) newExtensionEntryNo = "-30000"+newExtensionEntryNo+"700";

**else** **if** (newExtensionEntryNo.length() == 2) newExtensionEntryNo = "-3000"+newExtensionEntryNo+"700";

**else** **if** (newExtensionEntryNo.length() == 3) newExtensionEntryNo = "-300"+newExtensionEntryNo+"700";

**else** **if** (newExtensionEntryNo.length() == 4) newExtensionEntryNo = "-30"+newExtensionEntryNo+"700";

**else** **if** (newExtensionEntryNo.length() == 5) newExtensionEntryNo = "-3"+newExtensionEntryNo+"700";

                    newCellNumber = atoi(newExtensionEntryNo.c_str());

                }

**if** (cellNumberExtract.substr(1, 1) == "1" || cellNumberExtract.substr(1, 1) == "2"){

**for** (**int** counter3 = 0; counter3 < cellNoLingNoListCount/2; counter3++){

**if** (cellNoLingNoList [counter3*2] == activeCellStatusList [position*12+3]){

**if** ((to_string(cellNoLingNoList [counter3*2+1])).substr(1, 1) == "4"){

**if** (atoi((to_string(cellNoLingNoList [counter3*2+1])).substr(2, 5).c_str()) > cellExtensionEntry){

                                    cellExtensionEntry = atoi((to_string(cellNoLingNoList [counter3*2+1])).substr(2, 5).c_str());

                                }

                            }

                        }

                    }

                    cellExtensionEntry++;

                    newExtensionEntryNo = to_string(cellExtensionEntry);

**if** (newExtensionEntryNo.length() == 1) newExtensionEntryNo = "-40000"+newExtensionEntryNo+"700";

**else** **if** (newExtensionEntryNo.length() == 2) newExtensionEntryNo = "-4000"+newExtensionEntryNo+"700";

**else** **if** (newExtensionEntryNo.length() == 3) newExtensionEntryNo = "-400"+newExtensionEntryNo+"700";

**else** **if** (newExtensionEntryNo.length() == 4) newExtensionEntryNo = "-40"+newExtensionEntryNo+"700";

**else** **if** (newExtensionEntryNo.length() == 5) newExtensionEntryNo = "-4"+newExtensionEntryNo+"700";

                    newCellNumber = atoi(newExtensionEntryNo.c_str());

                }

**if** (cellNumberExtract.substr(1, 1) == "3"){

**for** (**int** counter3 = 0; counter3 < cellNoLingNoListCount/2; counter3++){

**if** (cellNoLingNoList [counter3*2] == activeCellStatusList [position*12+3]){

**if** ((to_string(cellNoLingNoList [counter3*2+1])).substr(1, 1) == "5"){

**if** (atoi((to_string(cellNoLingNoList [counter3*2+1])).substr(2, 5).c_str()) > cellExtensionEntry){

                                    cellExtensionEntry = atoi((to_string(cellNoLingNoList [counter3*2+1])).substr(2, 5).c_str());

                                }

                            }

                        }

                    }

                    cellExtensionEntry++;

                    newExtensionEntryNo = to_string(cellExtensionEntry);

**if** (newExtensionEntryNo.length() == 1) newExtensionEntryNo = "-50000"+newExtensionEntryNo+"700";

**else** **if** (newExtensionEntryNo.length() == 2) newExtensionEntryNo = "-5000"+newExtensionEntryNo+"700";

**else** **if** (newExtensionEntryNo.length() == 3) newExtensionEntryNo = "-500"+newExtensionEntryNo+"700";

**else** **if** (newExtensionEntryNo.length() == 4) newExtensionEntryNo = "-50"+newExtensionEntryNo+"700";

**else** **if** (newExtensionEntryNo.length() == 5) newExtensionEntryNo = "-5"+newExtensionEntryNo+"700";

                    newCellNumber = atoi(newExtensionEntryNo.c_str());

                }

**if** (cellNumberExtract.substr(1, 1) == "4"){

**for** (**int** counter3 = 0; counter3 < cellNoLingNoListCount/2; counter3++){

**if** (cellNoLingNoList [counter3*2] == activeCellStatusList [position*12+3]){

**if** ((to_string(cellNoLingNoList [counter3*2+1])).substr(1, 1) == "6"){

**if** (atoi((to_string(cellNoLingNoList [counter3*2+1])).substr(2, 5).c_str()) > cellExtensionEntry){

                                    cellExtensionEntry = atoi((to_string(cellNoLingNoList [counter3*2+1])).substr(2, 5).c_str());

                                }

                            }

                        }

                    }

                    cellExtensionEntry++;

                    newExtensionEntryNo = to_string(cellExtensionEntry);

**if** (newExtensionEntryNo.length() == 1) newExtensionEntryNo = "-60000"+newExtensionEntryNo+"700";

**else** **if** (newExtensionEntryNo.length() == 2) newExtensionEntryNo = "-6000"+newExtensionEntryNo+"700";

**else** **if** (newExtensionEntryNo.length() == 3) newExtensionEntryNo = "-600"+newExtensionEntryNo+"700";

**else** **if** (newExtensionEntryNo.length() == 4) newExtensionEntryNo = "-60"+newExtensionEntryNo+"700";

**else** **if** (newExtensionEntryNo.length() == 5) newExtensionEntryNo = "-6"+newExtensionEntryNo+"700";

                    newCellNumber = atoi(newExtensionEntryNo.c_str());

                }

            }

        }

    }

**return** newCellNumber;

}

-(**int**)cellNumberSubtraction:(**int**)position{

    string cellNumberExtract = to_string(activeCellStatusList [position*12]);

**if** (activeCellStatusList [position*12] < 0){

**if** ((**int**)cellNumberExtract.length() == 9) cellNumberExtract = "-0"+cellNumberExtract.substr(1);

    }

**else** **if** ((**int**)cellNumberExtract.length() == 8) cellNumberExtract = "0"+cellNumberExtract;

**int** cellNumberTempInt = activeCellStatusList [position*12];

**int** numberLength = (**int**)cellNumberExtract.length();

**int** zeroPoint = -1;

**int** checkFlag = 0;

**int** newCellNumber = 0;

**int** degitNumber = 0;

**int** cellExtensionEntry = 0;

    string degitExtract;

    string newExtensionEntryNo;

**if** (cellNumberTempInt == 0) newCellNumber = 100000000;

**else**{

**for** (**int** counter3 = 0; counter3 < numberLength; counter3++){

            degitExtract = cellNumberExtract.substr((**unsigned** **long**)counter3, 1);

**if** (checkFlag == 0 && degitExtract == "0"){

                zeroPoint = counter3;

                checkFlag = 1;

            }

**else** **if** (checkFlag == 1 && degitExtract != "0"){

                zeroPoint = -1;

                checkFlag = 0;

            }

        }

**if** (zeroPoint != -1){

            degitNumber = numberLength-zeroPoint;

**if** (degitNumber == 8) newCellNumber = cellNumberTempInt-10000000;

**else** **if** (degitNumber == 7) newCellNumber = cellNumberTempInt-1000000;

**else** **if** (degitNumber == 6) newCellNumber = cellNumberTempInt-100000;

**else** **if** (degitNumber == 5) newCellNumber = cellNumberTempInt-10000;

**else** **if** (degitNumber == 4) newCellNumber = cellNumberTempInt-1000;

**else** **if** (degitNumber == 3) newCellNumber = cellNumberTempInt-100;

**else** **if** (degitNumber == 2) newCellNumber = cellNumberTempInt-10;

**else** **if** (degitNumber == 1) newCellNumber = cellNumberTempInt-1;

        }

**else**{

            cellExtensionEntry = 0;

**if** (cellNumberTempInt > 0){

**if** (cellNumberExtract.substr(0, 1) == "0"){

**for** (**int** counter3 = 0; counter3 < cellNoLingNoListCount/2; counter3++){

**if** (cellNoLingNoList [counter3*2] == activeCellStatusList [position*12+3]){

**if** ((to_string(cellNoLingNoList [counter3*2+1])).substr(0, 1) == "3"){

**if** (atoi((to_string(cellNoLingNoList [counter3*2+1])).substr(1, 5).c_str()) > cellExtensionEntry){

                                    cellExtensionEntry = atoi((to_string(cellNoLingNoList [counter3*2+1])).substr(1, 5).c_str());

                                }

                            }

                        }

                    }

                    cellExtensionEntry++;

                    newExtensionEntryNo = to_string(cellExtensionEntry);

**if** (newExtensionEntryNo.length() == 1) newExtensionEntryNo = "30000"+newExtensionEntryNo+"300";

**else** **if** (newExtensionEntryNo.length() == 2) newExtensionEntryNo = "3000"+newExtensionEntryNo+"300";

**else** **if** (newExtensionEntryNo.length() == 3) newExtensionEntryNo = "300"+newExtensionEntryNo+"300";

**else** **if** (newExtensionEntryNo.length() == 4) newExtensionEntryNo = "30"+newExtensionEntryNo+"300";

**else** **if** (newExtensionEntryNo.length() == 5) newExtensionEntryNo = "3"+newExtensionEntryNo+"300";

                    newCellNumber = atoi(newExtensionEntryNo.c_str());

                }

**if** (cellNumberExtract.substr(0, 1) == "1" || cellNumberExtract.substr(0, 1) == "2"){

**for** (**int** counter3 = 0; counter3 < cellNoLingNoListCount/2; counter3++){

**if** (cellNoLingNoList [counter3*2] == activeCellStatusList [position*12+3]){

**if** ((to_string(cellNoLingNoList [counter3*2+1])).substr(0, 1) == "4"){

**if** (atoi((to_string(cellNoLingNoList [counter3*2+1])).substr(1, 5).c_str()) > cellExtensionEntry){

                                    cellExtensionEntry = atoi((to_string(cellNoLingNoList [counter3*2+1])).substr(1, 5).c_str());

                                }

                            }

                        }

                    }

                    cellExtensionEntry++;

                    newExtensionEntryNo = to_string(cellExtensionEntry);

**if** (newExtensionEntryNo.length() == 1) newExtensionEntryNo = "40000"+newExtensionEntryNo+"300";

**else** **if** (newExtensionEntryNo.length() == 2) newExtensionEntryNo = "4000"+newExtensionEntryNo+"300";

**else** **if** (newExtensionEntryNo.length() == 3) newExtensionEntryNo = "400"+newExtensionEntryNo+"300";

**else** **if** (newExtensionEntryNo.length() == 4) newExtensionEntryNo = "40"+newExtensionEntryNo+"300";

**else** **if** (newExtensionEntryNo.length() == 5) newExtensionEntryNo = "4"+newExtensionEntryNo+"300";

                    newCellNumber = atoi(newExtensionEntryNo.c_str());

                }

**if** (cellNumberExtract.substr(0, 1) == "3"){

**for** (**int** counter3 = 0; counter3 < cellNoLingNoListCount/2; counter3++){

**if** (cellNoLingNoList [counter3*2] == activeCellStatusList [position*12+3]){

**if** ((to_string(cellNoLingNoList [counter3*2+1])).substr(0, 1) == "5"){

**if** (atoi((to_string(cellNoLingNoList [counter3*2+1])).substr(1, 5).c_str()) > cellExtensionEntry){

                                    cellExtensionEntry = atoi((to_string(cellNoLingNoList [counter3*2+1])).substr(1, 5).c_str());

                                }

                            }

                        }

                    }

                    cellExtensionEntry++;

                    newExtensionEntryNo = to_string(cellExtensionEntry);

**if** (newExtensionEntryNo.length() == 1) newExtensionEntryNo = "50000"+newExtensionEntryNo+"300";

**else** **if** (newExtensionEntryNo.length() == 2) newExtensionEntryNo = "5000"+newExtensionEntryNo+"300";

**else** **if** (newExtensionEntryNo.length() == 3) newExtensionEntryNo = "500"+newExtensionEntryNo+"300";

**else** **if** (newExtensionEntryNo.length() == 4) newExtensionEntryNo = "50"+newExtensionEntryNo+"300";

**else** **if** (newExtensionEntryNo.length() == 5) newExtensionEntryNo = "5"+newExtensionEntryNo+"300";

                    newCellNumber = atoi(newExtensionEntryNo.c_str());

                }

**if** (cellNumberExtract.substr(0, 1) == "4"){

**for** (**int** counter3 = 0; counter3 < cellNoLingNoListCount/2; counter3++){

**if** (cellNoLingNoList [counter3*2] == activeCellStatusList [position*12+3]){

**if** ((to_string(cellNoLingNoList [counter3*2+1])).substr(0, 1) == "6"){

**if** (atoi((to_string(cellNoLingNoList [counter3*2+1])).substr(1, 5).c_str()) > cellExtensionEntry){

                                    cellExtensionEntry = atoi((to_string(cellNoLingNoList [counter3*2+1])).substr(1, 5).c_str());

                                }

                            }

                        }

                    }

                    cellExtensionEntry++;

                    newExtensionEntryNo = to_string(cellExtensionEntry);

**if** (newExtensionEntryNo.length() == 1) newExtensionEntryNo = "60000"+newExtensionEntryNo+"300";

**else** **if** (newExtensionEntryNo.length() == 2) newExtensionEntryNo = "6000"+newExtensionEntryNo+"300";

**else** **if** (newExtensionEntryNo.length() == 3) newExtensionEntryNo = "600"+newExtensionEntryNo+"300";

**else** **if** (newExtensionEntryNo.length() == 4) newExtensionEntryNo = "60"+newExtensionEntryNo+"300";

**else** **if** (newExtensionEntryNo.length() == 5) newExtensionEntryNo = "6"+newExtensionEntryNo+"300";

                    newCellNumber = atoi(newExtensionEntryNo.c_str());

                }

            }

**else**{

**if** (cellNumberExtract.substr(1, 1) == "0"){

**for** (**int** counter3 = 0; counter3 < cellNoLingNoListCount/2; counter3++){

**if** (cellNoLingNoList [counter3*2] == activeCellStatusList [position*12+3]){

**if** ((to_string(cellNoLingNoList [counter3*2+1])).substr(1, 1) == "3"){

**if** (atoi((to_string(cellNoLingNoList [counter3*2+1])).substr(2, 5).c_str()) > cellExtensionEntry){

                                    cellExtensionEntry = atoi((to_string(cellNoLingNoList [counter3*2+1])).substr(2, 5).c_str());

                                }

                            }

                        }

                    }

                    cellExtensionEntry++;

                    newExtensionEntryNo = to_string(cellExtensionEntry);

**if** (newExtensionEntryNo.length() == 1) newExtensionEntryNo = "-30000"+newExtensionEntryNo+"300";

**else** **if** (newExtensionEntryNo.length() == 2) newExtensionEntryNo = "-3000"+newExtensionEntryNo+"300";

**else** **if** (newExtensionEntryNo.length() == 3) newExtensionEntryNo = "-300"+newExtensionEntryNo+"300";

**else** **if** (newExtensionEntryNo.length() == 4) newExtensionEntryNo = "-30"+newExtensionEntryNo+"300";

**else** **if** (newExtensionEntryNo.length() == 5) newExtensionEntryNo = "-3"+newExtensionEntryNo+"300";

                    newCellNumber = atoi(newExtensionEntryNo.c_str());

                }

**if** (cellNumberExtract.substr(1, 1) == "1" || cellNumberExtract.substr(1, 1) == "2"){

**for** (**int** counter3 = 0; counter3 < cellNoLingNoListCount/2; counter3++){

**if** (cellNoLingNoList [counter3*2] == activeCellStatusList [position*12+3]){

**if** ((to_string(cellNoLingNoList [counter3*2+1])).substr(1, 1) == "4"){

**if** (atoi((to_string(cellNoLingNoList [counter3*2+1])).substr(2, 5).c_str()) > cellExtensionEntry){

                                    cellExtensionEntry = atoi((to_string(cellNoLingNoList [counter3*2+1])).substr(2, 5).c_str());

                                }

                            }

                        }

                    }

                    cellExtensionEntry++;

                    newExtensionEntryNo = to_string(cellExtensionEntry);

**if** (newExtensionEntryNo.length() == 1) newExtensionEntryNo = "-40000"+newExtensionEntryNo+"300";

**else** **if** (newExtensionEntryNo.length() == 2) newExtensionEntryNo = "-4000"+newExtensionEntryNo+"300";

**else** **if** (newExtensionEntryNo.length() == 3) newExtensionEntryNo = "-400"+newExtensionEntryNo+"300";

**else** **if** (newExtensionEntryNo.length() == 4) newExtensionEntryNo = "-40"+newExtensionEntryNo+"300";

**else** **if** (newExtensionEntryNo.length() == 5) newExtensionEntryNo = "-4"+newExtensionEntryNo+"300";

                    newCellNumber = atoi(newExtensionEntryNo.c_str());

                }

**if** (cellNumberExtract.substr(1, 1) == "3"){

**for** (**int** counter3 = 0; counter3 < cellNoLingNoListCount/2; counter3++){

**if** (cellNoLingNoList [counter3*2] == activeCellStatusList [position*12+3]){

**if** ((to_string(cellNoLingNoList [counter3*2+1])).substr(1, 1) == "5"){

**if** (atoi((to_string(cellNoLingNoList [counter3*2+1])).substr(2, 5).c_str()) > cellExtensionEntry){

                                    cellExtensionEntry = atoi((to_string(cellNoLingNoList [counter3*2+1])).substr(2, 5).c_str());

                                }

                            }

                        }

                    }

                    cellExtensionEntry++;

                    newExtensionEntryNo = to_string(cellExtensionEntry);

**if** (newExtensionEntryNo.length() == 1) newExtensionEntryNo = "-50000"+newExtensionEntryNo+"300";

**else** **if** (newExtensionEntryNo.length() == 2) newExtensionEntryNo = "-5000"+newExtensionEntryNo+"300";

**else** **if** (newExtensionEntryNo.length() == 3) newExtensionEntryNo = "-500"+newExtensionEntryNo+"300";

**else** **if** (newExtensionEntryNo.length() == 4) newExtensionEntryNo = "-50"+newExtensionEntryNo+"300";

**else** **if** (newExtensionEntryNo.length() == 5) newExtensionEntryNo = "-5"+newExtensionEntryNo+"300";

                    newCellNumber = atoi(newExtensionEntryNo.c_str());

                }

**if** (cellNumberExtract.substr(1, 1) == "4"){

**for** (**int** counter3 = 0; counter3 < cellNoLingNoListCount/2; counter3++){

**if** (cellNoLingNoList [counter3*2] == activeCellStatusList [position*12+3]){

**if** ((to_string(cellNoLingNoList [counter3*2+1])).substr(1, 1) == "6"){

**if** (atoi((to_string(cellNoLingNoList [counter3*2+1])).substr(2, 5).c_str()) > cellExtensionEntry){

                                    cellExtensionEntry = atoi((to_string(cellNoLingNoList [counter3*2+1])).substr(2, 5).c_str());

                                }

                            }

                        }

                    }

                    cellExtensionEntry++;

                    newExtensionEntryNo = to_string(cellExtensionEntry);

**if** (newExtensionEntryNo.length() == 1) newExtensionEntryNo = "-60000"+newExtensionEntryNo+"300";

**else** **if** (newExtensionEntryNo.length() == 2) newExtensionEntryNo = "-6000"+newExtensionEntryNo+"300";

**else** **if** (newExtensionEntryNo.length() == 3) newExtensionEntryNo = "-600"+newExtensionEntryNo+"300";

**else** **if** (newExtensionEntryNo.length() == 4) newExtensionEntryNo = "-60"+newExtensionEntryNo+"300";

**else** **if** (newExtensionEntryNo.length() == 5) newExtensionEntryNo = "-6"+newExtensionEntryNo+"300";

                    newCellNumber = atoi(newExtensionEntryNo.c_str());

                }

            }

        }

    }

**return** newCellNumber;

}

-(**int**)cellNumberAdditionSecond:(**int**)position{

    string cellNumberExtract = to_string(activeCellStatusList [position*12]);

**if** (activeCellStatusList [position*12] < 0){

**if** ((**int**)cellNumberExtract.length() == 9) cellNumberExtract = "-0"+cellNumberExtract.substr(1);

    }

**else** **if** ((**int**)cellNumberExtract.length() == 8) cellNumberExtract = "0"+cellNumberExtract;

**int** cellNumberTempInt = activeCellStatusList [position*12];

**int** numberLength = (**int**)cellNumberExtract.length();

**int** zeroPoint = -1;

**int** checkFlag = 0;

**int** newCellNumber = 0;

**int** degitNumber = 0;

**int** cellExtensionEntry = 0;

    string degitExtract;

    string newExtensionEntryNo;

**if** (cellNumberTempInt == 0) newCellNumber = -200000000;

**else**{

**for** (**int** counter3 = 0; counter3 < numberLength; counter3++){

            degitExtract = cellNumberExtract.substr((**unsigned** **long**)counter3, 1);

**if** (checkFlag == 0 && degitExtract == "0"){

                zeroPoint = counter3;

                checkFlag = 1;

            }

**else** **if** (checkFlag == 1 && degitExtract != "0"){

                zeroPoint = -1;

                checkFlag = 0;

            }

        }

**if** (zeroPoint != -1){

            degitNumber = numberLength-zeroPoint;

**if** (degitNumber == 8) newCellNumber = cellNumberTempInt+20000000;

**else** **if** (degitNumber == 7) newCellNumber = cellNumberTempInt+2000000;

**else** **if** (degitNumber == 6) newCellNumber = cellNumberTempInt+200000;

**else** **if** (degitNumber == 5) newCellNumber = cellNumberTempInt+20000;

**else** **if** (degitNumber == 4) newCellNumber = cellNumberTempInt+2000;

**else** **if** (degitNumber == 3) newCellNumber = cellNumberTempInt+200;

**else** **if** (degitNumber == 2) newCellNumber = cellNumberTempInt+20;

**else** **if** (degitNumber == 1) newCellNumber = cellNumberTempInt+2;

        }

**else**{

            cellExtensionEntry = 0;

**if** (cellNumberTempInt > 0){

**if** (cellNumberExtract.substr(0, 1) == "0"){

**for** (**int** counter3 = 0; counter3 < cellNoLingNoListCount/2; counter3++){

**if** (cellNoLingNoList [counter3*2] == activeCellStatusList [position*12+3]){

**if** ((to_string(cellNoLingNoList [counter3*2+1])).substr(0, 1) == "3"){

**if** (atoi((to_string(cellNoLingNoList [counter3*2+1])).substr(1, 5).c_str()) > cellExtensionEntry){

                                    cellExtensionEntry = atoi((to_string(cellNoLingNoList [counter3*2+1])).substr(1, 5).c_str());

                                }

                            }

                        }

                    }

                    cellExtensionEntry++;

                    newExtensionEntryNo = to_string(cellExtensionEntry);

**if** (newExtensionEntryNo.length() == 1) newExtensionEntryNo = "30000"+newExtensionEntryNo+"600";

**else** **if** (newExtensionEntryNo.length() == 2) newExtensionEntryNo = "3000"+newExtensionEntryNo+"600";

**else** **if** (newExtensionEntryNo.length() == 3) newExtensionEntryNo = "300"+newExtensionEntryNo+"600";

**else** **if** (newExtensionEntryNo.length() == 4) newExtensionEntryNo = "30"+newExtensionEntryNo+"600";

**else** **if** (newExtensionEntryNo.length() == 5) newExtensionEntryNo = "3"+newExtensionEntryNo+"600";

                    newCellNumber = atoi(newExtensionEntryNo.c_str());

                }

**if** (cellNumberExtract.substr(0, 1) == "1" || cellNumberExtract.substr(0, 1) == "2"){

**for** (**int** counter3 = 0; counter3 < cellNoLingNoListCount/2; counter3++){

**if** (cellNoLingNoList [counter3*2] == activeCellStatusList [position*12+3]){

**if** ((to_string(cellNoLingNoList [counter3*2+1])).substr(0, 1) == "4"){

**if** (atoi((to_string(cellNoLingNoList [counter3*2+1])).substr(1, 5).c_str()) > cellExtensionEntry){

                                    cellExtensionEntry = atoi((to_string(cellNoLingNoList [counter3*2+1])).substr(1, 5).c_str());

                                }

                            }

                        }

                    }

                    cellExtensionEntry++;

                    newExtensionEntryNo = to_string(cellExtensionEntry);

**if** (newExtensionEntryNo.length() == 1) newExtensionEntryNo = "40000"+newExtensionEntryNo+"600";

**else** **if** (newExtensionEntryNo.length() == 2) newExtensionEntryNo = "4000"+newExtensionEntryNo+"600";

**else** **if** (newExtensionEntryNo.length() == 3) newExtensionEntryNo = "400"+newExtensionEntryNo+"600";

**else** **if** (newExtensionEntryNo.length() == 4) newExtensionEntryNo = "40"+newExtensionEntryNo+"600";

**else** **if** (newExtensionEntryNo.length() == 5) newExtensionEntryNo = "4"+newExtensionEntryNo+"600";

                    newCellNumber = atoi(newExtensionEntryNo.c_str());

                }

**if** (cellNumberExtract.substr(0, 1) == "3"){

**for** (**int** counter3 = 0; counter3 < cellNoLingNoListCount/2; counter3++){

**if** (cellNoLingNoList [counter3*2] == activeCellStatusList [position*12+3]){

**if** ((to_string(cellNoLingNoList [counter3*2+1])).substr(0, 1) == "5"){

**if** (atoi((to_string(cellNoLingNoList [counter3*2+1])).substr(1, 5).c_str()) > cellExtensionEntry){

                                    cellExtensionEntry = atoi((to_string(cellNoLingNoList [counter3*2+1])).substr(1, 5).c_str());

                                }

                            }

                        }

                    }

                    cellExtensionEntry++;

                    newExtensionEntryNo = to_string(cellExtensionEntry);

**if** (newExtensionEntryNo.length() == 1) newExtensionEntryNo = "50000"+newExtensionEntryNo+"600";

**else** **if** (newExtensionEntryNo.length() == 2) newExtensionEntryNo = "5000"+newExtensionEntryNo+"600";

**else** **if** (newExtensionEntryNo.length() == 3) newExtensionEntryNo = "500"+newExtensionEntryNo+"600";

**else** **if** (newExtensionEntryNo.length() == 4) newExtensionEntryNo = "50"+newExtensionEntryNo+"600";

**else** **if** (newExtensionEntryNo.length() == 5) newExtensionEntryNo = "5"+newExtensionEntryNo+"600";

                    newCellNumber = atoi(newExtensionEntryNo.c_str());

                }

**if** (cellNumberExtract.substr(0, 1) == "4"){

**for** (**int** counter3 = 0; counter3 < cellNoLingNoListCount/2; counter3++){

**if** (cellNoLingNoList [counter3*2] == activeCellStatusList [position*12+3]){

**if** ((to_string(cellNoLingNoList [counter3*2+1])).substr(0, 1) == "6"){

**if** (atoi((to_string(cellNoLingNoList [counter3*2+1])).substr(1, 5).c_str()) > cellExtensionEntry){

                                    cellExtensionEntry = atoi((to_string(cellNoLingNoList [counter3*2+1])).substr(1, 5).c_str());

                                }

                            }

                        }

                    }

                    cellExtensionEntry++;

                    newExtensionEntryNo = to_string(cellExtensionEntry);

**if** (newExtensionEntryNo.length() == 1) newExtensionEntryNo = "60000"+newExtensionEntryNo+"600";

**else** **if** (newExtensionEntryNo.length() == 2) newExtensionEntryNo = "6000"+newExtensionEntryNo+"600";

**else** **if** (newExtensionEntryNo.length() == 3) newExtensionEntryNo = "600"+newExtensionEntryNo+"600";

**else** **if** (newExtensionEntryNo.length() == 4) newExtensionEntryNo = "60"+newExtensionEntryNo+"600";

**else** **if** (newExtensionEntryNo.length() == 5) newExtensionEntryNo = "6"+newExtensionEntryNo+"600";

                    newCellNumber = atoi(newExtensionEntryNo.c_str());

                }

            }

**else**{

**if** (cellNumberExtract.substr(1, 1) == "0"){

**for** (**int** counter3 = 0; counter3 < cellNoLingNoListCount/2; counter3++){

**if** (cellNoLingNoList [counter3*2] == activeCellStatusList [position*12+3]){

**if** ((to_string(cellNoLingNoList [counter3*2+1])).substr(1, 1) == "3"){

**if** (atoi((to_string(cellNoLingNoList [counter3*2+1])).substr(2, 5).c_str()) > cellExtensionEntry){

                                    cellExtensionEntry = atoi((to_string(cellNoLingNoList [counter3*2+1])).substr(2, 5).c_str());

                                }

                            }

                        }

                    }

                    cellExtensionEntry++;

                    newExtensionEntryNo = to_string(cellExtensionEntry);

**if** (newExtensionEntryNo.length() == 1) newExtensionEntryNo = "-30000"+newExtensionEntryNo+"600";

**else** **if** (newExtensionEntryNo.length() == 2) newExtensionEntryNo = "-3000"+newExtensionEntryNo+"600";

**else** **if** (newExtensionEntryNo.length() == 3) newExtensionEntryNo = "-300"+newExtensionEntryNo+"600";

**else** **if** (newExtensionEntryNo.length() == 4) newExtensionEntryNo = "-30"+newExtensionEntryNo+"600";

**else** **if** (newExtensionEntryNo.length() == 5) newExtensionEntryNo = "-3"+newExtensionEntryNo+"600";

                    newCellNumber = atoi(newExtensionEntryNo.c_str());

                }

**if** (cellNumberExtract.substr(1, 1) == "1" || cellNumberExtract.substr(1, 1) == "2"){

**for** (**int** counter3 = 0; counter3 < cellNoLingNoListCount/2; counter3++){

**if** (cellNoLingNoList [counter3*2] == activeCellStatusList [position*12+3]){

**if** ((to_string(cellNoLingNoList [counter3*2+1])).substr(1, 1) == "4"){

**if** (atoi((to_string(cellNoLingNoList [counter3*2+1])).substr(2, 5).c_str()) > cellExtensionEntry){

                                    cellExtensionEntry = atoi((to_string(cellNoLingNoList [counter3*2+1])).substr(2, 5).c_str());

                                }

                            }

                        }

                    }

                    cellExtensionEntry++;

                    newExtensionEntryNo = to_string(cellExtensionEntry);

**if** (newExtensionEntryNo.length() == 1) newExtensionEntryNo = "-40000"+newExtensionEntryNo+"600";

**else** **if** (newExtensionEntryNo.length() == 2) newExtensionEntryNo = "-4000"+newExtensionEntryNo+"600";

**else** **if** (newExtensionEntryNo.length() == 3) newExtensionEntryNo = "-400"+newExtensionEntryNo+"600";

**else** **if** (newExtensionEntryNo.length() == 4) newExtensionEntryNo = "-40"+newExtensionEntryNo+"600";

**else** **if** (newExtensionEntryNo.length() == 5) newExtensionEntryNo = "-4"+newExtensionEntryNo+"600";

                    newCellNumber = atoi(newExtensionEntryNo.c_str());

                }

**if** (cellNumberExtract.substr(1, 1) == "3"){

**for** (**int** counter3 = 0; counter3 < cellNoLingNoListCount/2; counter3++){

**if** (cellNoLingNoList [counter3*2] == activeCellStatusList [position*12+3]){

**if** ((to_string(cellNoLingNoList [counter3*2+1])).substr(1, 1) == "5"){

**if** (atoi((to_string(cellNoLingNoList [counter3*2+1])).substr(2, 5).c_str()) > cellExtensionEntry){

                                    cellExtensionEntry = atoi((to_string(cellNoLingNoList [counter3*2+1])).substr(2, 5).c_str());

                                }

                            }

                        }

                    }

                    cellExtensionEntry++;

                    newExtensionEntryNo = to_string(cellExtensionEntry);

**if** (newExtensionEntryNo.length() == 1) newExtensionEntryNo = "-50000"+newExtensionEntryNo+"600";

**else** **if** (newExtensionEntryNo.length() == 2) newExtensionEntryNo = "-5000"+newExtensionEntryNo+"600";

**else** **if** (newExtensionEntryNo.length() == 3) newExtensionEntryNo = "-500"+newExtensionEntryNo+"600";

**else** **if** (newExtensionEntryNo.length() == 4) newExtensionEntryNo = "-50"+newExtensionEntryNo+"600";

**else** **if** (newExtensionEntryNo.length() == 5) newExtensionEntryNo = "-5"+newExtensionEntryNo+"600";

                    newCellNumber = atoi(newExtensionEntryNo.c_str());

                }

**if** (cellNumberExtract.substr(1, 1) == "4"){

**for** (**int** counter3 = 0; counter3 < cellNoLingNoListCount/2; counter3++){

**if** (cellNoLingNoList [counter3*2] == activeCellStatusList [position*12+3]){

**if** ((to_string(cellNoLingNoList [counter3*2+1])).substr(1, 1) == "6"){

**if** (atoi((to_string(cellNoLingNoList [counter3*2+1])).substr(2, 5).c_str()) > cellExtensionEntry){

                                    cellExtensionEntry = atoi((to_string(cellNoLingNoList [counter3*2+1])).substr(2, 5).c_str());

                                }

                            }

                        }

                    }

                    cellExtensionEntry++;

                    newExtensionEntryNo = to_string(cellExtensionEntry);

**if** (newExtensionEntryNo.length() == 1) newExtensionEntryNo = "-60000"+newExtensionEntryNo+"600";

**else** **if** (newExtensionEntryNo.length() == 2) newExtensionEntryNo = "-6000"+newExtensionEntryNo+"600";

**else** **if** (newExtensionEntryNo.length() == 3) newExtensionEntryNo = "-600"+newExtensionEntryNo+"600";

**else** **if** (newExtensionEntryNo.length() == 4) newExtensionEntryNo = "-60"+newExtensionEntryNo+"600";

**else** **if** (newExtensionEntryNo.length() == 5) newExtensionEntryNo = "-6"+newExtensionEntryNo+"600";

                    newCellNumber = atoi(newExtensionEntryNo.c_str());

                }

            }

        }

    }

**return** newCellNumber;

}
